# Supplementary material for: Rationally designed Fe-cyclopentadienone with unique orientations for efficient asymmetric hydrogenation of acylsilanes
Source: Nat Commun. 2025 Dec 26;17:1167. doi: 10.1038/s41467-025-67933-9 (PMC12858946; doi:10.1038/s41467-025-67933-9)
Supplement: Supplementary file 1 — Supplementary Information [file 41467_2025_67933_MOESM1_ESM.pdf]

Supplementary Information

Rationally Designed Fe-Cyclopentadienone with Unique Orientations for Efficient Asymmetric Hydrogenation of Acylsilanes

Chaochao Xie[1†], Bo-Xuan Yao[2†], Kwok-Chung Law[1], Xumu Zhang[3], Shao-Fei Ni[2*] & Xuefeng Tan[1*]

[1]Department of Chemistry, City University of Hong Kong, Kowloon Tong, Hong Kong 999077, China

[2]College of Chemistry& Chemical Engineering, Shantou University, Shantou 515063, China

[3]Department of Chemistry, the Grubbs Institute, and Medi-X Pingshan, Southern University of Science and Technology, Shenzhen 518055, Guangdong, China

[†]These authors contributed equally to this work.

**e-mail: xuefetan@cityu.edu.hk; sfni@stu.edu.cn*

Table of Contents

I. General Information 1

II. Synthesis of the Aryacylsilane Substrates 2

III. Synthesis of the Fe Catalyst 21

IV. Reaction Condition Optimization 34

V. Catalytic Asymmetric Hydrogenation of Aromatic Substrates 38

VI. Catalytic Asymmetric Hydrogenation of Alkyl and Alkenyl Substrates 57

VII. Asymmetric Transfer Hydrogenation 66

VIII. Gram-Scale Reaction and Product Derivatizations 72

IX. Mechanistic Studies 75

X. X-ray Crystallography of Fe4 81

XI. Computational Details 83

XII. NMR Spectra 88

XIII. HPLC Traces 188

XIV. References 249

I. General Information

Flash column chromatography was performed over silica gel (200-300 mesh) purchased from Qindao Puke Co., China. All air or moisture sensitive reactions were conducted in oven-dried glassware under nitrogen atmosphere using anhydrous solvents. Anhydrous dichloromethane, diethyl ether, and tetrahydrofuran were purified by the Innovative® solvent purification system or distilled under common conditions. ^{1}H, ^{13}C and ^{19}F NMR spectra were collected on a Bruker AV 300, 400 and 600 MHz NMR spectrometer using residue solvent peaks as an internal standard (^{1}H NMR: $CDCl_3$ at 7.26 ppm; ^{13}C NMR: $CDCl_3$ at 77.0 ppm). Mass spectra were collected on a UV-SH-2 (Shimadzu 1700)-2. Optical rotations were measured on JASCO P-2000 polarimeter with $[\alpha]^{D}$ values reported in degrees; concentration (c) is in 10 mg/mL. The enantiomeric excess values were determined by chiral HPLC using an Agilent 1200 LC instrument with a Daicel CHIRALCEL OD-H or OJ-H column, or a Daicel CHIRALPAK AD-H or AS-H column.

II. Synthesis of the Aryacylsilane Substrates

General Procedure A.

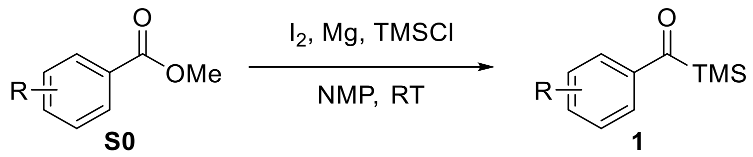

The arylacylsilanes (**1a**, **1d-1j**, **1l-1p**, **1r-1u**) were prepared according to a reported procedure.[1] Magnesium powder (1.2 g, 50.0 mmol), iodine (0.2 g, 0.8 mmol), NMP (60 mL) and excess chlorotrimethylsilane (25 mL, 200.0 mmol) were added and stirred for 15 minutes. Then substituted methyl benzoate **S0** (25.0 mmol) was slowly added and the reaction mixture was stirred for 16 h at room temperature. After compilation, the reaction mixture was quenched with saturated aqueous NH_4Cl (30 mL) and then stir an additional 1 h at room temperature. After this time, the mixture was diluted with pentane and the aqueous layer was extracted with pentane (3 × 100 mL). The combined extracts were washed with brine (10 mL) and dried over Na_2SO_4. After removal of the solvent under reduced pressure the crude material was purified by the column chromatography (eluent: *n*-hexane/EtOAc = 95:5) to yield **1**.

General Procedure B.

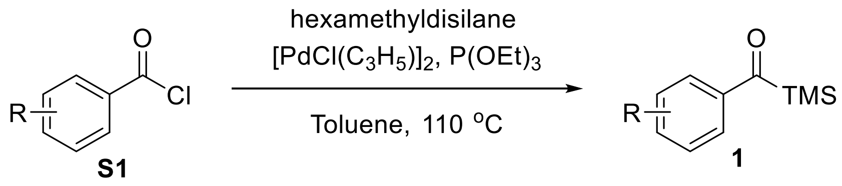

Arylacylsilanes (**1v-1aa**) were prepared according to the literature procedure.[2] Benzoyl chlorides **S1** (5.0 mmol) was added dropwise to a stirred solution of allylpalladium chloride dimer (91.5 mg, 0.25 mmol, 5 mol %), triethyl phosphite (83.1 mg, 0.5 mmol, 10 mol %) and hexamethyldisilane (1.2 g, 8.25 mmol) in toluene (3 mL) at 0 °C and stirred for 5 minutes. Then the mixture was stirred at 110 °C using oil bath overnight. After cooling to room

temperature, the crude product was concentrated under reduced pressure. The residue was purified by flash chromatography on silica gel to afford relevant arylacylsilanes (**1v-1aa**).

General Procedure C.

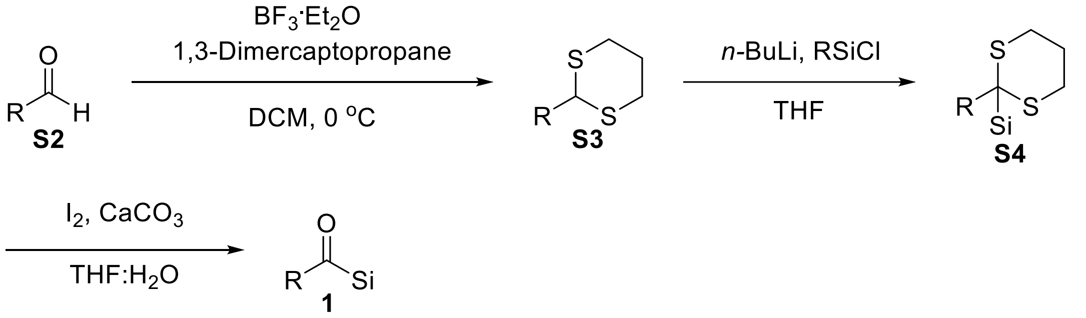

Substrate (**1b, 1c, 1k, 1q, 1ab-1ae, 3m, 3n**) were prepared according to a literature procedure.[3] To a stirred solution of aldehyde (20.0 mmol) and 1,3-propanedithiol (2.4 g, 22.0 mmol) in DCM (0.5 M) at 0 °C was added $BF_3{\cdot}OEt_2$ (3.1 g, 22.0 mmol) dropwise, after complete addition of $BF_3{\cdot}OEt_2$ at 0 °C, the reaction mixture was warmed to room temperature and continued for 3 h. After complete conversion of starting material (monitored by TLC analysis), the reaction was quenched by saturated aqueous $NaHCO_3$. The reaction was extracted by EtOAc three times, and then the combined organic layer was washed with brine and dried over Na_2SO_4. The solvent was removed under reduced pressure and the crude mixture was purified by silica gel column chromatography to yield **S3**.

To an oven dried round-bottom flask equipped with magnetic stir bar was added the above synthesized **S3** (20. 0 mmol) and THF (0.4 M). The resulting solution was kept at -78 °C for 15 minutes under N_2 atmosphere then added *n*-BuLi (24.0 mmol, 2.5 M in hexane) dropwise. The mixture was stirred at -78 °C for 1 h, then silyl chloride (24.0 mmol) was added at the same temperature. After stirring at -78 °C for 1 h, the reaction was gradually warmed to room temperature and stirred for additional 30 minutes. Then, the reaction mixture

was quenched by saturated aqueous NH_4Cl solution and extracted with EtOAc three times. The combined organic layer was washed by brine and dried over Na_2SO_4. The solvent was removed under reduced pressure and the crude mixture was purified by using silica gel column chromatography (*n*-hexanes/ EtOAc = 99:1) to yield **S4**.

To a solution of **S4** (5.25 mmol) in a combined solvent (THF/H_2O = 4:1, 35 mL), $CaCO_3$ (4.2 g, 42.0 mmol) and I_2 (8.1 g, 31.5 mmol) were added at 0 °C. The mixture was stirred for 8 h at room temperature, then quenched with saturated aqueous $Na_2S_2O_3$ (15 mL). The mixture was filtered and washed with EtOAc (15 mL). Then the organic layer was separated and washed with water (20 mL) and brine (20 mL), dried over Na_2SO_4, and concentrated. The residue was purified by silica gel column chromatography to afford relevant product **1**.

General Procedure D.

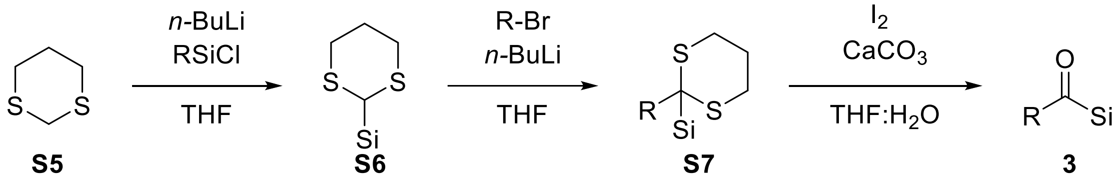

The alkylacylsilanes (**3a-3l**) were prepared according to a reported procedure.[4,5] To a solution of 1,3-dithiane **S5** (20.0 mmol) in anhydrous THF (1.0 M) was added *n*-BuLi (24.0 mmol, 2.5 M in hexane) dropwise at 0 °C under N_2. The resulting solution was stirred for 15 min, silyl chloride (50.0 mmol) was added and the reaction was gradually warmed to room temperature. Then, aqueous HCl (15 mL, 2.0 M) was added, the residue was extracted with diethyl ether (3 × 15 mL). The organic layers were combined, washed with brine, dried over Na_2SO_4 and concentrated. The residue was purified by silica gel column chromatography to yield **S6**.

For the remaining steps from **S6** to **3**, the same procedure with procedure C was adopted.

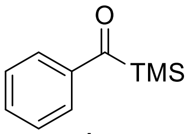

1a

Phenyl(trimethylsilyl)methanone (1a) was prepared as a yellow oil according to the General Procedure A. ^{1}**H NMR** was consistent to the literature report.[1]

^{1}**H NMR** (400 MHz, $CDCl_3$) δ 7.93 – 7.81 (m, 2H), 7.58 – 7.44 (m, 3H), 0.39 (s, 9H) ppm.

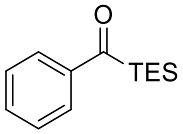

1b

Phenyl(triethylsilyl)methanone (1b) was prepared as a yellow oil according to the General Procedure C. ^{1}**H NMR** was consistent to the literature report.[6]

^{1}**H NMR** (300 MHz, $CDCl_3$) δ 7.83 (dd, *J* = 8.1, 1.63 Hz, 2H), 7.58 – 7.42 (m, 3H), 1.05 – 0.89 (m, 15H) ppm.

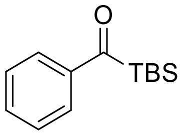

1c

(*tert*-Butyldimethylsilyl)(phenyl)methanone (1c) was prepared as a yellow oil according to the General Procedure C. ^{1}**H NMR** was consistent to the literature report.[7]

^{1}**H NMR** (300 MHz, $CDCl_3$) δ 7.89 – 7.76 (m, 2H), 7.57 – 7.40 (m, 3H), 0.98 (s, 9H), 0.39 (s, 6H) ppm.

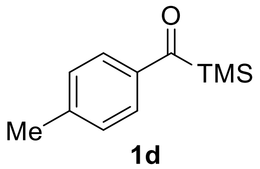

1d

***p*-Tolyl(trimethylsilyl)methanone (1d)** was prepared as a yellow oil according to the General Procedure A. **^{1}H NMR** was consistent to the literature report.[8]

^{1}H NMR (400 MHz, $CDCl_3$) δ 7.77 (d, *J* = 8.2 Hz, 2H), 7.29 (d, *J* = 7.7 Hz, 2H)., 2.42 (s, 3H), 0.39 (s, 9H) ppm.

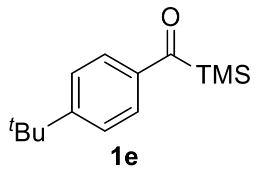

1e

(4-(*tert*-Butyl)phenyl)(trimethylsilyl)methanone (1e) was prepared as a yellow oil according to the General Procedure A. **^{1}H NMR** was consistent to the literature report.[6]

^{1}H NMR (300 MHz, $CDCl_3$) δ 7.83 (d, *J* = 8.5 Hz, 2H), 7.53 (d, *J* = 8.5 Hz, 2H), 1.37 (s, 9H), 0.40 (s, 9H) ppm.

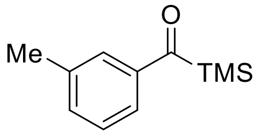

1f

***m*-Tolyl(trimethylsilyl)methanone (1f)** was prepared as a yellow oil according to the General Procedure A. **^{1}H NMR** was consistent to the literature report.[8]

^{1}H NMR (300 MHz, $CDCl_3$) δ 7.70 – 6.63 (m, 2H), 7.38 – 7.29 (m, 2H), 2.41 (s, 3H), 0.38 (s, 9H) ppm.

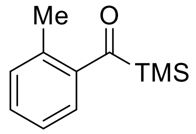

1g

***o*-Tolyl(trimethylsilyl)methanone (1g)** was prepared as a yellow oil according to the General Procedure A. **^{1}H NMR** was consistent to the literature report.[8]

^{1}H NMR (300 MHz, $CDCl_3$) δ 7.66 – 7.54 (m, 1H), 7.40 – 7.21 (m, 3H), 2.44 (s, 3H), 0.34 (d, *J* = 1.3 Hz, 9H) ppm.

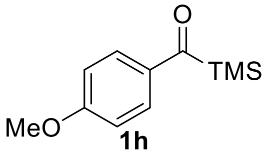

1h

(4-Methoxyphenyl)(trimethylsilyl)methanone (1h) was prepared as a yellow oil according to the General Procedure A. **^{1}H NMR** was consistent to the literature report.[9]

^{1}H NMR (300 MHz, $CDCl_3$) δ 7.85 (d, *J* = 8.8 Hz, 2H), 6.96 (d, *J* = 8.9, 2H), 3.87 (d, *J* = 1.4 Hz, 3H), 0.37 (s, 9H) ppm.

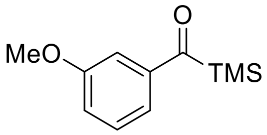

1i

(3-Methoxyphenyl)(trimethylsilyl)methanone (1i) was prepared as a yellow oil according to the General Procedure A. **^{1}H NMR** was consistent to the literature report.[10]

^{1}H NMR (400 MHz, $CDCl_3$) δ 7.5 – 7.45 (m, 1H), 7.39 (t, *J* = 7.8 Hz, 1H), 7.33 (dd, *J* = 2.7, 1.5 Hz, 1H), 7.09 (dd, *J* = 8.1, 1.8 Hz, 1H), 3.84 (s, 3H), 0.38 (s, 9H) ppm.

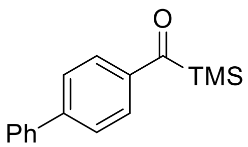

1j

[1,1'-Biphenyl]-4-yl(trimethylsilyl)methanone (1j) was prepared as a yellow oil according to the General Procedure A. **^{1}H NMR** was consistent to the literature report.[8]

¹H NMR (300 MHz, $CDCl_3$) δ 8.04 – 7.97 (m, 2H), 7.81 – 7.72 (m, 2H), 7.72 – 7.66 (m, 2H), 7.55 – 7.44 (m, 3H), 0.48 (s, 9H) ppm.

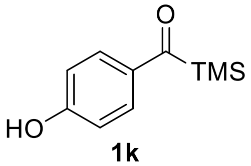

1k

(4-Hydroxyphenyl)(trimethylsilyl)methanone (1k) was prepared as a yellow oil according to the General Procedure C. **¹H NMR** was consistent to the literature report.[11]

¹H NMR (300 MHz, $CDCl_3$) δ 9.10 (s, 1H), 7.84 (d, J = 8.7 Hz, 2H), 7.04 (d, J = 8.7 Hz, 2H), 0.40 (s, 9H) ppm.

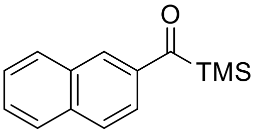

1l

Naphthalen-2-yl(trimethylsilyl)methanone (1l) was prepared as a yellow oil according to the General Procedure A. **¹H NMR** was consistent to the literature report.[8]

¹H NMR (400 MHz, $CDCl_3$) δ 8.53 – 8.35 (m, 1H), 8.06 – 7.99 (m, 1H), 7.99 – 7.87 (m, 3H), 7.65 – 7.55 (m, 2H), 0.50 (s, 9H) ppm.

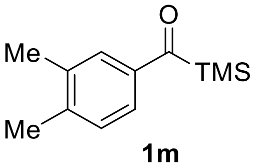

1m

(3,4-Dimethylphenyl)(trimethylsilyl)methanone (1m) was prepared as a yellow oil according to the General Procedure A. **¹H NMR** was consistent to the literature report.[8]

¹H NMR (300 MHz, $CDCl_3$) δ 7.66 – 7.59 (m, 2H), 7.25 (d, J = 8.3 Hz, 1H), 2.38 –

2.28 (m, 6H), 0.39 (s, 9H) ppm.

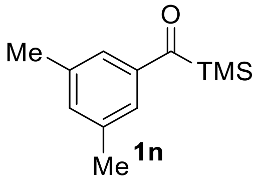
1n

(3,5-Dimethylphenyl)(trimethylsilyl)methanone (1n) was prepared as a yellow oil according to the General Procedure A. ^{1}**H NMR** was consistent to the literature report.[6]

^{1}**H NMR** (300 MHz, $CDCl_3$) δ 7.46 (s, 2H), 7.19 (s, 1H), 2.39 (s, 6H), 0.39 (s, 9H) ppm.

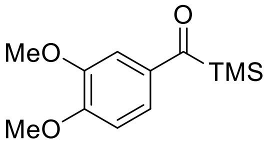
1o

(3,4-Dimethoxyphenyl)(trimethylsilyl)methanone (1o) was prepared as a yellow oil according to the General Procedure A. ^{1}**H NMR** was consistent to the literature report.[11]

^{1}**H NMR** (300 MHz, $CDCl_3$) δ 7.61 – 7.53 (m, 1H), 7.38 (t, J = 1.66 Hz, 1H), 7.01 – 6.86 (m, 1H), 3.97 (d, J = 2.4 Hz, 3H), 3.94 (d, J = 2.0 Hz, 3H), 0.39 (s, 9H) ppm.

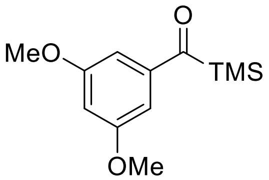
1p

(3,5-Dimethoxyphenyl)(trimethylsilyl)methanone (1p) was prepared as a yellow oil according to the General Procedure A. ^{1}**H NMR** was consistent to the literature report.[11]

^{1}**H NMR** (300 MHz, $CDCl_3$) δ 6.96 (t, J = 2.0 Hz, 2H), 6.62 – 6.56 (m, 1H), 3.95 – 3.63 (m, 6H), 0.35 (s, 9H) ppm.

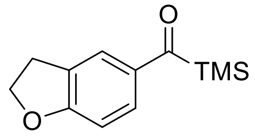

1q

(2,3-Dihydrobenzofuran-5-yl)(trimethylsilyl)methanone (1q) was prepared as a yellow oil according to the General Procedure C.

^{1}H NMR (300 MHz, $CDCl_3$) δ 7.75 – 7.67 (m, 2H), 6.81 (dd, *J* = 8.9, 1.6 Hz, 1H), 4.69 – 4.57 (m, 2H), 3.24 (t, *J* = 8.67 Hz, 2H), 0.36 (s, 9H) ppm.

^{13}C NMR (75 MHz, $CDCl_3$) δ 232.3, 164.0, 135.7, 130.6, 127.9, 123.9, 108.9, 72.1, 28.9, -1.1 ppm.

HR-MS (ESI) *m/z* Calcd. for $C_{12}H_{16}NaO_2Si^+$ $[M+Na]^+$: 243.0812, found: 243.0814.

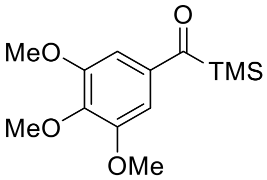

1r

(3,4,5-Trimethoxyphenyl)(trimethylsilyl)methanone (1r) was prepared as a yellow oil according to the General Procedure A. **^{1}H NMR** was consistent to the literature report.[12]

^{1}H NMR (300 MHz, $CDCl_3$) δ 7.12 (d, *J* = 1.1 Hz, 2H), 3.90 (d, *J* = 1.2 Hz, 9H), 0.37 (d, *J* = 1.1 Hz, 9H) ppm.

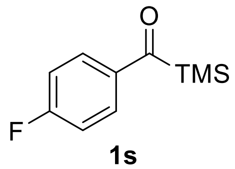

1s

(4-Fluorophenyl)(trimethylsilyl)methanone (1s) was prepared as a yellow oil according to the General Procedure A. **^{1}H NMR** was consistent to the literature report.[6]

^{1}H NMR (300 MHz, $CDCl_3$) δ 7.86 (dd, *J* = 8.7, 5.6 Hz, 2H), 7.13 (t, *J* = 8.6 Hz,

2H), 0.37 (s, 9H) ppm.

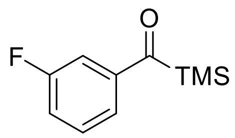

1t

(3-Fluorophenyl)(trimethylsilyl)methanone (1t) was prepared as a yellow oil according to the General Procedure A. ^{1}**H NMR** was consistent to the literature report.[6]

^{1}**H NMR** (300 MHz, $CDCl_3$) δ 7.70 – 7.59 (m, 1H), 7.50 – 7.41 (m, 2H), 7.28 – 7.16 (m, 1H), 0.39 (s, 9H) ppm.

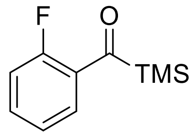

1u

(2-Fluorophenyl)(trimethylsilyl)methanone (1u) was prepared as a yellow oil according to the General Procedure A. ^{1}**H NMR** was consistent to the literature report.[13]

^{1}**H NMR** (300 MHz, $CDCl_3$) δ 7.62 – 7.43 (m, 2H), 7.21 (t, *J* = 7.5 Hz, 1H), 7.12 (dd, *J* = 10.6, 1.1 Hz, 1H), 0.30 (d, *J* = 2.63 Hz, 9H) ppm.

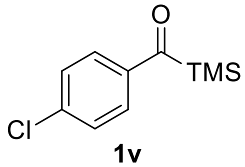

1v

(4-Chlorophenyl)(trimethylsilyl)methanone (1v) was prepared as a yellow oil according to the General Procedure B. ^{1}**H NMR** was consistent to the literature report.[6]

^{1}**H NMR** (300 MHz, $CDCl_3$) δ 7.79 (d, *J* = 8.6 Hz, 2H), 7.45 (d, *J* = 8.5 Hz, 2H), 0.38 (s, 9H) ppm.

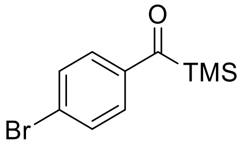

1w

(4-Bromophenyl)(trimethylsilyl)methanone (1w) was prepared as a yellow oil according to the General Procedure B. ^{1}**H NMR** was consistent to the literature report.[6]

^{1}**H NMR** (300 MHz, $CDCl_3$) δ 7.70 (d, *J* = 8.6 Hz, 2H), 7.61 (d, *J* = 8.6 Hz, 2H), 0.37 (s, 9H) ppm.

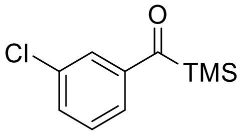

1x

(3-Chlorophenyl)(trimethylsilyl)methanone (1x) was prepared as a yellow oil according to the General Procedure B. ^{1}**H NMR** was consistent to the literature report.[8]

^{1}**H NMR** (400 MHz, $CDCl_3$) δ 7.77 (t, *J* = 1.9 Hz, 1H), 7.73 (dt, *J* = 7.5, 1.4 Hz, 1H), 7.54 – 7.48 (m, 1H), 7.43 (t, *J* = 7.74 Hz, 1H), 0.39 (s, 9H) ppm.

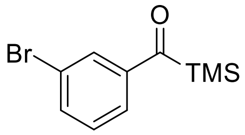

1y

(3-Bromophenyl)(trimethylsilyl)methanone (1y) was prepared as a yellow oil according to the General Procedure B. ^{1}**H NMR** was consistent to the literature report.[14]

^{1}**H NMR** (400 MHz, $CDCl_3$) δ 7.92 (d, *J* = 1.8 Hz, 1H), 7.78 (d, *J* = 7.7 Hz, 1H), 7.69 – 7.64 (m, 1H), 7.38 (t, *J* = 7.8 Hz, 1H), 0.39 (s, 9H) ppm.

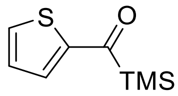

1z

Thiophen-2-yl(trimethylsilyl)methanone (1z) was prepared as a yellow oil according to the General Procedure B. ^{1}**H NMR** was consistent to the literature report.[11]

^{1}**H NMR** (300 MHz, $CDCl_3$) δ 7.76 (dd, *J* = 3.8, 1.1 Hz, 1H), 7.64 (dd, *J* = 4.9, 1.1 Hz, 1H), 7.16 (dd, *J* = 5.0, 3.8 Hz, 1H), 0.38 (s, 9H) ppm.

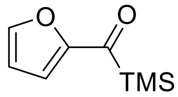

1aa

Furan-2-yl(trimethylsilyl)methanone (1aa) was prepared as a yellow oil according to the General Procedure B. ^{1}**H NMR** was consistent to the literature report.[10]

^{1}**H NMR** (300 MHz, $CDCl_3$) δ 7.61 (dd, *J* = 1.7 Hz, 1H), 7.09 (dd, *J* = 3.6 Hz, 1H), 6.55 (dd, *J* = 3.6, 1.7 Hz, 1H), 0.35 (s, 9H) ppm.

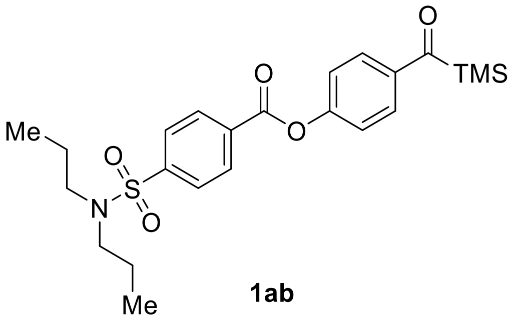

1ab

4-((Trimethylsilyl)carbonyl)phenyl 4-(*N,N*-dipropylsulfamoyl)benzoate (1ab) was prepared as a yellow oil according to the General Procedure C.

^{1}**H NMR** (400 MHz, $CDCl_3$) δ 8.34 (d, *J* = 8.5 Hz, 2H), 8.00 – 7.91 (m, 4H), 7.36 (d, *J* = 8.6 Hz, 2H), 3.24 – 3.05 (m, 4H), 1.68 – 1.49 (m, 4H), 0.90 (t, *J* = 7.4 Hz, 6H), 0.41 (s, 9H) ppm.

^{13}**C NMR** (75 MHz, $CDCl_3$) δ 234.1, 163.4, 153.8, 145.1, 139.2, 132.3, 130.9, 129.2,

127.2, 121.9, 49.9, 21.9, 11.1, -1.3 ppm.

HR-MS (ESI) *m/z* Calcd. for $C_{23}H_{31}NNaO_5SSi^+$ [M+Na]$^+$: 484.1584, found: 484.1584.

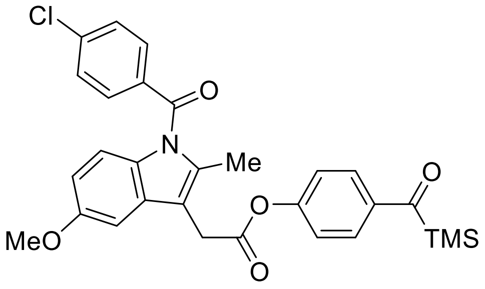

1ac

4-((Trimethylsilyl)carbonyl)phenyl 2-(1-(4-chlorobenzoyl)-5-methoxy-2-methyl-1*H*-indol-3-yl)acetate (1ac) was prepared as a yellow oil according to the General Procedure C.

^{1}H NMR (300 MHz, $CDCl_3$) δ 7.88 (d, *J* = 8.6 Hz, 2H), 7.70 (d, *J* = 8.5 Hz, 2H), 7.49 (d, *J* = 8.5 Hz, 2H), 7.21 (d, *J* = 8.6 Hz, 2H), 7.08 (d, *J* = 2.5 Hz, 1H), 6.91 (d, *J* = 9.0 Hz, 1H), 6.72 (dd, *J* = 9.0, 2.5 Hz, 1H), 3.96 (s, 2H), 3.86 (s, 3H), 2.49 (s, 3H), 0.38 (s, 9H) ppm.

^{13}C NMR (75 MHz, $CDCl_3$) δ 234.2, 168.8, 168.3, 156.1, 153.9, 139.4, 138.9, 136.3, 133.7, 131.2, 130.8, 130.4, 129.2, 129.1, 121.7, 115.1, 111.8, 111.6, 101.1, 55.7, 30.6, 13.4, -1.3 ppm.

HR-MS (ESI) *m/z* Calcd. for $C_{29}H_{28}ClNNaO_5Si^+$ [M+Na]$^+$: 556.1317, found: 556.1317.

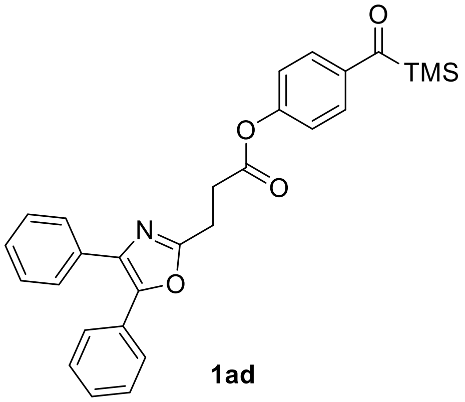

1ad

4-((Trimethylsilyl)carbonyl)phenyl 3-(4,5-diphenyloxazol-2-yl)propanoate (1ad) was prepared as a yellow oil according to the General Procedure C.

^{1}H NMR (300 MHz, $CDCl_3$) δ 7.94 – 7.86 (m, 2H), 7.73 – 7.66 (m, 2H), 7.66 – 7.58 (m, 2H), 7.47 – 7.30 (m, 6H), 7.30 – 7.22 (m, 2H), 3.39 – 3.28 (m, 2H), 3.27 – 3.15 (m, 2H), 0.40 (s, 9H) ppm.

^{13}C NMR (75 MHz, $CDCl_3$) δ 234.1, 190.9, 170.2, 161.3, 153.9, 145.6, 138.9, 135.1, 132.3, 131.2, 126.5 (2C), 121.9, 31.2, 23.4, -1.3 ppm.

HR-MS (ESI) *m/z* Calcd. for $C_{28}H_{27}NNaO_4Si^+$ $[M+Na]^+$: 492.1602, found: 492.1610.

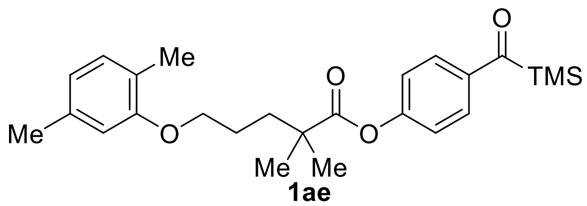

1ae

4-((Trimethylsilyl)carbonyl)phenyl 5-(2,5-dimethylphenoxy)-2,2-dimethyl pentanoate (1ae) was prepared as a yellow oil according to the General Procedure C**.**

^{1}H NMR (300 MHz, $CDCl_3$) δ 7.90 (d, *J* = 8.6 Hz, 2H), 7.17 (d, *J* = 8.6 Hz, 2H), 7.04 (d, *J* = 7.4 Hz, 1H), 6.75 – 6.61 (m, 2H), 4.03 (t, *J* = 4.8 Hz, 2H), 2.34 (s, 3H), 2.20 (s, 3H), 1.99 – 1.84 (m, 4H), 1.42 (s, 6H), 0.41 (s, 9H) ppm.

^{13}C NMR (75 MHz, $CDCl_3$) δ 234.1, 176.0, 156.8, 154.4, 138.8, 136.5, 130.4, 129.1, 123.6, 121.9, 120.8, 111.9, 67.6, 42.6, 37.1, 25.3, 25.1, 21.5, 15.9, -1.3 ppm.

HR-MS (ESI) *m/z* Calcd. for $C_{25}H_{35}O_4Si^+$ $[M+H]^+$: 427.2299, found: 427.2294.

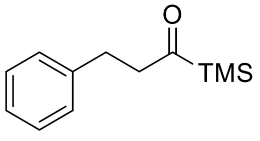

3a

3-Phenyl-1-(trimethylsilyl)propan-1-one (3a) was prepared as a colorless oil according to the General Procedure D. ^{1}H NMR was consistent to the literature

report.[9]

^{1}H NMR (300 MHz, $CDCl_3$) δ 7.35 – 7.27 (m, 2H), 7.25 – 7.16 (m, 3H), 3.02 – 2.94 (m, 2H), 2.91 – 2.83 (m, 2H), 0.23 (s, 9H) ppm.

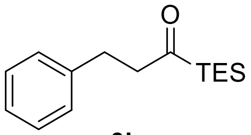

3b

3-Phenyl-1-(triethylsilyl)propan-1-one (3b) was prepared as a colorless oil according to the General Procedure D. **^{1}H NMR** was consistent to the literature report.[3]

^{1}H NMR (300 MHz, $CDCl_3$) δ 7.37 – 7.25 (m, 2H), 7.25 – 7.15 (m, 3H), 3.02 – 2.80 (m, 4H), 1.09 – 0.91 (m, 9H), 0.86 – 0.62 (m, 6H) ppm.

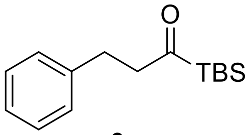

3c

1-(*tert*-Butyldimethylsilyl)-3-phenylpropan-1-one (3c) was prepared as a colorless oil according to the General Procedure D. **^{1}H NMR** was consistent to the literature report.[15]

^{1}H NMR (300 MHz, $CDCl_3$) δ 7.34 – 7.25 (m, 2H), 7.24 – 7.15 (m, 3H), 2.98 – 2.90 (m, 2H), 2.89 – 2.81 (m, 2H), 0.94 (s, 9H), 0.19 (s, 6H) ppm.

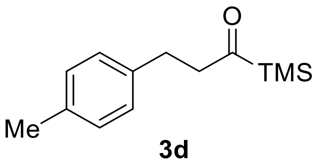

3d

3-(*p*-Tolyl)-1-(trimethylsilyl)propan-1-one (3d) was prepared as a colorless oil according to the General Procedure D.

^{1}H NMR (300 MHz, $CDCl_3$) δ 7.20 – 7.03 (m, 4H), 2.98 – 2.91 (m, 2H), 2.90 – 2.75 (m, 2H), 2.36 (s, 3H), 0.24 (s, 9H) ppm.

^{13}C NMR (75 MHz, $CDCl_3$) δ 247.2, 138.5, 135.4, 129.1, 128.2, 50.3, 27.8, 21.0, -3.2 ppm.

HR-MS (ESI) *m/z* Calcd. for $C_{13}H_{20}NaOSi^+$ [M+Na]$^+$: 243.1176, found: 243.1181.

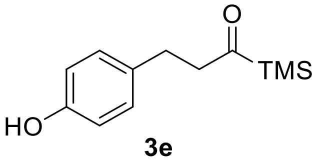

3e

3-(4-Hydroxyphenyl)-1-(trimethylsilyl)propan-1-one (3e) was prepared as a colorless oil according to the General Procedure D.

^{1}H NMR (300 MHz, $CDCl_3$) δ 7.03 (d, *J* = 8.5 Hz, 2H), 6.80 (d, *J* = 8.6 Hz, 2H), 6.22 (s, 1H), 3.00 – 2.90 (m, 2H), 2.84 – 2.74 (m, 2H), 0.21 (s, 9H) ppm.

^{13}C NMR (75 MHz, $CDCl_3$) δ 249.3, 154.0, 133.2, 129.4, 115.3, 50.4, 27.4, -3.2 ppm.

HR-MS (ESI) *m/z* Calcd. for $C_{12}H_{18}NaO_2Si^+$ [M+Na]$^+$: 245.0968, found: 245.0974.

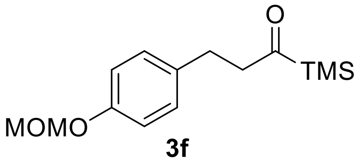

3f

3-(4-(Methoxymethoxy)phenyl)-1-(trimethylsilyl)propan-1-one (3f) was prepared as a colorless oil according to the General Procedure D.

^{1}H NMR (300 MHz, $CDCl_3$) δ 7.10 (d, *J* = 8.6 Hz, 2H), 6.97 (d, *J* = 8.6 Hz, 2H), 5.16 (s, 2H), 3.48 (s, 3H), 3.00 – 2.85 (m, 2H), 2.85 – 2.72 (m, 2H), 0.21 (s, 9H) ppm.

^{13}C NMR (75 MHz, $CDCl_3$) δ 247.2, 155.5, 135.0, 129.3, 116.3, 94.5, 55.9, 50.3, 27.3, -3.2 ppm.

HR-MS (ESI) *m/z* Calcd. for $C_{14}H_{22}NaO_3Si^+$ [M+Na]$^+$: 289.1230, found: 289.1238.

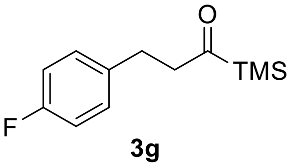

3g

3-(4-Fluorophenyl)-1-(trimethylsilyl)propan-1-one (3g) was prepared as a

colorless oil according to the General Procedure D.

^{1}H NMR (400 MHz, $CDCl_3$) δ 7.12 (dd, J = 8.5, 5.5 Hz, 2H), 7.00-6.90 (m, 2H), 2.98 – 2.86 (m, 2H), 2.85 – 2.74 (m, 2H), 0.19 (s, 9H).

^{13}C NMR (75 MHz, $CDCl_3$) δ 247.0, 161.3 (d, $^{1}J_{C\text{-}F}$ = 243.5 Hz), 137.3 (d, $^{4}J_{C\text{-}F}$ = 3.2 Hz), 129.7 (d, $^{3}J_{C\text{-}F}$ = 7.8 Hz), 115.2 (d, $^{2}J_{C\text{-}F}$ = 21.1 Hz), 50.0, 27.4, -3.3 ppm.

^{19}F NMR (282 MHz, $CDCl_3$) δ -117.5 (s) ppm.

HR-MS (ESI) *m/z* Calcd. for $C_{12}H_{17}FNaOSi^{+}$ $[M+Na]^{+}$: 247.0925, found: 247.0929.

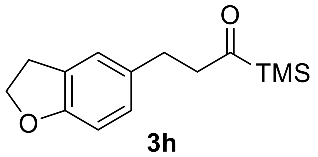

3h

3-(2,3-Dihydrobenzofuran-5-yl)-1-(trimethylsilyl)propan-1-one (3h) was prepared as a colorless oil according to the General Procedure D.

^{1}H NMR (300 MHz, $CDCl_3$) δ 7.02 (d, J = 1.9 Hz, 1H), 6.94 – 6.87 (m, 1H), 6.71 (d, J = 8.1 Hz, 1H), 4.56 (t, J = 8.7 Hz, 2H), 3.19 (t, J = 8.7 Hz, 2H), 2.96 – 2.85 (m, 2H), 2.82 – 2.70 (m, 2H), 0.21 (s, 9H) ppm.

^{13}C NMR (75 MHz, $CDCl_3$) δ 247.6, 158.3, 133.5, 127.6, 127.1, 124.9, 109.1, 71.2, 50.7, 29.8, 27.6, -3.2 ppm.

HR-MS (ESI) *m/z* Calcd. for $C_{14}H_{20}NaO_2Si^{+}$ $[M+Na]^{+}$: 271.1125, found: 271.1124.

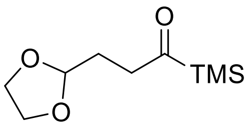

3i

3-(1,3-Dioxolan-2-yl)-1-(trimethylsilyl)propan-1-one (3i) was prepared as a colorless oil according to the General Procedure D.

^{1}H NMR (300 MHz, $CDCl_3$) δ 4.85 – 4.75 (m, 1H), 3.93 – 3.80 (m, 2H), 3.80 – 3.70 (m, 2H), 2.74 – 2.56 (m, 2H), 1.90 – 1.77 (m, 2H), 0.14 (s, 9H) ppm.

^{13}C NMR (75 MHz, $CDCl_3$) δ 246.4, 103.5, 64.8, 42.0, 25.9, -3.2 ppm.

HR-MS (ESI) *m/z* Calcd. for $C_9H_{18}NaO_3Si^+$ [M+Na]$^+$: 225.0917, found: 225.0919.

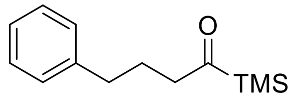

3j

4-Phenyl-1-(trimethylsilyl)butan-1-one (3j) was prepared as a colorless oil according to the General Procedure D. ^{1}**H NMR** was consistent to the literature report.[16]

^{1}**H NMR** (400 MHz, $CDCl_3$) δ 7.35 – 7.27 (m, 2H), 7.25 – 7.16 (m, 3H), 2.70 – 2.56 (m, 4H), 1.97 – 1.79 (m, 2H), 0.22 (s, 9H) ppm.

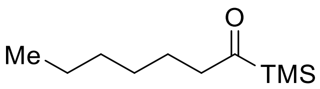

3k

1-(Trimethylsilyl)heptan-1-one (3k) was prepared as a colorless oil according to the General Procedure D. ^{1}**H NMR** was consistent to the literature report.[17]

^{1}**H NMR** (400 MHz, $CDCl_3$) δ 2.67 – 2.48 (m, 2H), 1.56 – 1.42 (m, 2H), 1.35 – 1.15 (m, 6H), 0.91 – 0.78 (m, 3H), 0.18 (t, *J* = 1.2 Hz, 9H) ppm.

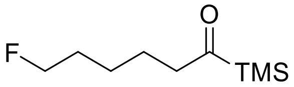

3l

5-Fluoro-1-(trimethylsilyl)pentan-1-one (3l) was prepared as a colorless oil according to the General Procedure D.

^{1}**H NMR** (300 MHz, $CDCl_3$) δ 4.51 – 4.37 (m, 1H), 4.37 – 4.21 (m, 1H), 2.69 – 2.47 (m, 2H), 1.74 – 1.44 (m, 4H), 1.40 – 1.24 (m, 2H), 0.15 (s, 9H) ppm.

^{13}**C NMR** (75 MHz, $CDCl_3$) δ 248.2, 83.9 (d, $^1J_{C\text{-}F}$ = 164.2 Hz), 48.2, 30.3 (d, $^2J_{C\text{-}F}$ = 19.5 Hz), 24.9 (d, $^3J_{C\text{-}F}$ = 5.3 Hz), 21.2, -3.2 ppm.

^{19}**F NMR** (282 MHz, $CDCl_3$) δ -218.37 (s) ppm.

HR-MS (ESI) *m/z* Calcd. for $C_9H_{19}FNaOSi^+$ [M+Na]$^+$: 213.1081, found: 213.1086.

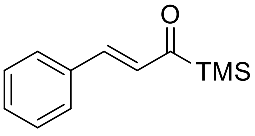

3m

(*E*)-3-Phenyl-1-(trimethylsilyl)prop-2-en-1-one (3m) was prepared as a colorless oil according to the General Procedure C. **^{1}H NMR** was consistent to the literature report.[16]

^{1}H NMR (400 MHz, $CDCl_3$) δ 7.60 – 7.53 (m, 2H), 7.46 (d, *J* = 16.4 Hz, 1H), 7.43 – 7.38 (m, 3H), 6.91 (d, *J* = 16.5 Hz, 1H), 0.34 (s, 9H) ppm.

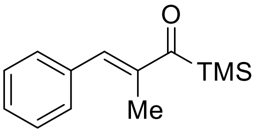

3n

(*E*)-2-Methyl-3-phenyl-1-(trimethylsilyl)prop-2-en-1-one (3n) was prepared as a colorless oil according to the General Procedure C. **^{1}H NMR** was consistent to the literature report.[18]

^{1}H NMR (300 MHz, $CDCl_3$) δ 7.53 – 7.32 (m, 6H), 2.00 (d, *J* = 1.5 Hz, 3H), 0.38 (s, 9H) ppm.

III. Synthesis of the Fe Catalyst

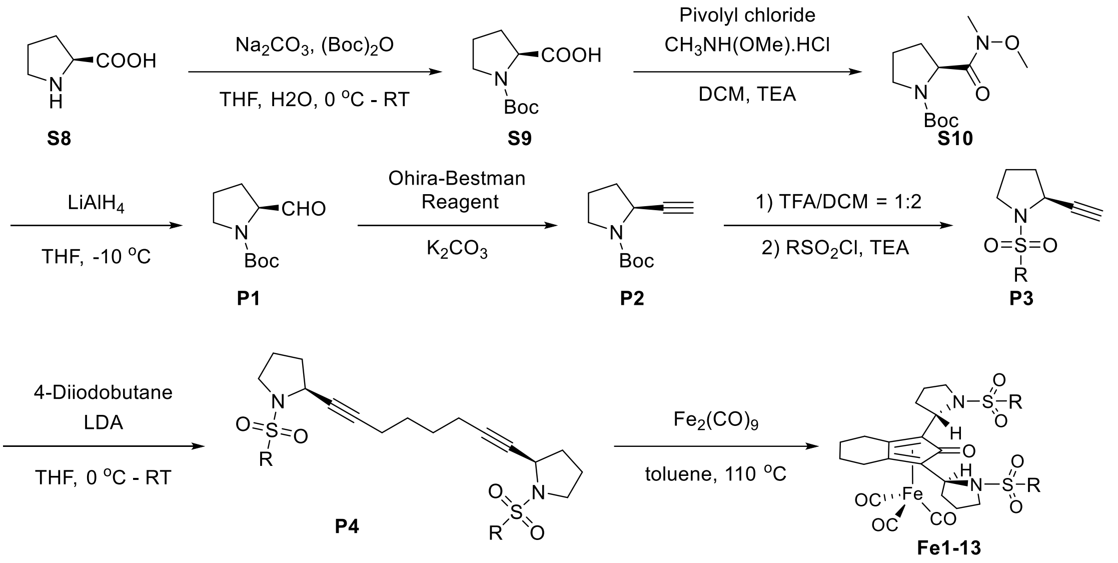

Compound **S9** was prepared according to a reported procedure.[19] To a suspension of *L*-proline (11.5 g, 115.0 mmol) in THF/H_2O (1:1), sodium bicarbonate (15.0 g, 130.0 mmol) was added and stirred at room temperature for 30 min, then Boc_2O (30.0 g, 136.5 mmol) was added and stirred for 12 h. The reaction mixture was monitored by TLC. The reaction mixture was concentrated under reduced pressure. The residue was adjusted pH-2 by using aqueous HCl solution. The aqueous layer was extracted with EtOAc (3 × 20 mL), washed with water and brine. The combined organic layer was dried over Na_2SO_4, filtered and concentrated under reduced pressure to get the desired product.

Compound **S10** was prepared according to a reported procedure.[20] To a stirred solution of *N*-Boc proline **S9** (8.1 g, 37.8 mmol) in dry DCM (c = 0.4 M, 95 mL) at 0 °C, triethylamine (4.2 g, 41.5 mmol) was added. After stirring for 15 minutes, trimethylacetyl chloride (4.8 g, 39.6 mmol) was added. After stirring for another 1 h at the same temperature, *N,O*-dimethylhydroxylamine hydrochloride (3.9 g, 39.6 mmol) was added in one pot, followed by dropwise addition of triethylamine (8.0 g, 79.3 mmol). The reaction mixture was stirred for another 1.5 h at 0 °C and subjected to dilute aqueous HCl workup. Flash

chromatography on a short pad silica gel (eluent: *n*-hexane/EtOAc = 1:1) afforded Weinreb amide **S10** (9.4 g, 96% yield) as colorless viscous oil.

Compound **P1** was prepared according to a reported procedure.[21] To a flame dried flask that was purged with nitrogen and cooled to -20 °C, was added a solution of *tert*-butyl (*S*)-2-(methoxy(methyl)carbamoyl)pyrrolidine-1-carboxylate **S10** (3.9 g, 15.1 mmol) in anhydrous THF (25 mL). $LiAlH_4$ (0.6 g, 15.1 mmol) was added carefully and the reaction was stirred under nitrogen for 30 minutes at -20 °C. The reaction was cautiously quenched with water at -20 °C. The organic layer was extracted with EtOAc three times, washed with 1 M HCl and then brine, and dried over Na_2SO_4. The solution was concentrated under reduced pressure to obtain the product **P1** (2.6 g, 86% yield) as a colourless oil.

Compound **P2** was prepared according to a reported procedure.[21] A solution of dimethyl (1-diazo-2-oxopropyl)phosphonate (3.9 g, 20.1 mmol), potassium carbonate (3.5 g, 25.2 mmol) and methanol (20 mL) was cooled to -10 °C and stirred for 30 minutes. In a separate flask, a solution of *tert*-butyl (*S*)-2-formylpyrrolidine-1-carboxylate **P1** (3.3 g, 16.8 mmol) in methanol (3 mL) was also cooled to -10 °C for 30 minutes and then added dropwise to the reaction mixture. The reaction was stirred for 1 h and then at room temperature for 12 h. The reaction was quenched with saturated aqueous $NaHCO_3$ and the organic layer was extracted with DCM three times and dried over Na_2SO_4. The product was concentrated under reduced pressure, and flash column chromatography on silica gel to afford the desired product **S12** (eluent: *n*-hexane/EtOAc = 5:1→ 3:1, 3.1 g, 81% yield. The overall yield from **S8** is 63.5%).

To a solution of **P2** (2.9 g, 15.0 mmol) in DCM (10 mL), was slowly add trifluoroacetic acid (5 mL) dropwise. The reaction was stirred for 5 h at room temperature. Then triethylamine was slowly added to the reaction mixture at 0 °C. Finally, corresponding RSO_2Cl (18.0 mmol) was added to the reaction. Completion of the reaction was monitored by TLC. The reaction was quenched with H_2O, the mixture was extracted with DCM three times and dried over

Na_2SO_4. The product was purified by flash column chromatography on silica gel to afford the desired product **P3** (eluent: *n*-hexane/EtOAc = 10:1→5:1, the overall yields ranged from 60–80%).

Under N_2 at 0 °C, to a solution of the above **P3** (5.0 mmol, 3.0 equiv.) in THF (5 mL) was added LDA (2.5 mL, 5.0 mmol, 2.0 M in hexane, 3.0 equiv.) dropwise. The reaction mixture was stirred at 0 °C for 0.5 h before a solution of 1,4-diiodobutane (0.5 g, 1.7 mmol, 1.0 equiv.) in THF (2 mL) was added. The reaction was slowly warmed to room temperature and stirred overnight. Then the reaction mixture was poured into a saturated aqueous NH_4Cl solution (10 mL) and extracted with EtOAc (10 mL × 3). The combined organic phases were dried over anhydrous Na_2SO_4, and filtered. The filtrate was concentrated under reduced pressure. The residue was purified by flash column chromatography on silica gel to afford the desired product **P4** (eluent: *n*-hexane/EtOAc = 5:1→3:1, the overall yields ranged from 50–70%).

Diiron nonacarbonyl (8.0 mmol) and the compound **P4** (4.0 mmol) were charged in a flame-dried Schlenk tube under inert atmosphere. Toluene (0.1 M) was added and the mixture was heated to 110 °C for 12 - 18 h. The reaction was cooled to room temperature, filtered through celite and rinsed with DCM. The corresponding products were purified via silica gel column chromatography.

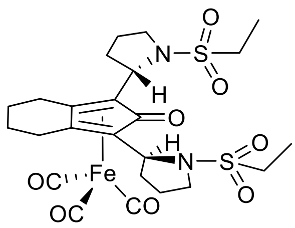

Fe1

Fe1 was prepared as a yellow solid. (12 h, eluent: *n*-hexane/EtOAc = 5:1→3:1, 58% yield. The overall yield from P2 is 18.8%). $[\alpha]_D^{25}$: -145.3 (*c* = 1.0, $CHCl_3$).

^{1}H NMR (300 MHz, $CDCl_3$) δ 4.63 (t, *J* = 7.39 Hz, 1H), 4.39 (t, *J* = 8.11 Hz, 1H), 3.86 (t, *J* = 8.22 Hz, 1H), 3.69 – 3.52 (m, 3H), 3.25 – 3.00 (m, 3H), 2.98 – 2.79 (m,

3H), 2.67 – 2.51 (m, 1H), 2.50 – 2.26 (m, 3H), 2.24 – 2.11 (m, 1H), 2.10 – 2.00 (m, 2H), 1.94 – 1.81 (m, 5H), 1.33 (t, *J* = 7.31 Hz, 3H), 1.26 (t, *J* = 7.33 Hz, 5H) ppm.

^{13}**C NMR** (75 MHz, $CDCl_3$) δ 208.7, 169.6, 103.6, 100.8, 83.8, 82.1, 53.7, 53.5, 50.9, 50.7, 48.6, 42.2, 36.9, 36.9, 27.3, 24.9, 22.0, 21.9, 21.9, 21.7, 8.2, 7.5 ppm.

HR-MS (ESI) m/z Calcd. for $C_{24}H_{33}FeN_2O_8S_2^+$ [M+H]+: 597.1022, found: 597.1014.

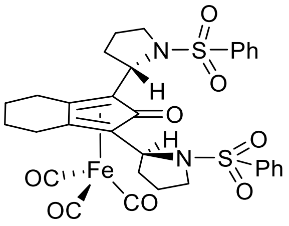

Fe2

Fe2 was prepared as a yellow solid. (12 h, eluent: *n*-hexane/EtOAc = 5:1→3:1, 69% yield. The overall yield from **P2** is 33.2%). $[\alpha]_D^{25}$: -98.6 (*c* = 1.0, $CHCl_3$).

^{1}**H NMR** (300 MHz, $CDCl_3$) δ 7.93 (d, *J* = 7.01 Hz, 2H), 7.83 (d, *J* = 6.96 Hz, 3H), 7.67 – 7.35 (m, 7H), 4.44 (t, *J* = 7.69 Hz, 1H), 4.37 (t, *J* = 6.99 Hz, 1H), 3.76 – 3.5 (m, 3H), 3.45 – 3.26 (m, 1H), 3.00 – 2.86 (m, 1H), 2.83 – 2.70 (m, 1H), 2.68 – 2.43 (m, 2H), 2.33 – 2.19 (m, 1H), 2.18 – 2.00 (m, 1H), 1.95 – 1.72 (m, 8H), 1.54 – 1.37 (m, 1H), 1.36 – 1.26 (m, 1H) ppm.

^{13}**C NMR** (75 MHz,$CDCl_3$) δ 208.7, 169.0, 139.2, 136.8, 132.9, 132.6, 129.2, 129.1, 127.9, 127.4, 102.1, 99.6, 85.6, 83.0, 55.6, 55.1, 50.7, 50.0, 35.3, 35.2, 26.5, 24.9, 21.9, 21.8 (3C) ppm.

HR-MS (ESI) m/z Calcd. for $C_{32}H_{33}FeN_2O_8S_2^+$ [M+H]+: 693.1022, found: 693.1016.

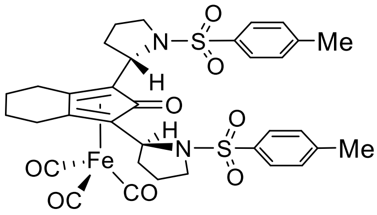

Fe3

Fe3 was prepared as a yellow solid. (12 h, eluent: *n*-hexane/EtOAc = 5:1→3:1, 75% yield. The overall yield from **P2** is 31%). $[\alpha]_D^{25}$: -179.9 (*c* = 1.0, $CHCl_3$).

^{1}H NMR (400 MHz, $CDCl_3$) δ 7.82 (d, *J* = 8.2 Hz, 2H), 7.73 (d, *J* = 8.3 Hz, 2H), 7.34 (d, *J* = 7.9 Hz, 2H), 7.31 – 7.22 (m, 2H), 4.45 (t, *J* = 7.7 Hz, 1H), 4.38 (t, *J* = 7.0 Hz, 1H), 3.74 – 3.56 (m, 3H), 3.43 – 3.31 (m, 1H), 3.03 – 2.89 (m, 1H), 2.85 – 2.74 (m, 1H), 2.70 – 2.49 (m, 2H), 2.44 (s, 3H), 2.39 (s, 3H), 2.32 – 2.18 (m, 1H), 2.17 – 2.06 (m, 1H), 1.93 – 1.77 (m, 7H), 1.51 – 1.22 (m, 3H) ppm.

^{13}C NMR (100 MHz, $CDCl_3$) δ 208.7, 169.1, 143.6, 143.3, 136.2, 133.9, 129.8, 129.7, 127.9, 127.4, 102.0, 99.5, 85.7, 83.2, 55.6, 55.1, 50.6, 50.0, 35.2, 35.2, 26.4, 24.9, 21.9, 21.8 (3C), 21.6, 21.5 ppm.

HR-MS (ESI) *m/z* Calcd. for $C_{34}H_{36}FeN_2NaO_8S_2^+$ $[M+Na]^+$: 743.1155, found: 743.1157.

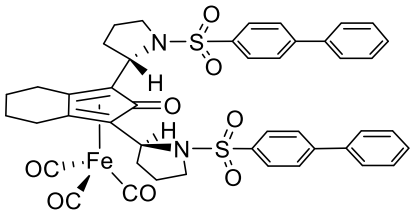

Fe4

Fe4 cat. was prepared as a yellow solid. (12 h, eluent: *n*-hexane/EtOAc = 5:1→ 3:1, 78% yield. The overall yield from **P2** is 36.2%). $[\alpha]_D^{25}$: -230.2 (*c* = 1.0, $CHCl_3$).

^{1}H NMR (400 MHz, $CDCl_3$) δ 7.97 (d, *J* = 8.4 Hz, 2H), 7.90 (d, *J* = 8.4 Hz, 2H), 7.70 (dd, *J* = 16.8, 8.4 Hz, 4H), 7.62 – 7.58 (m, 2H), 7.57 – 7.53 (m, 2H), 7.48 (t, *J* = 7.6 Hz, 2H), 7.46 – 7.39 (m, 3H), 7.39 – 7.33 (m,1H), 4.53 (t, *J* = 7.7 Hz, 1H), 4.44 (t, *J* = 7.2 Hz, 1H), 3.79 – 3.71 (m, 1H), 3.68 – 3.55 (m, 2H), 3.45 – 3.35 (m, 1H), 3.06 – 2.95 (m, 1H), 2.86 (dt, *J* = 17.1, 5.8 Hz, 1H), 2.73 – 2.53 (m, 2H), 2.38 – 2.26 (m, 1H), 2.24 – 2.14 (m, 1H), 2.03 – 1.95 (m, 1H), 1.94 – 1.73 (m, 7H), 1.62 – 1.53 (m, 1H), 1.42 – 1.32 (m, 1H) ppm.

^{13}C NMR (100 MHz, $CDCl_3$) δ 208.7, 169.1, 145.5, 145.2, 139.22, 139.18, 137.9, 135.3, 129.1, 129.0, 128.54, 128.51, 128.4, 128.0, 127.7, 127.6, 127.3, 127.2, 102.2, 99.8, 85.5, 83.1, 55.5, 55.1, 50.6, 50.1, 35.6, 35.4, 26.6, 24.8, 22.02, 21.99, 21.87, 21.85, ppm.

HR-MS (ESI) *m/z* Calcd. for $C_{44}H_{40}FeN_2NaO_8S_2^+$ [M+Na]$^+$: 867.1468, found: 867.1458.

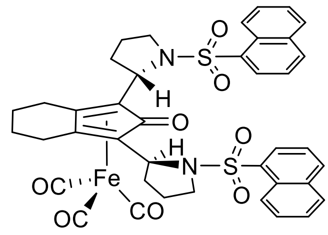

Fe5

Fe5 was prepared as a yellow solid. (12 h, eluent: *n*-hexane/EtOAc = 3:1→1:1, 61% yield. The overall yield from **P2** is 24.5%). $[\alpha]_D^{25}$: -168.5 (c = 1.0, $CHCl_3$).

^{1}H NMR (300 MHz, $CDCl_3$) δ 9.00 (d, *J* = 8.4 Hz, 1H), 8.73 (d, *J* = 8.6 Hz, 1H), 8.30 – 8.12 (m, 2H), 8.06 (d, *J* = 8.2 Hz, 1H), 8.01 – 7.86 (m, 2H), 7.82 (dd, *J* = 8.2, 1.35 Hz, 1H), 7.70 – 7.42 (m, 6H), 4.61 (t, *J* = 8.1 Hz, 1H), 4.54 (dd, *J* = 7.5, 5.4 Hz, 1H), 4.06 – 3.93 (m, 1H), 3.76 (td, *J* = 10.7, 5.5 Hz, 1H), 3.54 (t, *J* = 6.6 Hz, 2H), 3.05 – 2.87 (m, 1H), 2.62 – 2.36 (m, 4H), 2.29 – 2.13 (m, 1H), 1.80 – 1.52 (m, 7H), 1.38 – 1.25 (m, 2H), 1.19 – 1.09 (m, 1H) ppm.

^{13}C NMR (75 MHz, $CDCl_3$) δ 208.6, 168.4, 136.2, 134.4, 134.3 (2C), 133.9, 133.5, 130.4, 129.2, 128.9, 128.7, 128.6 (2C), 128.1, 128.0, 126.8, 126.8, 125.3, 125.2, 124.5, 124.0, 102.3, 99.3, 86.9, 81.8, 55.2, 54.4, 51.2, 49.6, 35.6, 35.0, 27.0, 25.4, 21.9, 21.7 (2C), 21.6 ppm.

HR-MS (ESI) *m/z* Calcd. for $C_{40}H_{37}FeN_2O_8S_2^+$ [M+H]$^+$: 793.1335, found: 793.1337.

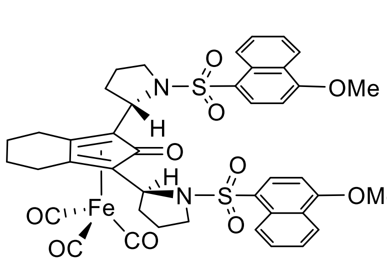

Fe6

Fe6 cat. was prepared as a yellow solid. (12 h, eluent: *n*-hexane/EtOAc = 3:1→ 1:1, 49% yield. The overall yield from **P2** is 19.3%). $[\alpha]_D^{25}$: -143.1 (c = 1.0, $CHCl_3$).

^{1}H NMR (400 MHz, $CDCl_3$) δ 8.91 (d, *J* = 8.6 Hz, 1H), 8.67 (d, *J* = 8.6 Hz, 1H),

8.34 (d, *J* = 8.4 Hz, 1H), 8.26 (d, *J* = 8.4 Hz, 1H), 8.18 (t, *J* = 8.4 Hz, 2H), 7.72 – 7.57 (m, 2H), 7.57 – 7.44 (m, 2H), 6.83 (dd, *J* = 8.4, 2.6 Hz, 2H), 4.69 – 4.58(m, 2H), 4.06 (s, 3H), 3.97 (s, 3H), 3.91 (t, *J* = 17.9, 1H), 3.76 – 3.63 (m, 1H), 3.62 – 3.45 (m, 2H), 3.04 – 2.84 (m, 1H), 2.66 – 2.35 (m, 4H), 2.28 – 2.13 (m, 1H), 2.12 – 1.94 (m, 1H), 1.80 – 1.54 (m, 7H), 1.53 – 1.38 (m, 1H), 1.26 – 1.17 (m, 1H) ppm.

^{13}C NMR (100 MHz, $CDCl_3$) δ 208.7, 168.7, 159.4 (2C), 132.4, 130.6, 130.4, 129.9, 128.5, 128.4, 127.7, 126.1, 126.0 (2C), 125.2, 125.0, 124.9, 122.7 (2C), 122.5, 102.6, 102.3, 102.0, 99.6, 87.0, 82.1, 56.0, 55.9, 54.8, 54.3, 51.1, 49.6, 35.56, 35.50, 27.0, 25.4, 21.9 (2C), 21.7 (2C) ppm.

HR-MS (ESI) *m/z* Calcd. for $C_{42}H_{41}FeN_2O_{10}S_2^+$ $[M+H]^+$: 853.1547, found: 853.1535.

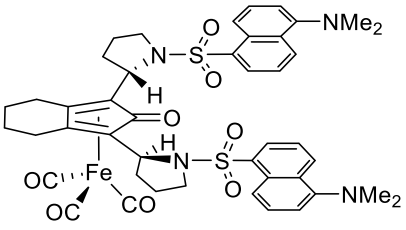

Fe7

Fe7 cat. was prepared as a yellow solid. (12 h, eluent: *n*-hexane/EtOAc = 2:1→ 1:3, 54% yield. The overall yield from **P2** is 18%). $[\alpha]_D^{25}$: -359.7 (*c* = 1.0, $CHCl_3$).

^{1}H NMR (300 MHz, $CDCl_3$) δ 8.66 (d, *J* = 8.7 Hz, 1H), 8.54 (d, *J* = 8.2 Hz, 1H), 8.41 (dd, *J* = 16.2, 8.5 Hz, 2H), 8.27 – 8.12 (m, 2H), 7.61 – 7.40 (m, 4H), 7.21 – 7.04 (m, 2H), 4.71 – 4.54 (m, 2H), 3.99 – 3.88 (m, 1H), 3.74 (td, *J* = 10.7, 5.4 Hz, 1H), 3.64 – 3.47 (m, 2H), 3.05 – 2.97 (m, 1H), 2.88 (s, 6H), 2.80 (s, 6H), 2.61 – 2.39 (m, 4H), 2.29 – 2.18 (m, 1H), 2.11 – 2.00 (m, 1H), 1.83 – 1.53 (m, 7H), 1.48 – 1.33 (m, 1H), 1.26 – 1.13 (m, 1H) ppm.

^{13}C NMR (75 MHz, $CDCl_3$) δ 208.6, 168.4, 151.5, 151.4, 136.3, 133.9, 130.6, 130.3, 130.2, 130.1, 130.1, 130.0, 129.9, 128.6, 128.0, 127.9, 123.5, 123.1, 119.8, 119.7, 115.2, 115.1, 102.4, 99.4, 87.1, 81.9, 55.3, 54.4, 51.2, 49.7, 45.4 (2C), 35.5, 34.9, 27.1, 25.4, 21.9, 21.7 (3C) ppm.

HR-MS (ESI) *m/z* Calcd. for $C_{44}H_{47}FeN_4O_8S_2^+$ $[M+H]^+$: 879.2179, found: 879.2165.

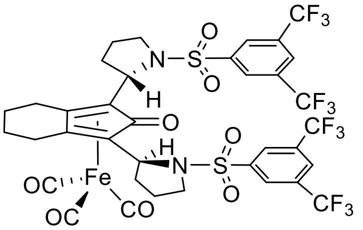

Fe8

Fe8 cat. was prepared as a yellow solid. (12 h, eluent: *n*-hexane/EtOAc = 5:1→ 3:1, 62% yield. The overall yield from **P2** is 34.1%). $[\alpha]_D^{25}$: -121.0 (c = 1.0, $CHCl_3$).

^{1}H NMR (300 MHz, $CDCl_3$) δ 8.39 (s, 2H), 8.25 (d, J = 3.9 Hz, 2H), 8.10 (s, 1H), 7.98 (s, 1H), 4.47 (t, J = 8.0 Hz, 1H), 4.26 (t, J = 6.8 Hz, 1H), 3.85 (t, J = 8.7 Hz, 1H), 3.79 – 3.57 (m, 2H), 3.44 – 3.25 (m, 1H), 3.18 – 2.98 (m, 1H), 2.69 – 2.56 (m, 2H), 2.50 – 2.39 (m, 1H), 2.35 – 2.23 (m, 1H), 2.21 – 2.06 (m, 2H), 2.02 – 1.70 (m, 7H), 1.67 – 1.51 (m, 1H), 1.50 – 1.34 (m, 1H) ppm.

^{13}C NMR (75 MHz, $CDCl_3$) δ 208.2, 168.9, 143.4, 140.4, 132.9 (q, $^2J_{C\text{-}F}$ = 34.7 Hz), 132.6 (q, $^2J_{C\text{-}F}$ = 34.4 Hz), 128.2, 127.3, 126.38, 126.03, 122.5 (q, $^1J_{C\text{-}F}$ = 273.6 Hz), 122.5 (q, $^1J_{C\text{-}F}$ = 271.8 Hz), 101.7, 100.1, 84.5, 81.3, 55.9, 54.9, 51.0, 50.2, 36.5, 34.5, 27.0, 25.0, 22.0, 21.8, 21.6, 21.6 ppm.

^{19}F NMR {^{1}H} (282 MHz, $CDCl_3$) δ -62.8, -62.9 ppm.

HR-MS (ESI) m/z Calcd. for $C_{36}H_{28}F_{12}FeN_2NaO_8S_2^+$ $[M+Na]^+$: 987.0337, found: 987.0328.

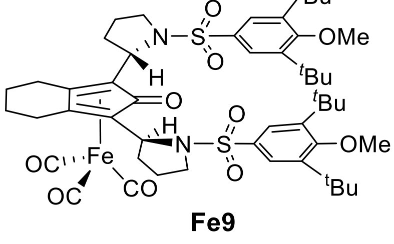

Fe9

Fe9 cat. was prepared as a yellow solid. (12 h, eluent: *n*-hexane/EtOAc = 5:1→ 3:1, 73% yield. The overall yield from **P2** is 46%). $[\alpha]_D^{25}$: -140.4 (c = 1.0, $CHCl_3$).

^{1}H NMR (400 MHz, $CDCl_3$) δ 7.76 (s, 2H), 7.74 (s, 2H), 4.29 – 4.19 (m, 2H), 3.72 (s, 7H), 3.68 – 3.58 (m, 2H), 3.53 – 3.39 (m, 1H), 3.16 –3.02 (m, 1H), 2.72 – 2.61

(m, 2H), 2.60 – 2.50 (m, 1H), 2.37 – 2.27 (m, 1H), 2.00 – 1.80 (m, 8H), 1.49 –1.41 (m, 36H), 1.33 – 1.26 (m, 2H), 1.18 – 1.01 (m, 1H) ppm.

^{13}C NMR (100 MHz, $CDCl_3$) δ 208.8, 169.0, 163.4, 163.3, 145.3, 145.1, 132.4, 131.5, 126.3, 125.9, 101.6, 99.6, 86.5, 82.4, 64.7, 64.7, 56.3, 55.2, 51.1, 50.3, 36.1 (2C), 34.6, 33.9, 31.8, 31.8, 26.1, 25.2, 22.0, 21.9, 21.8, 21.4 ppm.

HR-MS (ESI) *m/z* Calcd. for $C_{50}H_{69}FeN_2O_{10}S_2^+$ [M+H]$^+$: 977.3738, found: 977.3742.

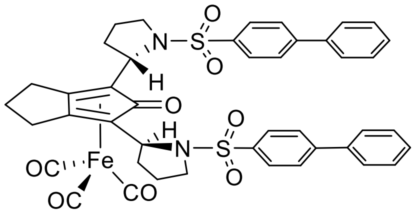

Fe10

Fe10 cat. was prepared as a yellow solid. (12 h, eluent: *n*-hexane/EtOAc = 5:1→ 3:1, 74% yield. The overall yield from **P2** is 42%). $[\alpha]_D^{25}$: -165.9 (*c* = 1.0, $CHCl_3$).

^{1}H NMR (400 MHz, $CDCl_3$) δ 7.89 (t, *J* = 9.6 Hz, 4H), 7.79 (d, *J* = 8.0 Hz, 2H), 7.73 – 7.56 (m, 6H), 7.56 – 7.35 (m, 6H), 4.62 (dt, *J* = 25.2, 6.7 Hz, 2H), 3.71 – 3.38 (m, 3H), 3.31 – 3.17 (m, 1H), 3.15 – 3.02 (m, 1H), 2.97 – 2.72 (m, 3H), 2.56 – 2.33 (m, 1H), 2.26 – 2.08 (m, 1H), 2.08 – 1.76 (m, 6H), 1.66 (dd, *J* = 12.9, 6.6 Hz, 1H), 1.52 – 1.37 (m, 1H) ppm.

^{13}C NMR (100 MHz, $CDCl_3$) δ 208.6, 172.2, 145.8, 145.4, 139.2 (2C), 136.6, 134.7, 129.1 (2C), 128.5 (3C), 128.1, 127.7 (2C), 127.4, 127.3 , 107.6, 106.1, 86.4, 83.4, 56.5, 56.3, 49.9, 49.5, 35.0, 34.1, 27.7, 26.4 (2C), 25.7, 24.0 ppm.

HR-MS (ESI) *m/z* Calcd. for $C_{43}H_{38}FeN_2NaO_8S_2^+$ [M+Na]$^+$: 853.1311, found: 853.1307.

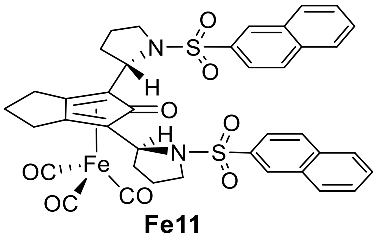

Fe11

Fe11 cat. was prepared as a yellow solid. (12 h, eluent: *n*-hexane/EtOAc = 5:1→ 3:1, 68% yield. The overall yield from **P2** is 37%). $[\alpha]_D^{25}$: -202.5 (c = 1.0, $CHCl_3$).

^{1}H NMR (400 MHz, $CDCl_3$) δ 8.44 (d, J = 5.1 Hz, 2H), 8.02 (q, J = 6.7 Hz, 4H), 7.96 – 7.84 (m, 4H), 7.74 – 7.52 (m, 4H), 4.75 (t, J = 8.01 Hz, 1H), 4.69 (t, J = 5.8 Hz, 1H), 3.68 – 3.43 (m, 3H), 3.27 (dd, J = 11.0, 6.8 Hz, 1H), 3.10 – 2.98 (m, 1H), 2.95 – 2.76 (m, 3H), 2.51 – 2.38 (m, 1H), 2.2 – 2.06 (m, 1H), 2.00 – 1.82 (m, 5H), 1.82 – 1.70 (m, 1H), 1.62 – 1.47 (m, 1H), 1.43 – 1.34 (m, 1H) ppm.

^{13}C NMR (100 MHz, $CDCl_3$) δ 208.6, 172.3, 135.0, 134.9, 134.8, 133.3, 132.2, 132.1, 129.6, 129.5 (2C), 129.4 (2C), 128.9 (2C), 128.8, 127.9 (2C), 127.7, 127.6, 123.2, 123.0, 107.4, 105.8, 86.3, 83.9, 56.8, 56.4, 49.9, 49.5, 34.9, 33.8, 31.6, 27.6, 26.5, 25.6, 24.0, 22.7, 14.2 ppm.

HR-MS (ESI) *m/z* Calcd. for $C_{39}H_{34}FeN_2NaO_8S_2^+$ $[M+Na]^+$: 801.0998, found: 801.0998.

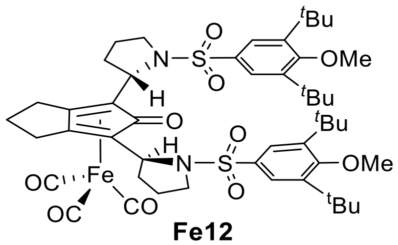

Fe12

Fe12 cat. was prepared as a yellow solid. (12 h, eluent: *n*-hexane/EtOAc = 5:1→ 3:1, 75% yield. The overall yield from **P2** is 40%). $[\alpha]_D^{25}$: -127.4 (c = 1.0, $CHCl_3$).

^{1}H NMR (300 MHz, $CDCl_3$) δ 7.76 (s, 2H), 7.74 (s, 2H), 4.30 – 4.18 (m, 2H), 3.72 (s, 7H), 3.65 (dd, J = 10.0, 4.12 Hz, 2H), 3.54 –3.42 (m, 1H), 3.16 – 3.01 (m, 1H), 2.70 – 2.51 (m, 3H), 2.41 – 2.25 (m, 1H), 2.04 – 1.76 (m, 8H), 1.51 – 1.40 (m, 36H), 1.18 – 1.10 (m, 1H) ppm.

^{13}C NMR (100 MHz, $CDCl_3$) δ 208.8, 172.4, 163.6, 163.4, 145.3 (2C), 131.5, 130.6, 126.4, 126.0, 107.7, 106.1, 87.0, 82.9, 64.7 (2C), 56.9, 56.5, 50.4, 49.3, 36.12, 36.09, 34.3, 33.2, 31.8, 31.8, 27.7, 26.5, 26.4, 25.5, 23.9 ppm.

HR-MS (ESI) *m/z* Calcd. for $C_{49}H_{66}FeN_2NaO_{10}S_2^+$ $[M+Na]^+$: 985.3401, found:

985.3399.

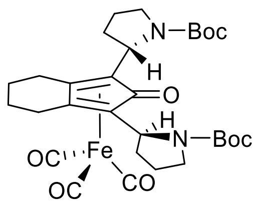

Fe 13

Fe13 was prepared as a yellow solid. (12 h, eluent: *n*-hexane/EtOAc = 5:1→3:1, 61% yield. The overall yield from **P2** is 27.5%). $[\alpha]_D^{25}$: -94.7 (*c* = 1.0, $CHCl_3$).

^{1}H NMR (300 MHz, $CDCl_3$) δ 4.90 – 4.09 (m, 2H), 3.70 – 3.01 (m, 4H), 2.73 – 1.44 (m, 34H) ppm.

^{13}C NMR (75 MHz, $CDCl_3$) δ 209.3, 169.5, 168.9, 154.5, 101.2, 85.2, 82.9, 80.0, 79.0 (2C), 53.4, 52.0, 48.7, 47.8, 38.8, 35.3, 33.2, 29.6, 25.6, 24.9, 22.0, 21.5 ppm.

HR-MS (ESI) *m/z* Calcd. for $C_{30}H_{41}FeN_2O_8^+$ $[M+H]^+$: 613.2207, found: 613.2200.

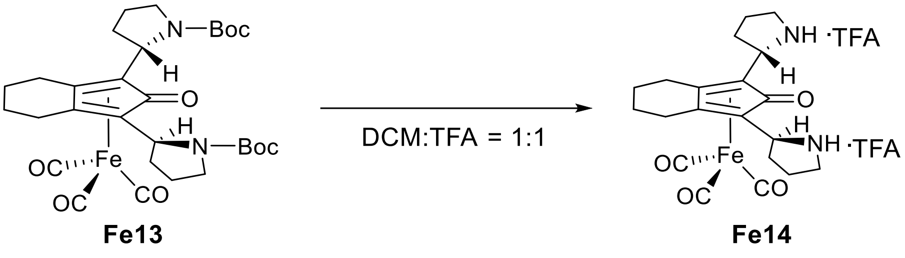

To a solution of **Fe13** (200 mg, 0.33 mmol) in DCM (3 mL), was slowly add trifluoroacetic acid (3 mL) dropwise. The reaction was stirred for 5 h at room temperature. Then removal of the solvent under reduced pressure to afford the desired product as a yellow solid. (120 mg, 88% yield.). $[\alpha]_D^{25}$: -44.4 (*c* = 1.0, MeOH).

Due to the rotamers of Boc-N moiety, the NMR spectra are very complex.

^{1}H NMR (300 MHz, MeOD) δ 4.18 – 3.97 (m, 2H), 3.50 – 3.33 (m, 4H), 2.64 – 2.24 (m, 6H), 2.22 – 1.84 (m, 6H), 1.83 – 1.61 (m, 4H) ppm.

^{13}C NMR (75 MHz, MeOD) δ 206.0, 169.1, 102.4, 101.4, 74.5 (2C), 57.0, 53.9, 46.9, 45.7, 30.5, 30.0, 24.8, 24.2, 20.9, 20.8, 20.3 ppm.

HR-MS (ESI) m/z Calcd. for $C_{20}H_{25}FeN_2O_4^+$ [M+H]+: 413.1158, found: 413.1159.

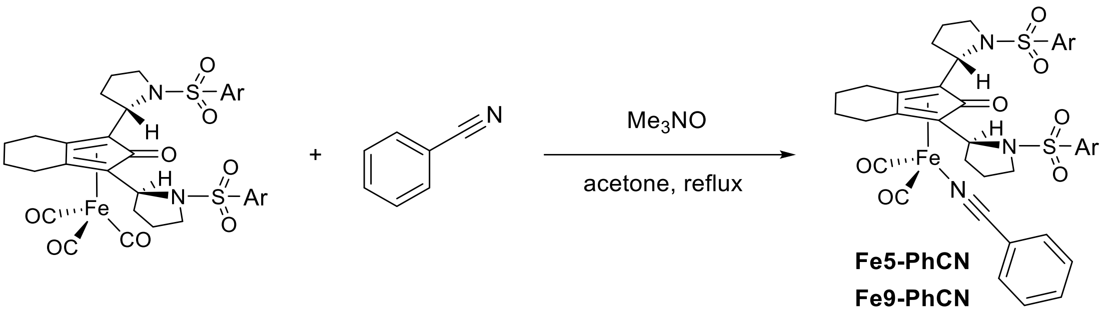

A solution of **Fe** compound (0.1 mmol, 1.0 equiv), benzonitrile (22 mg, 0.2 mmol, 2.0 equiv), and anhydrous Me_3NO (10 mg, 0.12 mmol, 1.2 equiv) in acetone (4 mL) stirred at reflux for 20 hours. The orange/brown reaction was diluted with 15 mL of water and was extracted with 3 × 10 mL of DCM. The combined organic layers were dried over anhydrous Na_2SO_4, filtered, and the solvent was evaporated to afford a solid. The corresponding products were purified via silica gel column chromatography.

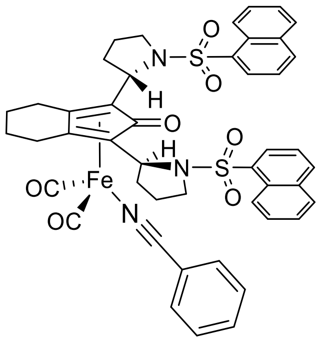

Fe5-PhCN

Fe5-PhCN was prepared as a yellow solid. (20 h, eluent: *n*-hexane/EtOAc = 3:1 →1:1, 65% yield). $[\alpha]_D^{25}$: -190.5 (c = 1.0, $CHCl_3$).

^{1}H NMR (300 MHz, $CDCl_3$) δ 8.91 (dd, J = 45.27, 7.48 Hz, 2H), 8.34 – 8.14 (m, 2H), 8.04 (d, J = 7.64 Hz, 1H), 7.97 – 7.84 (m, 2H), 7.84 – 7.69 (m, 3H), 7.68 – 7.34 (m, 9H), 4.86 – 4.61 (m, 2H), 4.02 – 3.69 (m, 2H), 3.67 – 3.45 (m, 2H), 2.69 – 2.39 (m, 2H), 2.38 – 2.15 (m, 2H), 2.14 – 1.88 (m, 3H), 1.71 – 1.26 (m, 9H) ppm.

^{13}C NMR (75 MHz, $CDCl_3$) δ 212.5, 166.4, 136.5, 134.3, 134.2, 134.1, 134.1, 133.6, 133.4, 132.9 (2C), 130.4, 129.1, 129.1, 128.8, 128.8, 128.7, 128.5, 127.9 (2C), 126.7,

126.6, 125.39, 125.36, 124.5, 124.3, 111.9, 98.0, 94.2, 84.5, 78.8, 55.7, 55.4, 50.8, 49.5, 34.9, 32.7, 26.8, 25.3, 21.7, 21.5 (3C) ppm.

HR-MS (ESI) *m*/*z* Calcd. for $C_{46}H_{42}FeN_3O_7S_2^+$ $[M+H]^+$: 868.1808, found: 868.1783.

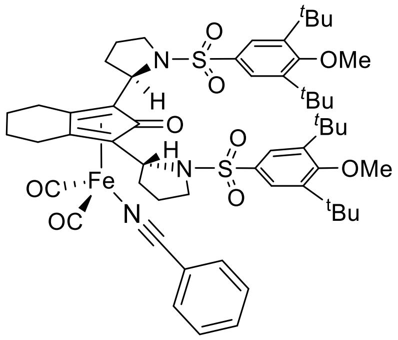

Fe9-PhCN

Fe9-PhCN was prepared as a yellow solid. (20 h, eluent: *n*-hexane/EtOAc = 5:1 →3:1, 75% yield). $[\alpha]_D^{25}$: 178.5 (*c* = 1.0, $CHCl_3$).

¹H NMR (300 MHz, $CDCl_3$) δ 7.85 (d, *J* = 7.21 Hz, 2H), 7.78 – 7.66 (m, 4H), 7.65 – 7.53 (m, 1H), 7.51 – 7.39 (m, 2H), 4.48 – 4.24 (m, 2H), 3.78 – 3.55 (m, 9H), 3.42 – 3.29 (m, 1H), 2.88 – 2.66 (m, 1H), 2.56 – 2.27 (m, 4H), 1.99 – 1.60 (m, 8H), 1.44 (d, *J* = 5.13 Hz, 36H), 1.26 – 1.16 (m, 2H) 1.16 – 0.99 (m, 1H) ppm.

¹³C NMR (75 MHz, $CDCl_3$) δ 212.8, 167.0, 163.1, 145.1, 145.0, 133.0 (3C), 131.8, 129.0, 126.2 (2C), 125.9 (2C), 112.4, 97.0, 94.6, 83.3, 79.9, 64.7, 57.3, 56.2, 50.8 50.2, 36.1 (3C), 33.4, 31.8, 25.8, 24.7, 21.9 (2C), 21.3 ppm.

HR-MS (ESI) *m*/*z* Calcd. for $C_{56}H_{74}FeN_3O_9S_2^+$ $[M+H]^+$: 1052.4210, found: 1052.4183.

IV. Reaction Condition Optimization

Supplementary Table 1. Screening of catalysts[a]

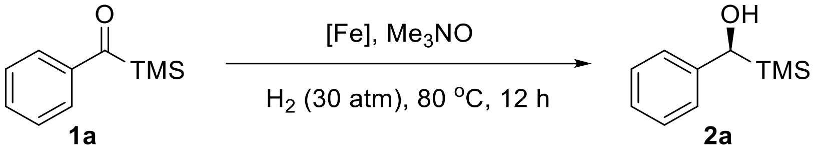

1a 2a

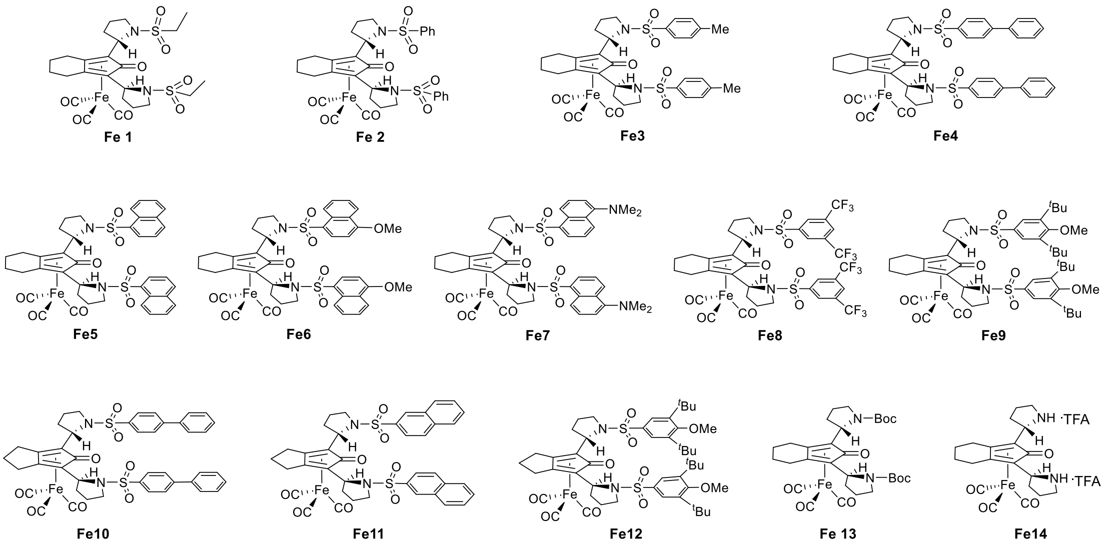

entry	[Fe]	yield (%)[b]	er[c]
1	**Fe1**	88	85:15
2	**Fe2**	87	88:12
3	**Fe3**	89	86:14
4	**Fe4**	87	86:14
5	**Fe5**	64	90:10
6	**Fe6**	87	89:11
7	**Fe7**	88	89.5:10.5
8	**Fe8**	37	87:13
9	**Fe9**	89	90.5:9.5
10	**Fe10**	87	82:18
11	**Fe11**	88	78:22
12	**Fe12**	87	86:14
13	**Fe13**	88	80:20
14	**Fe14**	20	63:37

[a]Reaction conditions: **1a** (0.2 mmol), **[Fe]** (2 mol %), Me_3NO (4 mol %), toluene (0.3 mL), H_2 (30 atm), 80 °C, 12 h. [b]Determined by crude ^1H NMR analysis using CH_2Br_2 as internal standard. [c]Determined by chiral HPLC analysis.

Supplementary Table 2. Screening of Solvents[a]

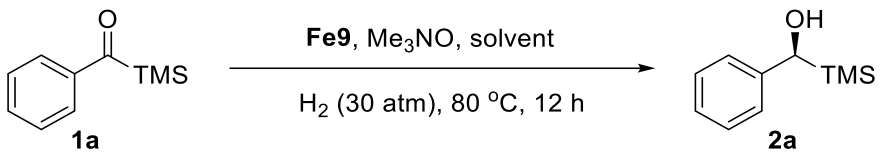

Entry	Solvent	Yield (%)[b]	Er[c]
1	Toluene	89	90.5:9.5
2	^{i}PrOH	87	90.5:9.5
3	THF	88	90.5:9.5
4[d]	^{i}PrOH/H_2O	90	91:9
5[e]	^{i}PrOH/H_2O	89	91:9

[a]Reaction condition: **1a** (0.2 mmol), **Fe9** (2 mol %), Me_3NO (4 mol %), solvent (0.3 mL), H_2 (30 atm), 80 °C, 12 h. [b]Determined by crude ^1H NMR analysis using CH_2Br_2 as internal standard. [c]Determined by chiral HPLC analysis. [d] ^{i}PrOH/H_2O = 4:1. [e]**Fe9** (0.5 mol %).

Supplementary Table 3. Screening of Temperature and Additives[a]

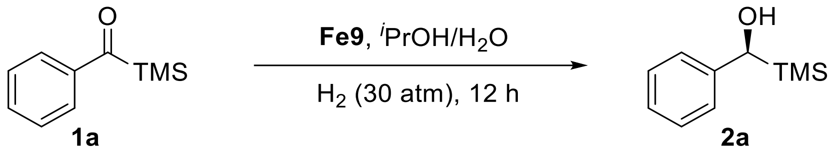

Entry	T (°C)	Additive	Yield (%)[b]	Er[c]
1	80	Me_3NO	90	91:9
2	80	K_2CO_3	86	90.5:9.5
3	80	Na_2CO_3	74	90:10
4	80	^{t}BuOK	trace	--
5	60	Me_3NO	93	93:7
6[d]	60	Me_3NO	72	93:7
7[e]	60	Me_3NO	35	93:7
8[f]	60	Me_3NO	50	92:8
9	40	Me_3NO	16	94.5:5.5

[a]Reaction condition: **1a** (0.2 mmol), **Fe9** (2 mol %), Me_3NO (4 mol %), ^{i}PrOH/H_2O = 4:1 (0.3 mL), H_2 (30 atm), 12 h. [b]Determined by crude ^{1}H NMR analysis using CH_2Br_2 as internal standard. [c]Determined by chiral HPLC analysis. [d]**Fe9** (1 mol %), Me_3NO (2 mol %). [e] ^{i}PrOH (0.3 mL) as solvent. [f]Toluene (0.3 mL) as solvent.

Supplementary Table 4. Optimization of Reaction Concentration and Additives Equivalents[a]

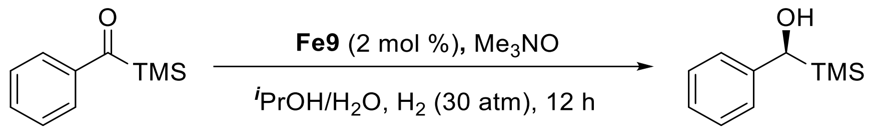

1a **2a**

Entry	Me_3NO (mol %)	Concentration	Yield (%)[b]	Er[c]
1	1	0.6 M	trace	--
2	2	0.6 M	45	93:7
3	4	0.6 M	93	93:7
4	6	0.6 M	90	92.5:7.5
5	10	0.6 M	87	92:8
6	4	0.3 M	95	93:7
7	4	0.1 M	94	92.5:7.5

[a]Reaction condition: **1a** (0.2 mmol), **Fe9** (2 mol %), Me_3NO (1-10 mol %), $^{i}PrOH/H_2O$ = 4:1, H_2 (30 atm), 60 °C. [b]Determined by crude ^1H NMR analysis using CH_2Br_2 as internal standard. [c]Determined by chiral HPLC analysis.

V. Catalytic Asymmetric Hydrogenation of Aromatic Substrates

General Procedure E

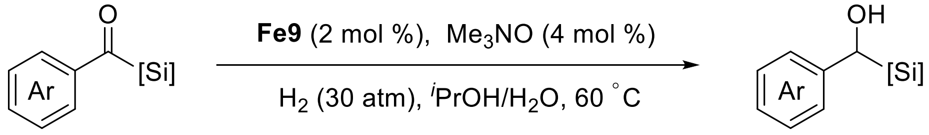

1 **2**

Under nitrogen atmosphere, to a 5-mL glass vial equipped with a magnetic stir bar, was added **Fe9** (9.7 mg, 0.01 mmol, 2 mol %), solvent (^{i}PrOH 1.2 mL and H_2O 0.3 mL), Me_3NO (1.5 mg, 0.02 mmol, 4 mol %) and substrate **1** (0.5 mmol, 1.0 equiv.). The vial was transferred to a 50-mL autoclave and purged with H_2 two times (charge 10 atm H_2 and slowly release the H_2 each time), then the autoclave was charged with H_2 (30 atm). The autoclave was stirred and heated on a stir plate at 60 °C for 12 h. Took out the autoclave, cooled down to ambient temperature, and H_2 was carefully released. After removal of the solvent under reduced pressure, the residue was purified by silica gel column chromatography to afford the desired product **2**.

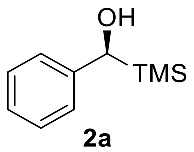

2a

(*S*)-Phenyl(trimethylsilyl)methanol (2a) was prepared as a colorless oil from **1a** (90 mg, 0.5 mmol) according to the General Procedure E (12 h, eluent: *n*-hexane/EtOAc = 20:1 → 15:1, 85.3 mg, 95% yield, 93:7 er). **^{1}H NMR** was consistent to the literature report.[22]

$[\alpha]_D^{25}$: -62.5 (c = 1.0, $CHCl_3$). HPLC analysis of the product: Daicel CHIRALCEL OD-H column; 10% *i*-PrOH in *n*-hexane; 1.0 mL/min; retention times: 5.2 min (major), 7.3 min (minor).

^{1}H NMR (400 MHz, $CDCl_3$) δ 7.35 (dd, J = 8.4, 6.96 Hz, 2H), 7.25 – 7.19 (m, 3H), 4.53 (s, 1H), 2.05 – 1.96 (m, 1H), 0.07 (s, 9H) ppm.

^{13}**C NMR** (100 MHz, $CDCl_3$) δ 144.3, 128.1, 125.8, 125.0, 70.5, -4.1 ppm.

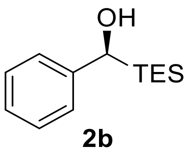

2b

(*S*)-Phenyl(triethylsilyl)methanol (**2b**) was prepared as a colorless oil from **1b** (112 mg, 0.5 mmol) according to the General Procedure E (12 h, eluent: *n*-hexane/EtOAc = 20:1→15:1, 100.8 mg, 90% yield, 92:8 er). ^{1}**H NMR** was consistent to the literature report.[23]

$[\alpha]_D^{25}$: -66.8 (*c* = 1.0, $CHCl_3$). HPLC analysis of the product: Daicel CHIRALCEL OD-H column; 10% *i*-PrOH in *n*-hexane; 1.0 mL/min; retention times: 5.4 min (major), 9.1 min (minor).

^{1}**H NMR** (300 MHz, $CDCl_3$) δ 7.40 – 7.30 (m, 2H), 7.30 – 7.14 (m, 3H), 4.69 (s, 1H), 1.85 – 1.70 (m, 1H), 0.97 (t, *J* = 7.9 Hz, 9H), 0.70 – 0.50 (m, 6H).

^{13}**C NMR** (75 MHz, $CDCl_3$) δ 144.8, 128.2, 125.7, 124.9, 68.8, 7.4, 1.4 ppm.

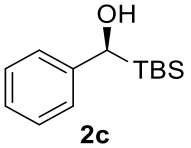

2c

(*S*)-(*tert*-Butyldimethylsilyl)(phenyl)methanol (**2c**) was prepared as a colorless oil from **1c** (112 mg, 0.5 mmol) according to the General Procedure E (12 h, eluent: *n*-hexane/EtOAc = 20:1→15:1, 101 mg, 91% yield, 91:9 er). ^{1}**H NMR** was consistent to the literature report.[15]

$[\alpha]_D^{25}$: -37.4 (*c* = 1.0, $CHCl_3$). HPLC analysis of the product: Daicel CHIRALCEL OD-H column; 10% *i*-PrOH in *n*-hexane; 1.0 mL/min; retention times: 6.4 min (major), 11.9 min (minor).

^{1}**H NMR** (400 MHz, $CDCl_3$) δ 7.40 – 7.32 (m, 2H), 7.29 – 7.19 (m, 3H), 4.70 (s, 1H), 1.97 – 1.80 (m, 1H), 1.04 (d, *J* = 2.9 Hz, 9H), 0.08 (d, *J* = 2.5 Hz, 3H), -0.13 (d, *J* = 2.5 Hz, 3H) ppm.

^{13}C NMR (100 MHz, $CDCl_3$) δ 144.9, 128.2, 125.9, 125.5, 68.9, 27.1, 17.2, -7.1, -9.3 ppm.

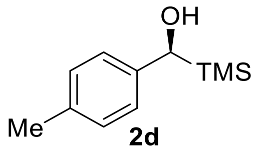

2d

(*S*)-*p*-Tolyl(trimethylsilyl)methanol (**2d**) was prepared as a colorless oil from **1d** (97 mg, 0.5 mmol) according to the General Procedure E (12 h, eluent: *n*-hexane/EtOAc = 20:1→15:1, 90 mg, 92% yield, 95:5 er). **^{1}H NMR** was consistent to the literature report.[22]

$[\alpha]_D^{25}$: -98.6 (c = 1.0, $CHCl_3$). HPLC analysis of the product: Daicel CHIRALCEL OD-H column; 10% *i*-PrOH in *n*-hexane; 1.0 mL/min; retention times: 4.5 min (major), 5.7 min (minor).

^{1}H NMR (300 MHz, $CDCl_3$) δ 7.22 – 7.01 (m, 4H), 4.50 (s, 1H), 2.37 (s, 3H), 1.84 (s, 1H), 0.05 (s, 9H) ppm.

^{13}C NMR (75 MHz, $CDCl_3$) δ 141.2, 135.3, 128.8, 125.0, 70.42, 21.1, -4.1 ppm.

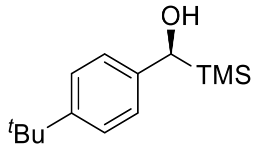

2e

(*S*)-(4-(*tert*-Butyl)phenyl)(trimethylsilyl)methanol (**2e**) was prepared as a colorless oil from **1e** (117 mg, 0.5 mmol) according to the General Procedure E (12 h, eluent: *n*-hexane/EtOAc = 20:1→15:1, 95 mg, 80% yield, 95:5 er). **^{1}H NMR** was consistent to the literature report.[22]

$[\alpha]_D^{25}$: -109.7 (c = 1.0, $CHCl_3$). HPLC analysis of the product: Daicel CHIRALCEL OD-H column; 10% *i*-PrOH in *n*-hexane; 1.0 mL/min; retention times: 4.1 min (major), 4.6 min (minor).

^{1}H NMR (300 MHz, $CDCl_3$) δ 7.41 – 7.32 (m, 2H), 7.20 – 7.10 (m, 2H), 4.52 (s, 1H), 1.74 (s, 1H), 1.35 (s, 9H), 0.06 (s, 9H) ppm.

^{13}C NMR (75 MHz, $CDCl_3$) δ 148.6, 141.2, 125.0, 124.7, 70.4, 34.4, 31.4, -4.0 ppm.

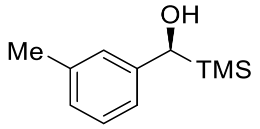

2f

(*S*)-*m*-Tolyl(trimethylsilyl)methanol (2f) was prepared as a colorless oil from **1f** (97 mg, 0.5 mmol) according to the General Procedure E (12 h, eluent: *n*-hexane/EtOAc = 20:1→15:1, 95 mg, 98% yield, 94:6 er). **^{1}H NMR** was consistent to the literature report.[22]

$[\alpha]_D^{25}$: -74.5 (*c* = 1.0, $CHCl_3$). HPLC analysis of the product: Daicel CHIRALCEL OD-H column; 10% *i*-PrOH in *n*-hexane; 1.0 mL/min; retention times: 4.6 min (major), 5.1 min (minor).

^{1}H NMR (300 MHz, $CDCl_3$) δ 7.24 (t, *J* = 7.4 Hz, 1H), 7.13 – 6.98 (m, 3H), 4.50 (s, 1H), 2.39 (s, 3H), 1.96 (d, *J* = 2.8 Hz, 1H), 0.07 (d, *J* = 1.23 Hz, 9H) ppm.

^{13}C NMR (75 MHz, $CDCl_3$) δ 144.3, 137.7, 128.1, 126.6, 125.6, 122.1, 70.5, 21.6, -4.0 ppm.

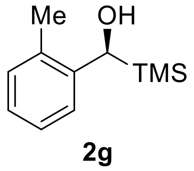

2g

(*S*)-*o*-Tolyl(trimethylsilyl)methanol (2g) was prepared as a colorless oil from **1g** (97 mg, 0.5 mmol) according to the General Procedure E (12 h, eluent: *n*-hexane/EtOAc = 20:1→15:1, 88 mg, 91% yield, 75:25 er). **^{1}H NMR** was consistent to the literature report.[22]

$[\alpha]_D^{25}$: -74.5 (*c* = 1.0, $CHCl_3$). HPLC analysis of the product: Daicel CHIRALPAK AD-H column; 4% *i*-PrOH in *n*-hexane; 1.0 mL/min; retention times: 6.0 min (major), 5.5 min (minor).

^{1}H NMR (300 MHz, $CDCl_3$) δ 7.42 (d, *J* = 7.7 Hz, 1H), 7.26 (td, *J* = 7.9, 6.9, 2.4 Hz, 1H), 7.20 – 7.09 (m, 2H), 4.83 (s, 1H), 2.28 (s, 3H), 1.88 (s, 1H), 0.11 (s, 9H) ppm.

^{13}C NMR (75 MHz, $CDCl_3$) δ 142.7, 133.1, 130.1, 126.1, 125.7, 125.6, 66.0, 19.8, -3.6 ppm.

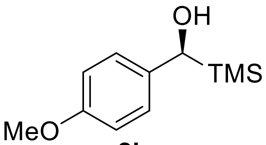

2h

(*S*)-(4-Methoxyphenyl)(trimethylsilyl)methanol (2h) was prepared as a colorless oil from **1h** (104 mg, 0.5 mmol) according to the General Procedure E (12 h, eluent: *n*-hexane/EtOAc = 20:1→15:1, 100 mg, 95% yield, 94:6 er). **^{1}H NMR** was consistent to the literature report.[22]

$[\alpha]_D^{25}$: -84.3 (*c* = 1.0, $CHCl_3$). HPLC analysis of the product: Daicel CHIRALCEL OD-H column; 10% *i*-PrOH in *n*-hexane; 1.0 mL/min; retention times: 6.0 min (major), 6.9 min (minor).

^{1}H NMR (400 MHz, $CDCl_3$) δ 7.14 (d, *J* = 8.8 Hz, 2H), 6.88 (d, *J* = 8.7 Hz, 2H), 4.47 (s, 1H), 3.82 (s, 3H), 1.81 – 1.69 (m, 1H), 0.03 (s, 9H) ppm.

^{13}C NMR (100 MHz, $CDCl_3$) δ 157.8, 136.3, 126.2, 113.6, 70.1, 55.3, -4.1 ppm.

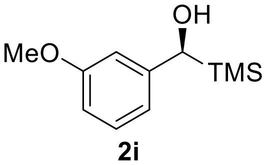

2i

(*S*)-(3-Methoxyphenyl)(trimethylsilyl)methanol (2i) was prepared as a colorless oil from **1i** (104 mg, 0.5 mmol) according to the General Procedure E

(12 h, eluent: *n*-hexane/EtOAc = 20:1→15:1, 97.6 mg, 93% yield, 92:8 er). **^{1}H NMR** was consistent to the literature report.[24]

$[\alpha]_D^{25}$: -64.8 (*c* = 1.0, $CHCl_3$). HPLC analysis of the product: Daicel CHIRALCEL OD-H column; 10% *i*-PrOH in *n*-hexane; 1.0 mL/min; retention times: 7.6 min (major), 15.1 min (minor).

^{1}H NMR (300 MHz, $CDCl_3$) δ 7.28 – 7.13 (m, 1H), 6.84 – 6.69 (m, 3H), 4.51 (s, 1H), 3.81 (s, 3H), 1.92 (s, 1H), 0.05 (s, 9H) ppm.

^{13}C NMR (75 MHz, $CDCl_3$) δ 159.6, 146.2, 129.1, 117.4, 111.2, 110.4, 70.5, 55.1, -4.0 ppm.

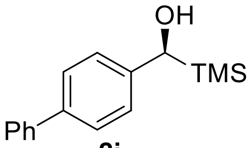

2j

(*S*)-[1,1'-Biphenyl]-4-yl(trimethylsilyl)methanol (2j) was prepared as a colorless oil from **1j** (127 mg, 0.5 mmol) according to the General Procedure E (12 h, eluent: *n*-hexane/EtOAc = 20:1→15:1, 121.5 mg, 95% yield, 93:7 er). **^{1}H NMR** was consistent to the literature report.[22]

$[\alpha]_D^{25}$: -93.2 (*c* = 1.0, $CHCl_3$). HPLC analysis of the product: Daicel CHIRALCEL OD-H column; 10% *i*-PrOH in *n*-hexane; 1.0 mL/min; retention times: 6.9 min (major), 9.1 min (minor).

^{1}H NMR (300 MHz, $CDCl_3$) δ 7.70 – 7.54 (m, 4H), 7.54 – 7.44 (m, 2H), 7.43 – 7.35 (m, 1H), 7.34 – 7.27 (m, 2H), 4.61 (s, 1H), 2.04 (s, 1H), 0.11 (s, 9H) ppm.

^{13}C NMR (75 MHz, $CDCl_3$) δ 143.5, 141.0, 138.6, 128.8, 127.1, 126.9, 126.9, 125.4, 70.4, -4.0 ppm.

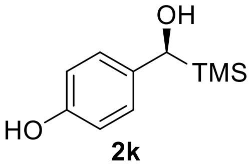

2k

(*S*)-4-(Hydroxy(trimethylsilyl)methyl)phenol (2k) was prepared as a colorless oil from **1k** (97 mg, 0.5 mmol) according to the General Procedure E (12 h, eluent: *n*-hexane/EtOAc = 5:1→2:1, 90.2 mg, 92% yield, 96:4 er).

$[\alpha]_D^{25}$: -70.6 (*c* = 1.0, $CHCl_3$). HPLC analysis of the product: Daicel CHIRALPAK AS-H column; 10% *i*-PrOH in *n*-hexane; 1.0 mL/min; retention times: 10.0 min (major), 8.4 min (minor).

^{1}H NMR (300 MHz, Methanol-d_4) δ 6.95 (d, *J* = 8.4 Hz, 2H), 6.66 (d, *J* = 8.6 Hz, 2H), 4.28 (s, 1H), -0.09 (s, 9H) ppm.

^{13}C NMR (75 MHz, Methanol-d_4) δ 155.0, 135.0, 126.2, 114.4, 69.2, -5.1 ppm.

HR-MS (ESI) *m/z* Calcd. for $C_{10}H_{16}NaO_2Si^+$ $[M+Na]^+$: 219.0812, found: 219.0812

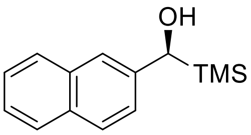

2l

(*S*)-Naphthalen-2-yl(trimethylsilyl)methanol (2l) was prepared as a colorless oil from **1l** (114 mg, 0.5 mmol) according to the General Procedure E (12 h, eluent: *n*-hexane/EtOAc = 20:1→15:1, 105.8 mg, 92% yield, 93:7 er). **^{1}H NMR** was consistent to the literature report.[25]

$[\alpha]_D^{25}$: -80.2 (*c* = 1.0, $CHCl_3$). HPLC analysis of the product: Daicel CHIRALCEL OD-H; 10% *i*-PrOH in *n*-hexane; 1.0 mL/min; retention times: 8.4 min (major), 12.9 min (minor).

^{1}H NMR (400 MHz, $CDCl_3$) δ 7.88 – 7.77 (m, 3H), 7.71 – 7.64 (m, 1H), 7.60 – 7.46 (m, 2H), 7.33 (dd, *J* = 8.5, 1.8 Hz, 1H), 4.73 (s, 1H), 1.82 (s, 1H), 0.08 (s, 9H) ppm.

^{13}C NMR (100 MHz, $CDCl_3$) δ 142.0, 133.5, 132.1, 127.7, 127.6, 127.6, 126.0, 125.1, 124.2, 122.5, 70.8, -4.0 ppm.

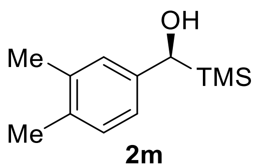

2m

(*S*)-(3,4-Dimethylphenyl)(trimethylsilyl)methanol (2m) was prepared as a colorless oil from **1m** (103 mg, 0.5 mmol) according to the General Procedure E (12 h, eluent: *n*-hexane/EtOAc = 20:1→15:1, 93.6 mg, 90% yield, 94:6 er).

$[\alpha]_D^{25}$: -67.2 (*c* = 1.0, $CHCl_3$). HPLC analysis of the product: Daicel CHIRALCEL OD-H; 10% *i*-PrOH in *n*-hexane; 1.0 mL/min; retention times: 4.8 min (major), 6.9 min (minor).

^{1}H NMR (300 MHz, $CDCl_3$) δ 7.10 (d, *J* = 7.7 Hz, 1H), 7.04 – 6.90 (m, 2H), 4.48 (s, 1H), 2.32 – 2.23 (m, 6H), 1.91 (s, 1H), 0.06 (s, 9H) ppm.

^{13}C NMR (75 MHz, $CDCl_3$) δ 141.7, 136.3, 133.9, 129.4, 126.3, 122.5, 70.4, 19.9, 19.4, -4.0 ppm.

HR-MS (ESI) *m/z* Calcd. for $C_{12}H_{20}NaOSi^+$ $[M+Na]^+$: 231.1176, found: 231.1172.

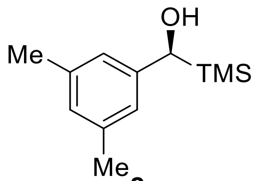

2n

(*S*)-(3,5-Dimethylphenyl)(trimethylsilyl)methanol (2n) was prepared as a colorless oil from **1n** (103 mg, 0.5 mmol) according to the General Procedure E (12 h, eluent: *n*-hexane/EtOAc = 20:1→15:1, 100.5 mg, 97% yield, 95:5 er).

$[\alpha]_D^{25}$: -74.2 (*c* = 1.0, $CHCl_3$). HPLC analysis of the product: Daicel CHIRALCEL OD-H; 5% *i*-PrOH in *n*-hexane; 1.0 mL/min; retention times: 4.4 min (major), 4.9 min (minor).

^{1}H NMR (400 MHz, $CDCl_3$) δ 6.96 – 6.47 (m, 3H), 4.47 (s, 1H), 2.35 (s, 6H), 0.07 (s, 9H) ppm.

^{13}C NMR (100 MHz, $CDCl_3$) δ 144.3, 137.6, 127.5, 122.8, 70.5, 21.4, -4.0 ppm.

HR-MS (ESI) *m/z* Calcd. for $C_{12}H_{20}NaOSi^+$ $[M+Na]^+$: 231.1176, found: 231.1174.

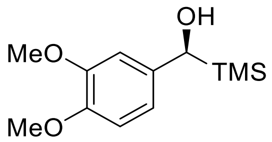

2o

(*S*)-(3,4-Dimethoxyphenyl)(trimethylsilyl)methanol (2o) was prepared as a colorless oil from **1o** (119 mg, 0.5 mmol) according to the General Procedure E (12 h, eluent: *n*-hexane/EtOAc = 10:1→5:1, 115 mg, 96% yield, 91:9 er).

$[\alpha]_D^{25}$: -68.5 (c = 1.0, $CHCl_3$). HPLC analysis of the product: Daicel CHIRALPAK AS-H; 10% *i*-PrOH in *n*-hexane; 1.0 mL/min; retention times: 6.1 min (major), 5.7 min (minor).

^{1}H NMR (300 MHz, $CDCl_3$) δ 6.92 – 6.60 (m, 3H), 4.46 (d, J = 2.3 Hz, 1H), 3.95 – 3.78 (m, 6H), 1.84 (s, 1H), 0.02 (s, 9H) ppm.

^{13}C NMR (75 MHz, $CDCl_3$) δ 148.8, 147.1, 136.9, 116.9, 110.9, 108.5, 70.3, 55.9, 55.8, -4.0 ppm.

HR-MS (ESI) *m/z* Calcd. for $C_{12}H_{20}NaO_3Si^+$ $[M+Na]^+$: 263.1074, found: 231. 263.1071.

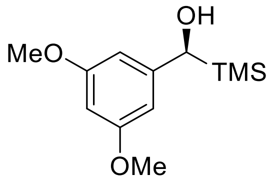

2p

(*S*)-(3,5-Dimethoxyphenyl)(trimethylsilyl)methanol (2p) was prepared as a colorless oil from **1p** (119 mg, 0.5 mmol) according to the General Procedure E (12 h, eluent: *n*-hexane/EtOAc = 10:1→5:1, 110.3 mg, 92% yield, 92:8 er). **^{1}H NMR** was consistent to the literature report.[22]

$[\alpha]_D^{25}$: -57.9 (c = 1.0, $CHCl_3$). HPLC analysis of the product: Daicel CHIRALCEL OD-H; 10% *i*-PrOH in *n*-hexane; 1.0 mL/min; retention times: 4.9 min (major), 7.4 min (minor).

^{1}H NMR (300 MHz, $CDCl_3$) δ 6.37 (d, *J* = 2.4 Hz, 2H), 6.30 (t, *J* = 2.4 Hz, 1H), 4.48 (s , 1H), 3.79 (s, 6H), 1.84 (s, 1H), 0.05 (s, 9H) ppm.

^{13}C NMR (75 MHz, $CDCl_3$) δ 160.6, 147.1, 102.8, 97.8, 70.7, 55.5, -4.0 ppm.

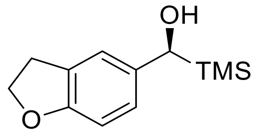

2q

(*S*)-(2,3-Dihydrobenzofuran-5-yl)(trimethylsilyl)methanol (2q) was prepared as a colorless oil from **1q** (110 mg, 0.5 mmol) according to the General Procedure E (12 h, eluent: *n*-hexane/EtOAc = 10:1→5:1, 107.6 mg, 97% yield, 94:6 er).

$[\alpha]_D^{25}$: -74.04 (*c* = 1.0, $CHCl_3$). HPLC analysis of the product: Daicel CHIRALCEL OD-H; 10% *i*-PrOH in *n*-hexane; 1.0 mL/min; retention times: 8.4 min (major), 11.4 min (minor).

^{1}H NMR (300 MHz, $CDCl_3$) δ 7.15 – 7.02 (m, 1H), 7.00 – 6.90 (m, 1H), 6.74 (d, *J* = 8.2 Hz, 1H), 4.57 (t, *J* = 8.8 Hz, 2H), 4.44 (s, 1H), 3.21 (t, *J* = 8.6 Hz, 2H), 1.71 (s, 1H), 0.03 (s, 9H) ppm.

^{13}C NMR (75 MHz, $CDCl_3$) δ 158.4, 136.3, 126.9, 124.8, 121.8, 108.8, 71.2, 70.4, 29.8, -4.0 ppm.

HR-MS (ESI) *m/z* Calcd. for $C_{12}H_{16}NaO_2Si^+$ $[M+Na]^+$: 243.0812, found: 243.0814.

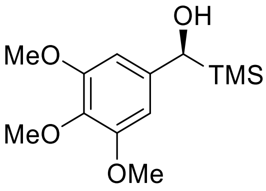

2r

(*S*)-(3,4,5-Trimethoxyphenyl)(trimethylsilyl)methanol (2r) was prepared as a colorless oil from **1r** (134 mg, 0.5 mmol) according to the General Procedure E

(12 h, eluent: *n*-hexane/EtOAc = 10:1→5:1, 121.5 mg, 90% yield, 90:10 er). **^{1}H NMR** was consistent to the literature report.[22]

$[\alpha]_D^{25}$: -37.0 (*c* = 1.0, $CHCl_3$). HPLC analysis of the product: Daicel CHIRALCEL OD-H; 10% *i*-PrOH in *n*-hexane; 1.0 mL/min; retention times: 12.0 min (major), 17.4 min (minor).

^{1}H NMR (300 MHz, $CDCl_3$) δ 6.44 – 6.30 (m, 2H), 4.42 (s, 1H), 3.82 (s, 6H), 3.81 (s, 3H), 2.03 (s, 1H), 0.02 (s, 9H) ppm.

^{13}C NMR (75 MHz, $CDCl_3$) δ 152.9, 140.2, 135.6, 101.7, 70.6, 60.9, 56.0, -4.0 ppm.

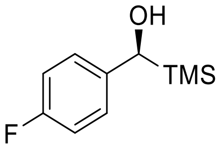

2s

(*S*)-(4-Fluorophenyl)(trimethylsilyl)methanol (2s) was prepared as a colorless oil from **1s** (98 mg, 0.5 mmol) according to the General Procedure E (12 h, eluent: *n*-hexane/EtOAc = 20:1→15:1, 95 mg, 95% yield, 90:10 er). **^{1}H NMR** was consistent to the literature report.[22]

$[\alpha]_D^{25}$: -59.3 (*c* = 1.0, $CHCl_3$). HPLC analysis of the product: Daicel CHIRALCEL OD-H; 10% *i*-PrOH in *n*-hexane; 1.0 mL/min; retention times: 4.2 min (major), 4.8 min (minor).

^{1}H NMR (300 MHz, $CDCl_3$) δ 7.20 – 7.09 (m, 2H), 7.01 (t, *J* = 8.8 Hz, 2H), 4.49 (s, 1H), 1.99 (s, 1H), 0.02 (s, 9H) ppm.

^{13}C NMR (75 MHz, $CDCl_3$) δ 161.2 (d, $^1J_{C\text{-}F}$ = 243.3 Hz), 139.9 (d, $^4J_{C\text{-}F}$ = 3.0 Hz), 126.3 (d, $^3J_{C\text{-}F}$ = 7.8 Hz), 114.9 (d, $^2J_{C\text{-}F}$ = 21.2 Hz), 70.0, -4.21 ppm.

^{19}F NMR {^{1}H} (282 MHz, $CDCl_3$) δ -117.6 (s) ppm.

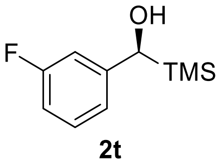

2t

(S)-(3-Fluorophenyl)(trimethylsilyl)methanol (2t) was prepared as a colorless oil from **1t** (98 mg, 0.5 mmol) according to the General Procedure E (12 h, eluent: *n*-hexane/EtOAc = 20:1→15:1, 81.2 mg, 82% yield, 93:7 er).

$[\alpha]_D^{25}$: -47.9 (*c* = 1.0, $CHCl_3$). HPLC analysis of the product: Daicel CHIRALCEL OD-H; 10% *i*-PrOH in *n*-hexane; 1.0 mL/min; retention times: 4.3 min (major), 5.7 min (minor).

^{1}H NMR (300 MHz, $CDCl_3$) δ 7.33 – 7.20 (m, 1H), 6.99 – 6.81 (m, 3H), 4.53 (s, 1H), 1.94 (s, 1H), 0.04 (s, 9H) ppm.

^{13}C NMR (75 MHz, $CDCl_3$) δ 163.0 (d, $^1J_{C\text{-}F}$ = 245.2 Hz), 147.2 (d, $^3J_{C\text{-}F}$ = 6.8 Hz), 129.5 (d, $^3J_{C\text{-}F}$ = 8.4 Hz), 120.4 (d, $^4J_{C\text{-}F}$ = 2.76 Hz), 112.5 (d, $^2J_{C\text{-}F}$ = 21.4 Hz), 111.7 (d, $^2J_{C\text{-}F}$ = 22.0 Hz), 70.13 (d, *J* = 1.7 Hz), -4.2 ppm.

^{19}F NMR {^{1}H} (282 MHz, $CDCl_3$) δ -113.39 (s) ppm.

HR-MS (ESI) *m/z* Calcd. for $C_{10}H_{15}FNaOSi^+$ $[M+Na]^+$: 221.0768, found: 221.0769.

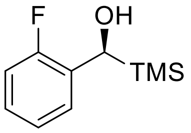

2u

(S)-(2-Fluorophenyl)(trimethylsilyl)methanol (2u) was prepared as a colorless oil from **1u** (98 mg, 0.5 mmol) according to the General Procedure E (12 h, eluent: *n*-hexane/EtOAc = 20:1→15:1, 94 mg, 95% yield, 88:12 er).

$[\alpha]_D^{25}$: -47.9 (*c* = 1.0, $CHCl_3$). HPLC analysis of the product: Daicel CHIRALPAK AS-H; 5% *i*-PrOH in *n*-hexane; 1.0 mL/min; retention times: 4.9 min (major), 4.3 min (minor).

^{1}H NMR (300 MHz, $CDCl_3$) δ 7.51 – 7.33 (m, 1H), 7.21 – 7.09 (m, 2H), 7.09 – 6.89 (m, 1H), 4.88 (d, *J* = 3.4 Hz, 1H), 1.99 (s, 1H), 0.06 (s, 9H) ppm.

^{13}C NMR (75 MHz, $CDCl_3$) δ 158.4 (d, $^1J_{C\text{-}F}$ = 243.3 Hz), 131.6 (d, *J* = 14.1 Hz), 127.0 (d, $^4J_{C\text{-}F}$ = 3.1 Hz), 126.9 (d, $^3J_{C\text{-}F}$ = 6.7 Hz), 124.1 (d, $^4J_{C\text{-}F}$ = 3.3 Hz), 114.8 (d, $^2J_{C\text{-}F}$ = 22.0 Hz), 63.2 (d, *J* = 1.6 Hz), -4.2 (d, *J* = 1.5 Hz) ppm.

^{19}**F NMR** {^{1}H} (282 MHz, $CDCl_3$) δ -117.6 (s) ppm.

HR-MS (ESI) *m/z* Calcd. for $C_{10}H_{15}FNaOSi^+$ [M+Na]$^+$: 221.0768, found: 221.0765.

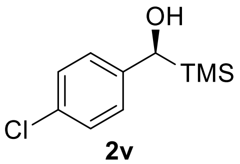

2v

(*S*)-(4-Chlorophenyl)(trimethylsilyl)methanol (2v) was prepared as a colorless oil from **1v** (106 mg, 0.5 mmol) according to the General Procedure E (12 h, eluent: *n*-hexane/EtOAc = 20:1→15:1, 100.5 mg, 94% yield, 92:8 er). ^{1}**H NMR** was consistent to the literature report.[22]

$[\alpha]_D^{25}$: -78.9 (*c* = 1.0, $CHCl_3$). HPLC analysis of the product: Daicel CHIRALCEL OD-H; 10% *i*-PrOH in *n*-hexane; 1.0 mL/min; retention times: 4.5 min (major), 5.4 min (minor).

^{1}**H NMR** (300 MHz, $CDCl_3$) δ 7.28 (d, *J* = 8.5 Hz, 2H), 7.12 (d, *J* = 8.6 Hz, 2H), 4.49 (s, 1H), 2.00 (s, 1H), 0.02 (s, 9H) ppm.

^{13}**C NMR** (75 MHz, $CDCl_3$) δ 142.8, 131.2, 128.2, 126.2, 69.9, -4.2 ppm.

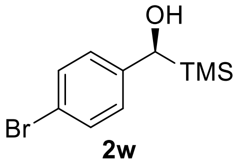

2w

(*S*)-(4-Bromophenyl)(trimethylsilyl)methanol (2w) was prepared as a colorless oil from **1w** (128 mg, 0.5 mmol) according to the General Procedure E (12 h, eluent: *n*-hexane/EtOAc = 20:1→15:1, 116 mg, 90% yield, 93:7 er). ^{1}**H NMR** was consistent to the literature report.[22]

$[\alpha]_D^{25}$: -77.5 (*c* = 1.0, $CHCl_3$). HPLC analysis of the product: Daicel CHIRALCEL OD-H; 10% *i*-PrOH in *n*-hexane; 1.0 mL/min; retention times: 5.0 min (major), 6.1 min (minor).

¹H NMR (300 MHz, $CDCl_3$) δ 7.43 (d, *J* = 8.4 Hz, 2H), 7.17 – 7.00 (m, 2H), 4.48 (s, 1H), 1.93 (s, 1H), 0.02 (s, 9H) ppm.

¹³C NMR (75 MHz, $CDCl_3$) δ 143.3, 131.2, 126.6, 119.2, 70.0, -4.2 ppm.

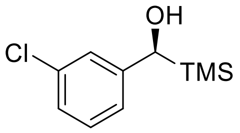

2x

(*S*)-(3-Chlorophenyl)(trimethylsilyl)methanol (2x) was prepared as a colorless oil from **1x** (106 mg, 0.5 mmol) according to the General Procedure E (12 h, eluent: *n*-hexane/EtOAc = 20:1→15:1, 100.5 mg, 94% yield, 92:8 er). **¹H NMR** was consistent to the literature report.[22]

$[\alpha]_D^{25}$: -70.1 (*c* = 1.0, $CHCl_3$). HPLC analysis of the product: Daicel CHIRALCEL OD-H; 10% *i*-PrOH in *n*-hexane; 1.0 mL/min; retention times: 4.9 min (major), 7.4 min (minor).

¹H NMR (300 MHz, $CDCl_3$) δ 7.32 – 7.11 (m, 3H), 7.05 (d, *J* = 7.7 Hz, 1H), 4.50 (s, 1H), 1.99 (s, 1H), 0.04 (s, 9H) ppm.

¹³C NMR (75 MHz, CDCl3) δ 146.6, 134.2, 129.4, 125.8, 124.8, 123.0, 70.0, -4.2 ppm.

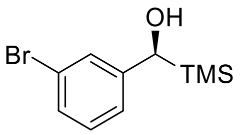

2y

(*S*)-(3-Bromophenyl)(trimethylsilyl)methanol (2y) was prepared as a colorless oil from **1y** (128 mg, 0.5 mmol) according to the General Procedure E (12 h, eluent: *n*-hexane/EtOAc = 20:1→15:1, 120 mg, 93% yield, 92:8 er).

$[\alpha]_D^{25}$: -70.1 (*c* = 1.0, $CHCl_3$). HPLC analysis of the product: Daicel CHIRALCEL OD-H; 10% *i*-PrOH in *n*-hexane; 1.0 mL/min; retention times: 4.7 min (major), 6.8 min (minor).

^{1}H NMR (300 MHz, $CDCl_3$) δ 7.41 – 7.28 (m, 2H), 7.22 – 7.13 (m, 1H), 7.10 (dd, *J* = 7.8, 1.7 Hz, 1H), 4.49 (s, 1H), 1.99 (s, 1H), 0.04 (s, 9H) ppm.

^{13}C NMR (75 MHz, $CDCl_3$) δ 146.8, 129.7, 128.8, 127.7, 123.4, 122.5, 69.9, -4.1 ppm.

HR-MS (ESI) *m/z* Calcd. for $C_{10}H_{15}BrNaOSi^+$ $[M+Na]^+$: 280.9968, found: 280.9969.

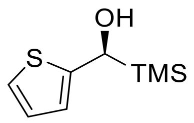

2z

(*S*)-Thiophen-2-yl(trimethylsilyl)methanol (2z) was prepared as a colorless oil from **1z** (92 mg, 0.5 mmol) according to the General Procedure E (12 h, eluent: *n*-hexane/EtOAc = 20:1→15:1, 88 mg, 95% yield, 94:6 er). **^{1}H NMR** was consistent to the literature report.[26]

$[\alpha]_D^{25}$: -72.6 (*c* = 1.0, $CHCl_3$). HPLC analysis of the product: Daicel CHIRALCEL OD-H; 10% *i*-PrOH in *n*-hexane; 1.0 mL/min; retention times: 4.6 min (major), 5.2 min (minor).

^{1}H NMR (400 MHz, $CDCl_3$) δ 7.18 (dd, *J* = 5.1, 1.2 Hz, 1H), 6.99 (dd, *J* = 5.1, 3.5 Hz, 1H), 6.83 (dd, *J* = 3.5, 1.2 Hz, 1H), 4.76 (s, 1H), 1.96 (s, 1H), 0.11 (s, 9H) ppm.

^{13}C NMR (100 MHz, $CDCl_3$) δ 148.2, 126.8, 123.1, 121.9, 66.6, -4.0 ppm.

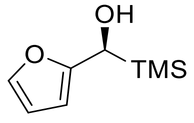

2aa

(*S*)-Furan-2-yl(trimethylsilyl)methanol (2aa) was prepared as a colorless oil from **1aa** (84 mg, 0.5 mmol) according to the General Procedure E (12 h, eluent: *n*-hexane/EtOAc = 20:1→15:1, 77 mg, 91% yield, 92:8 er).

$[\alpha]_D^{25}$: -12.3 (*c* = 1.0, $CHCl_3$). HPLC analysis of the product: Daicel CHIRALCEL OJ-H; 2% *i*-PrOH in *n*-hexane; 1.0 mL/min; retention times: 6.8 min (major), 7.5 min (minor).

^{1}H NMR (300 MHz, $CDCl_3$) δ 7.37 (d, *J* = 1.9 Hz, 1H), 6.34 (dd, *J* = 3.2, 1.8 Hz, 1H), 6.15 (d, *J* = 3.2 Hz, 1H), 4.45 (s, 1H), 1.77 (s, 1H), 0.11 (s, 9H) ppm.

^{13}C NMR (75 MHz, $CDCl_3$) δ 157.1, 141.7, 110.3, 105.6, 63.3, -3.7 ppm.

HR-MS (ESI) *m*/*z* Calcd. for $C_8H_{14}NaO_2Si^+$ $[M+Na]^+$: 193.0655, found: 193.0662.

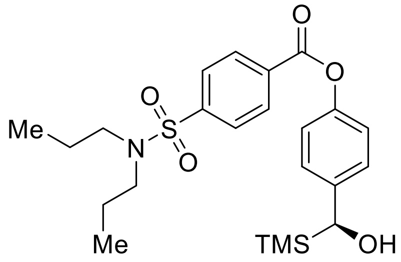

2ab

(*S*)-4-(Hydroxy(trimethylsilyl)methyl)phenyl 4-(*N*,*N* dipropylsulfamoyl) benzoate (2ab) was prepared as a colorless oil from **1ab** (230.5 mg, 0.5 mmol) according to the General Procedure E (12 h, eluent: *n*-hexane/EtOAc = 5:1→2:1, 210.6 mg, 91% yield, 94:6 er).

$[\alpha]_D^{25}$: -86.3 (*c* = 1.0, $CHCl_3$). HPLC analysis of the product: Daicel CHIRALCEL OD-H; 30% *i*-PrOH in *n*-hexane; 1.0 mL/min; retention times: 9.6 min (major), 13.0 min (minor).

^{1}H NMR (300 MHz, $CDCl_3$) δ 8.44 – 8.07 (m, 2H), 8.05 – 7.82 (m, 2H), 7.34 – 7.25 (m, 2H), 7.18 (d, *J* = 8.6 Hz, 2H), 4.58 (s, 1H), 3.24 – 3.01 (m, 4H), 1.93 (s, 1H), 1.68 – 1.49 (m, 4H), 0.90 (t, *J* = 7.4Hz, 6H), 0.05 (s, 9H) ppm.

^{13}C NMR (75 MHz, $CDCl_3$) δ 164.0, 148.6, 144.8, 142.5, 132.9, 130.8, 127.2, 125.9, 121.1, 70.0, 49.9, 21.9, 11.2, -4.1 ppm.

HR-MS (ESI) *m*/*z* Calcd. for $C_{23}H_{33}NNaO_5SSi^+$ $[M+Na]^+$: 486.1741, found: 486.1739.

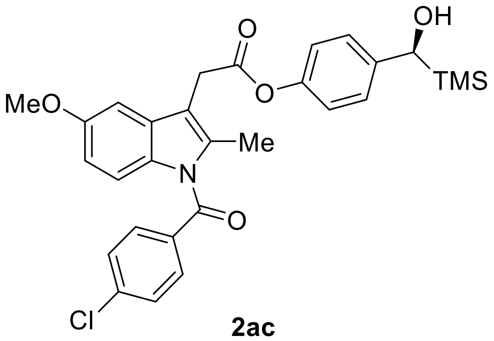

2ac

(*S*)-4-(Hydroxy(trimethylsilyl)methyl)phenyl 2-(1-(4-chlorobenzoyl)-5-methoxy-2-methyl-1*H*-indol-3-yl)acetate (2ac) was prepared as a colorless oil from **1ac** (266.5 mg, 0.5 mmol) according to the General Procedure D (12 h, eluent: *n*-hexane/EtOAc = 5:1→2:1, 246 mg, 92% yield, 96:4 er).

$[\alpha]_D^{25}$: -31.8 (*c* = 1.0, $CHCl_3$). HPLC analysis of the product: Daicel CHIRALCEL OD-H; 30% *i*-PrOH in *n*-hexane; 1.0 mL/min; retention times: 13.5 min (major), 22.8 min (minor).

^{1}H NMR (300 MHz, $CDCl_3$) δ 7.70 (d, *J* = 8.5 Hz, 2H), 7.49 (d, *J* = 8.5 Hz, 2H), 7.19 (d, *J* = 8.6 Hz, 2H), 7.08 (d, *J* = 2.5 Hz, 1H), 7.02 (d, *J* = 8.6 Hz, 2H), 6.91 (d, *J* = 9.0 Hz, 1H), 6.72 (dd, *J* = 9.0, 2.5 Hz, 1H), 4.52 (s, 1H), 3.92 (s, 2H), 3.86 (s, 3H), 2.48 (s, 3H), 1.87 (s, 1H), 0.02 (s, 9H) ppm.

^{13}C NMR (75 MHz, $CDCl_3$) δ 169.6, 168.4, 156.1, 148.7, 142.1, 139.4, 136.2, 133.8, 131.2, 130.8, 130.6, 129.2, 125.8, 121.1, 115.1, 112.1, 111.8, 101.2, 70.0, 55.8, 30.6, 13.5, -4.2 ppm.

HR-MS (ESI) *m/z* Calcd. for $C_{29}H_{31}ClNO_5Si^+$ $[M+H]^+$: 536.1655, found: 536.1651.

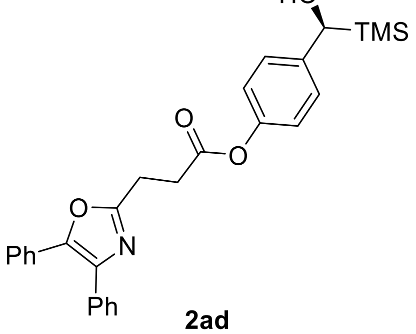

2ad

(*S*)-4-(Hydroxy(trimethylsilyl)methyl)phenyl 3-(4,5-diphenyloxazol-2-yl) propanoate (2ad) was prepared as a colorless oil from **1ad** (234.5 mg, 0.5 mmol) according to the General Procedure E (12 h, eluent: *n*-hexane/EtOAc = 5:1→2:1, 183.7 mg, 78% yield, 95:5 er).

$[\alpha]_D^{25}$: -31.1 (c = 1.0, $CHCl_3$). HPLC analysis of the product: Daicel CHIRALPAK As-H; 10% *i*-PrOH in *n*-hexane; 1.0 mL/min; retention times: 10.5 min (major), 8.7 min (minor).

^{1}H NMR (400 MHz, $CDCl_3$) δ 7.68 (d, J = 6.5 Hz, 2H), 7.61 (d, J = 6.2 Hz, 2H), 7.43 – 7.34 (m, 6H), 7.21 (d, J = 8.6 Hz, 2H), 7.11 – 7.02 (m, 2H), 4.53 (s, 1H), 3.32 (t, J = 7.2 Hz, 2H), 3.18 (t, J = 7.3 Hz, 2H), 2.05 (s, 1H), 0.04 (s, 9H) ppm.

^{13}C NMR (75 MHz, $CDCl_3$) δ 170.9, 161.6, 148.6, 145.6, 142.1, 135.1, 132.3, 128.9, 128.7, 128.6, 128.6, 128.2, 127.9, 126.5, 125.8, 121.2, 70.0, 31.3, 23.6, -4.1 ppm.

HR-MS (ESI) *m/z* Calcd. for $C_{28}H_{30}NO_4Si^+$ $[M+H]^+$: 472.1939, found: 472.1929.

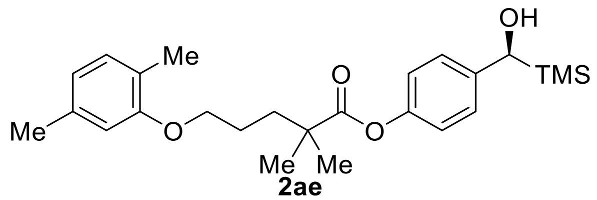

2ae

(*S*)-4-(Hydroxy(trimethylsilyl)methyl)phenyl 5-(2,5-dimethylphenoxy)-2,2-dimethylpentanoate (2ae) was prepared as a colorless oil from **1ae** (214.1 mg, 0.5 mmol) according to the General Procedure E (12 h, eluent: *n*-hexane/EtOAc = 20:1→15:1, 196.8 mg, 92% yield, 95:5 er).

$[\alpha]_D^{25}$: -41.4 (c = 1.0, $CHCl_3$). HPLC analysis of the product: Daicel CHIRALCEL OD-H; 10% *i*-PrOH in *n*-hexane; 1.0 mL/min; retention times: 6.3 min (major), 7.4 min (minor).

^{1}H NMR (300 MHz, $CDCl_3$) δ 7.22 (d, J = 8.4 Hz, 2H), 7.03 (t, J = 8.9 Hz, 3H), 6.74 – 6.63 (m, 2H), 4.55 (s, 1H), 4.02 (s, 2H), 2.35 (s, 3H), 2.22 (s, 3H), 1.92 (d, J = 2.6 Hz, 4H), 1.41 (s, 6H), 0.05 (s, 9H) ppm.

^{13}C NMR (75 MHz, $CDCl_3$) δ 176.6, 156.9, 149.0, 141.7, 136.5, 130.4, 125.8, 123.6, 121.2, 120.8, 111.9, 70.1, 67.8, 42.4, 37.2, 25.3, 25.2, 21.5, 15.9, -4.1 ppm.

HR-MS (ESI) *m/z* Calcd. for $C_{25}H_{36}NaO_4Si^+$ $[M+Na]^+$: 451.2275, found: 451.2270.

VI. Catalytic Asymmetric Hydrogenation of Alkyl and Alkenyl Substrates

General Procedure F.

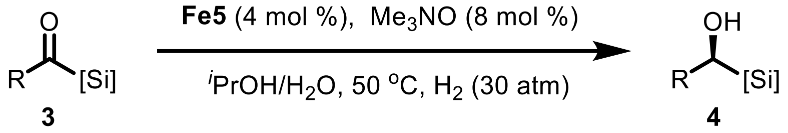

Under nitrogen atmosphere, to a 5-mL glass vial equipped a magnetic stir bar, was added **Fe5** (15.8 mg, 0.02 mmol, 4 mol %), solvent (^{i}PrOH 1.2 mL and H_2O 0.3 mL), Me_3NO (3.0 mg, 0.02 mmol, 8 mol %) and substrate **1** (0.5 mmol, 1.0 equiv.). The via was transferred to a 50-mL autoclave and purged with H_2 two times (charge 10 atm H_2 and slowly release the H_2 each time), then the autoclave was charged with H_2 (30 atm). The autoclave was stirred and heated on a stir plate at 50 °C for 12 h. After cool down to ambient temperature, H_2 was carefully released. After removal of the solvent under reduced pressure, the residue was purified by silica gel column chromatography to afford the desired product **4**.

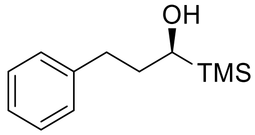

4a

(*S*)-3-Phenyl-1-(trimethylsilyl)propan-1-ol (4a) was prepared as a colorless oil from **3a** (103 mg, 0.5 mmol) according to the General Procedure F (12 h, eluent: *n*-hexane/EtOAc = 15:1→10:1, 96 mg, 94% yield, 96:4 er). ^{1}**H NMR** was consistent to the literature report.[27]

$[\alpha]_D^{25}$: 9.8 (c = 1.0, $CHCl_3$). HPLC analysis of the product: Daicel CHIRALCEL OD-H; 10% *i*-PrOH in *n*-hexane; 1.0 mL/min; retention times: 7.5 min (major), 4.8 min (minor).

^{1}H NMR (300 MHz, $CDCl_3$) δ 7.40 – 7.31 (m, 2H), 7.30 – 7.20 (m, 3H), 3.45 – 3.31 (m, 1H), 3.10 – 2.85 (m, 1H), 2.79 – 2.60 (m, 1H), 1.95 – 1.80 (m, 2H), 1.31 (s, 1H), 0.10 (s, 9H) ppm.

^{13}C NMR (75 MHz, $CDCl_3$) δ 142.3, 128.6, 128.5, 125.9, 65.5, 35.4, 33.4, -3.9 ppm.

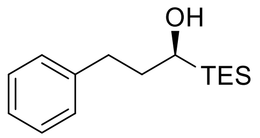

4b

(*S*)-3-Phenyl-1-(triethylsilyl)propan-1-ol (4b) was prepared as a colorless oil from **3b** (124 mg, 0.5 mmol) according to the General Procedure F (12 h, eluent: *n*-hexane/EtOAc = 15:1→10:1, 117.3 mg, 94% yield, 95:5 er). **^{1}H NMR** was consistent to the literature report.[27]

$[\alpha]_D^{25}$: 15.1 (*c* = 1.0, $CHCl_3$). HPLC analysis of the product: Daicel CHIRALCEL OD-H; 10% *i*-PrOH in *n*-hexane; 1.0 mL/min; retention times: 5.8 min (major), 4.4 min (minor).

^{1}H NMR (300 MHz, $CDCl_3$) δ 7.34 (dd, *J* = 8.3, 6.9 Hz, 2H), 7.30 – 7.18 (m, 3H), 3.56 (dd, *J* = 11.1, 3.0 Hz, 1H), 3.08 – 2.92 (m, 1H), 2.79 – 2.59 (m, 1H), 2.08 – 1.62 (m, 3H), 1.08 – 0.95 (m, 9H), 0.72 – 0.55 (m, 6H) ppm.

^{13}C NMR (75 MHz, $CDCl_3$) δ 142.3, 128.5, 128.5, 125.8, 64.1, 35.9, 33.6, 7.6, 1.7 ppm.

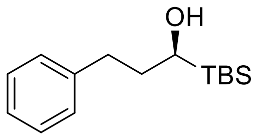

4c

(*S*)-1-(*tert*-Butyldimethylsilyl)-3-phenylpropan-1-ol (4c) was prepared as a colorless oil from **3c** (124 mg, 0.5 mmol) according to the General Procedure F (12 h, eluent: *n*-hexane/EtOAc = 15:1→10:1, 100 mg, 80% yield, 92:8 er). **^{1}H NMR** was consistent to the literature report.[15]

$[\alpha]_D^{25}$: 6.6 (c = 1.0, $CHCl_3$). HPLC analysis of the product: Daicel CHIRALCEL OD-H; 10% *i*-PrOH in *n*-hexane; 1.0 mL/min; retention times: 5.7 min (major), 4.3 min (minor).

^{1}H NMR (300 MHz, $CDCl_3$) δ 7.39 – 7.31 (m, 2H), 7.30 – 7.20 (m, 3H), 3.67 – 3.49 (m, 1H), 3.12 – 2.92 (m, 1H), 2.77 – 2.50 (m, 1H), 2.00 – 1.79 (m, 2H), 1.39 (s, 1H), 1.00 (s, 9H), 0.08 (s, 3H), 0.02 (s, 3H) ppm.

^{13}C NMR (75 MHz, $CDCl_3$) δ 142.3, 128.6, 128.5, 125.9, 64.0, 36.5, 33.5, 27.1, 16.8, -7.5, -8.5 ppm.

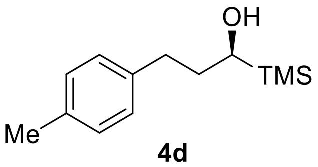

4d

(*S*)-3-(*p*-Tolyl)-1-(trimethylsilyl)propan-1-ol (4d) was prepared as a colorless oil from **3d** (110 mg, 0.5 mmol) according to the General Procedure F (12 h, eluent: *n*-hexane/EtOAc = 15:1→10:1, 102 mg, 92% yield, 95:5 er).

$[\alpha]_D^{25}$: 16.3 (c = 1.0, $CHCl_3$). HPLC analysis of the product: Daicel CHIRALCEL OD-H; 10% *i*-PrOH in *n*-hexane; 1.0 mL/min; retention times: 5.0 min (major), 4.2 min (minor).

^{1}H NMR (300 MHz, $CDCl_3$) δ 7.15 (s, 4H), 3.47 – 3.30 (m, 1H), 3.03 – 2.84 (m, 1H), 2.73 – 2.59 (m, 1H), 2.37 (s, 3H), 1.92 – 1.75 (m, 2H), 1.31 (s, 1H), 0.08 (s, 9H) ppm.

^{13}C NMR (75 MHz, $CDCl_3$) δ 139.2, 135.3, 129.2, 128.4, 65.6, 35.5, 32.9, 21.0, -3.9 ppm.

HR-MS (ESI) *m/z* Calcd. for $C_{13}H_{22}NaOSi^+$ $[M+Na]^+$: 245.1332, found: 245.1333.

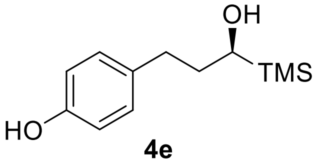

4e

(*S*)-4-(3-Hydroxy-3-(trimethylsilyl)propyl)phenol (4e) was prepared as a colorless oil from **3e** (111 mg, 0.5 mmol) according to the General Procedure F (12 h, eluent: *n*-hexane/EtOAc = 15:1→10:1, 102 mg, 92% yield, 96:4 er).

$[\alpha]_D^{25}$: 12.7 (c = 1.0, $CHCl_3$). HPLC analysis of the product: Daicel CHIRALCEL OD-H; 10% *i*-PrOH in *n*-hexane; 1.0 mL/min; retention times: 14.3 min (major), 9.6 min (minor).

^{1}H NMR (300 MHz, $CDCl_3$) δ 7.08 (d, J = 8.5 Hz, 2H), 6.77 (d, J = 8.5 Hz, 2H), 6.24 (s, 1H), 3.55 – 3.28 (m, 1H), 2.92 – 2.77 (m , 1H), 2.70 – 2.55 (m, 1H), 1.90 – 1.72 (m, 2H), 1.52 (s, 1H), 0.07 (s, 9H) ppm.

^{13}C NMR (75 MHz, $CDCl_3$) δ 154.0, 133.8, 129.5, 115.4, 66.0, 35.3, 32.4, -3.9 ppm.

HR-MS (ESI) *m/z* Calcd. for $C_{12}H_{20}NaO_2Si^+$ [M+Na]$^+$: 247.1125, found: 247.1123.

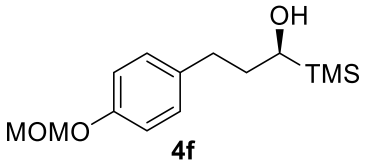

4f

(*S*)-3-(4-(Methoxymethoxy)phenyl)-1-(trimethylsilyl)propan-1-ol (4f) was prepared as a colorless oil from **3f** (133 mg, 0.5 mmol) according to the General Procedure F (12 h, eluent: *n*-hexane/EtOAc = 15:1→10:1, 116.6 mg, 87% yield, 96:4 er).

$[\alpha]_D^{25}$: 10.0 (c = 1.0, $CHCl_3$). HPLC analysis of the product: Daicel CHIRALCEL OD-H; 10% *i*-PrOH in *n*-hexane; 1.0 mL/min; retention times: 7.9 min (major), 5.4 min (minor).

^{1}H NMR (300 MHz, $CDCl_3$) δ 7.16 (d, J = 8.6 Hz, 2H), 7.00 (d, J = 8.6 Hz, 2H), 5.18 (s, 2H), 3.51 (s, 3H), 3.41 – 3.29 (m, 1H), 2.96 – 2.83 (m, 1H), 2.70 – 2.54 (m, 1H), 1.93 – 1.70 (m, 2H), 1.18 (s, 1H), 0.06 (s, 9H).

^{13}C NMR (75 MHz, $CDCl_3$) δ 155.4, 135.6, 129.4, 116.3, 94.6, 65.4, 56.0, 35.5, 32.5, -4.0 ppm.

HR-MS (ESI) *m/z* Calcd. for $C_{14}H_{24}NaO_3Si^+$ [M+Na]$^+$: 291.1387, found: 291.1389.

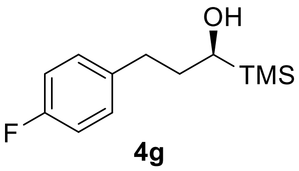

4g

(*S*)-3-(4-Fluorophenyl)-1-(trimethylsilyl)propan-1-ol (4g) was prepared as a colorless oil from **3g** (112 mg, 0.5 mmol) according to the General Procedure F (12 h, eluent: *n*-hexane/EtOAc = 15:1→10:1, 106.2 mg, 94% yield, 94:6 er).

$[\alpha]_D^{25}$: 12.4 (*c* = 1.0, $CHCl_3$). HPLC analysis of the product: Daicel CHIRALCEL OD-H; 10% *i*-PrOH in *n*-hexane; 1.0 mL/min; retention times: 4.3 min (major), 4.0 min (minor).

^{1}H NMR (300 MHz, $CDCl_3$) δ 7.19 (dd, *J* = 8.5, 5.6 Hz, 2H), 6.99 (t, *J* = 8.7 Hz, 2H), 3.34 (dd, *J* = 9.8, 4.4 Hz, 1H), 3.00 – 2.86 (m, 1H), 2.76 – 2.52 (m, 1H), 1.92 – 1.71 (m, 2H), 1.38 (s, 1H), 0.08 (s, 9H) ppm.

^{13}C NMR (75 MHz, $CDCl_3$) δ 161.3 (d, $^1J_{C\text{-}F}$ = 243.2 Hz), 137.9 (d, $^4J_{C\text{-}F}$ = 3.17 Hz), 129.8 (d, $^3J_{C\text{-}F}$ = 7.80 Hz), 115.1 (d, $^2J_{C\text{-}F}$ = 21.07 Hz), 65.3, 35.5, 32.4, -4.0 ppm.

^{19}F NMR {^{1}H} (282 MHz, $CDCl_3$) δ -117.7 (s) ppm.

HR-MS (ESI) *m/z* Calcd. for $C_{12}H_{19}FNaOSi^+$ $[M+Na]^+$: 249.1081, found: 249.1090.

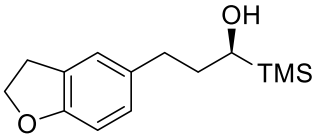

4h

(*S*)-3-(2,3-Dihydrobenzofuran-5-yl)-1-(trimethylsilyl)propan-1-ol (4h) was prepared as a colorless oil from **3h** (124 mg, 0.5 mmol) according to the General Procedure F (12 h, eluent: *n*-hexane/EtOAc = 15:1→10:1, 113.5 mg, 91% yield, 95:5 er).

$[\alpha]_D^{25}$: 11.7 (*c* = 1.0, $CHCl_3$). HPLC analysis of the product: Daicel CHIRALCEL OD-H; 10% *i*-PrOH in *n*-hexane; 1.0 mL/min; retention times: 7.9 min (major), 6.0 min (minor).

^{1}**H NMR** (400 MHz, $CDCl_3$) δ 7.08 (d, *J* = 1.9 Hz, 1H), 6.97 (dd, *J* = 8.1, 1.9 Hz, 1H), 6.74 (d, *J* = 8.1 Hz, 1H), 4.57 (t, *J* = 8.7 Hz, 2H), 3.41 – 3.32 (m, 1H), 3.21 (t, *J* = 8.7 Hz, 2H), 2.93 – 2.82 (m, 1H), 2.68 – 2.56 (m , 1H), 1.85 – 1.75 (m, 2H), 1.27 (s, 1H), 0.07 (s, 9H) ppm.

^{13}**C NMR** (101 MHz, $CDCl_3$) δ 158.3, 134.2, 127.8, 127.1, 125.0, 109.1, 71.2, 65.5, 35.8, 32.8, 29.8, -3.9 ppm.

HR-MS (ESI) *m/z* Calcd. for $C_{14}H_{22}NaO_2Si^+$ $[M+Na]^+$: 273.1281, found: 273.1280.

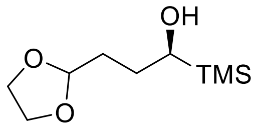

4i

(*S*)-3-(1,3-Dioxolan-2-yl)-1-(trimethylsilyl)propan-1-ol (4i) was prepared as a colorless oil from **3i** (101 mg, 0.5 mmol) according to the General Procedure F (12 h, eluent: *n*-hexane/EtOAc = 15:1→10:1, 97 mg, 95% yield, 94:6 er).

$[\alpha]_D^{25}$: -4.0 (*c* = 1.0, $CHCl_3$). HPLC analysis of the derived product (**(*S*)-3-(1,3-dioxolan-2-yl)-1-(trimethylsilyl)propyl naphthalene-2-sulfonate**): Daicel CHIRALCEL OD-H; 10% *i*-PrOH in *n*-hexane; 1.0 mL/min; retention times: 8.5 min (major), 9.5 min (minor).

^{1}**H NMR** (400 MHz, $CDCl_3$) δ 4.89 (t, *J* = 4.6 Hz, 1H), 4.03 – 3.94 (m, 2H), 3.93 – 3.80 (m, 2H), 3.28 (dd, *J* = 10.7, 3.4 Hz, 1H), 2.26 (s, 1H), 1.97 – 1.76 (m, 2H), 1.73 – 1.52 (m, 2H), 0.03 (s, 9H) ppm.

^{13}**C NMR** (100 MHz, $CDCl_3$) δ 104.6, 65.5, 64.9, 64.8, 31.7, 27.5, -4.0 ppm.

HR-MS (ESI) *m/z* Calcd. for $C_9H_{20}NaO_3Si^+$ $[M+Na]^+$: 227.1074, found: 227.1081.

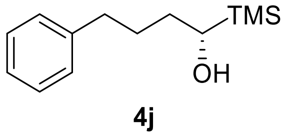

4j

4-Phenyl-1-(trimethylsilyl)butan-1-one (4j) was prepared as a colorless oil from **3j** (109 mg, 0.5 mmol) according to the General Procedure F (12 h, eluent:

n-hexane/EtOAc = 15:1→10:1, 102 mg, 93% yield, 89:11 er). ^{1}H NMR was consistent to the literature report.[28]

$[\alpha]_D^{25}$: 1.6 (c = 1.0, $CHCl_3$). HPLC analysis of the product: Daicel CHIRALCEL OD-H; 10% i-PrOH in n-hexane; 1.0 mL/min; retention times: 5.1 min (major), 5.9 min (minor).

^{1}H NMR (300 MHz, $CDCl_3$) δ 7.35 – 7.27 (m, 2H), 7.23 (d, J = 7.1 Hz, 3H), 3.37 (dd, J = 7.7, 5.9 Hz, 1H), 2.79 – 2.56 (m, 2H), 2.05 – 1.79 (m, 1H), 1.77 – 1.51 (m, 3H), 1.22 (s, 1H), 0.08 (s, 9H) ppm.

^{13}C NMR (75 MHz, $CDCl_3$) δ 142.5, 128.4, 128.3, 125.7, 65.9, 35.8, 33.3, 28.7, -3.9 ppm.

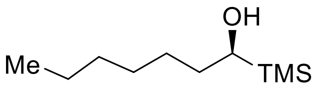

4k

(*S*)-1-(Trimethylsilyl)heptan-1-ol (4k) was prepared as a colorless oil from **3k** (93 mg, 0.5 mmol) according to the General Procedure F (12 h, eluent: n-hexane/EtOAc = 15:1→10:1, 89 mg, 95% yield, 90:10 er). ^{1}H NMR was consistent to the literature report.[29]

$[\alpha]_D^{25}$: -1.8 (c = 1.0, $CHCl_3$). HPLC analysis of the derived product (**(*S*)-1-(trimethylsilyl)heptyl naphthalene-2-sulfonate**): Daicel CHIRALPAK AD-H; 10% i-PrOH in n-hexane; 1.0 mL/min; retention times: 4.7 min (major), 5.1 min (minor).

^{1}H NMR (300 MHz, $CDCl_3$) δ 3.30 (dd, J = 9.0, 4.6 Hz, 1H), 1.63 – 1.43 (m, 3H), 1.41 – 1.17 (m, 8H), 0.98 – 0.81 (m, 3H), 0.05 (s, 9H) ppm.

^{13}C NMR (75 MHz, $CDCl_3$) δ 66.1, 33.5, 31.9, 29.3, 26.8, 22.7, 14.1, -3.9 ppm.

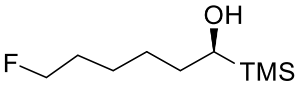

4l

(*S*)-6-Fluoro-1-(trimethylsilyl)hexan-1-ol (4l) was prepared as a colorless oil from **3l** (95 mg, 0.5 mmol) according to the General Procedure F (12 h, eluent: *n*-hexane/EtOAc = 15:1→10:1, 86 mg, 90% yield, 87:13 er).

$[\alpha]_D^{25}$: 3.7 (*c* = 1.0, $CHCl_3$). HPLC analysis of the derived product (**(*S*)-6-fluoro-1-(trimethylsilyl)hexyl naphthalene-2-sulfonate**): Daicel CHIRALPAK AD-H; 10% *i*-PrOH in *n*-hexane; 1.0 mL/min; retention times: 6.8 min (major), 7.6 min (minor).

^{1}H NMR (400 MHz, $CDCl_3$) δ 4.50 (t, *J* = 6.2 Hz, 1H), 4.38 (t, *J* = 6.1 Hz, 1H), 3.34 – 3.22 (m, 1H), 1.82 – 1.56 (m, 3H), 1.54 – 1.31 (m, 6H), 0.03 (s, 9H) ppm.

^{13}C NMR (100 MHz, $CDCl_3$) δ 84.1 (d, $^1J_{C-F}$ = 164.1 Hz), 65.8, 33.3, 30.4 (d, $^2J_{C-F}$ = 19.43 Hz), 26.5, 25.1 (d, $^3J_{C-F}$ = 5.44 Hz), -4.0 ppm.

^{19}F NMR {^{1}H} (282 MHz, $CDCl_3$) δ -218.1 (s) ppm.

HR-MS (ESI) *m/z* Calcd. for $C_9H_{21}FNaOSi^+$ [M+Na]$^+$: 215.1238, found: 215.1238.

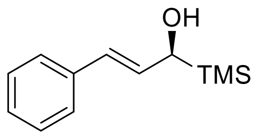

4m

(*S*,*E*)-3-Phenyl-1-(trimethylsilyl)prop-2-en-1-ol (4m) was prepared as a colorless oil from **3m** (102 mg, 0.5 mmol) according to the General Procedure F (12 h, eluent: *n*-hexane/EtOAc = 15:1→10:1, 93 mg, 90% yield, 95:5 er). ^{1}H NMR was consistent to the literature report.[30]

$[\alpha]_D^{25}$: -49.2 (*c* = 1.0, $CHCl_3$). HPLC analysis of the product: Daicel CHIRALPAK AD-H; 5% *i*-PrOH in *n*-hexane; 1.0 mL/min; retention times: 8.6 min (major), 8.1 min (minor).

^{1}H NMR (300 MHz, $CDCl_3$) δ 7.44 – 7.31 (m, 4H), 7.30 – 7.20 (m, 1H), 6.56 – 6.37 (m, 2H), 4.23 (d, *J* = 4.4 Hz, 1H), 1.67 (s, 1H), 0.15 (s, 9H) ppm.

^{13}C NMR (75 MHz, $CDCl_3$) δ 137.6, 132.0, 128.6, 126.9, 126.0, 125.5, 68.9, -4.0 ppm.

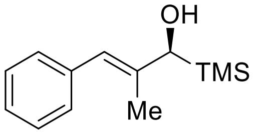

4n

(*S*,*E*)-2-Methyl-3-phenyl-1-(trimethylsilyl)prop-2-en-1-ol (4n) was prepared as a colorless oil from **3n** (109 mg, 0.5 mmol) according to the General Procedure F (12 h, eluent: *n*-hexane/EtOAc = 15:1→10:1, 96.5 mg, 88% yield, 92:8 er).

$[\alpha]_D^{25}$: -6.6 (*c* = 1.0, $CHCl_3$). HPLC analysis of the product: Daicel CHIRALPAK AD-H; 5% *i*-PrOH in *n*-hexane; 1.0 mL/min; retention times: 8.6 min (major), 8.1 min (minor).

^{1}H NMR (300 MHz, $CDCl_3$) δ 7.43 – 7.27 (m, 4H), 7.28 – 7.18 (m, 1H), 6.43 (t, *J* = 1.6 Hz, 1H), 4.05 (s, 1H), 1.91 (d, *J* = 1.4 Hz, 3H), 1.62 (d, *J* = 4.2 Hz, 1H), 0.16 (s, 9H) ppm.

^{13}C NMR (75 MHz, $CDCl_3$) δ 141.4, 138.3, 128.9, 128.1, 125.9, 121.5, 73.3, 16.8, -3.1 ppm.

HR-MS (ESI) *m/z* Calcd. for $C_{13}H_{20}NaOSi^+$ $[M+Na]^+$: 243.1176, found: 243.1175.

VII. Asymmetric Transfer Hydrogenation

General Procedure G.

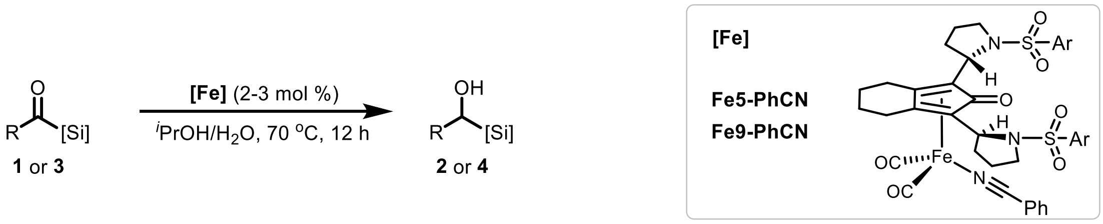

Under a nitrogen atmosphere, a 4-mL glass vial with a screw cap and a magnetic stir bar was charged with Fe-catalyst, (-)-**Fe9-PhCN,** 6.3 mg, 2 mol % for substrate **1**; **Fe5-PhCN,** 7.8 mg, 3 mol % for substrate **3**), solvent (1.5 mL ^{i}PrOH and 0.1 mL H_2O), and substrate **1** or **3** (0.3 mmol, 1.0 equiv.). The vial was tightly sealed with the screw cap. The mixture was stirred and heated in an oil bath at 70 °C for 12 hours. After cooling to room temperature, the solvent was removed under reduced pressure, and the residue was purified by silica gel column chromatography to obtain the desired product **2** or **4**.

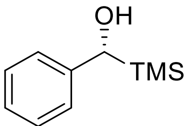

2a

(*R*)-Phenyl(trimethylsilyl)methanol (2a) was prepared as a colorless oil from **1a** (53.4 mg, 0.3 mmol) according to the General Procedure G (12 h, eluent: *n*-hexane/EtOAc = 20:1→15:1, 48.6 mg, 90% yield, 92:8 er).

HPLC analysis of the product: Daicel CHIRALCEL OD-H column; 10% *i*-PrOH in *n*-hexane; 1.0 mL/min; retention times: 7.4 min (major), 5.2 min (minor).

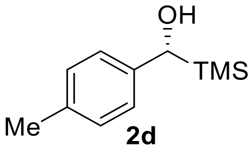

2d

(*R*)-*p*-Tolyl(trimethylsilyl)methanol (**2d**) was prepared as a colorless oil from **1d** (57.6 mg, 0.3 mmol) according to the General Procedure G (12 h, eluent: *n*-hexane/EtOAc = 20:1→15:1, 51.8 mg, 89% yield, 94:6 er).

HPLC analysis of the product: Daicel CHIRALCEL OD-H column; 10% *i*-PrOH in *n*-hexane; 1.0 mL/min; retention times: 5.9 min (major), 4.5 min (minor).

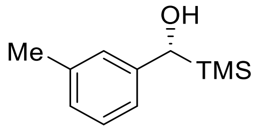

2f

(*R*)-*m*-Tolyl(trimethylsilyl)methanol (2f) was prepared as a colorless oil from **1f** (57.6 mg, 0.3 mmol) according to the General Procedure G (12 h, eluent: *n*-hexane/EtOAc = 20:1→15:1, 53.5 mg, 92% yield, 93:7 er).

HPLC analysis of the product: Daicel CHIRALCEL OD-H column; 10% *i*-PrOH in *n*-hexane; 1.0 mL/min; retention times: 5.2 min (major), 4.6 min (minor).

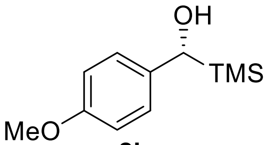

2h

(*R*)-(4-Methoxyphenyl)(trimethylsilyl)methanol (2h) was prepared as a colorless oil from **1h** (63 mg, 0.3 mmol) according to the General Procedure G (12 h, eluent: *n*-hexane/EtOAc = 20:1→15:1, 51.7 mg, 82% yield, 92:8 er).

HPLC analysis of the product: Daicel CHIRALCEL OD-H column; 10% *i*-PrOH in *n*-hexane; 1.0 mL/min; retention times: 7.1 min (major), 6.1 min (minor).

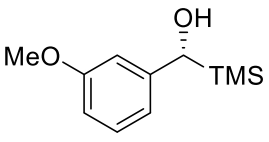

2i

(*R*)-(3-Methoxyphenyl)(trimethylsilyl)methanol (2i) was prepared as a colorless oil from **1i** (63 mg, 0.3 mmol) according to the General Procedure G (12 h, eluent: *n*-hexane/EtOAc = 20:1→15:1, 55.4 mg, 88% yield, 90:10 er).

HPLC analysis of the product: Daicel CHIRALCEL OD-H column; 10% *i*-PrOH in *n*-hexane; 1.0 mL/min; retention times: 16.2 min (major), 7.9 min (minor).

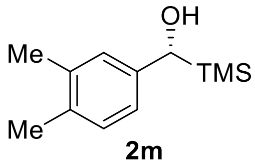

2m

(*R*)-(3,4-Dimethylphenyl)(trimethylsilyl)methanol (2m) was prepared as a colorless oil from **1m** (61.8 mg, 0.3 mmol) according to the General Procedure G (12 h, eluent: *n*-hexane/EtOAc = 20:1→15:1, 53.6 mg, 86% yield, 94:6 er).

HPLC analysis of the product: Daicel CHIRALCEL OD-H; 10% *i*-PrOH in *n*-hexane; 1.0 mL/min; retention times: 7.1 min (major), 4.9 min (minor).

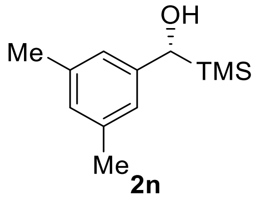

2n

(*R*)-(3,5-Dimethylphenyl)(trimethylsilyl)methanol (2n) was prepared as a colorless oil from **1n** (61.8 mg, 0.3 mmol) according to the General Procedure G (12 h, eluent: *n*-hexane/EtOAc = 20:1→15:1, 57.7 mg, 93% yield, 93:7 er).

HPLC analysis of the product: Daicel CHIRALCEL OD-H; 5% *i*-PrOH in *n*-hexane; 1.0 mL/min; retention times: 4.7 min (major), 4.3 min (minor).

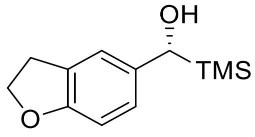

2q

(*R*)-(2,3-Dihydrobenzofuran-5-yl)(trimethylsilyl)methanol (2q) was prepared as a colorless oil from **1q** (66mg, 0.3 mmol) according to the General Procedure G (12 h, eluent: *n*-hexane/EtOAc = 10:1→5:1, 61.9 mg, 93% yield, 92:8 er).

HPLC analysis of the product: Daicel CHIRALCEL OD-H; 10% *i*-PrOH in *n*-hexane; 1.0 mL/min; retention times: 11.8 min (major), 8.5 min (minor).

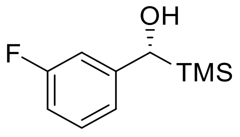

2t

(*R*)-(3-Fluorophenyl)(trimethylsilyl)methanol (2t) was prepared as a colorless oil from **1t** (58.8 mg, 0.3 mmol) according to the General Procedure G (12 h, eluent: *n*-hexane/EtOAc = 20:1→15:1, 52.3 mg, 88% yield, 92:8 er).

$[\alpha]_D^{25}$: -47.9 (c = 1.0, $CHCl_3$). HPLC analysis of the product: Daicel CHIRALCEL OD-H; 10% *i*-PrOH in *n*-hexane; 1.0 mL/min; retention times: 6.0 min (major), 4.5 min (minor).

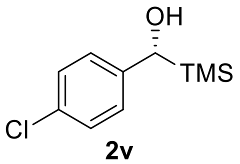

2v

(*R*)-(4-Chlorophenyl)(trimethylsilyl)methanol (2v) was prepared as a colorless oil from **1v** (63.6 mg, 0.3 mmol) according to the General Procedure G (12 h, eluent: *n*-hexane/EtOAc = 20:1→15:1, 58.4 mg, 91% yield, 91:9 er).

HPLC analysis of the product: Daicel CHIRALCEL OD-H; 10% *i*-PrOH in *n*-hexane; 1.0 mL/min; retention times: 5.6 min (major), 4.6 min (minor).

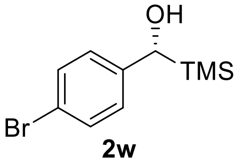

2w

(*R*)-(4-Bromophenyl)(trimethylsilyl)methanol (2w) was prepared as a colorless oil from **1w** (76.8 mg, 0.3 mmol) according to the General Procedure G (12 h, eluent: *n*-hexane/EtOAc = 20:1→15:1, 50.5 mg, 65% yield, 90:10 er).

HPLC analysis of the product: Daicel CHIRALCEL OD-H; 10% *i*-PrOH in *n*-hexane; 1.0 mL/min; retention times: 6.3 min (major), 5.0 min (minor).

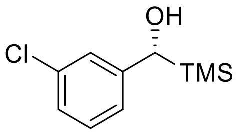

2x

(*R*)-(3-Chlorophenyl)(trimethylsilyl)methanol (2x) was prepared as a colorless oil from **1x** (63.6 mg, 0.3 mmol) according to the General Procedure G (12 h, eluent: *n*-hexane/EtOAc = 20:1→15:1, 57.6 mg, 90% yield, 92:8 er).

HPLC analysis of the product: Daicel CHIRALCEL OD-H; 10% *i*-PrOH in *n*-hexane; 1.0 mL/min; retention times: 6.9 min (major), 4.7 min (minor).

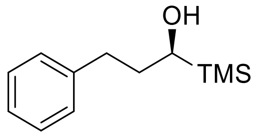

4a

(*S*)-3-Phenyl-1-(trimethylsilyl)propan-1-ol (4a) was prepared as a colorless oil from **3a** (61.8 mg, 0.3 mmol) according to the General Procedure G (12 h, eluent: *n*-hexane/EtOAc = 15:1→10:1, 49.6 mg, 80% yield, 95:5 er).

HPLC analysis of the product: Daicel CHIRALCEL OD-H; 10% *i*-PrOH in *n*-hexane; 1.0 mL/min; retention times: 7.6 min (major), 4.9 min (minor).

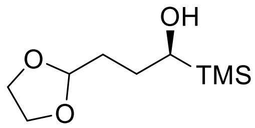

4i

(*S*)-3-(1,3-Dioxolan-2-yl)-1-(trimethylsilyl)propan-1-ol (4i) was prepared as a colorless oil from **3i** (61.2 mg, 0.3 mmol) according to the General Procedure G (12 h, eluent: *n*-hexane/EtOAc = 15:1→10:1, 52.6 mg, 86% yield, 92:8 er).

HPLC analysis of the derived product (**(*S*)-3-(1,3-dioxolan-2-yl)-1-(trimethylsilyl)propyl naphthalene-2-sulfonate**): Daicel CHIRALCEL OD-H; 10% *i*-PrOH in *n*-hexane; 1.0 mL/min; retention times: 8.2 min (major), 9.2 min (minor).

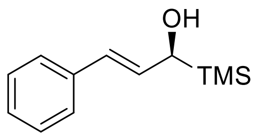

4m

(*S,E*)-3-Phenyl-1-(trimethylsilyl)prop-2-en-1-ol (4m) was prepared as a colorless oil from **3m** (61.8 mg, 0.3 mmol) according to the General Procedure G (12 h, eluent: *n*-hexane/EtOAc = 15:1→10:1, 49.5 mg, 80% yield, 94:6 er).

HPLC analysis of the product: Daicel CHIRALPAK AD-H; 5% *i*-PrOH in *n*-hexane; 1.0 mL/min; retention times: 9.2 min (major), 8.6 min (minor).

VIII. Gram-Scale Reaction and Product Derivatizations

Gram-scale synthesis of 2a

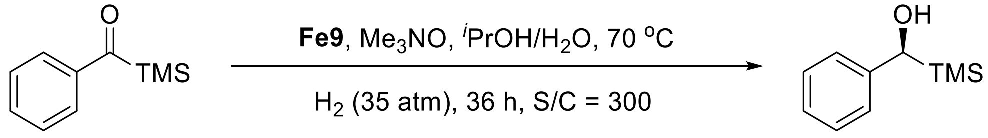

1a, 1.78 g   **2a**, 1.55 g, 86% yield, 92:8 er

Under nitrogen atmosphere, to a 10-mL glass vial equipped a magnetic stir bar, was added **Fe9** (32.5 mg, 0.033 mmol, 0.33 mol %), solvent (^{i}PrOH 5.0 mL and H_2O 0.1 mL), Me_3NO (5.0 mg, 0.066 mmol, 0.66 mol %) and substrate **1a** (1.78 g, 10 mmol, 1.0 equiv.). The via was transferred to a 50-mL autoclave and purged with H_2 two times (charge 10 atm H_2 and slowly release the H_2 each time), then the autoclave was charged with H_2 (35 atm). The autoclave was stirred and heated on a stir plate at 70 °C for 36 h. After cool down to ambient temperature, H_2 was carefully released. After removal of the solvent under reduced pressure, the residue was purified by silica gel column chromatography to afford the desired product **2a** (1.55 g, 86% yield, 92:8 er).

The experiment of S/C = 500 was carried out under the same condition, but using the following quantities: **1a** (0.89 g, 5 mmol, 1.0 equiv.), **Fe9** (9.8 mg, 0.01 mmol, 0.2 mol %), Me_3NO (2.5 mg, 0.02 mmol, 0.4 mol %), solvent (^{i}PrOH 3.0 mL and H_2O 0.1 mL). After work up, yielded **2a** (0.46 g, 51% yield, 92:8 er).

Turnover number experiment

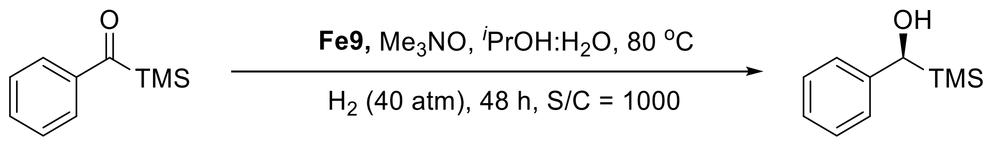

1a, 1.78 g   **2a**, 1.57 g, 87% yield, 91:9 er

Under nitrogen atmosphere, to a 10-mL glass vial equipped a magnetic stir bar, was added **Fe9** (9.8 mg, 0.01 mmol, 0.1 mol %), solvent (^{i}PrOH 5.0 mL and H_2O 0.1 mL), Me_3NO (2.5 mg, 0.02 mmol, 0.2 mol %) and substrate **1a** (1.78 g, 10 mmol, 1.0 equiv.). The via was transferred to a 50-mL autoclave and purged

with H_2 two times (charge 10 atm H_2 and slowly release the H_2 each time), then the autoclave was charged with H_2 (40 atm). The autoclave was stirred and heated on a stir plate at 80 °C for 48 h. After cool down to ambient temperature, H_2 was carefully released. After removal of the solvent under reduced pressure, the residue was purified by silica gel column chromatography to afford the desired product **2a** (1.57 g, 87% yield, 91:9 er).

Product derivatizations

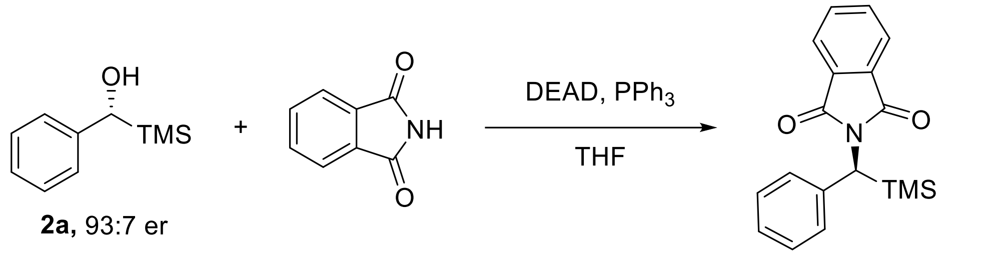

(*S*)-2-(Phenyl(trimethylsilyl)methyl)isoindoline-1,3-dione (5a). A mixture of corresponding **2a** (50.0 mg, 0.28 mmol, 93:7 er), isoindoline-1,3- dione (41.2 mg, 0.28 mmol), PPh_3 (146.9 mg, 0.56 mmol), THF (2 mL) and DEAD (97.5 mg, 0.56 mmol) was stirred at room temperature. After 24 h of stirring, H_2O (10 mL) was added to quench the reaction, and the mixture was transferred to a separatory funnel. The product was extracted with EtOAc (3 × 10 mL). The combined organic phases were dried over Na_2SO_4. The residue was purified by flash column chromatography on silica gel (eluent: *n*-hexane/EtOAc = 20:1→15:1) to give the pure product **5a** (77.0 mg, 89% yield, 93:7 er).

$[\alpha]_D^{25}$: 60.6 (c = 1.0, $CHCl_3$). HPLC analysis of the product: Daicel CHIRALPAK AD-H; 2% *i*-PrOH in *n*-hexane; 1.0 mL/min; retention times: 8.2 min (major), 7.7 min (minor).

^{1}H NMR (300 MHz, $CDCl_3$) δ 7.86 (dd, J = 5.5, 3.0 Hz, 2H), 7.73 (dd, J = 5.5, 3.1 Hz, 2H), 7.38 – 7.33 (m, 1H), 7.29 (d, J = 3.8 Hz, 3H), 7.23 – 7.15 (m, 1H), 4.73 (s, 1H), 0.19 (s, 9H) ppm.

^{13}**C NMR** (75 MHz, $CDCl_3$) δ 169.3, 139.9, 134.0 (2C), 132.0, 128.5 (3C), 127.6 (2C), 126.4, 123.3 (2C). 47.6, -1.3 ppm.

HR-MS (ESI) *m/z* Calcd. for $C_{18}H_{19}NNaO_2Si^+$ $[M+Na]^+$: 332.1077, found: 332.1077.

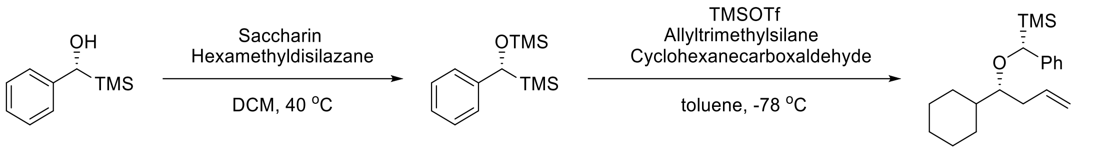

2a, 93:7 er **5b**, 87% yield **5c**, 85% yield, dr >20:1

To a solution of alcohol **2a** (50.0 mg, 0.28 mmol, 93:7 er) and saccharin (38.4 mg, 0.21 mmol) in 3 mL DCM at 40 °C was added hexamethyldisilazane (34.0 mg, 0.21 mmol, 0.75 equiv). The reaction mixture was stirred at 40 °C for 16 h, cooled to 25 °C, filtered through a cotton plug, and concentrated in vacuo. The residue was purified by flash column chromatography on silica gel (eluent: *n*-hexane/EtOAc = 80:1→60:1) to give the pure product **5b** (61.4 mg, 87% yield).

((*R*)-(((*R*)-1-Cyclohexylbut-3-en-1-yl)oxy)(phenyl)methyl)trimethylsilane (5c)

^{1}**H NMR** was consistent to the literature report.[31] To a cooled (–78 °C) 0.1 M solution of **5b** (60 mg, 0.24 mmol) in toluene were added allyltrimethylsilane (30.2 mg, 0.26 mmol), cyclohexanecarboxaldehyde (29.1 mg, 0.26 mmol), and TMSOTf (11.6 mg, 0.05 mmol). The reaction was stirred at –78 °C for 1 h whereupon saturated aqueous $NaHCO_3$ was added. Following extraction with EtOAc, the organic layer was washed with saturated aqueous $NaHCO_3$ and brine, dried over Na_2SO_4, and concentrated in vacuo. The residue was purified by flash column chromatography on silica gel (eluent: *n*-hexane/EtOAc = 80:1 →60:1) to give the pure product **5c** (65.0 mg, 85% yield).

^{1}**H NMR** (300 MHz, $CDCl_3$) δ 7.37 – 7.26 (m, 2H), 7.25 – 7.11 (m, 3H), 5.89 – 5.71 (m, 1H), 5.01 (s, 1H), 4.96 (d, *J* = 4.9 Hz, 1H), 4.21 (s, 1H), 3.27 – 3.10 (m, 1H), 2.26 – 2.06 (m, 2H), 1.87 – 1.57 (m, 6H), 1.36 – 0.99 (m, 5H), 0.01 (s, 9H) ppm.

^{13}**C NMR** (75 MHz, $CDCl_3$) δ 142.5, 136.9, 127.7, 126.5, 125.5, 115.7, 82.6, 39.7, 35.8, 28.8, 27.9, 26.9, 26.8, 26.6, -3.7 ppm.

$[\alpha]_D^{25}$: 49.5 (*c* = 1.0, $CHCl_3$).

IX. Mechanistic Studies

Deuterium-labeling experiments

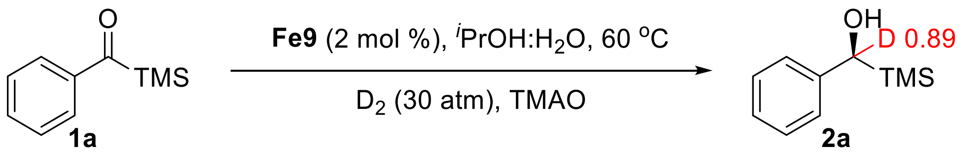

Under nitrogen atmosphere, to a 5-mL glass vial equipped a magnetic stir bar, was added **Fe9** (3.9 mg, 0.004 mmol, 2 mol %), solvent (^{i}PrOH 0.5 mL and H_2O 0.1 mL), Me_3NO (0.6 mg, 0.008 mmol, 4 mol %) and substrate **1a** (35.6 mg, 0.2 mmol, 1.0 equiv.). The via was transferred to a 50-mL autoclave and purged with D_2 two times (charge 10 atm D_2 and slowly release the D_2 each time), then the autoclave was charged with D_2 (30 atm). The autoclave was stirred and heated on a stir plate at 60 °C for 12 h. After cool down to ambient temperature, H_2 was carefully released. After removal of the solvent under reduced pressure, the residue was purified by silica gel column chromatography to afford the desired product **2a**. Integration of the ^{1}H NMR spectra indicated a deuteration of benzylic proton of 89 %.

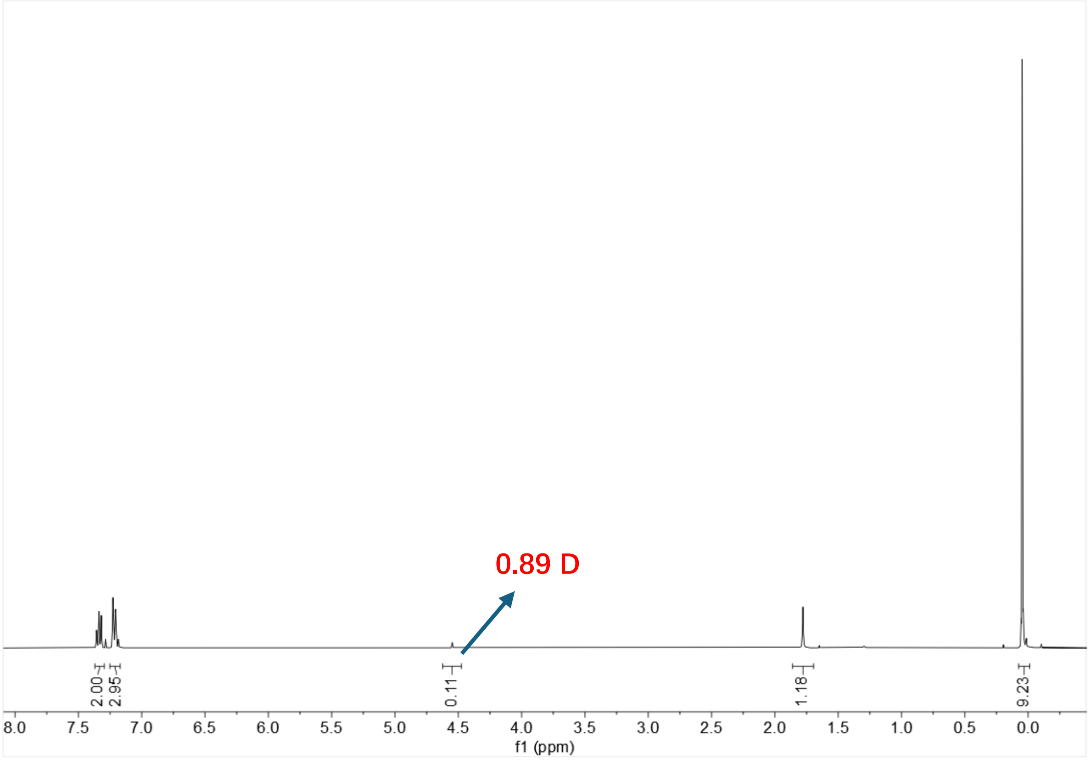

Supplementary Figure 1. ^{1}H NMR of **2a** using D_2 as deuterium source.

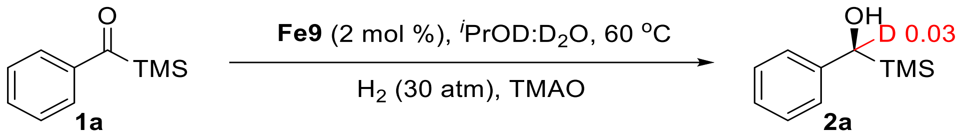

Under nitrogen atmosphere, to a 5-mL glass vial equipped a magnetic stir bar, was added **Fe9** (3.9 mg, 0.004 mmol, 2 mol %), solvent (ⁱPrOD 0.5 mL and D_2O 0.1 mL), Me_3NO (0.6 mg, 0.008 mmol, 4 mol %) and substrate **1a** (35.6 mg, 0.2 mmol, 1.0 equiv.). The via was transferred to a 50-mL autoclave and purged with H_2 two times (charge 10 atm H_2 and slowly release the H_2 each time), then the autoclave was charged with H_2 (30 atm). The autoclave was stirred and heated on a stir plate at 60 °C for 12 h. After cool down to ambient temperature, H_2 was carefully released. After removal of the solvent under reduced pressure, the residue was purified by silica gel column chromatography to afford the desired product **2a**. Integration of the ^1H NMR spectra indicated a deuteration of benzylic proton of 3 %.

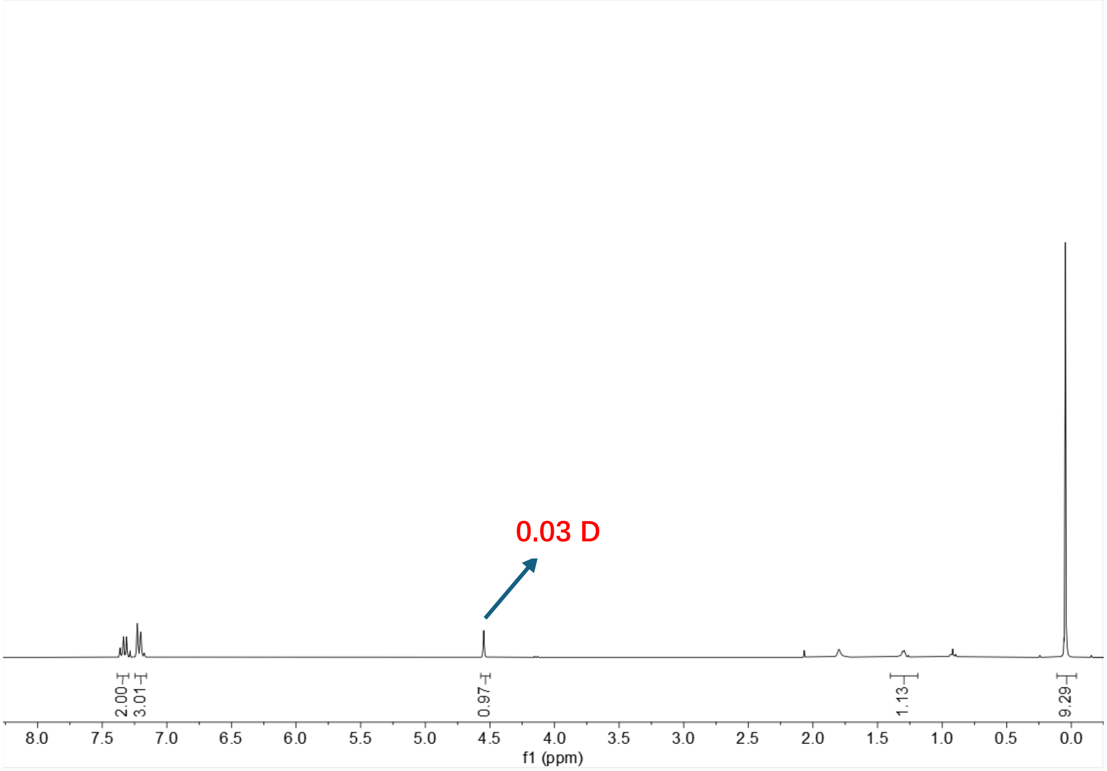

Supplementary Figure 2. ^1H NMR of **2a** using ⁱPrOD/D_2O as deuterium source.

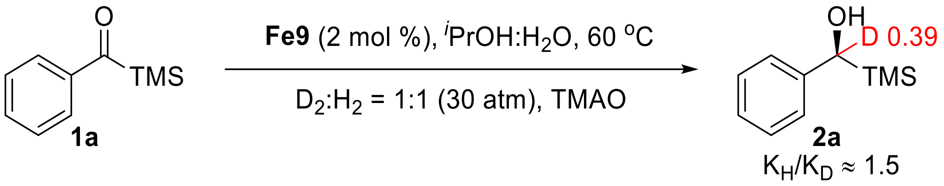

Under nitrogen atmosphere, to a 5-mL glass vial equipped a magnetic stir bar, was added **Fe9** (3.9 mg, 0.004 mmol, 2 mol %), solvent (^{i}PrOH 0.5 mL and H_2O 0.1 mL), Me_3NO (0.6 mg, 0.008 mmol, 4 mol %) and substrate **1a** (35.6 mg, 0.2 mmol, 1.0 equiv.). The via was transferred to a 50-mL autoclave and purged with H_2 two times (charge 10 atm H_2 and slowly release the H_2 each time), then the autoclave was charged with H_2 (15 atm) and the autoclave was charged with D_2 (15 atm). The autoclave was stirred and heated on a stir plate at 60 °C for 12 h. After cool down to ambient temperature, H_2 and D_2 was carefully released. After removal of the solvent under reduced pressure, the residue was purified by silica gel column chromatography to afford the desired product **2a**. Integration of the ^{1}H NMR spectra indicated a deuteration of benzylic proton of 39 %.

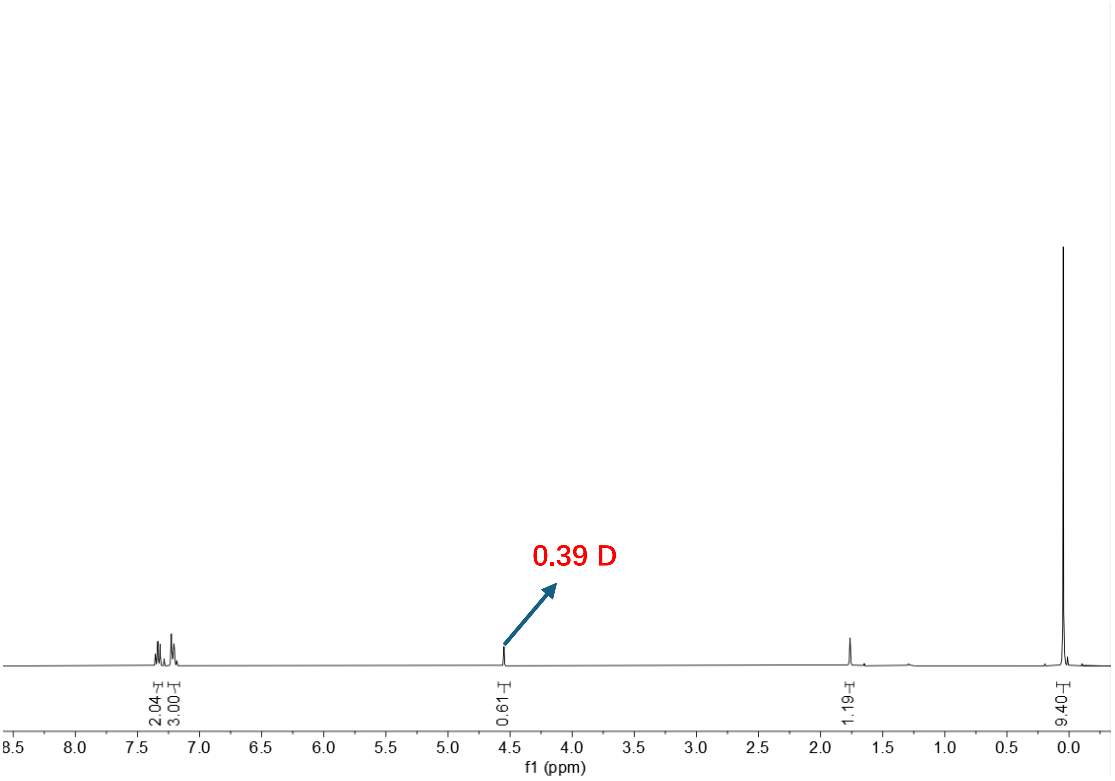

Supplementary Figure 3. ^{1}H NMR of **2a** using D_2 as deuterium source.

Control Experiment

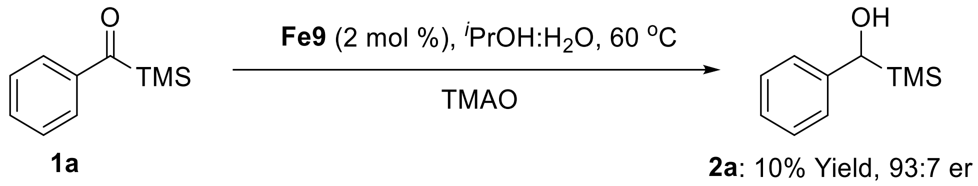

Under nitrogen atmosphere, **1a** (35.6 mg, 0.2 mmol, 1.0 equiv.), **Fe9** (3.9 mg, 0.004 mmol, 2 mol %), Me_3NO (0.6 mg, 0.008 mmol, 4 mol %) and ^{i}PrOH/H_2O (9:1, 1.0 mL) were added to a 10-mL vial equipped with a magnetic stir bar. The vial was strictly sealed and stirred at 60 °C for 12 h. After cool down to room temperature, the solvent was removed under reduced pressure. The residue was determined by ^{1}H NMR analysis using CH_2Br_2 as an internal standard (10 % yield). HPLC analysis revealed the same enantiomeric ratio with H_2 (93:7 er).

Non-linear effect

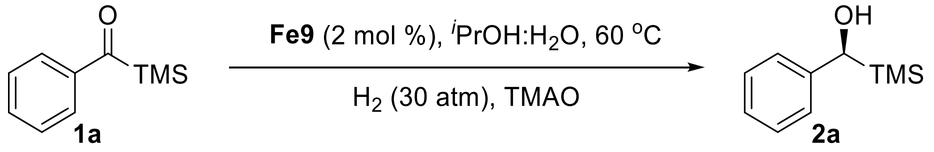

1a (35.6 mg, 0.2 mmol, 1.0 equiv.), **Fe9** (3.9 mg, 0.004 mmol, 2 mol %) with different enantiomeric excess (1st run: 0% ee, 2nd run: 20% ee; 3rd run: 40% ee; 4th run: 60% ee; 5th run: 80% ee; 6th run: 100% ee) were placed in a hydrogenation tube. The tube was transferred to the nitrogen-filled glovebox, and ^{i}PrOH:H_2O (4:1, 0.6 mL) was added. The reaction was stirred under H_2 (30 atm) at 60 °C in the stainless steel autoclave for 12 hours. The residue was purified by column chromatography on silica gel (*n*-hexane/EtOAc = 15 : 1) to afford the desired product.

Ee of **Fe9**	0	20	40	60	80	100
Ee of **2a**	1.1	15.1	33.3	51	69	86

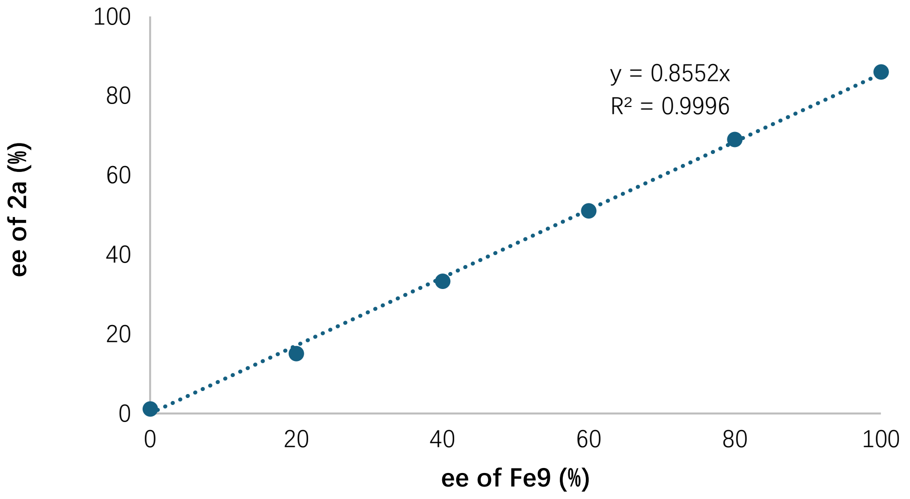

Supplementary Figure 4. Non-linear effect experiment.

Dimensional NMR Test of Fe4

The NMR test was measured in $CDCl_3$ on a 600 MHz NMR instrument. First, Heteronuclear Single Quantum Coherence (HSQC) experiment was tested to indicate **H**^{c} and **H**^{d} are bonded to the same carbon **C**^{1}, and **H**^{c} and **H**^{d} are bonded to the same carbon **C**^{2}.

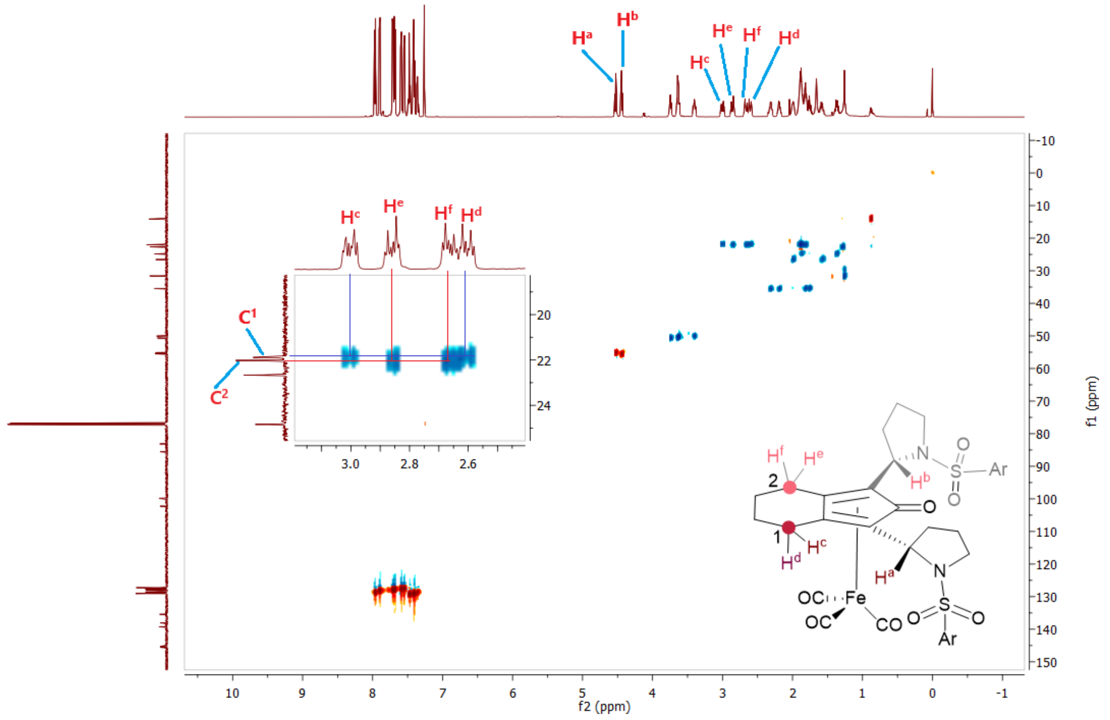

Supplementary Figure 5. HSQC spectroscopy of **Fe4**.

Next, ¹H two-dimensional NOE spectroscopy (NOESY) was measured to illustrate the **H^c^/H^d^** have strong correlation to **H^a^**, and **H^e^/H^f^** only show weak correlation to **H^b^**.

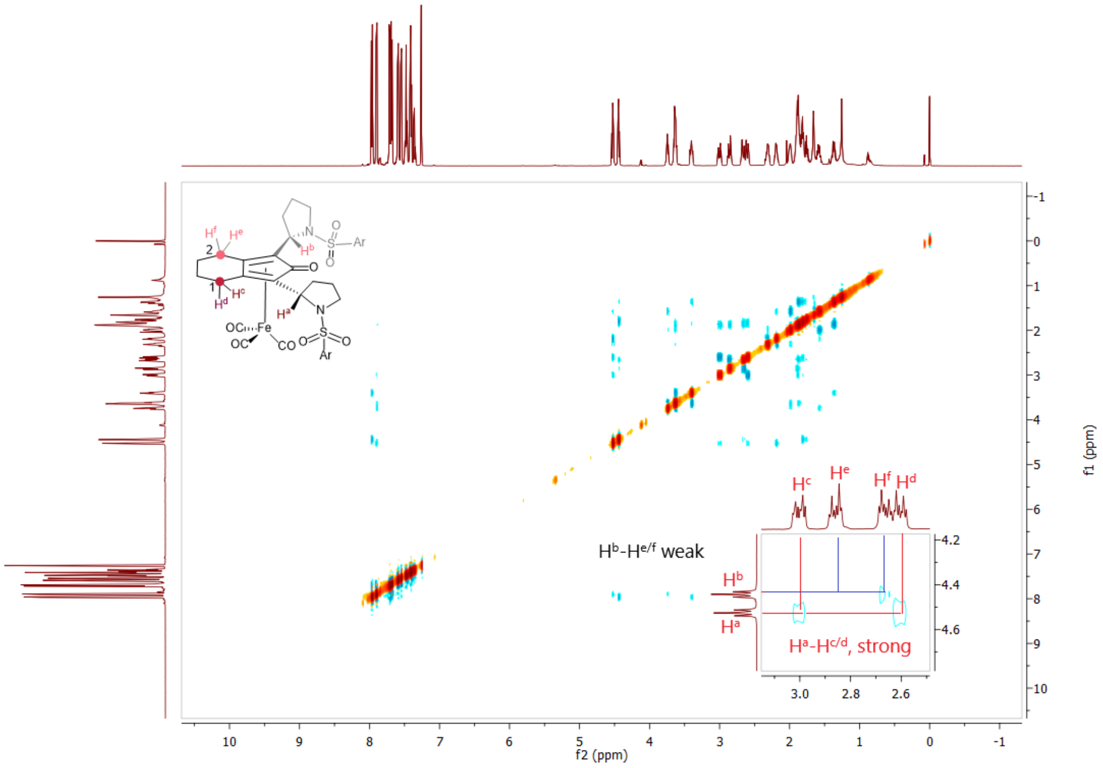

Supplementary Figure 6. NOE spectroscopy (NOESY) of **Fe4**.

X. X-ray Crystallography of Fe4

The structure and absolute stereochemistry of the **Fe4** were determined by X-ray crystallography. Single crystals of **Fe4** were obtained from dichloromethane and *n*-hexane by vapor deposition. The crystal is a complex combining two DCM molecues. The X-ray data of compound **Fe4·(DCM)₂** have been deposited at the Cambridge Crystallographic Data Center (CCDC 2446024). Molecular structure of **Fe4** with thermal displacement parameters drawn at 50% probability.

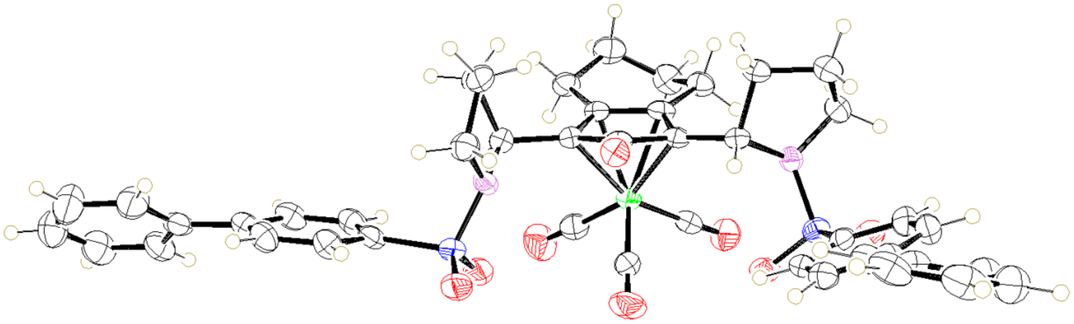

Supplementary Figure 7. Crystal structure of **Fe4**.

Supplementary Table 5. Sample and crystal data for Fe4(DCM)₂.

CCDC	2446024	
Chemical formula	$C_{46}H_{44}Cl_4FeN_2O_8S_2$	
Formula weight	1014.60 g/mol	
Temperature	213(2) K	
Wavelength	0.71073 Å	
Crystal system	monoclinic	
Space group	P 1 21 1	
Unit cell	a = 7.434(2) Å	α = 90°
	b = 21.061(5) Å	β = 94.935(10)°
	c = 15.047(3) Å	γ = 90°
Volume	2347.1(10) $Å^3$	
Z	2	
Density (calculated)	1.436 g/cm^3	

Absorption coefficient	0.692 mm^{-1}
F(000)	1048

XI. Computational Details

All calculations were performed using Gaussian 16, Revision A.03 package.[32] All of the reactants, intermediates, transition states, products were optimized by the DFT with the ωB97X-D functional.[33] For geometry optimizations and frequency calculations, BS-I basis set system was employed. In BS-I, we employed LANL2DZ basis set for Fe with effective core potentials, 6-31G(d) basis sets for C, H, O, N, S, and Si. All the stationary structures were characterized with no imaginary frequency and the transition state structures (TSs) were characterized with a single imaginary frequency. Intrinsic reaction coordinate (IRC) calculations were performed on the TSs. The solvent effect of toluene was evaluated through the SMD method, [34] in which a better basis set system BS-II was used. In BS-II, we employed SDD basis set for Fe with effective core potentials, 6-311++G(2d,2p) basis sets for C, H, O, N, S, and Si. The weak interaction was analyzed using the Multiwfn program.[35] All reported energies are free energies at a concentration of 1 M and a temperature of 298.15 K.

All calculations were carried out based on **Fe4**. At first, we have calculated the spin states of all Fe complexes involved in the reaction pathway, and the corresponding Gibbs free energies are presented in **Fig. S1**.

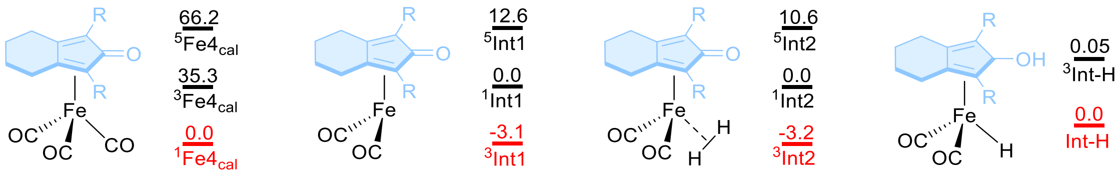

Supplementary Figure 8. The relative free energies of Fe complexes in different spin states.

Full catalytic cycle

As illustrated in Fig. S2, the catalytic cycle starts from activation of the pre-catalyst ^{1}**Fe4$_{cal}$** initiates with Me_3NO-assisted CO ligand dissociation, accompanied by the release of CO_2 and Me_3N. This process yields intermediate

¹Int1 with a favorable free energy of -51.3 kcal·mol⁻¹. The corresponding transition state **¹TS1** presents an activation barrier of 12.2 kcal·mol⁻¹, indicating both kinetic accessibility and thermodynamic favorability. Subsequently, **¹Int1** undergoes a spin crossover via the minimum energy crossing point (**MECP**), affording the more stable triplet intermediate **³Int1**. Association of H_2 to **³Int1** generates intermediate **³Int2**, which undergoes a spin crossover through **MECP**, leading to the singlet intermediate **¹Int2**. The latter proceeds via **¹TS2**, involving H–H bond cleavage ($\Delta G^{\ddagger}$ = 27.0 kcal·mol⁻¹), representing the rate-determining step. **Int-H** then reacts with **1a** to form **¹Int3**, followed by a concerted hydrogen transfer through **¹TS*S*** ($\Delta G^{\ddagger}$ = 13.7 kcal·mol⁻¹) to afford the desired product **2a**. This final step is thermodynamically favorable, and **¹Int1** undergoes spin crossover via **MECP** to regenerate **³Int1**, thereby completing the catalytic cycle.

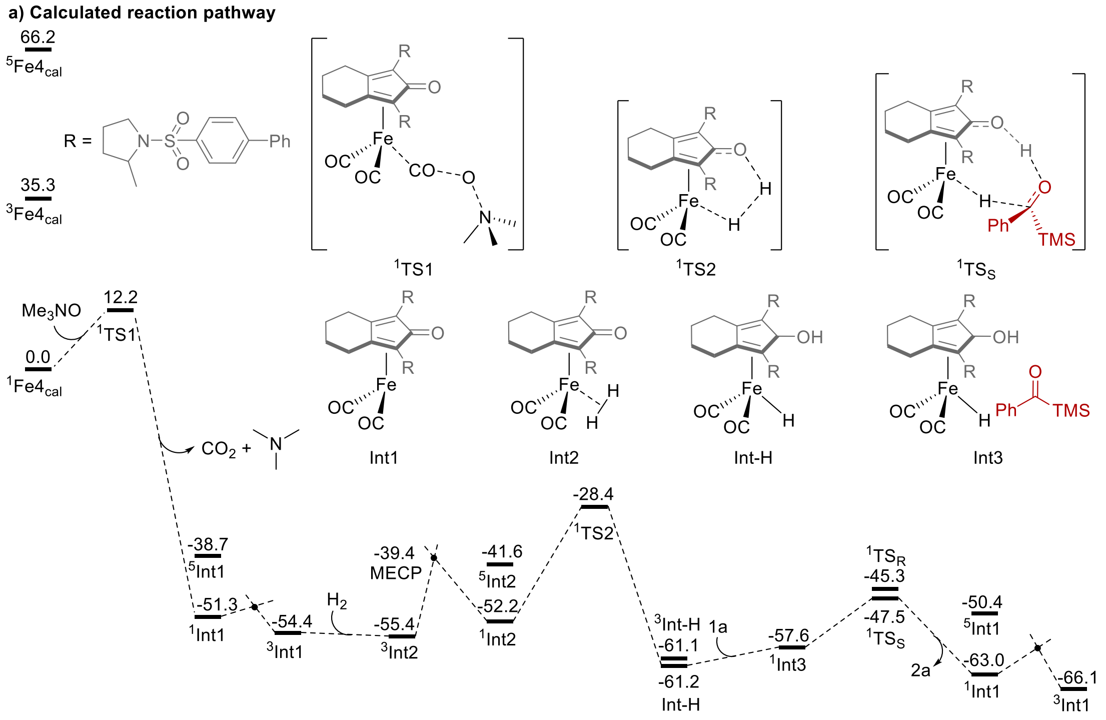

Supplementary Figure 9. DFT calculated free energies of the whole catalytic cycles.

In Figure S3, structural analysis provided key insights into the **¹Fe4cal** catalyst's unique architecture, particularly the downward orientation of its $-SO_2R$ arms.

Fig. S3 reveals that the steric demand of the CH_2 group on the five-membered ring exceeds that of the N group, driving the arm downward. Additional stabilization arises from C–H···O interactions (b_1 = 2.969 Å, b_2 = 3.399 Å) between CO ligands and arm hydrogens, maintaining this orientation and creating a chiral catalytic framework. This arrangement results in distinct spatial environments for the two arms, generating a special chiral environment that enhances enantioselectivity. The activated intermediate **Int-H** preserves this structural asymmetry, with a hydrogen bond (b_3 = 1.786 Å) maintaining the chiral pocket for subsequent asymmetric hydrogenation.

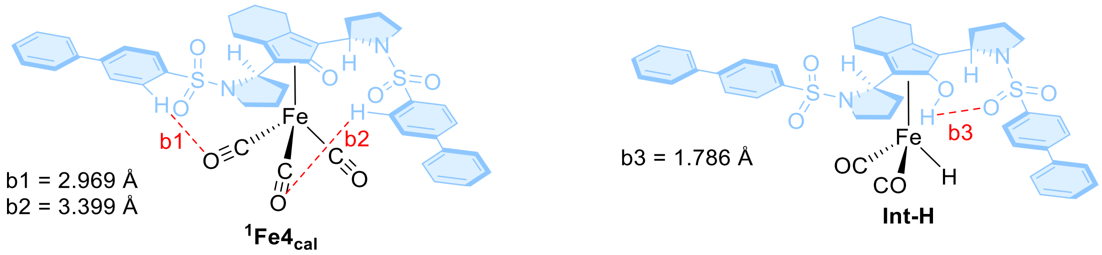

Supplementary Figure 10. Schematic structures of the pre-catalyst ^{1}**Fe4**$_{cal}$ and intermediate **Int-H**

In Fig. S4, the non-covalent interaction (NCI) analysis of the enantiodetermining transition state **TS**$_S$ revealed critical stabilizing interactions, including C–H···π (b_4 = 2.881 Å, b_5 = 2.567 Å) and C–H···O (b_6 = 2.354 Å) contacts. In contrast, the disfavored transition state **TS**$_R$ lacked comparable interactions, providing a structural basis for the observed enantiocontrol in the [Fe]-catalyzed transformation.

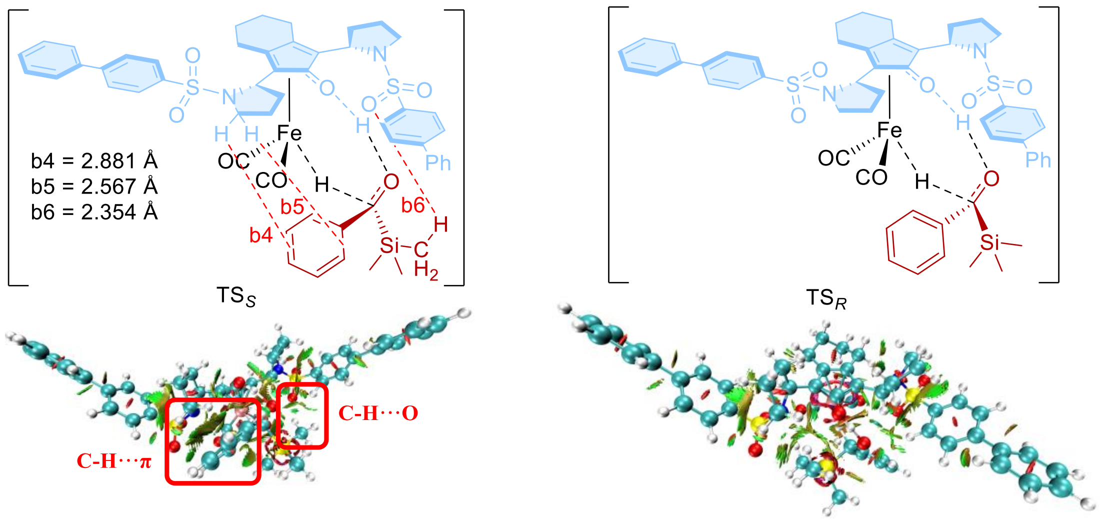

Supplementary Figure 11. Non-covalent interaction analysis of transition state **TS*S*** and **TS*R*.**

Comparison of tert-butyl group with trimethylsilyl group

During the implementation of our experiments, we found the silyl group is important for enantiocontrol. When we change the TMS in **1a** to *t*-Bu, the enantiomeric ratio dropped significantly.

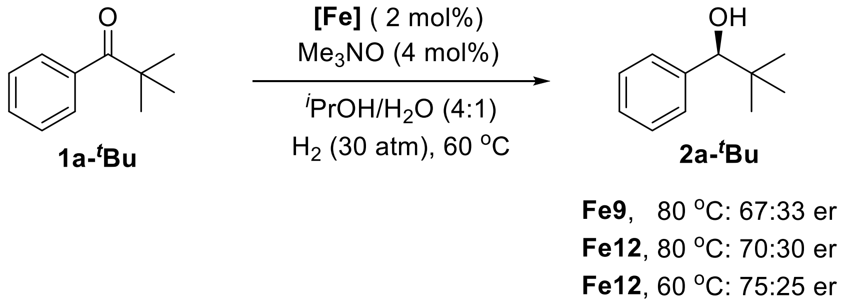

To clarify the poor performance of **1a-*t*Bu**, we checked the key transition state for enantiocontrol of **1a-*t*Bu** (Figure S5). Theoretical calculations indicate that this phenomenon mainly arises from the larger atomic radius of silicon compared to carbon, which enables the silyl group to form a stronger C–H···O

weak interaction with the catalyst (b_6 = 2.354 Å). Although the tert-butyl group can also participate in a similar interaction, the longer interaction distance (b_9 = 2.614 Å) leads to a weaker stabilizing effect relative to the silyl group.

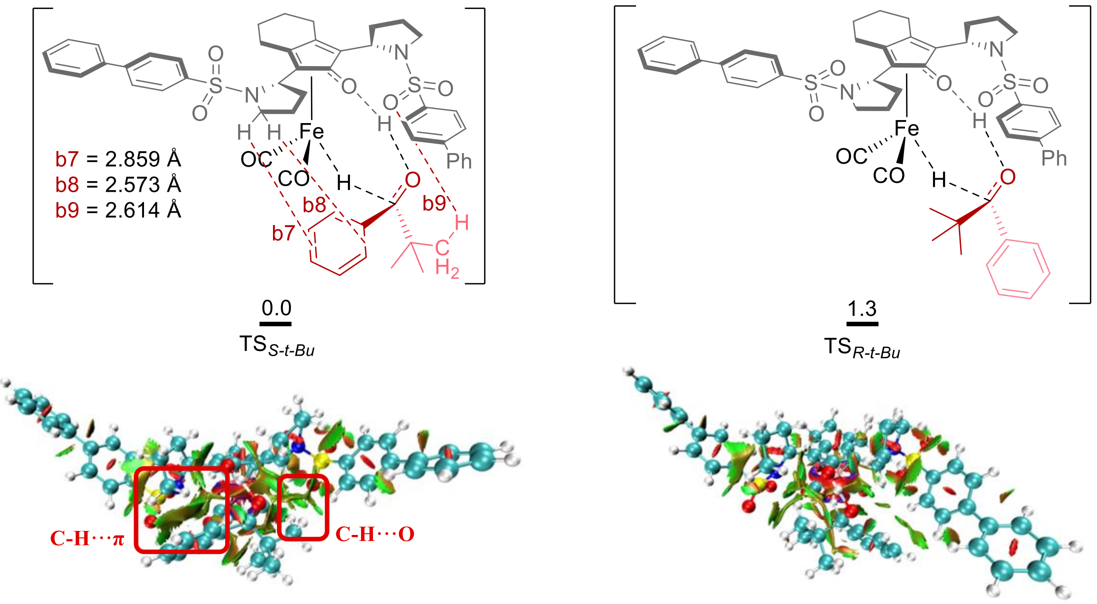

Supplementary Figure 12. | Non-covalent interaction analysis of transition state **TS*S-t-Bu*** and **TS*R-t-Bu***.

XII.NMR Spectra

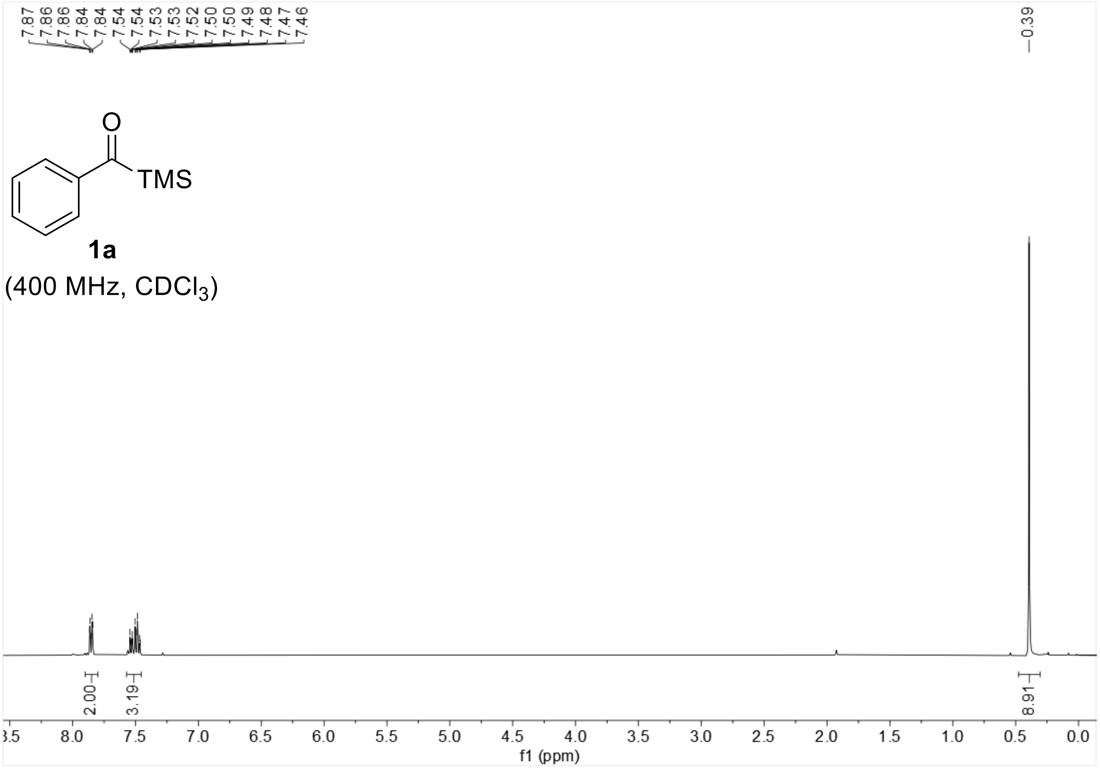

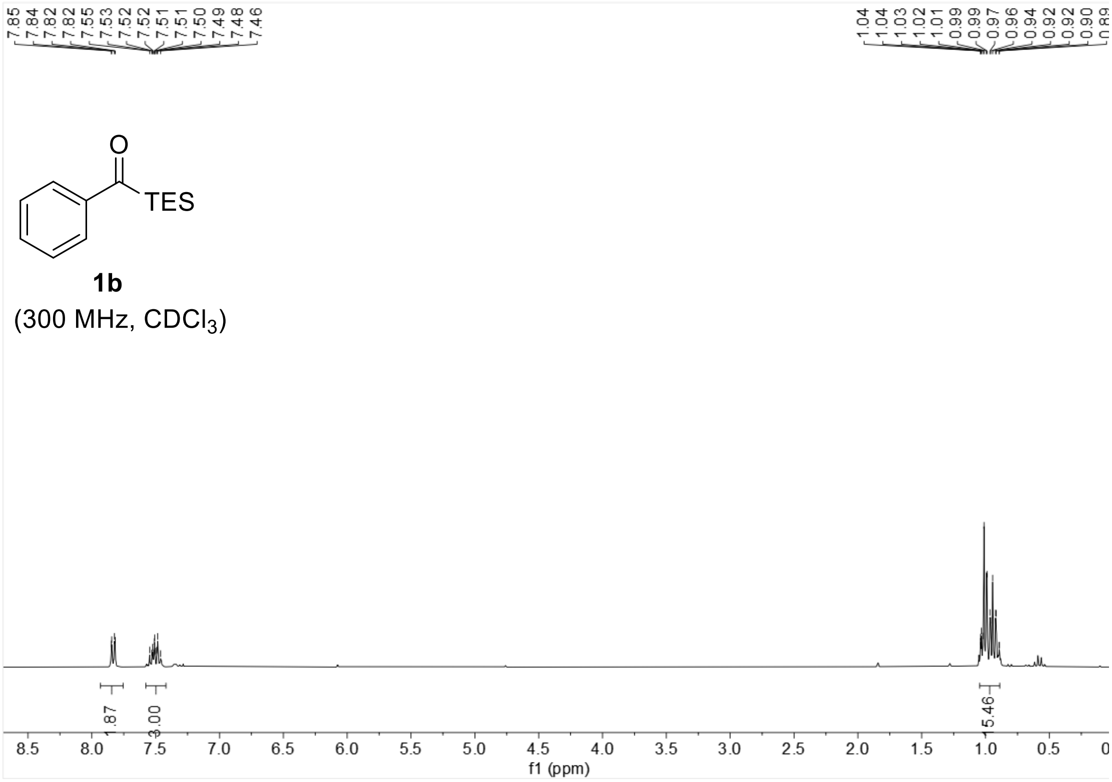

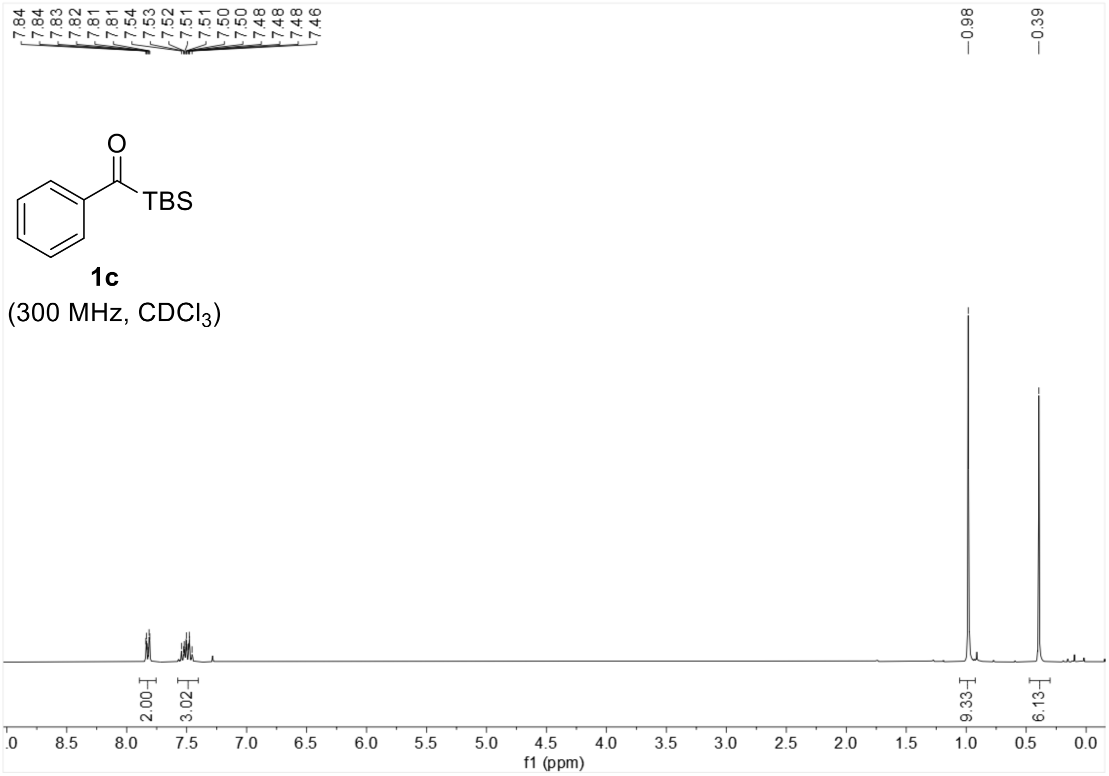
1c
(300 MHz, CDCl3)
f1 (ppm)

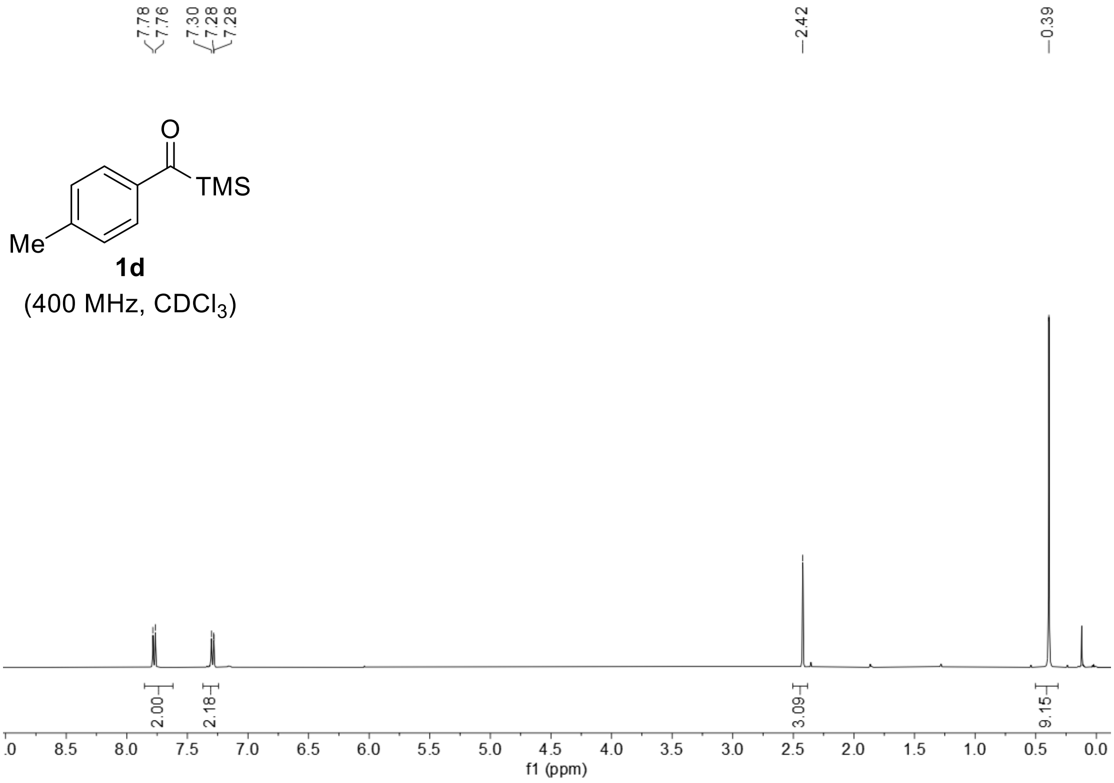
1d
(400 MHz, CDCl3)
f1 (ppm)

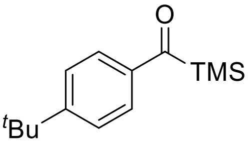

1e

(300 MHz, $CDCl_3$)

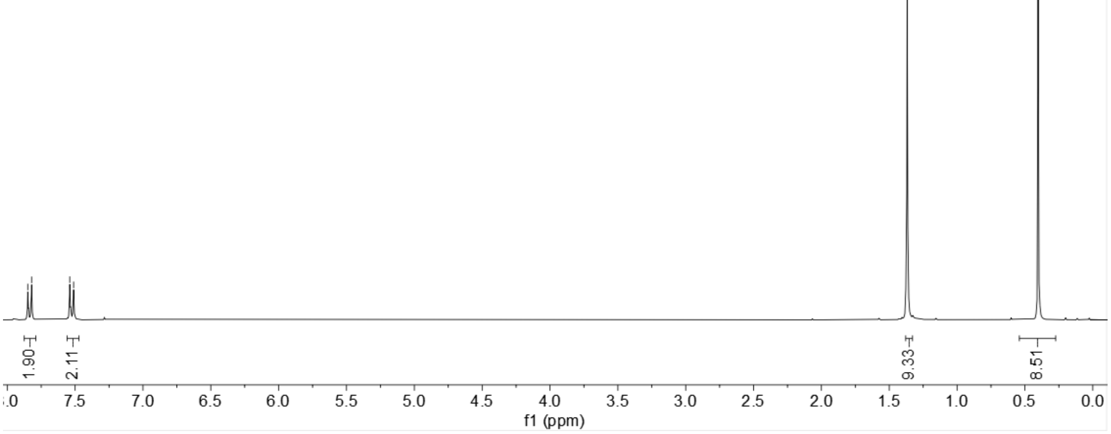

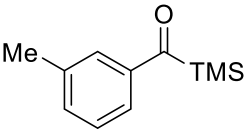

1f

(300 MHz, $CDCl_3$)

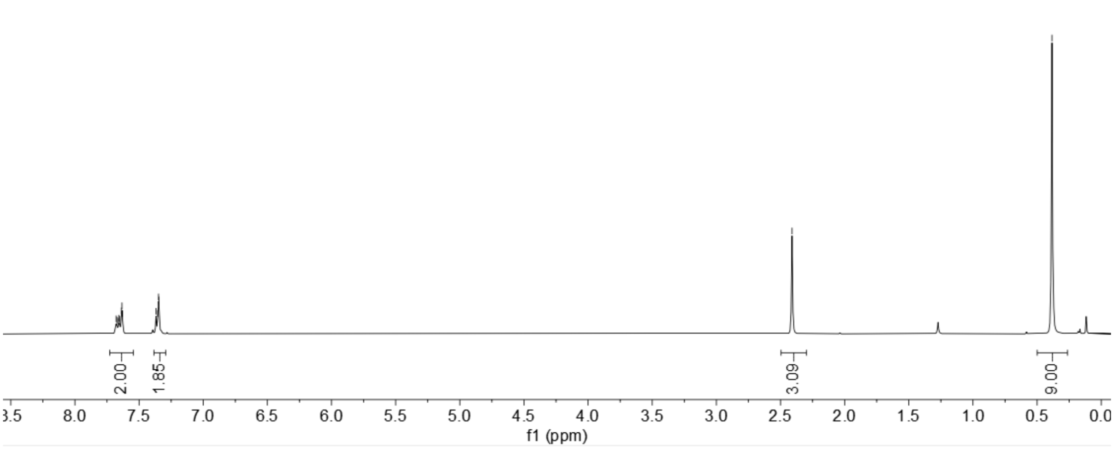

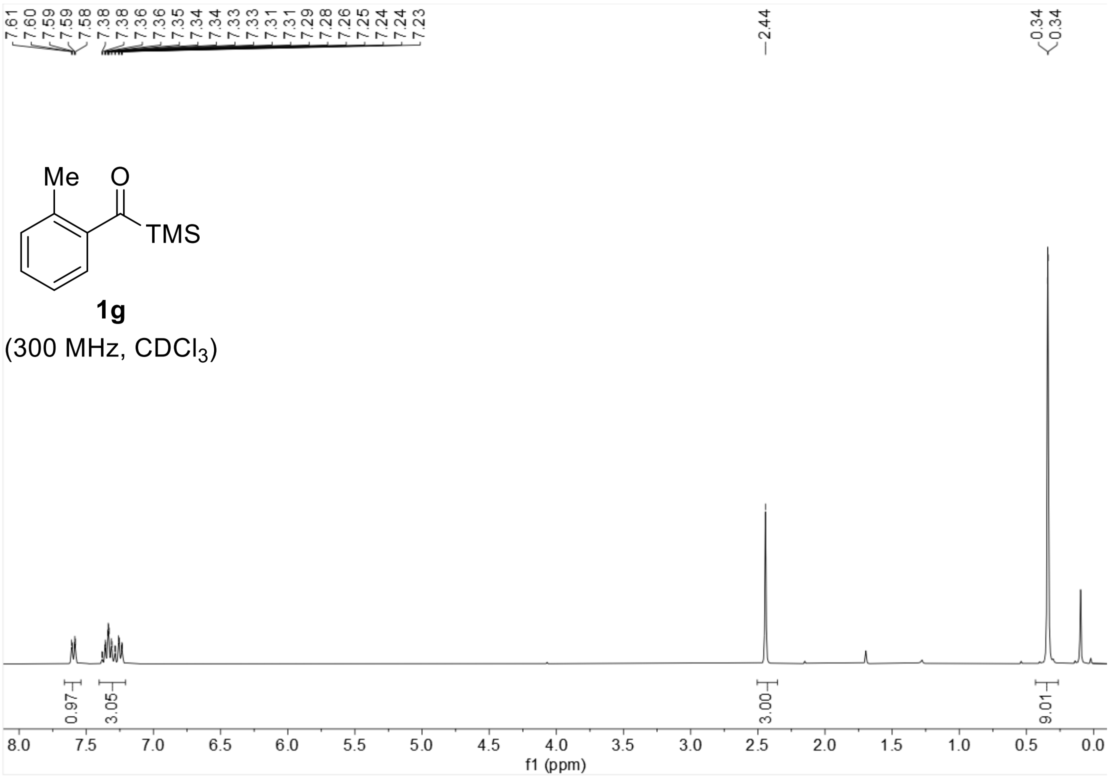

1g
(300 MHz, $CDCl_3$)

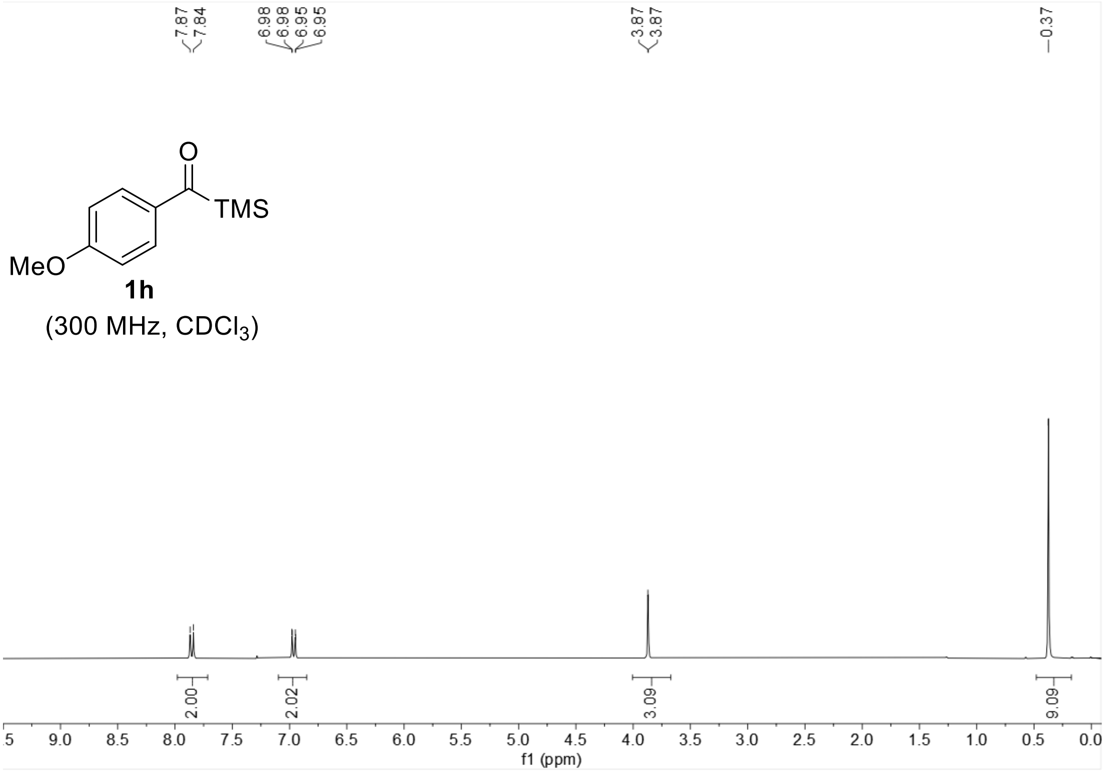

1h
(300 MHz, $CDCl_3$)

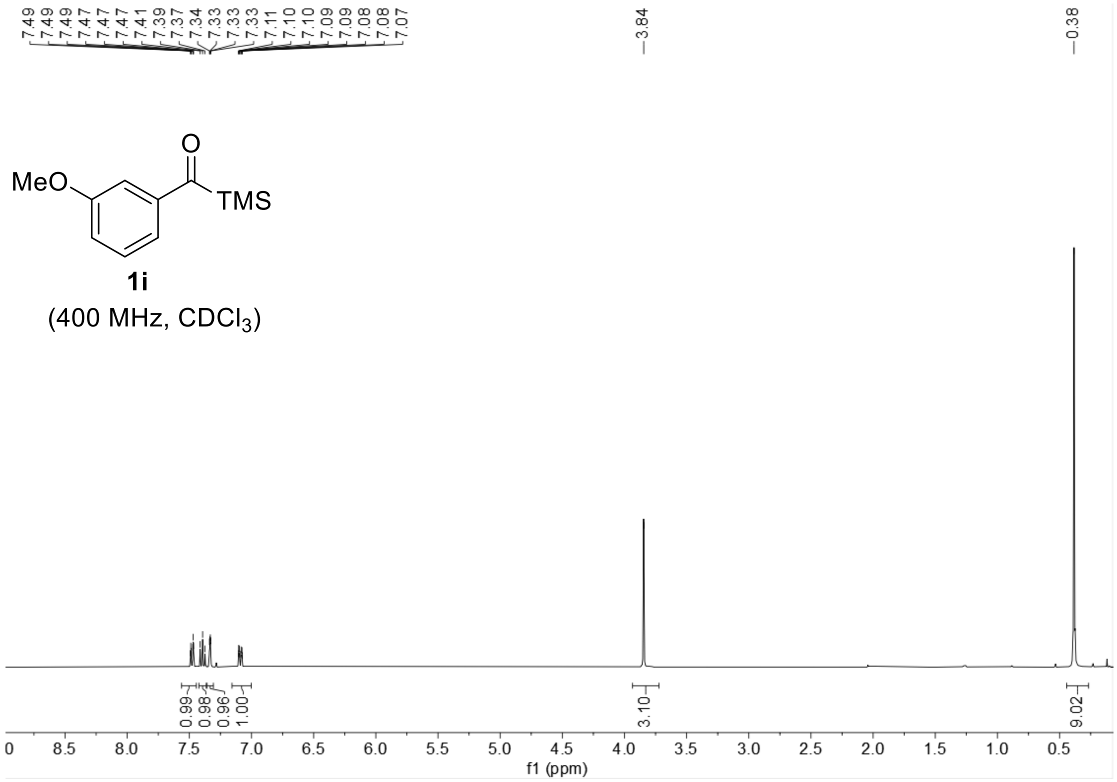
7.49
7.49
7.49
7.47
7.47
7.47
7.41
7.39
7.37
7.34
7.33
7.33
7.33
7.11
7.10
7.10
7.09
7.09
7.08
7.08
7.07
3.84
0.38
MeO
O
TMS
1i
(400 MHz, CDCl3)
0.99
0.98
0.96
1.00
3.10
9.02
f1 (ppm)

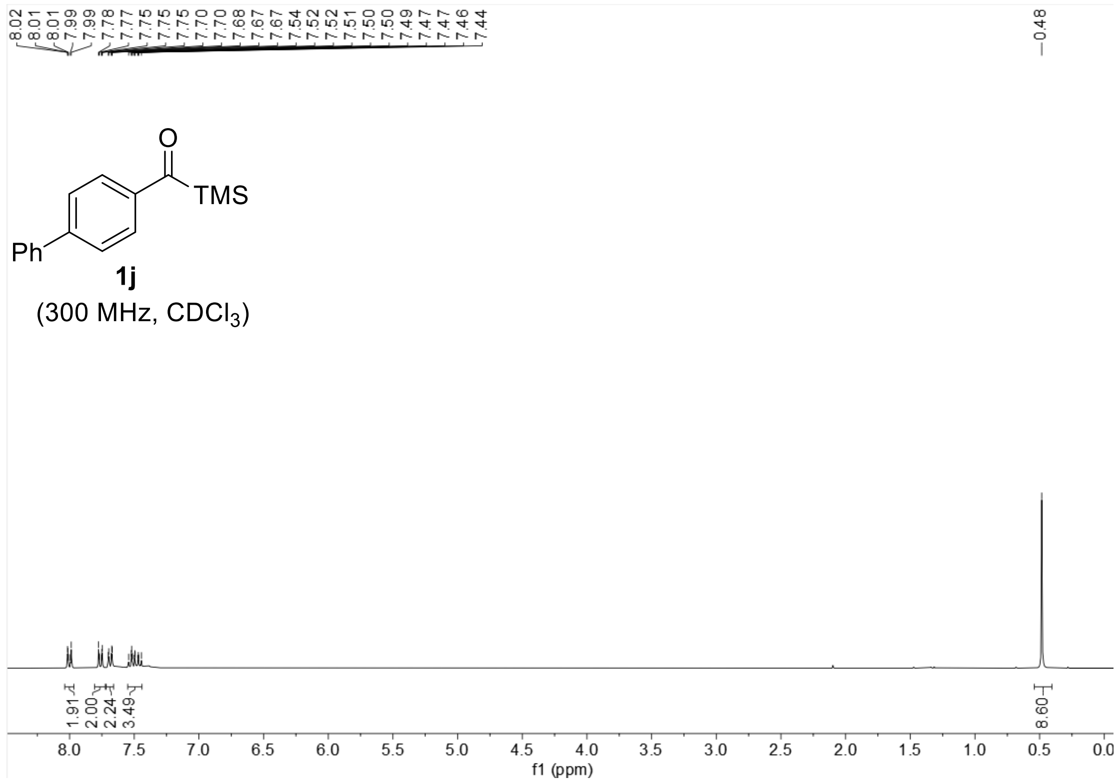
8.02
8.01
8.01
7.99
7.99
7.78
7.77
7.75
7.75
7.75
7.70
7.70
7.68
7.67
7.67
7.54
7.52
7.51
7.50
7.49
7.47
7.47
7.46
7.44
0.48
O
TMS
Ph
1j
(300 MHz, CDCl3)
1.91
2.00
2.24
3.49
8.60
f1 (ppm)

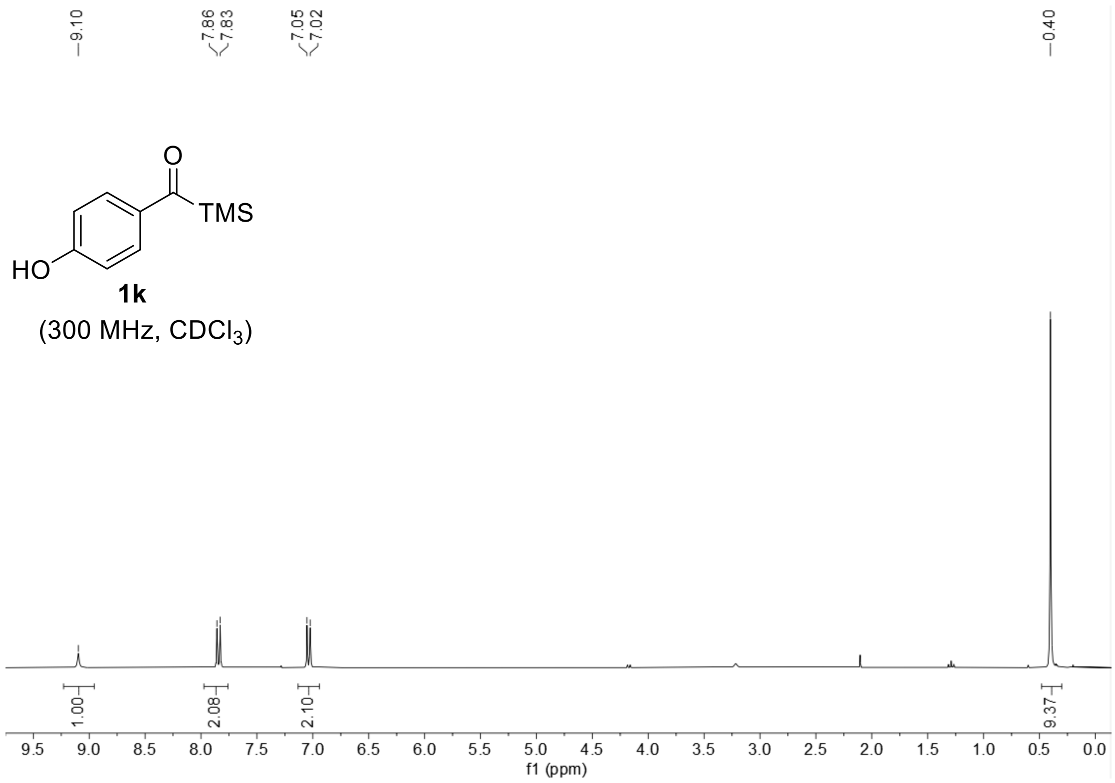

1k

(300 MHz, $CDCl_3$)

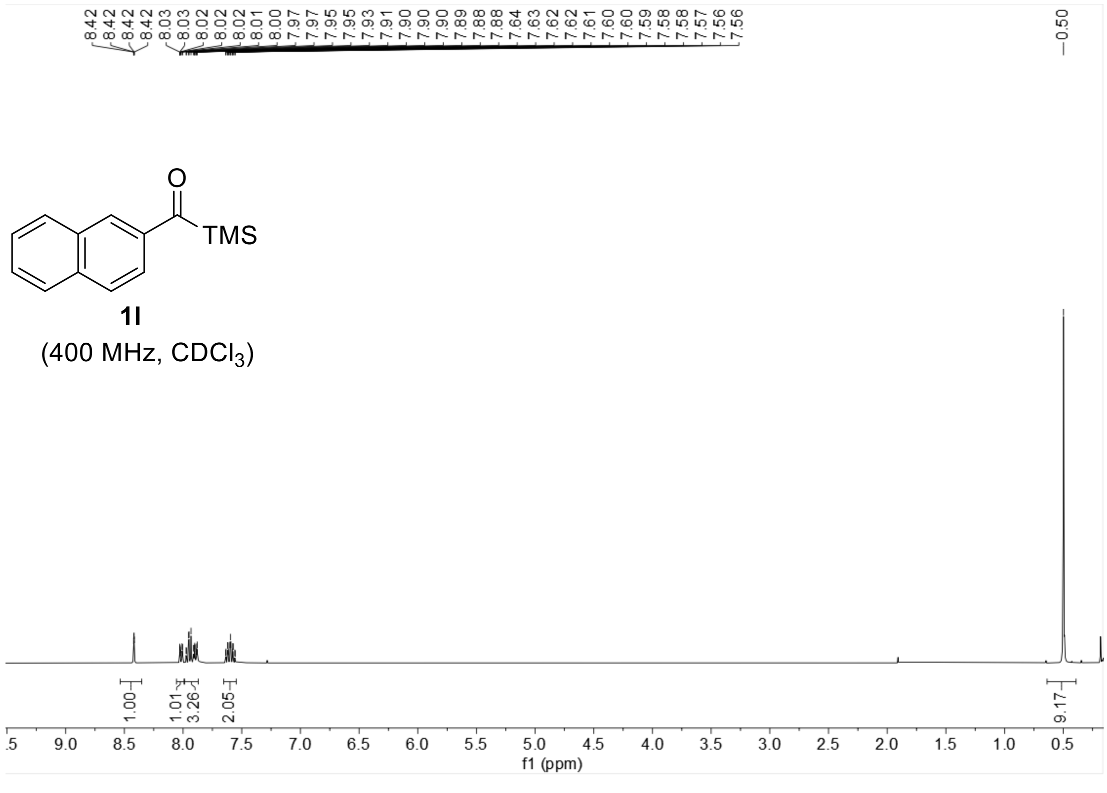

1l

(400 MHz, $CDCl_3$)

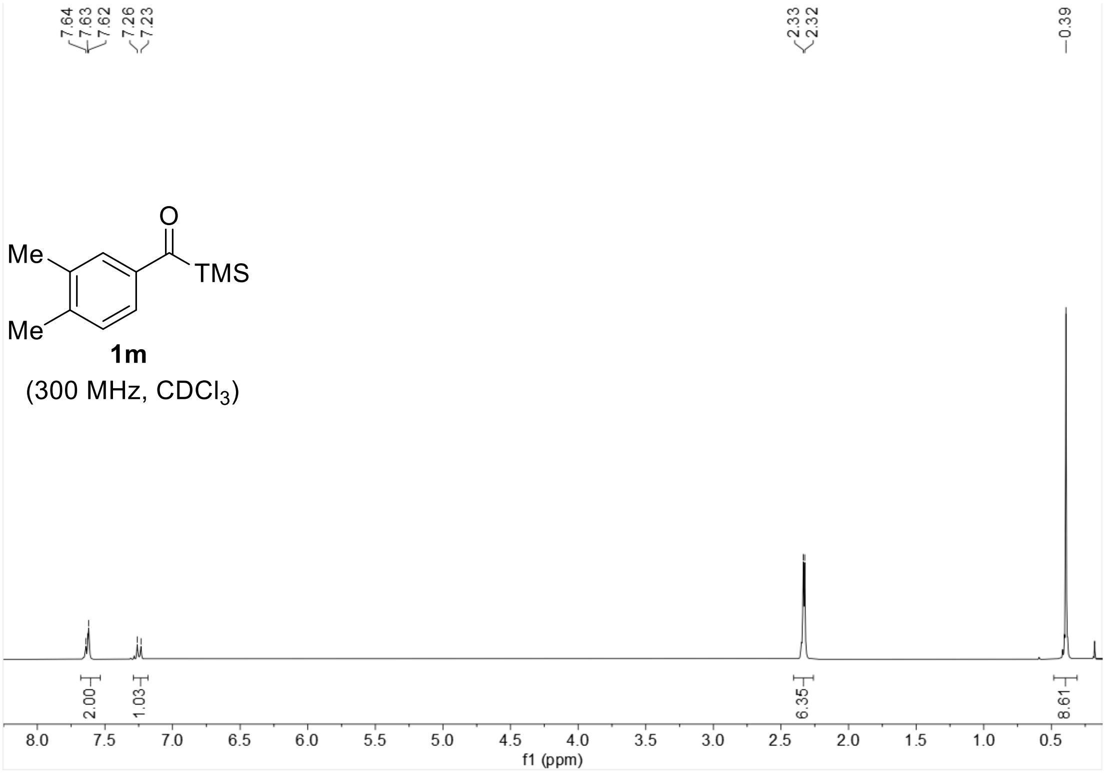
7.64
7.63
7.62
7.26
7.23
2.33
2.32
0.39
O
Me
TMS
Me
1m
(300 MHz, CDCl3)
2.00
1.03
6.35
8.61
8.0 7.5 7.0 6.5 6.0 5.5 5.0 4.5 4.0 3.5 3.0 2.5 2.0 1.5 1.0 0.5
f1 (ppm)

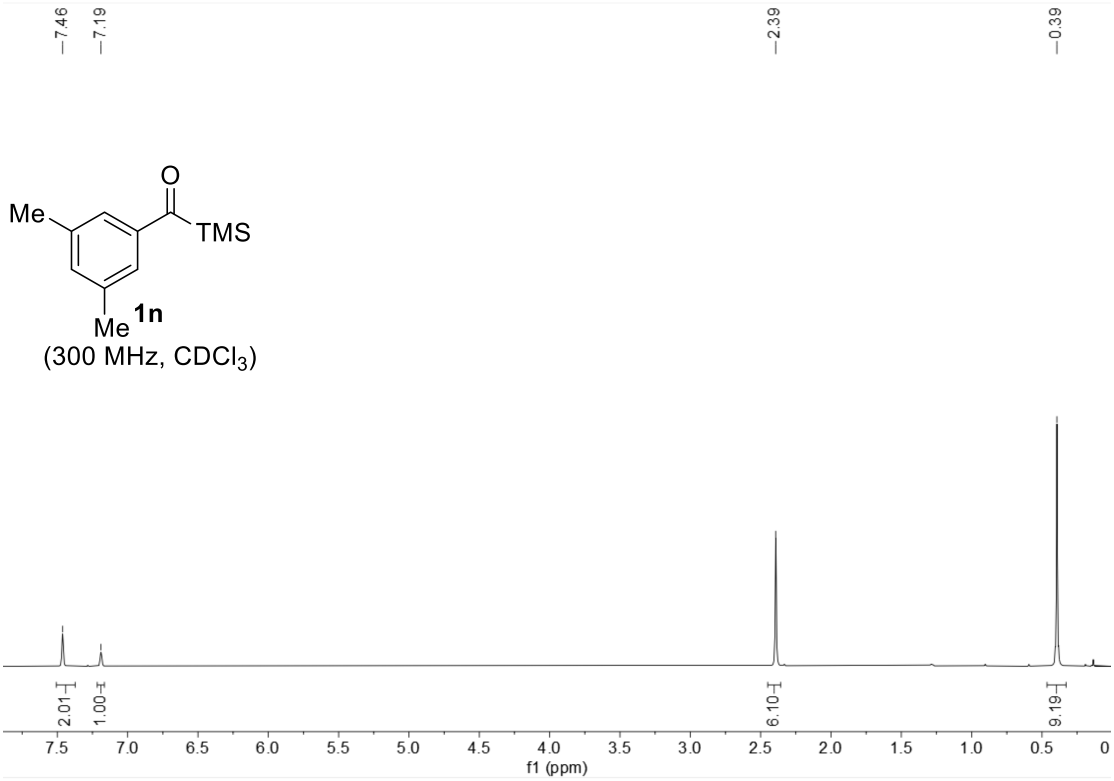
7.46
7.19
2.39
0.39
O
Me
TMS
Me
1n
(300 MHz, CDCl3)
2.01
1.00
6.10
9.19
7.5 7.0 6.5 6.0 5.5 5.0 4.5 4.0 3.5 3.0 2.5 2.0 1.5 1.0 0.5 0
f1 (ppm)

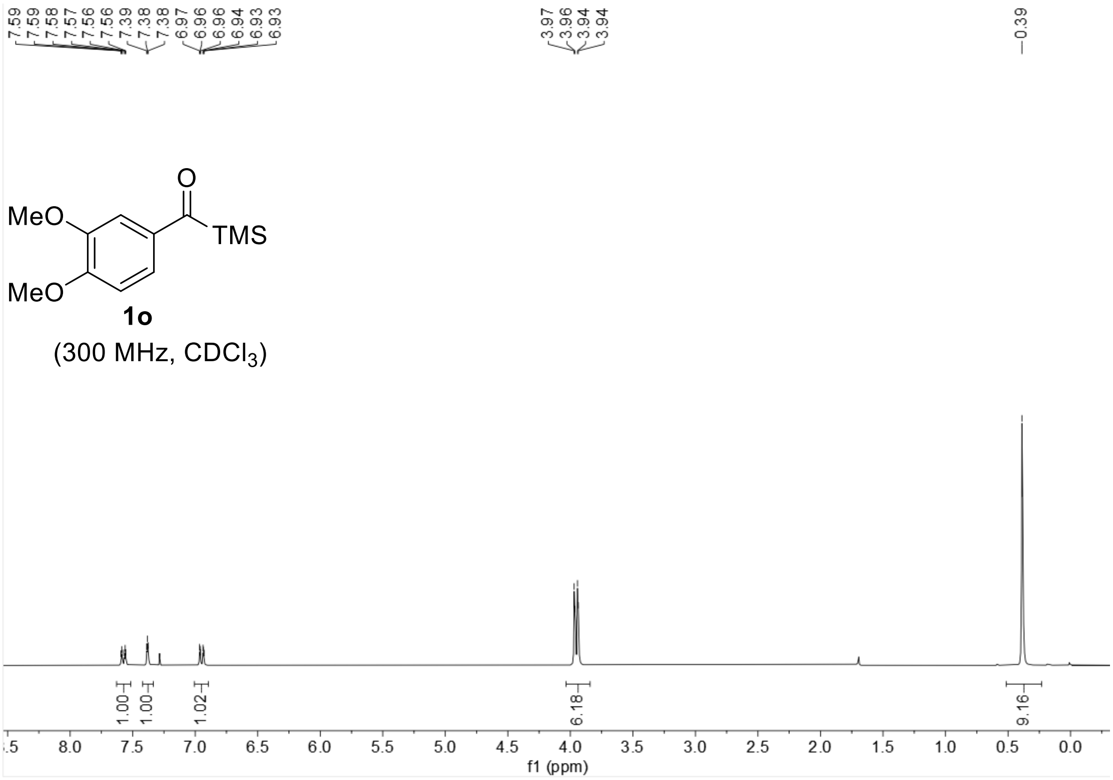

1o
(300 MHz, $CDCl_3$)

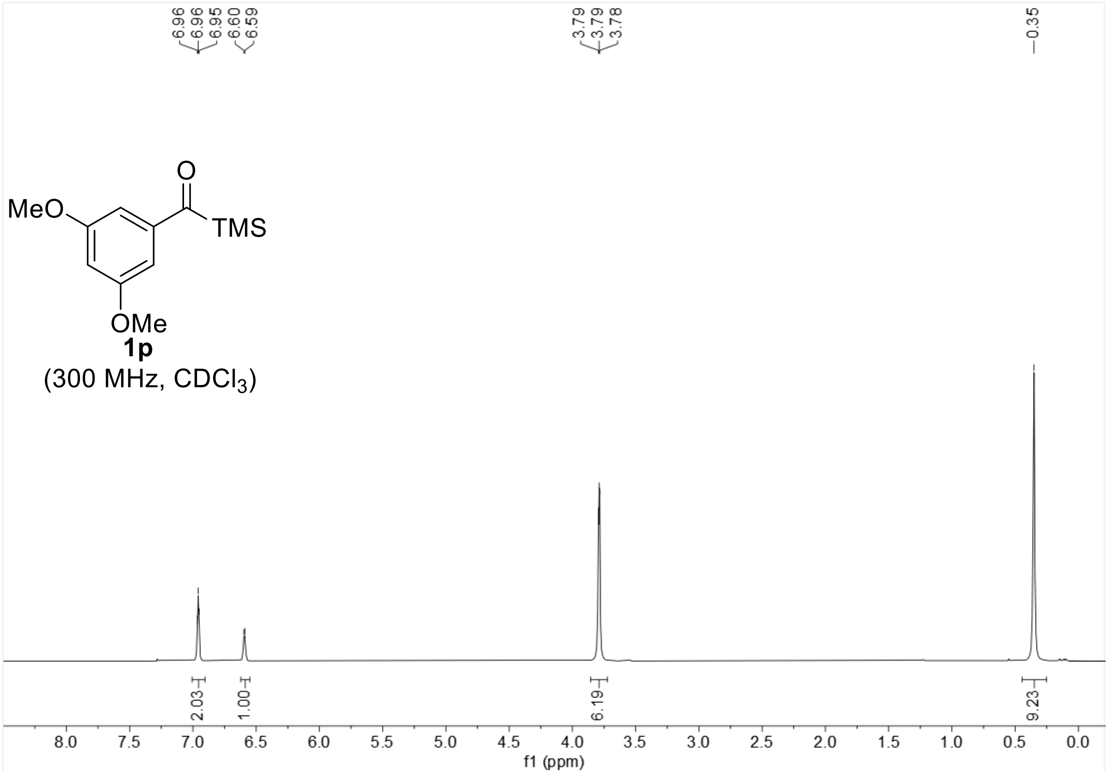

1p
(300 MHz, $CDCl_3$)

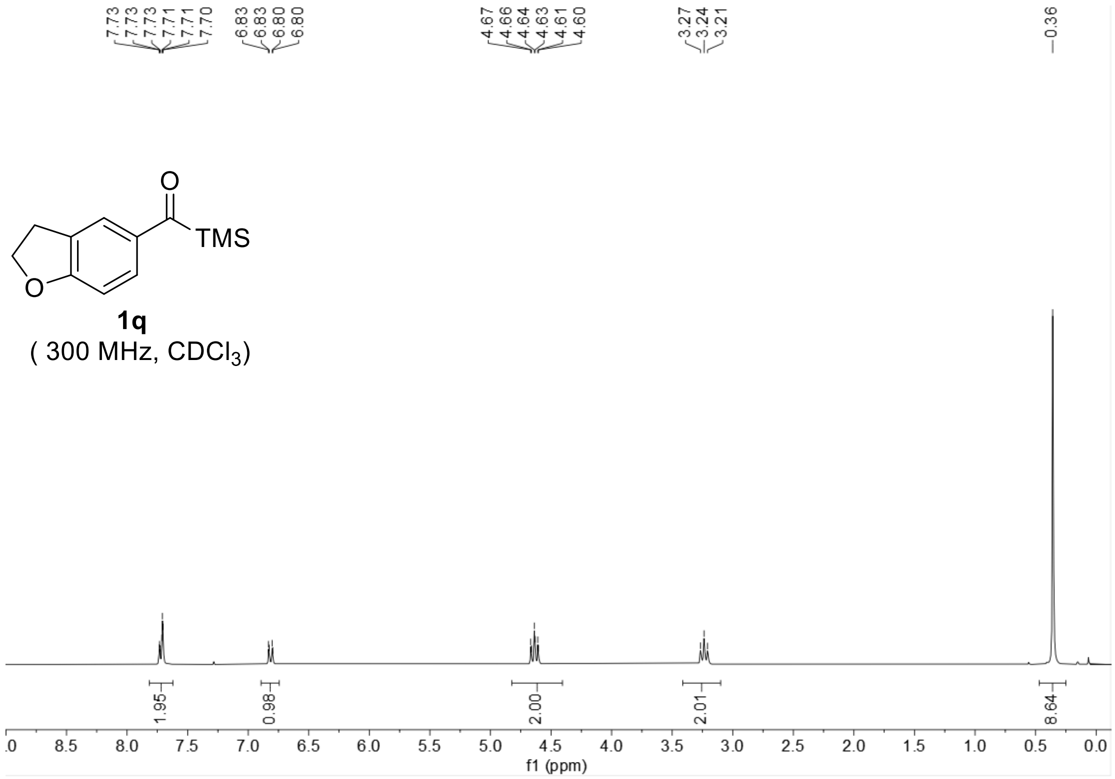
7.73
7.73
7.73
7.71
7.71
7.70
6.83
6.83
6.80
6.80
4.67
4.66
4.64
4.63
4.61
4.60
3.27
3.24
3.21
0.36
O
TMS
O
1q
(300 MHz, $CDCl_3$)
1.95
0.98
2.00
2.01
8.64
f1 (ppm)

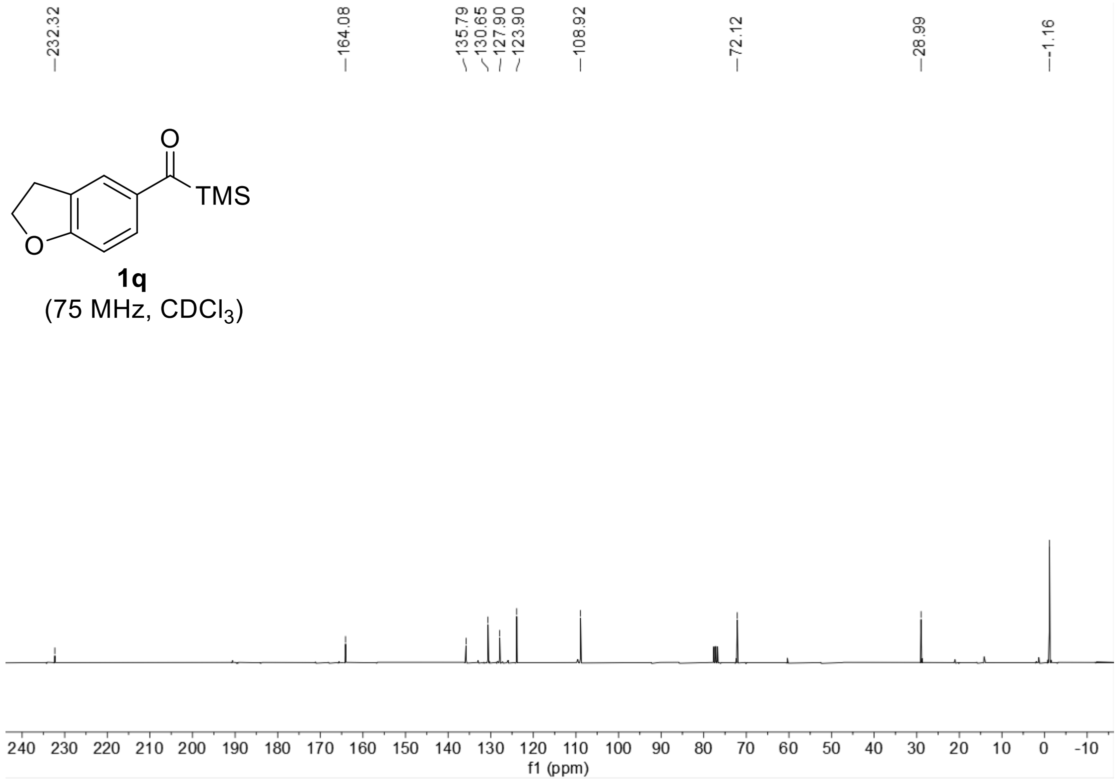
232.32
164.08
135.79
130.65
127.90
123.90
108.92
72.12
28.99
-1.16
O
TMS
O
1q
(75 MHz, $CDCl_3$)
f1 (ppm)

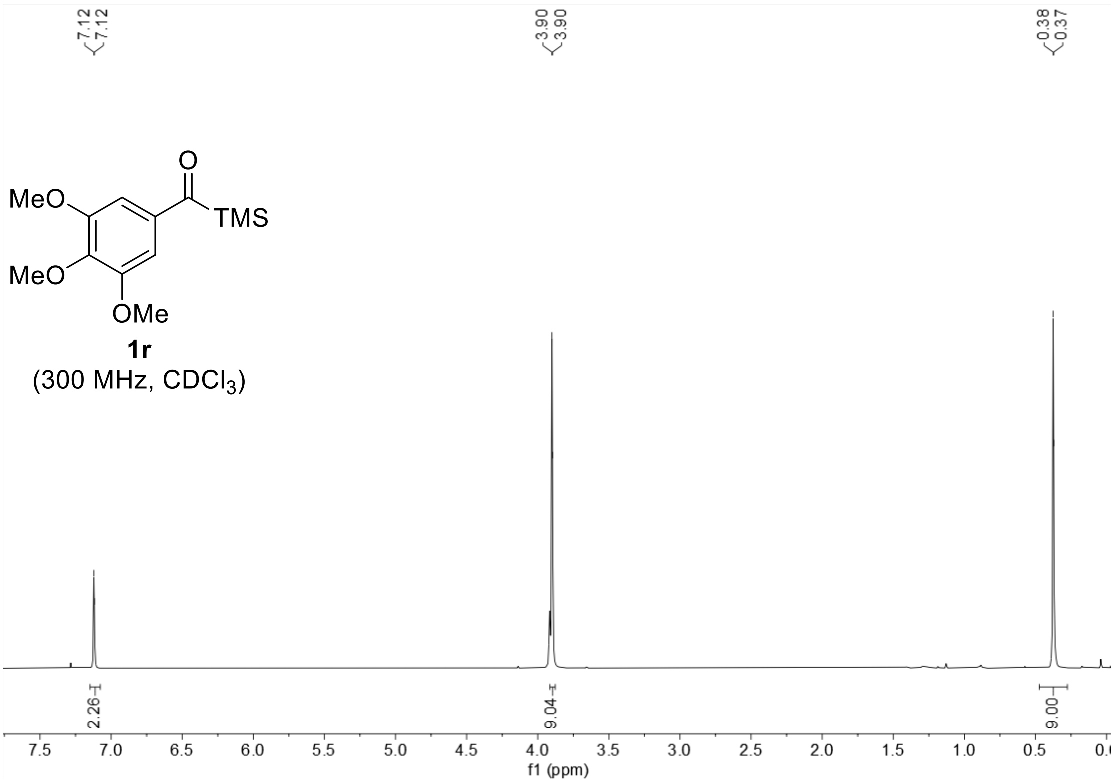

1r
(300 MHz, $CDCl_3$)

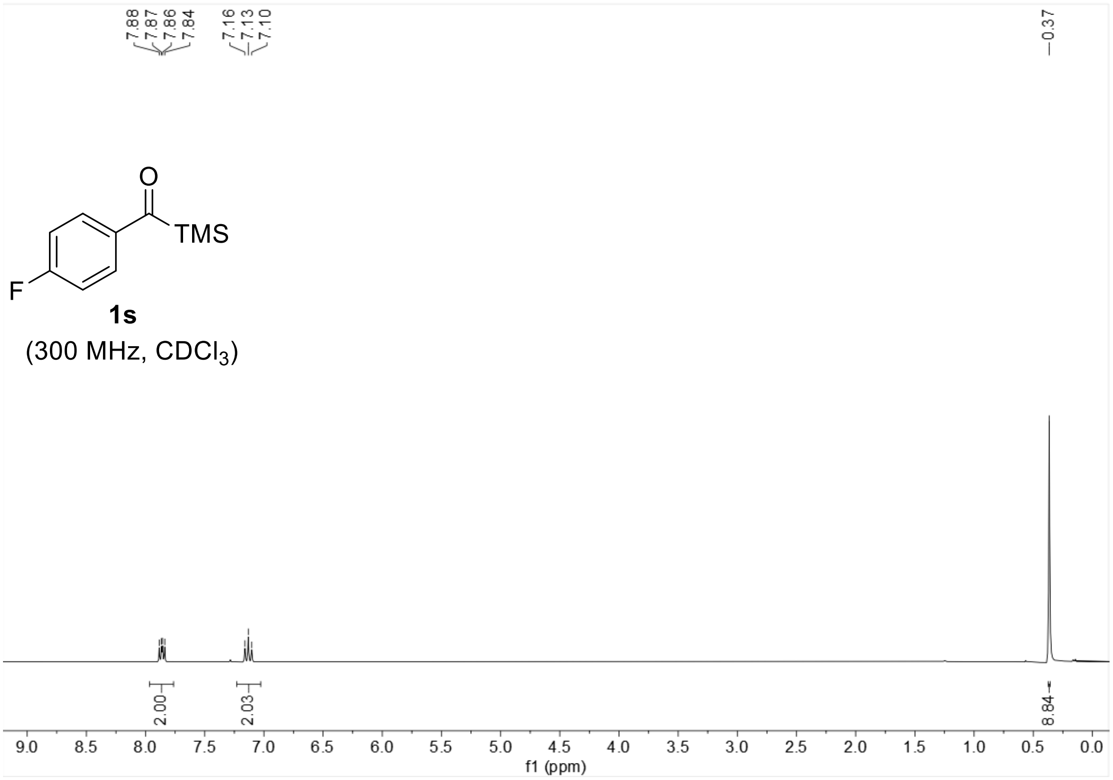

1s
(300 MHz, $CDCl_3$)

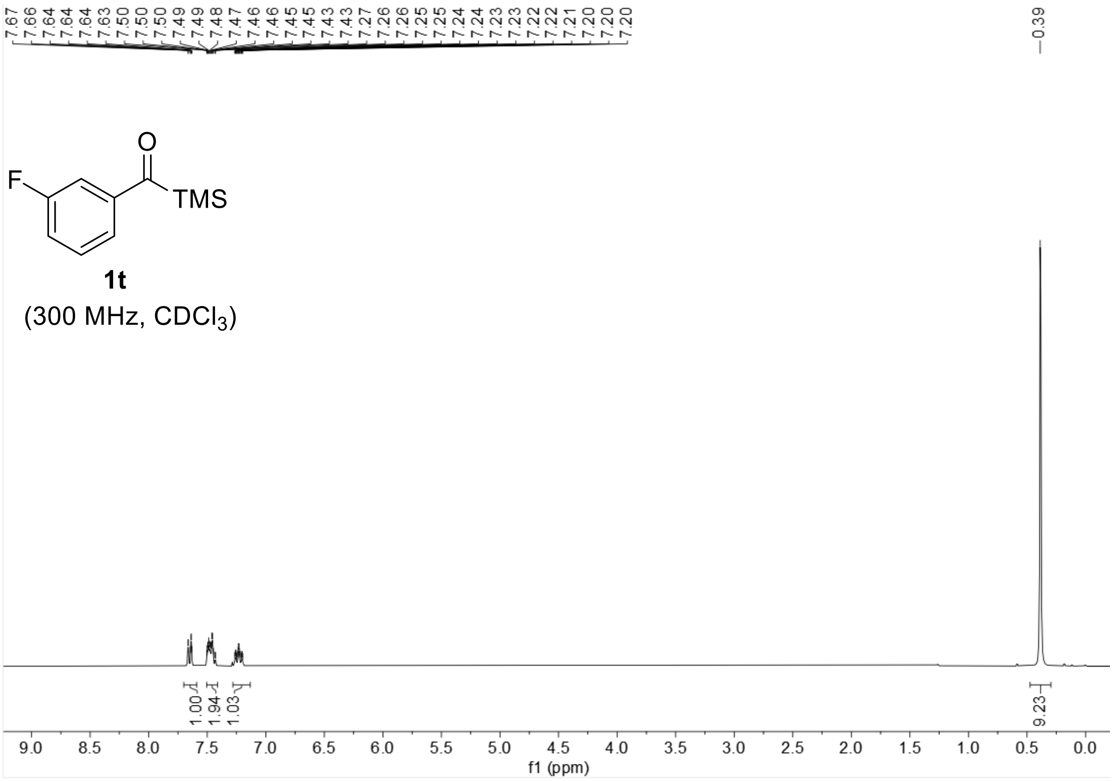

7.67 7.66 7.64 7.64 7.64 7.63 7.50 7.50 7.50 7.49 7.49 7.48 7.47 7.46 7.46 7.45 7.45 7.43 7.43 7.27 7.26 7.26 7.25 7.24 7.24 7.23 7.23 7.22 7.22 7.21 7.20 7.20 7.20
0.39
F
O
TMS
1t
(300 MHz, CDCl3)
1.00
1.94
1.03
9.23
f1 (ppm)

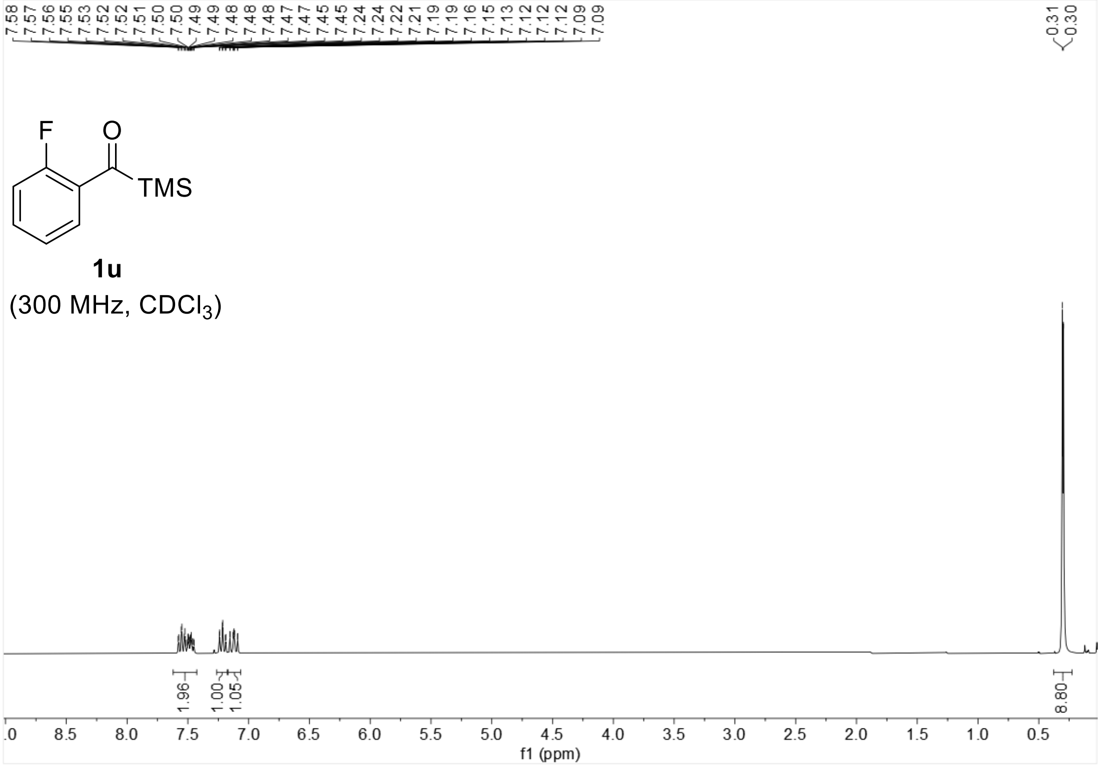

7.58 7.57 7.56 7.55 7.53 7.52 7.52 7.51 7.50 7.50 7.49 7.49 7.48 7.48 7.48 7.47 7.45 7.45 7.24 7.24 7.22 7.21 7.19 7.16 7.15 7.13 7.12 7.12 7.12 7.09
0.31
0.30
F
O
TMS
1u
(300 MHz, CDCl3)
1.96
1.00
1.05
8.80
f1 (ppm)

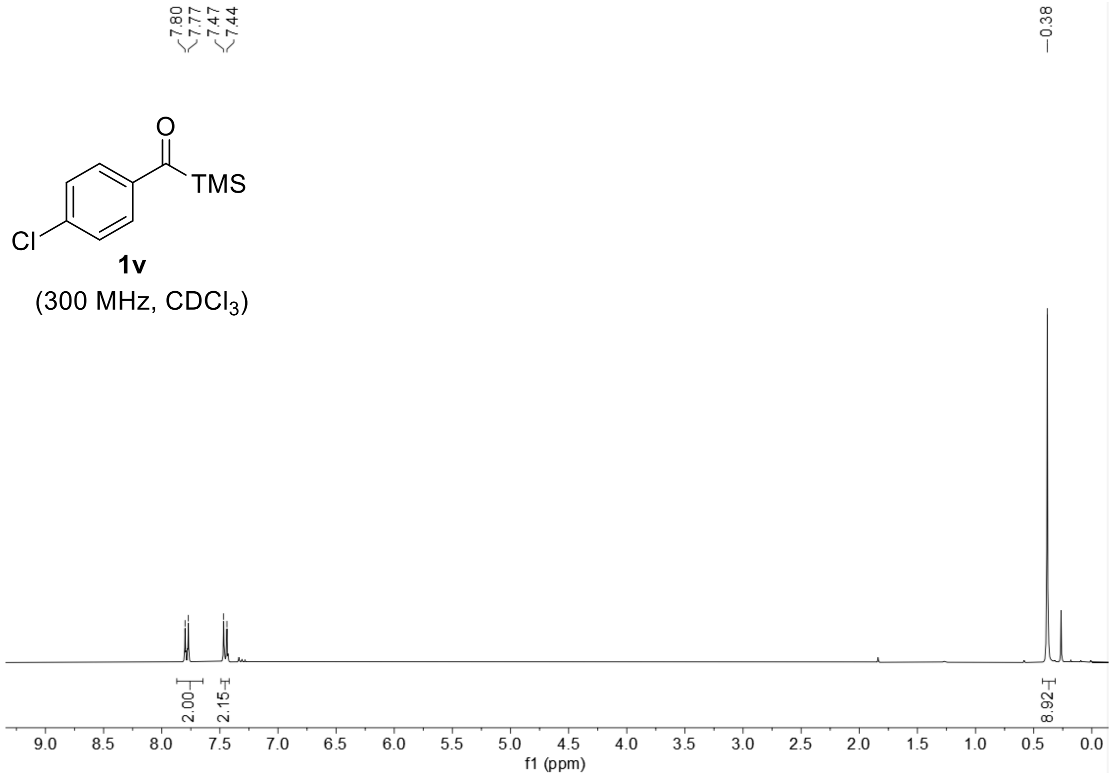

1v

(300 MHz, $CDCl_3$)

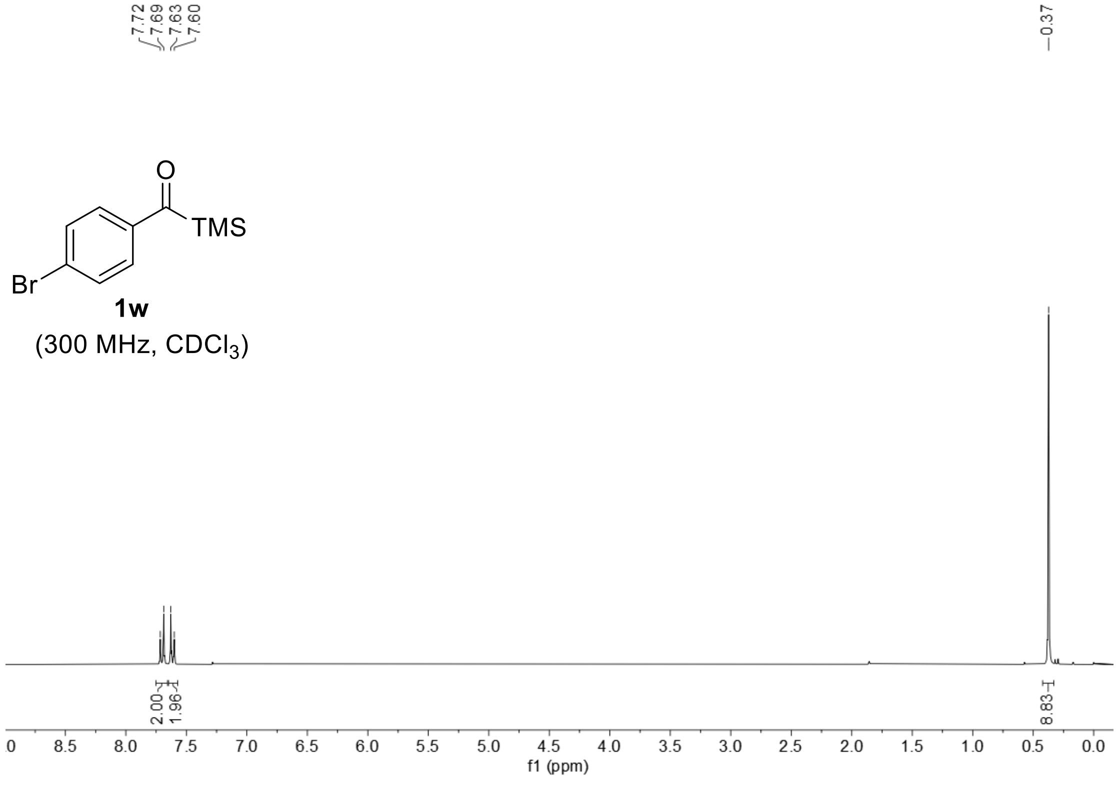

1w

(300 MHz, $CDCl_3$)

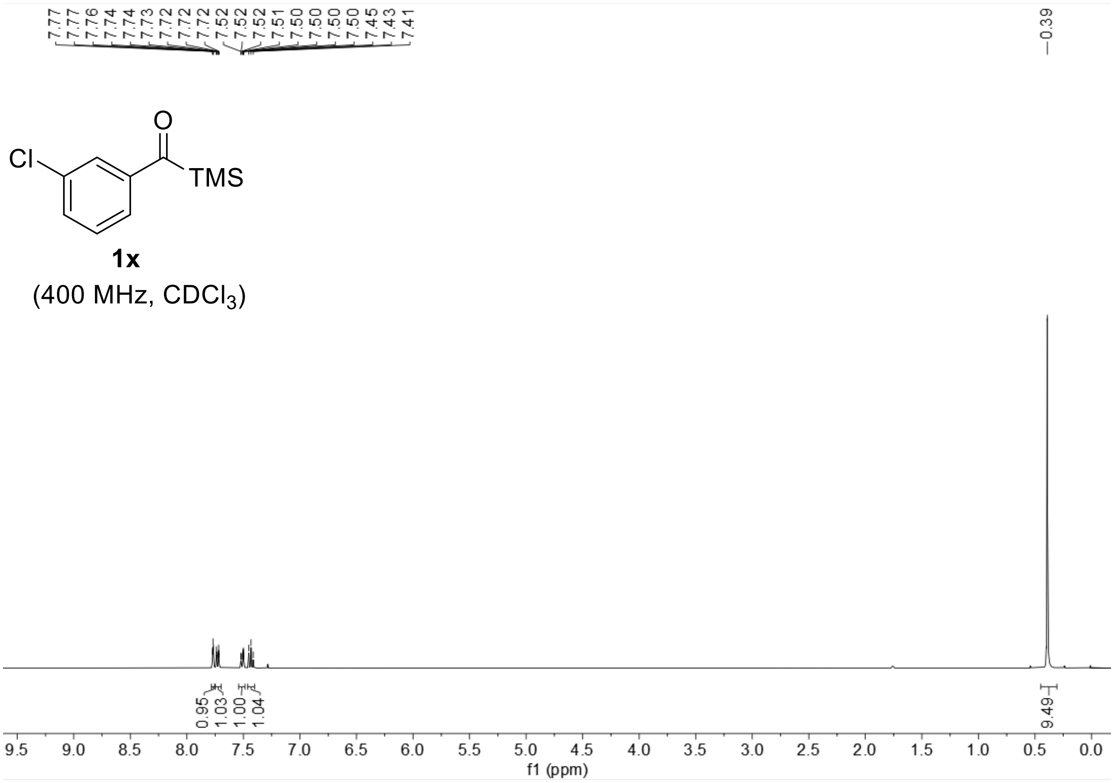
7.77
7.77
7.76
7.74
7.74
7.73
7.72
7.72
7.72
7.72
7.52
7.52
7.52
7.51
7.50
7.50
7.50
7.50
7.45
7.43
7.41
0.39
Cl
O
TMS
1x
(400 MHz, CDCl3)
0.95
1.03
1.00
1.04
9.49
9.5 9.0 8.5 8.0 7.5 7.0 6.5 6.0 5.5 5.0 4.5 4.0 3.5 3.0 2.5 2.0 1.5 1.0 0.5 0.0
f1 (ppm)

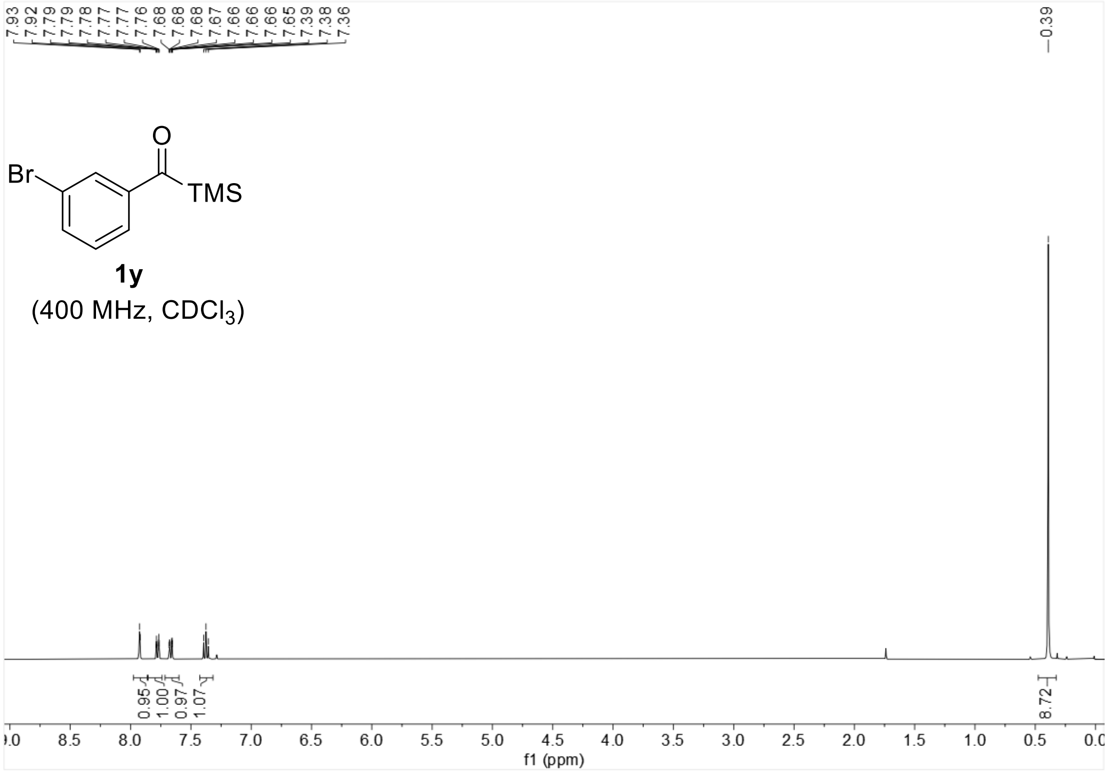
7.93
7.92
7.79
7.79
7.78
7.77
7.77
7.76
7.68
7.68
7.68
7.67
7.66
7.66
7.66
7.65
7.39
7.38
7.36
0.39
Br
O
TMS
1y
(400 MHz, CDCl3)
0.95
1.00
0.97
1.07
8.72
8.5 8.0 7.5 7.0 6.5 6.0 5.5 5.0 4.5 4.0 3.5 3.0 2.5 2.0 1.5 1.0 0.5
f1 (ppm)

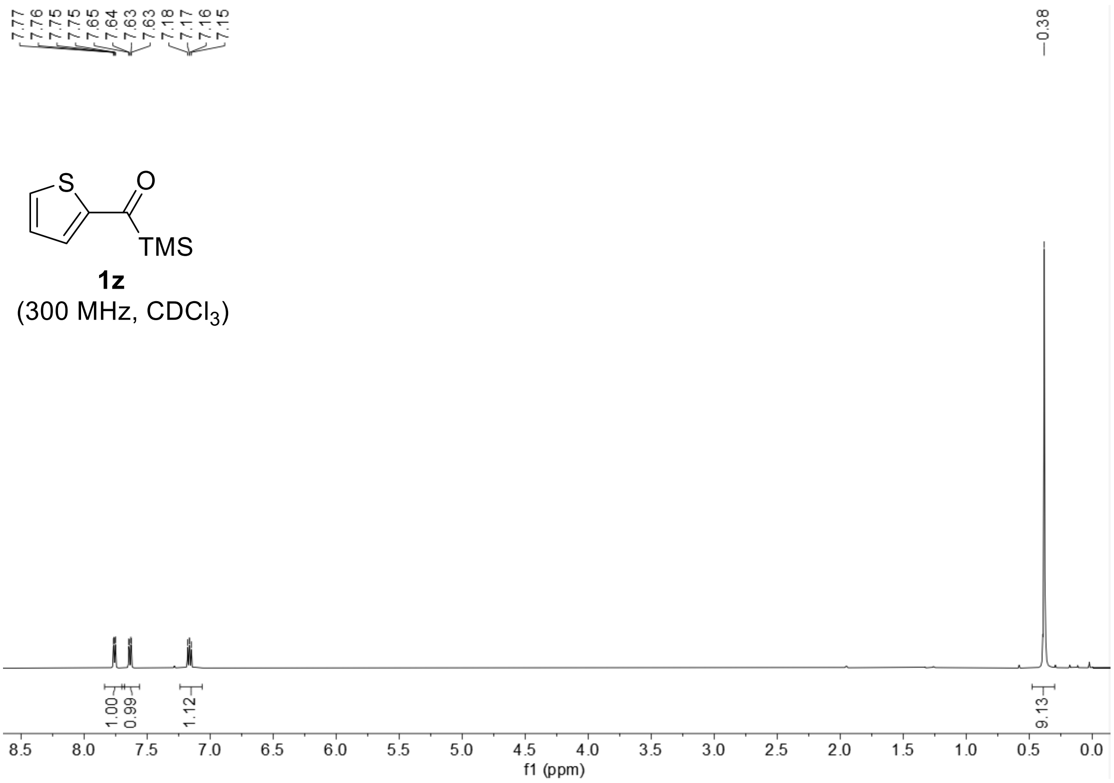

1z

(300 MHz, $CDCl_3$)

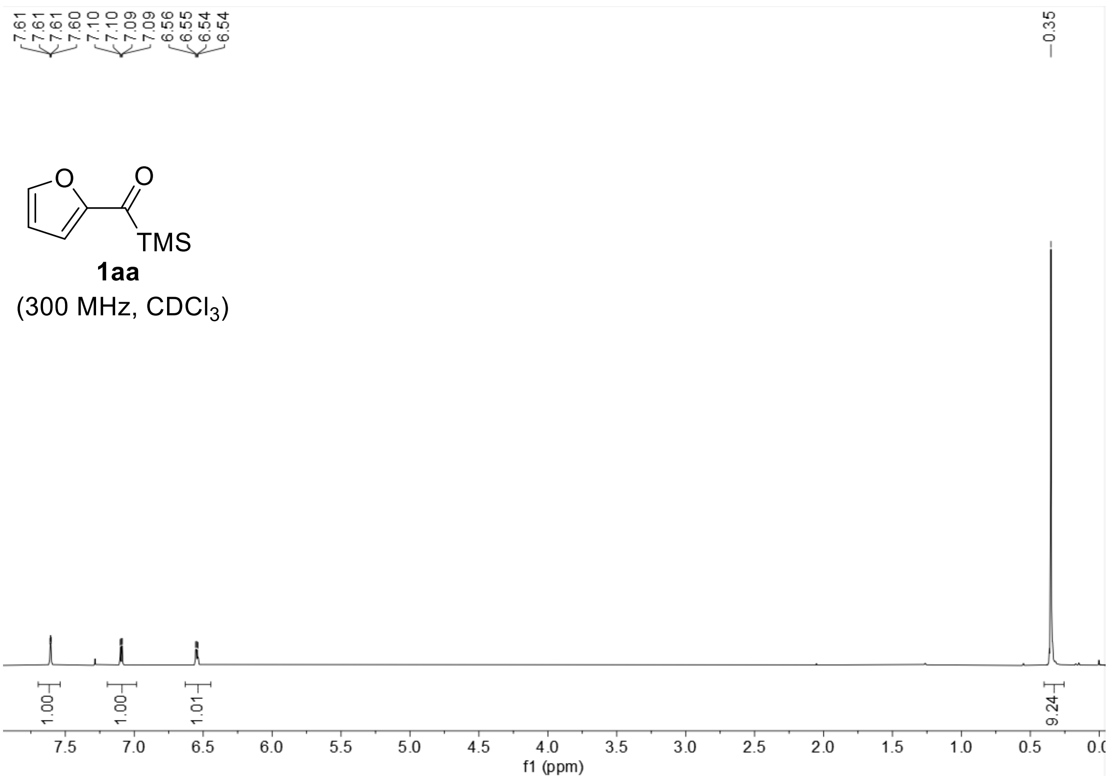

1aa

(300 MHz, $CDCl_3$)

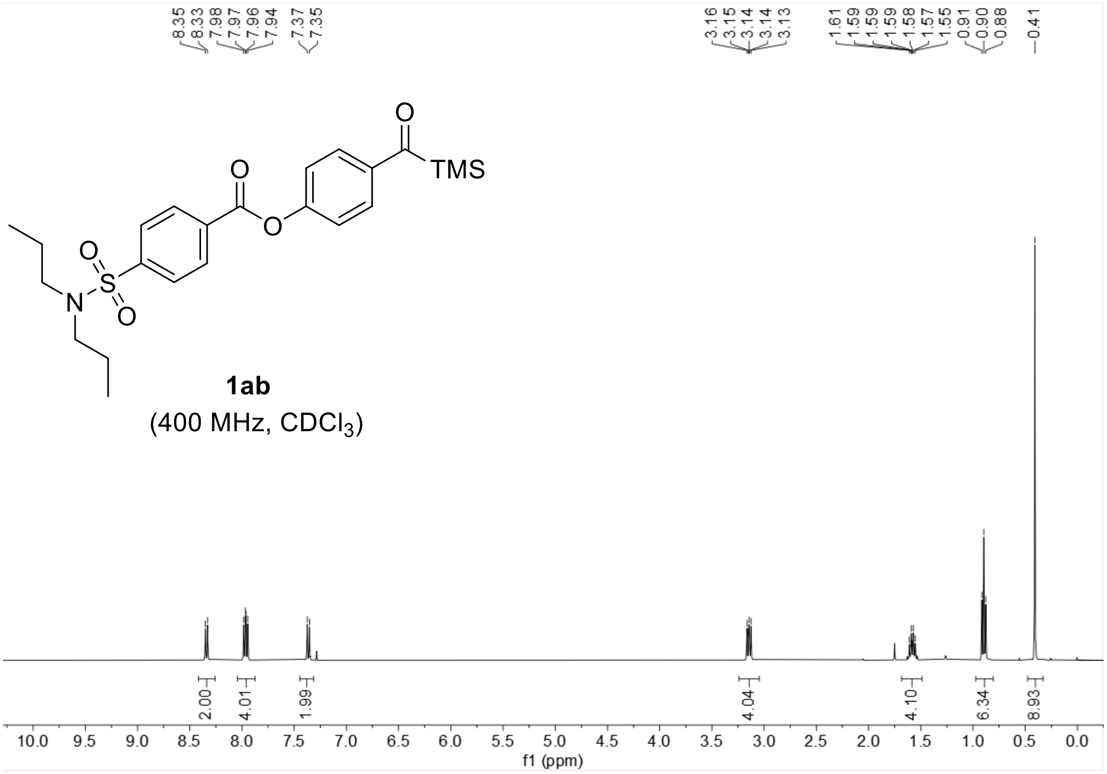

8.35 8.33 7.98 7.97 7.96 7.94 7.37 7.35
3.16 3.15 3.14 3.14 3.13
1.61 1.59 1.59 1.59 1.58 1.57 1.55 0.91 0.90 0.88
0.41
O
TMS
O
O
O
S
N
O
1ab
(400 MHz, CDCl3)
2.00 4.01 1.99 4.04 4.10 6.34 8.93
f1 (ppm)

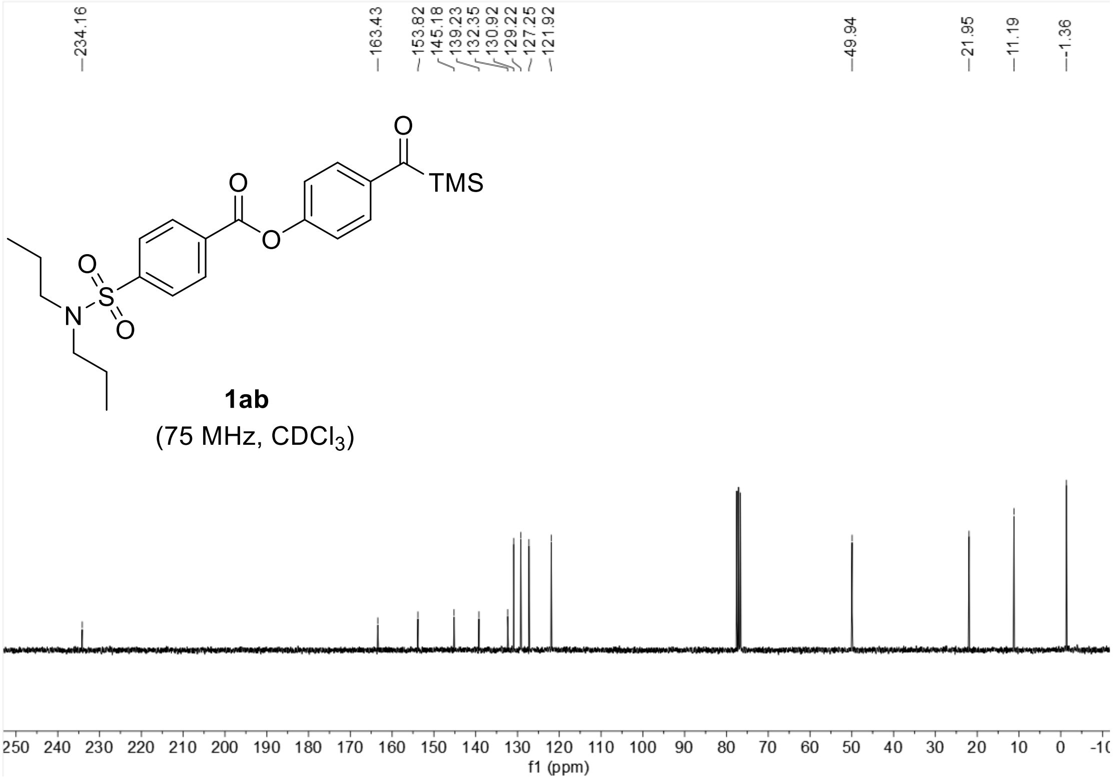

234.16
163.43 153.82 145.18 139.23 132.35 130.92 129.22 127.25 121.92
49.94
21.95 11.19 -1.36
O
TMS
O
O
O
S
N
O
1ab
(75 MHz, CDCl3)
f1 (ppm)

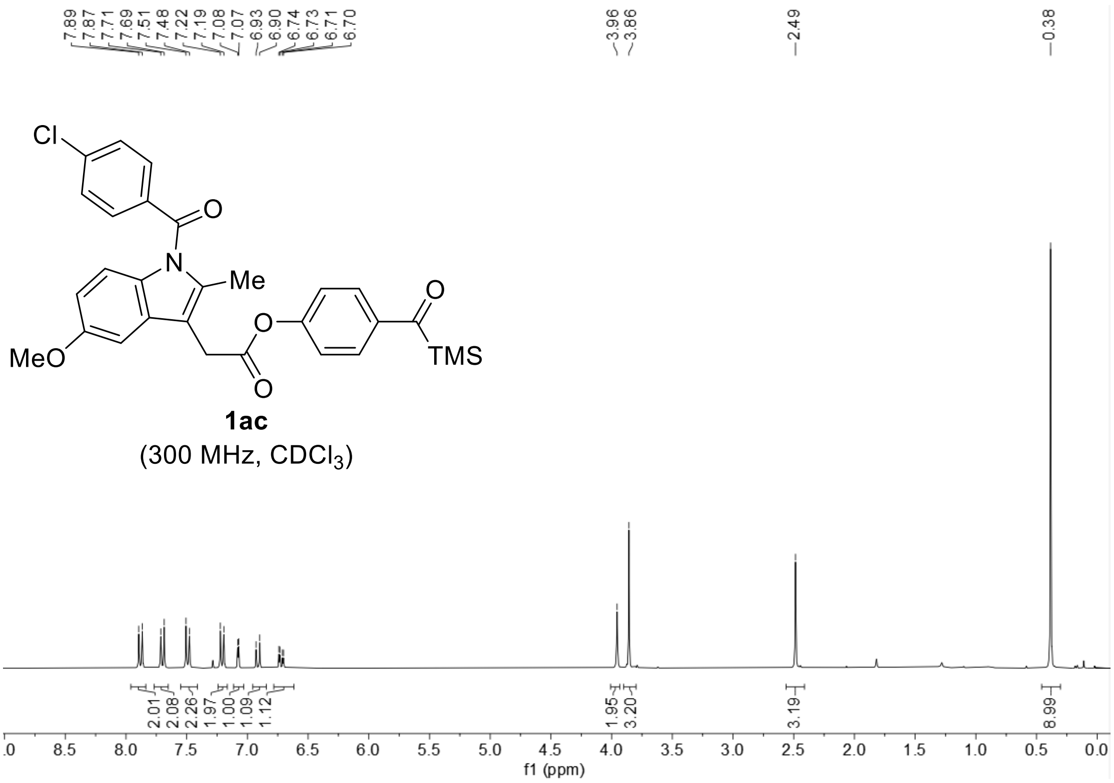
7.89
7.87
7.71
7.69
7.51
7.48
7.22
7.19
7.08
7.07
6.93
6.90
6.74
6.73
6.71
6.70
3.96
3.86
2.49
0.38
Cl
O
N
Me
O
O
MeO
TMS
O
1ac
(300 MHz, CDCl3)
2.01
2.08
2.26
1.97
1.00
1.09
1.12
1.95
3.20
3.19
8.99
f1 (ppm)

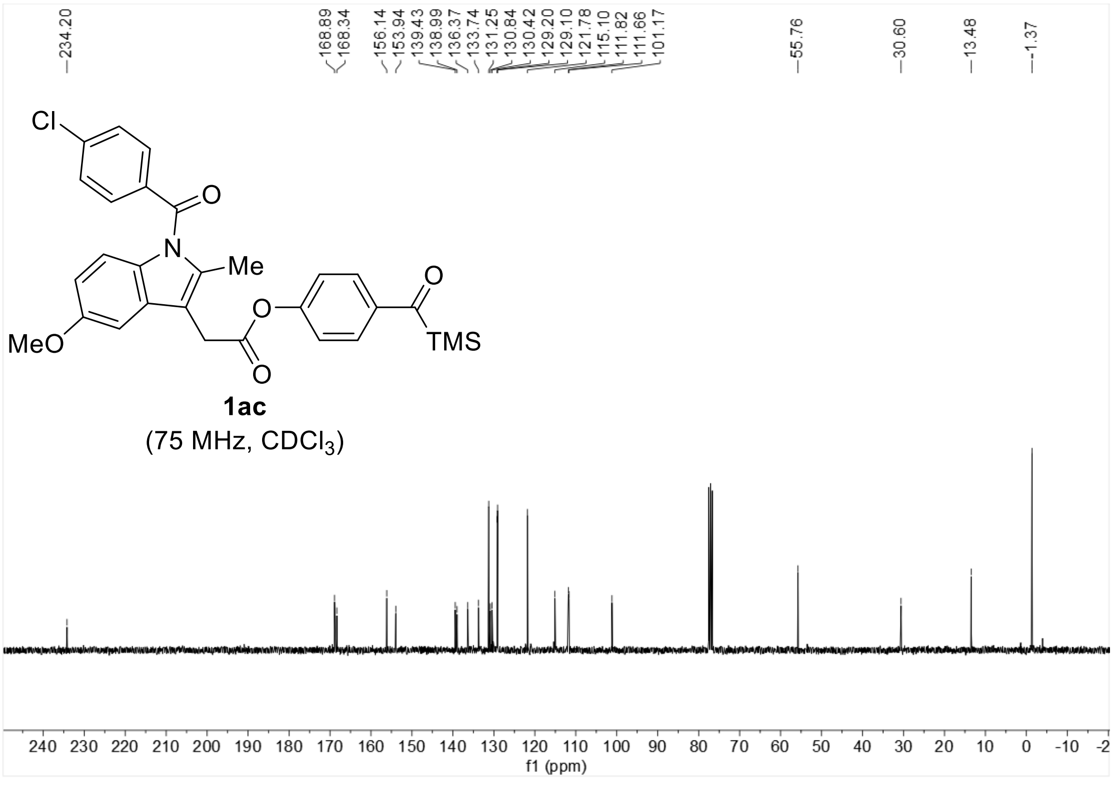
234.20
168.89
168.34
156.14
153.94
139.43
138.99
136.37
133.74
131.25
130.84
130.42
129.20
129.10
121.78
115.10
111.82
111.66
101.17
55.76
30.60
13.48
-1.37
Cl
O
N
Me
O
O
MeO
TMS
O
1ac
(75 MHz, CDCl3)
f1 (ppm)

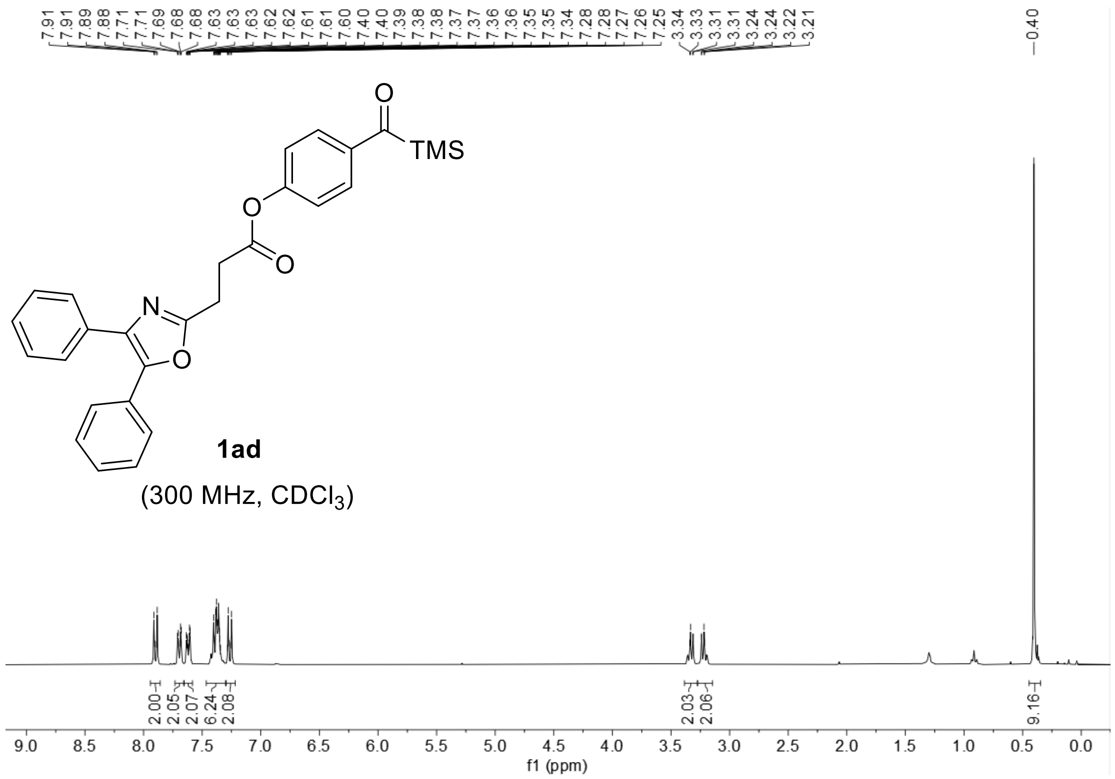
7.91 7.91 7.89 7.88 7.71 7.71 7.69 7.68 7.68 7.63 7.63 7.62 7.62 7.61 7.61 7.60 7.40 7.40 7.39 7.38 7.38 7.37 7.37 7.36 7.36 7.35 7.35 7.34 7.28 7.28 7.27 7.26 7.25 3.34 3.33 3.31 3.31 3.24 3.24 3.22 3.21
0.40
TMS
O
N
1ad
(300 MHz, CDCl3)
2.00 2.05 2.07 6.24 2.08
2.03 2.06
9.16
9.0 8.5 8.0 7.5 7.0 6.5 6.0 5.5 5.0 4.5 4.0 3.5 3.0 2.5 2.0 1.5 1.0 0.5 0.0
f1 (ppm)

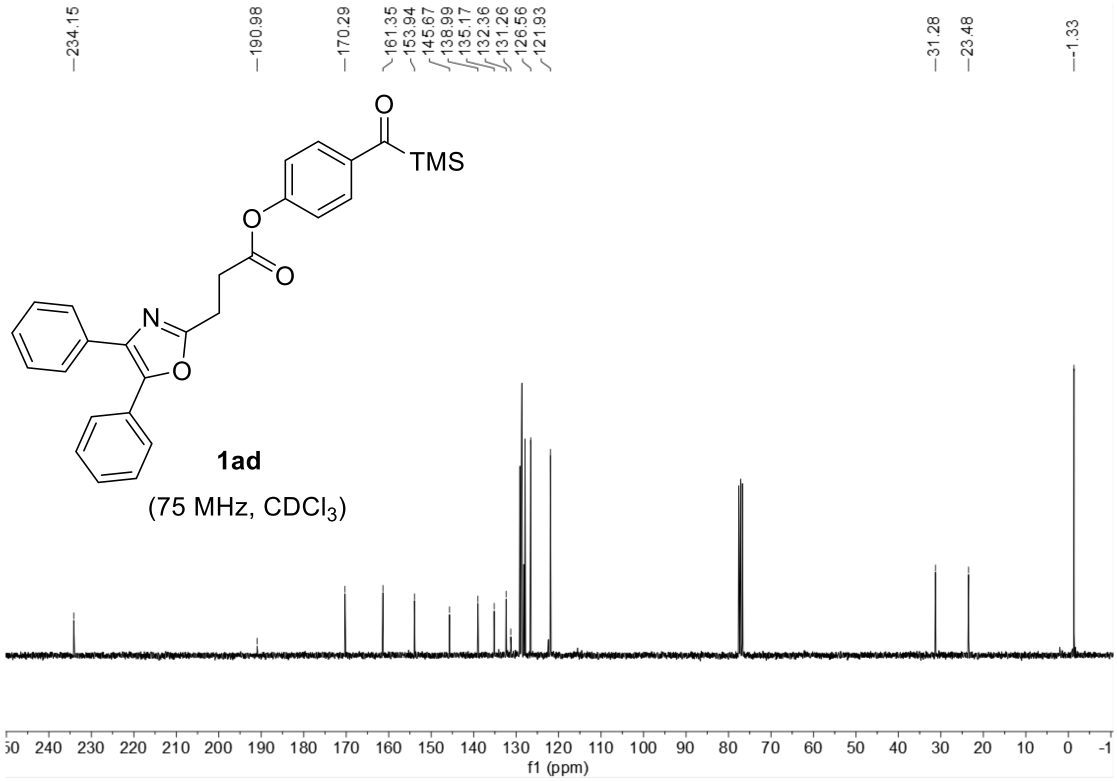
234.15
190.98
170.29
161.35 153.94 145.67 138.99 135.17 132.36 131.26 126.56 121.93
31.28
23.48
1.33
TMS
O
N
1ad
(75 MHz, CDCl3)
240 230 220 210 200 190 180 170 160 150 140 130 120 110 100 90 80 70 60 50 40 30 20 10 0
f1 (ppm)

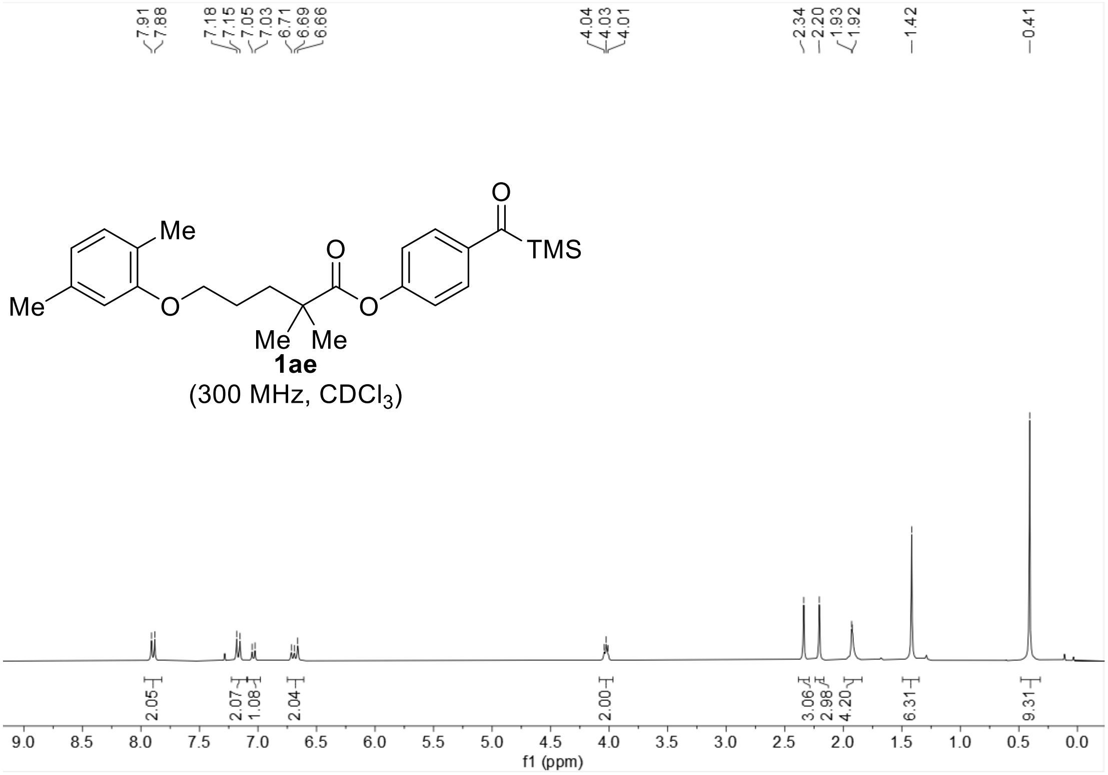
7.91
7.88
7.18
7.15
7.05
7.03
6.71
6.69
6.66
4.04
4.03
4.01
2.34
2.20
1.93
1.92
1.42
0.41
Me
O
TMS
Me
O
O
Me
Me
1ae
(300 MHz, CDCl3)
2.05
2.07
1.08
2.04
2.00
3.06
2.98
4.20
6.31
9.31
9.0 8.5 8.0 7.5 7.0 6.5 6.0 5.5 5.0 4.5 4.0 3.5 3.0 2.5 2.0 1.5 1.0 0.5 0.0
f1 (ppm)

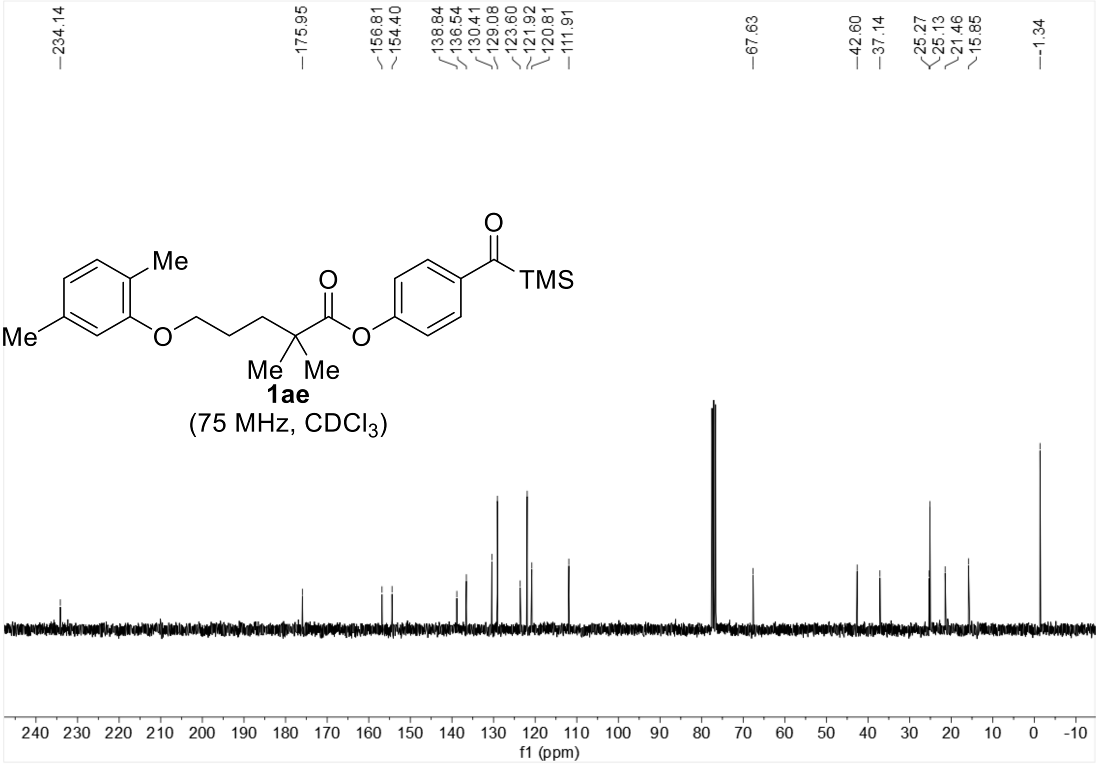
234.14
175.95
156.81
154.40
138.84
136.54
130.41
129.08
123.60
121.92
120.81
111.91
67.63
42.60
37.14
25.27
25.13
21.46
15.85
-1.34
Me
O
TMS
Me
O
O
Me
Me
1ae
(75 MHz, CDCl3)
240 230 220 210 200 190 180 170 160 150 140 130 120 110 100 90 80 70 60 50 40 30 20 10 0 -10
f1 (ppm)

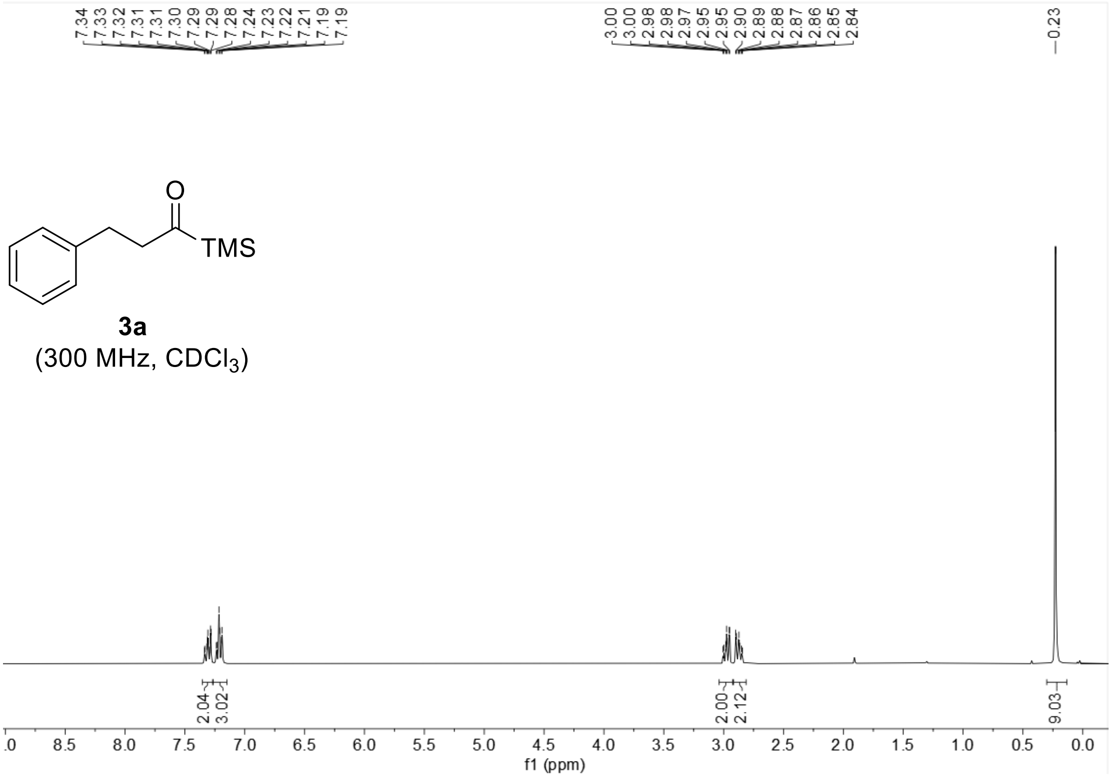

3a
(300 MHz, $CDCl_3$)

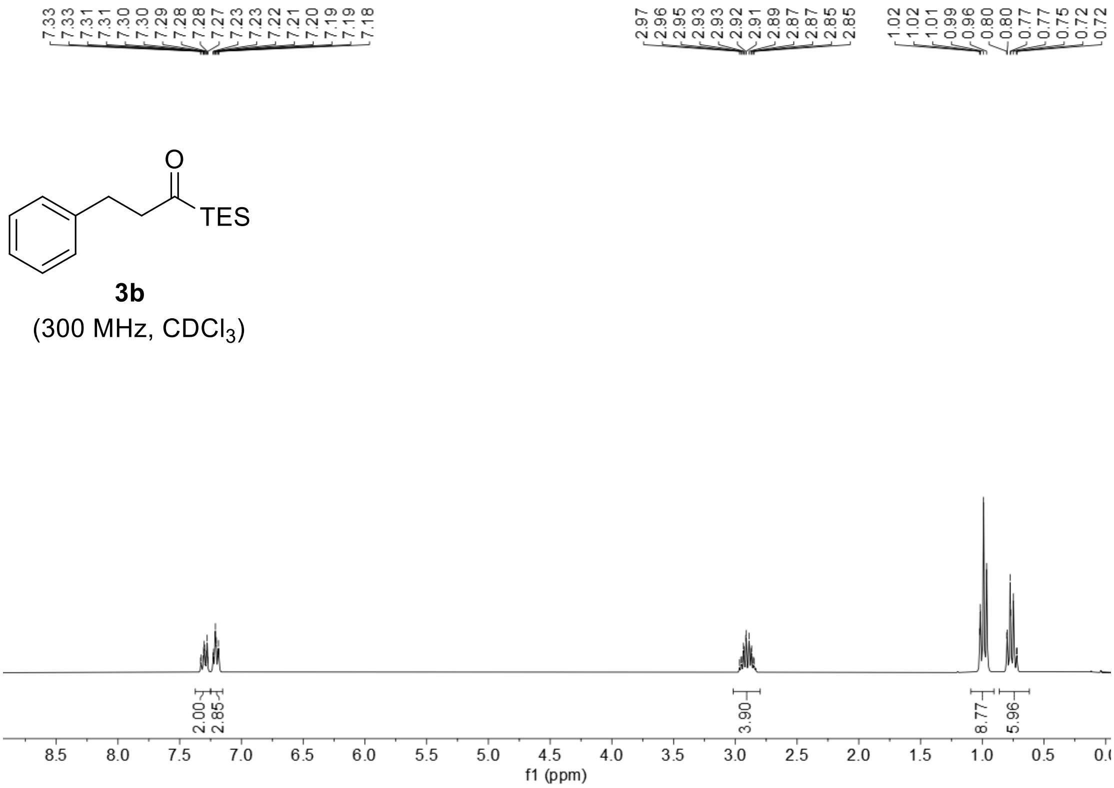

3b
(300 MHz, $CDCl_3$)

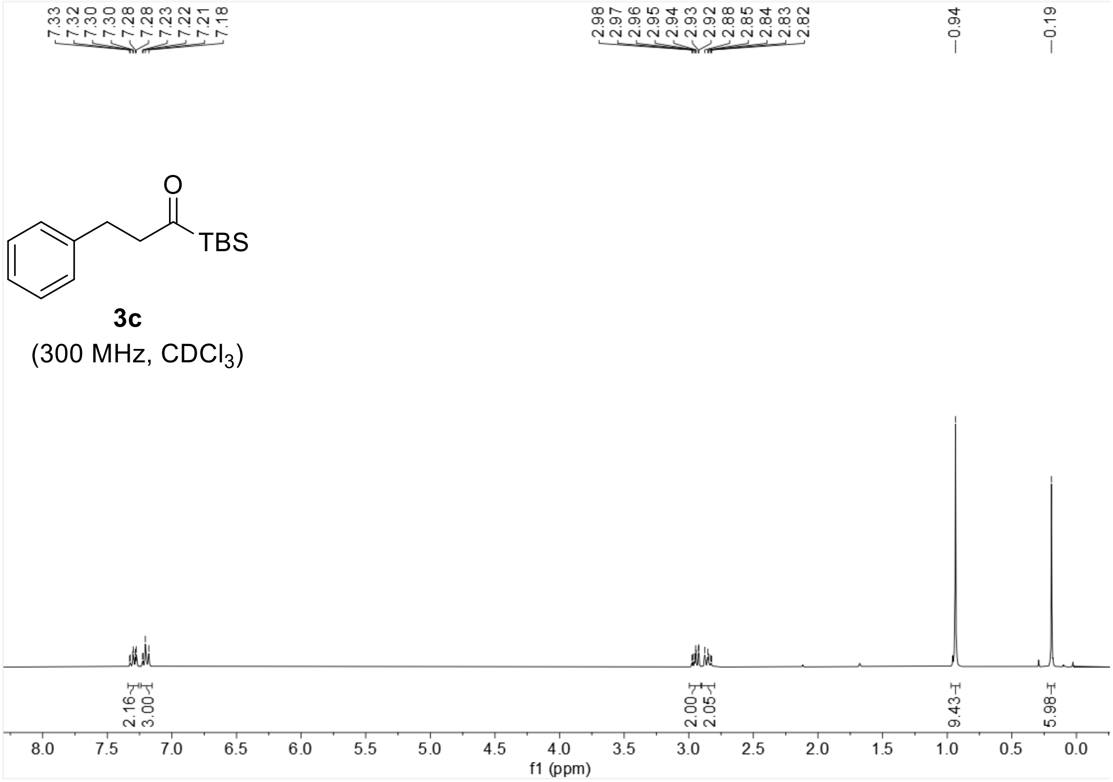

7.33
7.32
7.30
7.28
7.28
7.23
7.22
7.21
7.18
2.98
2.97
2.96
2.95
2.94
2.93
2.92
2.88
2.85
2.84
2.83
2.82
0.94
0.19
O
TBS
3c
(300 MHz, CDCl3)
2.16
3.00
2.00
2.05
9.43
5.98
8.0
7.5
7.0
6.5
6.0
5.5
5.0
4.5
4.0
3.5
3.0
2.5
2.0
1.5
1.0
0.5
0.0
f1 (ppm)

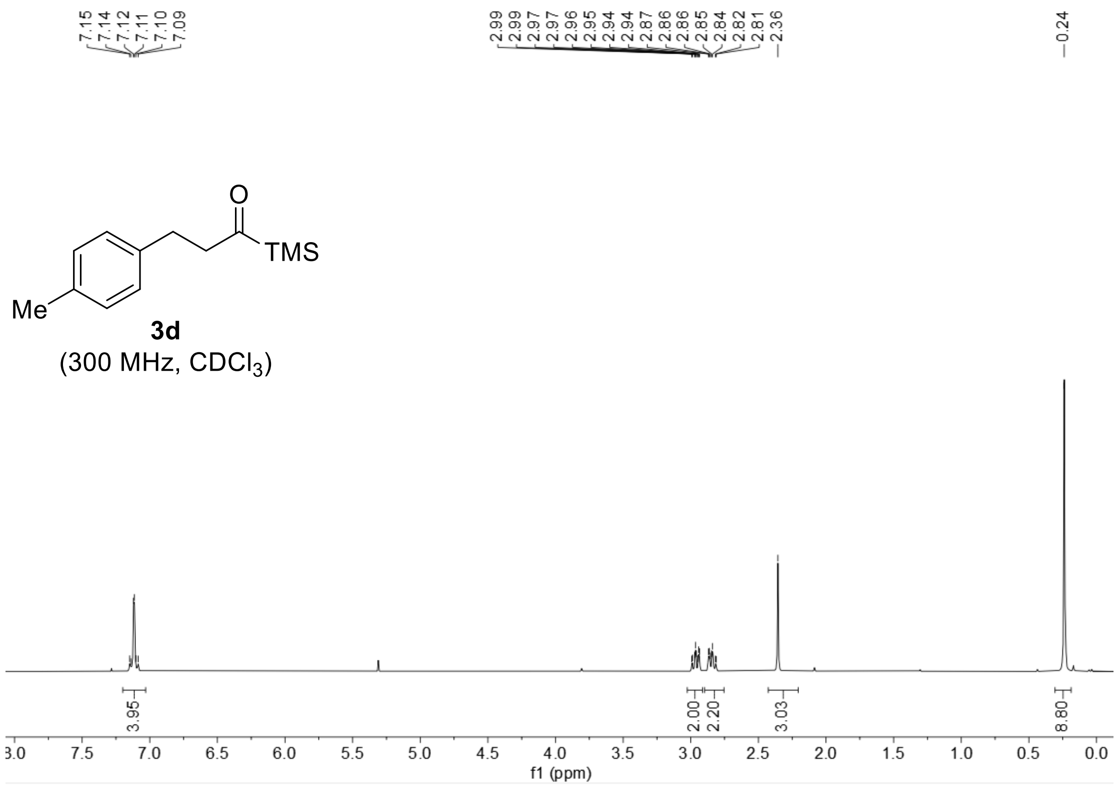
7.15
7.14
7.12
7.11
7.10
7.09
2.99
2.99
2.97
2.96
2.95
2.94
2.94
2.87
2.86
2.86
2.85
2.84
2.82
2.81
2.36
0.24
O
TMS
Me
3d
(300 MHz, $CDCl_3$)
3.95
2.00
2.20
3.03
8.80
f1 (ppm)

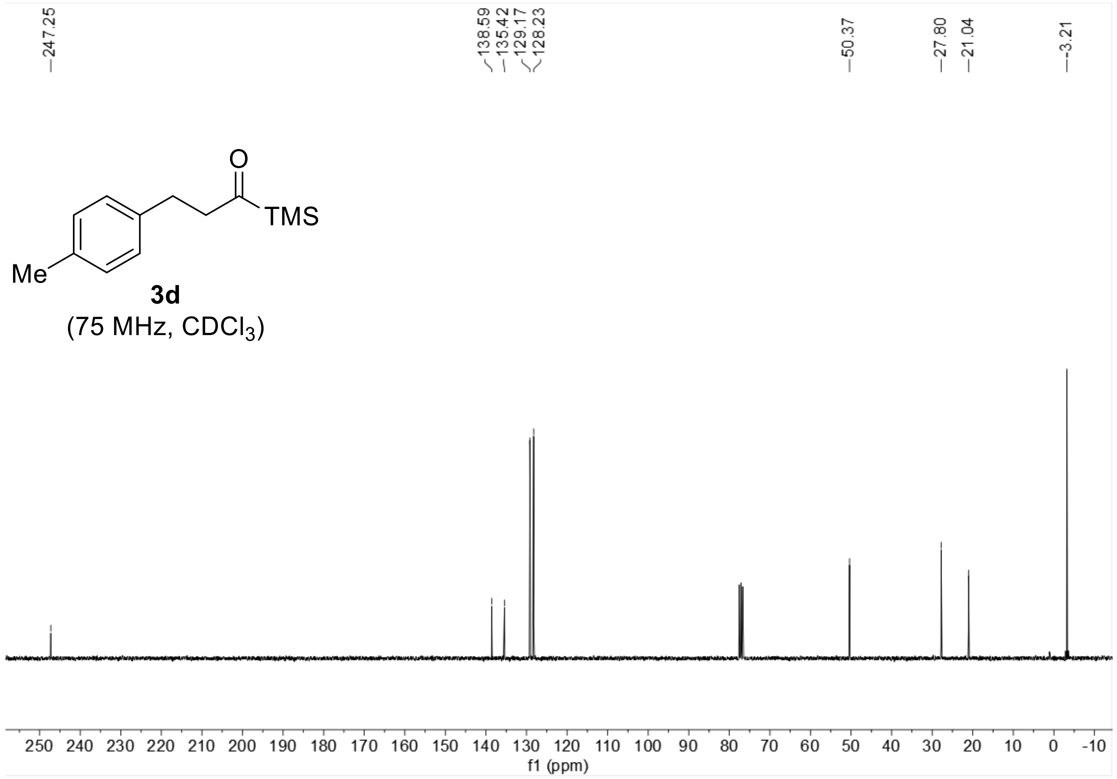
247.25
138.59
135.42
129.17
128.23
50.37
27.80
21.04
-3.21
O
TMS
Me
3d
(75 MHz, $CDCl_3$)
f1 (ppm)

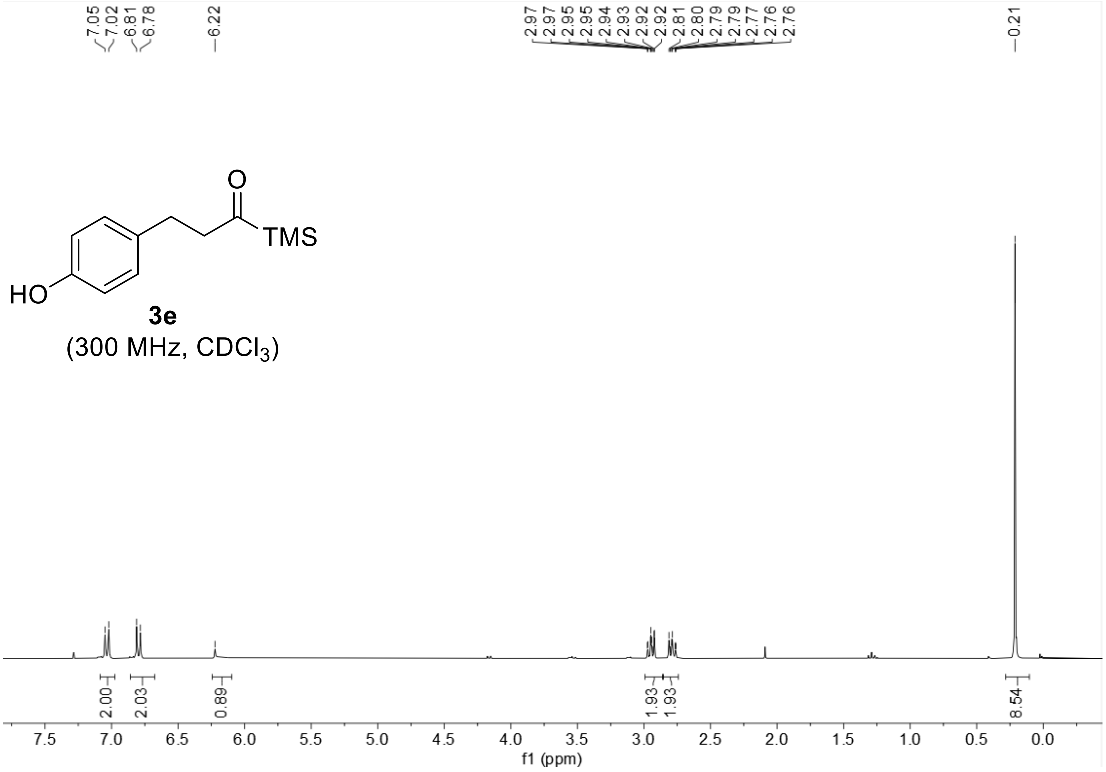
7.05
7.02
6.81
6.78
6.22
2.97
2.97
2.95
2.95
2.94
2.93
2.92
2.92
2.81
2.80
2.79
2.79
2.77
2.76
2.76
0.21
O
TMS
HO
3e
(300 MHz, CDCl3)
2.00
2.03
0.89
1.93
1.93
8.54
7.5
7.0
6.5
6.0
5.5
5.0
4.5
4.0
3.5
3.0
2.5
2.0
1.5
1.0
0.5
0.0
f1 (ppm)

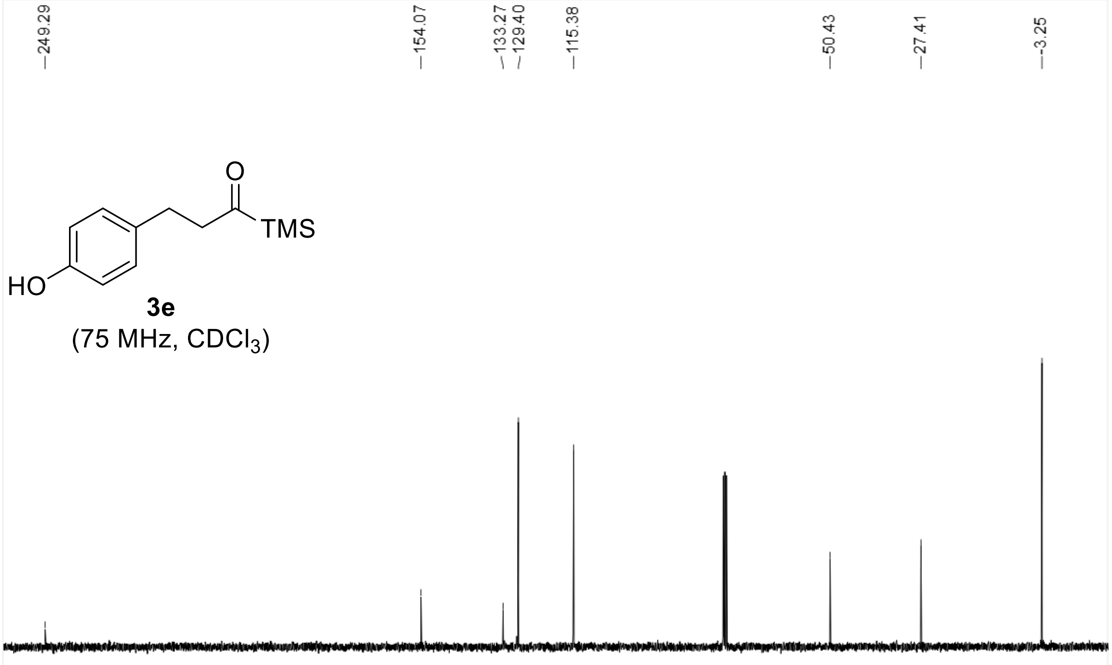
249.29
154.07
133.27
129.40
115.38
50.43
27.41
-3.25
O
TMS
HO
3e
(75 MHz, CDCl3)
250
240
230
220
210
200
190
180
170
160
150
140
130
120
110
100
90
80
70
60
50
40
30
20
10
0
-10
f1 (ppm)

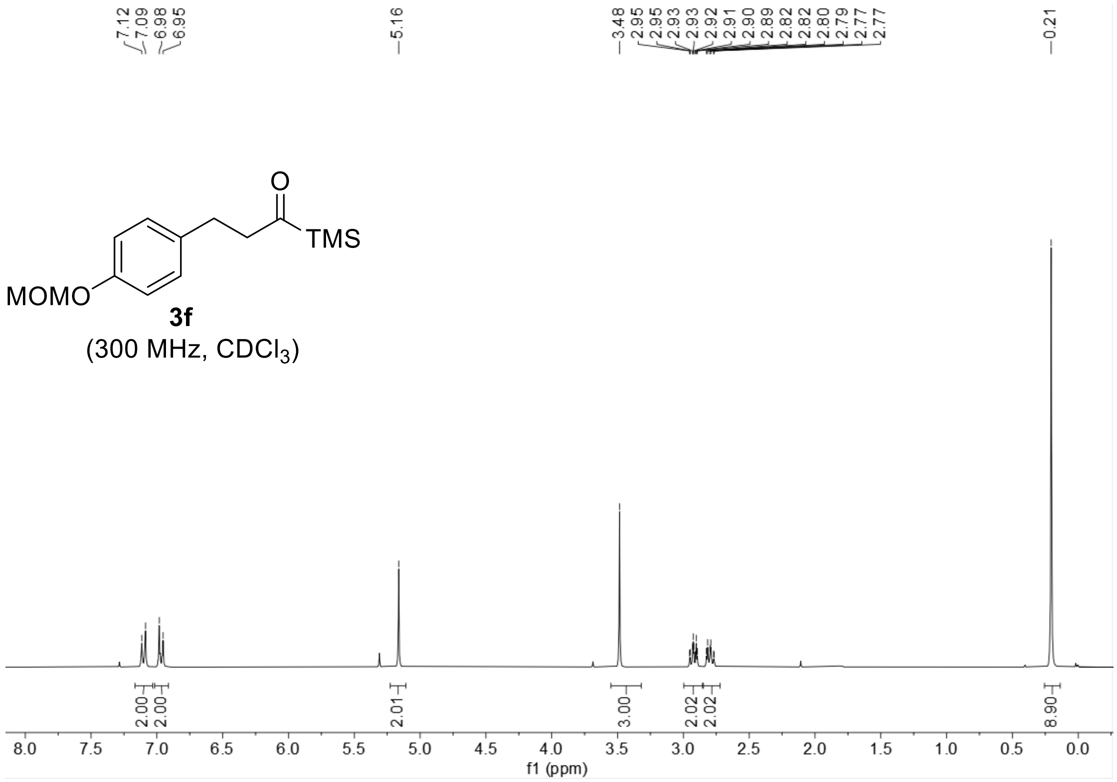
7.12
7.09
6.98
6.95
5.16
3.48
2.95
2.95
2.93
2.93
2.92
2.91
2.90
2.89
2.82
2.82
2.80
2.79
2.77
2.77
0.21
O
TMS
MOMO
3f
(300 MHz, CDCl3)
2.00
2.00
2.01
3.00
2.02
2.02
8.90
f1 (ppm)

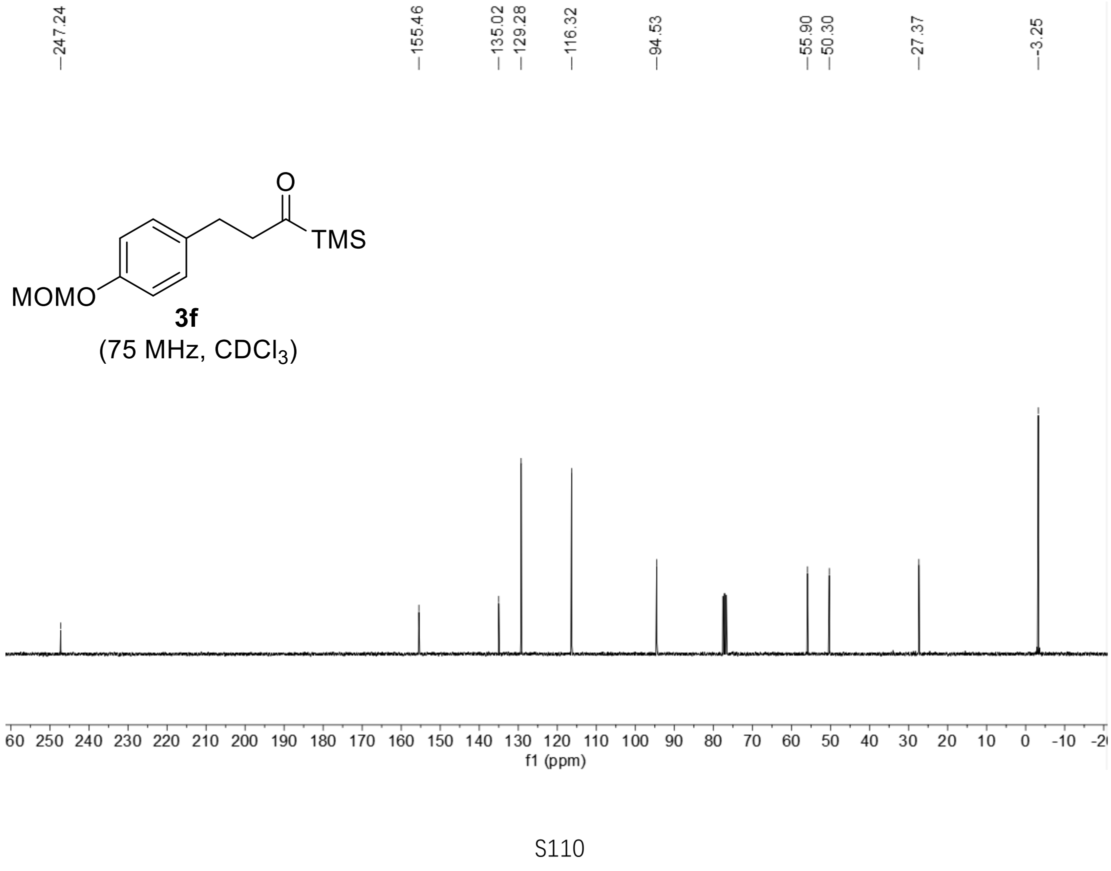
247.24
155.46
135.02
129.28
116.32
94.53
55.90
50.30
27.37
-3.25
O
TMS
MOMO
3f
(75 MHz, CDCl3)
f1 (ppm)

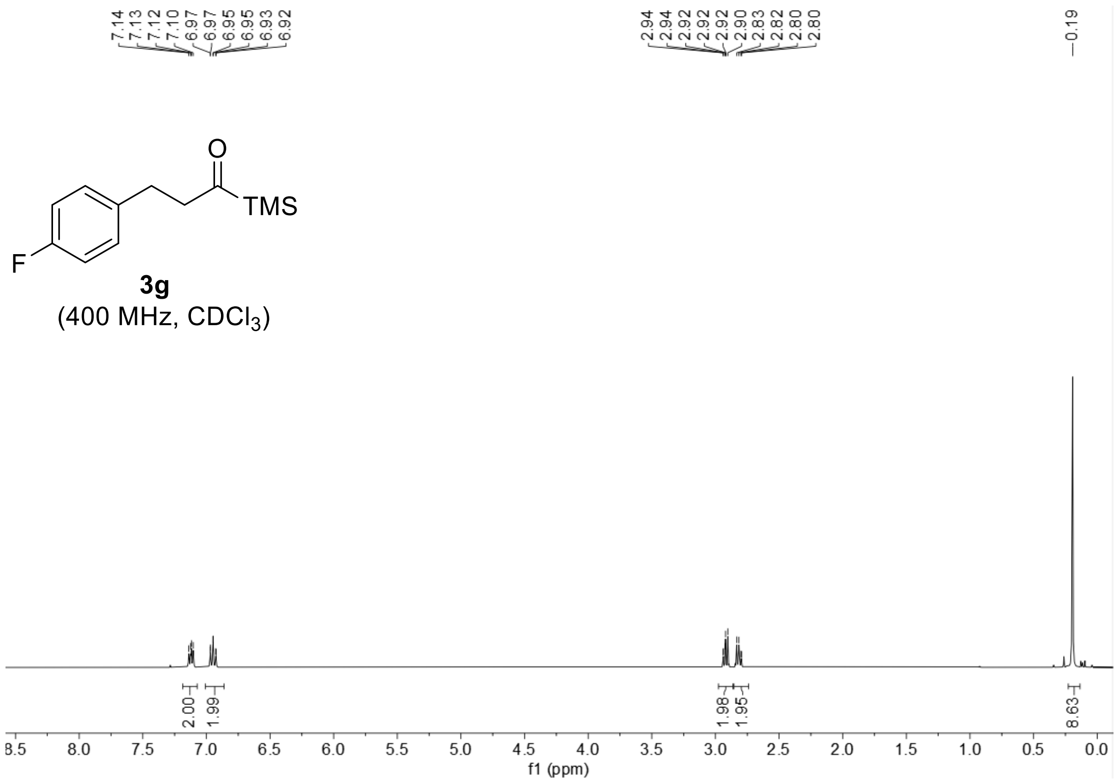

3g
(400 MHz, $CDCl_3$)

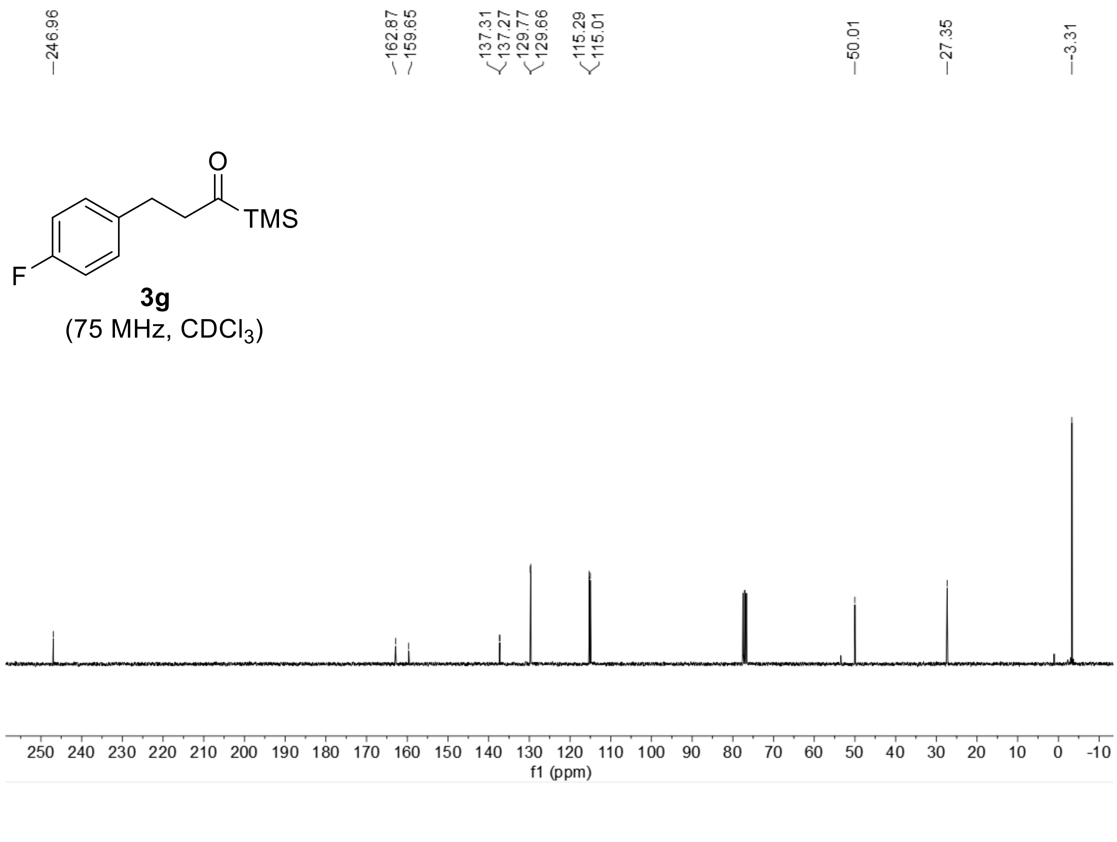

3g
(75 MHz, $CDCl_3$)

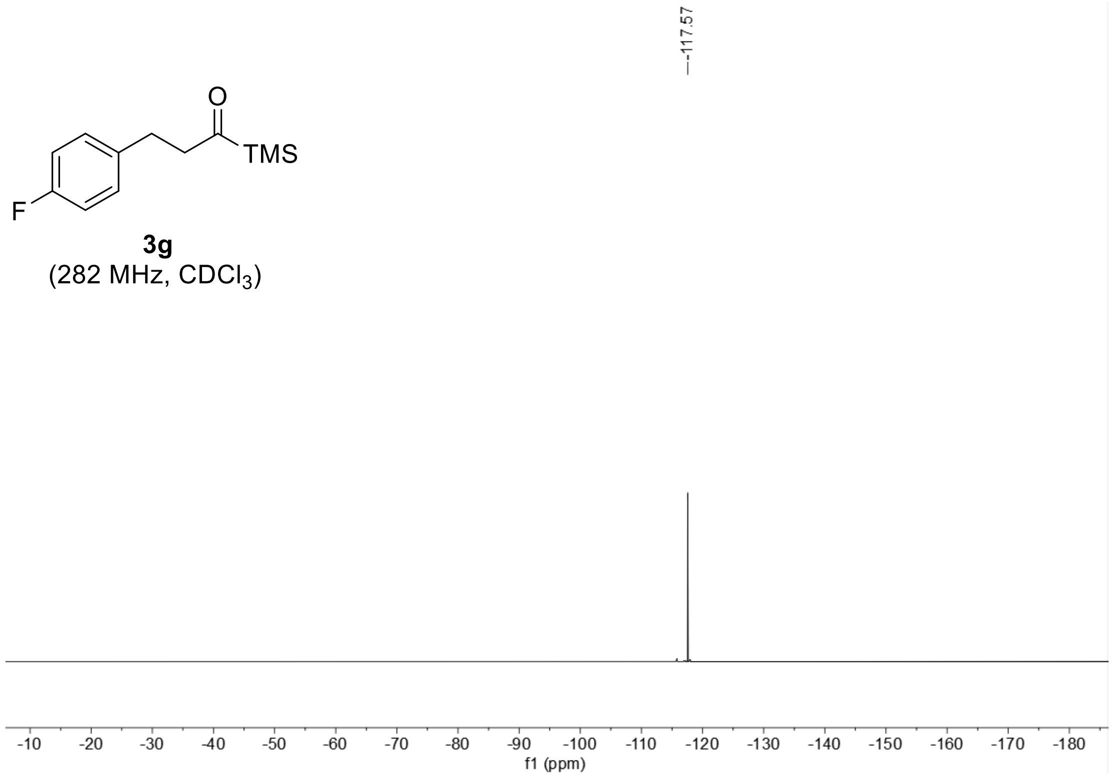
-117.57
O
TMS
F
3g
(282 MHz, CDCl3)
-10
-20
-30
-40
-50
-60
-70
-80
-90
-100
-110
-120
-130
-140
-150
-160
-170
-180
f1 (ppm)

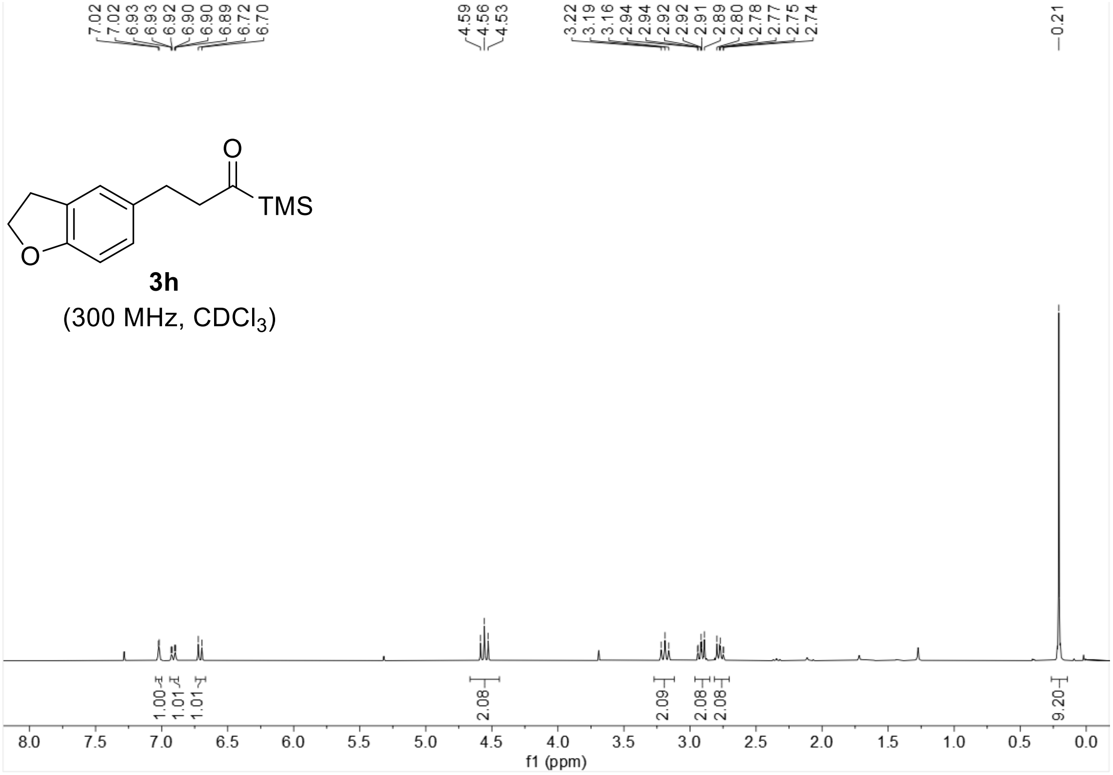

3h

(300 MHz, $CDCl_3$)

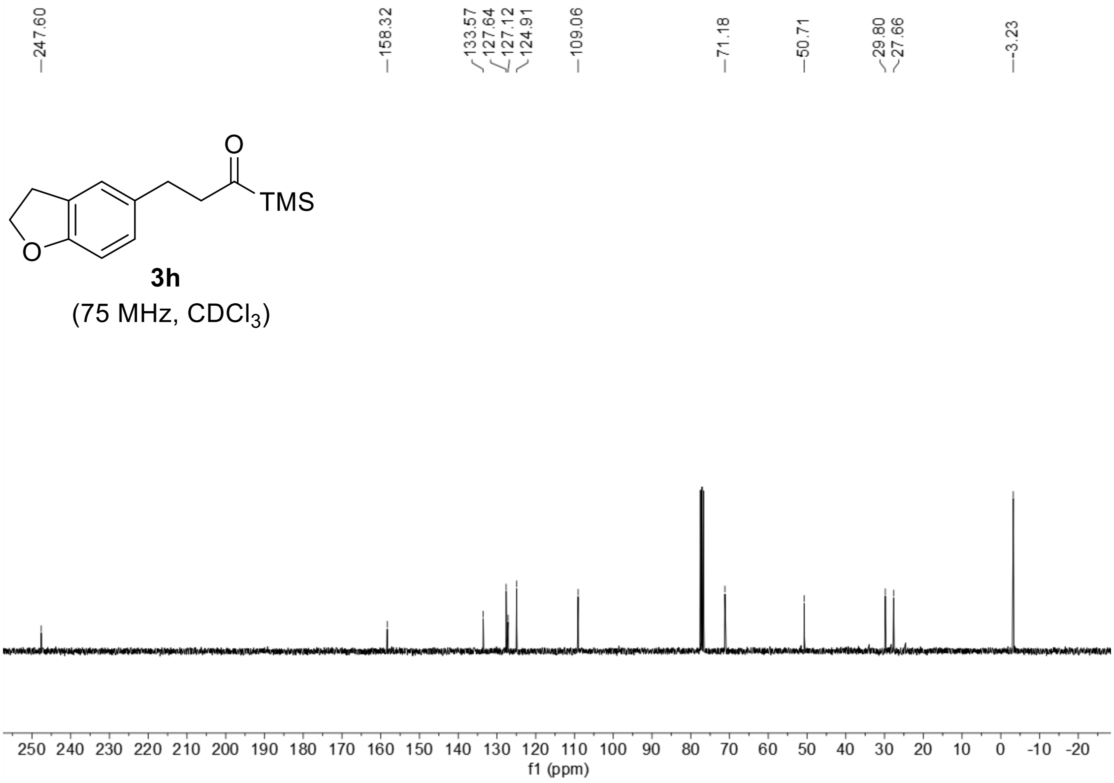

3h

(75 MHz, $CDCl_3$)

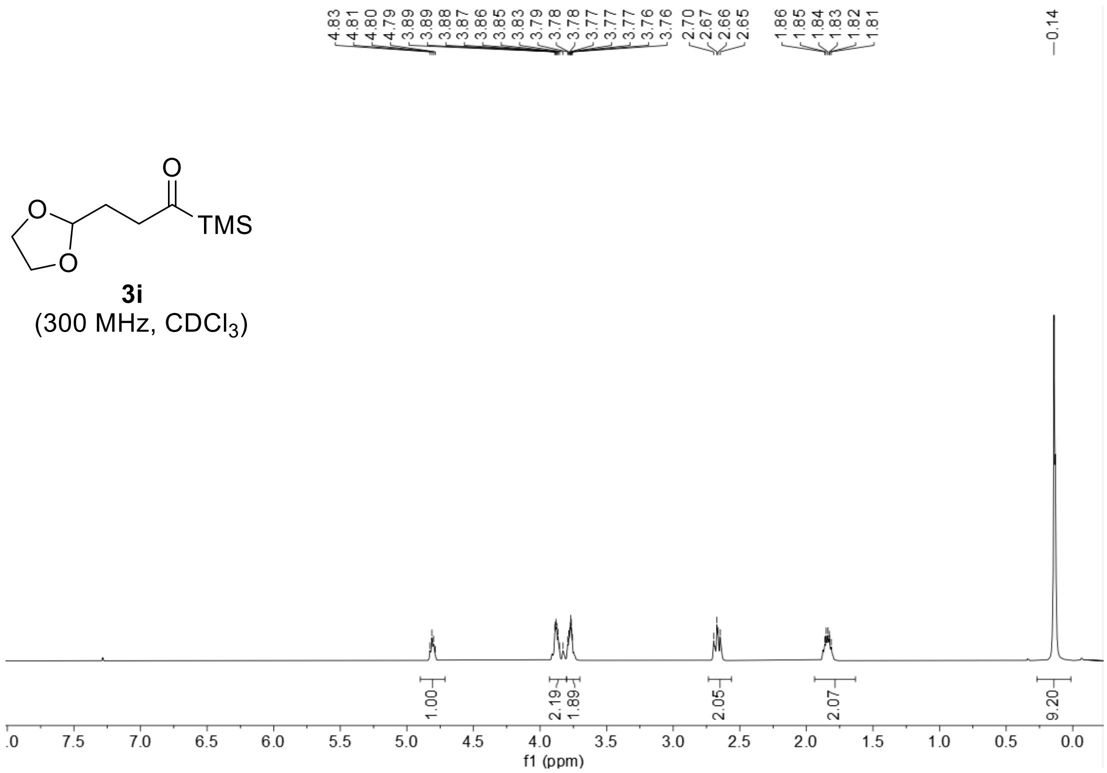
TMS
3i
(300 MHz, CDCl3)
f1 (ppm)

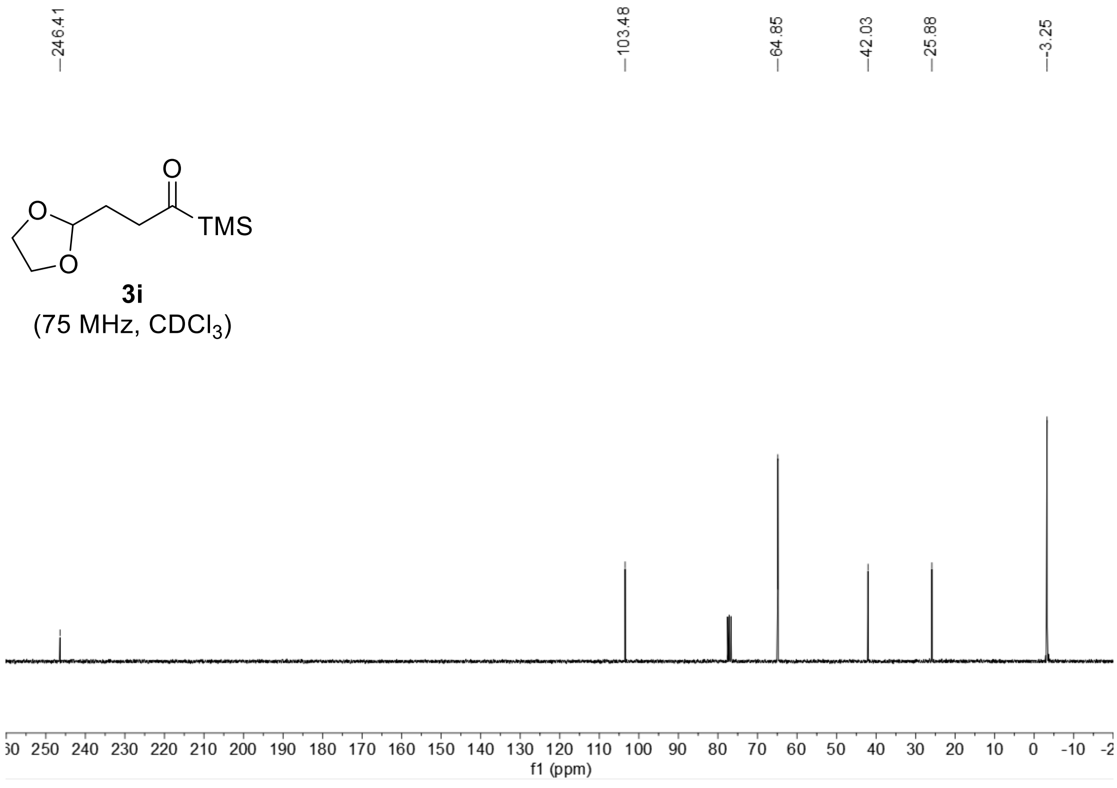
TMS
3i
(75 MHz, CDCl3)
246.41
103.48
64.85
42.03
25.88
-3.25
f1 (ppm)

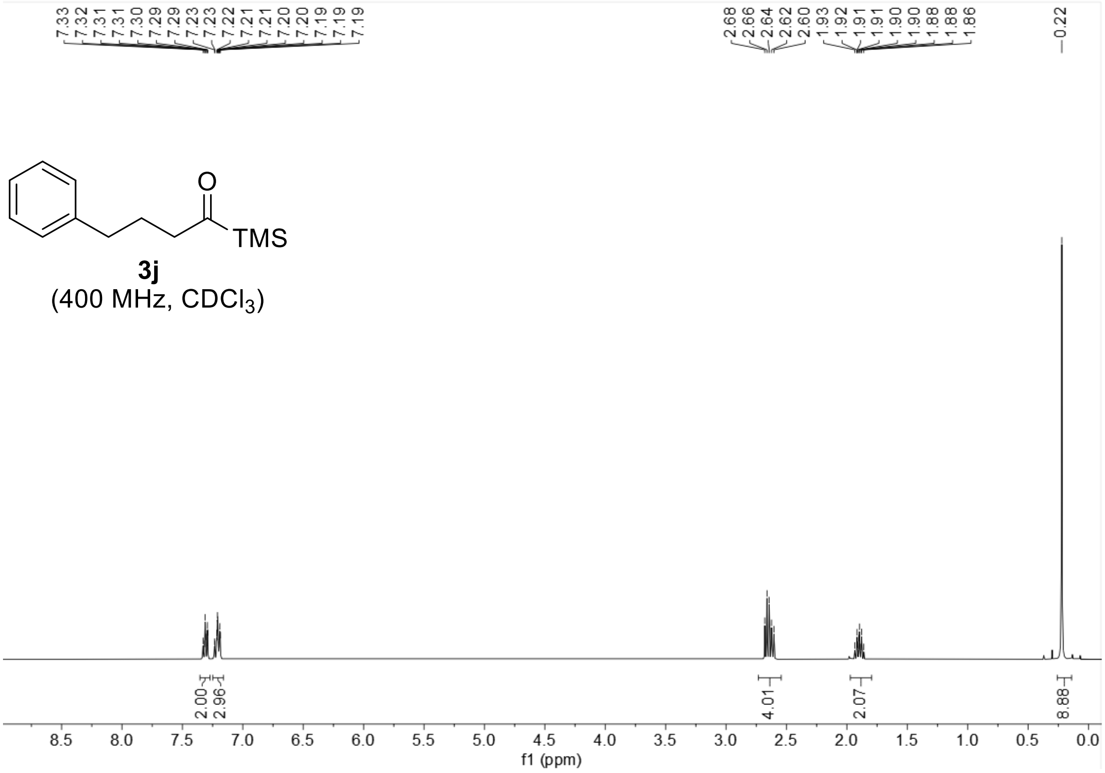

3j
(400 MHz, $CDCl_3$)

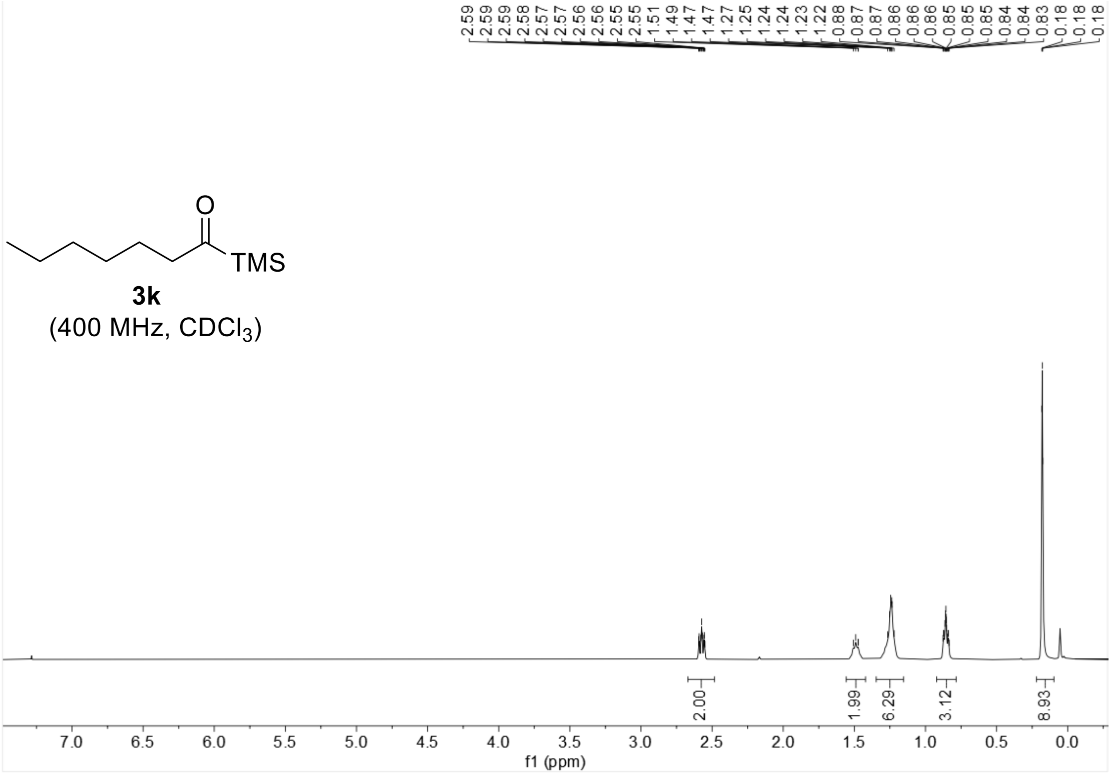

3k
(400 MHz, $CDCl_3$)

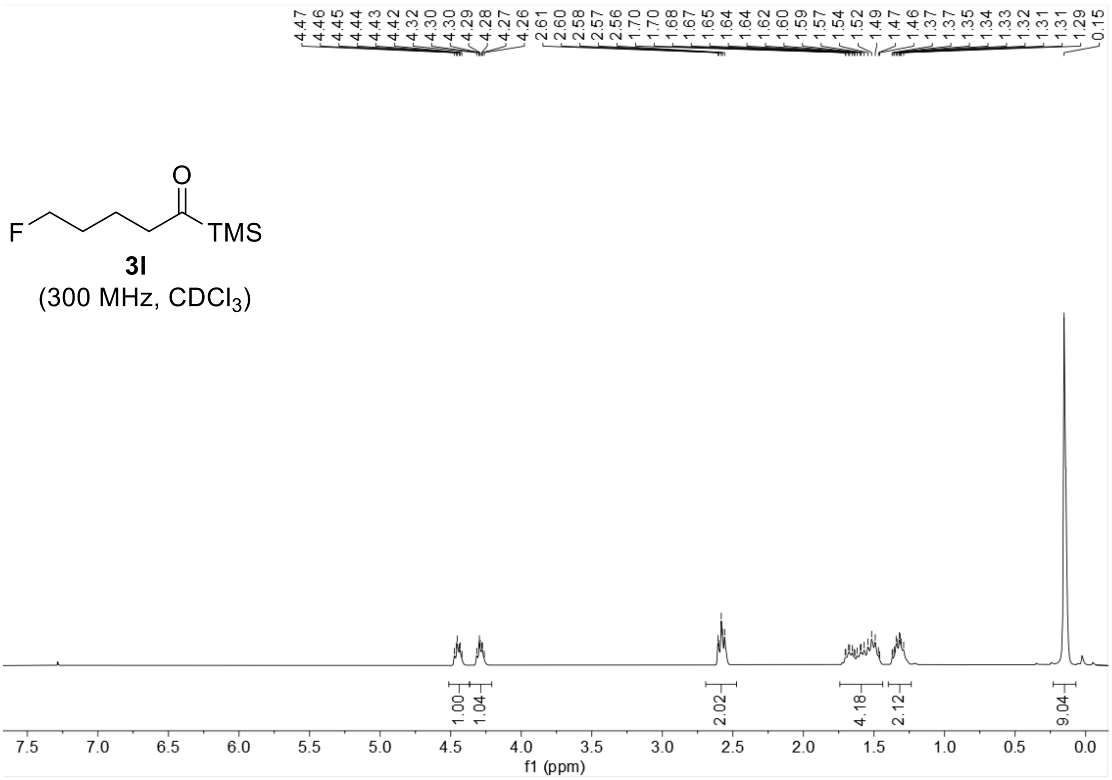
4.47
4.46
4.45
4.44
4.43
4.42
4.32
4.30
4.30
4.29
4.28
4.27
4.26
2.61
2.60
2.58
2.57
2.56
1.70
1.70
1.68
1.67
1.65
1.64
1.64
1.62
1.60
1.59
1.57
1.54
1.52
1.49
1.47
1.46
1.37
1.37
1.35
1.34
1.33
1.32
1.31
1.31
1.29
0.15
F
O
TMS
3l
(300 MHz, CDCl3)
1.00
1.04
2.02
4.18
2.12
9.04
f1 (ppm)

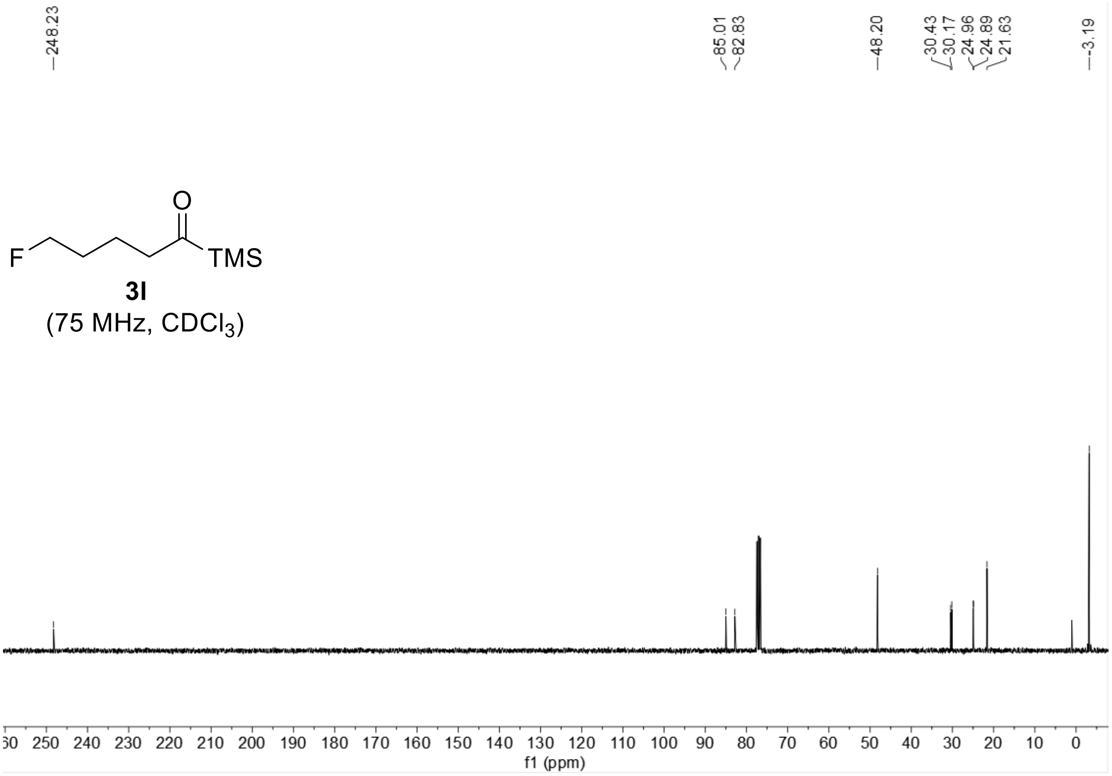
248.23
85.01
82.83
48.20
30.43
30.17
24.96
24.89
21.63
-3.19
F
O
TMS
3l
(75 MHz, CDCl3)
f1 (ppm)

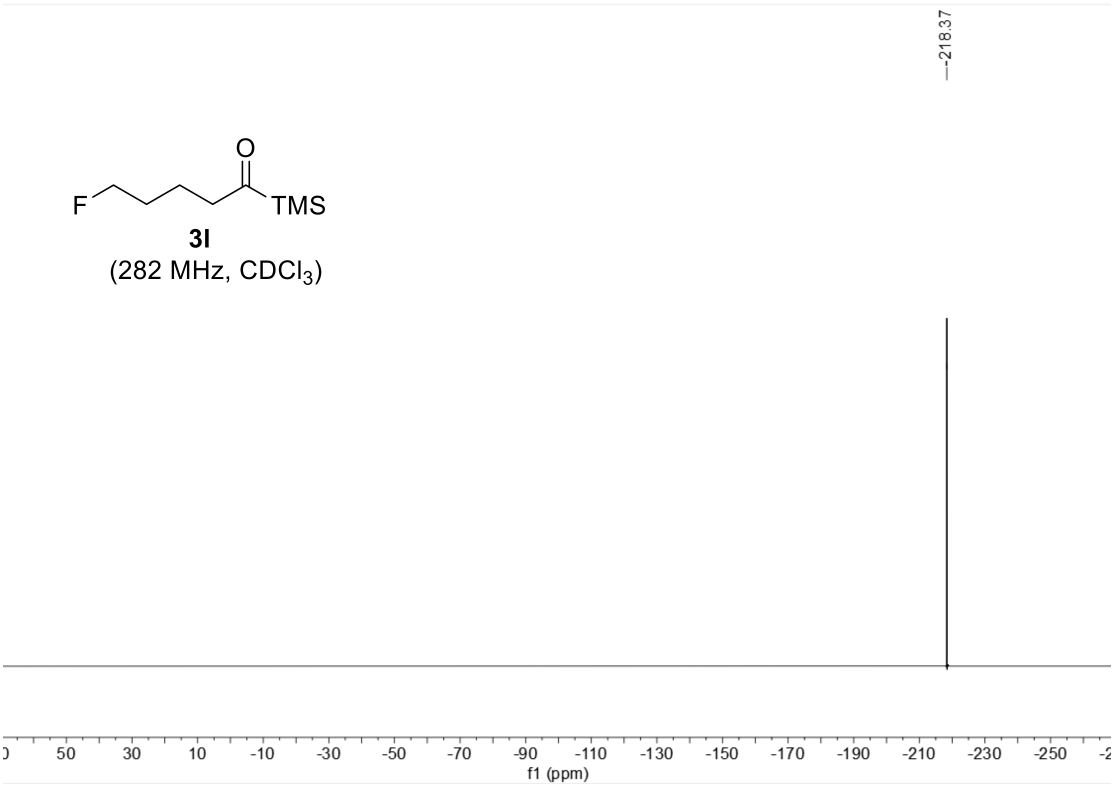
-218.37
F
O
TMS
3l
(282 MHz, CDCl3)
50
30
10
-10
-30
-50
-70
-90
-110
-130
-150
-170
-190
-210
-230
-250
f1 (ppm)

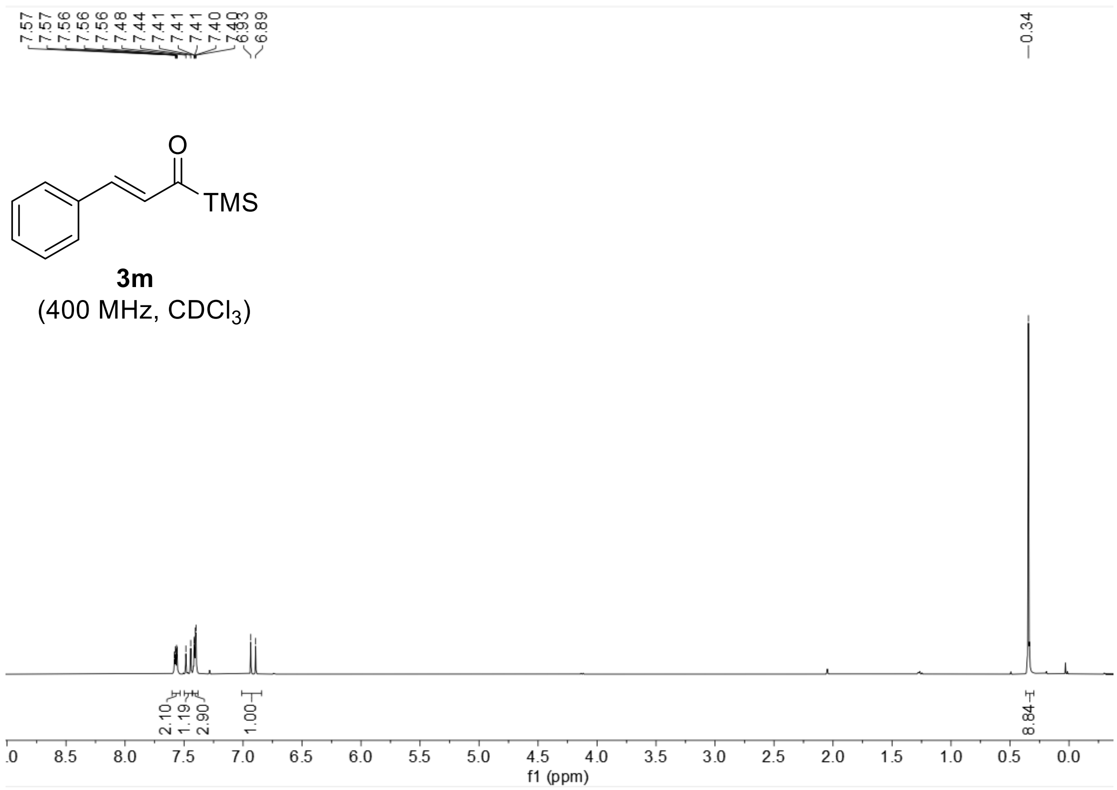

3m
(400 MHz, $CDCl_3$)

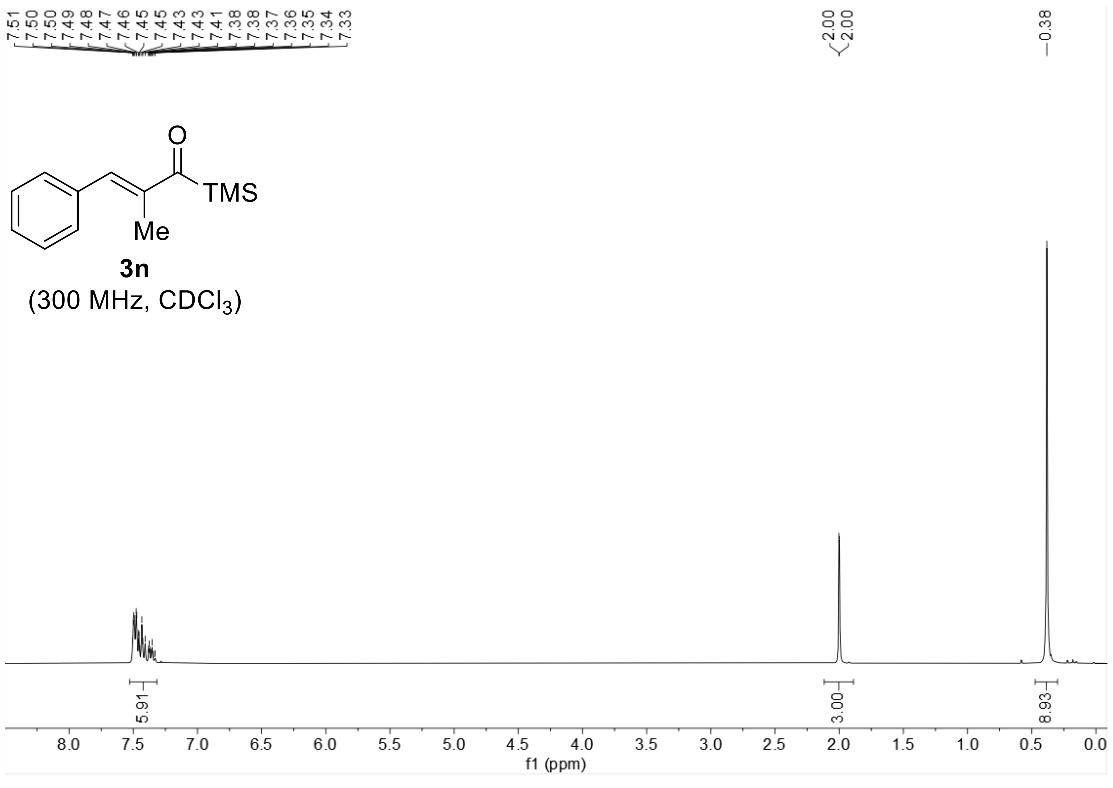

3n
(300 MHz, $CDCl_3$)

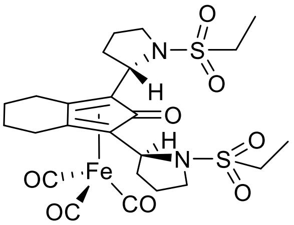

Fe1

(300 MHz, $CDCl_3$)

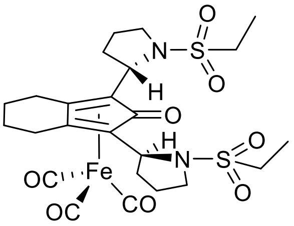

Fe1

(75 MHz, $CDCl_3$)

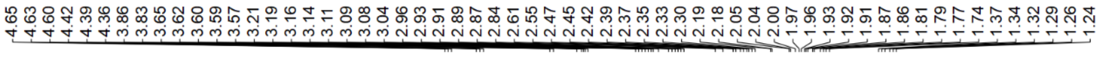

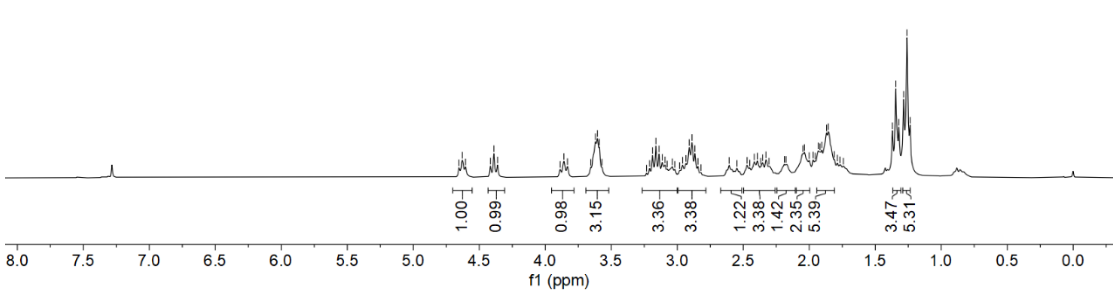

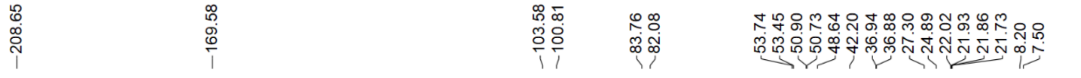

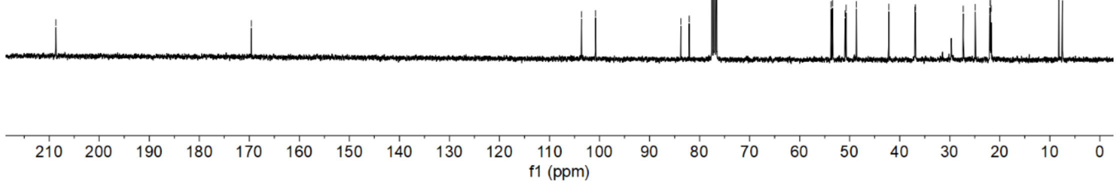

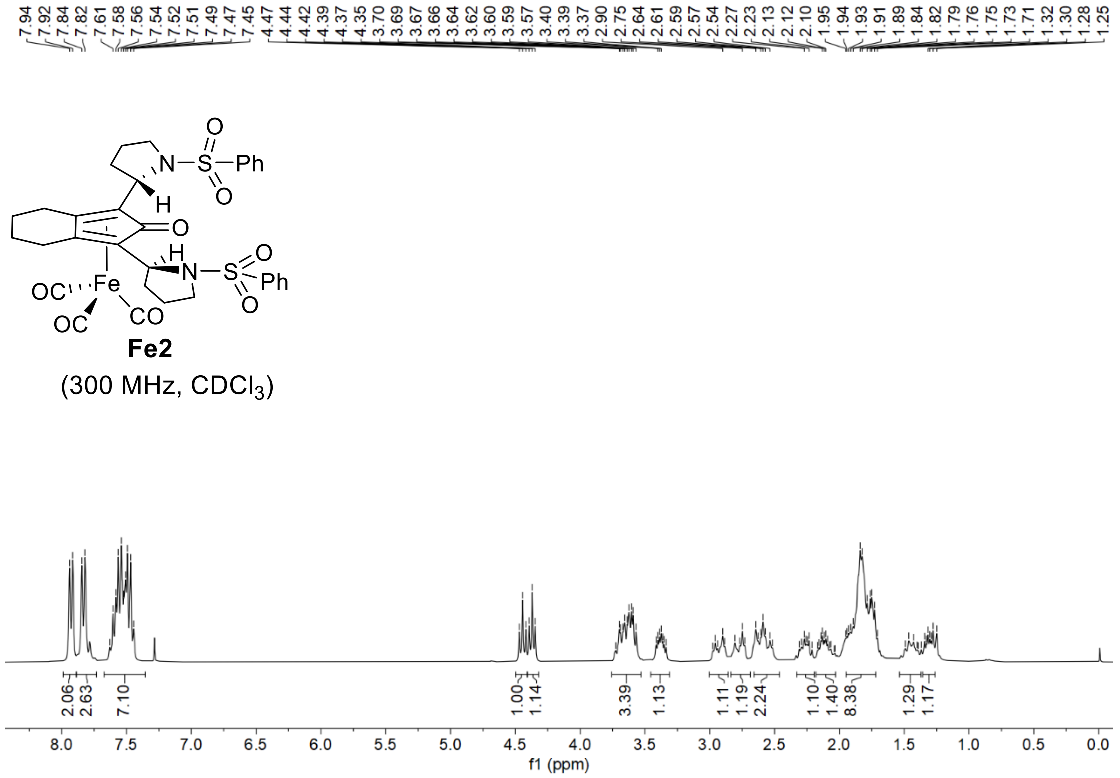
7.94 7.92 7.84 7.82 7.61 7.58 7.56 7.54 7.52 7.51 7.49 7.47 7.45 4.47 4.44 4.42 4.39 4.37 4.35 3.70 3.69 3.67 3.66 3.64 3.62 3.60 3.59 3.57 3.40 3.39 3.37 2.90 2.75 2.64 2.61 2.59 2.57 2.54 2.27 2.23 2.13 2.12 2.10 1.95 1.94 1.93 1.91 1.89 1.84 1.82 1.79 1.76 1.75 1.73 1.71 1.32 1.30 1.28 1.25
Fe2
(300 MHz, CDCl3)
2.06 2.63 7.10 1.00 1.14 3.39 1.13 1.11 1.19 2.24 1.10 1.40 8.38 1.29 1.17
f1 (ppm)

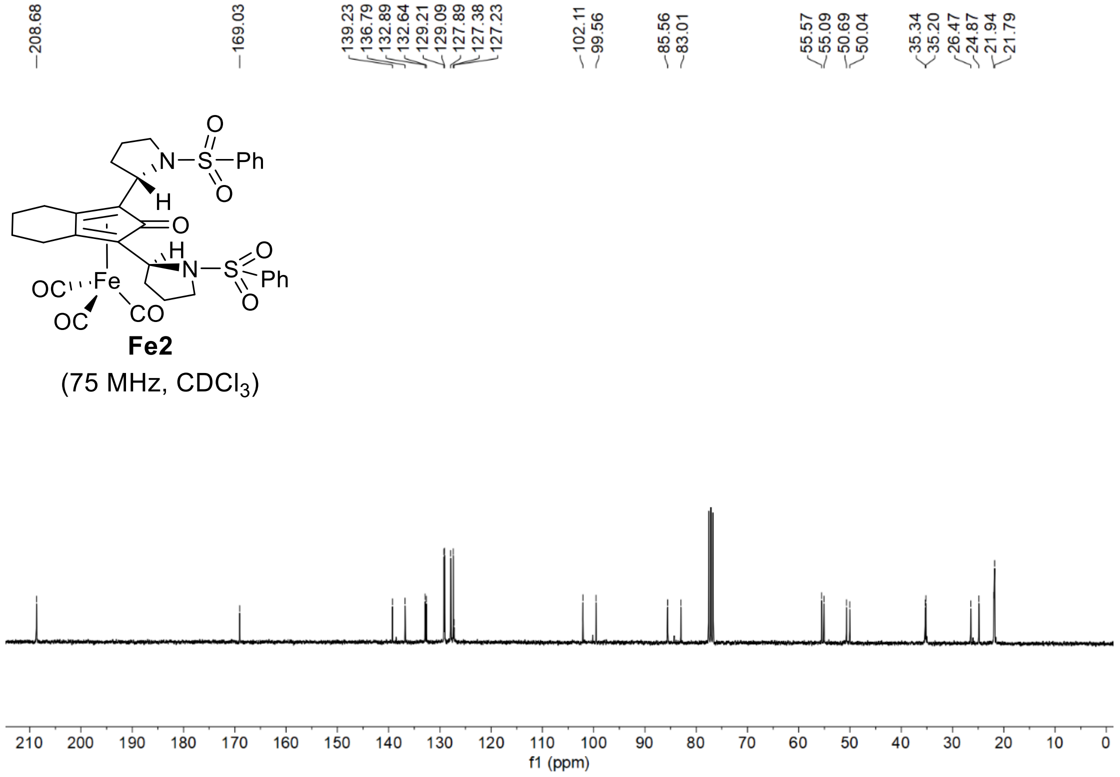
208.68 169.03 139.23 136.79 132.89 132.64 129.21 129.09 127.89 127.38 127.23 102.11 99.56 85.56 83.01 55.57 55.09 50.69 50.04 35.34 35.20 26.47 24.87 21.94 21.79
Fe2
(75 MHz, CDCl3)
f1 (ppm)

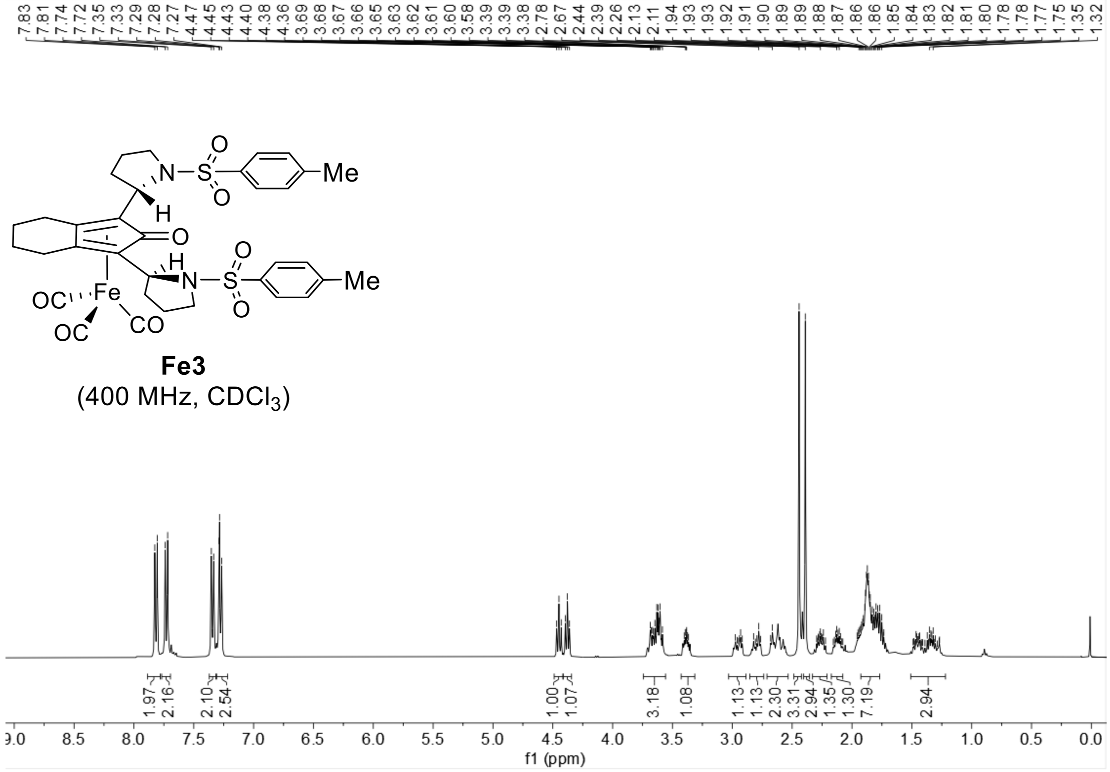
7.83 7.81 7.74 7.72 7.35 7.33 7.29 7.28 7.27 4.47 4.45 4.43 4.40 4.38 4.36 3.69 3.68 3.67 3.66 3.65 3.63 3.62 3.61 3.60 3.58 3.39 3.39 3.38 2.78 2.67 2.44 2.39 2.26 2.13 2.11 1.94 1.93 1.93 1.92 1.91 1.90 1.89 1.89 1.88 1.87 1.86 1.86 1.85 1.84 1.83 1.82 1.81 1.80 1.78 1.77 1.75 1.35 1.32
Fe3
(400 MHz, CDCl$_3$)
1.97 2.16 2.10 2.54 1.00 1.07 3.18 1.08 1.13 1.13 2.30 3.31 2.94 1.35 1.30 7.19 2.94
f1 (ppm)

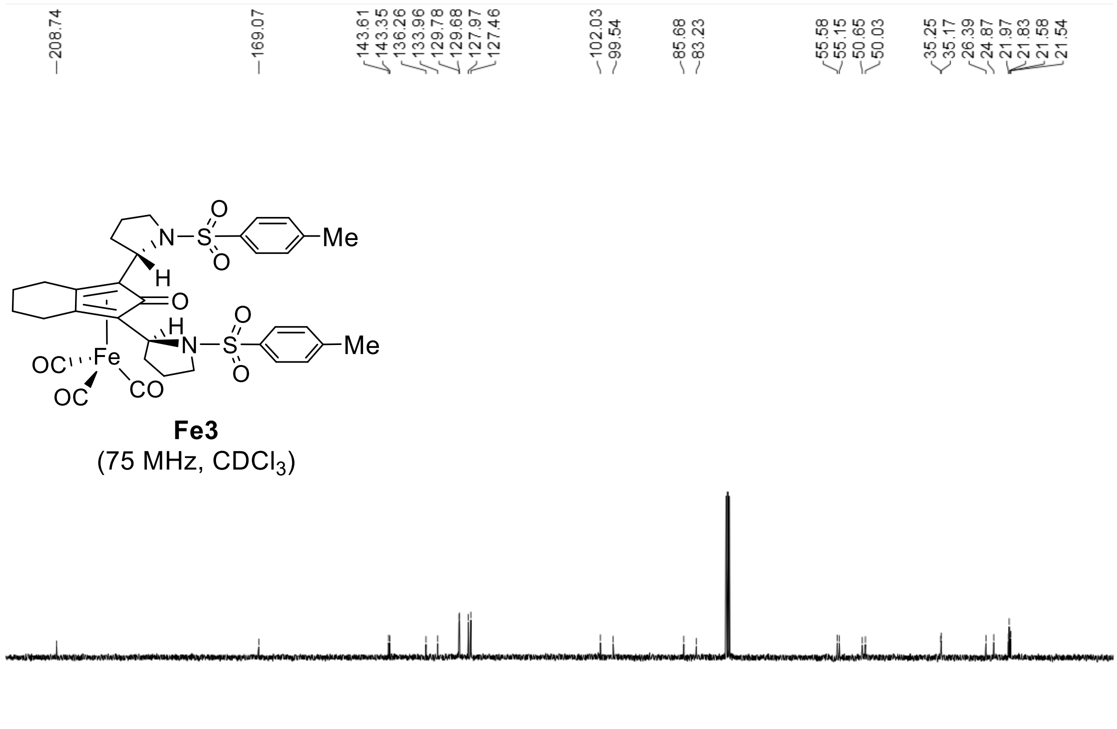
208.74 169.07 143.61 143.35 136.26 133.96 129.78 129.68 127.97 127.46 102.03 99.54 85.68 83.23 55.58 55.15 50.65 50.03 35.25 35.17 26.39 24.87 21.97 21.83 21.58 21.54
Fe3
(75 MHz, CDCl$_3$)
f1 (ppm)

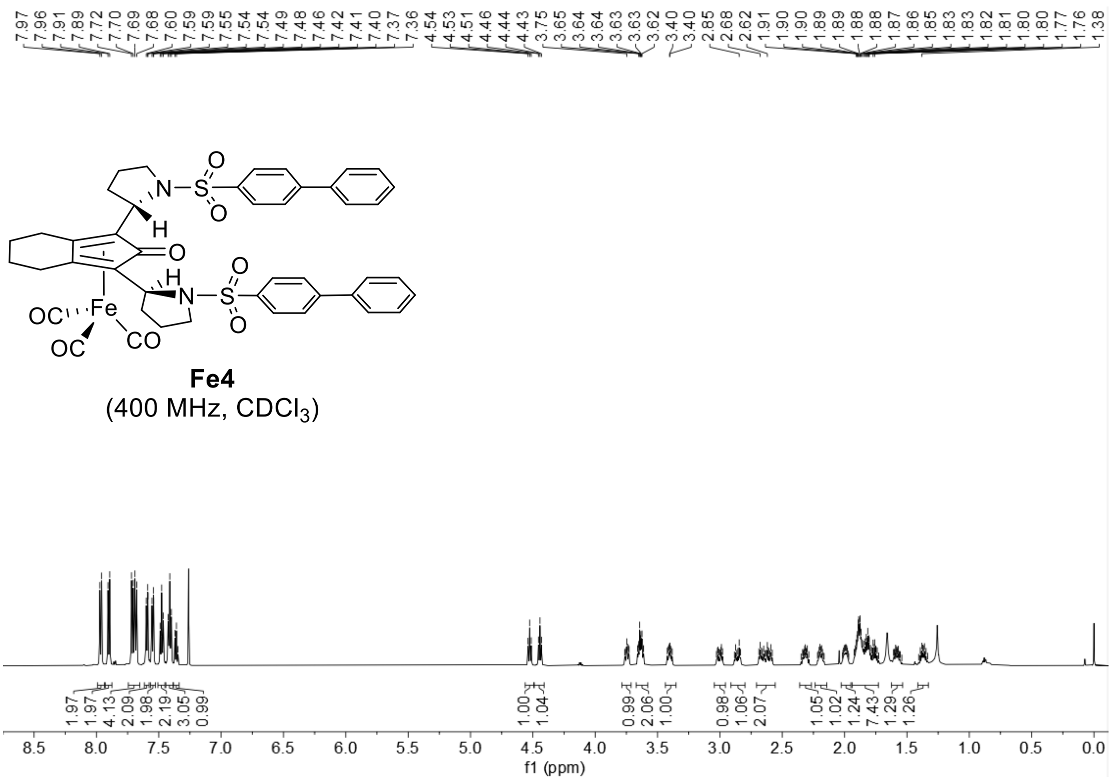

Fe4
(400 MHz, $CDCl_3$)

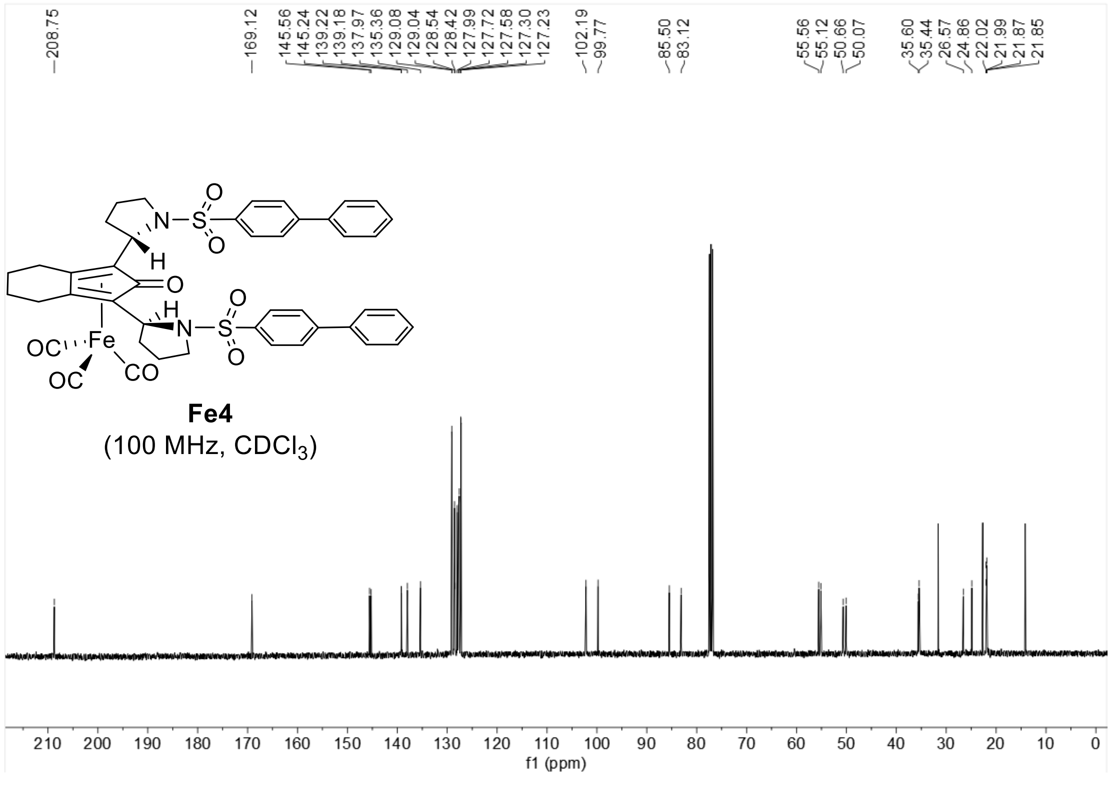

Fe4
(100 MHz, $CDCl_3$)

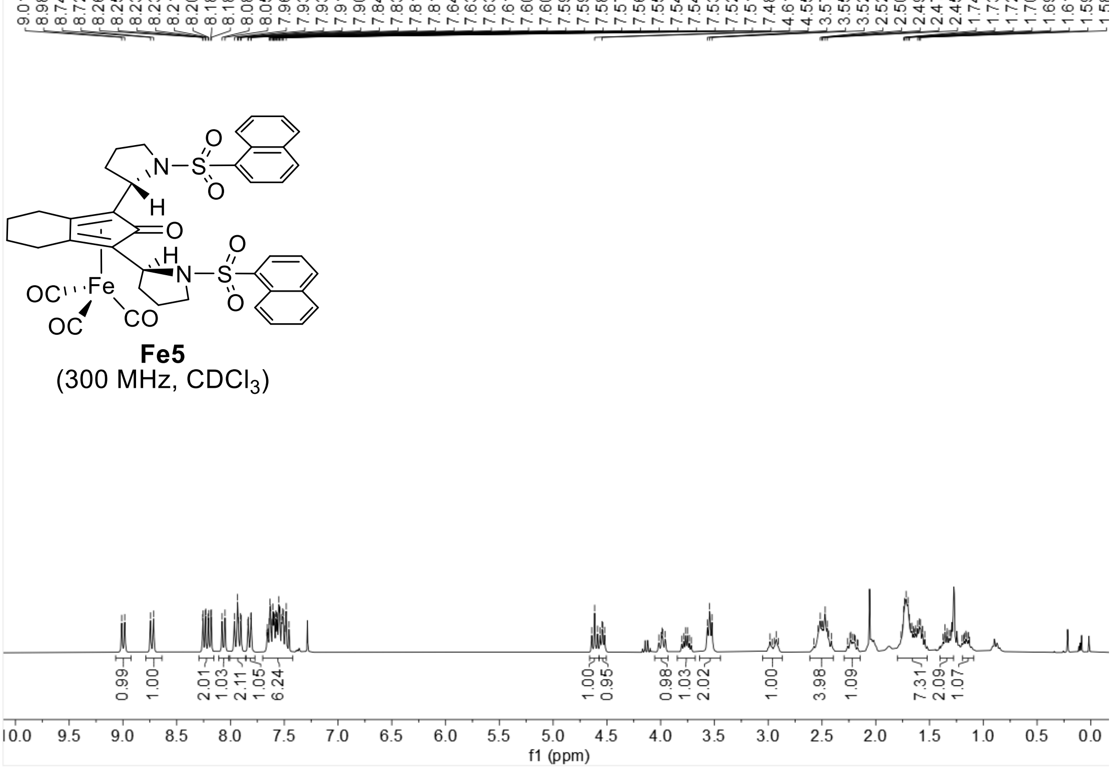

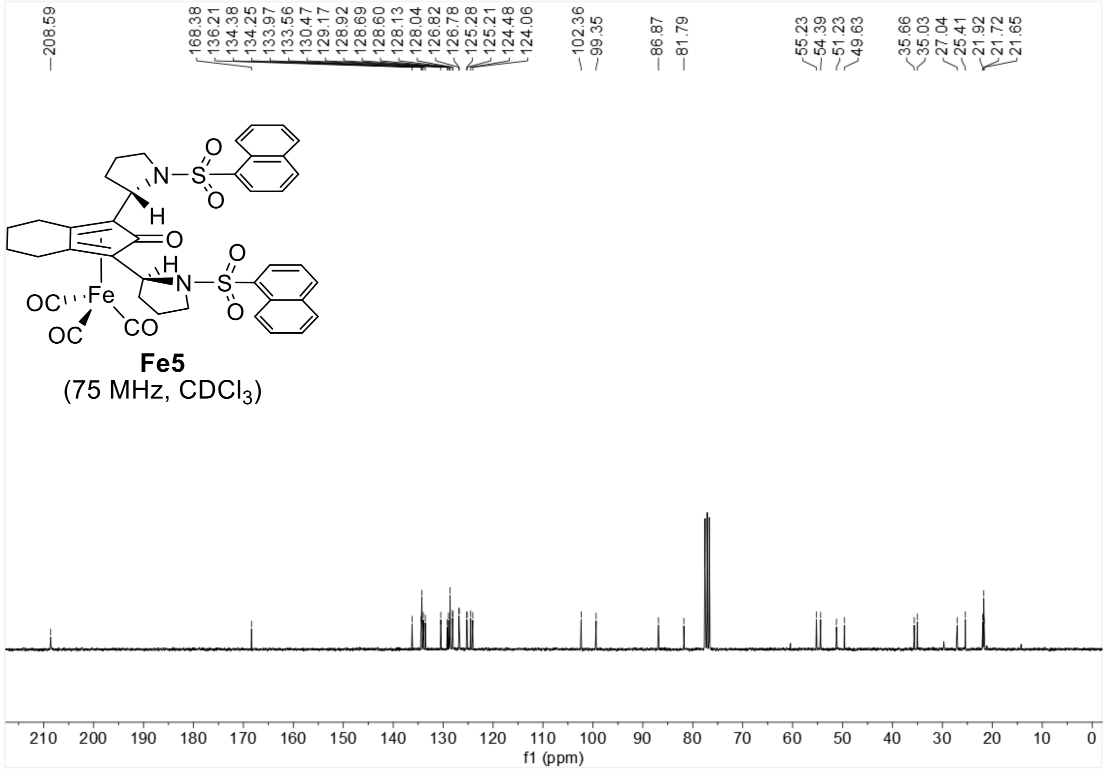

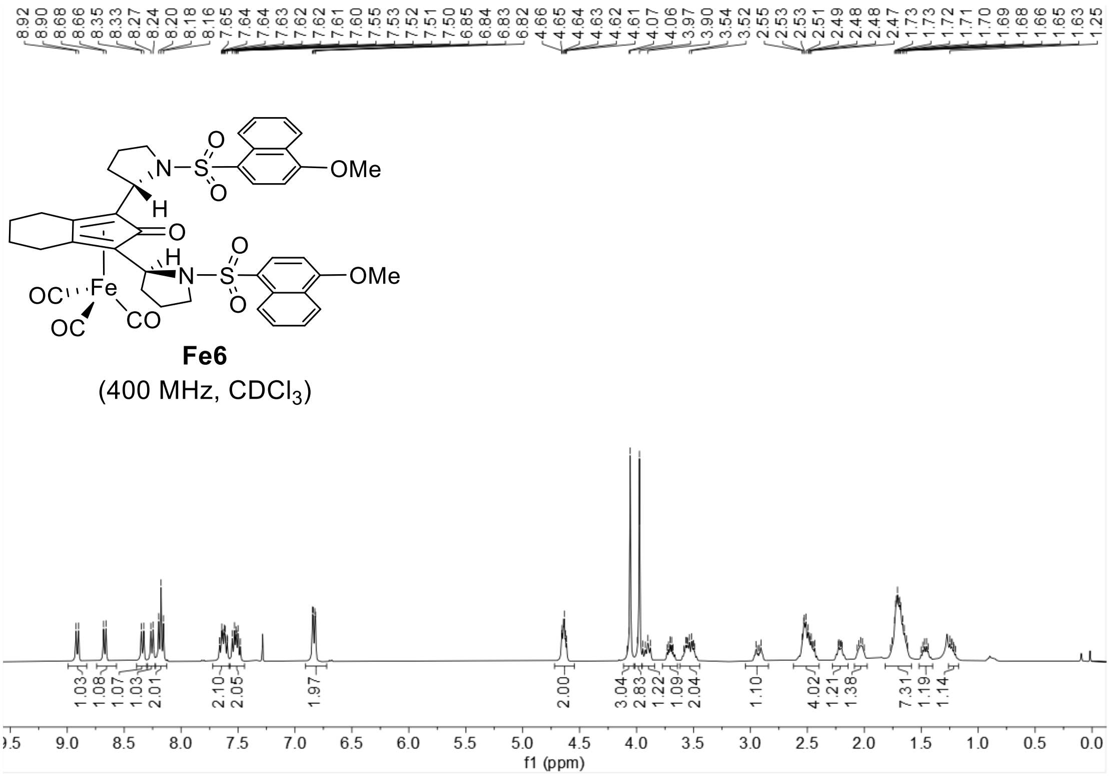
8.92 8.90 8.68 8.66 8.35 8.33 8.27 8.24 8.20 8.18 8.16 7.65 7.64 7.64 7.63 7.62 7.62 7.61 7.60 7.55 7.53 7.52 7.51 7.50 6.85 6.84 6.83 6.82 4.66 4.65 4.64 4.63 4.62 4.61 4.07 4.06 3.97 3.90 3.54 3.52 2.55 2.53 2.53 2.51 2.49 2.48 2.48 2.47 1.73 1.73 1.72 1.71 1.70 1.69 1.68 1.66 1.65 1.63 1.25
OMe
OMe
Fe6
(400 MHz, CDCl3)
1.03 1.08 1.07 1.03 2.01 2.10 2.05 1.97 2.00 3.04 2.83 1.22 1.09 2.04 1.10 4.02 1.21 1.38 7.31 1.19 1.14
f1 (ppm)

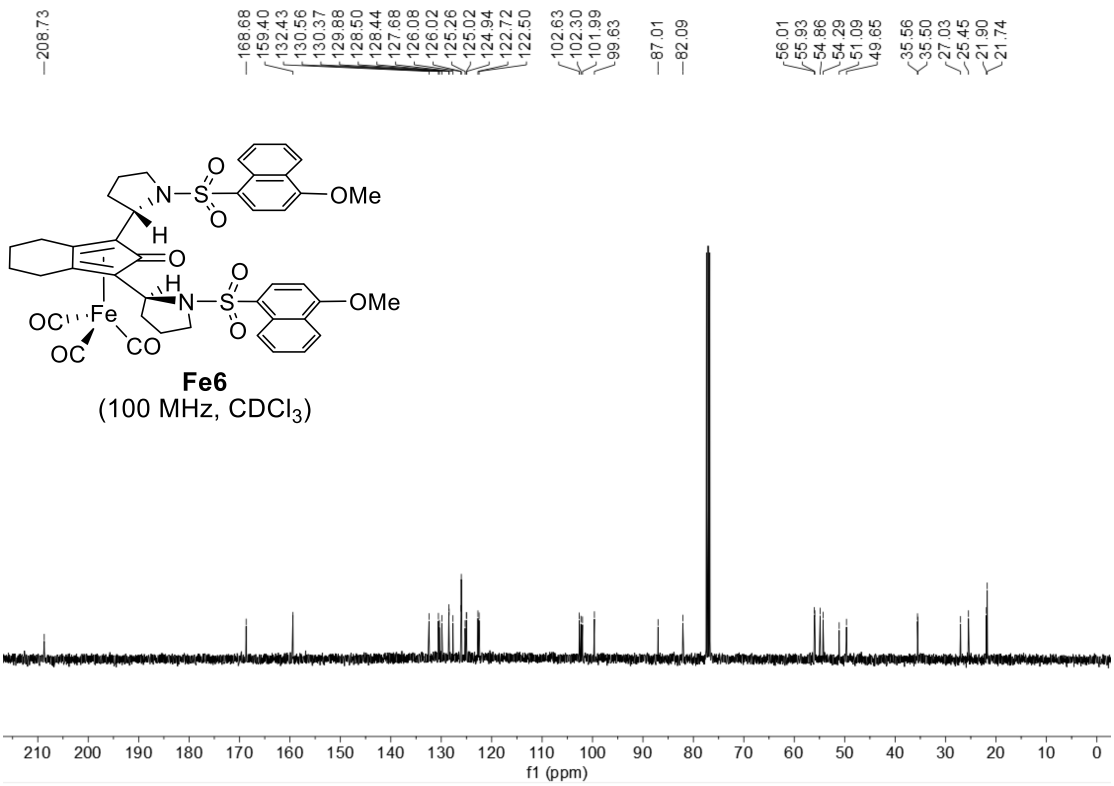
208.73 168.68 159.40 132.43 130.56 130.37 129.88 128.50 128.44 127.68 126.08 126.02 125.26 125.02 124.94 122.72 122.50 102.63 102.30 101.99 99.63 87.01 82.09 56.01 55.93 54.86 54.29 51.09 49.65 35.56 35.50 27.03 25.45 21.90 21.74
OMe
OMe
Fe6
(100 MHz, CDCl3)
f1 (ppm)

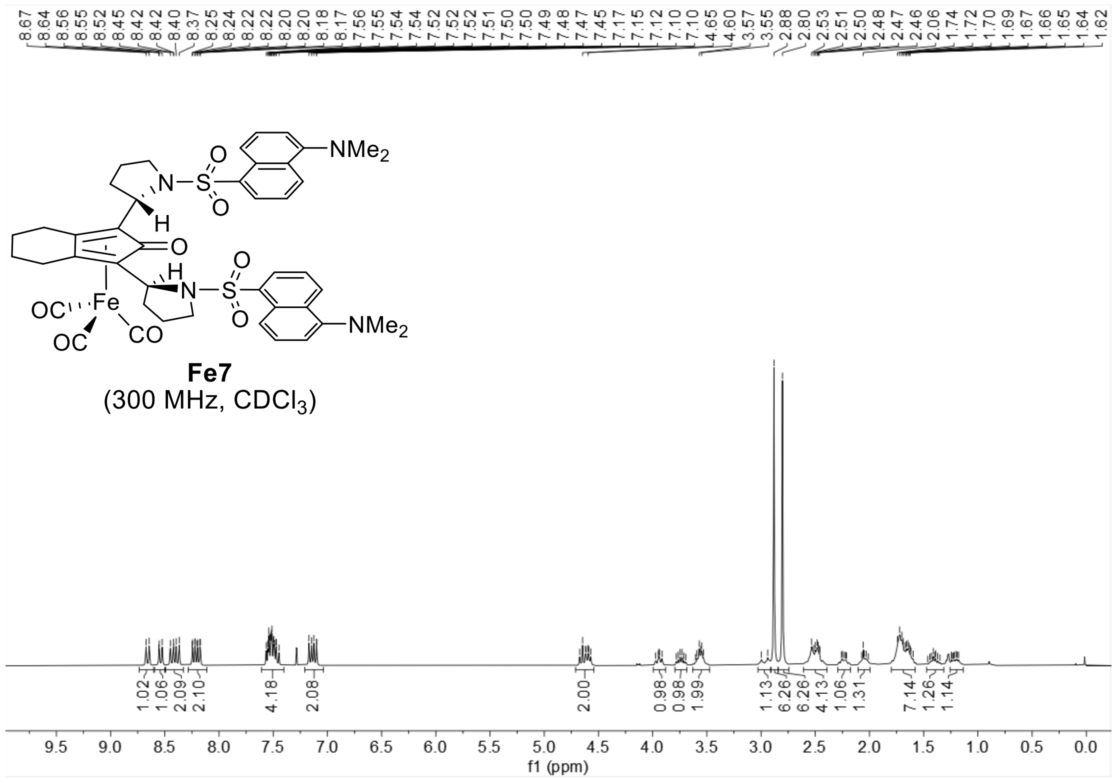
NMe2
Fe7
(300 MHz, CDCl3)
f1 (ppm)

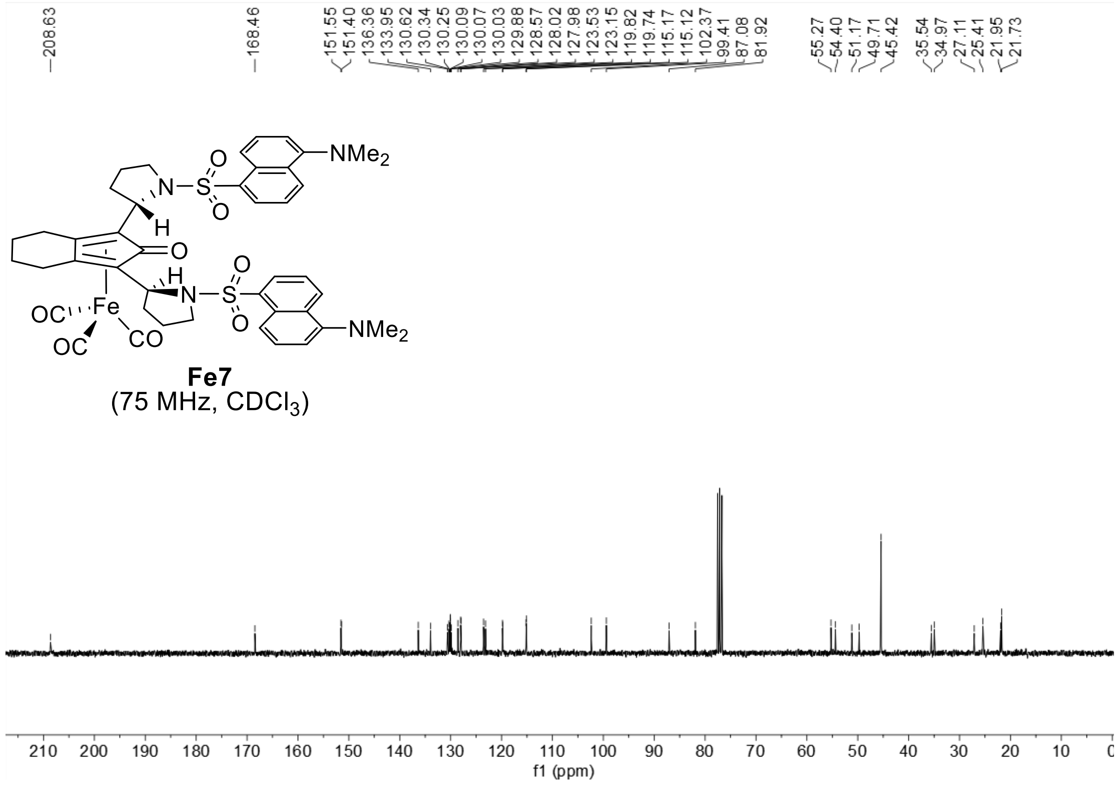
NMe2
Fe7
(75 MHz, CDCl3)
f1 (ppm)

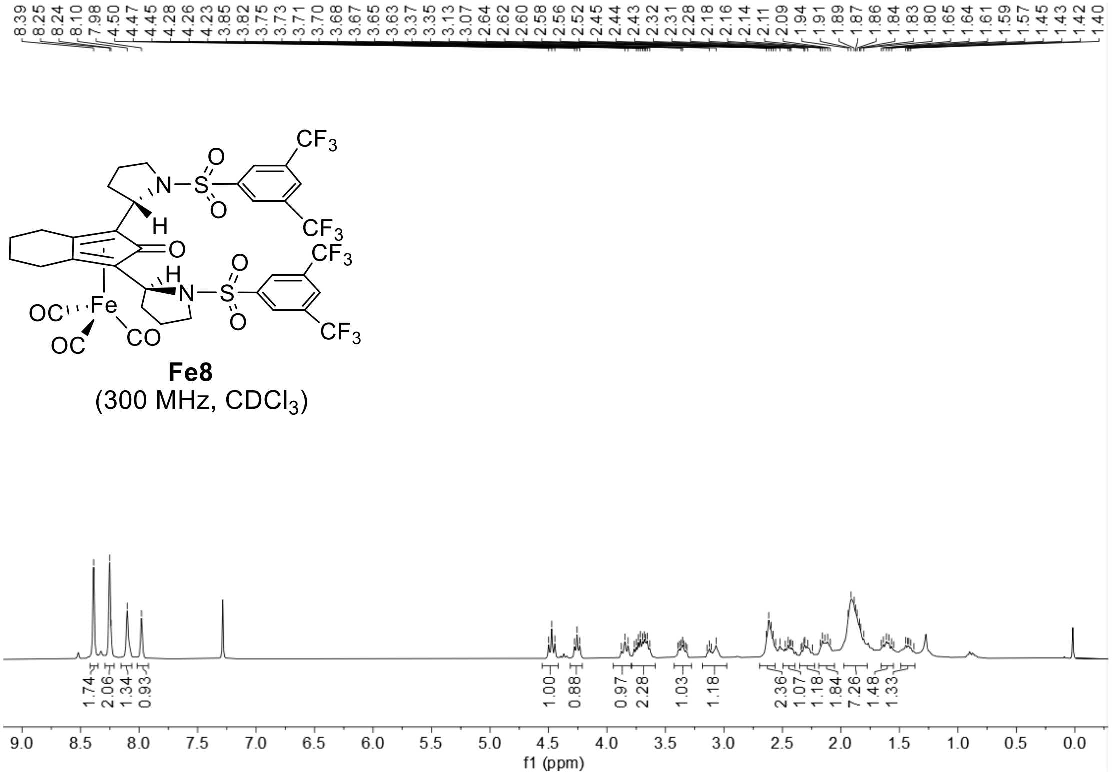
8.39 8.25 8.24 8.10 7.98 4.50 4.47 4.45 4.28 4.26 4.23 3.85 3.82 3.75 3.73 3.71 3.70 3.68 3.67 3.65 3.63 3.37 3.35 3.13 3.07 2.64 2.62 2.60 2.58 2.56 2.52 2.45 2.44 2.43 2.32 2.31 2.28 2.18 2.16 2.14 2.11 2.09 1.94 1.91 1.89 1.87 1.86 1.84 1.83 1.80 1.65 1.64 1.61 1.59 1.57 1.45 1.43 1.42 1.40
CF3
N
S
O
O
H
O
CF3
CF3
H
N
S
O
O
CF3
OC
Fe
OC
CO
Fe8
(300 MHz, CDCl3)
1.74 2.06 1.34 0.93 1.00 0.88 0.97 2.28 1.03 1.18 2.36 1.07 1.18 1.84 7.26 1.48 1.33
9.0 8.5 8.0 7.5 7.0 6.5 6.0 5.5 5.0 4.5 4.0 3.5 3.0 2.5 2.0 1.5 1.0 0.5 0.0
f1 (ppm)

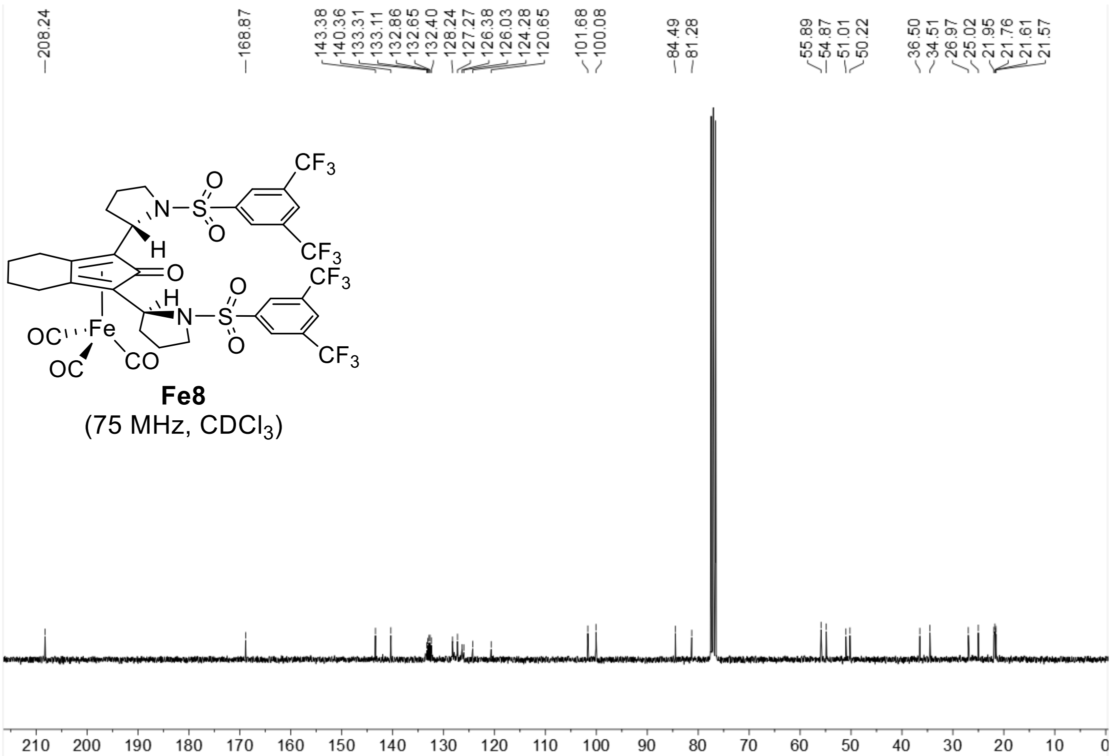
208.24 168.87 143.38 140.36 133.31 133.11 132.86 132.65 132.40 128.24 127.27 126.38 126.03 124.28 120.65 101.68 100.08 84.49 81.28 55.89 54.87 51.01 50.22 36.50 34.51 26.97 25.02 21.95 21.76 21.61 21.57
Fe8
(75 MHz, CDCl3)
210 200 190 180 170 160 150 140 130 120 110 100 90 80 70 60 50 40 30 20 10 0
f1 (ppm)

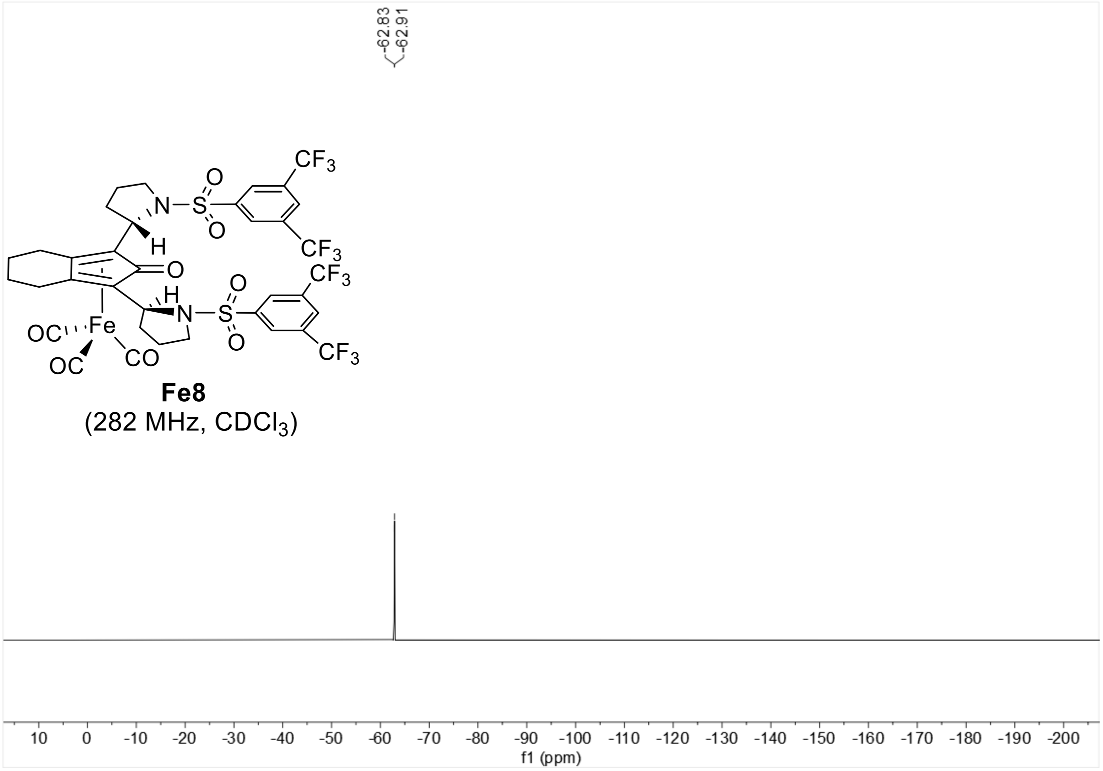

-62.83
-62.91
CF3
O
N
S
O
H
CF3
O
CF3
H
O
N
S
O
OC
Fe
OC
CO
CF3
Fe8
(282 MHz, CDCl3)
10 0 -10 -20 -30 -40 -50 -60 -70 -80 -90 -100 -110 -120 -130 -140 -150 -160 -170 -180 -190 -200
f1 (ppm)

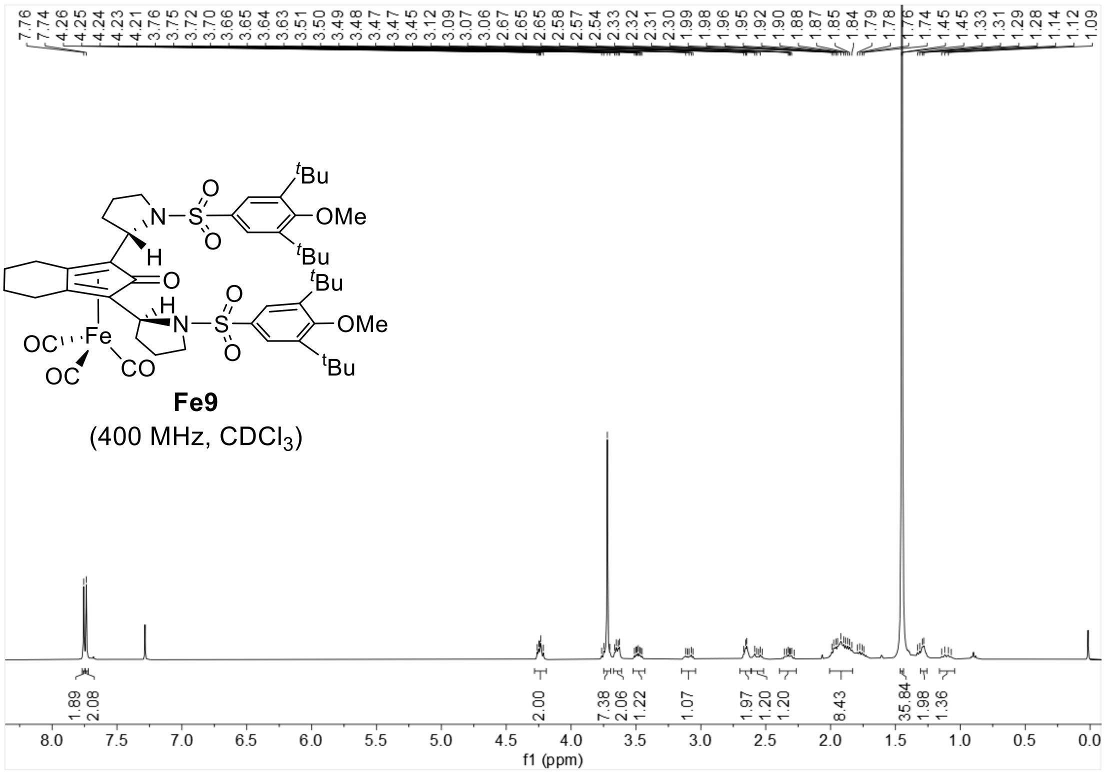
7.76
7.74
4.26
4.25
4.24
4.23
4.21
3.76
3.75
3.72
3.70
3.66
3.65
3.64
3.63
3.51
3.50
3.49
3.48
3.47
3.47
3.45
3.12
3.09
3.07
3.06
2.67
2.65
2.58
2.57
2.54
2.33
2.32
2.31
2.30
1.99
1.98
1.96
1.95
1.92
1.90
1.88
1.87
1.85
1.84
1.79
1.78
1.76
1.74
1.45
1.45
1.33
1.31
1.29
1.28
1.14
1.12
1.09
Fe9
(400 MHz, $CDCl_3$)
1.89
2.08
2.00
7.38
2.06
1.22
1.07
1.97
1.20
1.20
8.43
35.84
1.98
1.36
f1 (ppm)

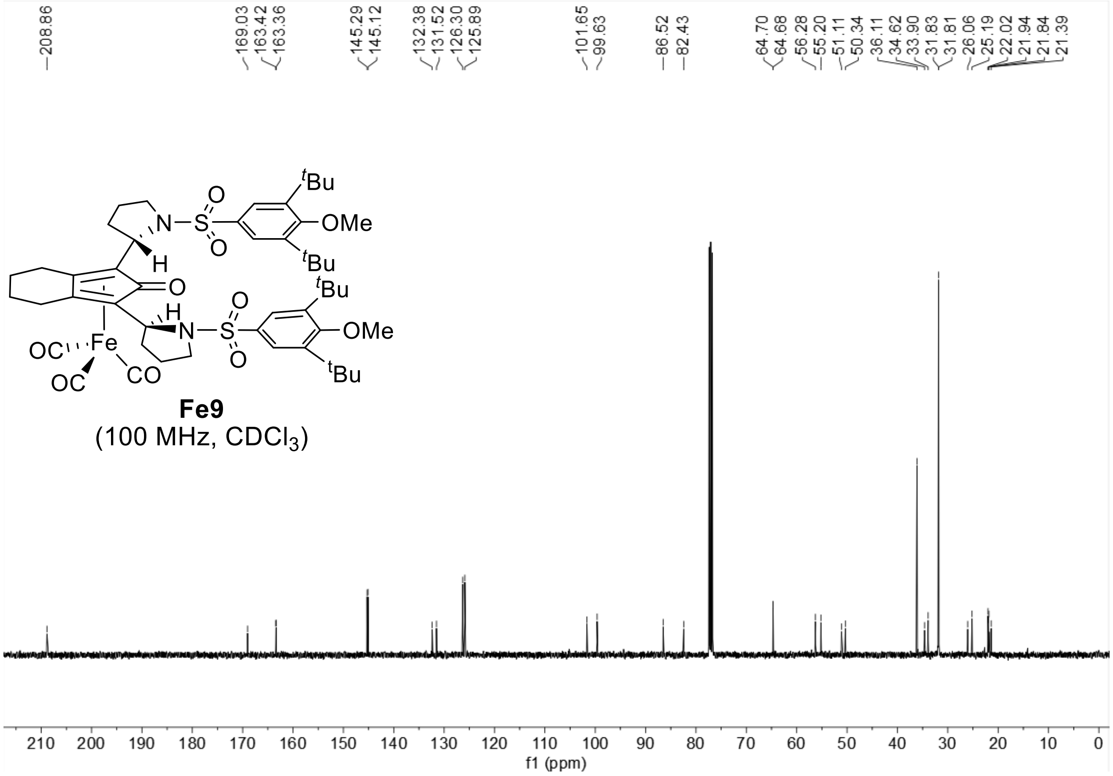
208.86
169.03
163.42
163.36
145.29
145.12
132.38
131.52
126.30
125.89
101.65
99.63
86.52
82.43
64.70
64.68
56.28
55.20
51.11
50.34
36.11
34.62
33.90
31.83
31.81
26.06
25.19
22.02
21.94
21.84
21.39
Fe9
(100 MHz, $CDCl_3$)
f1 (ppm)

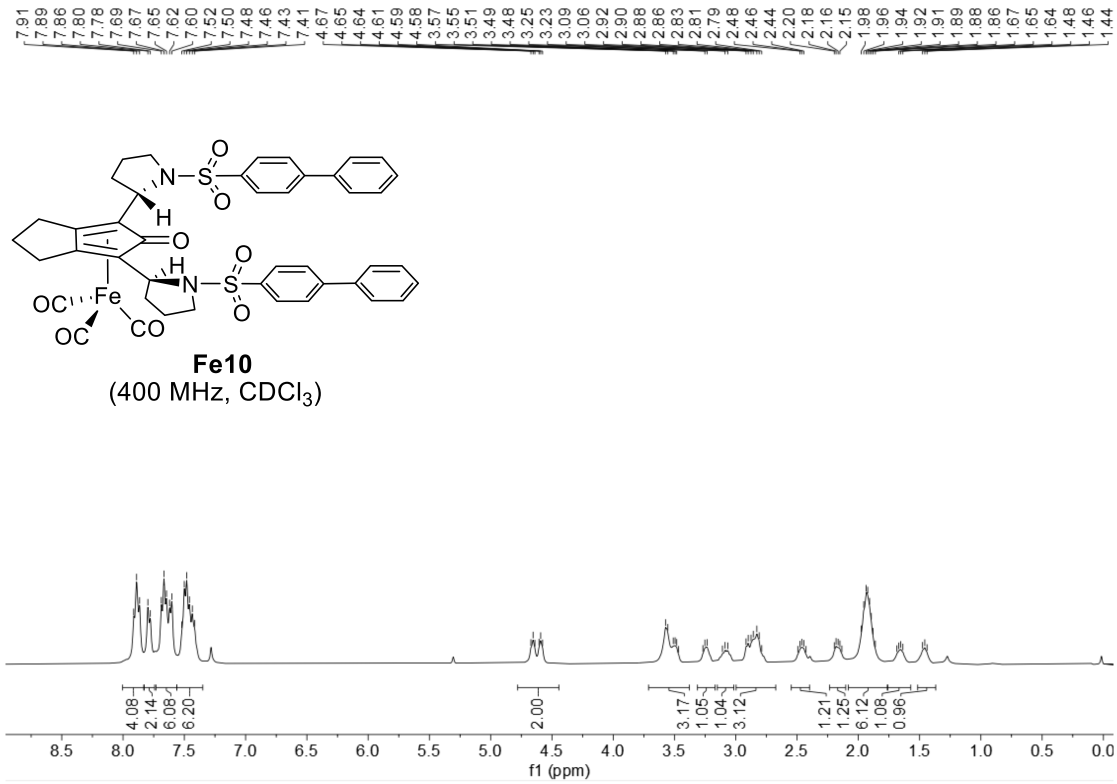
7.91 7.89 7.86 7.80 7.78 7.69 7.67 7.65 7.62 7.60 7.52 7.50 7.48 7.46 7.43 7.41 4.67 4.64 4.61 4.59 4.58 3.57 3.55 3.51 3.49 3.48 3.25 3.23 3.09 3.06 2.92 2.90 2.88 2.86 2.83 2.81 2.79 2.48 2.46 2.44 2.20 2.18 2.16 2.15 1.98 1.96 1.94 1.92 1.91 1.89 1.88 1.86 1.67 1.65 1.64 1.48 1.46 1.44
Fe10
(400 MHz, $CDCl_3$)
4.08 2.14 6.08 6.20 2.00 3.17 1.05 1.04 3.12 1.21 1.25 6.12 1.08 0.96
f1 (ppm)

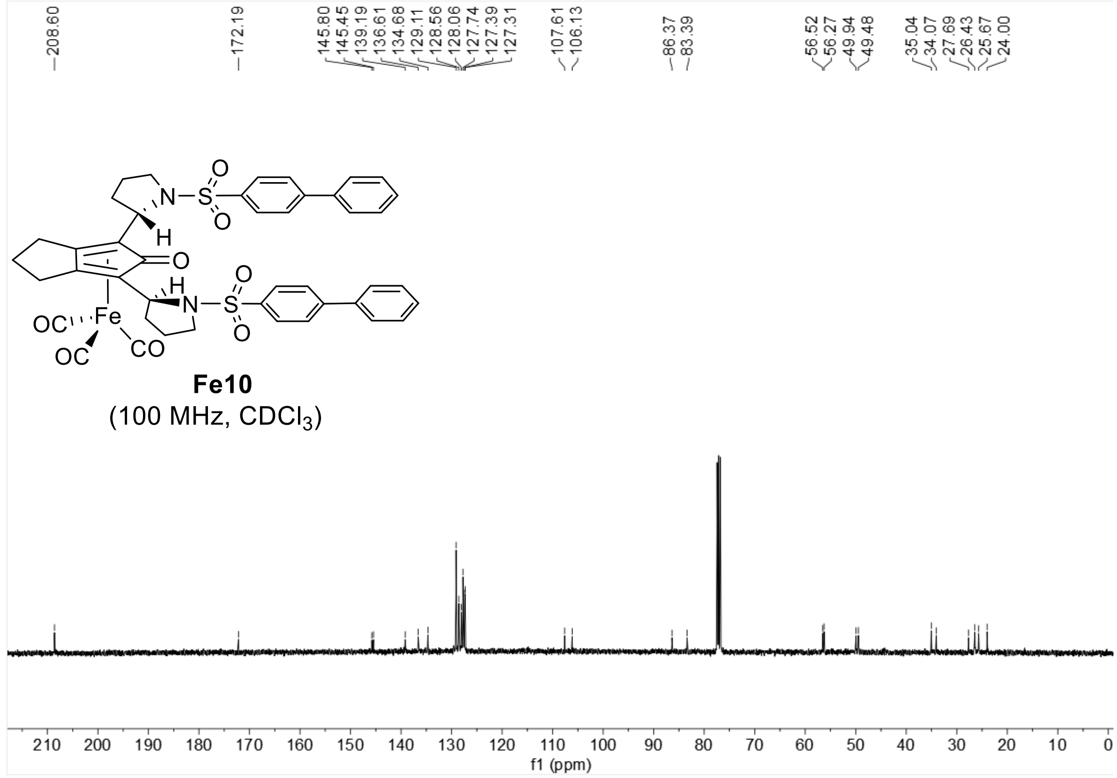
208.60 172.19 145.80 145.45 139.19 136.61 134.68 129.11 128.56 128.06 127.74 127.39 127.31 107.61 106.13 86.37 83.39 56.52 56.27 49.94 49.48 35.04 34.07 27.69 26.43 25.67 24.00
Fe10
(100 MHz, $CDCl_3$)
f1 (ppm)

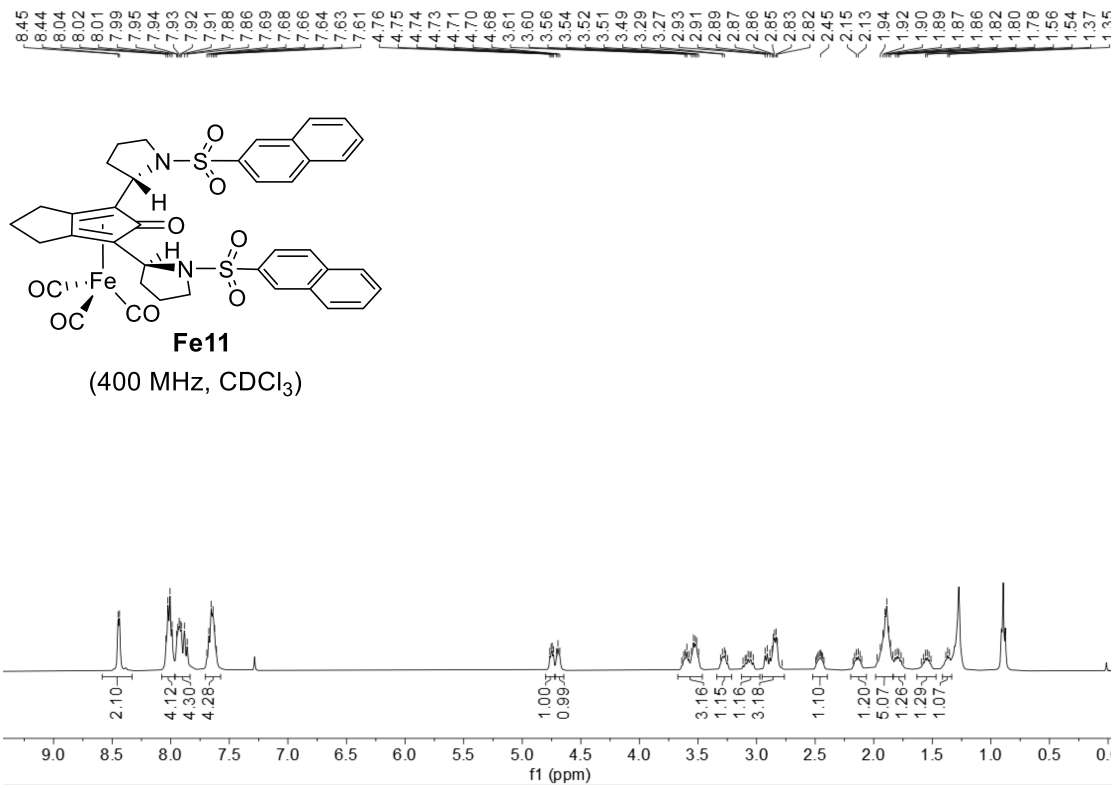
8.45 8.44 8.04 8.02 8.01 7.99 7.95 7.94 7.93 7.92 7.91 7.88 7.86 7.69 7.68 7.66 7.64 7.63 7.61 4.76 4.75 4.74 4.73 4.71 4.70 4.68 3.61 3.60 3.56 3.54 3.52 3.51 3.49 3.29 3.27 2.93 2.91 2.89 2.87 2.86 2.85 2.83 2.82 2.45 2.15 2.13 1.94 1.92 1.90 1.89 1.87 1.86 1.82 1.80 1.78 1.56 1.54 1.37
Fe11
(400 MHz, $CDCl_3$)
2.10 4.12 4.30 4.28 1.00 0.99 3.16 1.15 1.16 3.18 1.10 1.20 5.07 1.26 1.29 1.07
9.0 8.5 8.0 7.5 7.0 6.5 6.0 5.5 5.0 4.5 4.0 3.5 3.0 2.5 2.0 1.5 1.0 0.5
f1 (ppm)

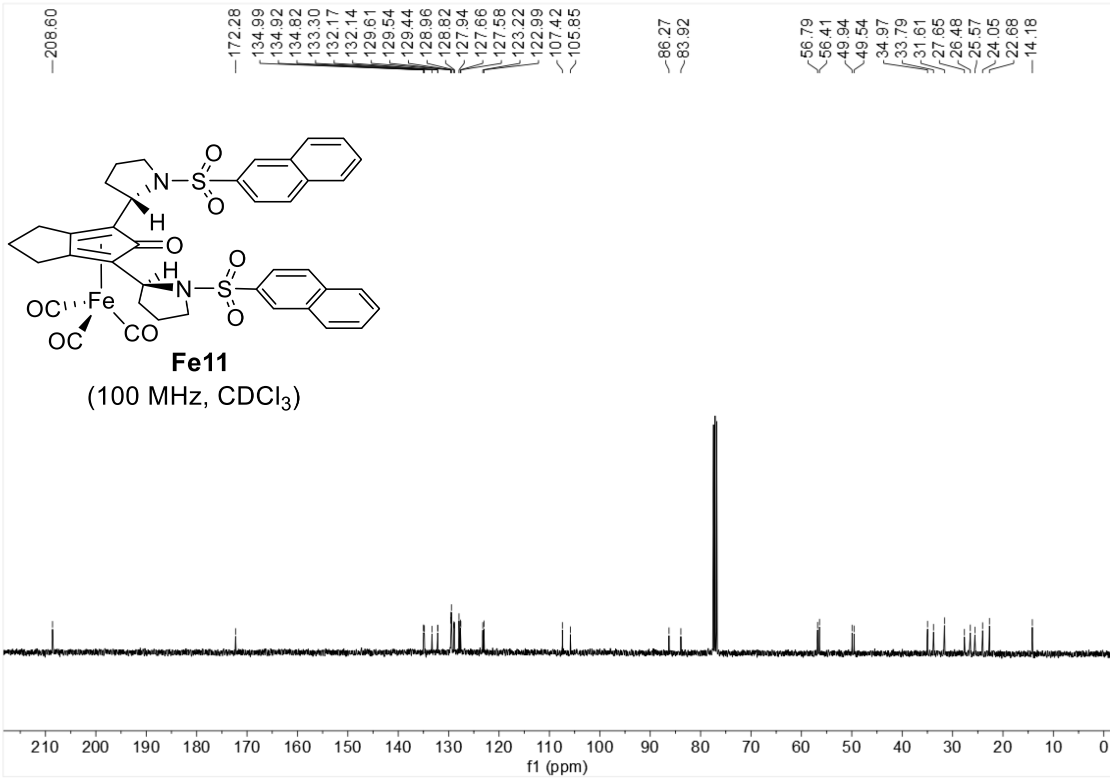
208.60 172.28 134.99 134.92 134.82 133.30 132.17 132.14 129.61 129.54 129.44 128.96 128.82 127.94 127.66 127.58 123.22 122.99 107.42 105.85 86.27 83.92 56.79 56.41 49.94 49.54 34.97 33.79 31.61 27.65 26.48 25.57 24.05 22.68 14.18
Fe11
(100 MHz, $CDCl_3$)
210 200 190 180 170 160 150 140 130 120 110 100 90 80 70 60 50 40 30 20 10 0
f1 (ppm)

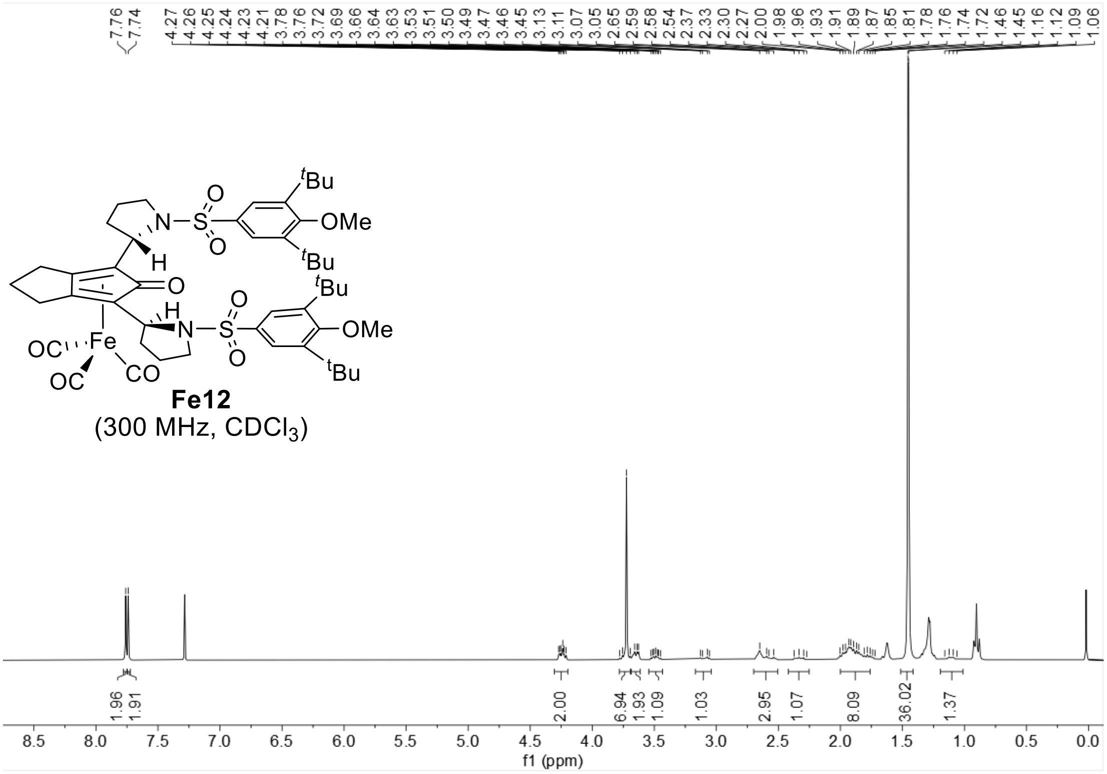
7.76
7.74
4.27
4.26
4.25
4.24
4.23
4.21
3.78
3.76
3.72
3.69
3.66
3.64
3.63
3.53
3.51
3.50
3.49
3.47
3.46
3.45
3.13
3.11
3.07
3.05
2.65
2.59
2.58
2.54
2.37
2.33
2.30
2.27
2.00
1.98
1.96
1.93
1.91
1.89
1.87
1.85
1.81
1.78
1.76
1.74
1.72
1.46
1.45
1.16
1.12
1.09
1.06
tBu
OMe
tBu
tBu
OMe
tBu
Fe12
(300 MHz, CDCl3)
1.96
1.91
2.00
6.94
1.93
1.09
1.03
2.95
1.07
8.09
36.02
1.37
8.5 8.0 7.5 7.0 6.5 6.0 5.5 5.0 4.5 4.0 3.5 3.0 2.5 2.0 1.5 1.0 0.5 0.0
f1 (ppm)

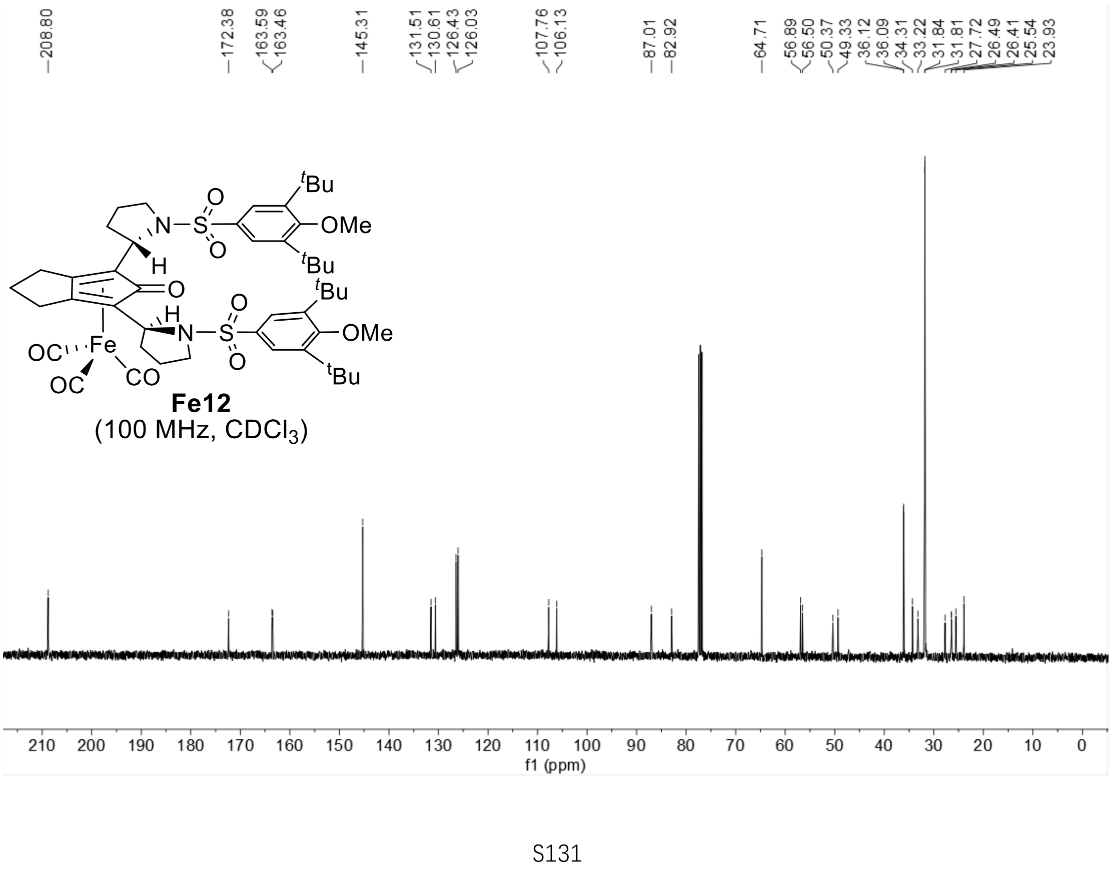
208.80
172.38
163.59
163.46
145.31
131.51
130.61
126.43
126.03
107.76
106.13
87.01
82.92
64.71
56.89
56.50
50.37
49.33
36.12
36.09
34.31
33.22
31.84
31.81
27.72
26.49
26.41
25.54
23.93
tBu
OMe
tBu
tBu
OMe
tBu
Fe12
(100 MHz, CDCl3)
210 200 190 180 170 160 150 140 130 120 110 100 90 80 70 60 50 40 30 20 10 0
f1 (ppm)

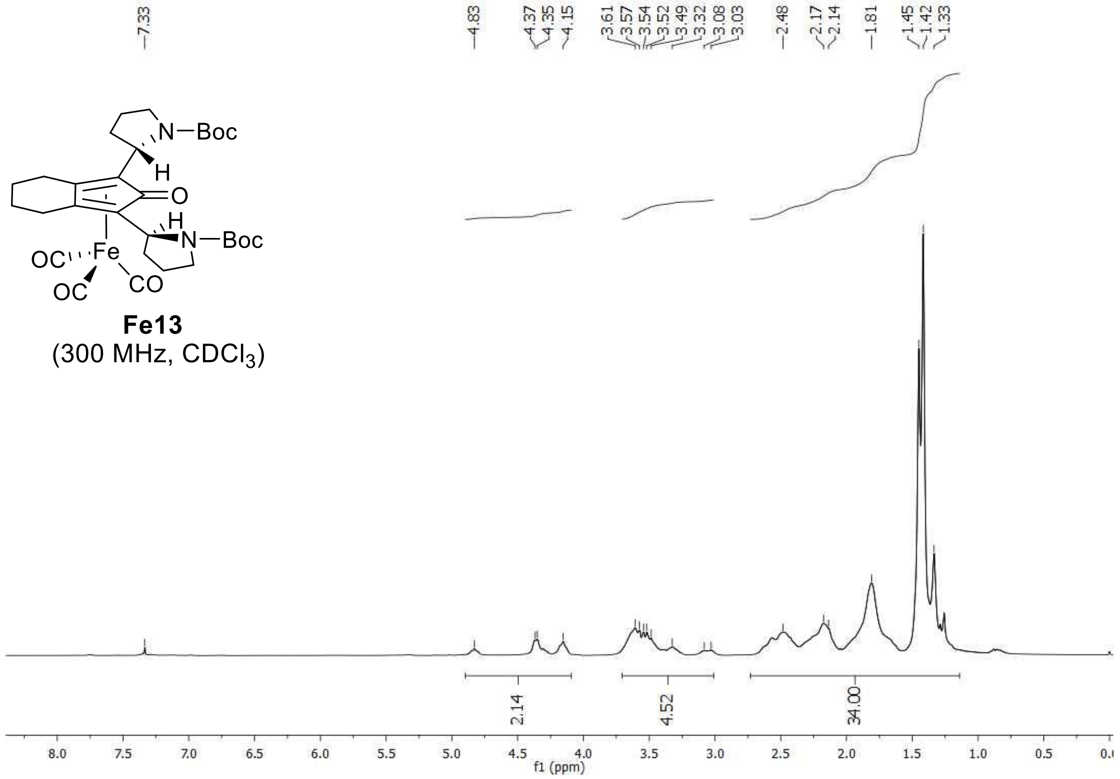

Fe13
(300 MHz, $CDCl_3$)

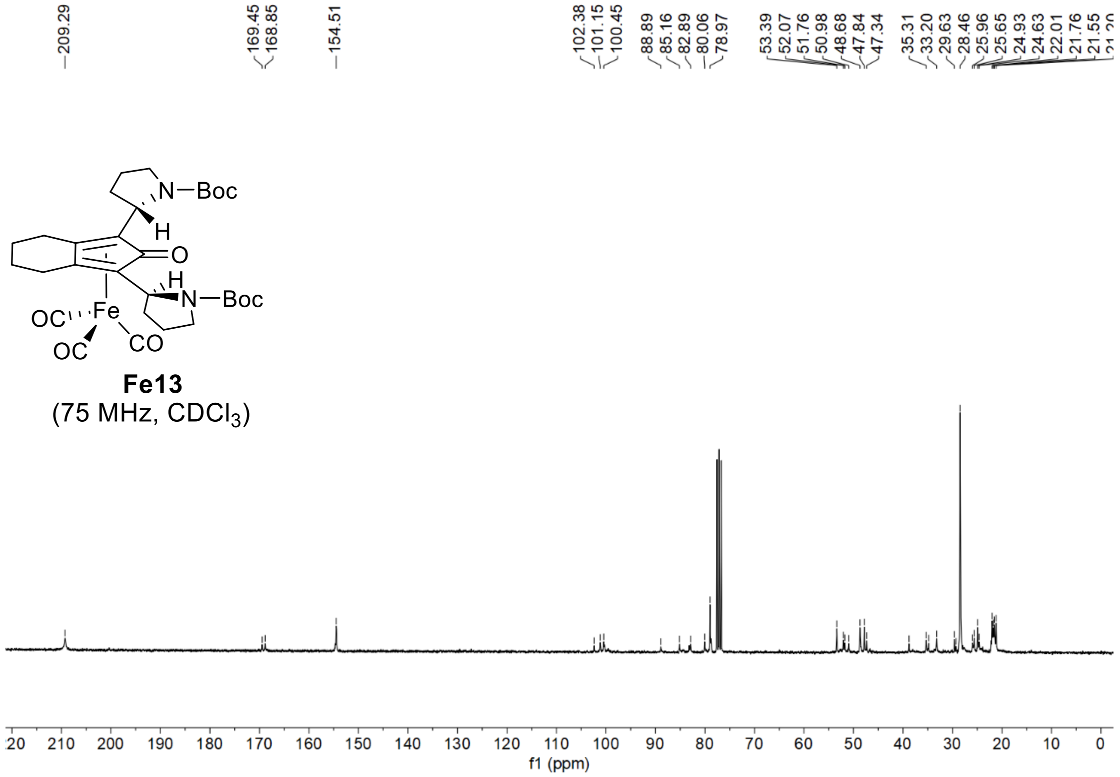

Fe13
(75 MHz, $CDCl_3$)

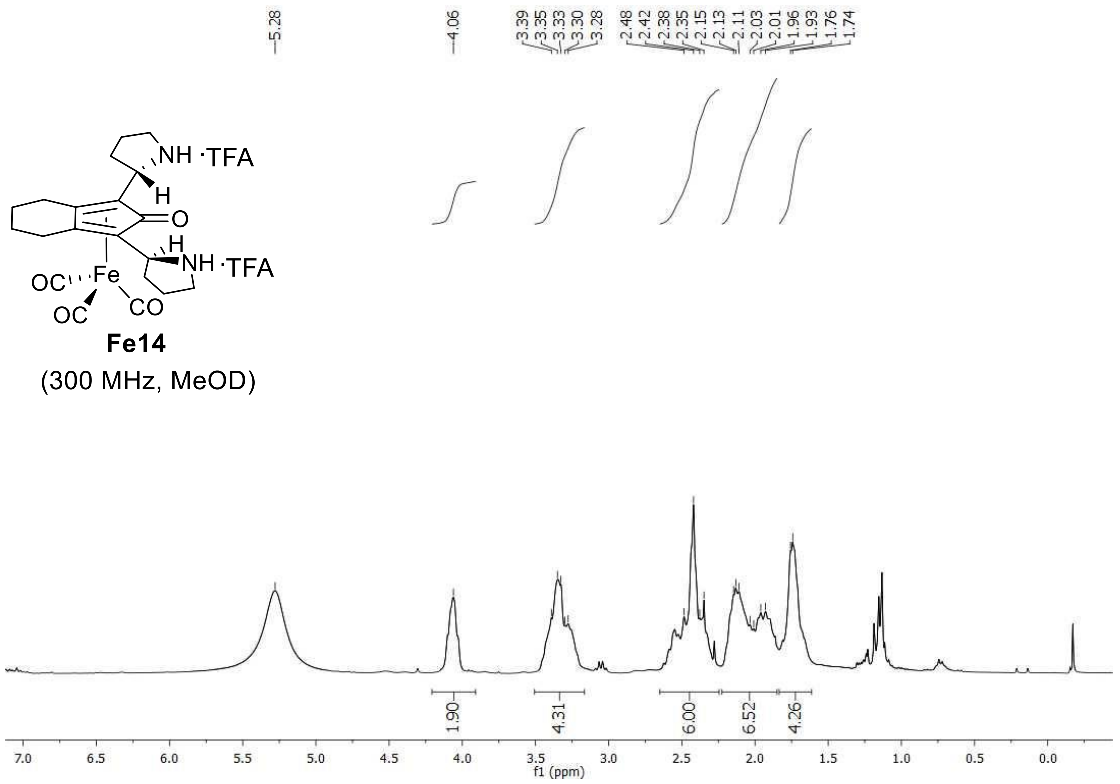
5.28
4.06
3.39 3.35 3.33 3.30 3.28
2.48 2.42 2.38 2.35 2.15 2.13 2.11 2.03 2.01 1.96 1.93 1.76 1.74
NH ·TFA
H
O
H
NH ·TFA
OC
Fe
OC
CO
Fe14
(300 MHz, MeOD)
1.90
4.31
6.00
6.52
4.26
7.0 6.5 6.0 5.5 5.0 4.5 4.0 3.5 3.0 2.5 2.0 1.5 1.0 0.5 0.0
f1 (ppm)

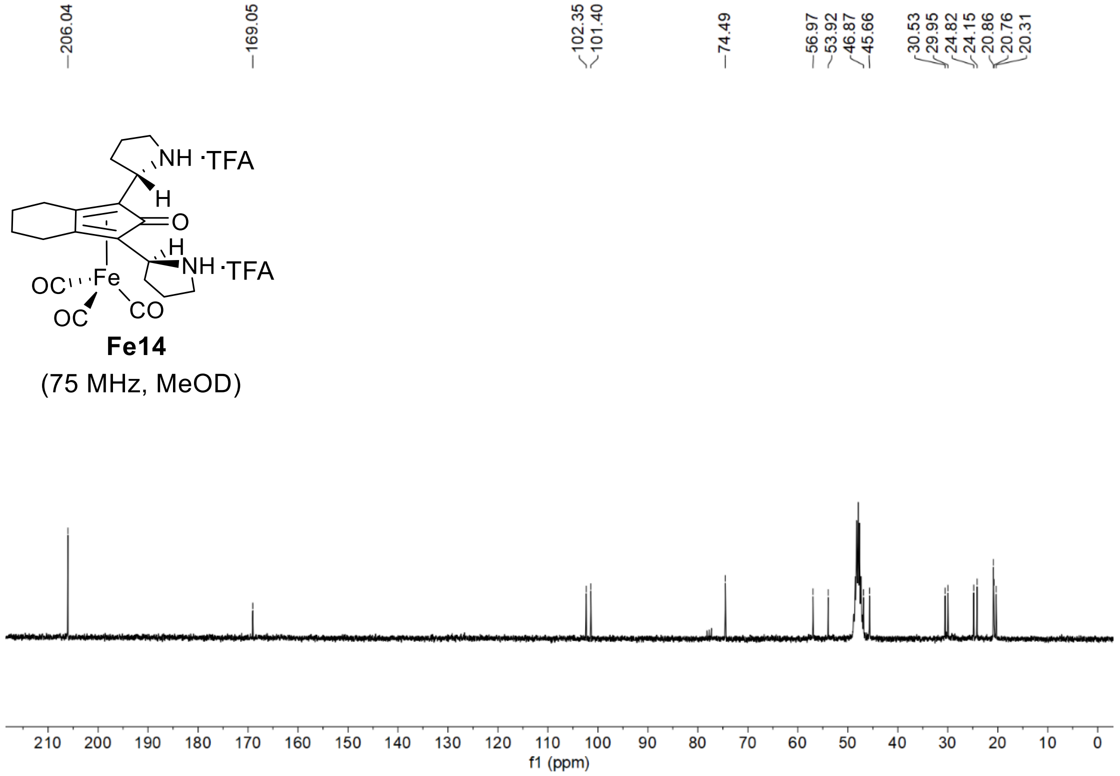
206.04
169.05
102.35 101.40
74.49
56.97 53.92 46.87 45.66
30.53 29.95 24.82 24.15 20.86 20.76 20.31
NH ·TFA
H
O
H
NH ·TFA
OC
Fe
OC
CO
Fe14
(75 MHz, MeOD)
210 200 190 180 170 160 150 140 130 120 110 100 90 80 70 60 50 40 30 20 10 0
f1 (ppm)

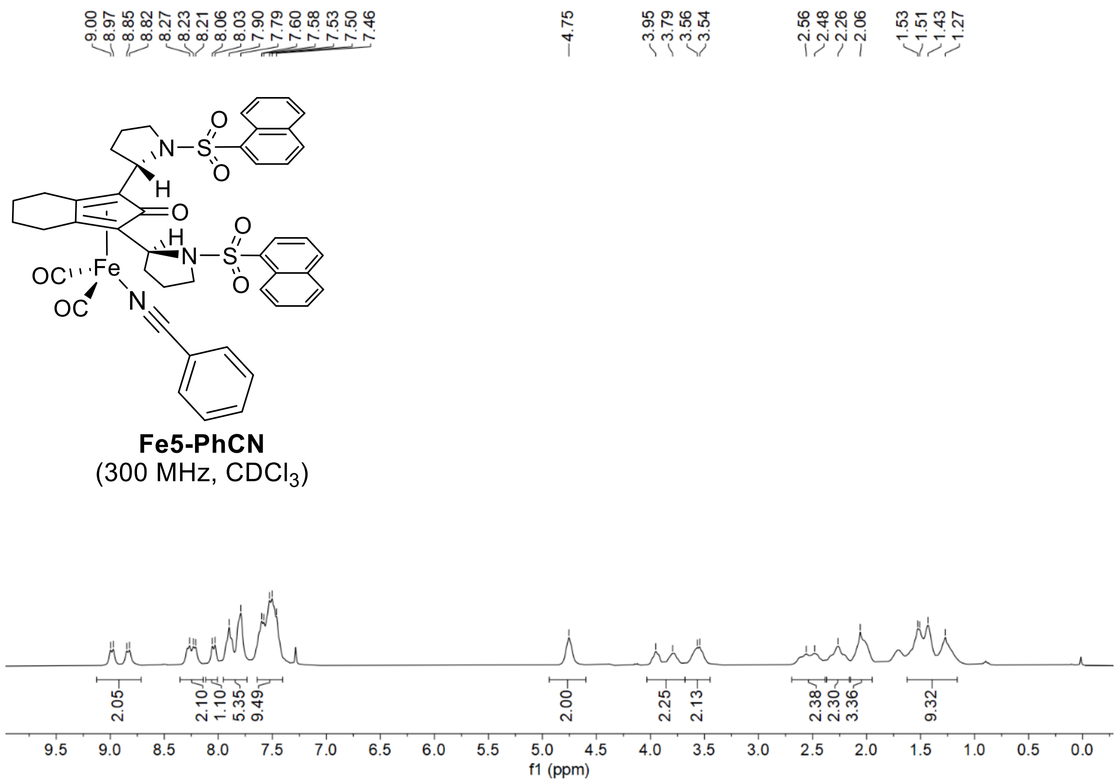
9.00
8.97
8.85
8.82
8.27
8.23
8.21
8.06
8.03
7.90
7.79
7.60
7.58
7.53
7.50
7.46
4.75
3.95
3.79
3.56
3.54
2.56
2.48
2.26
2.06
1.53
1.51
1.43
1.27
Fe5-PhCN
(300 MHz, $CDCl_3$)
2.05
2.10
1.10
5.35
9.49
2.00
2.25
2.13
2.38
2.30
3.36
9.32
f1 (ppm)

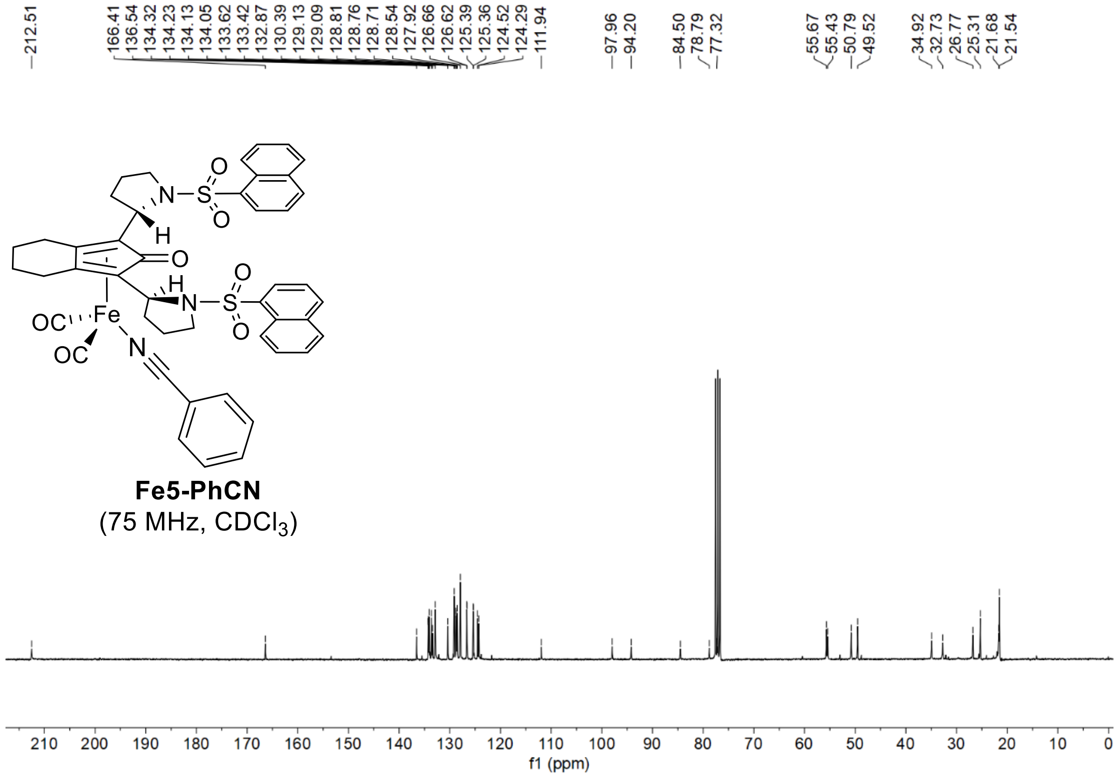
212.51
166.41
136.54
134.32
134.23
134.13
134.05
133.62
133.42
132.87
130.39
129.13
129.09
128.81
128.76
128.71
128.54
127.92
126.66
126.62
125.39
125.36
124.52
124.29
111.94
97.96
94.20
84.50
78.79
77.32
55.67
55.43
50.79
49.52
34.92
32.73
26.77
25.31
21.68
21.54
Fe5-PhCN
(75 MHz, $CDCl_3$)
f1 (ppm)

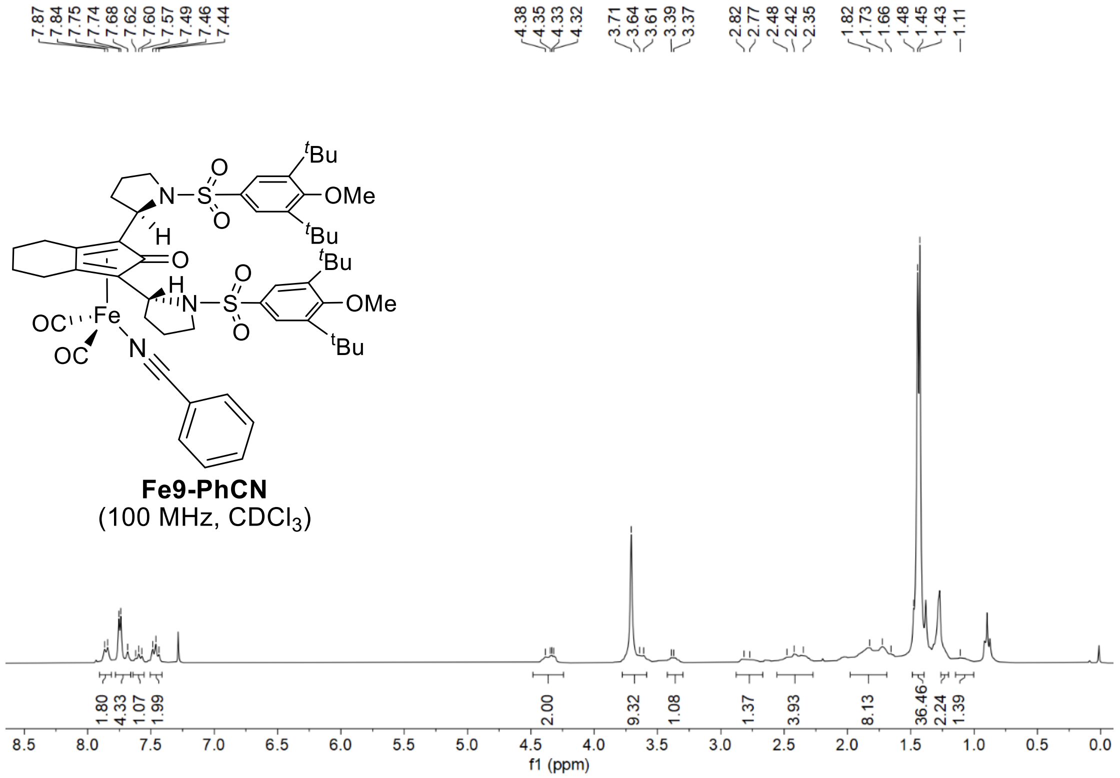
7.87 7.84 7.75 7.74 7.68 7.62 7.60 7.57 7.49 7.46 7.44
4.38 4.35 4.33 4.32 3.71 3.64 3.61 3.39 3.37 2.82 2.77 2.48 2.42 2.35 1.82 1.73 1.66 1.48 1.45 1.43 1.11
Fe9-PhCN
(100 MHz, CDCl3)
1.80 4.33 1.07 1.99 2.00 9.32 1.08 1.37 3.93 8.13 36.46 2.24 1.39
f1 (ppm)

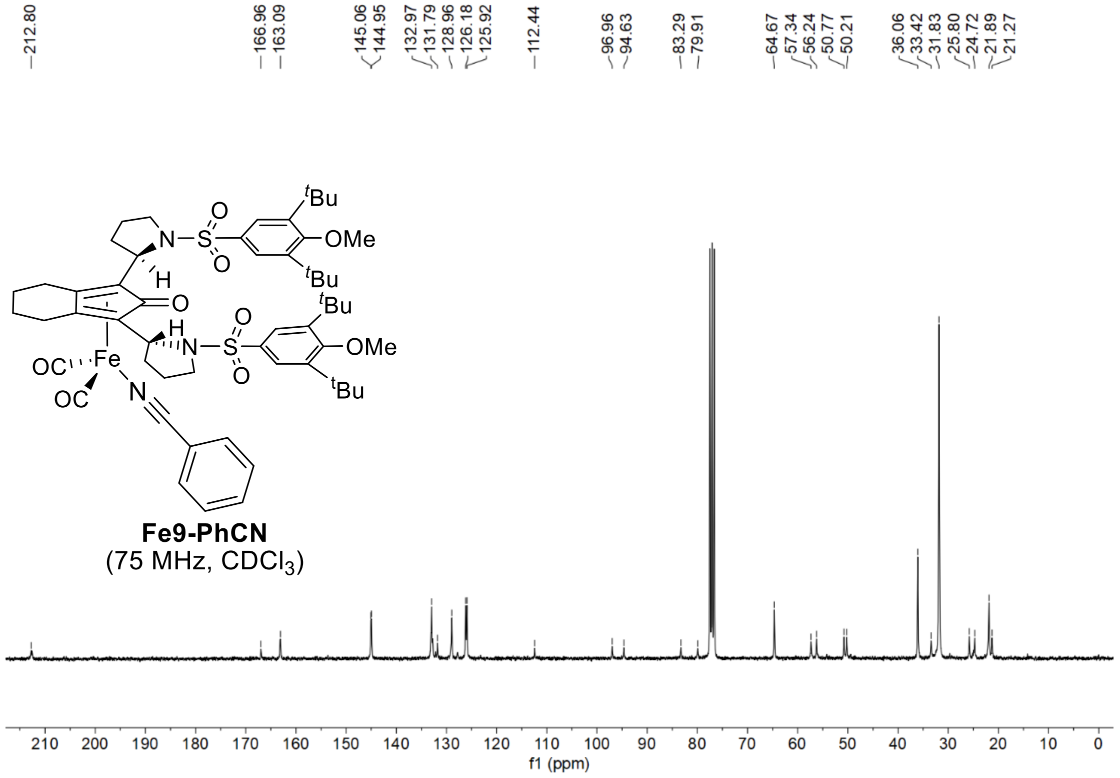
212.80 166.96 163.09 145.06 144.95 132.97 131.79 128.96 126.18 125.92 112.44 96.96 94.63 83.29 79.91 64.67 57.34 56.24 50.77 50.21 36.06 33.42 31.83 25.80 24.72 21.89 21.27
Fe9-PhCN
(75 MHz, CDCl3)
f1 (ppm)

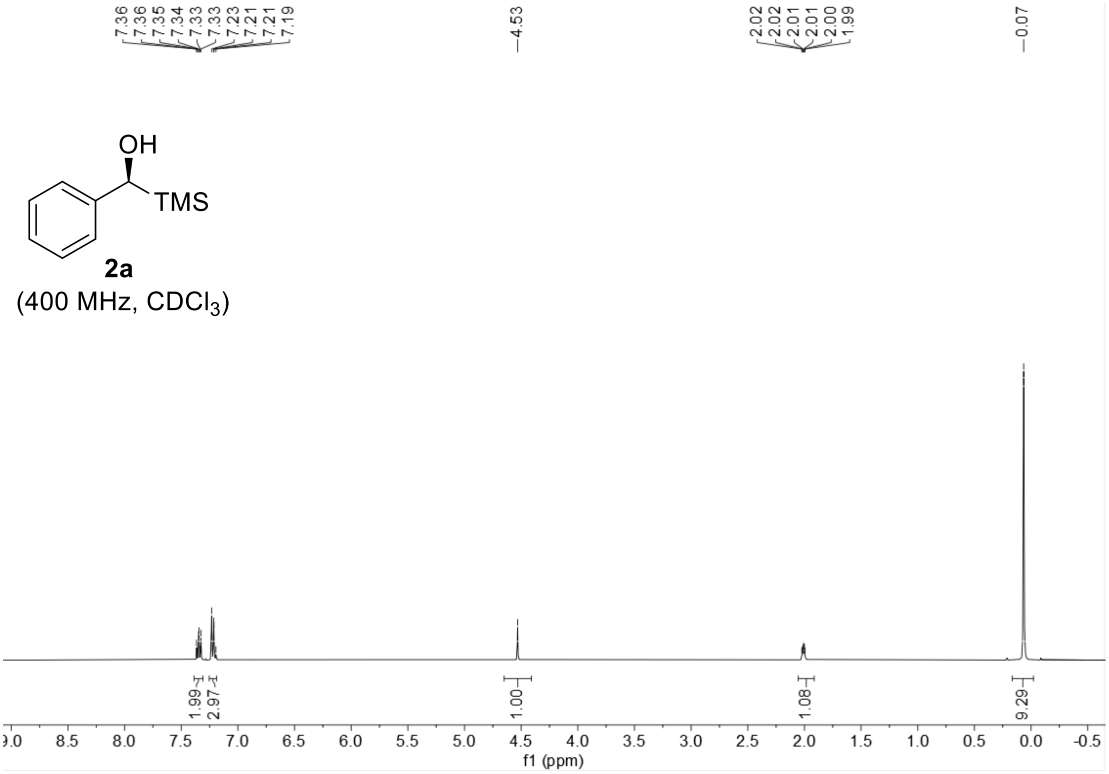
7.36
7.36
7.35
7.34
7.33
7.33
7.23
7.21
7.21
7.19
4.53
2.02
2.02
2.01
2.01
2.00
1.99
0.07
OH
TMS
2a
(400 MHz, CDCl3)
1.99
2.97
1.00
1.08
9.29
f1 (ppm)

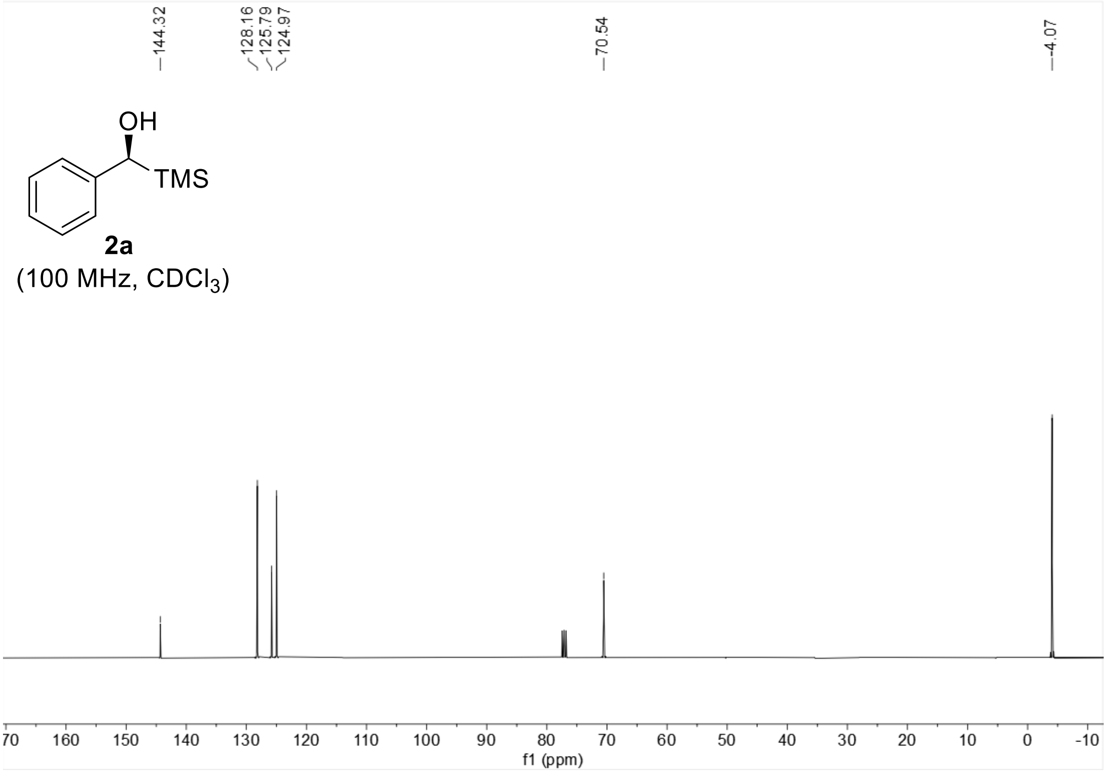
144.32
128.16
125.79
124.97
70.54
-4.07
OH
TMS
2a
(100 MHz, CDCl3)
f1 (ppm)

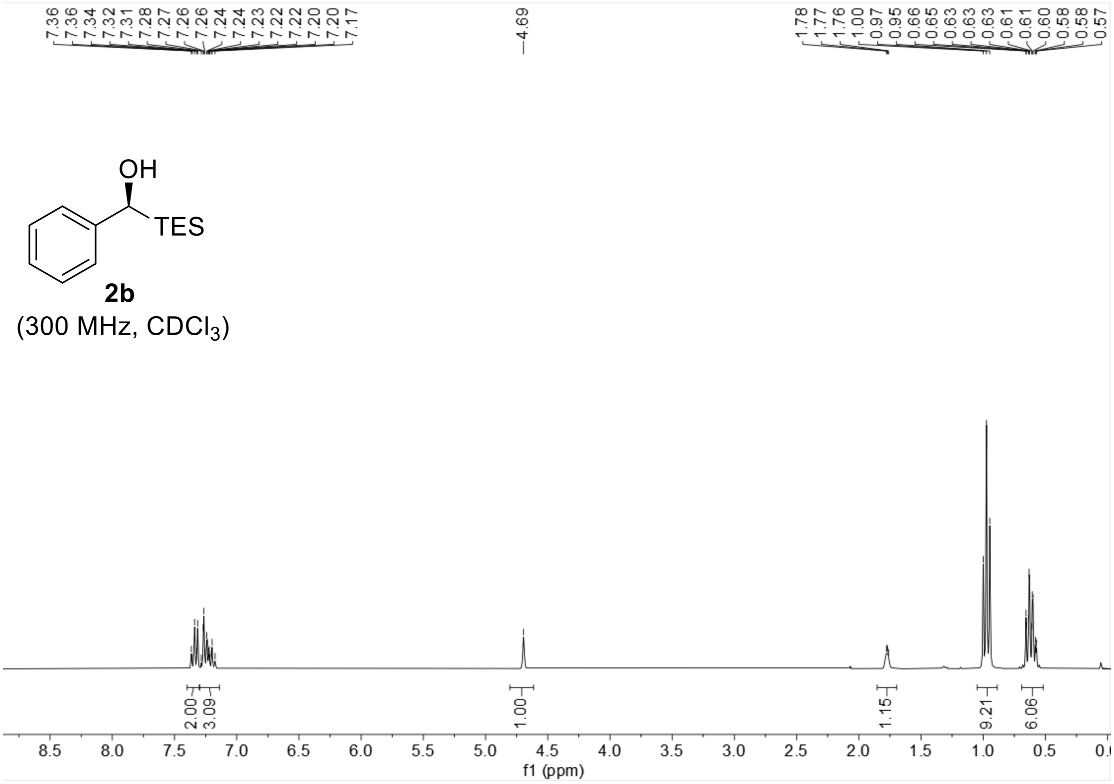
7.36
7.36
7.34
7.32
7.31
7.28
7.27
7.26
7.26
7.24
7.24
7.23
7.22
7.22
7.20
7.17
4.69
1.78
1.77
1.76
1.00
0.97
0.95
0.66
0.65
0.63
0.63
0.63
0.61
0.61
0.60
0.58
0.58
0.57
OH
TES
2b
(300 MHz, CDCl3)
2.00
3.09
1.00
1.15
9.21
6.06
f1 (ppm)

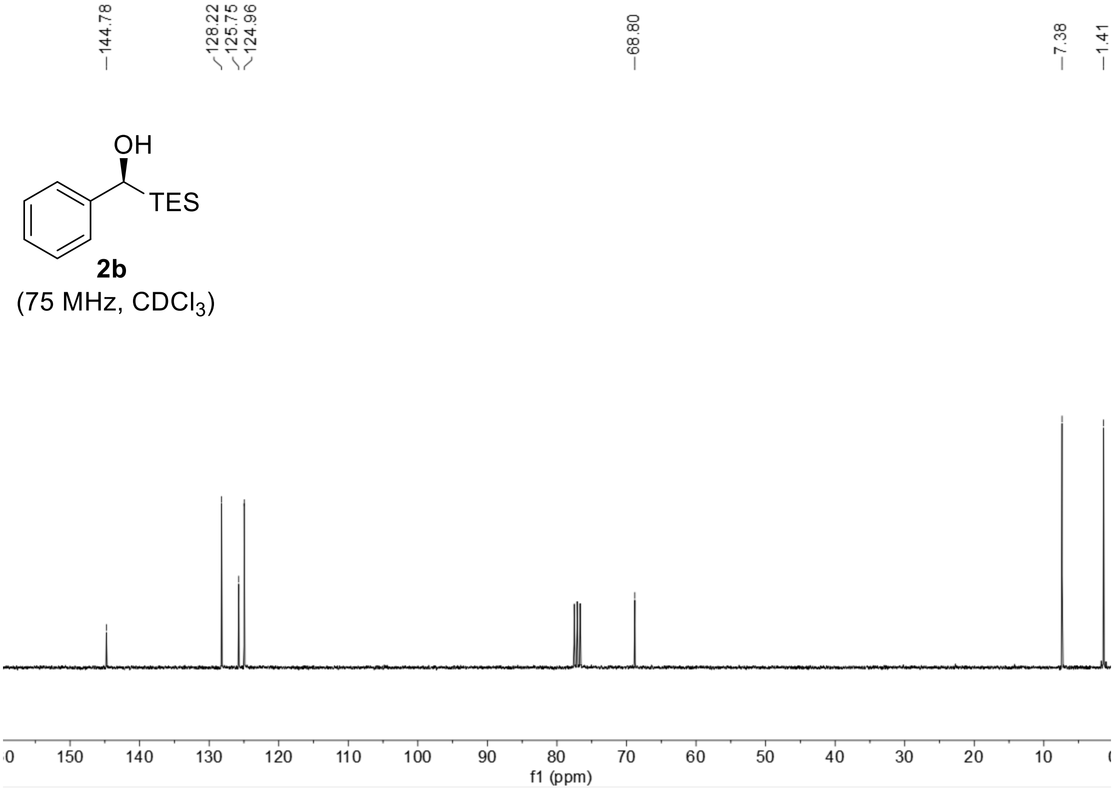
144.78
128.22
125.75
124.96
68.80
7.38
1.41
OH
TES
2b
(75 MHz, CDCl3)
f1 (ppm)

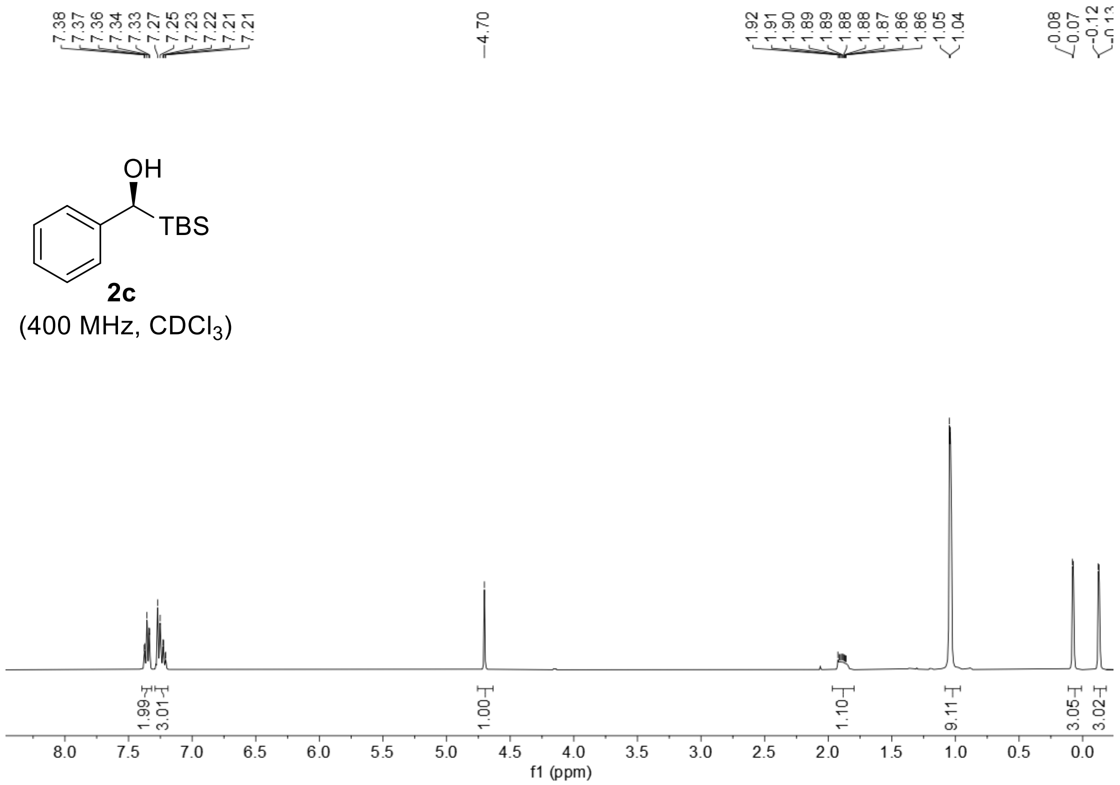

2c
(400 MHz, $CDCl_3$)

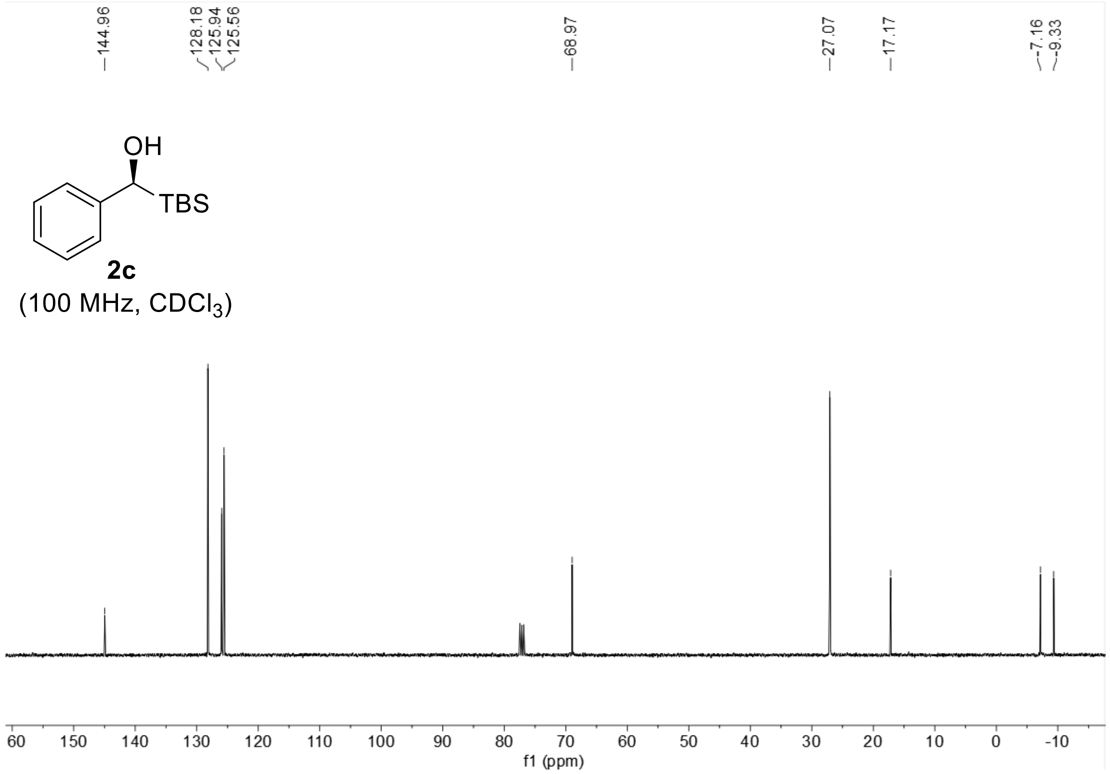

2c
(100 MHz, $CDCl_3$)

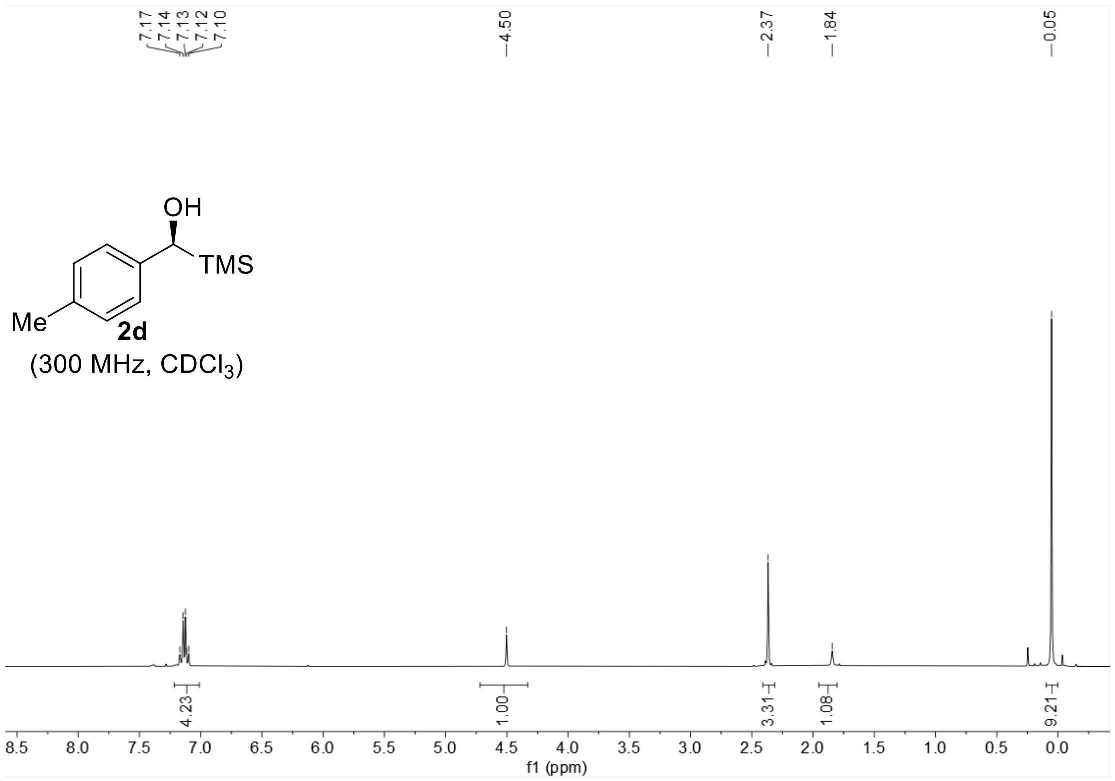
7.17
7.14
7.13
7.12
7.10
4.50
2.37
1.84
0.05
OH
TMS
Me
2d
(300 MHz, CDCl3)
4.23
1.00
3.31
1.08
9.21
8.5 8.0 7.5 7.0 6.5 6.0 5.5 5.0 4.5 4.0 3.5 3.0 2.5 2.0 1.5 1.0 0.5 0.0
f1 (ppm)

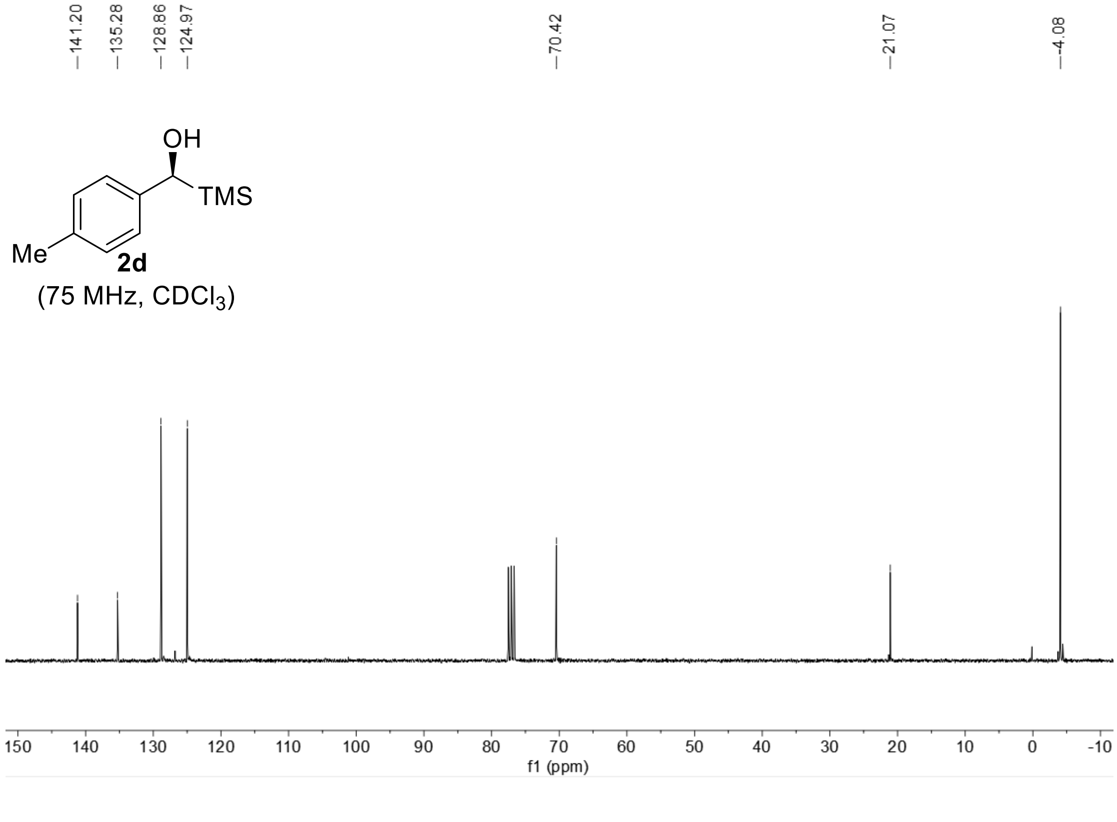
141.20
135.28
128.86
124.97
70.42
21.07
-4.08
OH
TMS
Me
2d
(75 MHz, CDCl3)
150 140 130 120 110 100 90 80 70 60 50 40 30 20 10 0 -10
f1 (ppm)

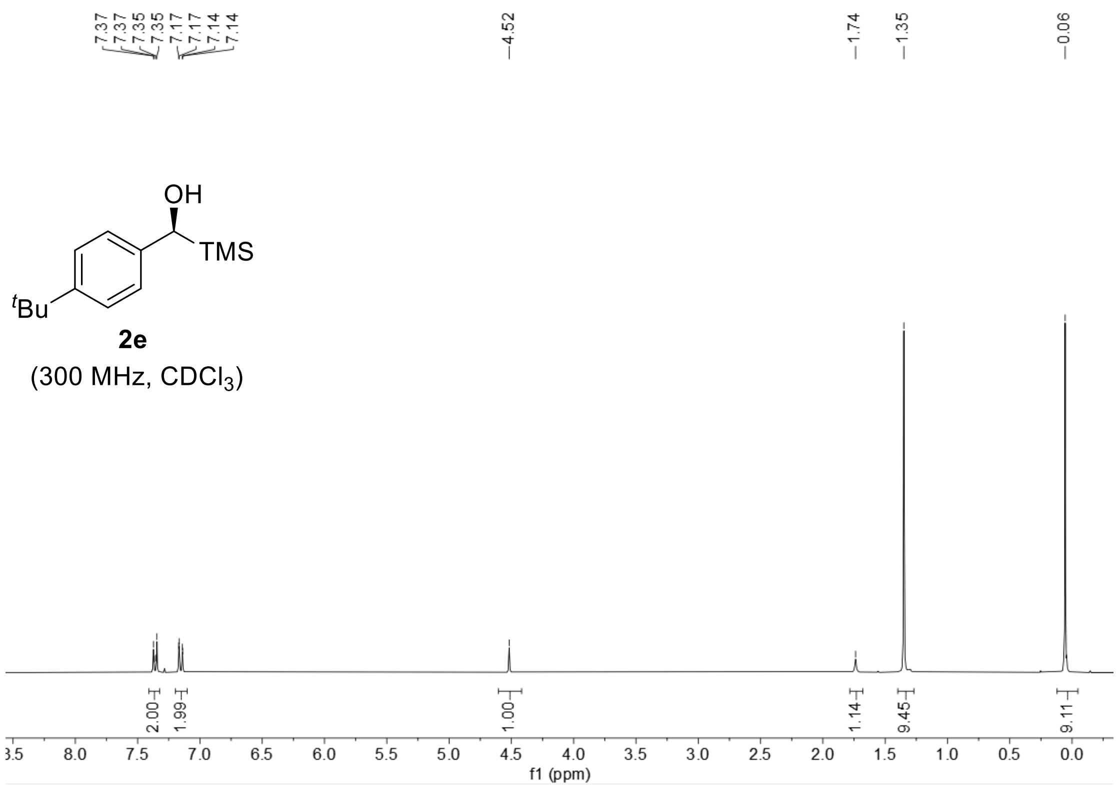
7.37
7.37
7.35
7.35
7.17
7.17
7.14
7.14
4.52
1.74
1.35
0.06
OH
TMS
tBu
2e
(300 MHz, CDCl3)
2.00
1.99
1.00
1.14
9.45
9.11
f1 (ppm)

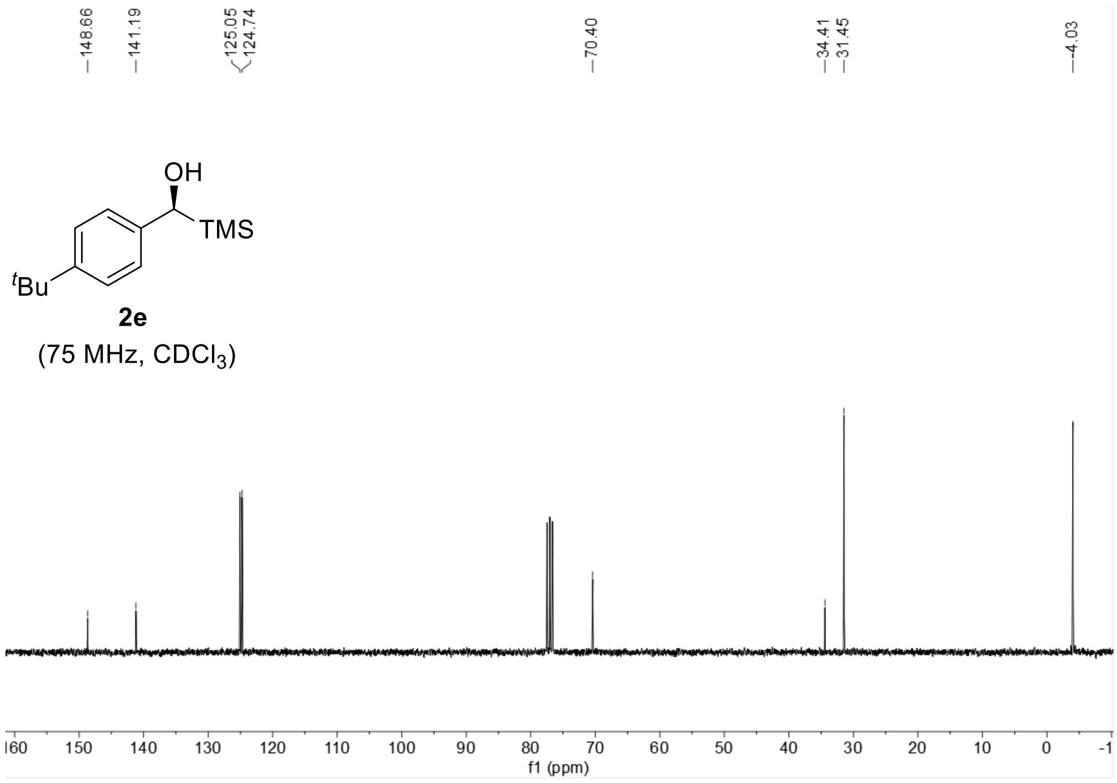
148.66
141.19
125.05
124.74
70.40
34.41
31.45
-4.03
OH
TMS
tBu
2e
(75 MHz, CDCl3)
f1 (ppm)

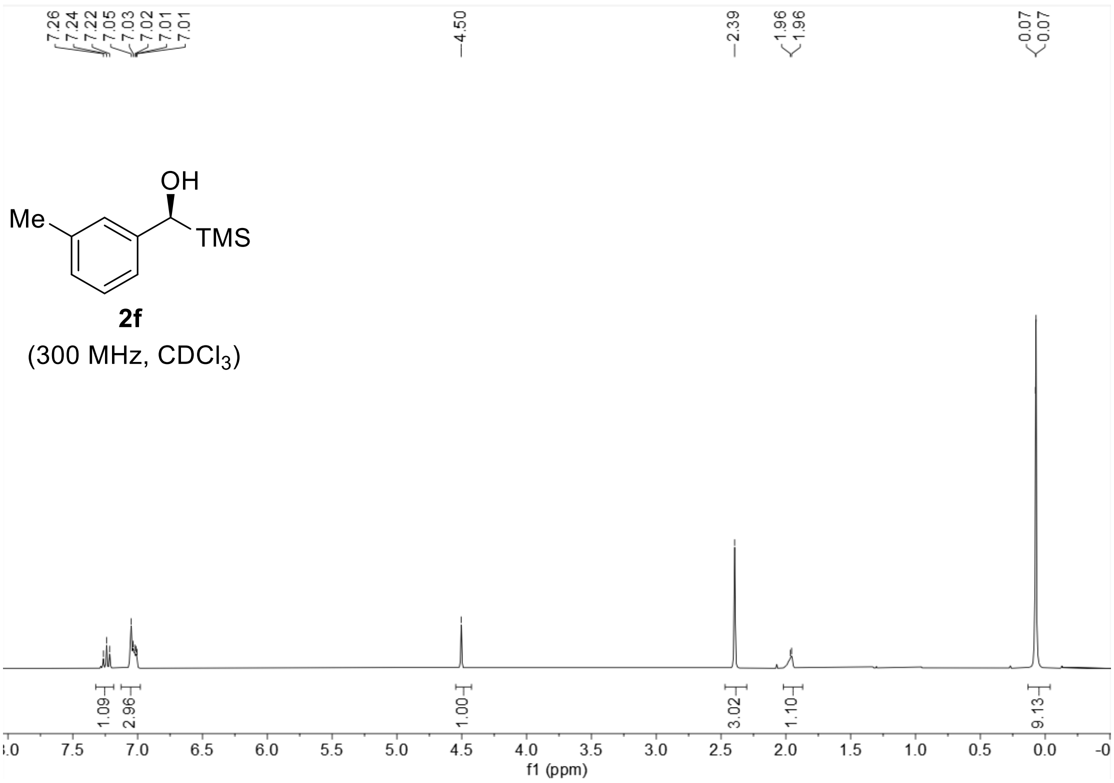

OH
Me
TMS
2f
(300 MHz, CDCl3)
7.26
7.24
7.22
7.05
7.03
7.02
7.01
7.01
4.50
2.39
1.96
1.96
0.07
0.07
1.09
2.96
1.00
3.02
1.10
9.13
f1 (ppm)

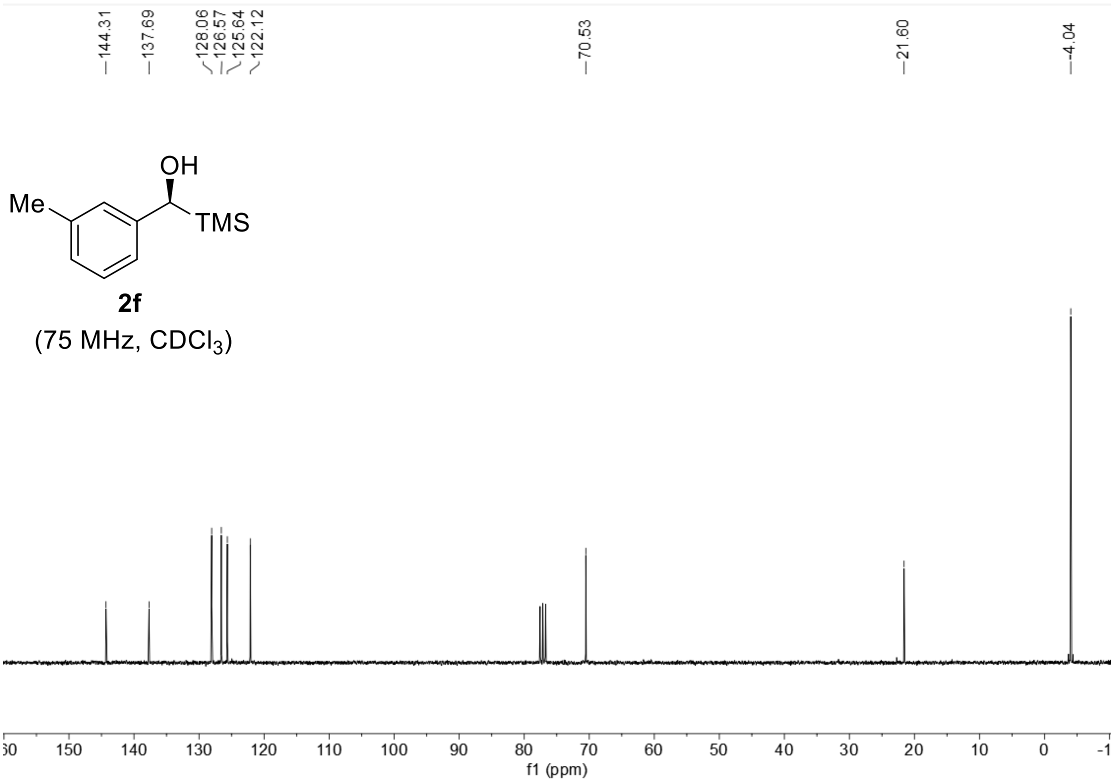

OH
Me
TMS
2f
(75 MHz, CDCl3)
144.31
137.69
128.06
126.57
125.64
122.12
70.53
21.60
-4.04
f1 (ppm)

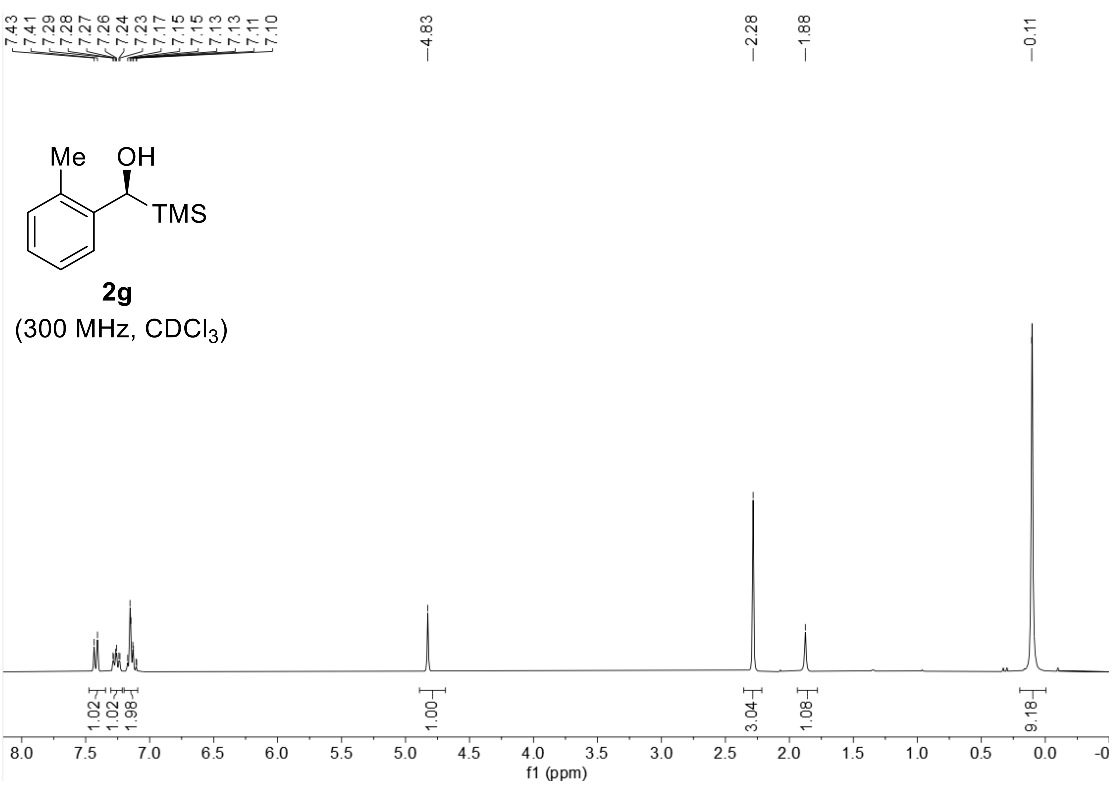
7.43
7.41
7.29
7.28
7.27
7.26
7.24
7.23
7.17
7.15
7.15
7.13
7.13
7.11
7.10
4.83
2.28
1.88
0.11
Me
OH
TMS
2g
(300 MHz, CDCl3)
1.02
1.02
1.98
1.00
3.04
1.08
9.18
8.0
7.5
7.0
6.5
6.0
5.5
5.0
4.5
4.0
3.5
3.0
2.5
2.0
1.5
1.0
0.5
0.0
-0
f1 (ppm)

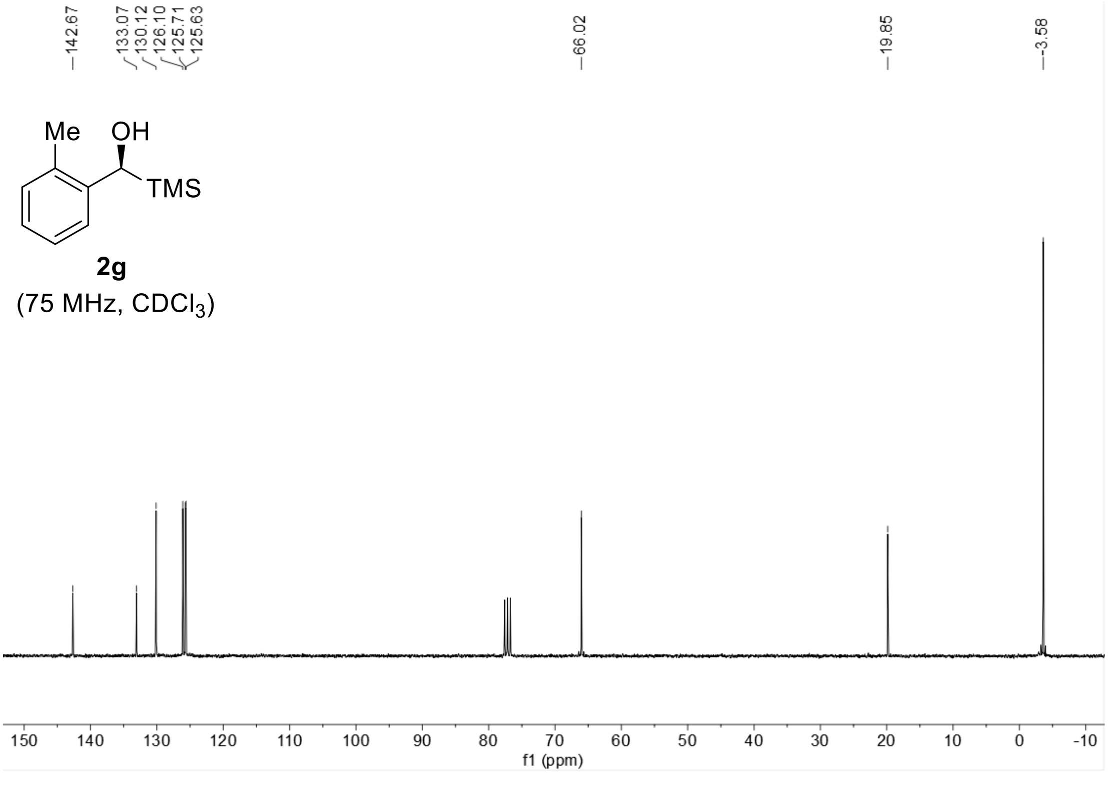
142.67
133.07
130.12
126.10
125.71
125.63
66.02
19.85
-3.58
Me
OH
TMS
2g
(75 MHz, CDCl3)
150
140
130
120
110
100
90
80
70
60
50
40
30
20
10
0
-10
f1 (ppm)

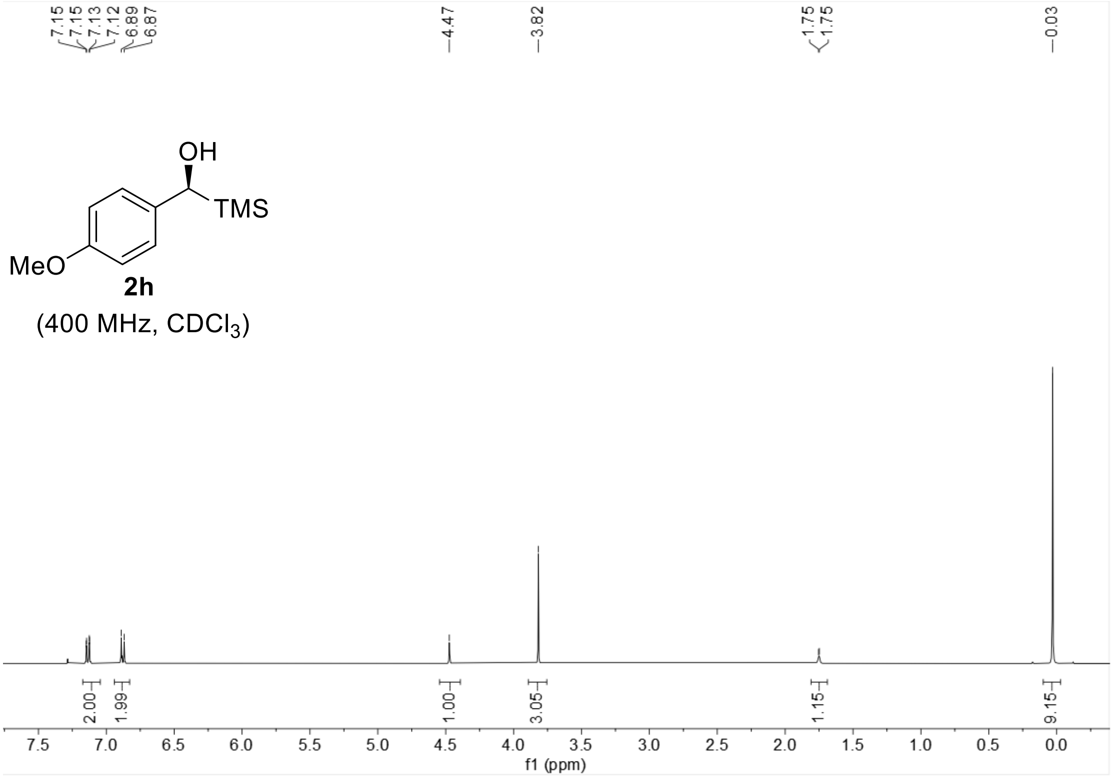
7.15
7.15
7.13
7.12
6.89
6.87
4.47
3.82
1.75
1.75
0.03
OH
TMS
MeO
2h
(400 MHz, CDCl3)
2.00
1.99
1.00
3.05
1.15
9.15
7.5 7.0 6.5 6.0 5.5 5.0 4.5 4.0 3.5 3.0 2.5 2.0 1.5 1.0 0.5 0.0
f1 (ppm)

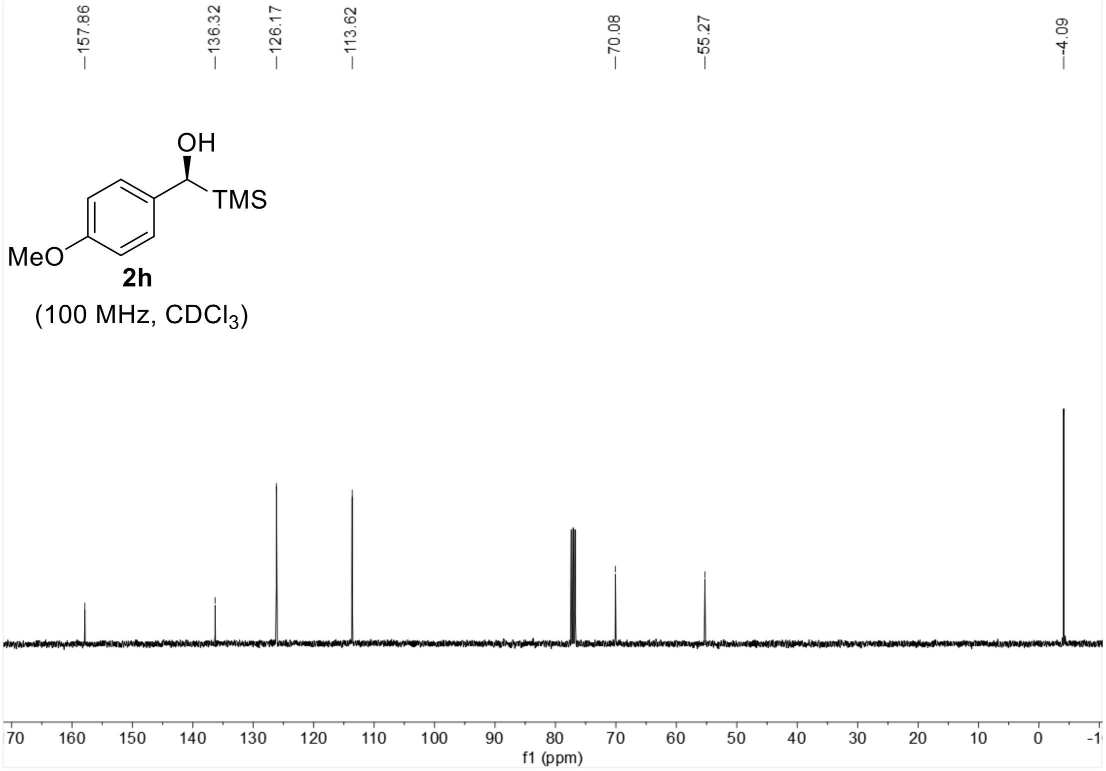
157.86
136.32
126.17
113.62
70.08
55.27
-4.09
OH
TMS
MeO
2h
(100 MHz, CDCl3)
70 160 150 140 130 120 110 100 90 80 70 60 50 40 30 20 10 0
f1 (ppm)

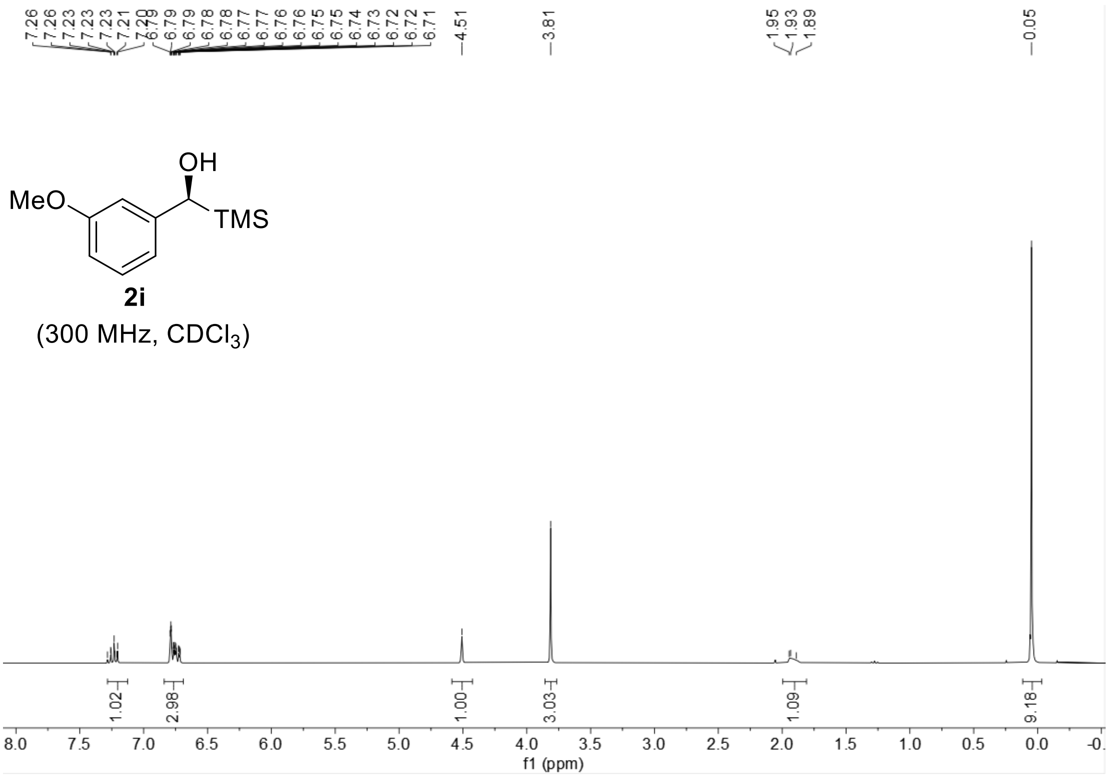
7.26
7.26
7.23
7.23
7.23
7.21
7.20
7.20
6.79
6.79
6.79
6.78
6.78
6.77
6.77
6.76
6.76
6.75
6.75
6.74
6.73
6.72
6.72
6.71
4.51
3.81
1.95
1.93
1.89
0.05
OH
MeO
TMS
2i
(300 MHz, CDCl3)
1.02
2.98
1.00
3.03
1.09
9.18
8.0 7.5 7.0 6.5 6.0 5.5 5.0 4.5 4.0 3.5 3.0 2.5 2.0 1.5 1.0 0.5 0.0 -0.
f1 (ppm)

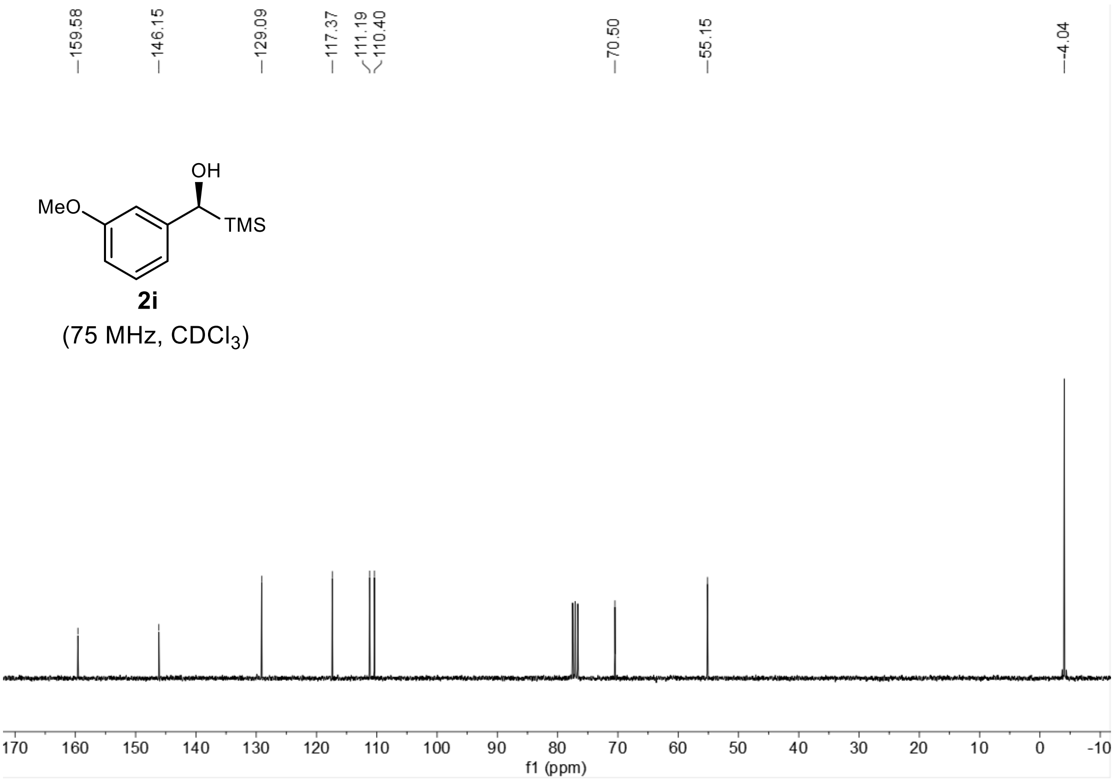
159.58
146.15
129.09
117.37
111.19
110.40
70.50
55.15
-4.04
OH
MeO
TMS
2i
(75 MHz, CDCl3)
170 160 150 140 130 120 110 100 90 80 70 60 50 40 30 20 10 0 -10
f1 (ppm)

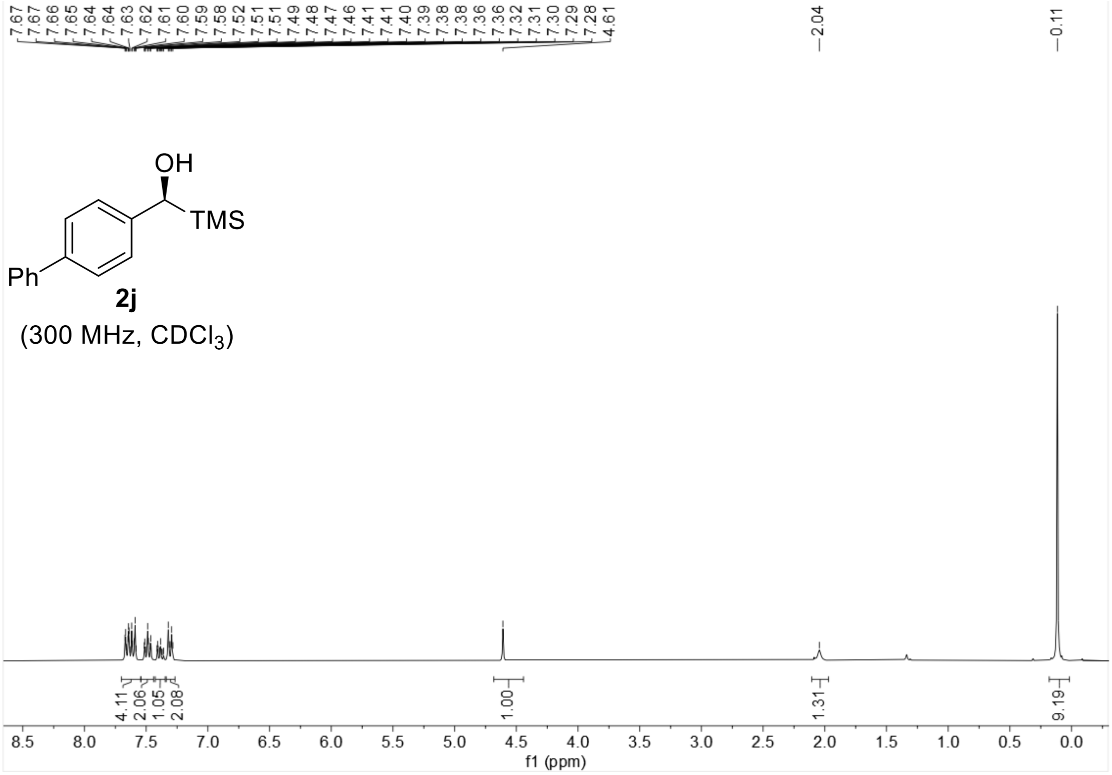
7.67
7.67
7.66
7.65
7.64
7.64
7.63
7.62
7.61
7.60
7.59
7.58
7.52
7.51
7.51
7.49
7.48
7.47
7.46
7.41
7.41
7.40
7.39
7.38
7.38
7.36
7.36
7.32
7.31
7.30
7.29
7.28
4.61
2.04
0.11
OH
TMS
Ph
2j
(300 MHz, CDCl3)
4.11
2.06
1.05
2.08
1.00
1.31
9.19
f1 (ppm)

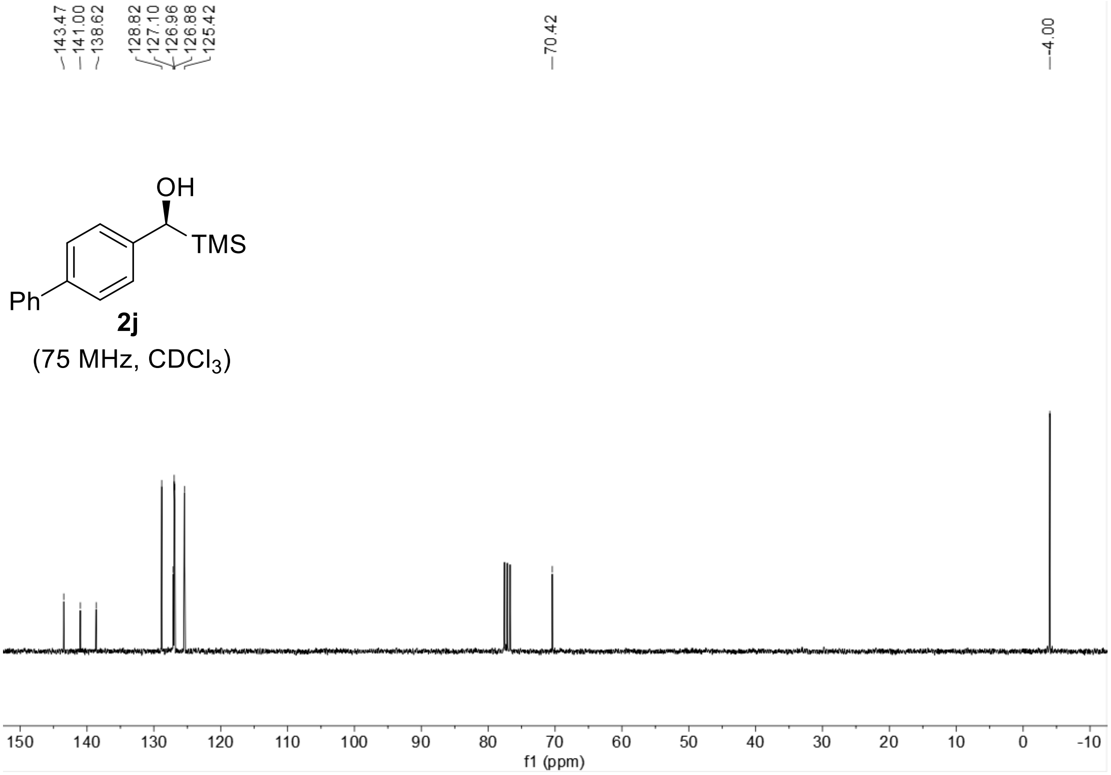
143.47
141.00
138.62
128.82
127.10
126.96
126.88
125.42
70.42
-4.00
OH
TMS
Ph
2j
(75 MHz, CDCl3)
f1 (ppm)

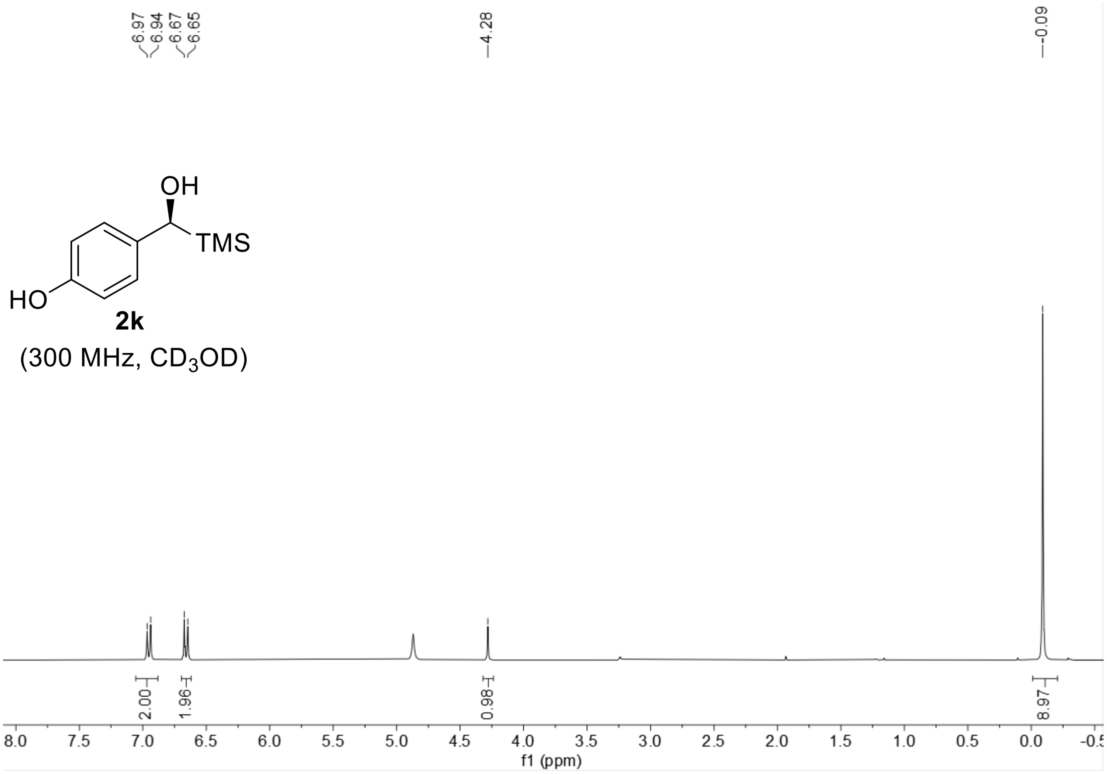
6.97
6.94
6.67
6.65
4.28
-0.09
OH
TMS
HO
2k
(300 MHz, CD3OD)
2.00
1.96
0.98
8.97
f1 (ppm)

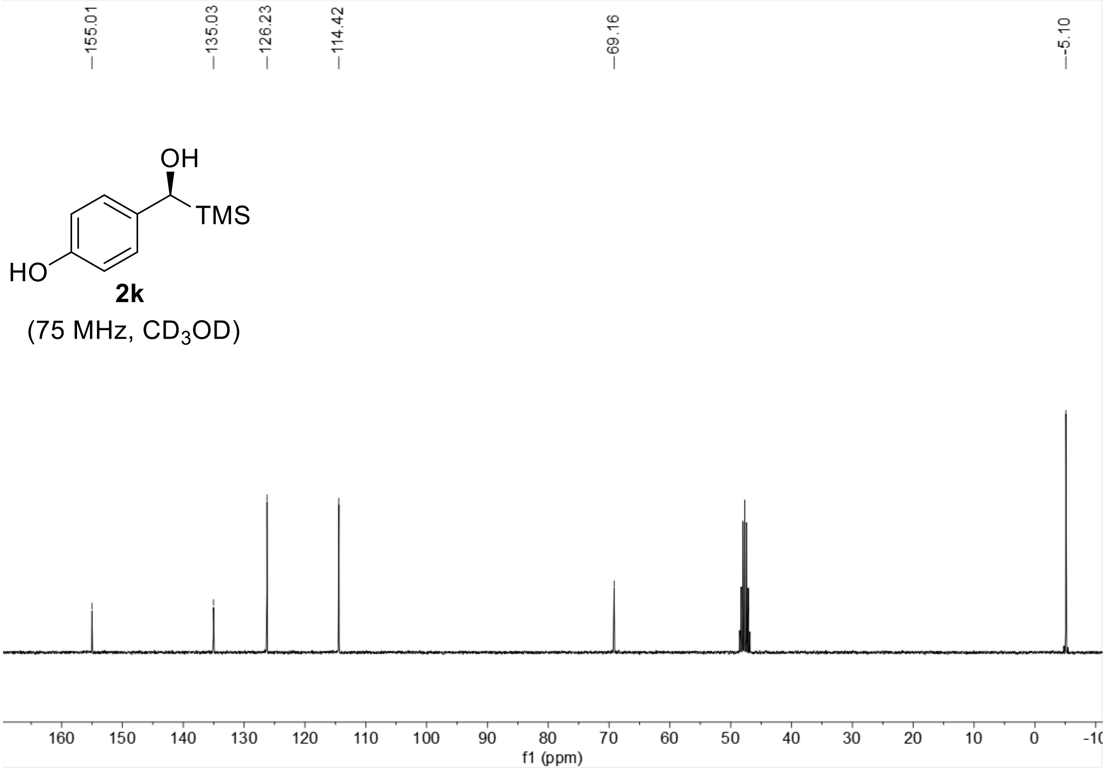
155.01
135.03
126.23
114.42
69.16
-5.10
OH
TMS
HO
2k
(75 MHz, CD3OD)
f1 (ppm)

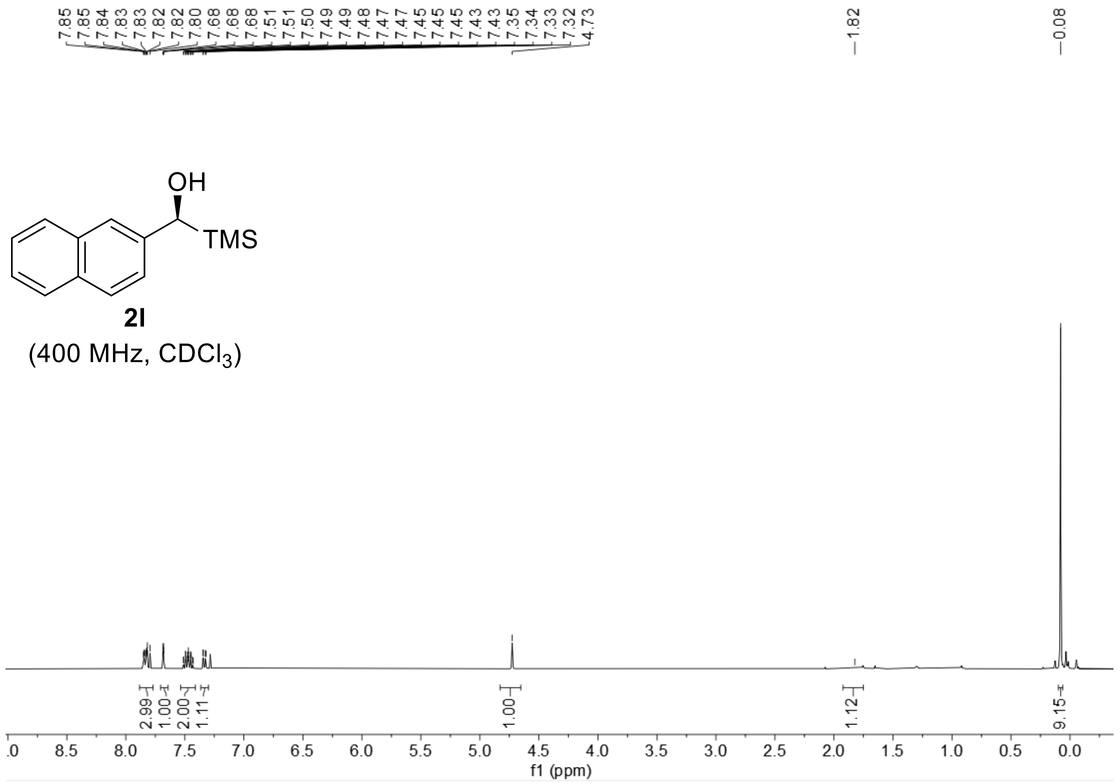
7.85
7.85
7.84
7.83
7.83
7.82
7.82
7.80
7.68
7.68
7.68
7.51
7.51
7.50
7.49
7.48
7.47
7.47
7.45
7.45
7.45
7.43
7.43
7.35
7.34
7.33
7.32
4.73
1.82
0.08
OH
TMS
2l
(400 MHz, CDCl3)
2.99
1.00
2.00
1.11
1.00
1.12
9.15
f1 (ppm)

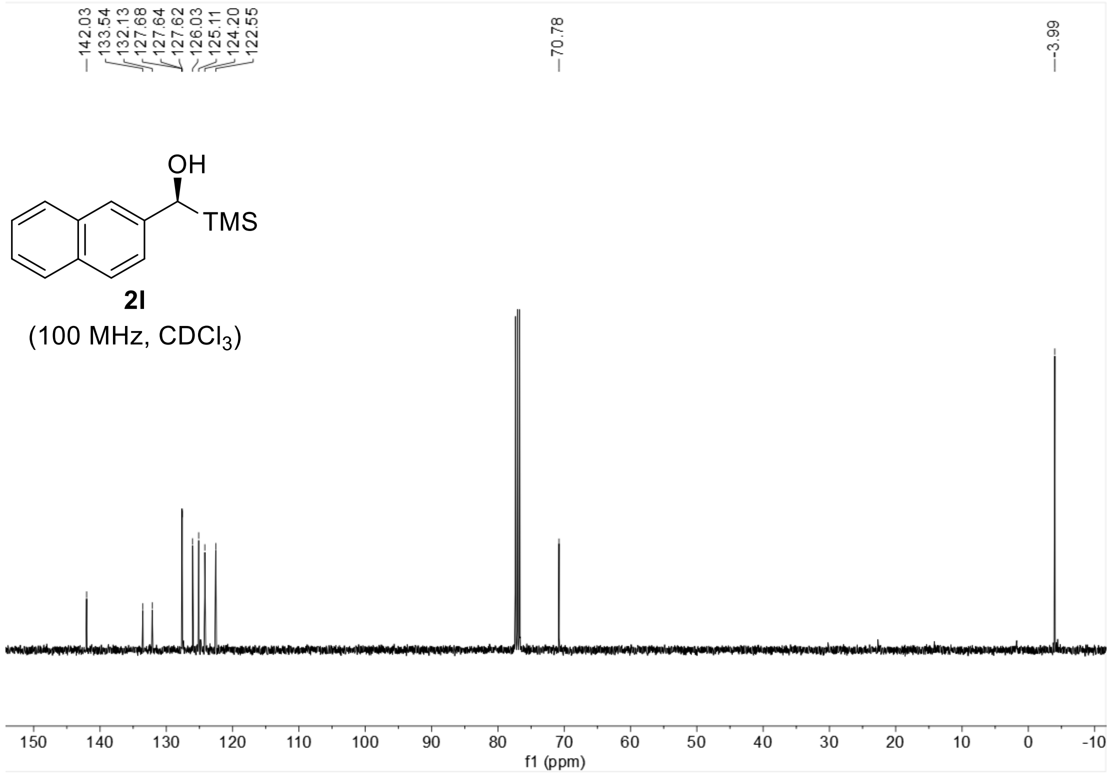
142.03
133.54
132.13
127.68
127.64
127.62
126.03
125.11
124.20
122.55
70.78
-3.99
OH
TMS
2l
(100 MHz, CDCl3)
f1 (ppm)

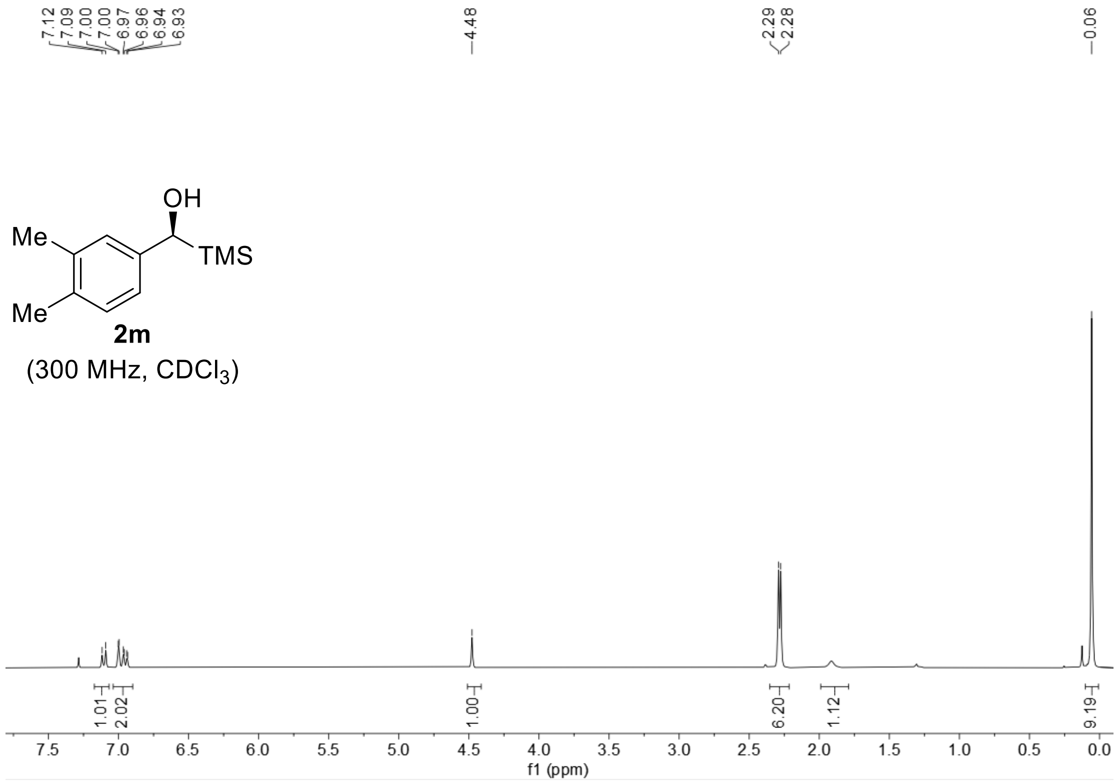
7.12
7.09
7.00
7.00
6.97
6.96
6.94
6.93
4.48
2.29
2.28
0.06
OH
Me
TMS
Me
2m
(300 MHz, CDCl3)
1.01
2.02
1.00
6.20
1.12
9.19
7.5 7.0 6.5 6.0 5.5 5.0 4.5 4.0 3.5 3.0 2.5 2.0 1.5 1.0 0.5 0.0
f1 (ppm)

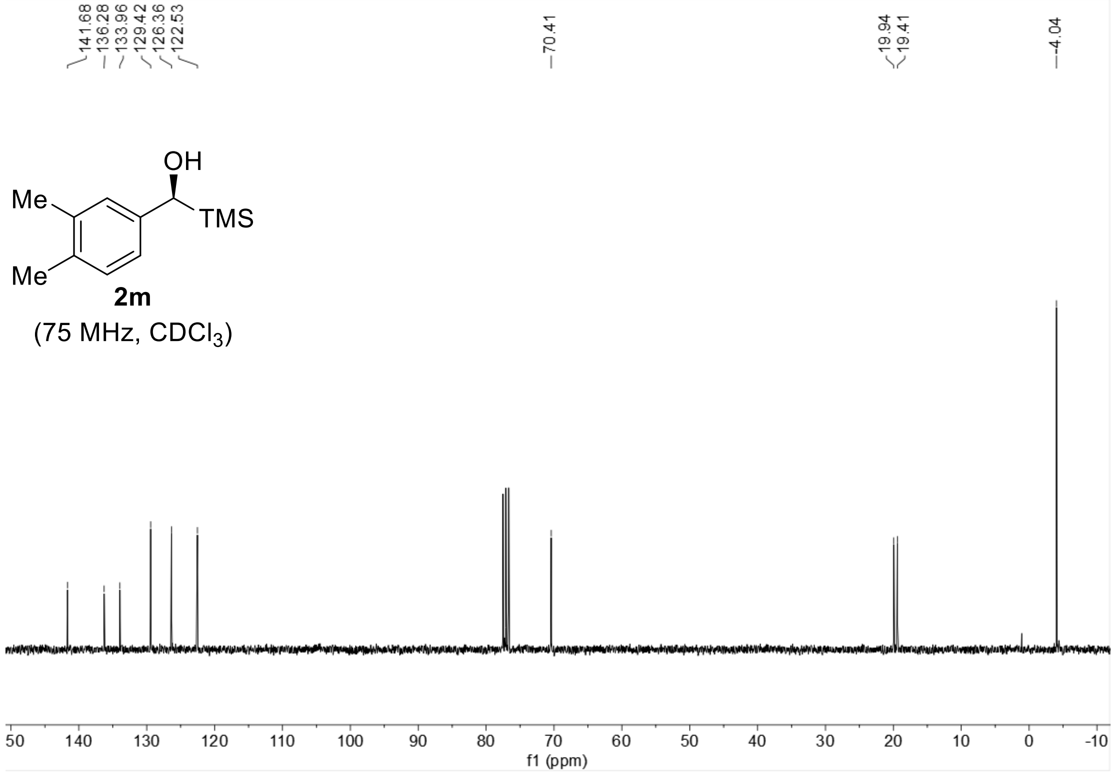
141.68
136.28
133.96
129.42
126.36
122.53
70.41
19.94
19.41
-4.04
OH
Me
TMS
Me
2m
(75 MHz, CDCl3)
50 140 130 120 110 100 90 80 70 60 50 40 30 20 10 0 -10
f1 (ppm)

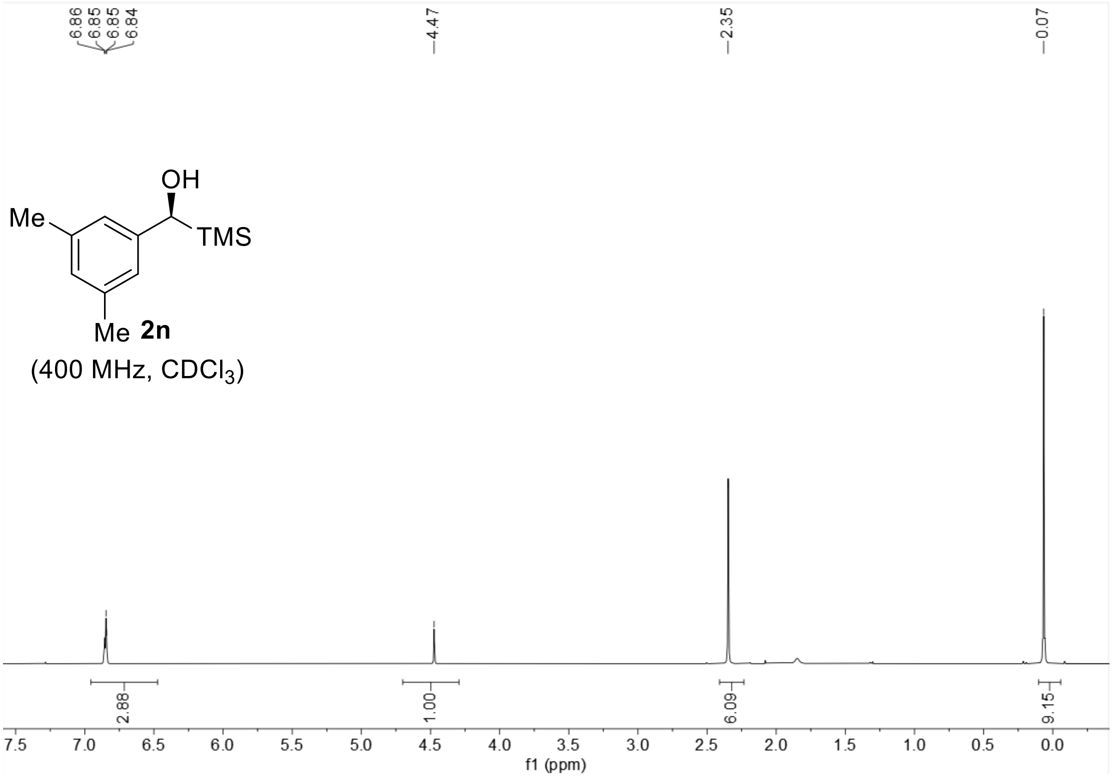
6.86
6.85
6.85
6.84
4.47
2.35
0.07
OH
Me
TMS
Me 2n
(400 MHz, CDCl3)
2.88
1.00
6.09
9.15
f1 (ppm)

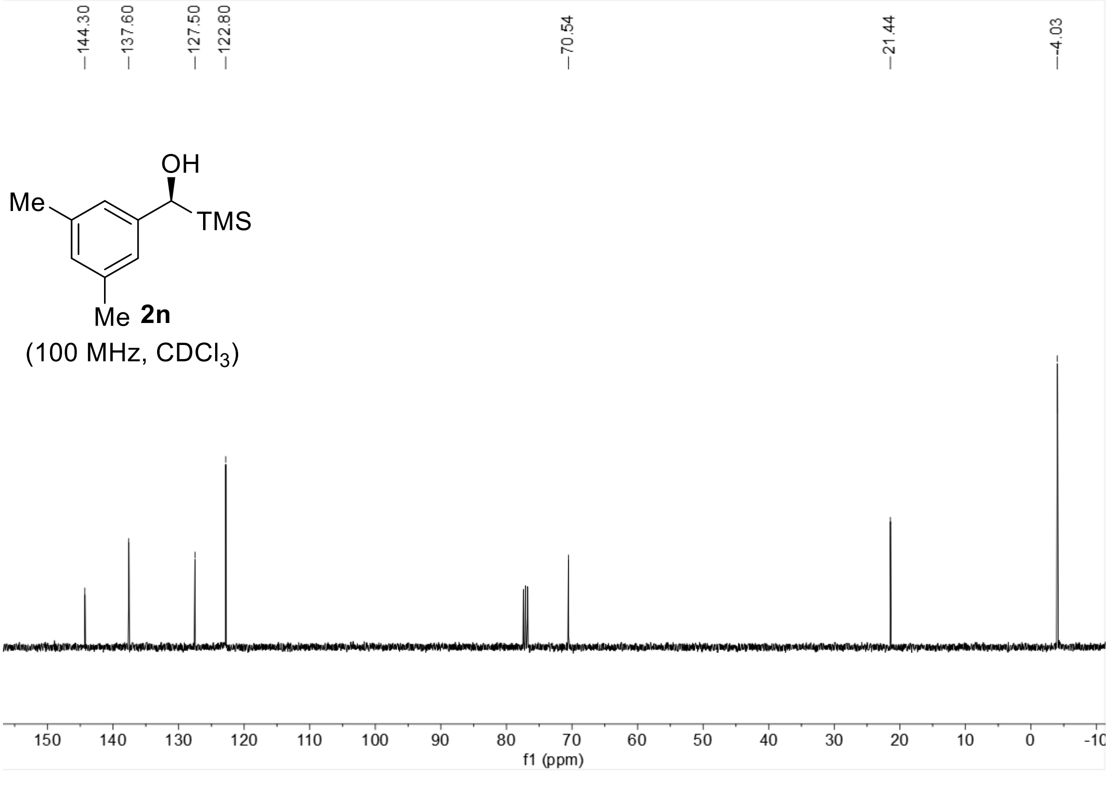
144.30
137.60
127.50
122.80
70.54
21.44
-4.03
OH
Me
TMS
Me 2n
(100 MHz, CDCl3)
f1 (ppm)

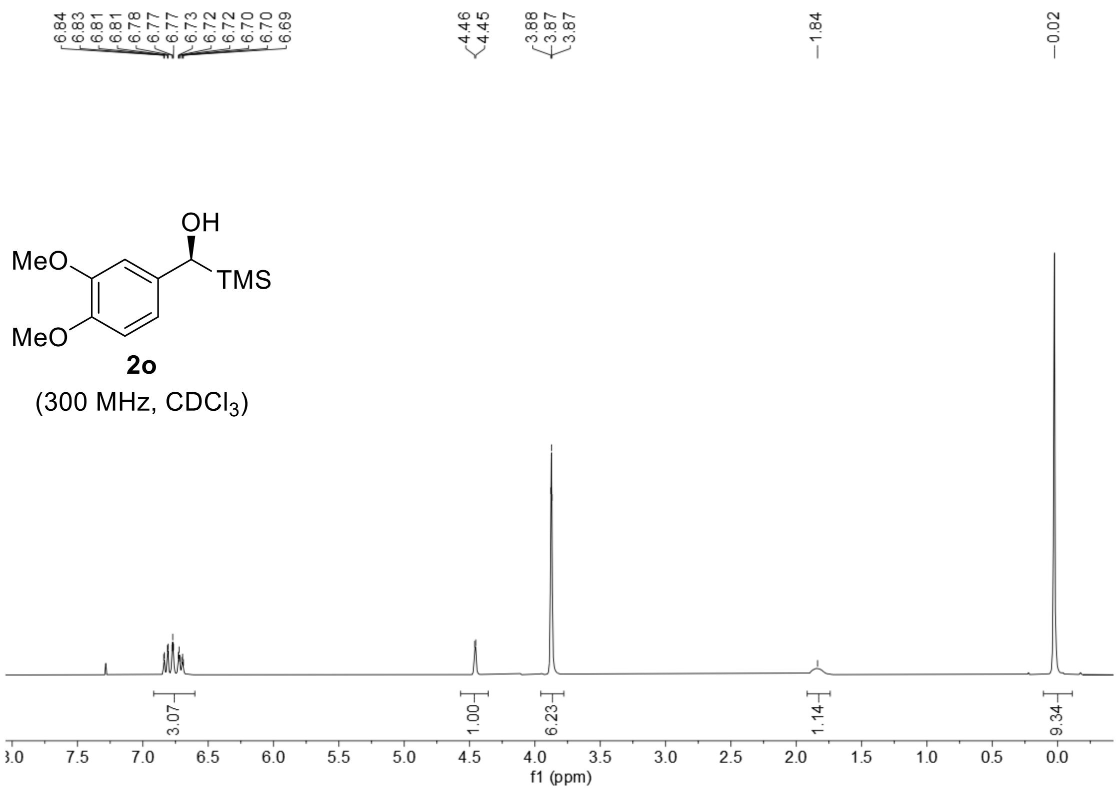
6.84
6.83
6.81
6.81
6.78
6.77
6.77
6.73
6.72
6.72
6.70
6.70
6.69
4.46
4.45
3.88
3.87
3.87
1.84
0.02
OH
MeO
TMS
MeO
2o
(300 MHz, CDCl3)
3.07
1.00
6.23
1.14
9.34
f1 (ppm)

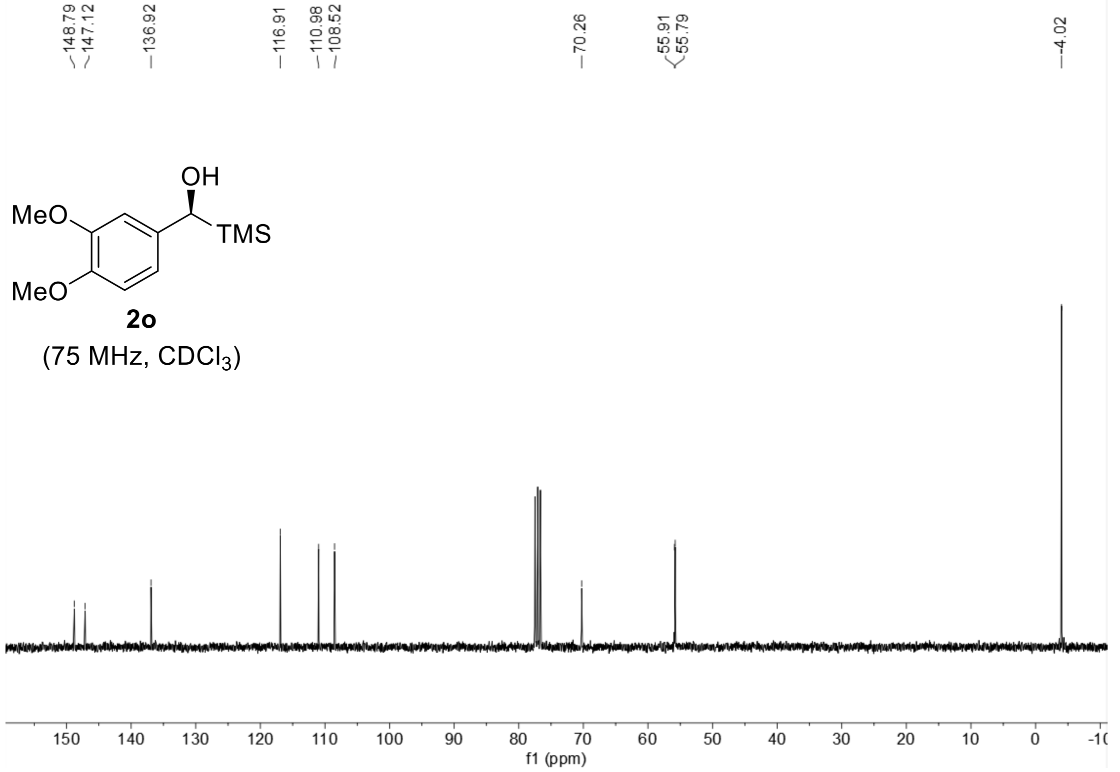
148.79
147.12
136.92
116.91
110.98
108.52
70.26
55.91
55.79
4.02
OH
MeO
TMS
MeO
2o
(75 MHz, CDCl3)
f1 (ppm)

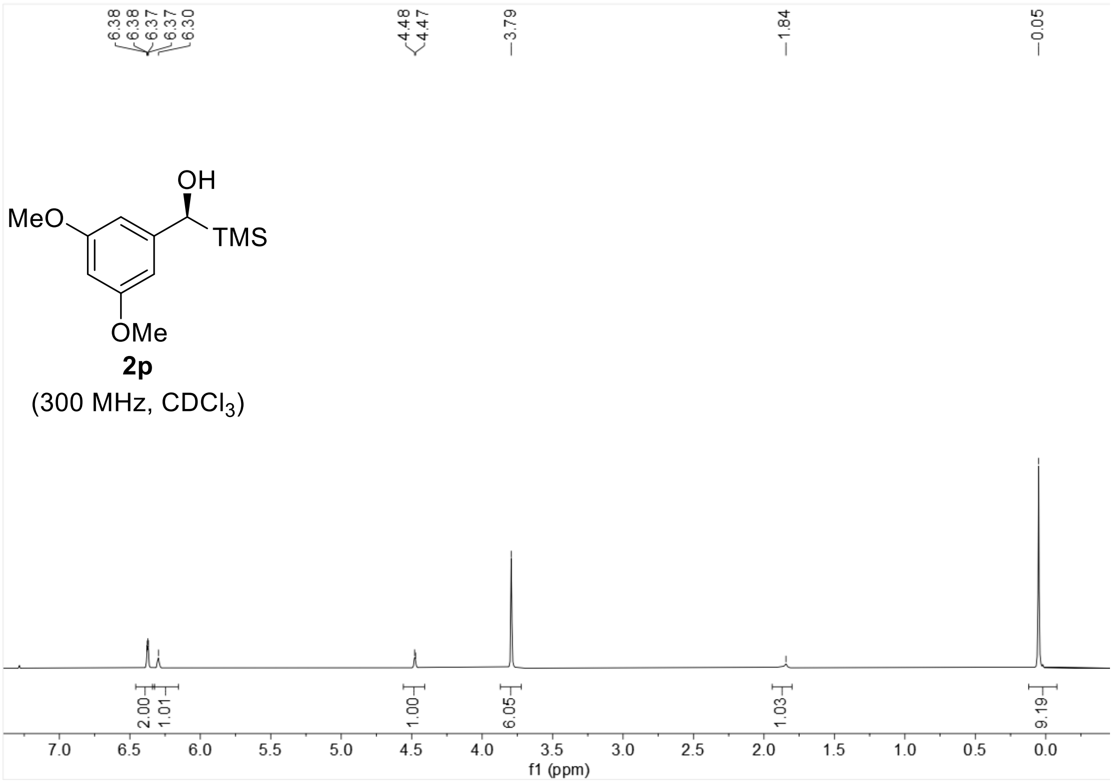

2p

(300 MHz, $CDCl_3$)

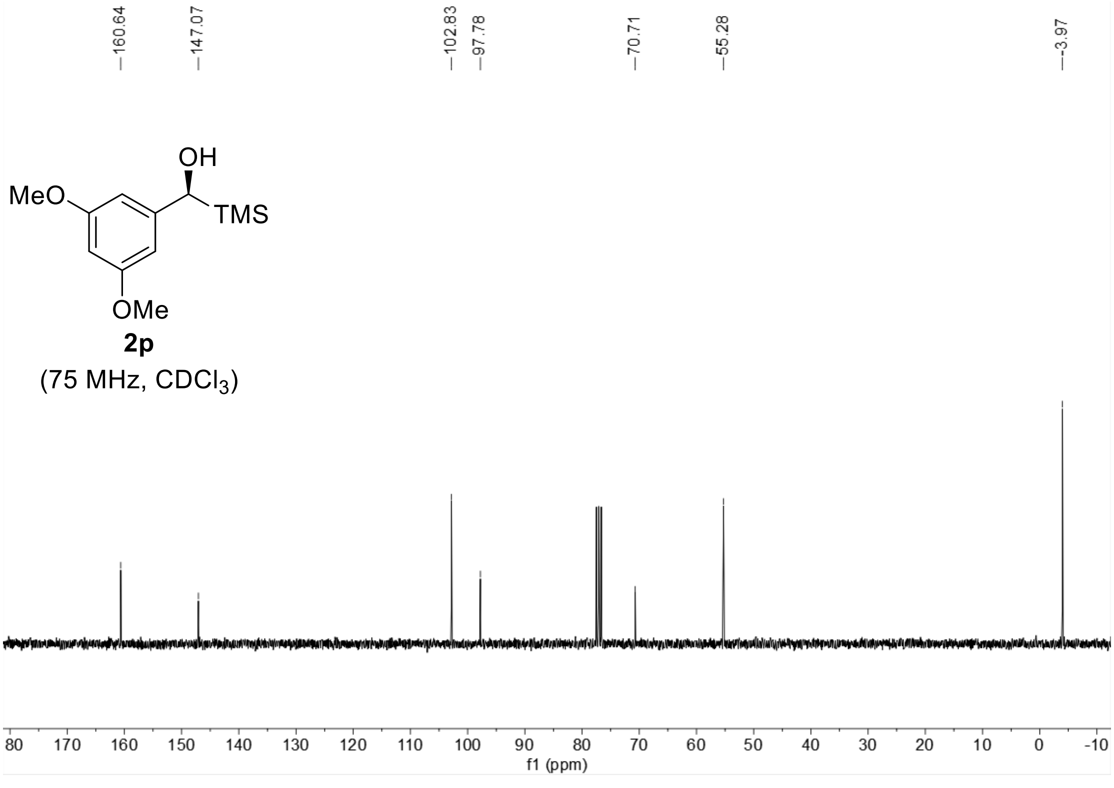

2p

(75 MHz, $CDCl_3$)

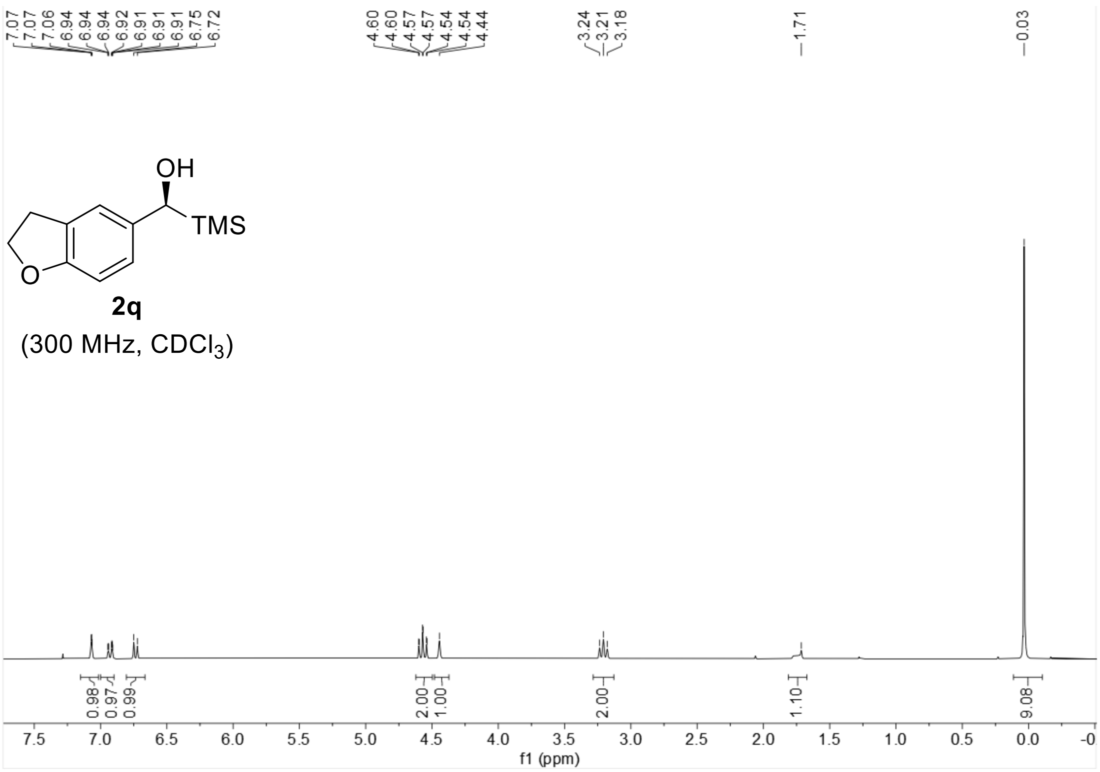
7.07
7.07
7.06
6.94
6.94
6.94
6.92
6.91
6.91
6.91
6.75
6.72
4.60
4.60
4.57
4.57
4.54
4.54
4.44
3.24
3.21
3.18
1.71
0.03
OH
TMS
O
2q
(300 MHz, CDCl3)
0.98
0.97
0.99
2.00
1.00
2.00
1.10
9.08
f1 (ppm)

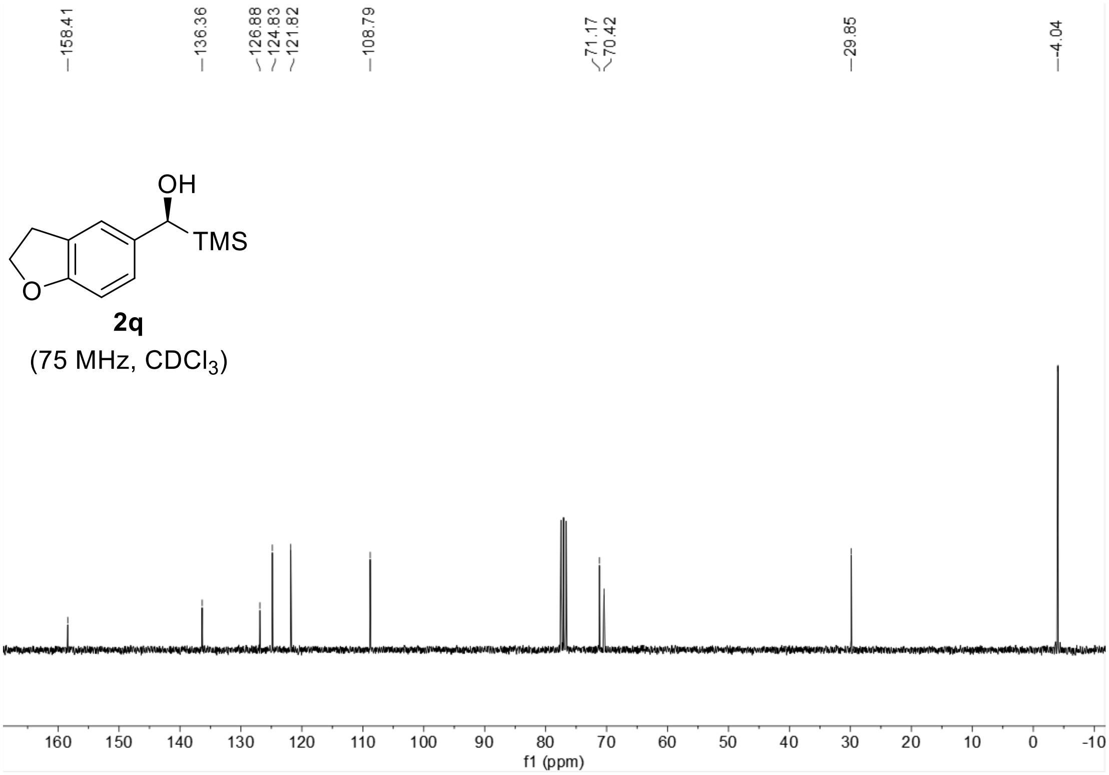
158.41
136.36
126.88
124.83
121.82
108.79
71.17
70.42
29.85
-4.04
OH
TMS
O
2q
(75 MHz, CDCl3)
f1 (ppm)

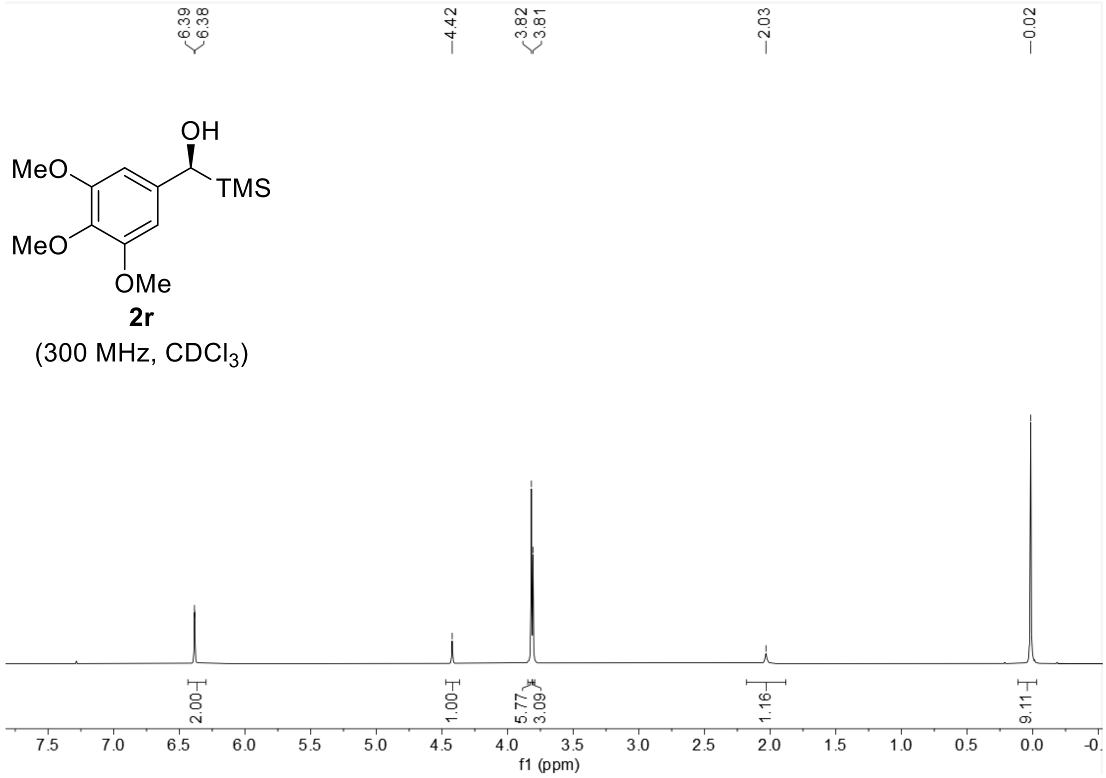

6.39
6.38
4.42
3.82
3.81
2.03
0.02
OH
MeO
TMS
MeO
OMe
2r
(300 MHz, CDCl3)
2.00
1.00
5.77
3.09
1.16
9.11
f1 (ppm)

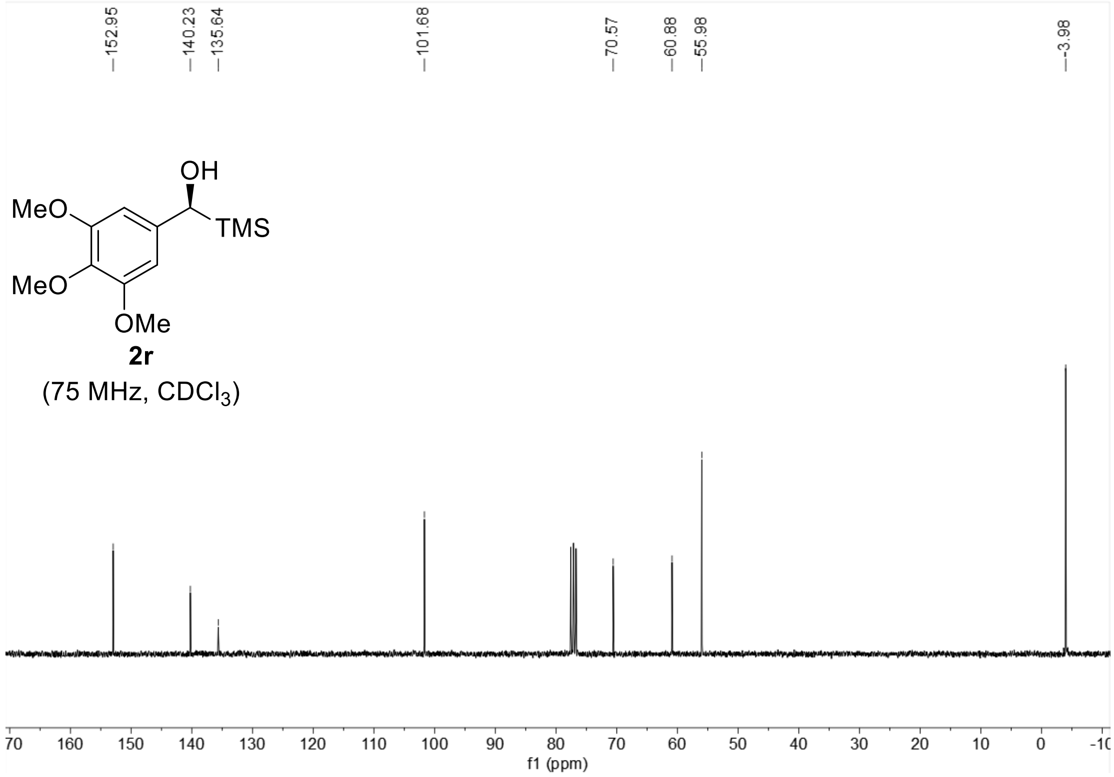

152.95
140.23
135.64
101.68
70.57
60.88
55.98
-3.98
OH
MeO
TMS
MeO
OMe
2r
(75 MHz, CDCl3)
f1 (ppm)

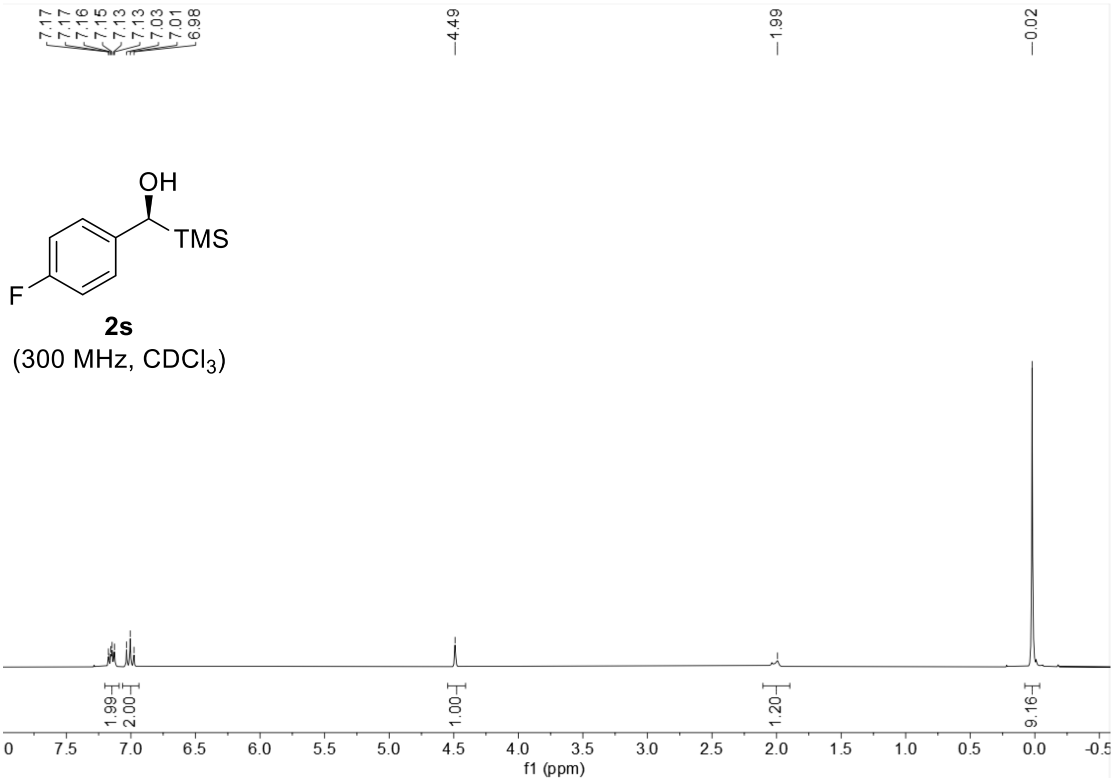
7.17
7.17
7.16
7.15
7.13
7.13
7.03
7.01
6.98
4.49
1.99
0.02
OH
TMS
F
2s
(300 MHz, CDCl3)
1.99
2.00
1.00
1.20
9.16
f1 (ppm)

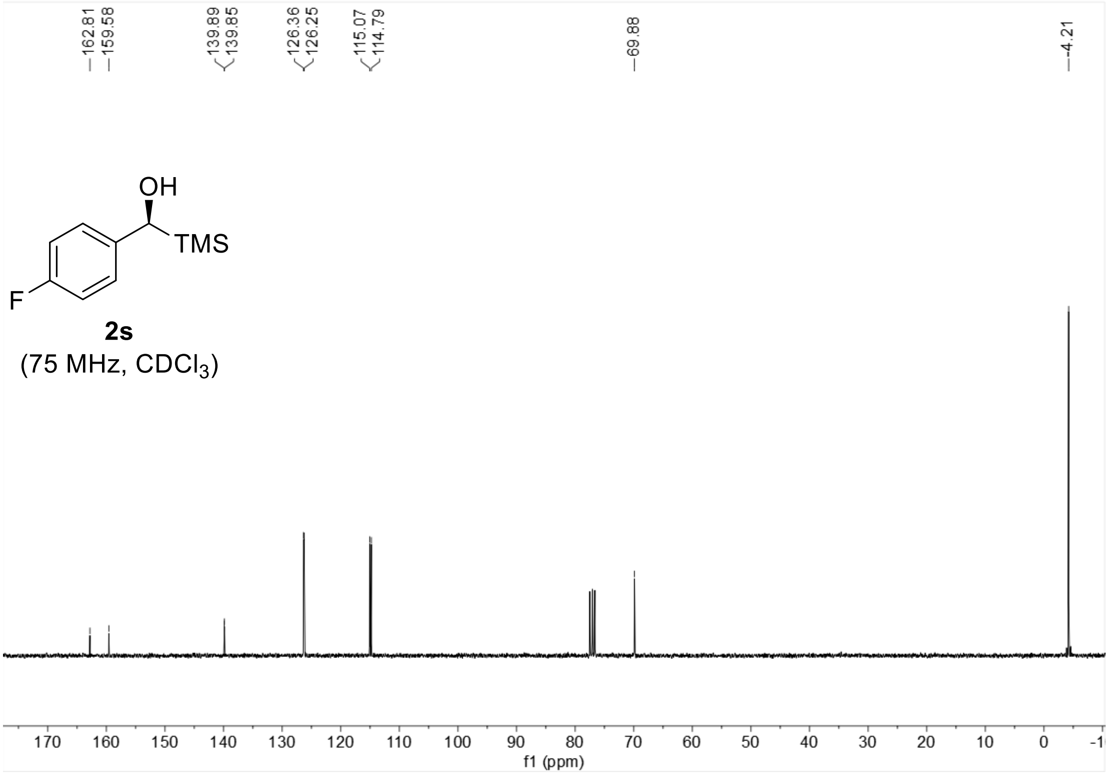
162.81
159.58
139.89
139.85
126.36
126.25
115.07
114.79
69.88
-4.21
OH
TMS
F
2s
(75 MHz, CDCl3)
f1 (ppm)

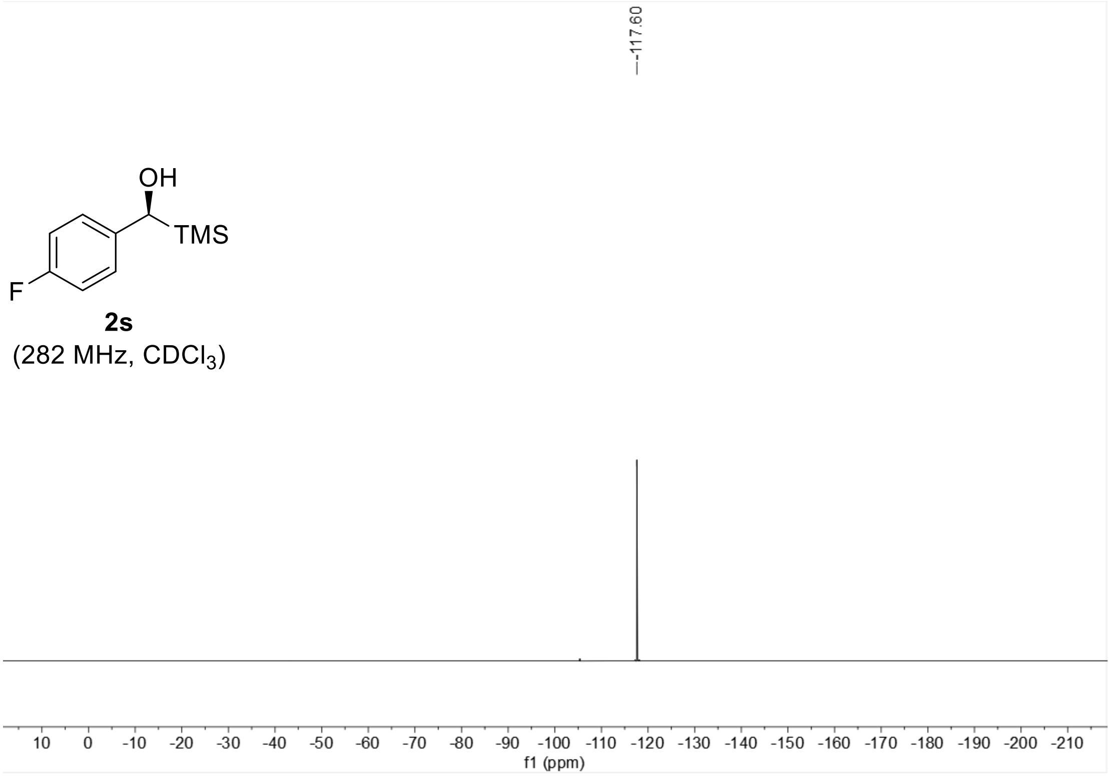

-117.60
OH
TMS
F
2s
(282 MHz, CDCl3)
10 0 -10 -20 -30 -40 -50 -60 -70 -80 -90 -100 -110 -120 -130 -140 -150 -160 -170 -180 -190 -200 -210
f1 (ppm)

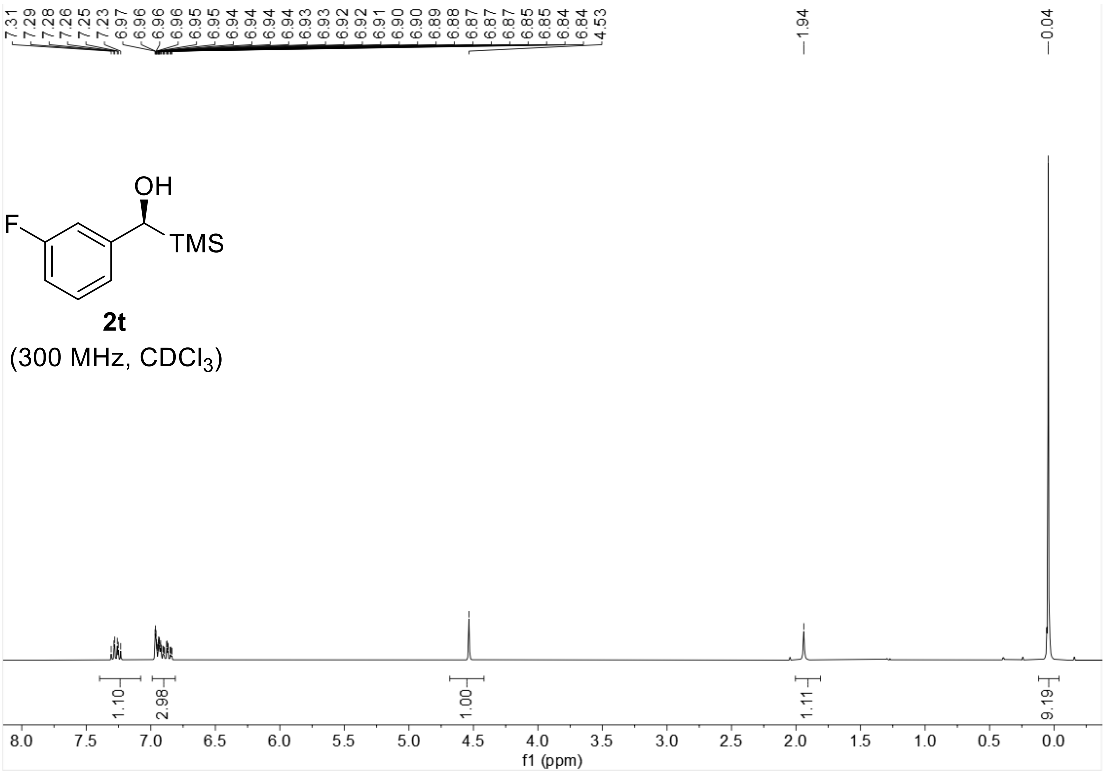

2t
(300 MHz, $CDCl_3$)

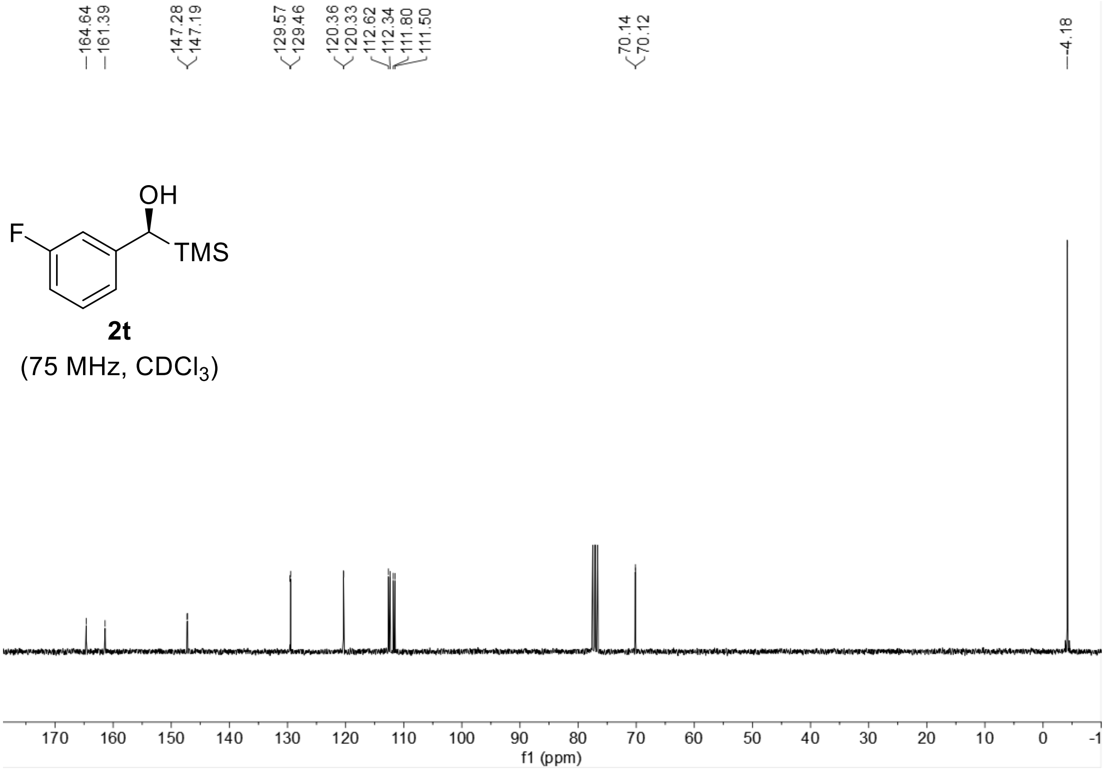

2t
(75 MHz, $CDCl_3$)

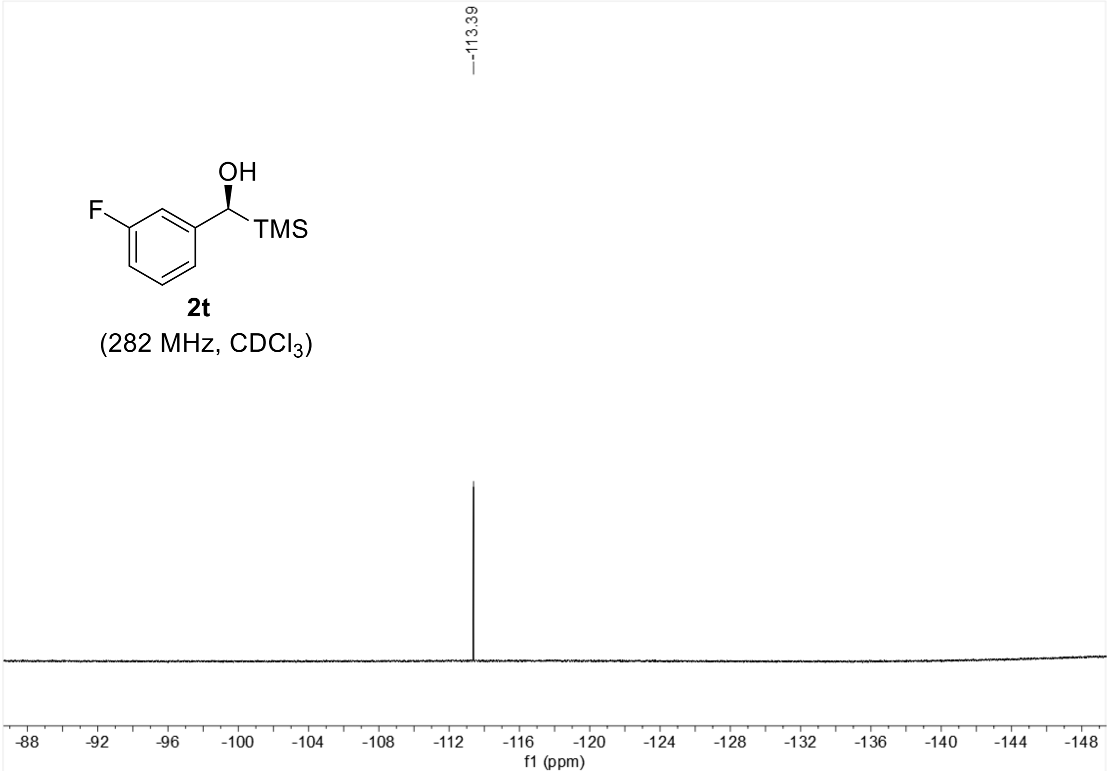

2t
(282 MHz, $CDCl_3$)

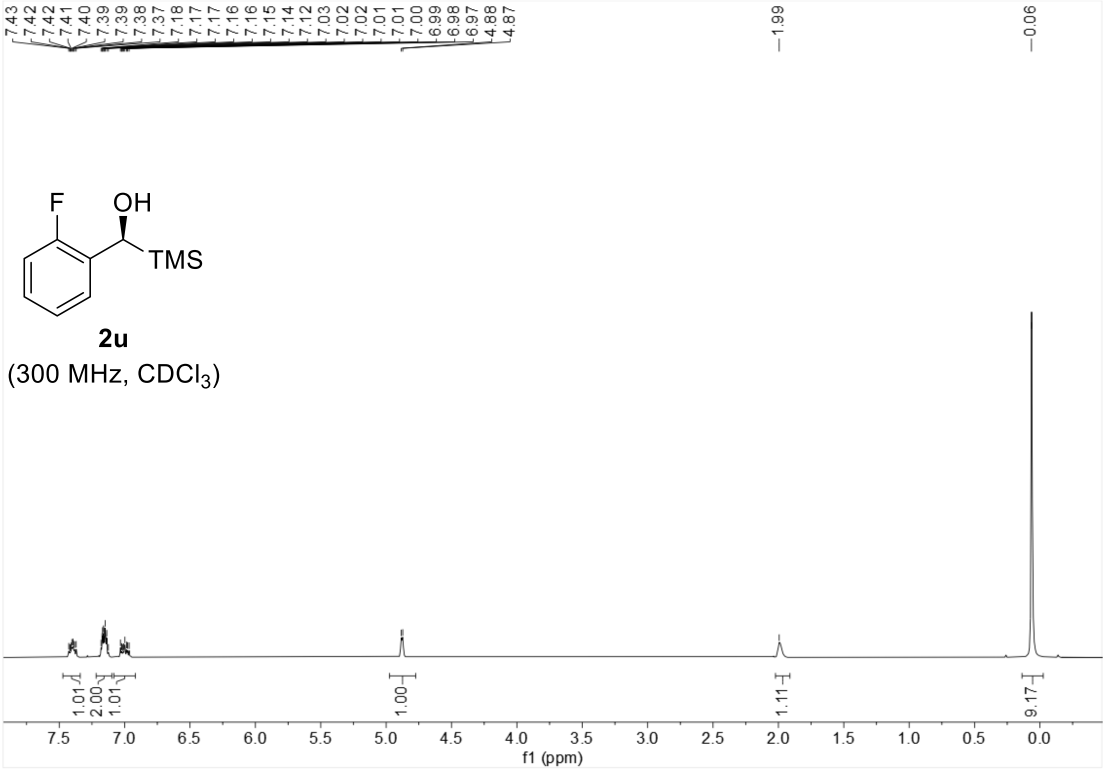
7.43
7.42
7.42
7.41
7.40
7.39
7.39
7.38
7.37
7.18
7.17
7.17
7.16
7.16
7.15
7.14
7.12
7.03
7.02
7.02
7.01
7.01
7.00
6.99
6.98
6.97
4.88
4.87
1.99
0.06
F
OH
TMS
2u
(300 MHz, CDCl3)
1.01
2.00
1.01
1.00
1.11
9.17
f1 (ppm)

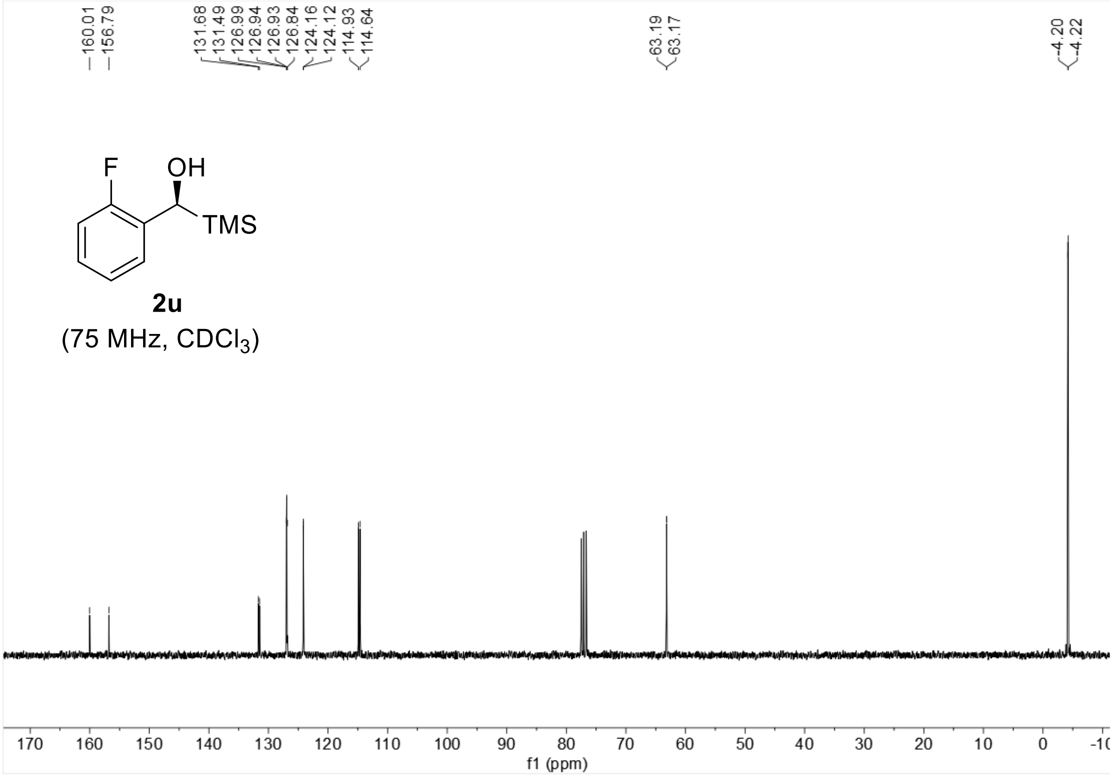
160.01
156.79
131.68
131.49
126.99
126.94
126.93
126.84
124.16
124.12
114.93
114.64
63.19
63.17
-4.20
-4.22
F
OH
TMS
2u
(75 MHz, CDCl3)
f1 (ppm)

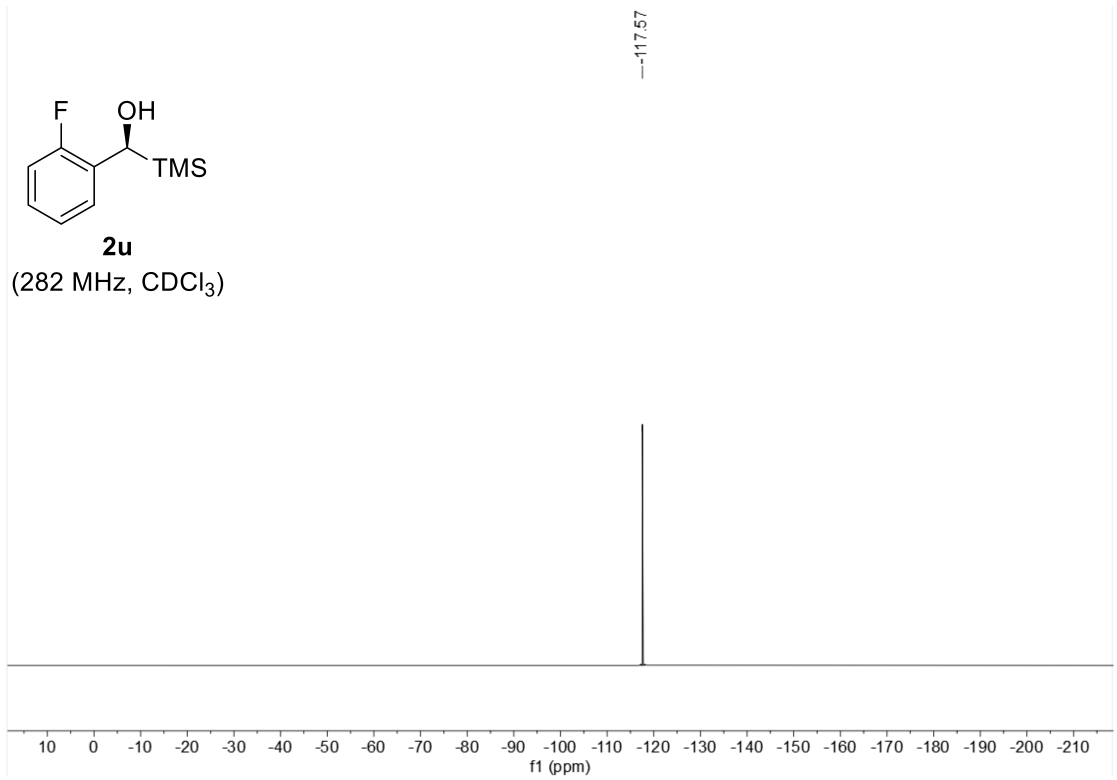
-117.57
F
OH
TMS
2u
(282 MHz, CDCl3)
10 0 -10 -20 -30 -40 -50 -60 -70 -80 -90 -100 -110 -120 -130 -140 -150 -160 -170 -180 -190 -200 -210
f1 (ppm)

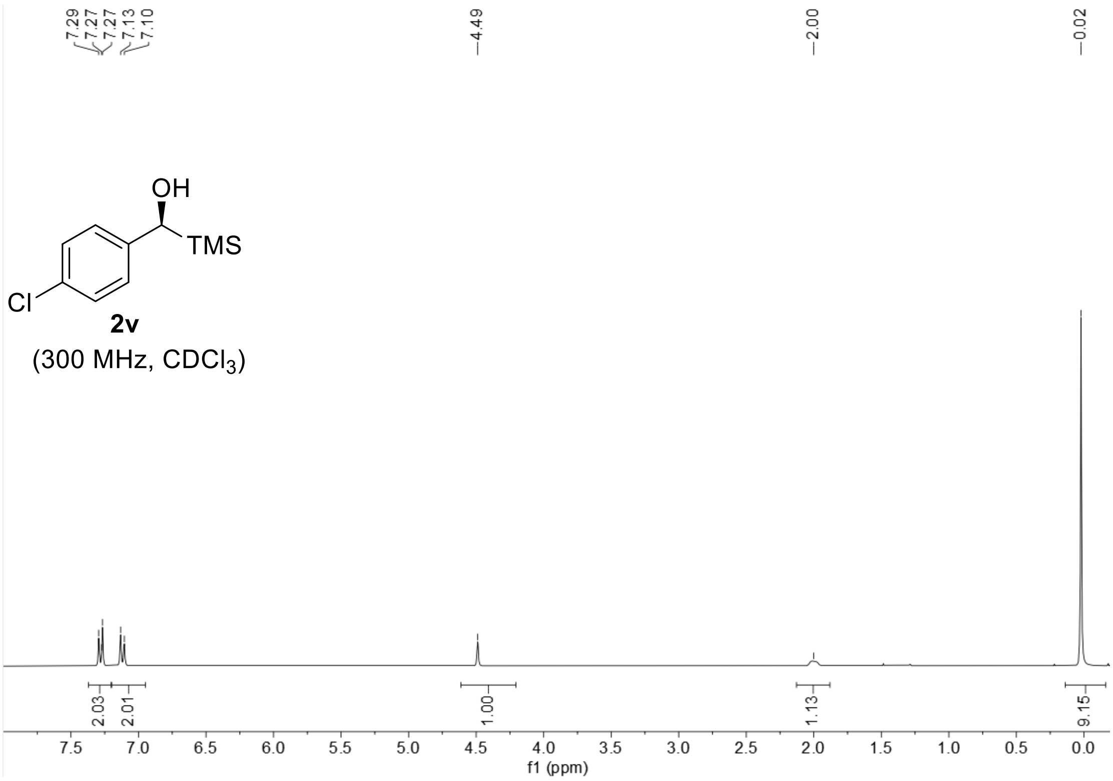
7.29
7.27
7.27
7.13
7.10
4.49
2.00
0.02
OH
TMS
Cl
2v
(300 MHz, CDCl3)
2.03
2.01
1.00
1.13
9.15
7.5 7.0 6.5 6.0 5.5 5.0 4.5 4.0 3.5 3.0 2.5 2.0 1.5 1.0 0.5 0.0
f1 (ppm)

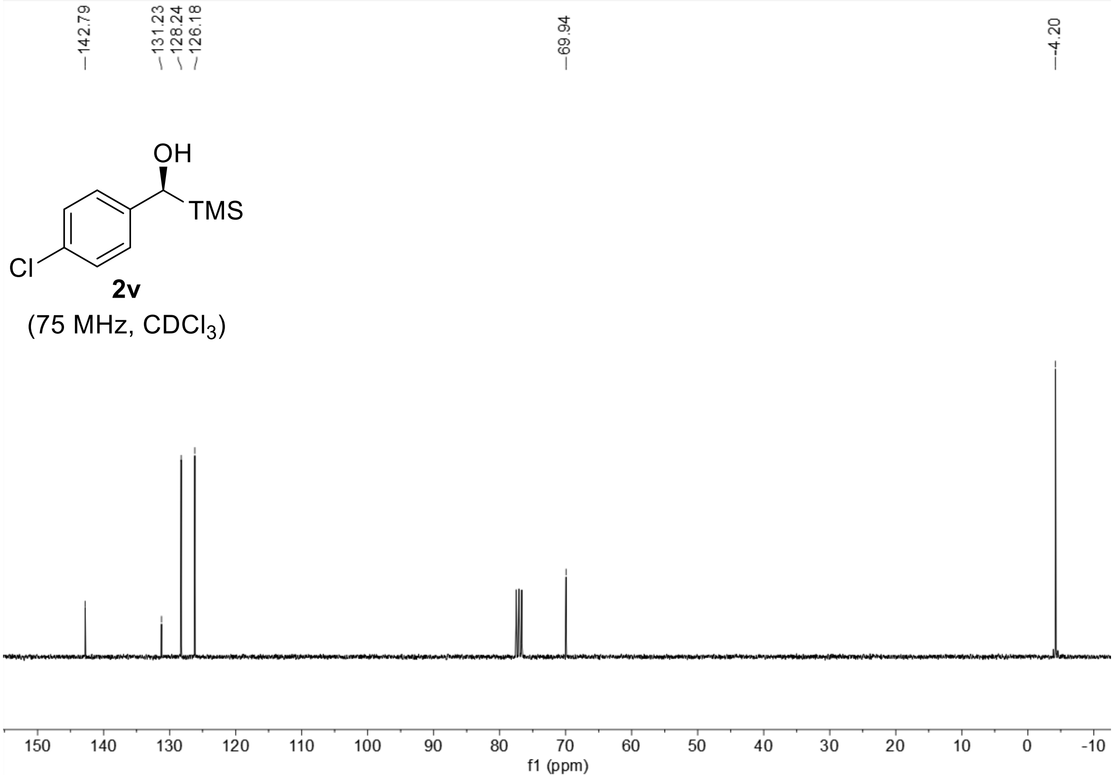
142.79
131.23
128.24
126.18
69.94
-4.20
OH
TMS
Cl
2v
(75 MHz, CDCl3)
150 140 130 120 110 100 90 80 70 60 50 40 30 20 10 0 -10
f1 (ppm)

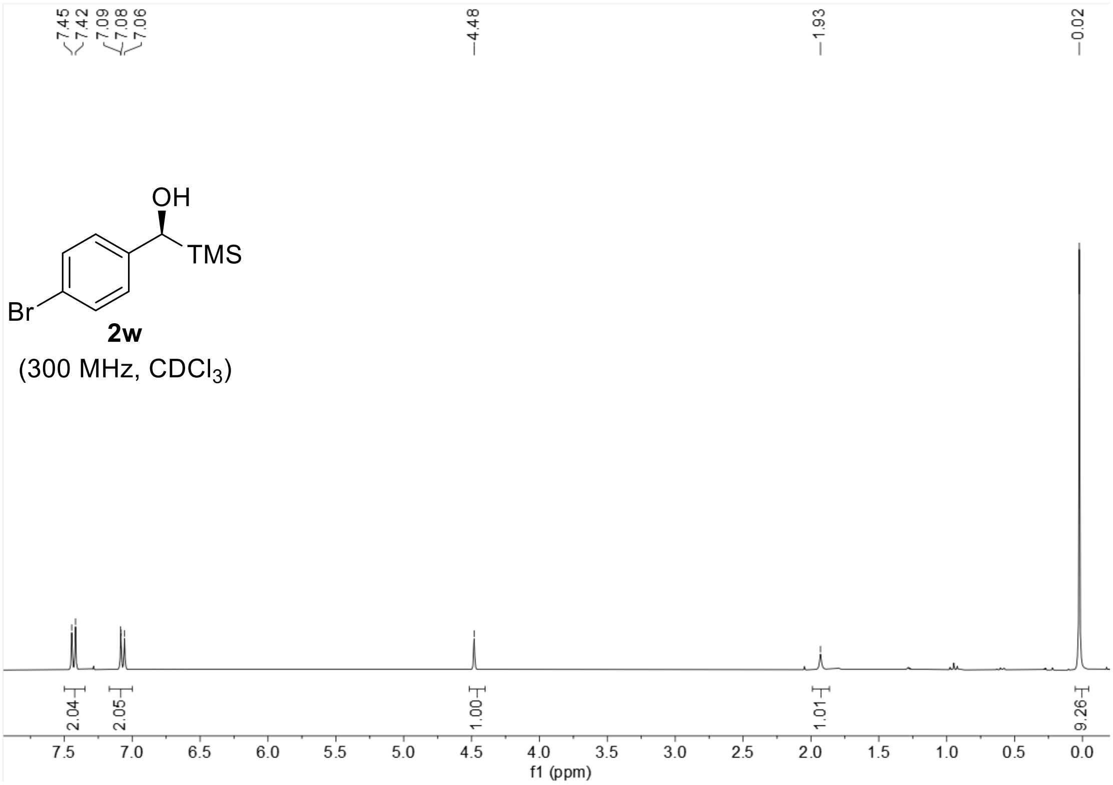
7.45
7.42
7.09
7.08
7.06
4.48
1.93
0.02
OH
TMS
Br
2w
(300 MHz, CDCl3)
2.04
2.05
1.00
1.01
9.26
f1 (ppm)

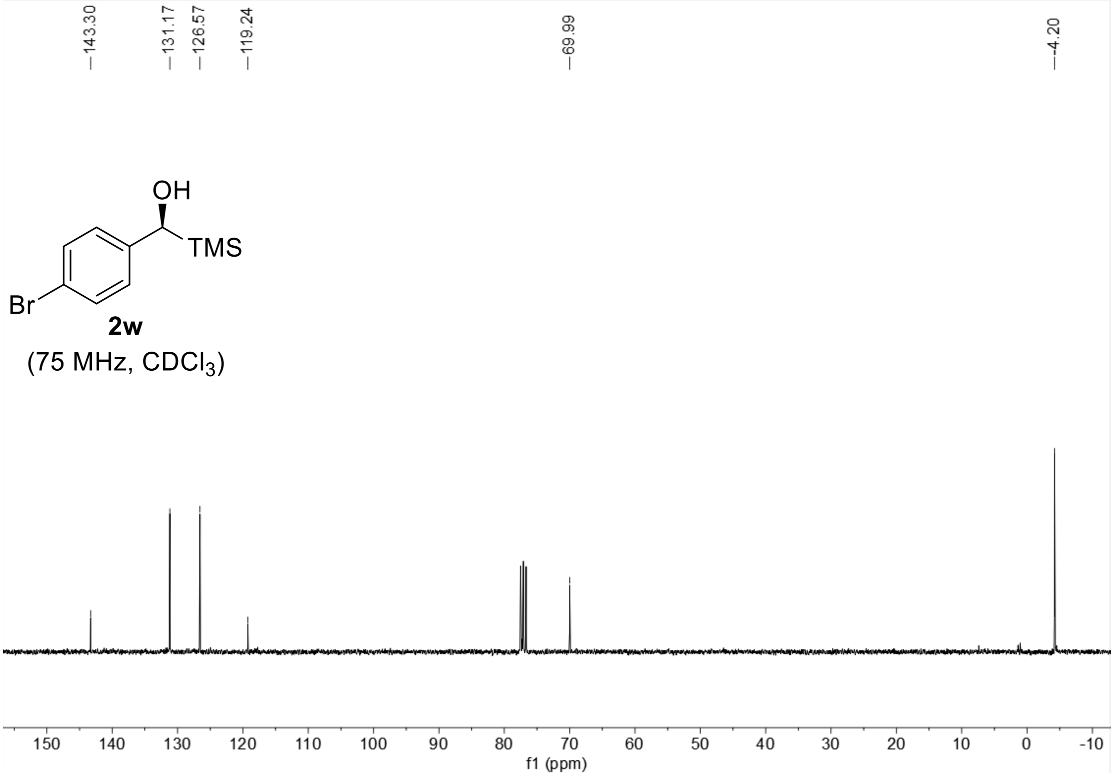
143.30
131.17
126.57
119.24
69.99
-4.20
OH
TMS
Br
2w
(75 MHz, CDCl3)
f1 (ppm)

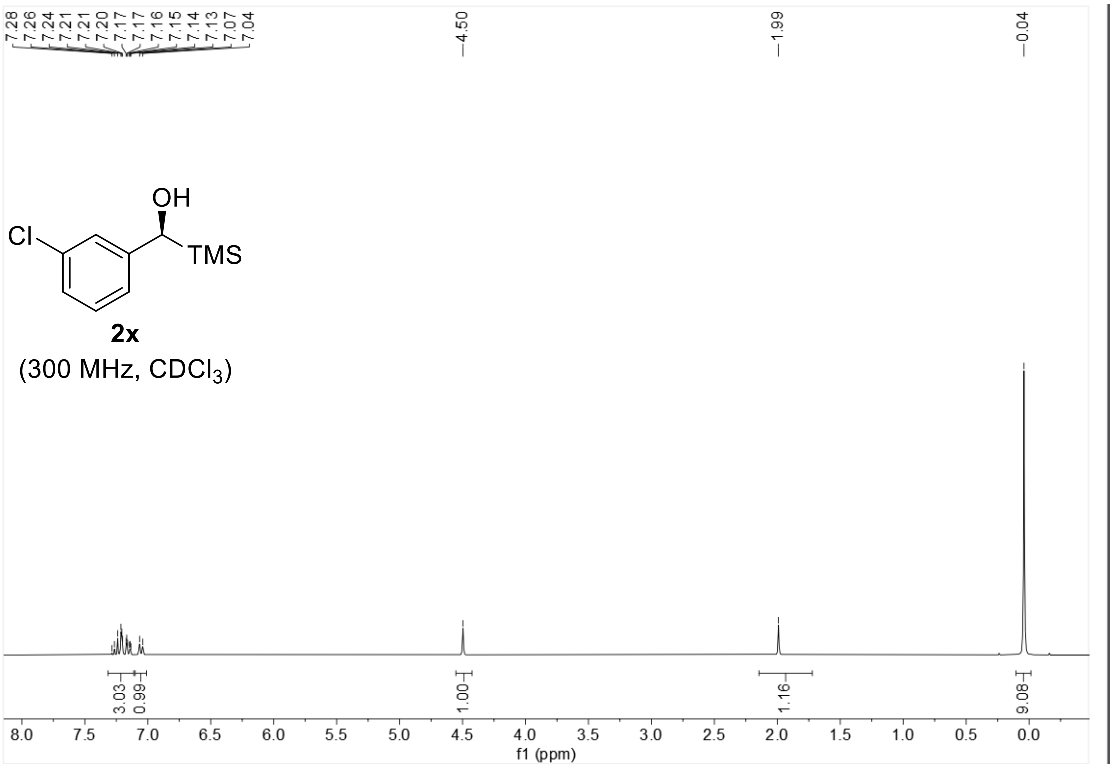
7.28
7.26
7.24
7.21
7.21
7.20
7.17
7.17
7.16
7.15
7.14
7.13
7.07
7.04
4.50
1.99
0.04
OH
Cl
TMS
2x
(300 MHz, CDCl3)
3.03
0.99
1.00
1.16
9.08
f1 (ppm)

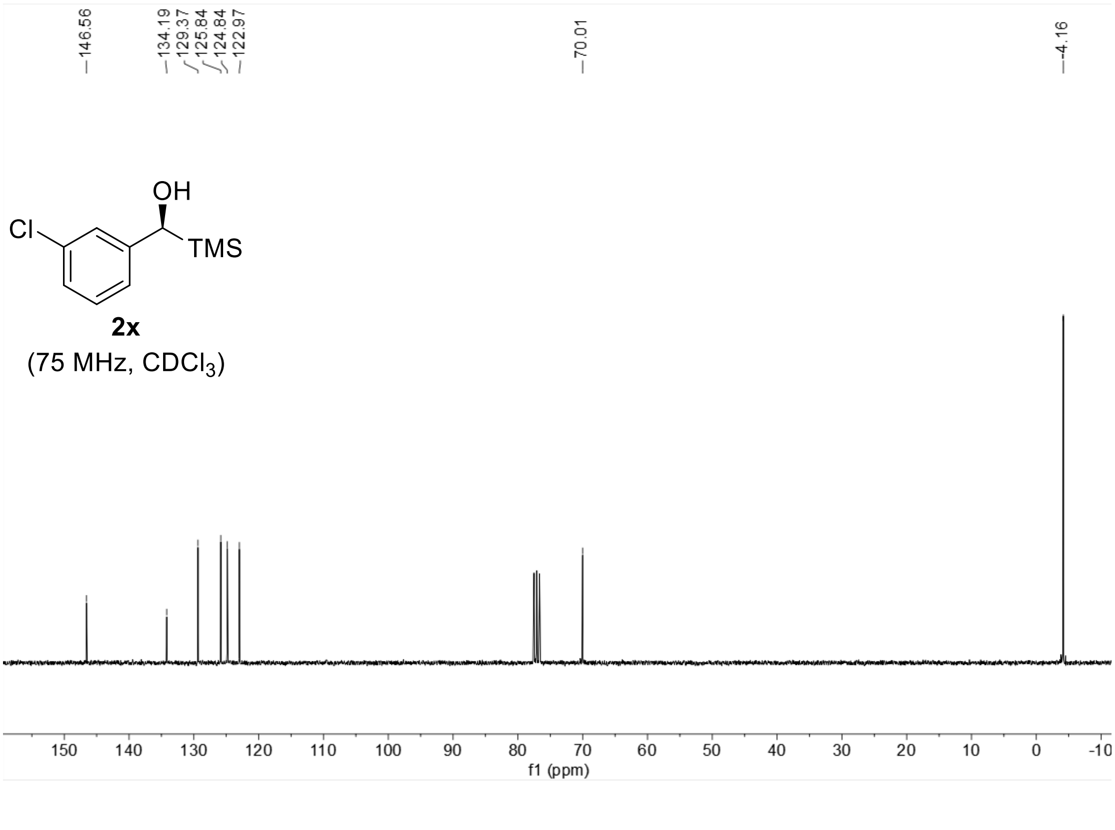
146.56
134.19
129.37
125.84
124.84
122.97
70.01
-4.16
OH
Cl
TMS
2x
(75 MHz, CDCl3)
f1 (ppm)

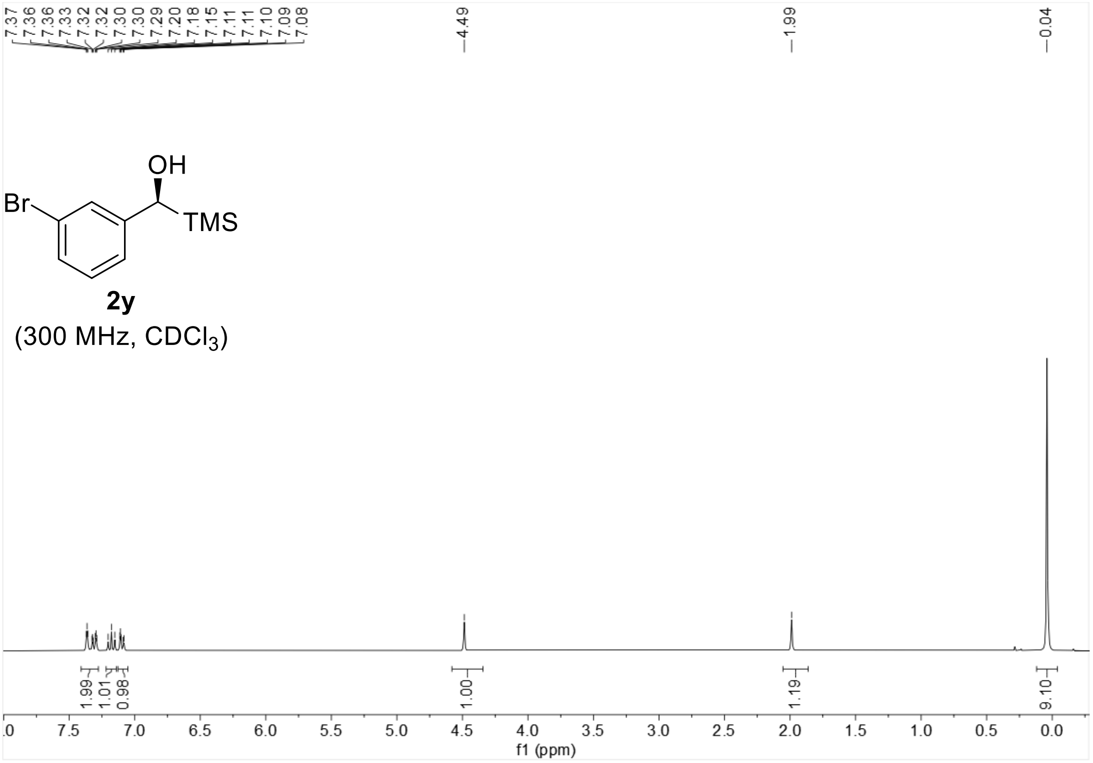
7.37
7.36
7.36
7.33
7.32
7.32
7.30
7.30
7.29
7.20
7.18
7.15
7.11
7.11
7.10
7.09
7.08
4.49
1.99
0.04
OH
Br
TMS
2y
(300 MHz, CDCl3)
1.99
1.01
0.98
1.00
1.19
9.10
f1 (ppm)

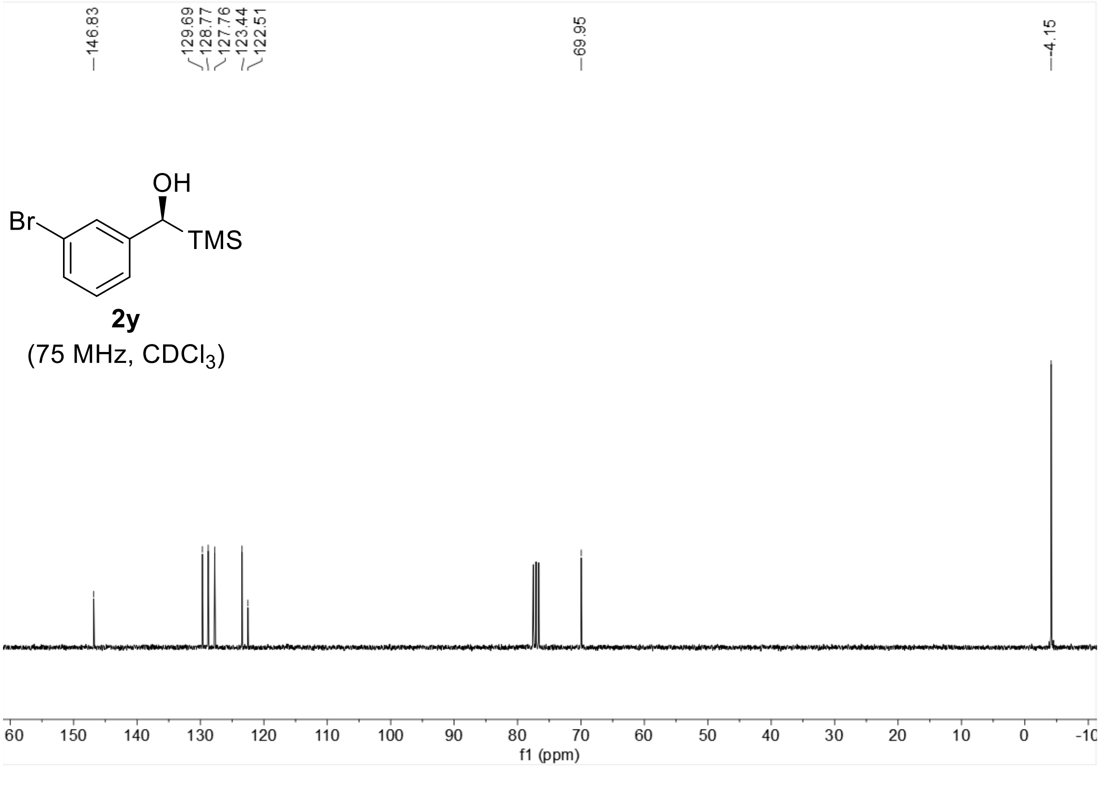
146.83
129.69
128.77
127.76
123.44
122.51
69.95
-4.15
OH
Br
TMS
2y
(75 MHz, CDCl3)
f1 (ppm)

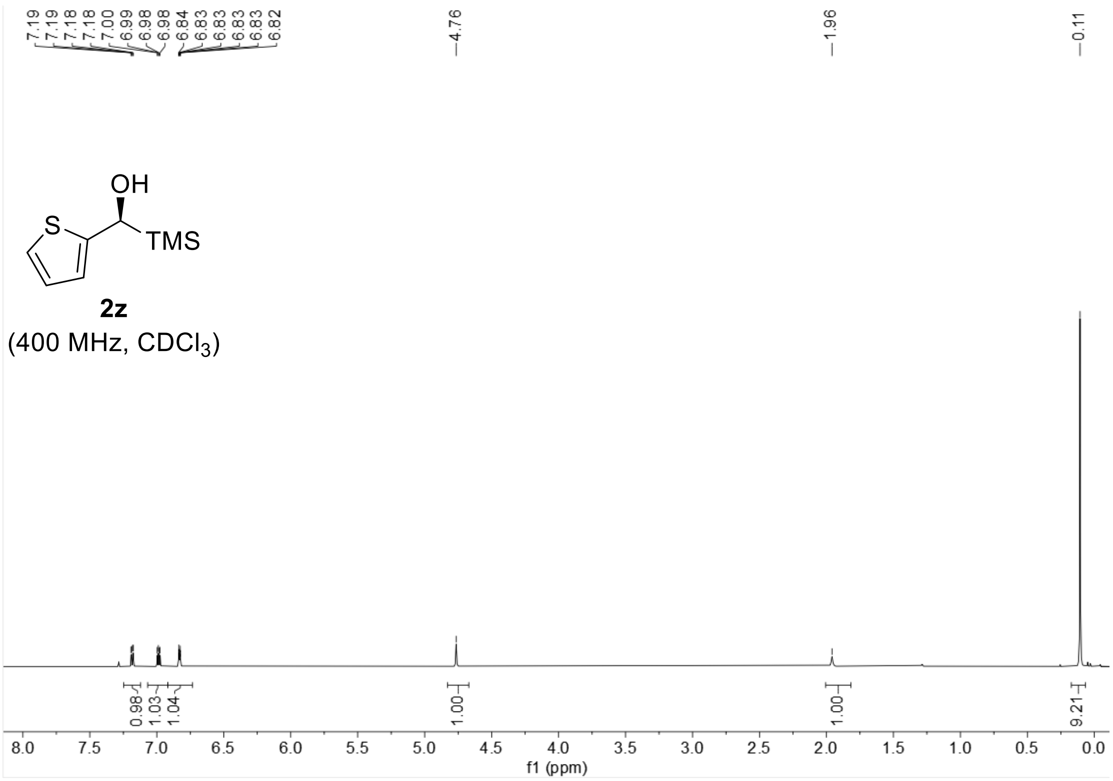

2z

(400 MHz, $CDCl_3$)

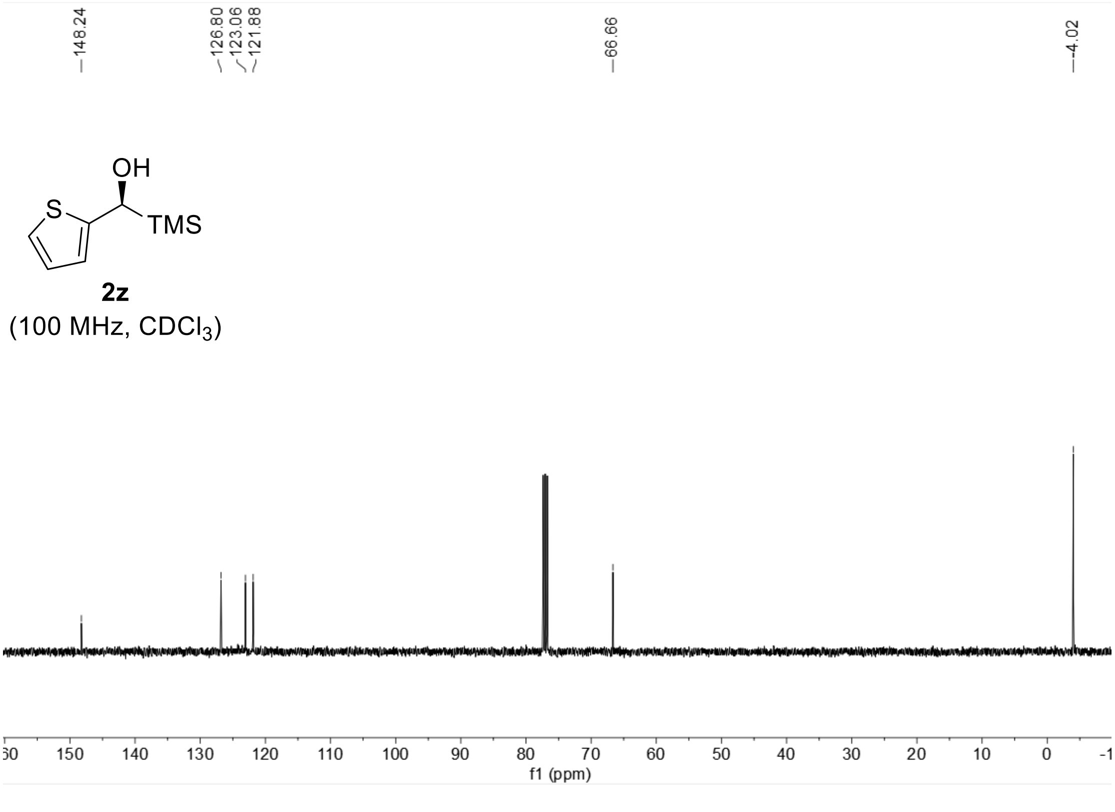

2z

(100 MHz, $CDCl_3$)

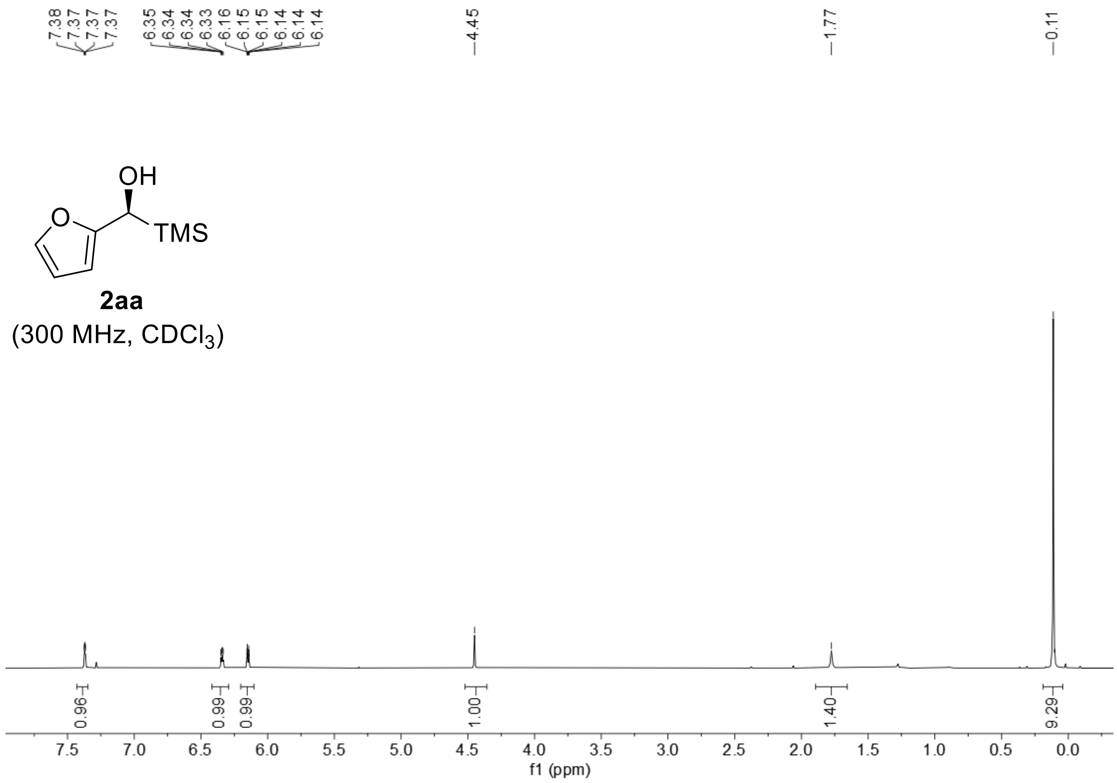
7.38 7.37 7.37 7.37
6.35 6.34 6.34 6.33 6.16 6.15 6.15 6.14 6.14 6.14
4.45
1.77
0.11
OH
O
TMS
2aa
(300 MHz, $CDCl_3$)
0.96
0.99
0.99
1.00
1.40
9.29
7.5 7.0 6.5 6.0 5.5 5.0 4.5 4.0 3.5 3.0 2.5 2.0 1.5 1.0 0.5 0.0
f1 (ppm)

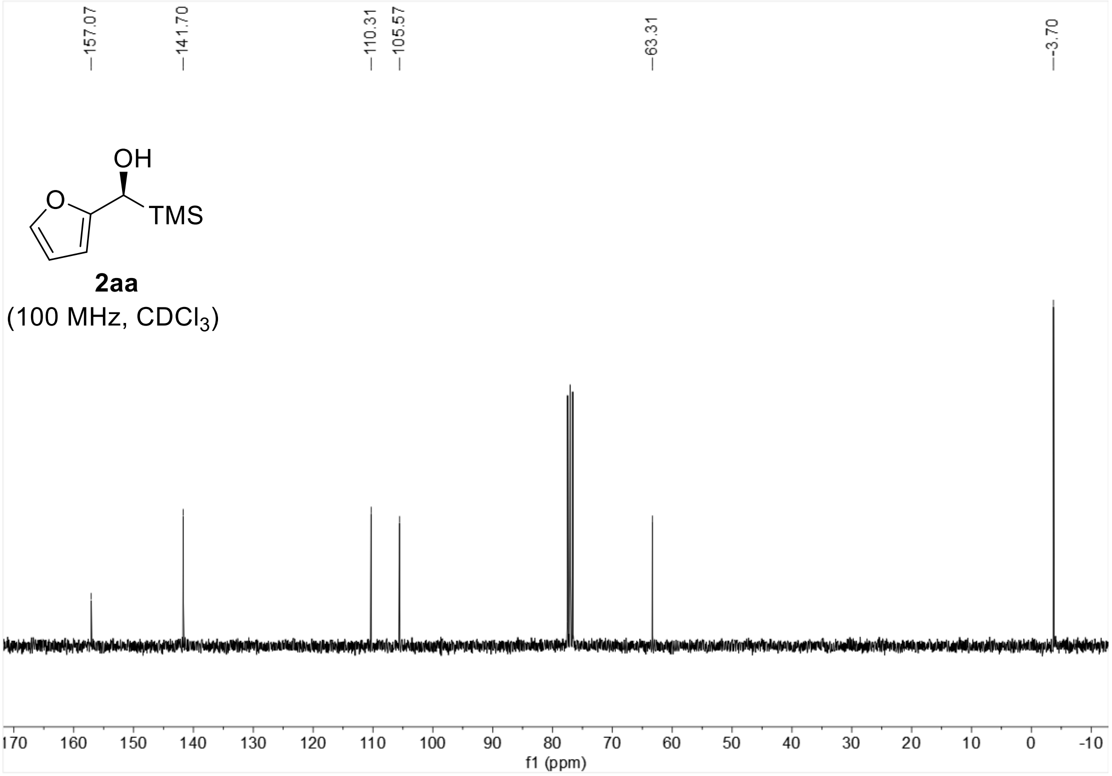
157.07
141.70
110.31 105.57
63.31
-3.70
OH
O
TMS
2aa
(100 MHz, $CDCl_3$)
170 160 150 140 130 120 110 100 90 80 70 60 50 40 30 20 10 0 -10
f1 (ppm)

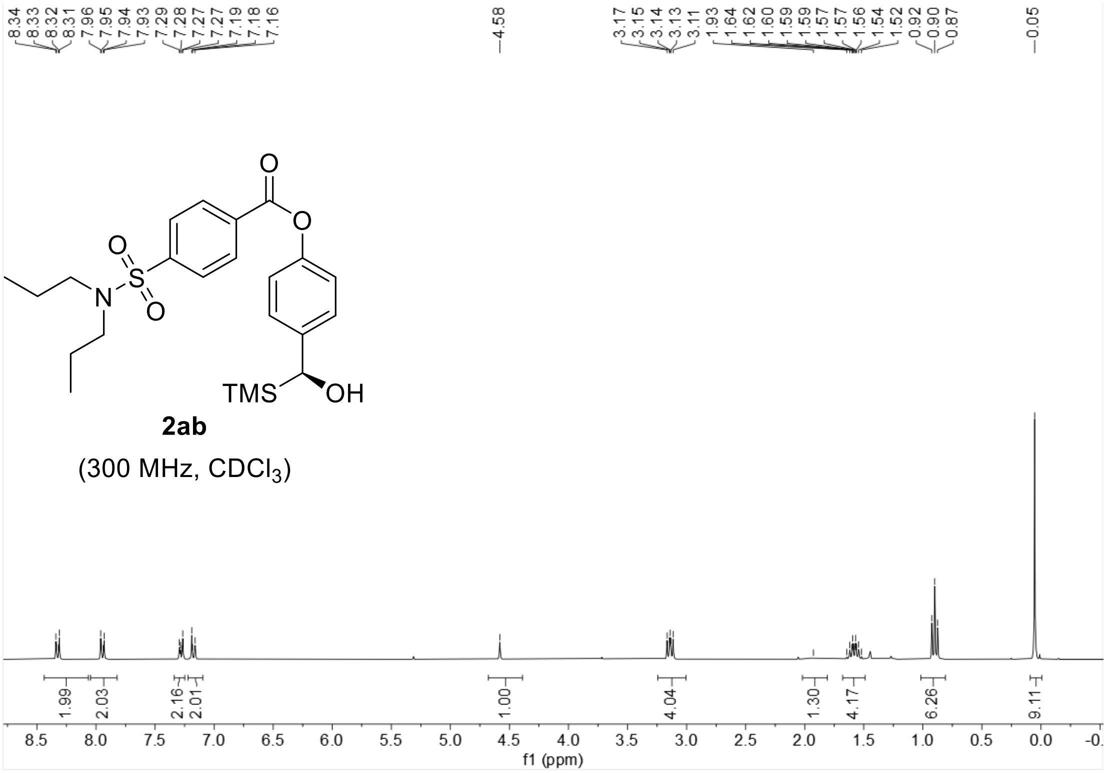

2ab

(300 MHz, $CDCl_3$)

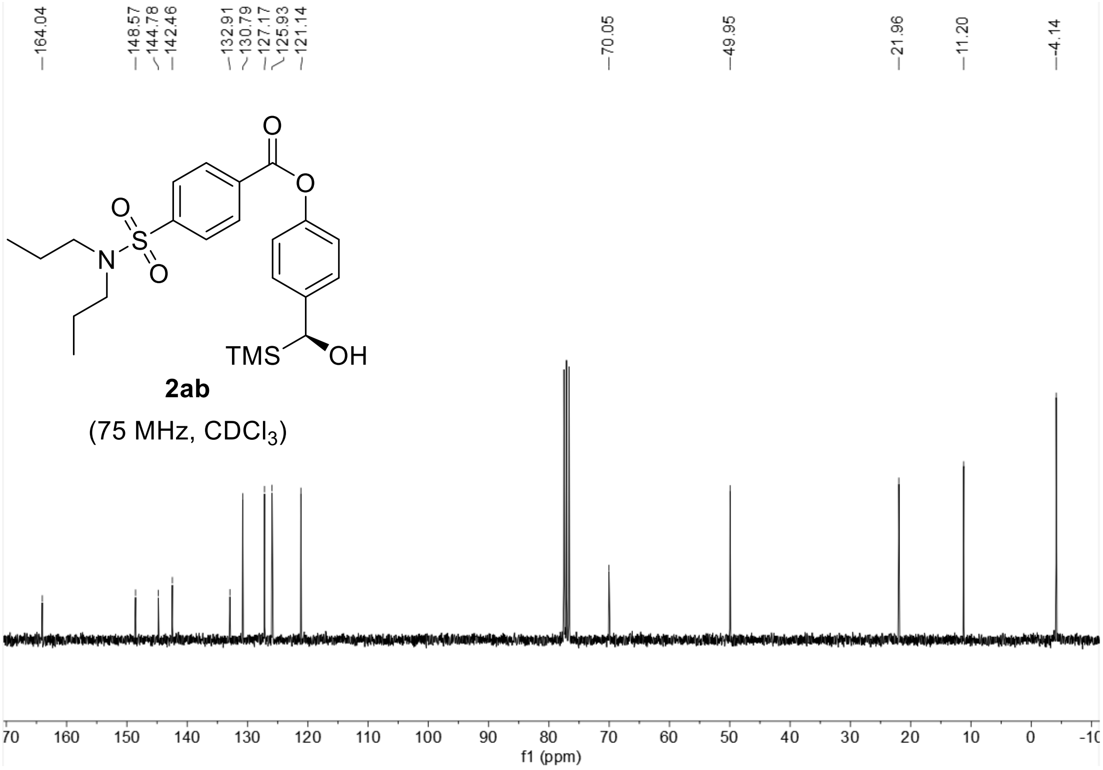

2ab

(75 MHz, $CDCl_3$)

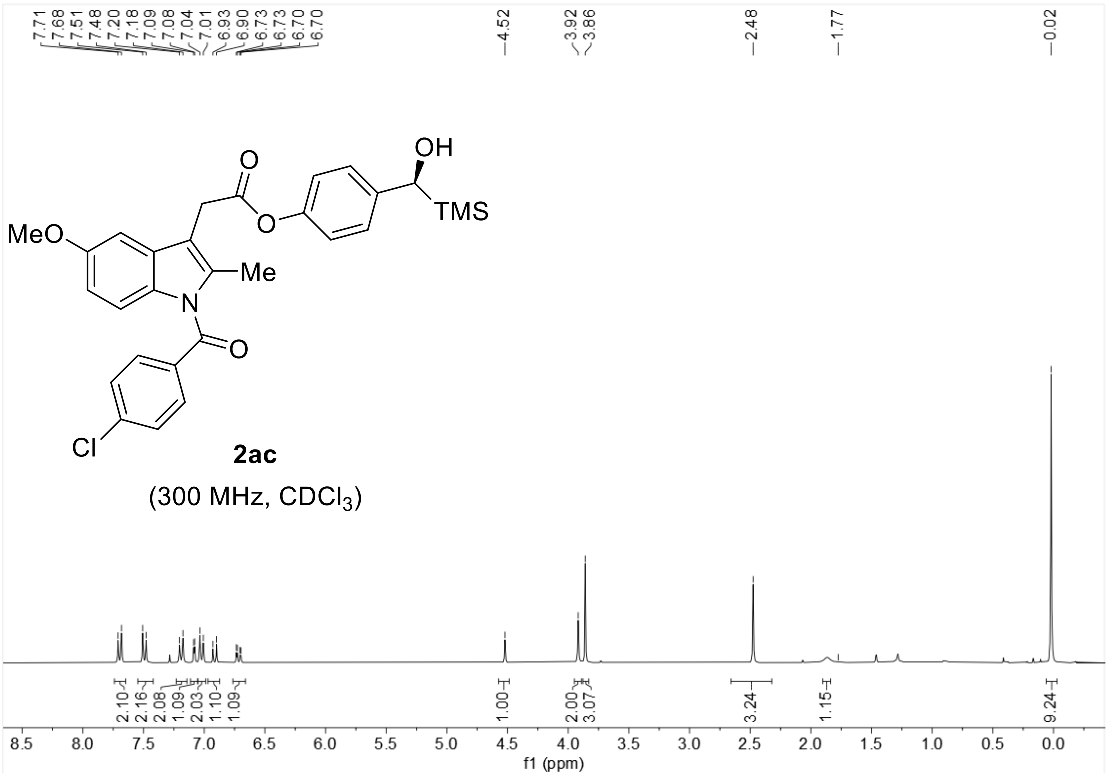

2ac
(300 MHz, $CDCl_3$)

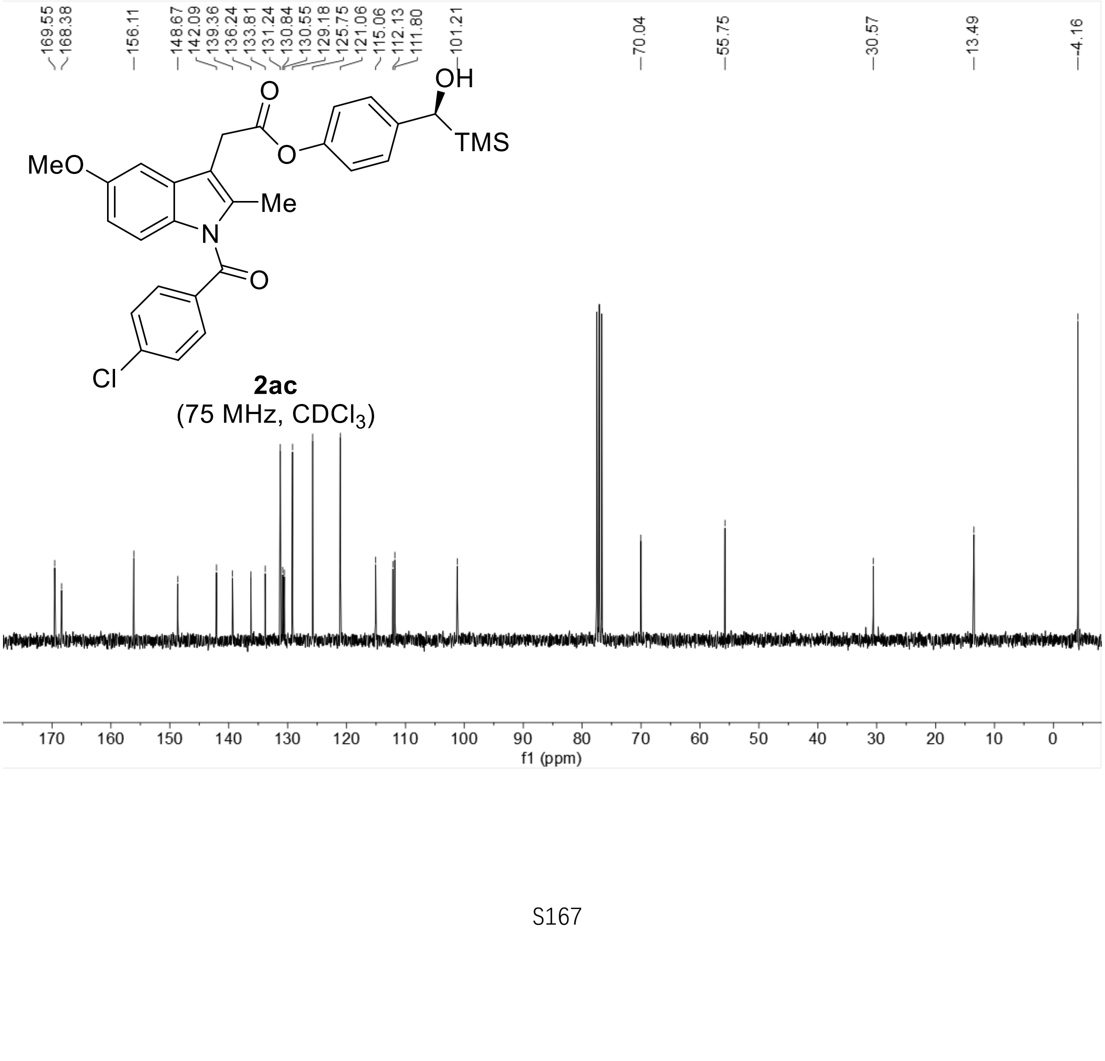

2ac
(75 MHz, $CDCl_3$)

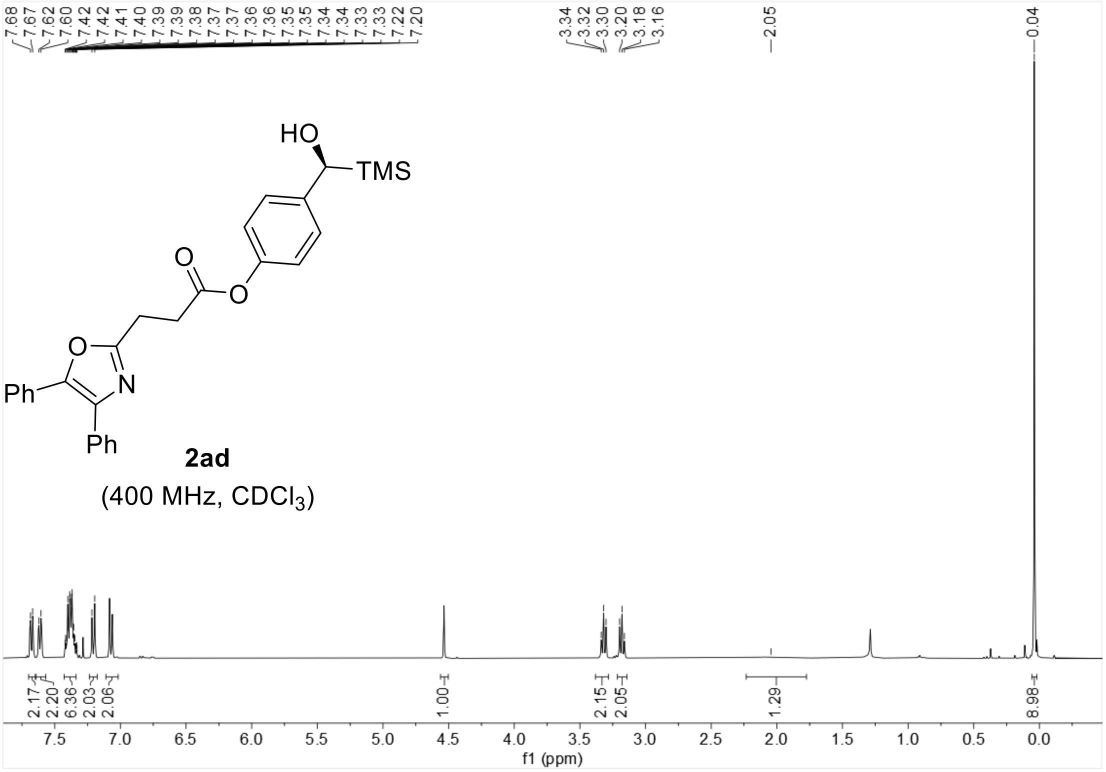

2ad
(400 MHz, CDCl3)

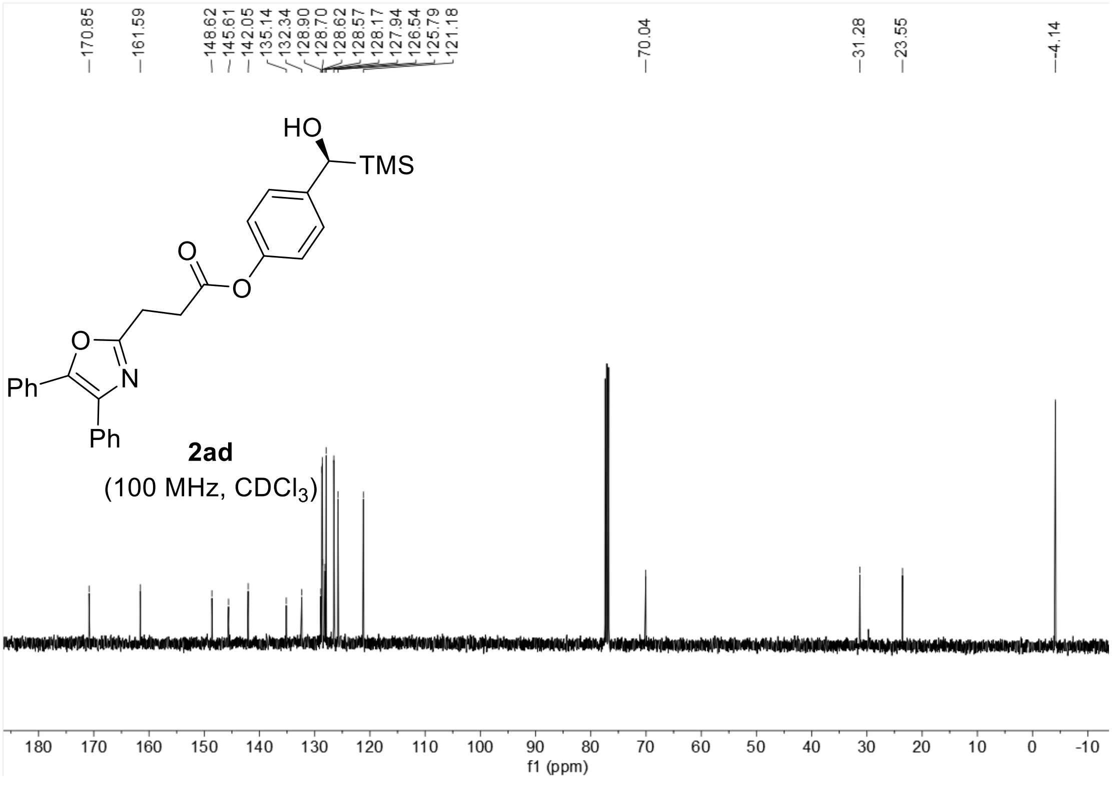

2ad
(100 MHz, CDCl3)

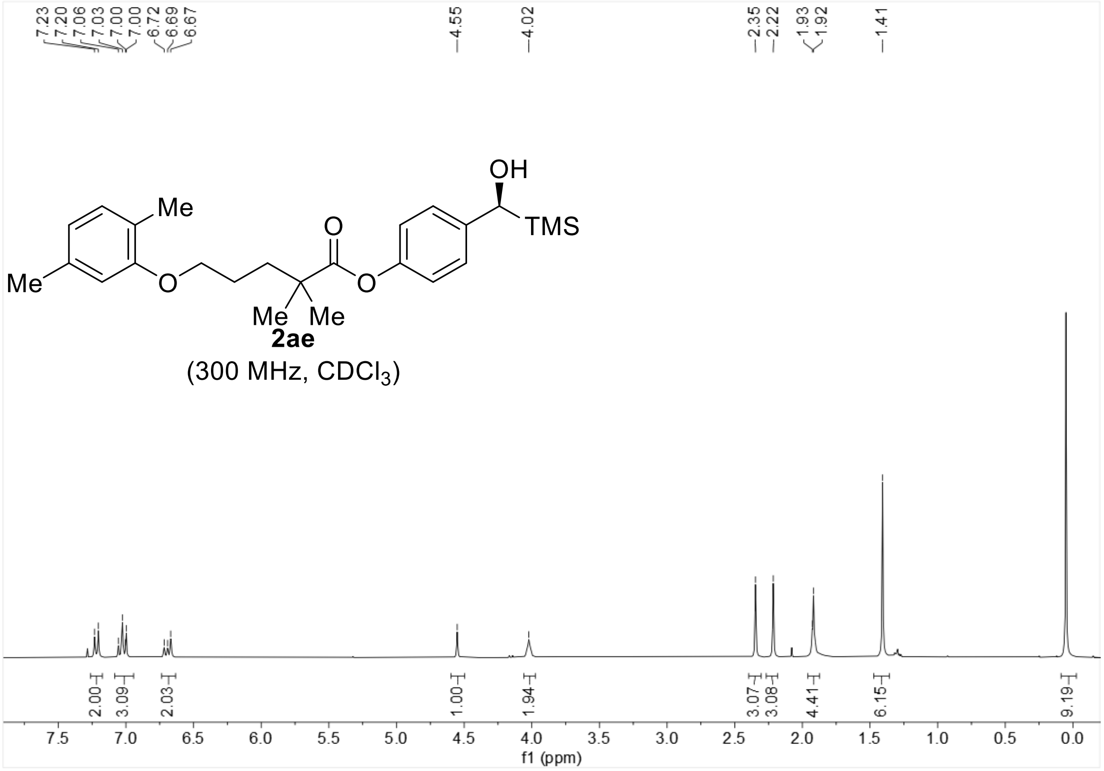
7.23
7.20
7.06
7.03
7.00
7.00
6.72
6.69
6.67
4.55
4.02
2.35
2.22
1.93
1.92
1.41
OH
TMS
Me
O
Me
O
O
Me
Me
2ae
(300 MHz, CDCl3)
2.00
3.09
2.03
1.00
1.94
3.07
3.08
4.41
6.15
9.19
f1 (ppm)

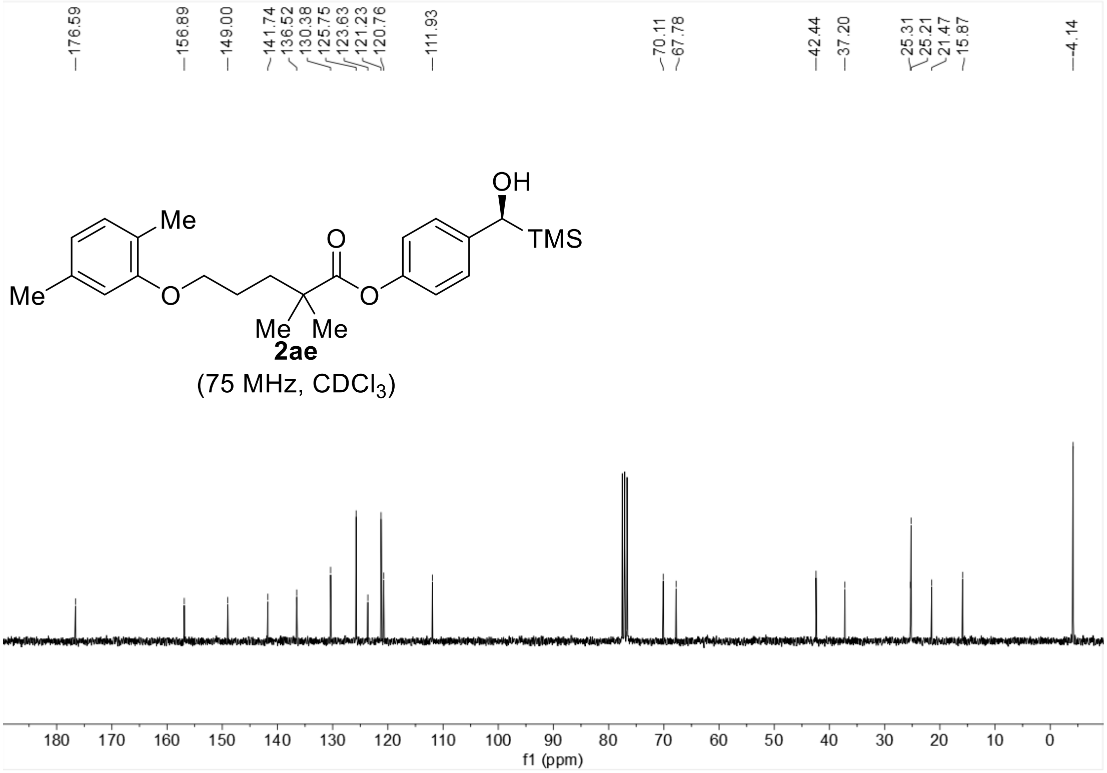
176.59
156.89
149.00
141.74
136.52
130.38
125.75
123.63
121.23
120.76
111.93
70.11
67.78
42.44
37.20
25.31
25.21
21.47
15.87
-4.14
OH
TMS
Me
O
Me
O
O
Me
Me
2ae
(75 MHz, CDCl3)
f1 (ppm)

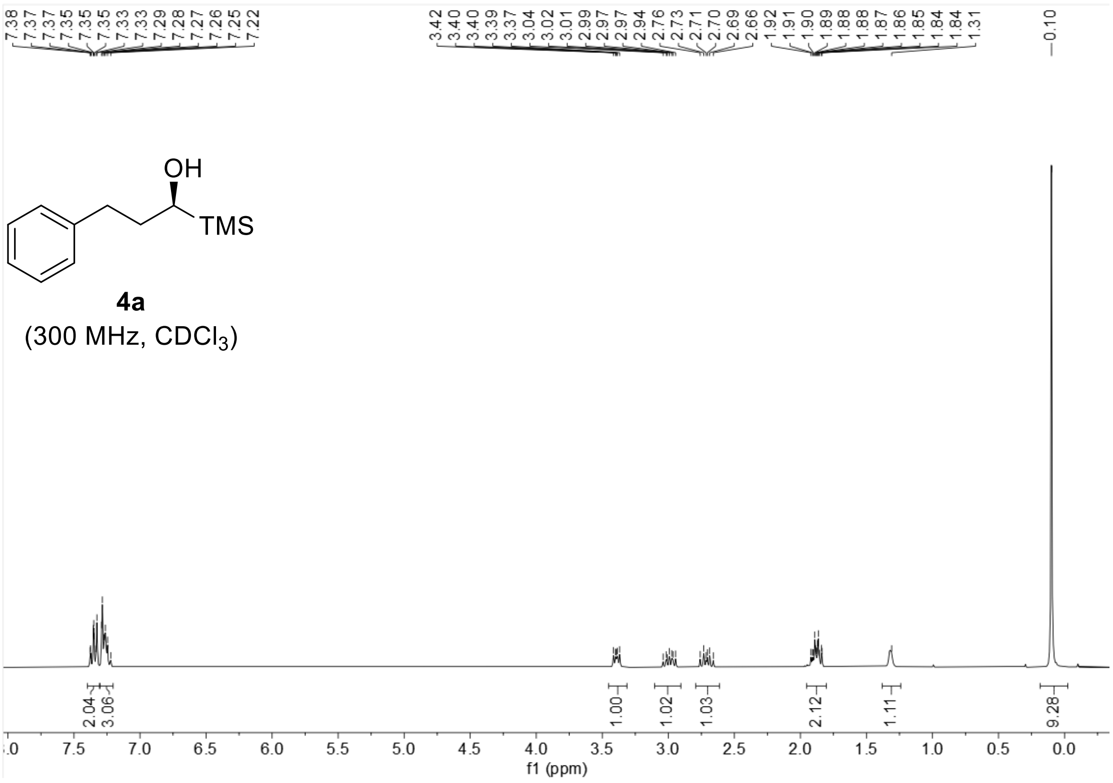
7.38
7.37
7.37
7.35
7.35
7.35
7.33
7.33
7.29
7.28
7.27
7.26
7.25
7.22
3.42
3.40
3.40
3.39
3.37
3.04
3.02
3.01
2.99
2.97
2.97
2.94
2.76
2.73
2.71
2.70
2.69
2.66
1.92
1.91
1.90
1.89
1.88
1.88
1.87
1.86
1.85
1.84
1.84
1.31
0.10
OH
TMS
4a
(300 MHz, CDCl3)
2.04
3.06
1.00
1.02
1.03
2.12
1.11
9.28
7.5
7.0
6.5
6.0
5.5
5.0
4.5
4.0
3.5
3.0
2.5
2.0
1.5
1.0
0.5
0.0
f1 (ppm)

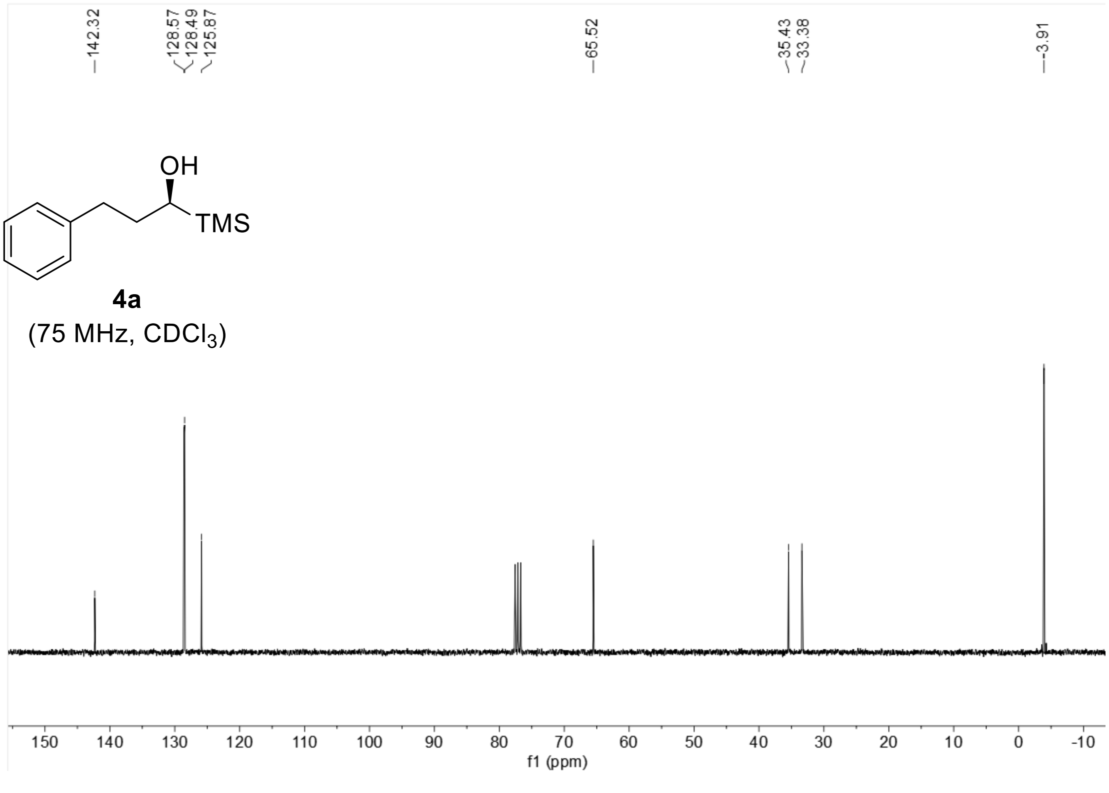
142.32
128.57
128.49
125.87
65.52
35.43
33.38
-3.91
OH
TMS
4a
(75 MHz, CDCl3)
150
140
130
120
110
100
90
80
70
60
50
40
30
20
10
0
-10
f1 (ppm)

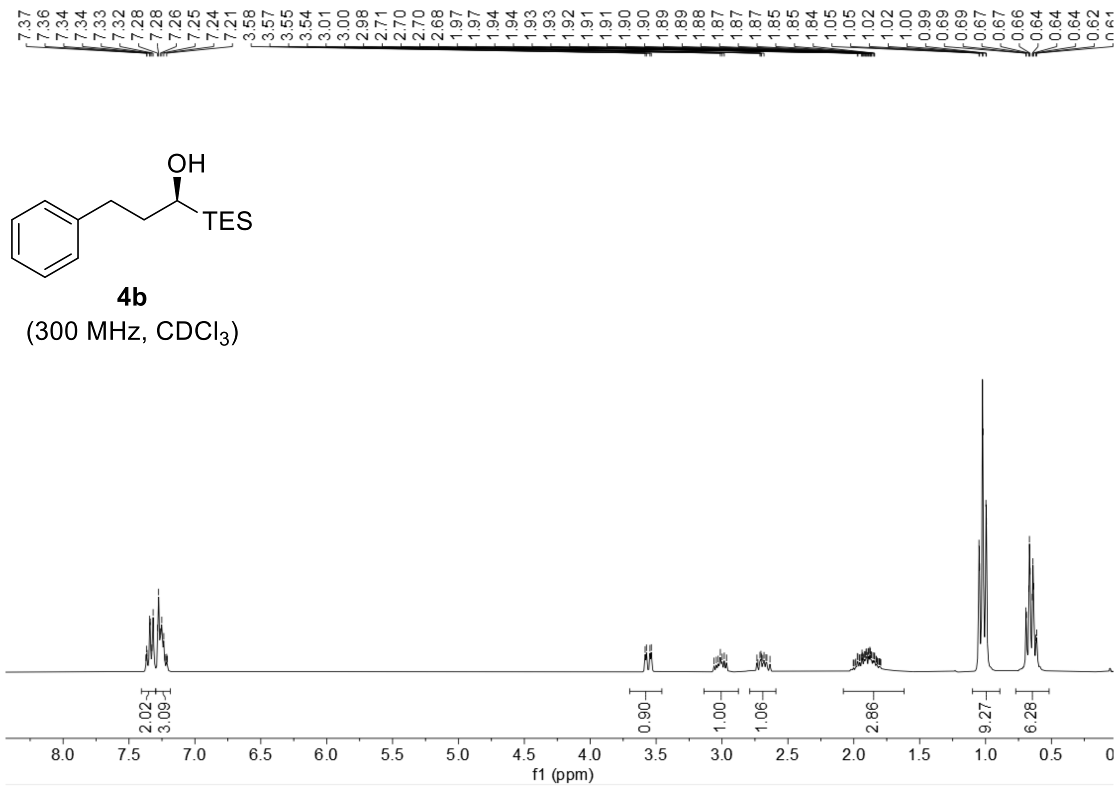

4b
(300 MHz, $CDCl_3$)

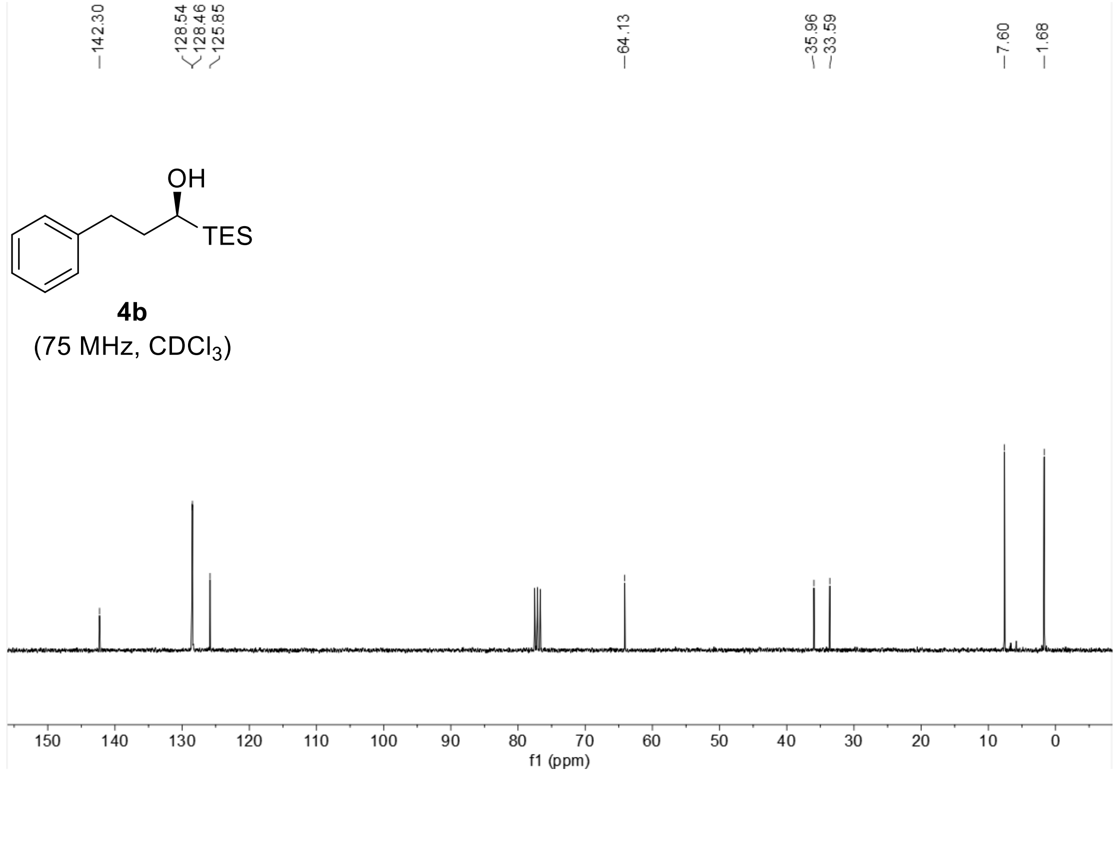

4b
(75 MHz, $CDCl_3$)

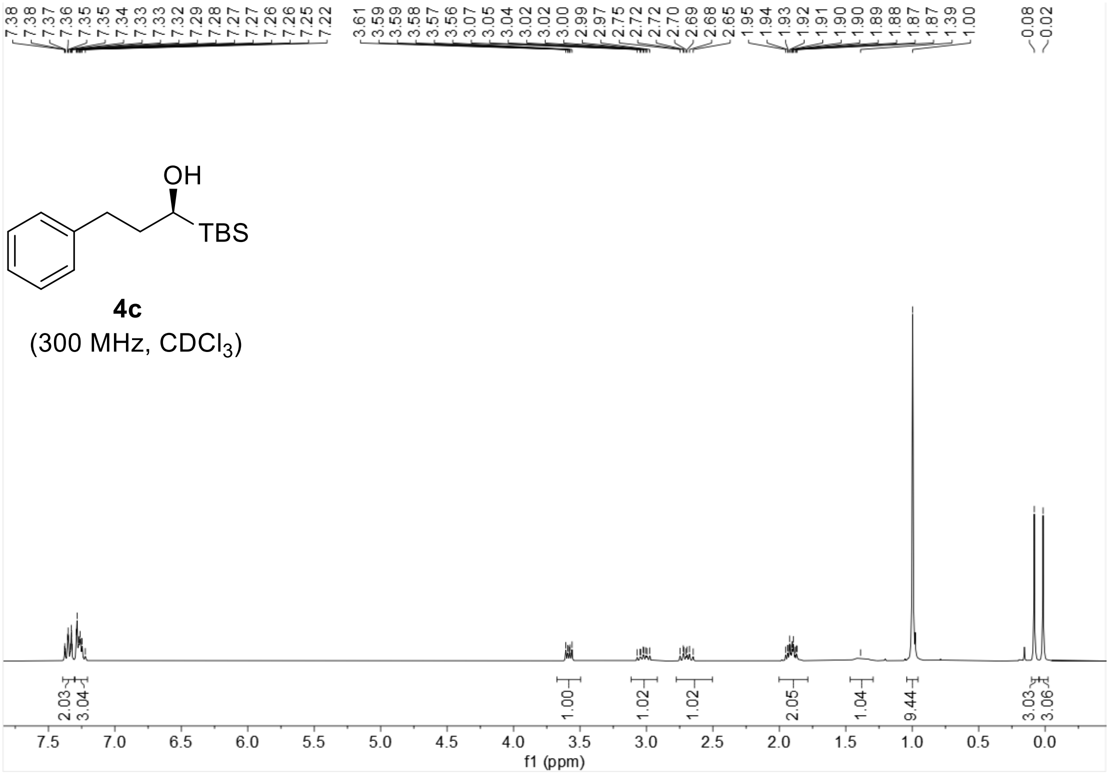
OH
TBS
4c
(300 MHz, CDCl3)
f1 (ppm)

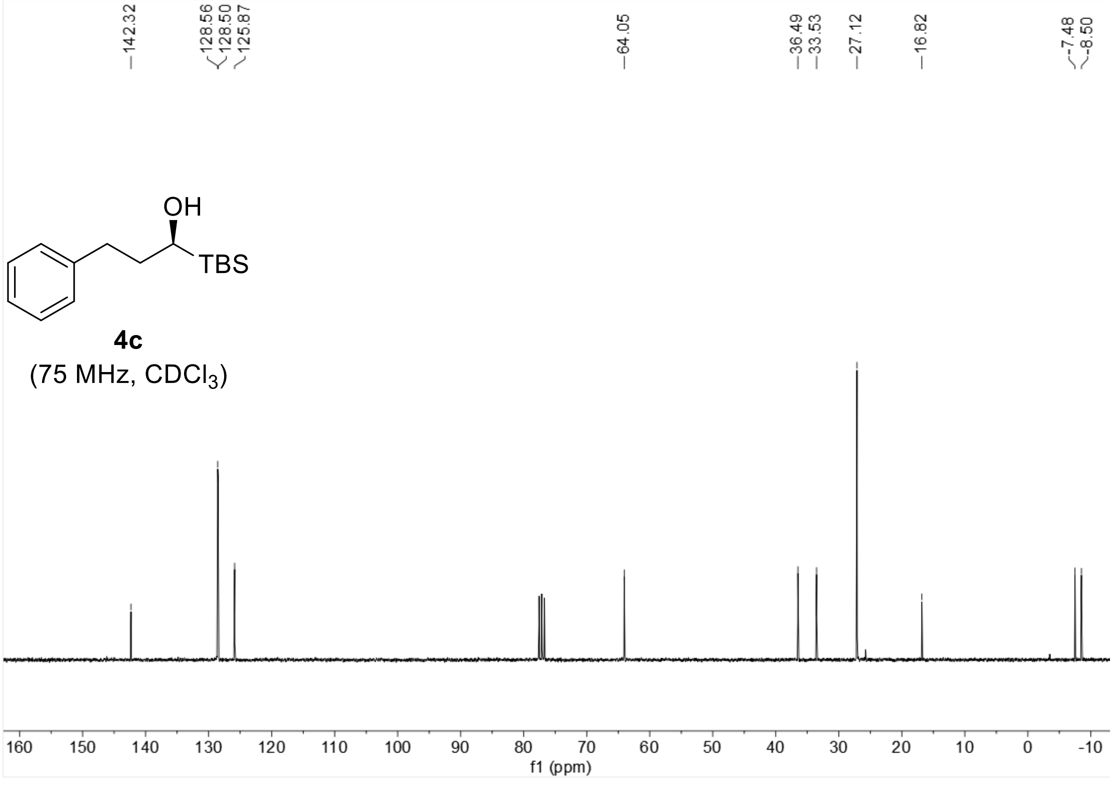
OH
TBS
4c
(75 MHz, CDCl3)
f1 (ppm)

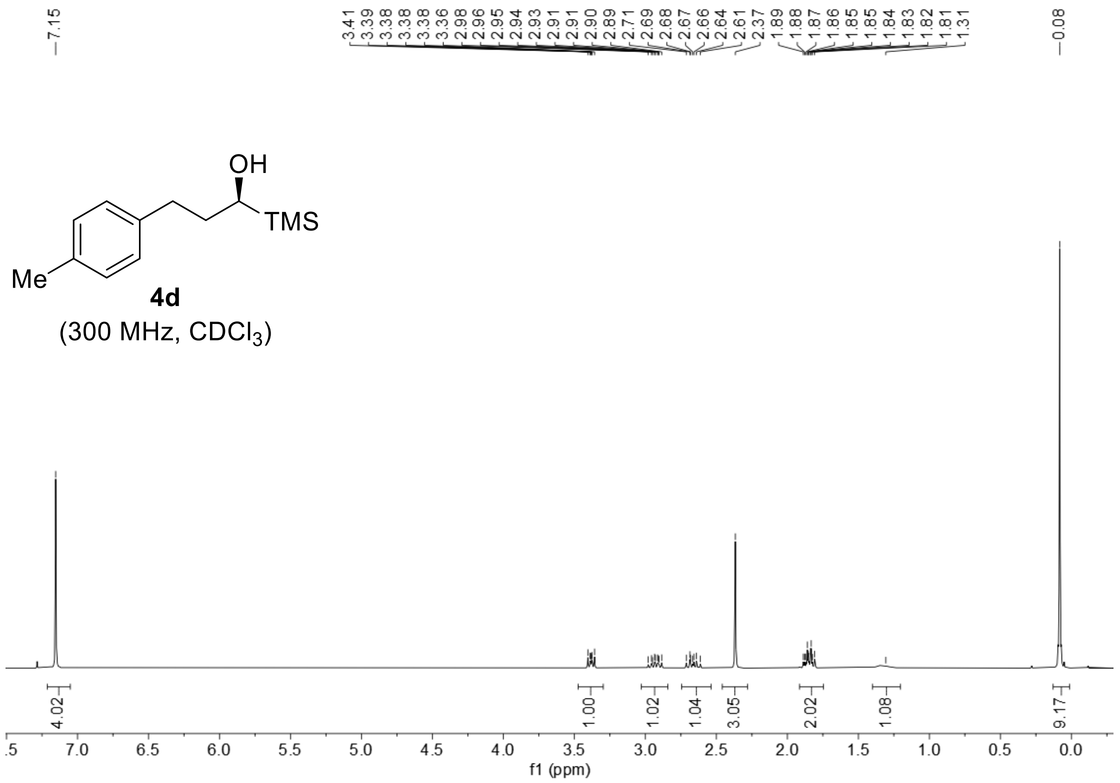

4d

(300 MHz, $CDCl_3$)

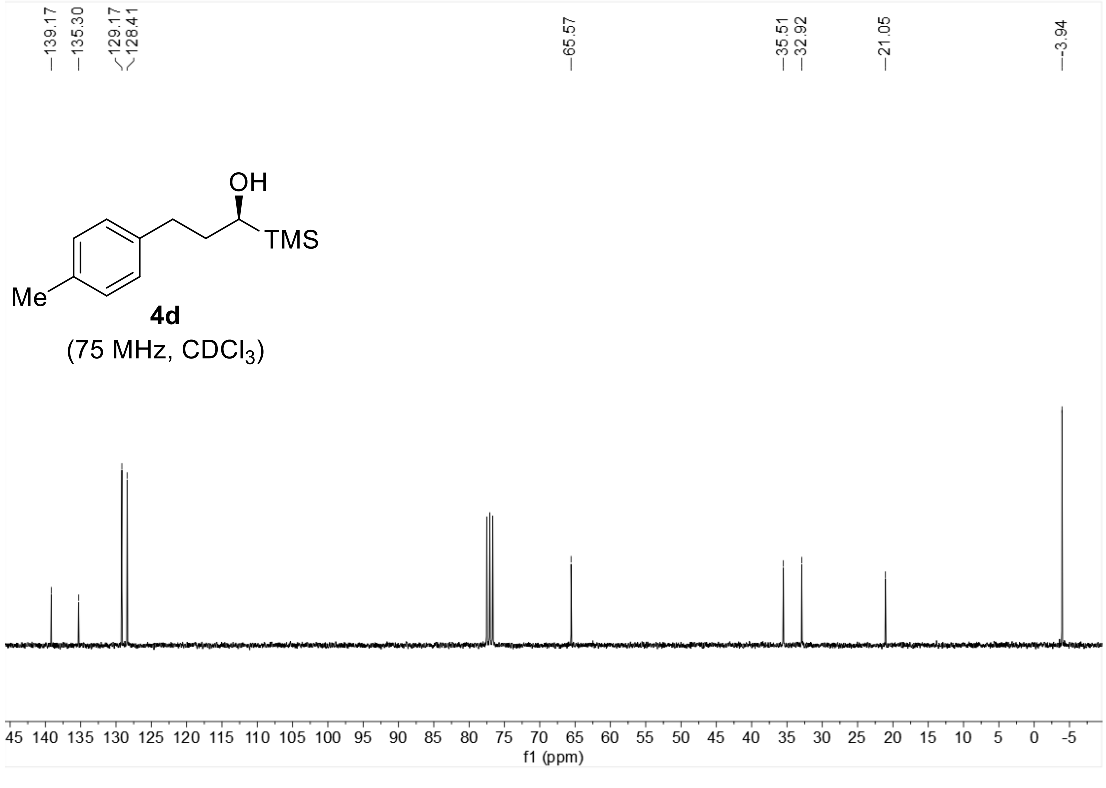

4d

(75 MHz, $CDCl_3$)

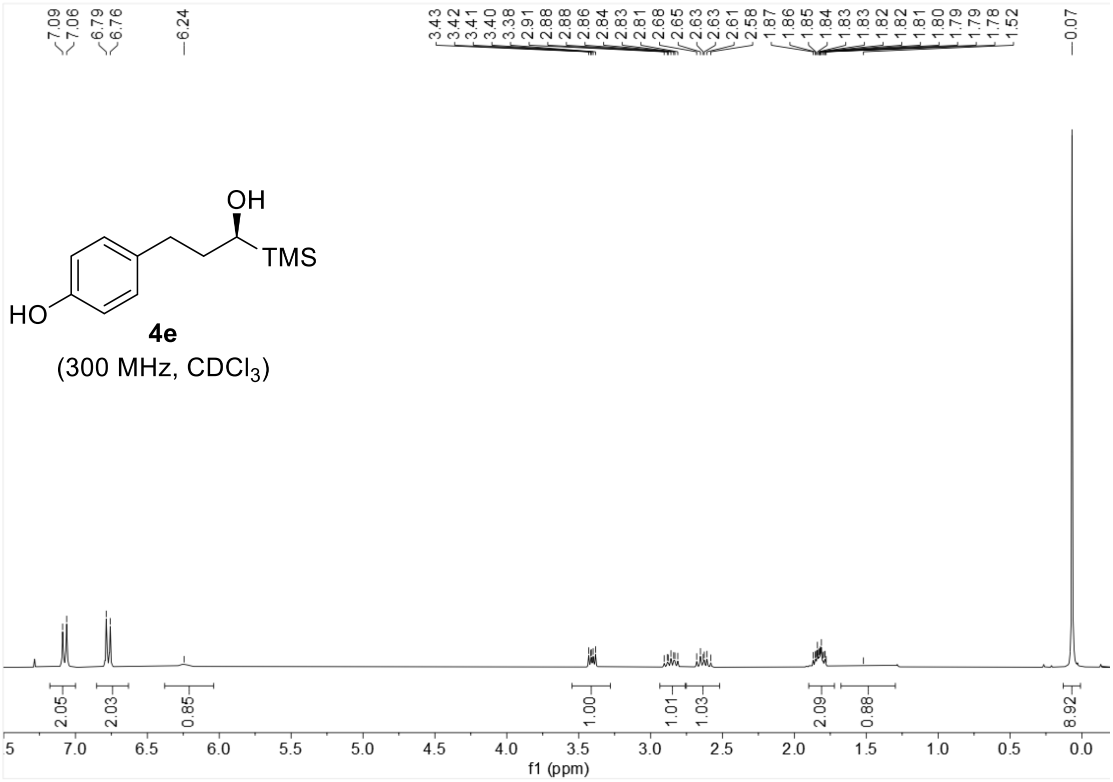
7.09 7.06 6.79 6.76 6.24
3.43 3.42 3.41 3.40 3.38 2.91 2.88 2.88 2.86 2.84 2.83 2.81 2.68 2.65 2.63 2.63 2.61 2.58 1.87 1.86 1.85 1.84 1.83 1.83 1.82 1.82 1.81 1.80 1.79 1.79 1.78 1.52
0.07
OH
TMS
HO
4e
(300 MHz, CDCl3)
2.05 2.03 0.85 1.00 1.01 1.03 2.09 0.88 8.92
f1 (ppm)

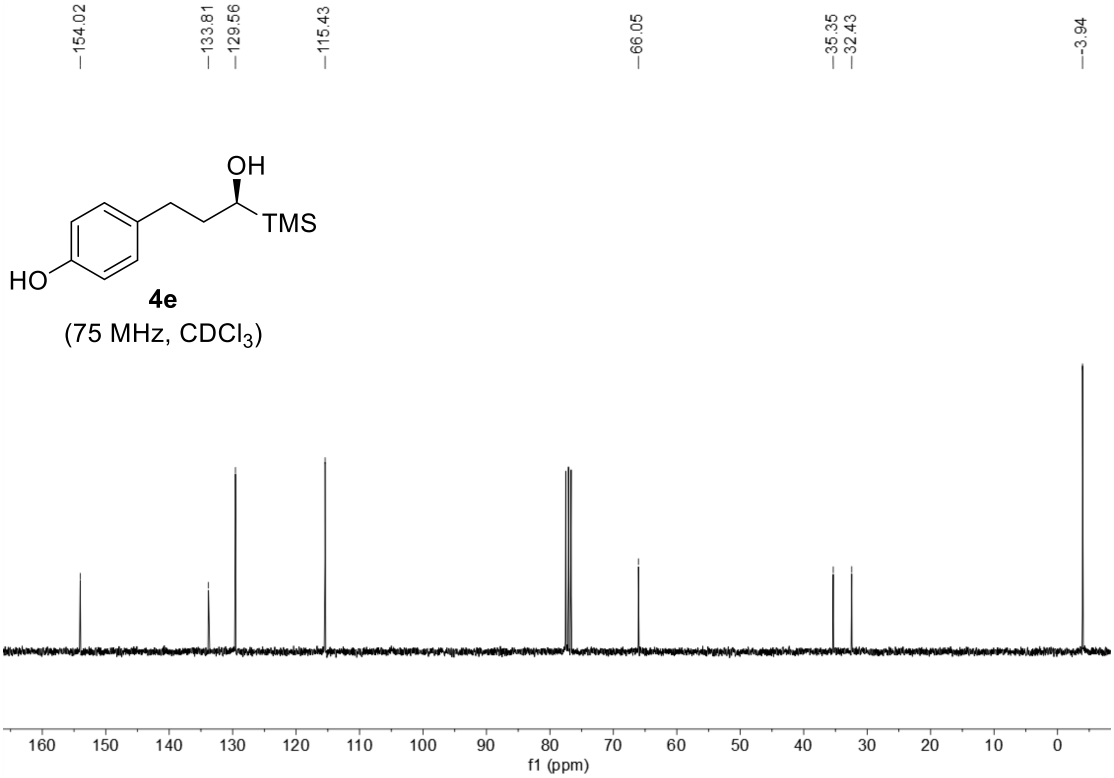
154.02 133.81 129.56 115.43 66.05 35.35 32.43 -3.94
OH
TMS
HO
4e
(75 MHz, CDCl3)
f1 (ppm)

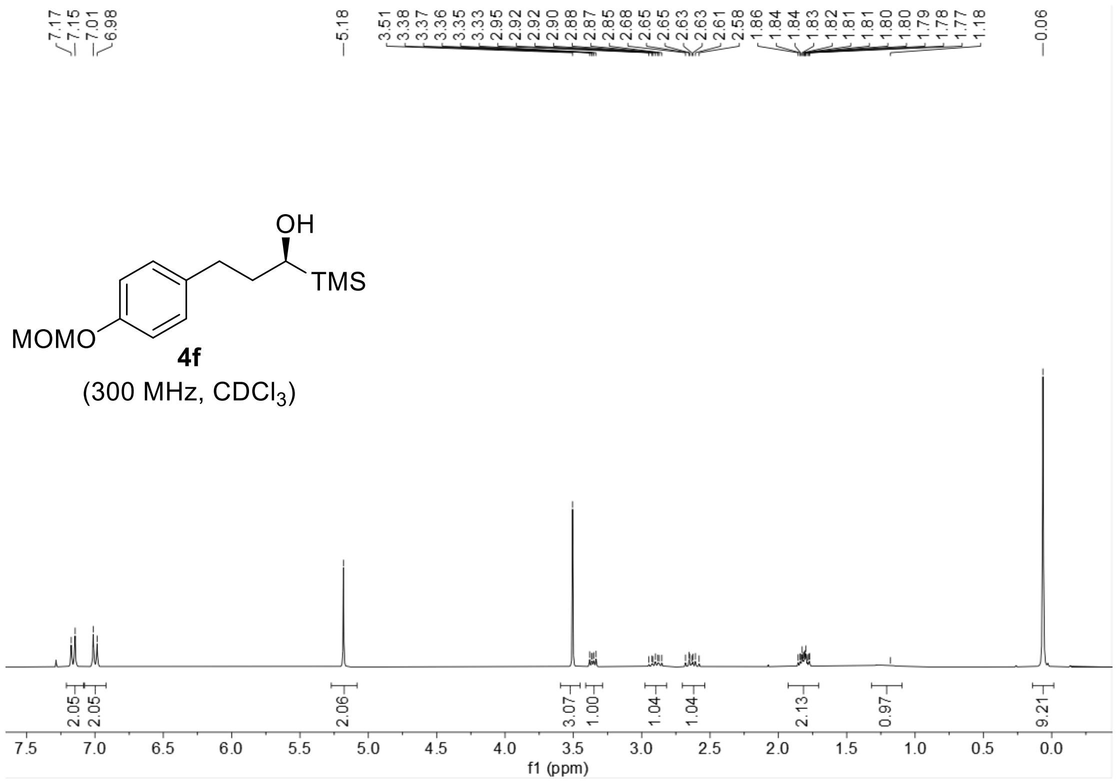
OH
TMS
MOMO
4f
(300 MHz, CDCl3)
f1 (ppm)

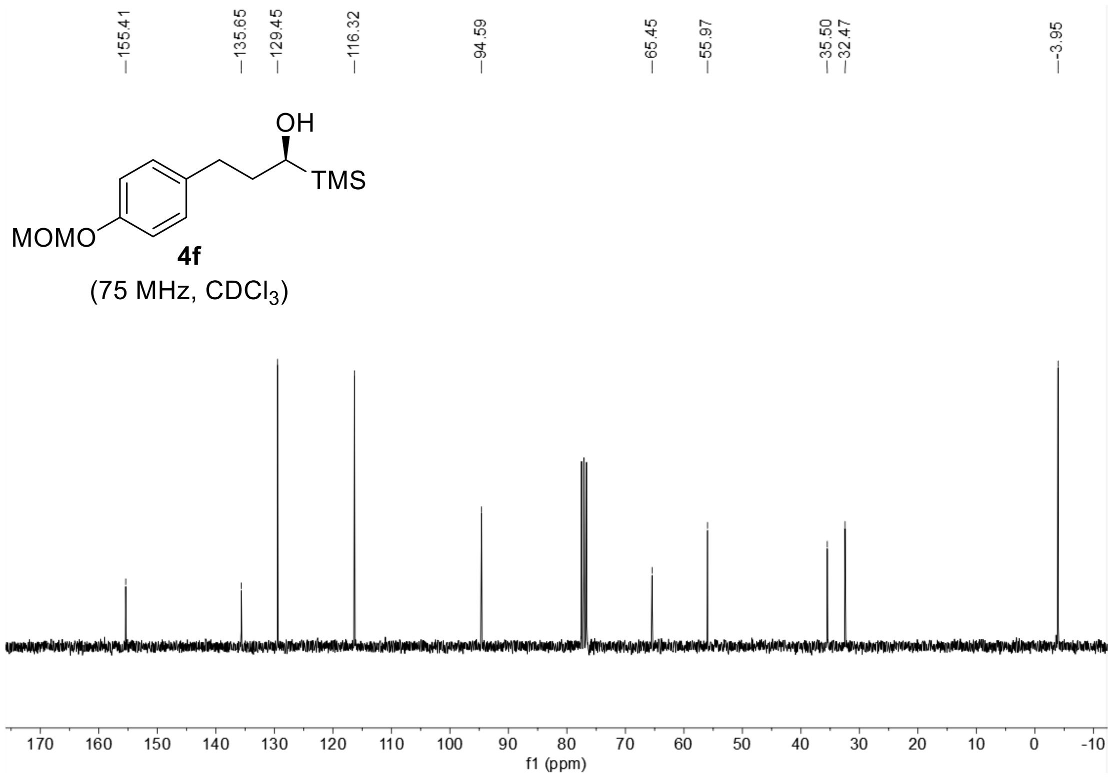
OH
TMS
MOMO
4f
(75 MHz, CDCl3)
f1 (ppm)

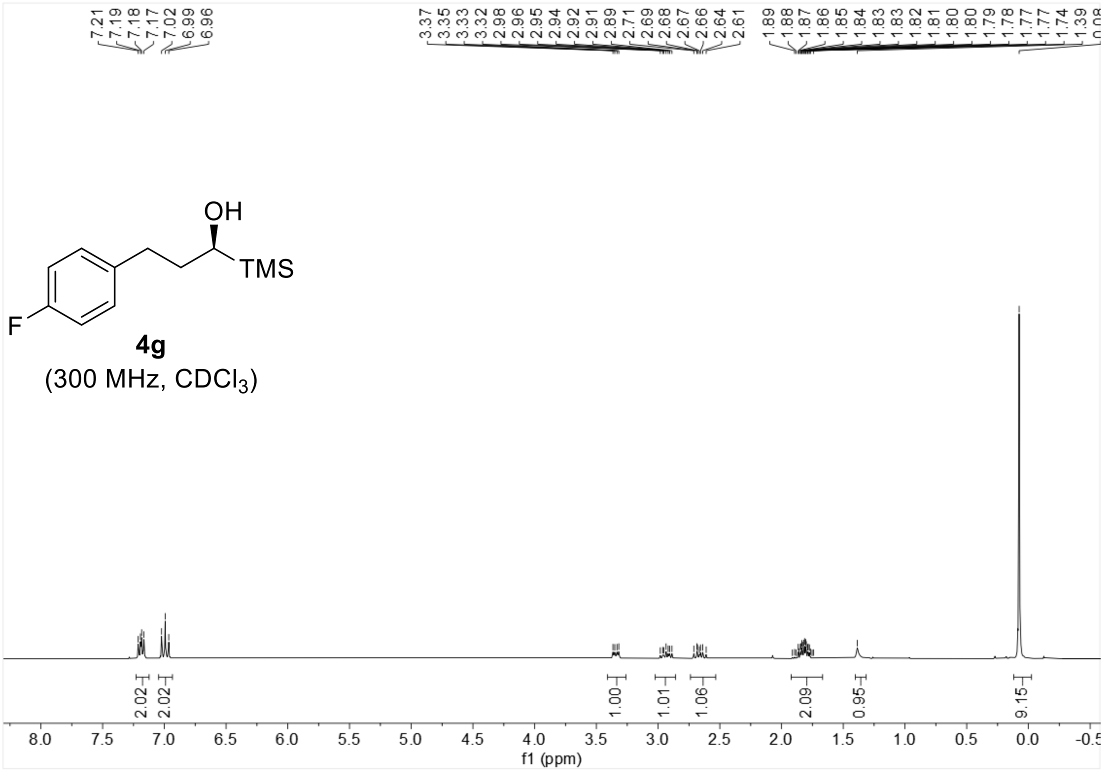
7.21
7.19
7.18
7.17
7.02
6.99
6.96
3.37
3.35
3.33
3.32
2.98
2.96
2.95
2.94
2.92
2.91
2.89
2.71
2.69
2.68
2.67
2.66
2.64
2.61
1.89
1.88
1.87
1.86
1.85
1.84
1.83
1.83
1.82
1.81
1.80
1.80
1.79
1.78
1.77
1.77
1.74
1.39
OH
TMS
F
4g
(300 MHz, CDCl3)
2.02
2.02
1.00
1.01
1.06
2.09
0.95
9.15
f1 (ppm)

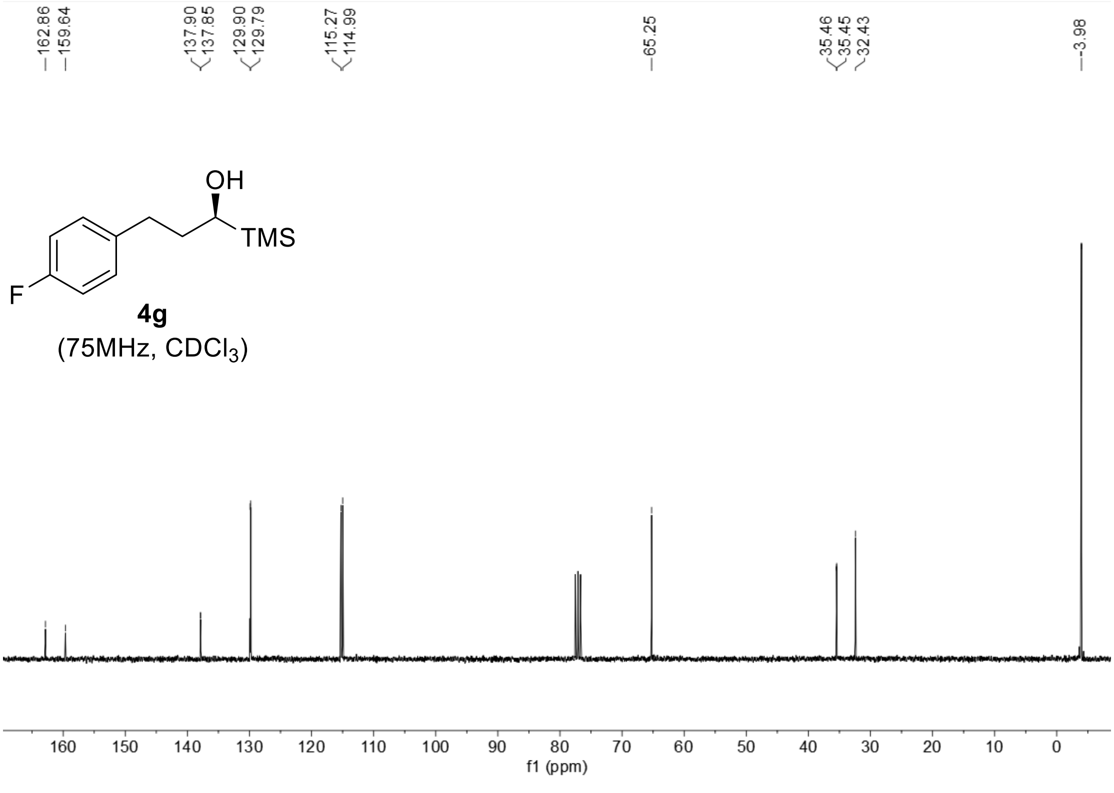
162.86
159.64
137.90
137.85
129.90
129.79
115.27
114.99
65.25
35.46
35.45
32.43
-3.98
OH
TMS
F
4g
(75MHz, CDCl3)
f1 (ppm)

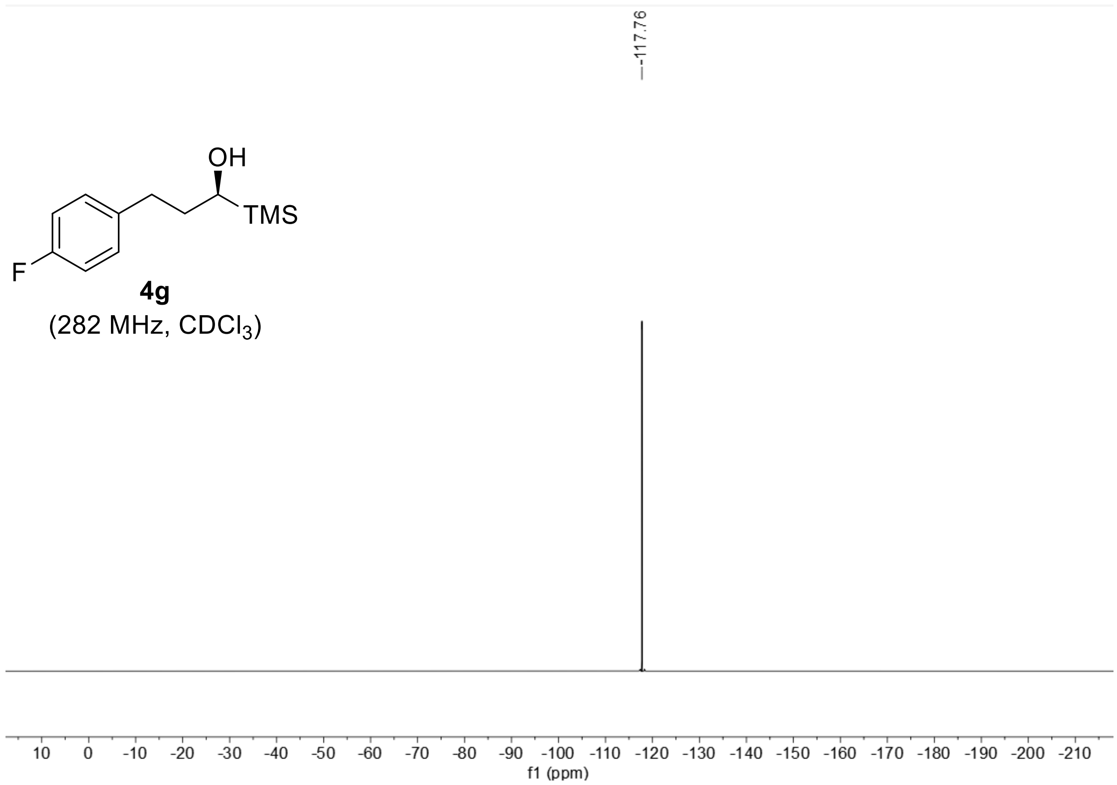
-117.76
OH
TMS
F
4g
(282 MHz, CDCl$_3$)
10 0 -10 -20 -30 -40 -50 -60 -70 -80 -90 -100 -110 -120 -130 -140 -150 -160 -170 -180 -190 -200 -210
f1 (ppm)

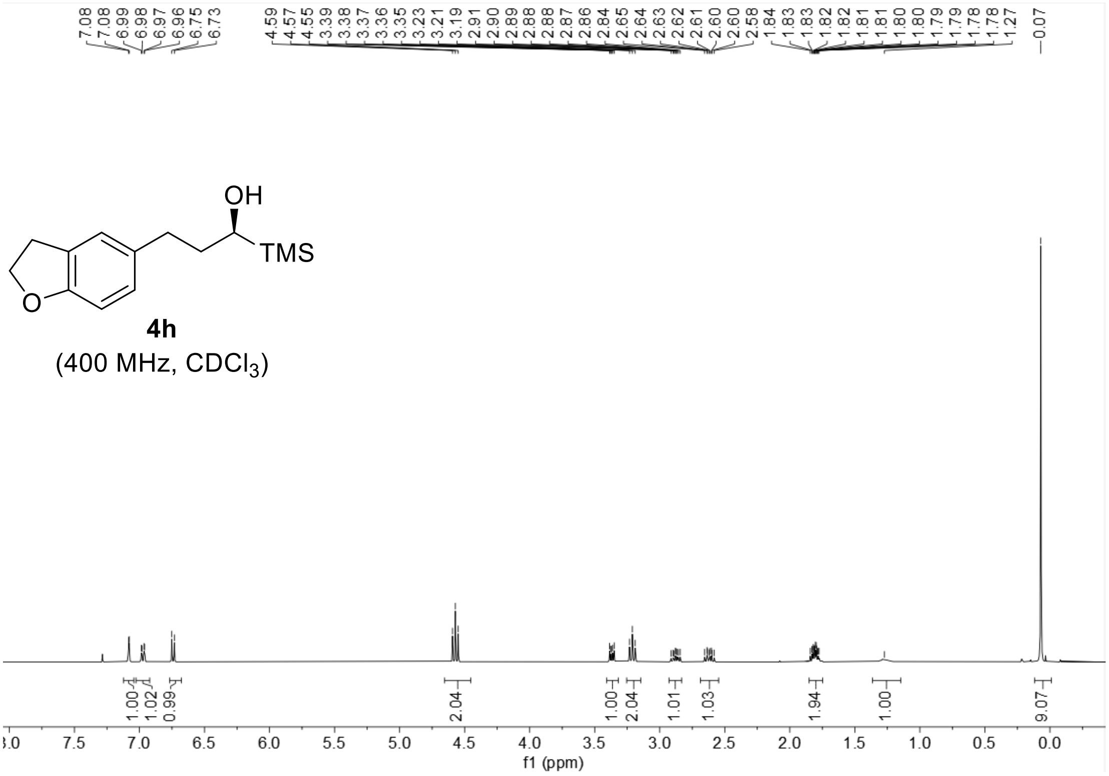

7.08 7.08 6.99 6.98 6.97 6.96 6.75 6.73
4.59 4.57 4.55 3.39 3.38 3.37 3.36 3.35 3.23 3.21 3.19 2.91 2.90 2.89 2.88 2.88 2.87 2.86 2.84 2.65 2.64 2.63 2.62 2.61 2.60 2.60 2.58 1.84 1.83 1.83 1.82 1.82 1.81 1.81 1.80 1.80 1.79 1.79 1.78 1.78 1.27
0.07
OH
TMS
O
4h
(400 MHz, CDCl3)
1.00 1.02 0.99 2.04 1.00 2.04 1.01 1.03 1.94 1.00 9.07
f1 (ppm)

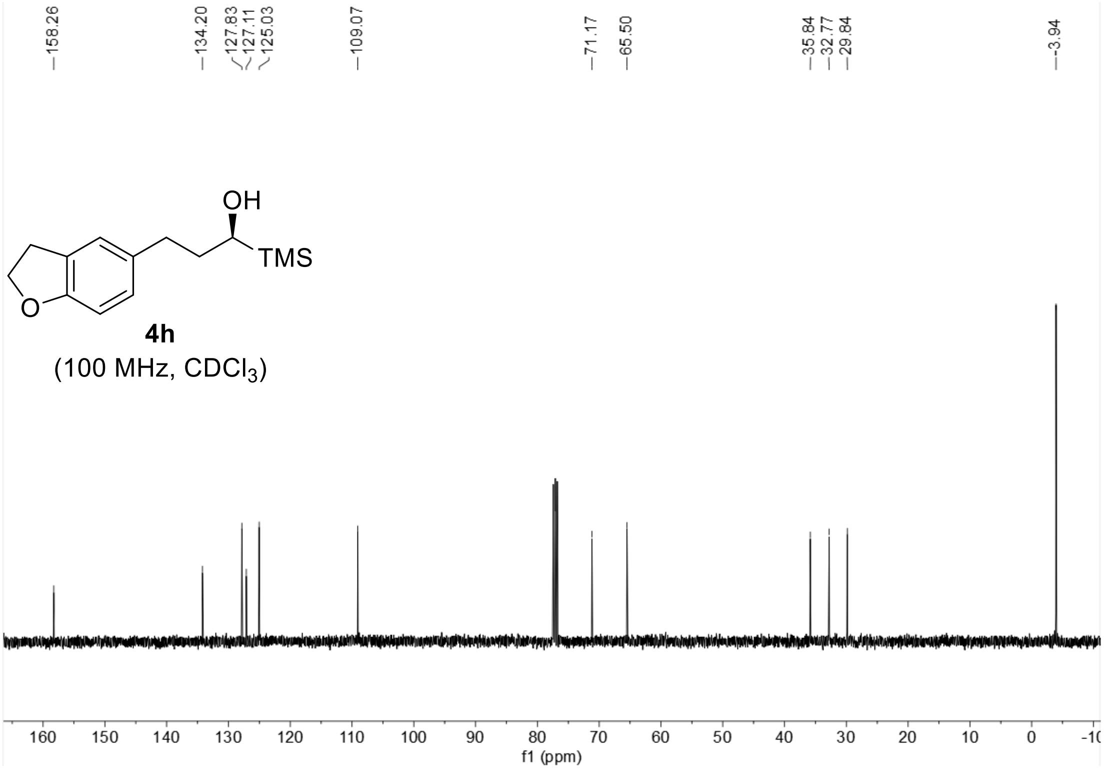

158.26
134.20 127.83 127.11 125.03
109.07
71.17 65.50
35.84 32.77 29.84
-3.94
OH
TMS
O
4h
(100 MHz, CDCl3)
f1 (ppm)

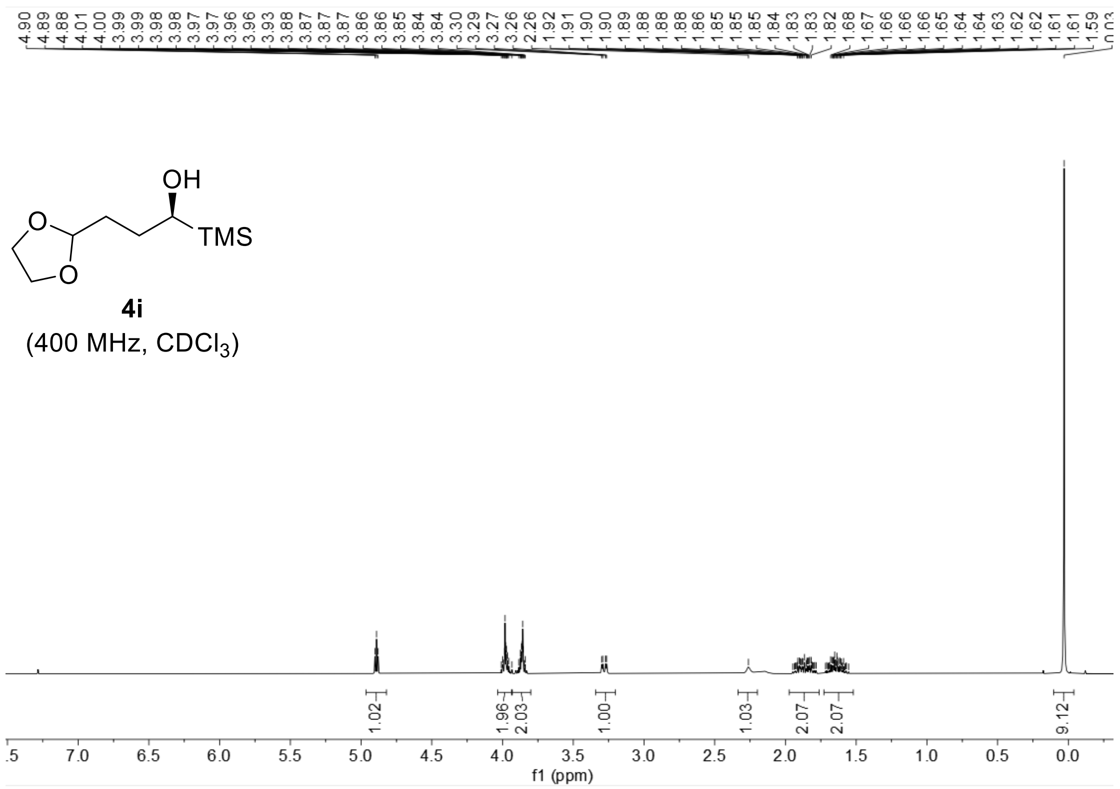
4.90 4.89 4.88 4.01 4.00 3.99 3.99 3.98 3.98 3.97 3.97 3.96 3.93 3.88 3.87 3.87 3.87 3.86 3.86 3.85 3.84 3.30 3.29 3.27 3.26 2.26 1.92 1.91 1.90 1.89 1.88 1.88 1.88 1.86 1.85 1.85 1.85 1.84 1.83 1.83 1.82 1.68 1.67 1.66 1.66 1.66 1.65 1.64 1.64 1.63 1.62 1.62 1.61 1.61 1.59
OH
TMS
O
O
4i
(400 MHz, CDCl3)
1.02
1.96
2.03
1.00
1.03
2.07
2.07
9.12
f1 (ppm)

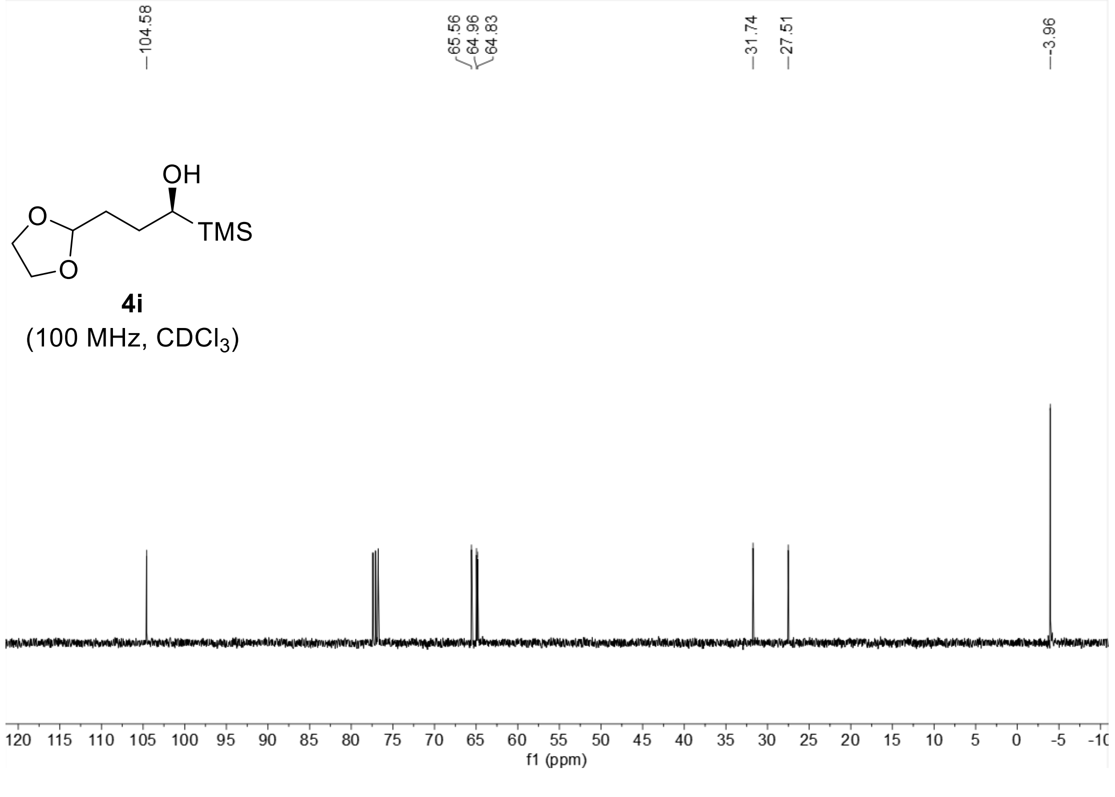
104.58
65.56
64.96
64.83
31.74
27.51
-3.96
OH
TMS
O
O
4i
(100 MHz, CDCl3)
f1 (ppm)

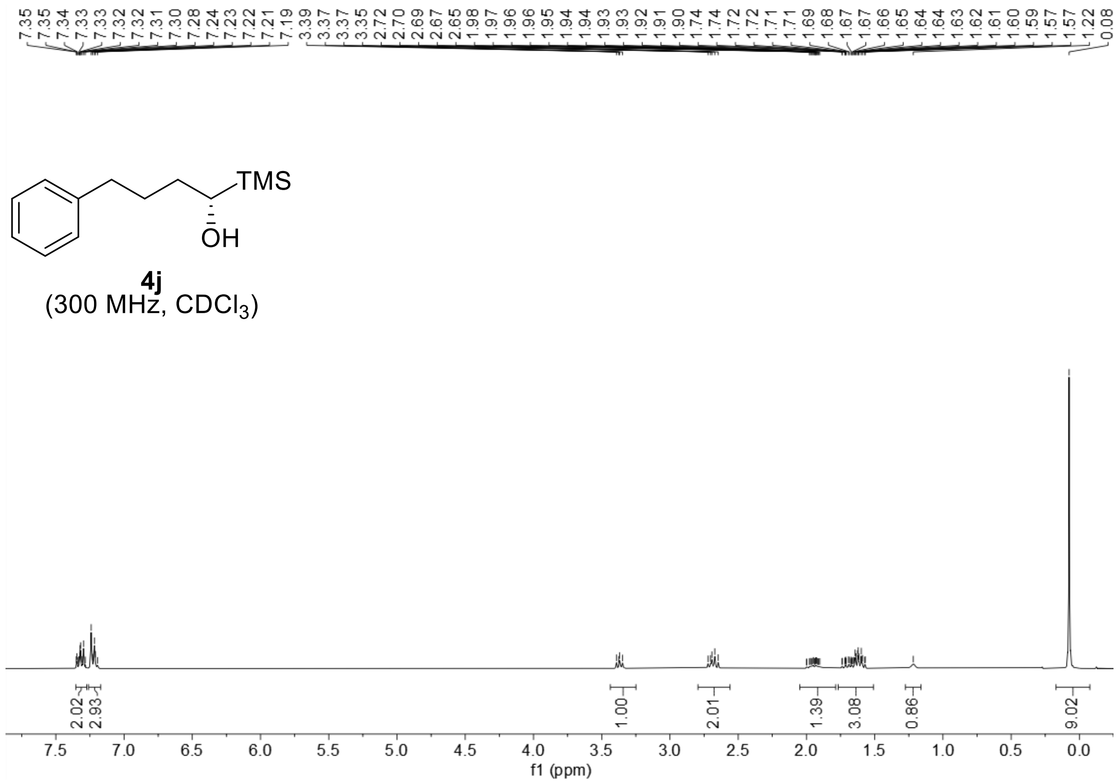
TMS
OH
4j
(300 MHz, CDCl3)
f1 (ppm)

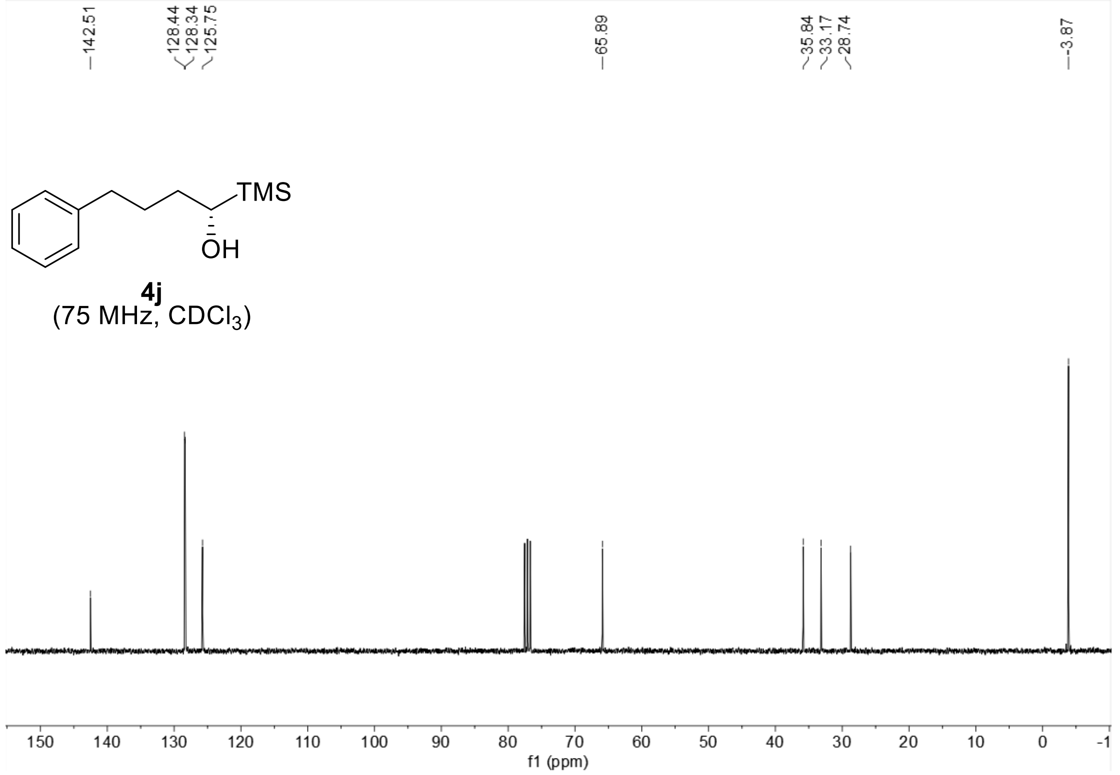
142.51
128.44
128.34
125.75
65.89
35.84
33.17
28.74
-3.87
TMS
OH
4j
(75 MHz, CDCl3)
f1 (ppm)

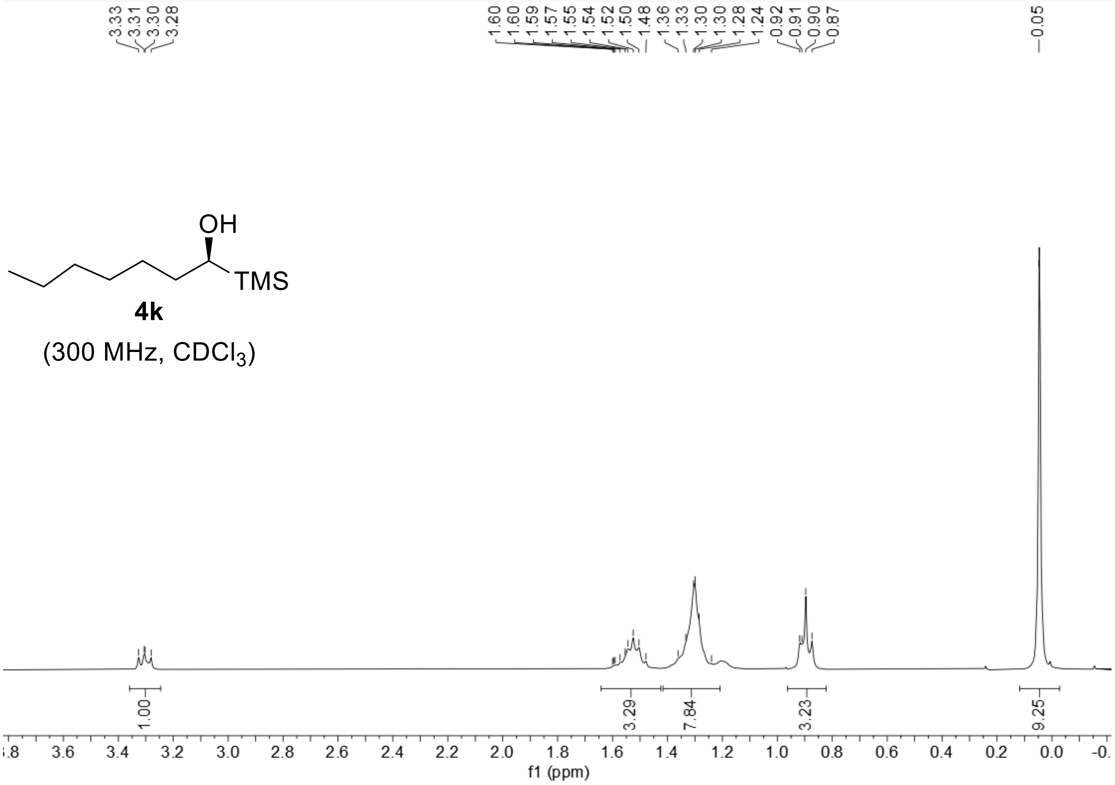
3.33
3.31
3.30
3.28
1.60
1.60
1.59
1.57
1.55
1.54
1.52
1.50
1.48
1.36
1.33
1.30
1.30
1.28
1.24
0.92
0.91
0.90
0.87
0.05
OH
TMS
4k
(300 MHz, CDCl$_3$)
1.00
3.29
7.84
3.23
9.25
f1 (ppm)

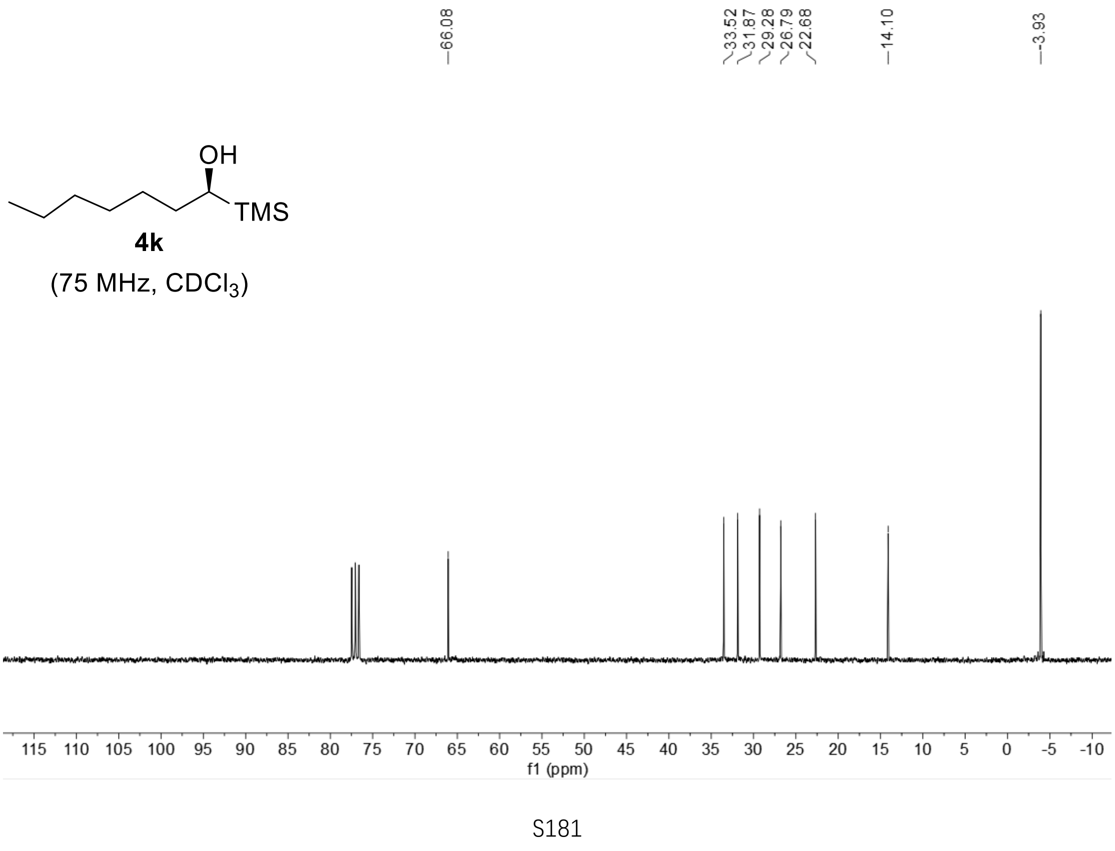
66.08
33.52
31.87
29.28
26.79
22.68
14.10
-3.93
OH
TMS
4k
(75 MHz, CDCl$_3$)
f1 (ppm)

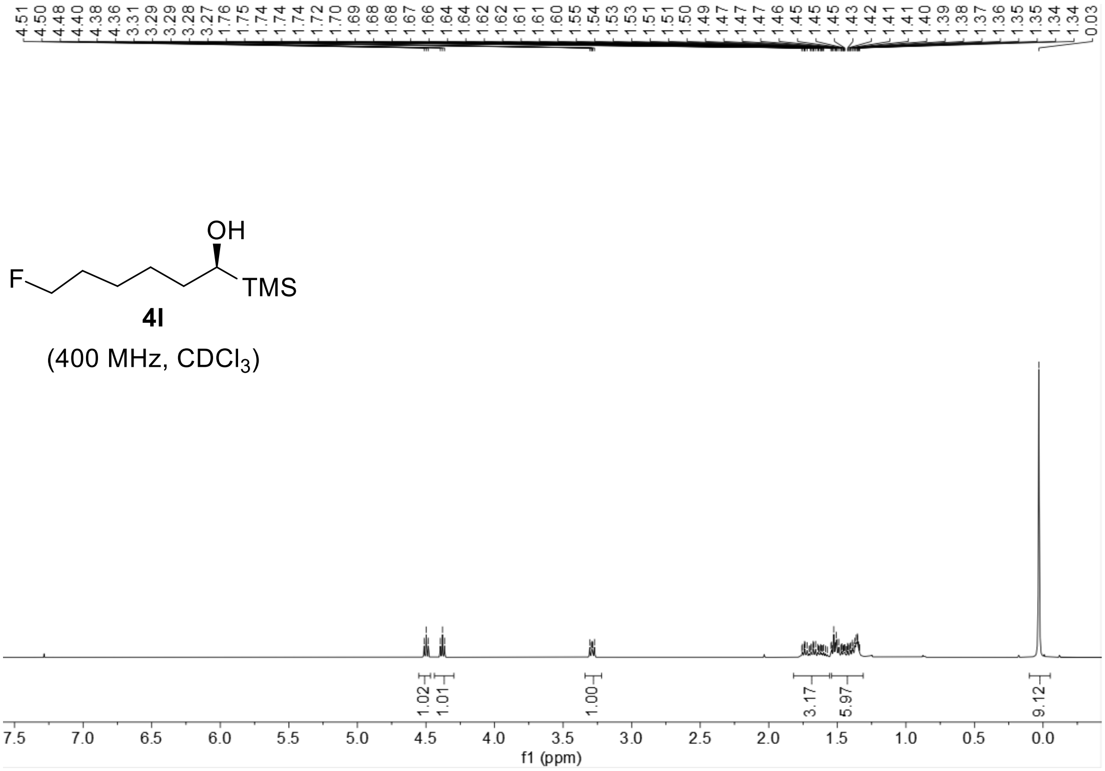
4.51 4.50 4.48 4.40 4.38 4.36 3.31 3.29 3.29 3.28 3.27 1.76 1.75 1.74 1.74 1.74 1.72 1.70 1.69 1.68 1.67 1.66 1.64 1.64 1.62 1.62 1.61 1.61 1.60 1.55 1.54 1.53 1.53 1.51 1.51 1.50 1.49 1.47 1.47 1.47 1.46 1.45 1.45 1.45 1.43 1.42 1.41 1.41 1.40 1.39 1.38 1.37 1.36 1.35 1.34 1.34 0.03
OH
F
TMS
4l
(400 MHz, CDCl3)
1.02
1.01
1.00
3.17
5.97
9.12
f1 (ppm)

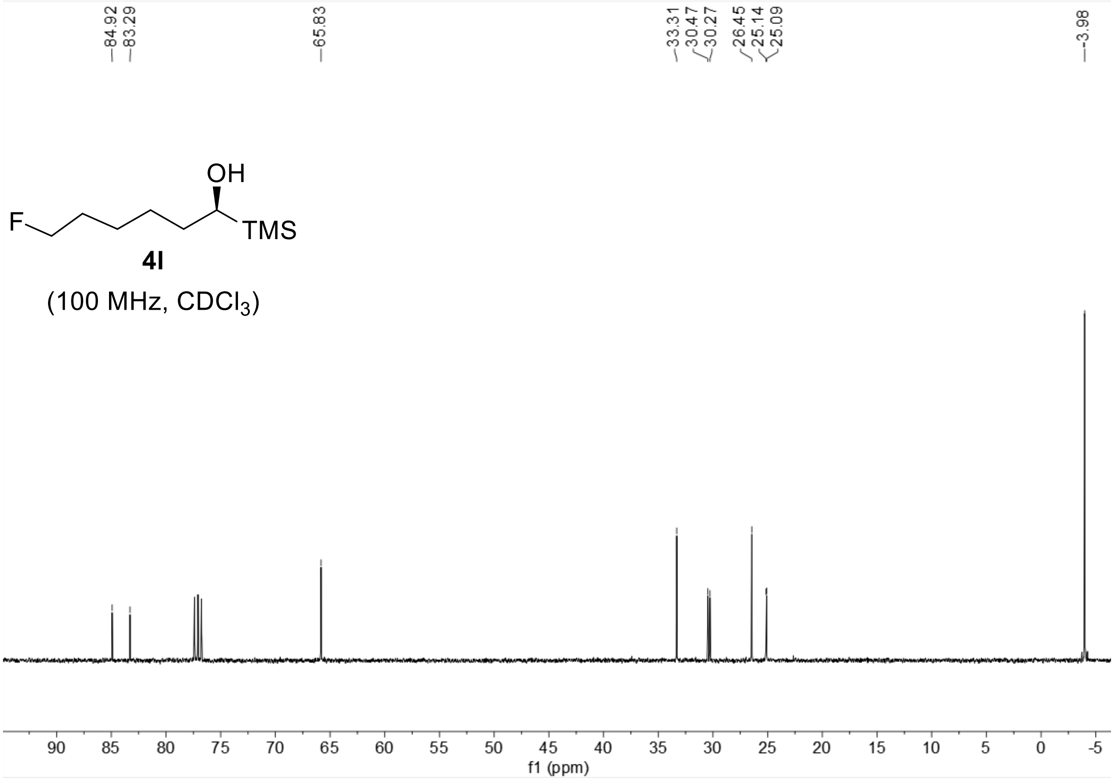
84.92
83.29
65.83
33.31
30.47
30.27
26.45
25.14
25.09
-3.98
OH
F
TMS
4l
(100 MHz, CDCl3)
f1 (ppm)

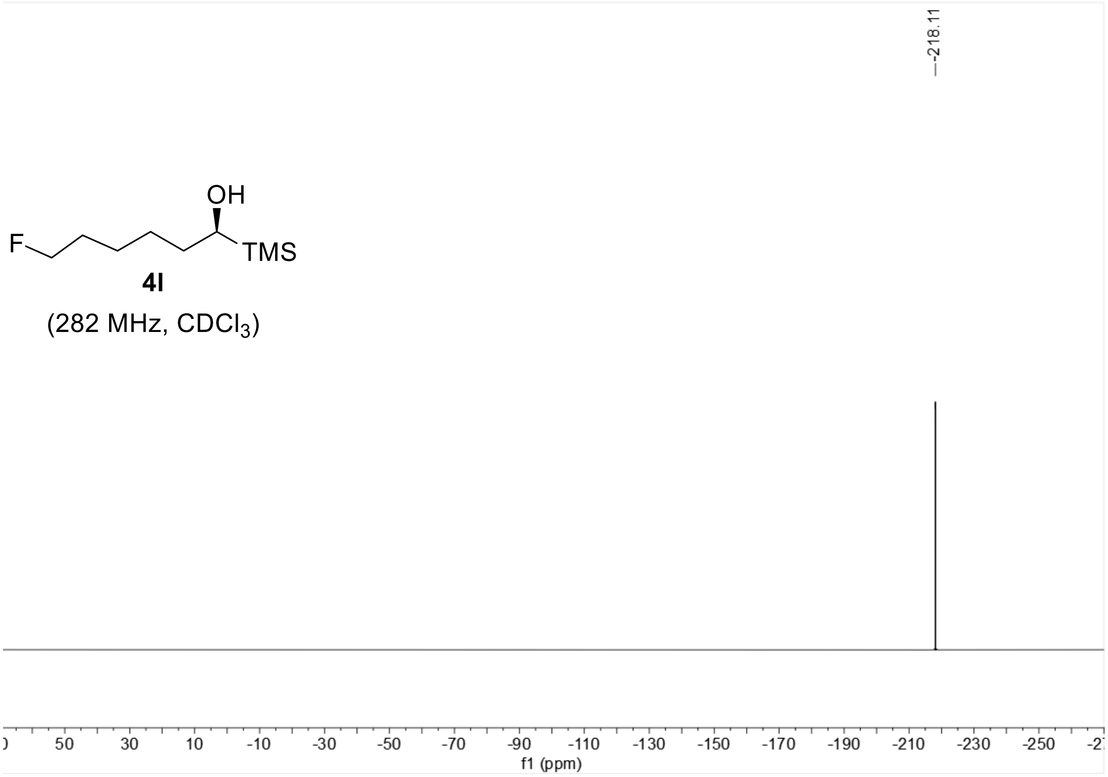

—218.11
OH
F
TMS
4l
(282 MHz, CDCl3)
50
30
10
-10
-30
-50
-70
-90
-110
-130
-150
-170
-190
-210
-230
-250
f1 (ppm)

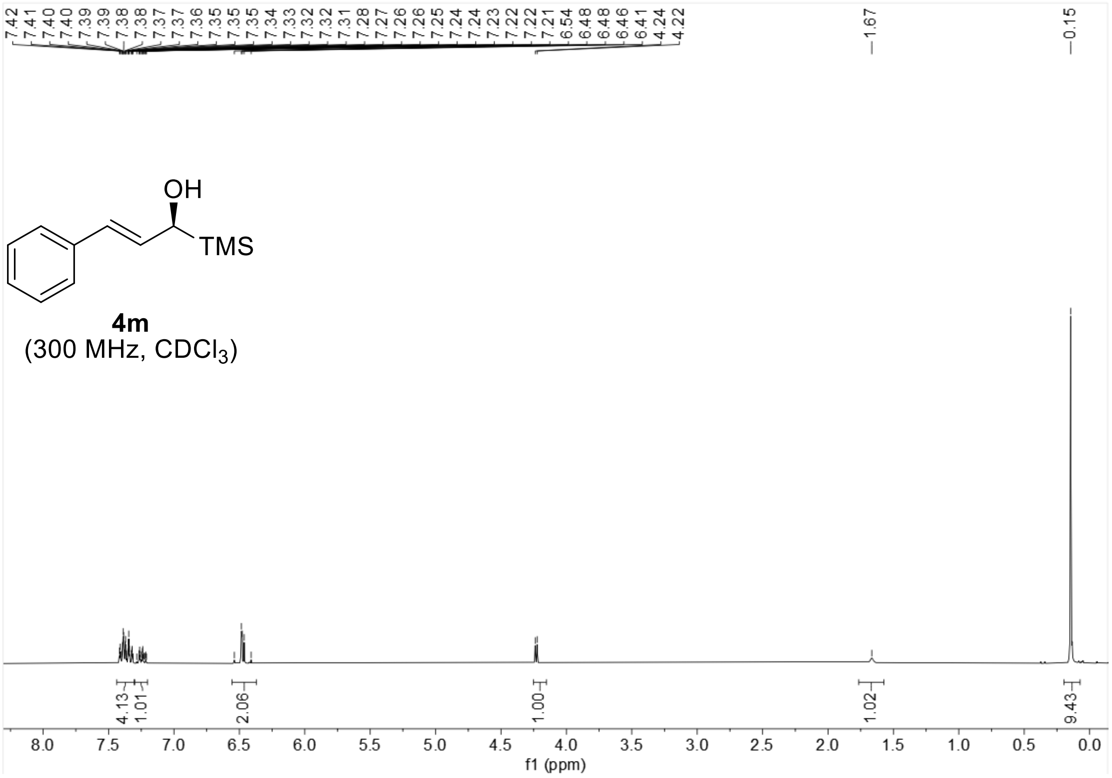
7.42
7.41
7.40
7.40
7.39
7.38
7.38
7.37
7.37
7.36
7.35
7.35
7.35
7.34
7.33
7.32
7.32
7.31
7.28
7.27
7.26
7.26
7.25
7.24
7.24
7.23
7.22
7.22
7.21
6.54
6.48
6.48
6.46
6.41
4.24
4.22
1.67
0.15
OH
TMS
4m
(300 MHz, CDCl3)
4.13
1.01
2.06
1.00
1.02
9.43
f1 (ppm)

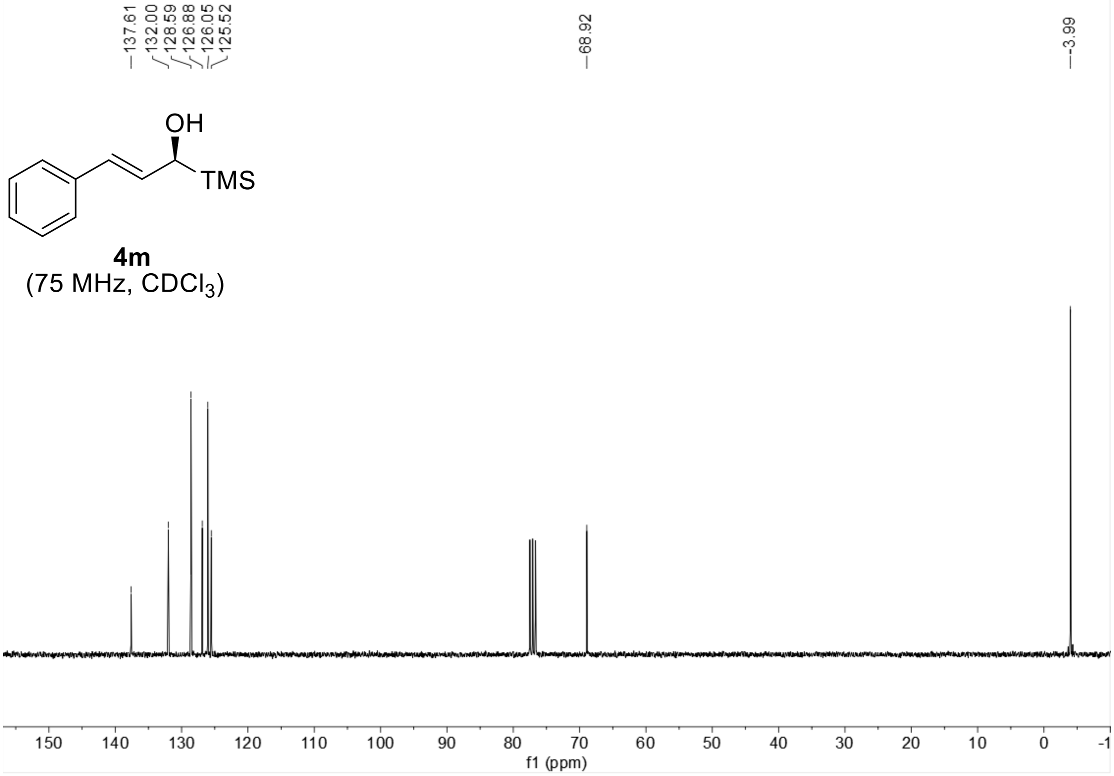
137.61
132.00
128.59
126.88
126.05
125.52
68.92
-3.99
OH
TMS
4m
(75 MHz, CDCl3)
f1 (ppm)

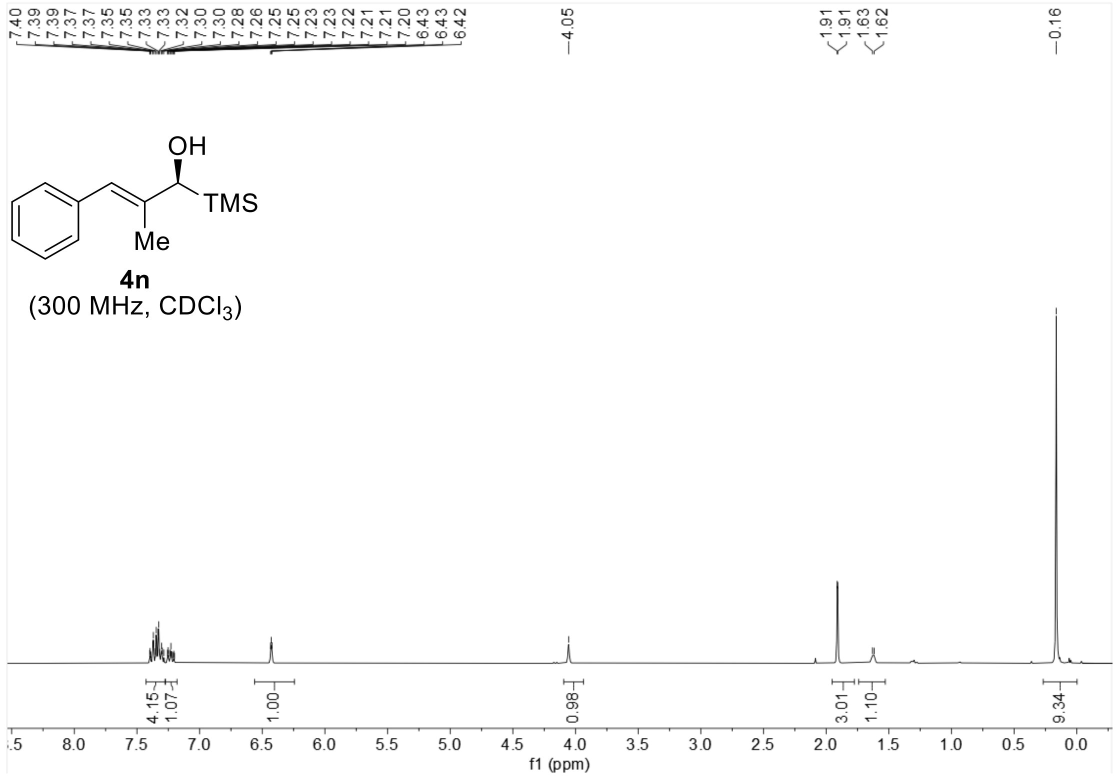
7.40
7.39
7.39
7.37
7.37
7.35
7.35
7.33
7.33
7.32
7.30
7.30
7.28
7.26
7.25
7.25
7.23
7.23
7.22
7.21
7.21
7.20
6.43
6.43
6.42
4.05
1.91
1.91
1.63
1.62
0.16
OH
TMS
Me
4n
(300 MHz, CDCl3)
4.15
1.07
1.00
0.98
3.01
1.10
9.34
f1 (ppm)

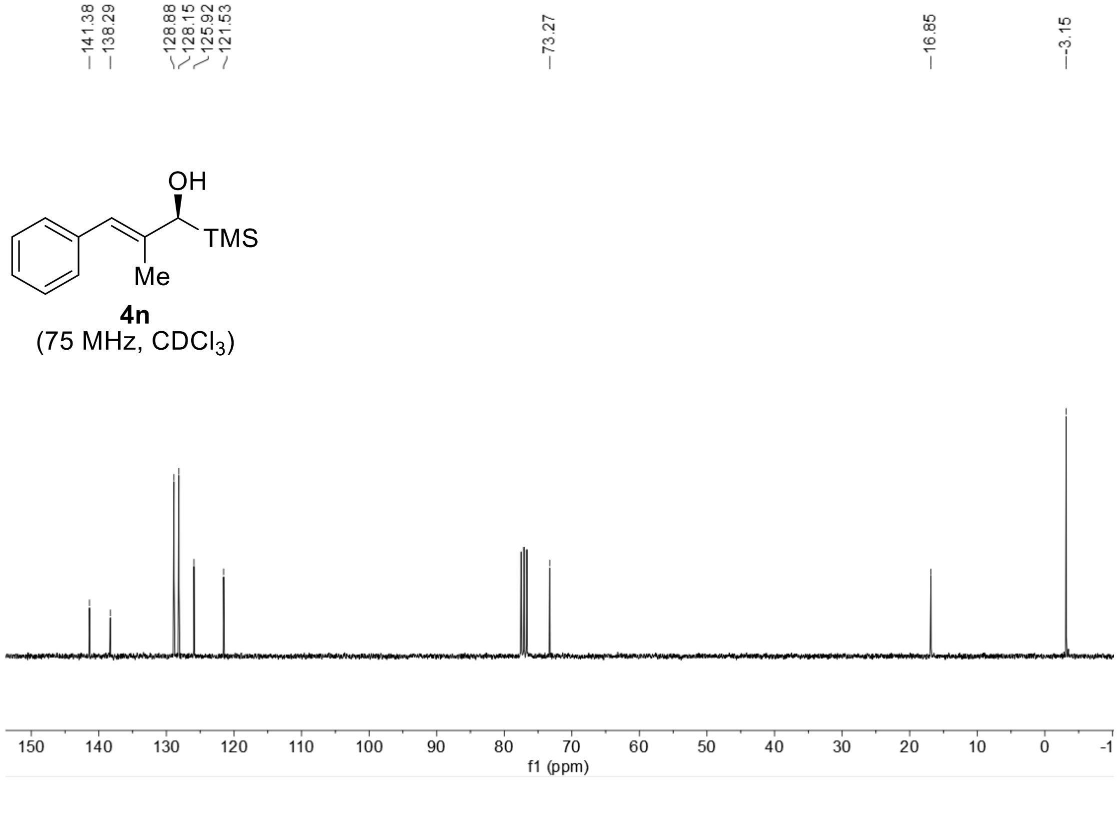
141.38
138.29
128.88
128.15
125.92
121.53
73.27
16.85
-3.15
OH
TMS
Me
4n
(75 MHz, CDCl3)
f1 (ppm)

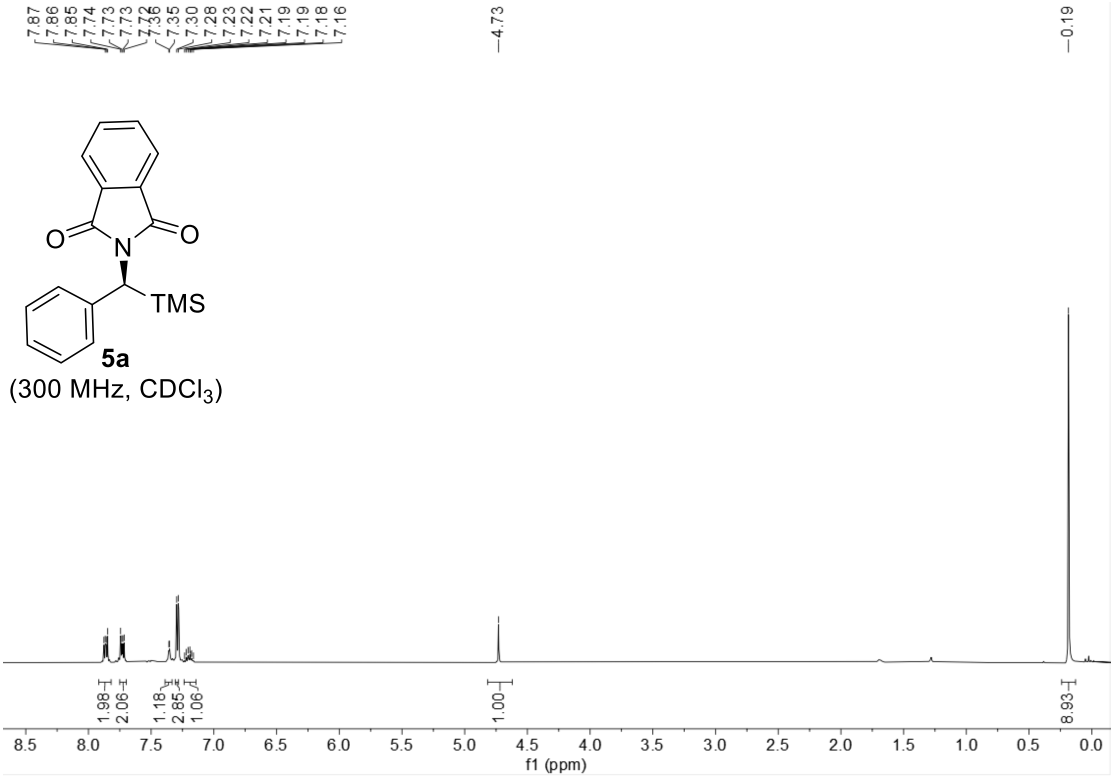
7.87
7.86
7.85
7.74
7.73
7.73
7.72
7.36
7.35
7.30
7.28
7.23
7.22
7.21
7.19
7.18
7.16
4.73
0.19
O
N
O
TMS
5a
(300 MHz, CDCl3)
1.98
2.06
1.18
2.85
1.06
1.00
8.93
f1 (ppm)

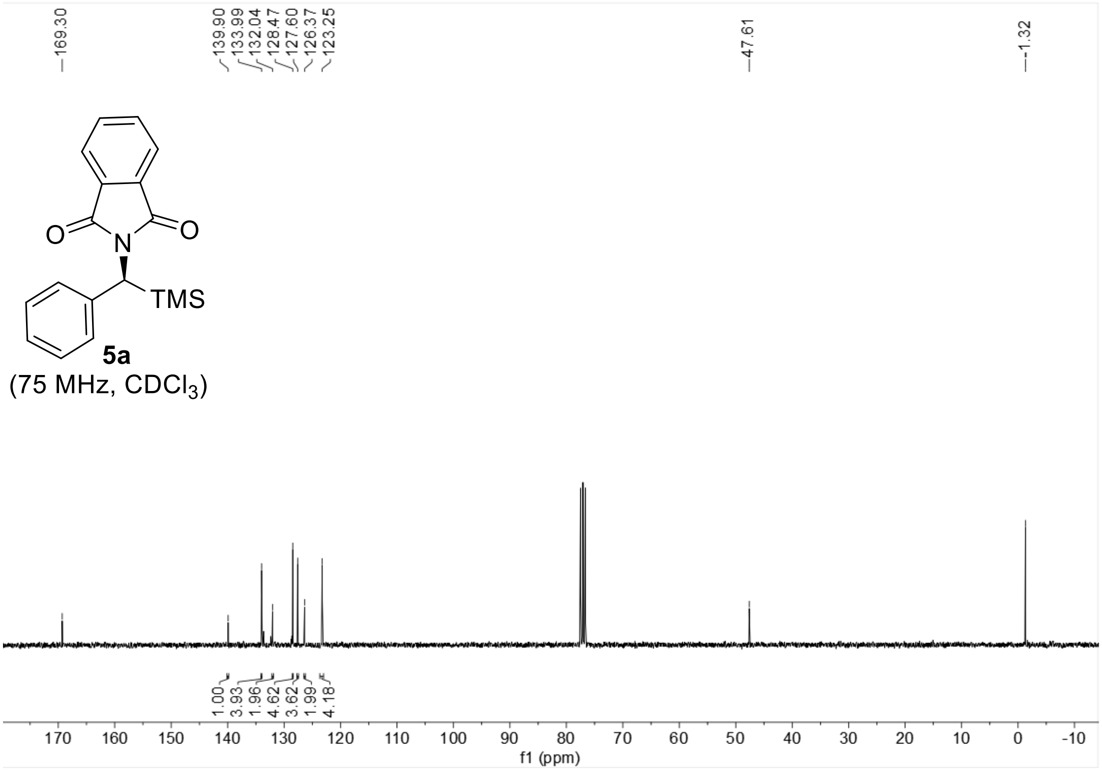
169.30
139.90
133.99
132.04
128.47
127.60
126.37
123.25
47.61
-1.32
O
N
O
TMS
5a
(75 MHz, CDCl3)
1.00
3.93
1.96
4.62
3.62
1.99
4.18
f1 (ppm)

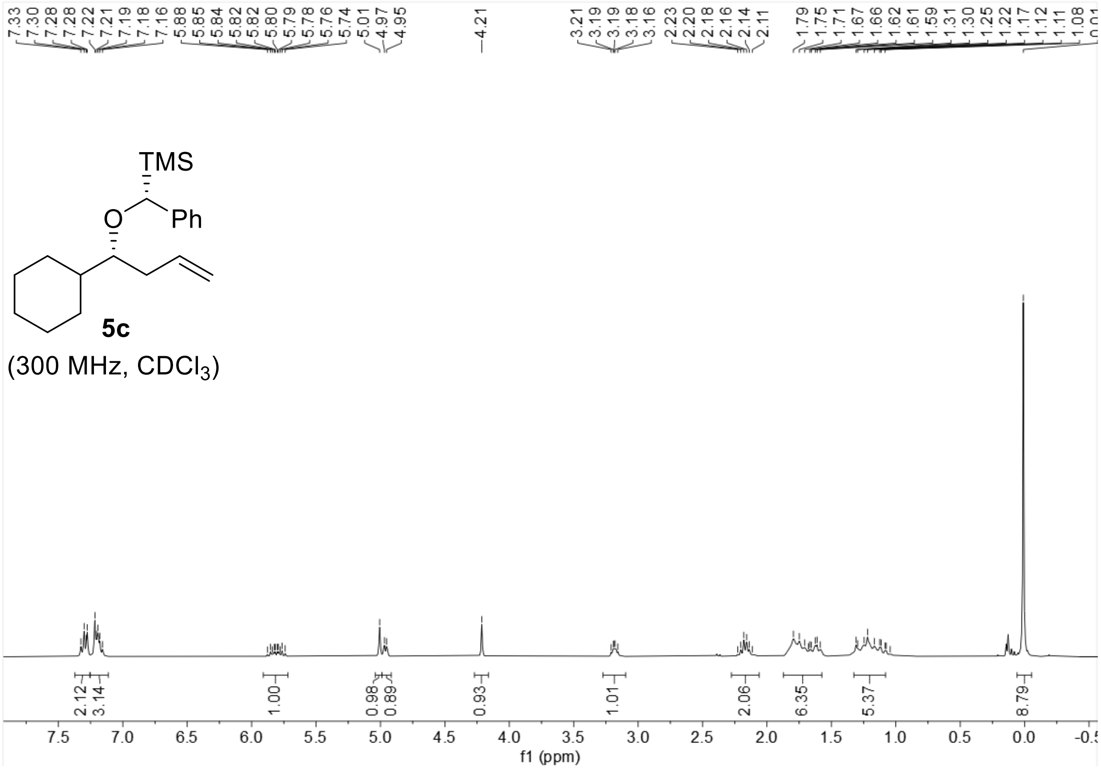
7.33 7.30 7.28 7.28 7.22 7.21 7.19 7.18 7.16 5.88 5.85 5.84 5.82 5.82 5.80 5.79 5.78 5.76 5.74 5.01 4.97 4.95
4.21
3.21 3.19 3.19 3.18 3.16 2.23 2.20 2.18 2.16 2.14 2.11
1.79 1.75 1.71 1.67 1.66 1.62 1.61 1.59 1.31 1.30 1.25 1.22 1.17 1.12 1.11 1.08
TMS
O
Ph
5c
(300 MHz, CDCl3)
2.12 3.14 1.00 0.98 0.89 0.93 1.01 2.06 6.35 5.37 8.79
f1 (ppm)

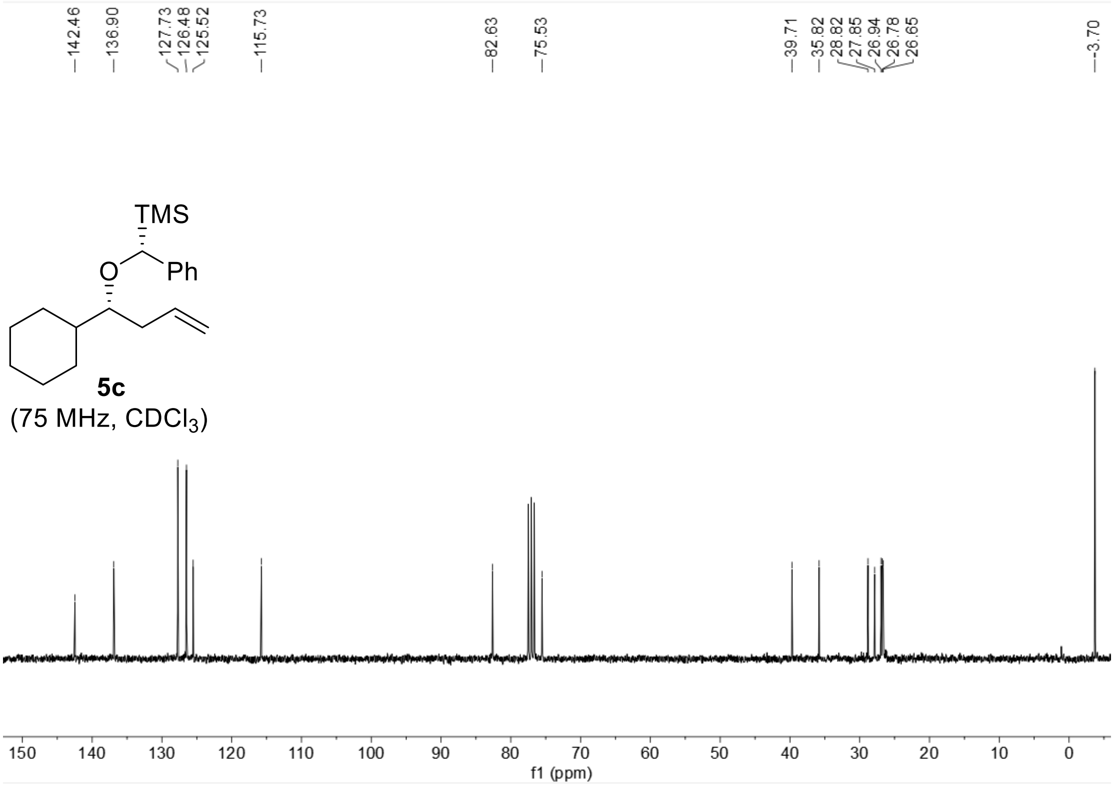
142.46
136.90
127.73 126.48 125.52
115.73
82.63
75.53
39.71
35.82 28.82 27.85 26.94 26.78 26.65
-3.70
TMS
O
Ph
5c
(75 MHz, CDCl3)
f1 (ppm)

XIII. HPLC Traces

(*S*)-Phenyl(trimethylsilyl)methanol (2a)

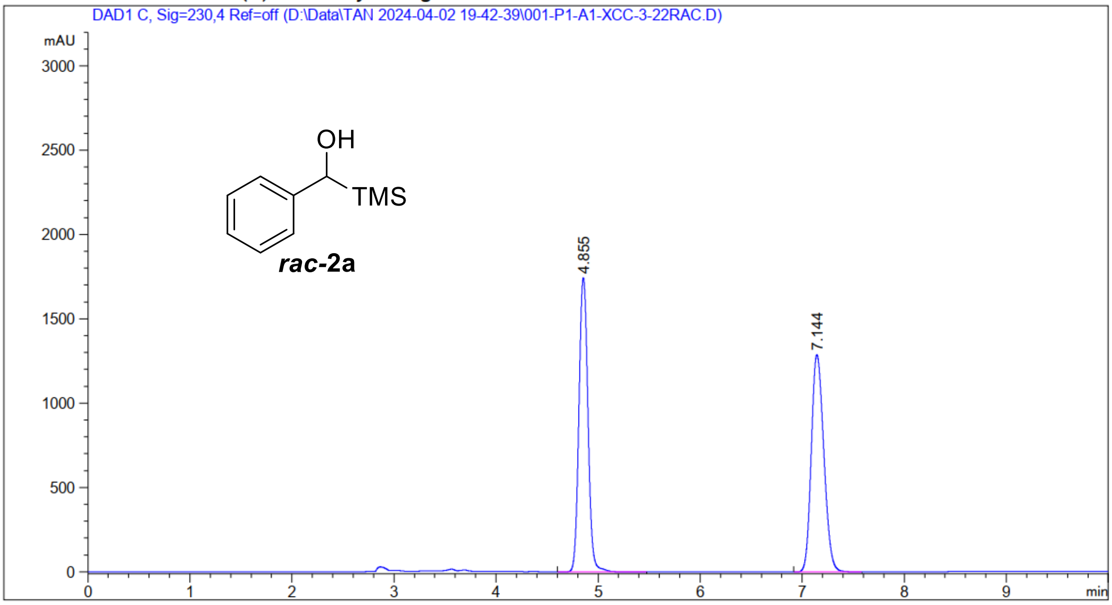

Peak #	RetTime [min]	Type	Width [min]	Area [mAU*s]	Height [mAU]	Area %
1	4.855	BB	0.0967	1.07248e4	1744.13904	49.3407
2	7.144	BB	0.1341	1.10114e4	1289.18213	50.6593

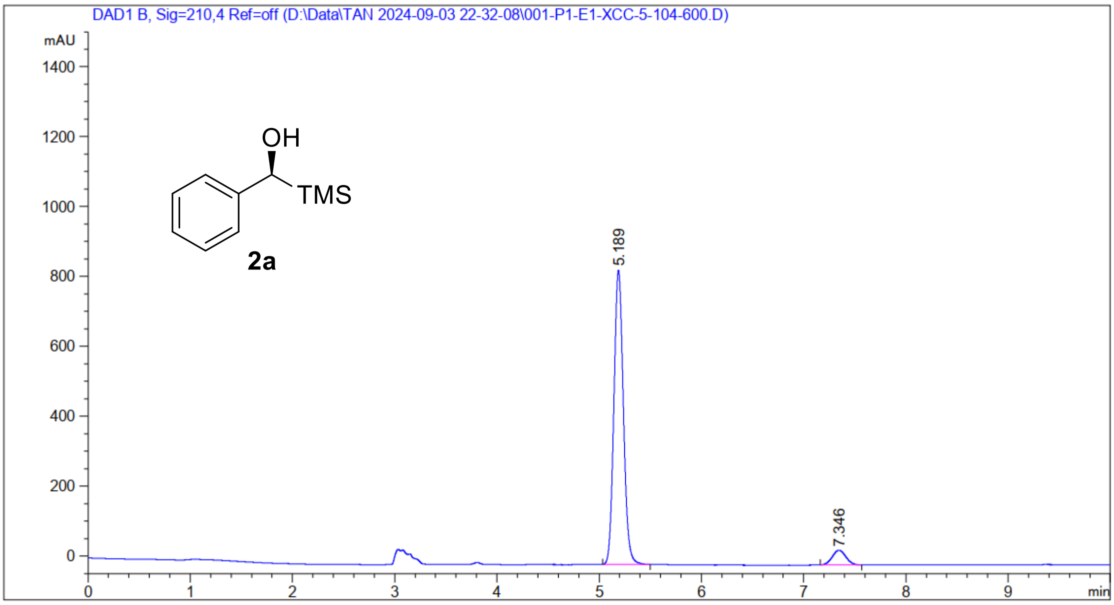

Peak #	RetTime [min]	Type	Width [min]	Area [mAU*s]	Height [mAU]	Area %
1	5.189	BB	0.0976	5409.49219	842.06946	93.3154
2	7.346	BB	0.1085	387.50891	42.05543	6.6846

(*S*)-Phenyl(triethylsilyl)methanol (2b)

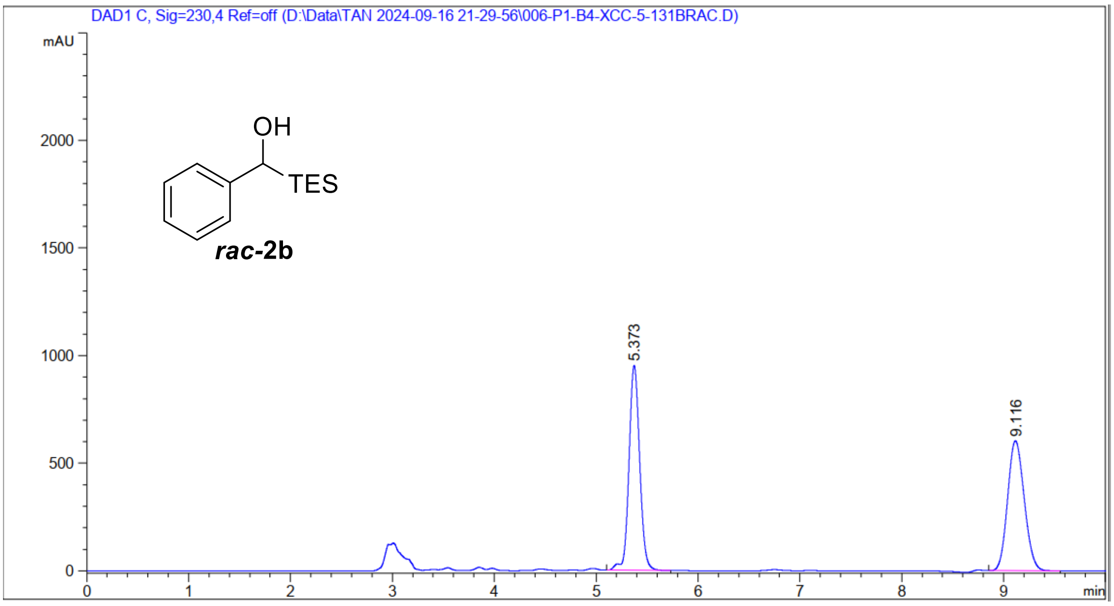

Peak #	RetTime [min]	Type	Width [min]	Area [mAU*s]	Height [mAU]	Area %
1	5.373	BB	0.1132	6943.78906	951.04779	50.3586
2	9.116	BB	0.1747	6844.88965	603.57013	49.6414

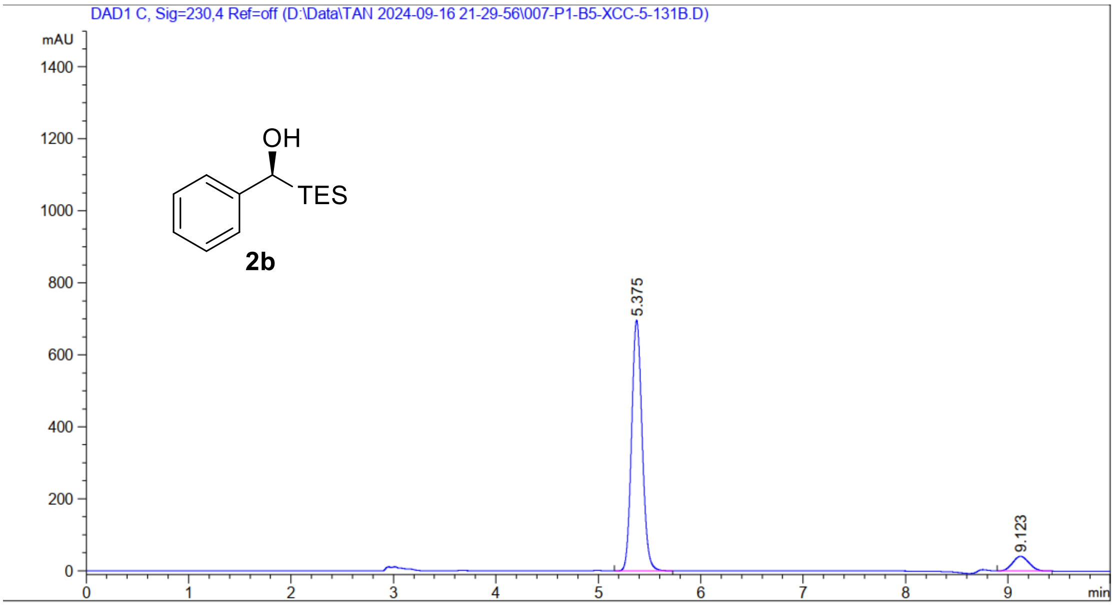

Peak #	RetTime [min]	Type	Width [min]	Area [mAU*s]	Height [mAU]	Area %
1	5.375	BB	0.1107	4976.09717	696.48895	91.6785
2	9.123	BB	0.1334	451.66943	40.71584	8.3215

(*S*)-(*tert*-Butyldimethylsilyl)(phenyl)methanol (2c)

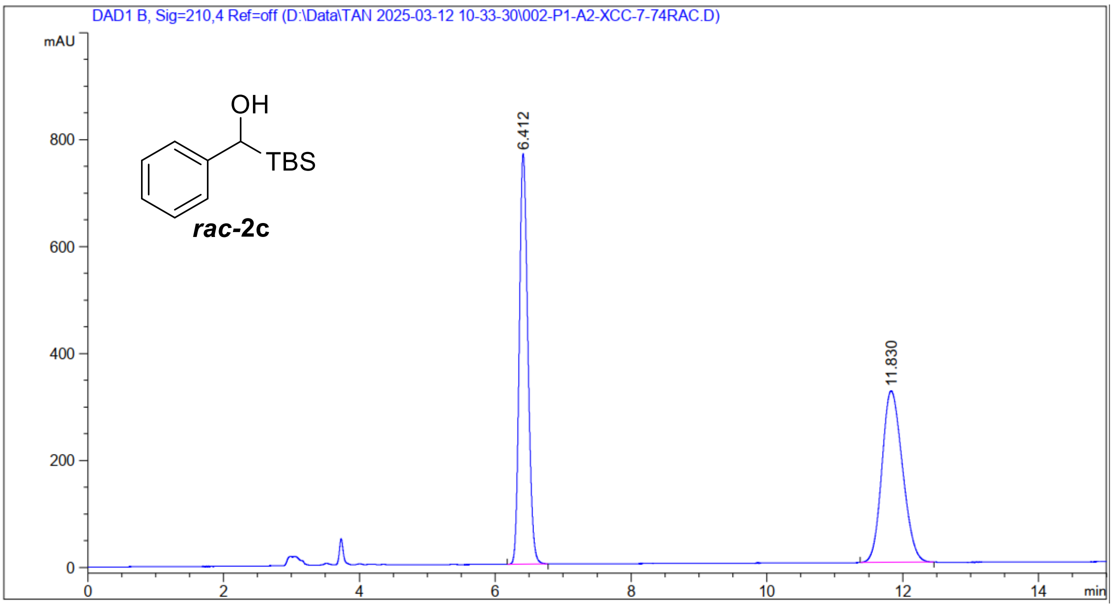

Peak #	RetTime [min]	Type	Width [min]	Area [mAU*s]	Height [mAU]	Area %
1	6.412	BB	0.1309	6720.98389	767.62018	49.8612
2	11.830	BB	0.2513	6758.41504	320.75977	50.1388

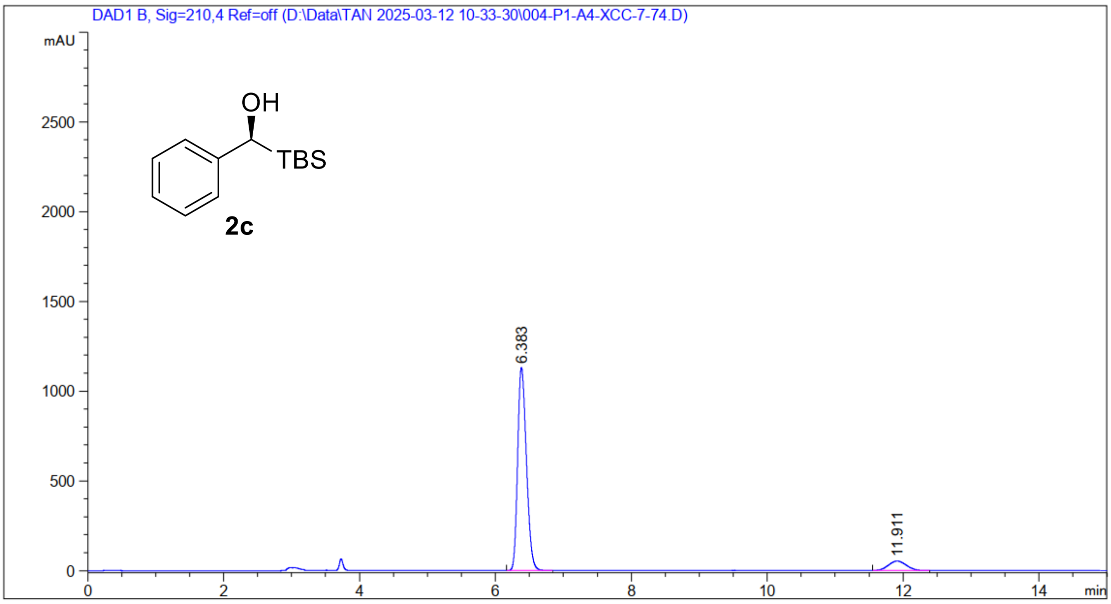

Peak #	RetTime [min]	Type	Width [min]	Area [mAU*s]	Height [mAU]	Area %
1	6.383	BB	0.1331	1.01412e4	1130.99390	91.2673
2	11.911	BB	0.2160	970.33386	52.78405	8.7327

(*S*)-*p*-Tolyl(trimethylsilyl)methanol (2d)

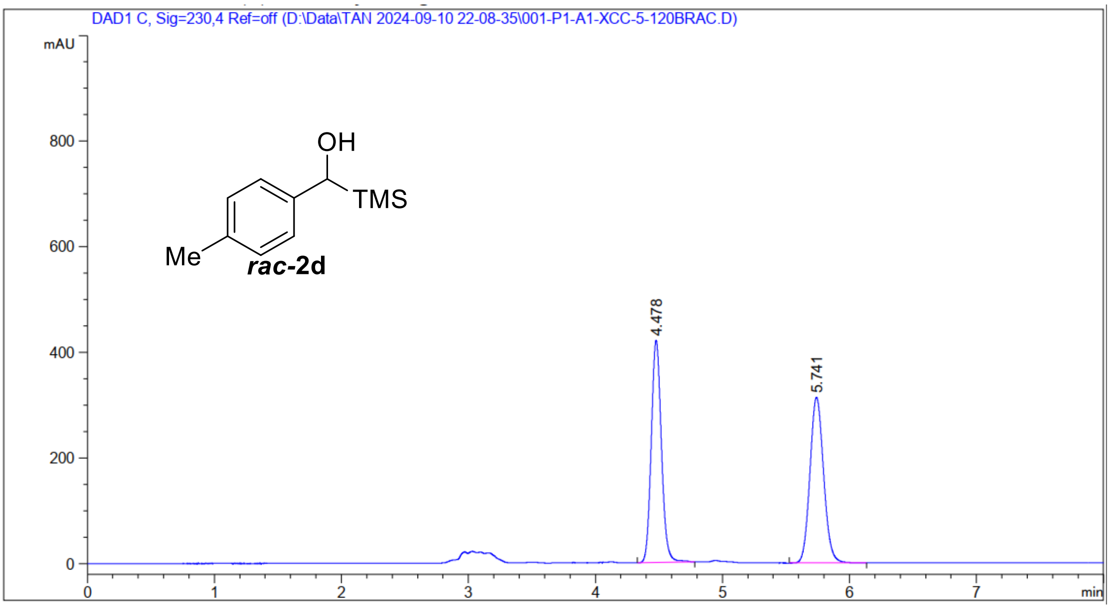

Peak #	RetTime [min]	Type	Width [min]	Area [mAU*s]	Height [mAU]	Area %
1	4.478	BB	0.0865	2344.88110	420.91937	49.9523
2	5.741	BB	0.1169	2349.35522	313.66473	50.0477

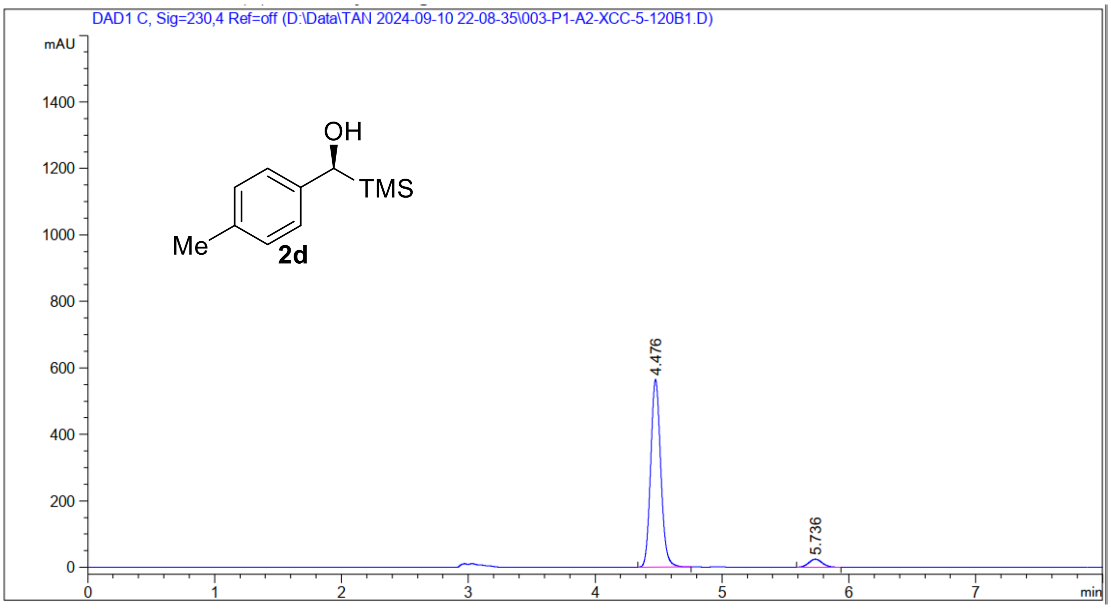

Peak #	RetTime [min]	Type	Width [min]	Area [mAU*s]	Height [mAU]	Area %
1	4.476	BB	0.0856	3132.60010	564.67401	94.6256
2	5.736	BB	0.0864	177.92160	24.22363	5.3744

(*S*)-(4-(*tert*-Butyl)phenyl)(trimethylsilyl)methanol (2e)

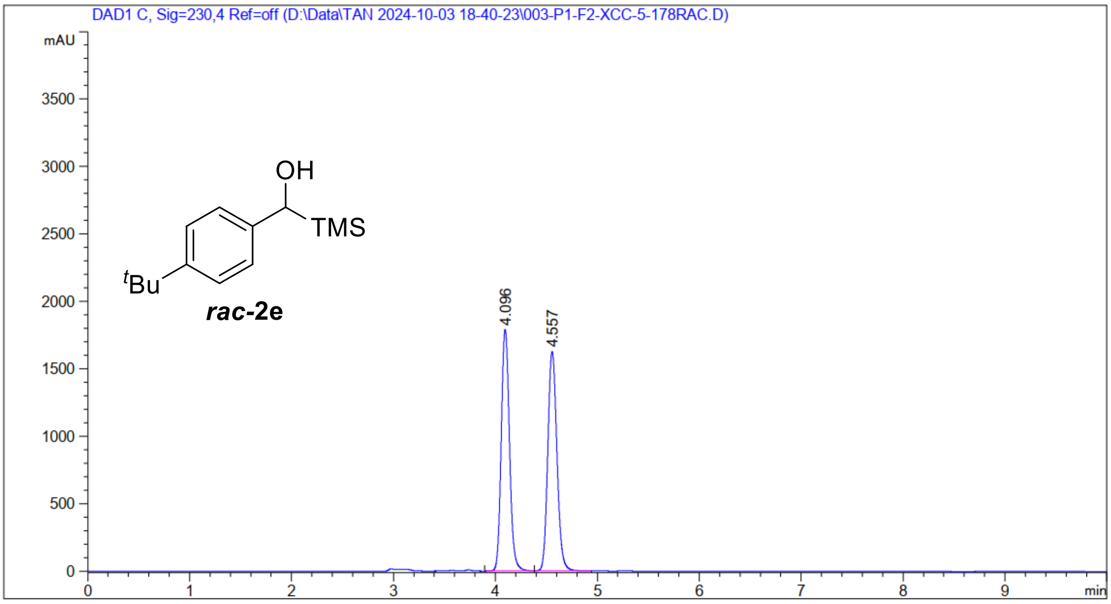

Peak #	RetTime [min]	Type	Width [min]	Area [mAU*s]	Height [mAU]	Area %
1	4.096	BB	0.0876	1.00380e4	1789.26685	49.7950
2	4.557	BB	0.0970	1.01206e4	1626.59863	50.2050

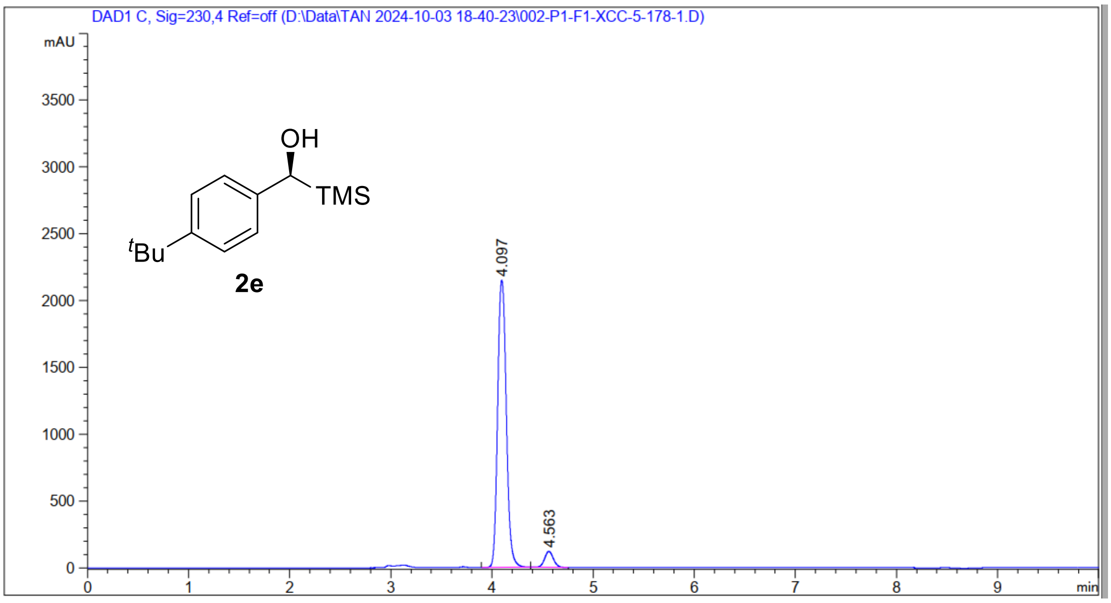

Peak #	RetTime [min]	Type	Width [min]	Area [mAU*s]	Height [mAU]	Area %
1	4.097	BB	0.0930	1.27842e4	2149.36914	94.6074
2	4.563	BB	0.0938	728.70459	119.93669	5.3926

(*S*)-*m*-Tolyl(trimethylsilyl)methanol (2f)

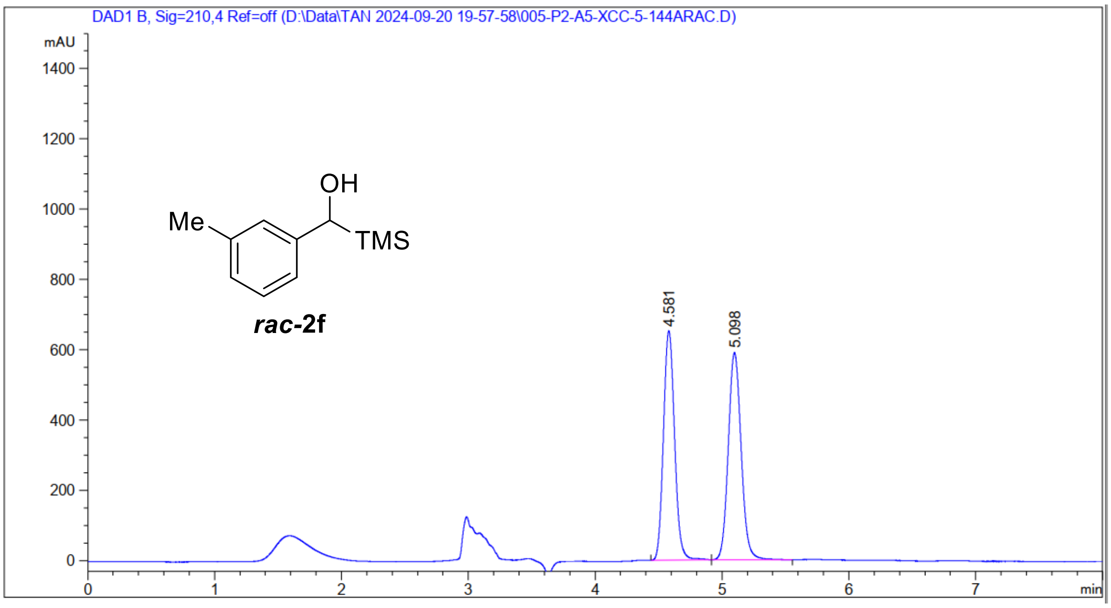

Peak #	RetTime [min]	Type	Width [min]	Area [mAU*s]	Height [mAU]	Area %
1	4.581	BB	0.0923	3987.31519	652.72144	48.9608
2	5.098	BV R	0.1088	4156.58203	589.30377	51.0392

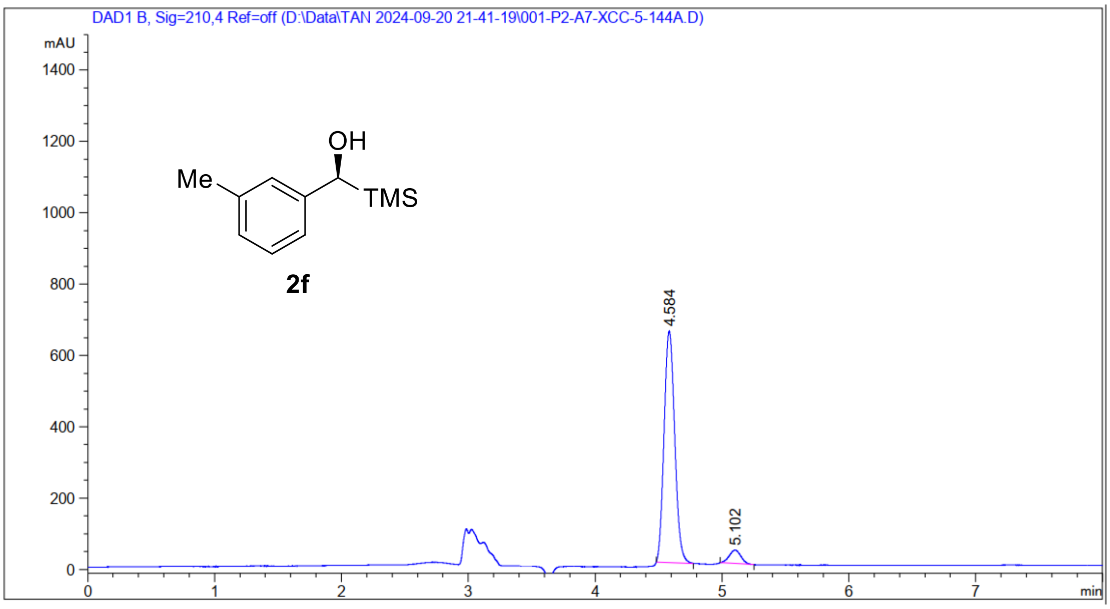

Peak #	RetTime [min]	Type	Width [min]	Area [mAU*s]	Height [mAU]	Area %
1	4.584	BB	0.0732	3772.05298	649.22278	93.9355
2	5.102	BB	0.0758	243.52527	37.66419	6.0645

(*S*)-*o*-Tolyl(trimethylsilyl)methanol (2g)

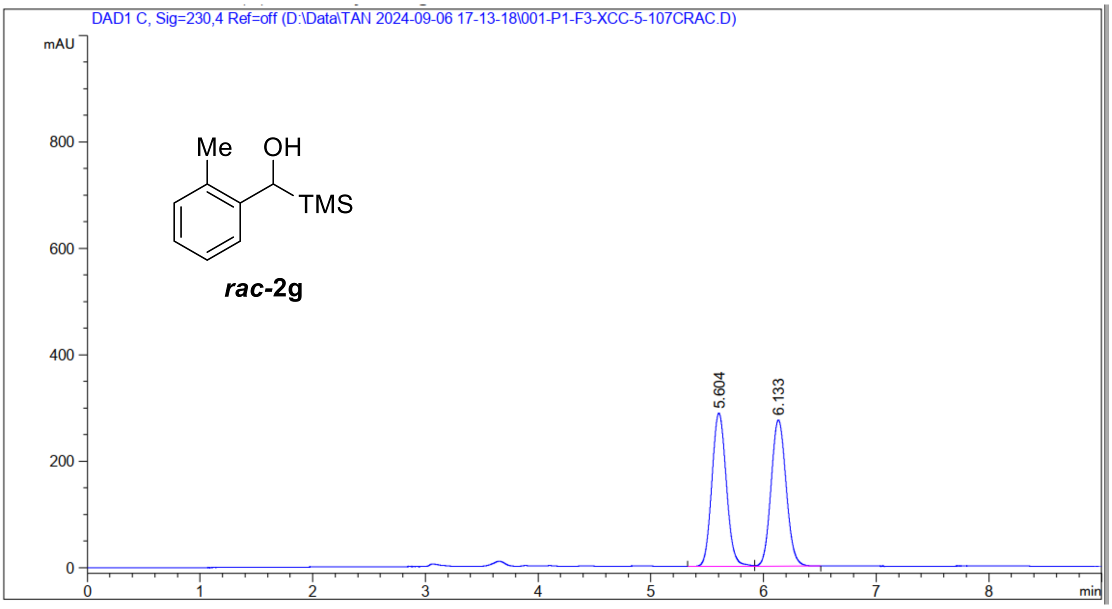

Peak #	RetTime [min]	Type	Width [min]	Area [mAU*s]	Height [mAU]	Area %
1	5.604	BV	0.1394	2575.10083	287.72900	50.1326
2	6.133	VB	0.1468	2561.48096	274.73346	49.8674

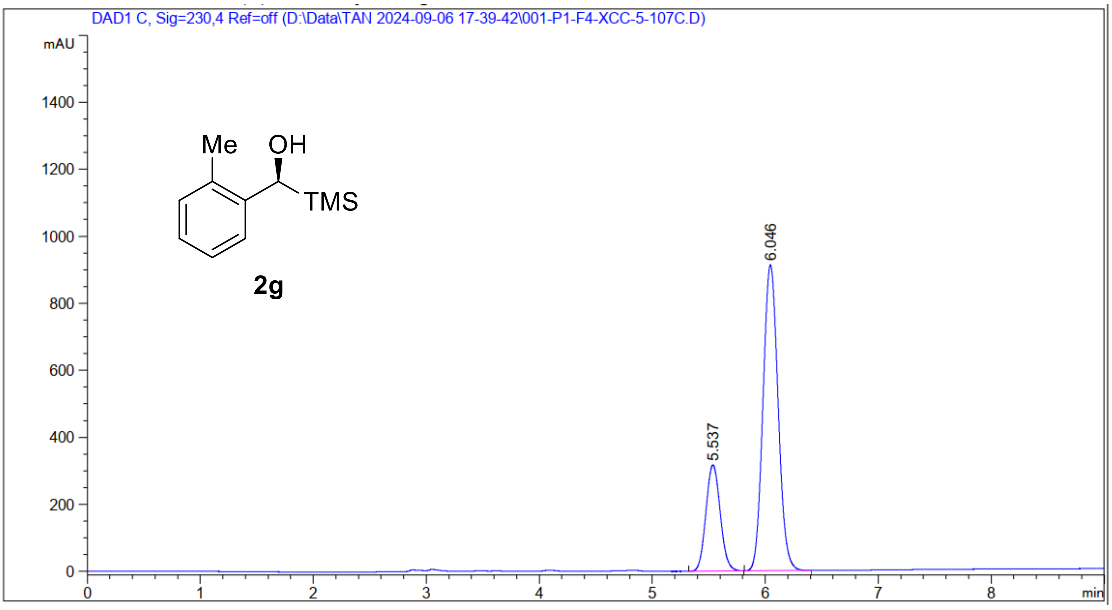

Peak #	RetTime [min]	Type	Width [min]	Area [mAU*s]	Height [mAU]	Area %
1	5.537	BB	0.1354	2789.59302	316.80930	24.7570
2	6.046	BB	0.1430	8478.29492	912.68536	75.2430

(*S*)-(4-Methoxyphenyl)(trimethylsilyl)methanol (2h)

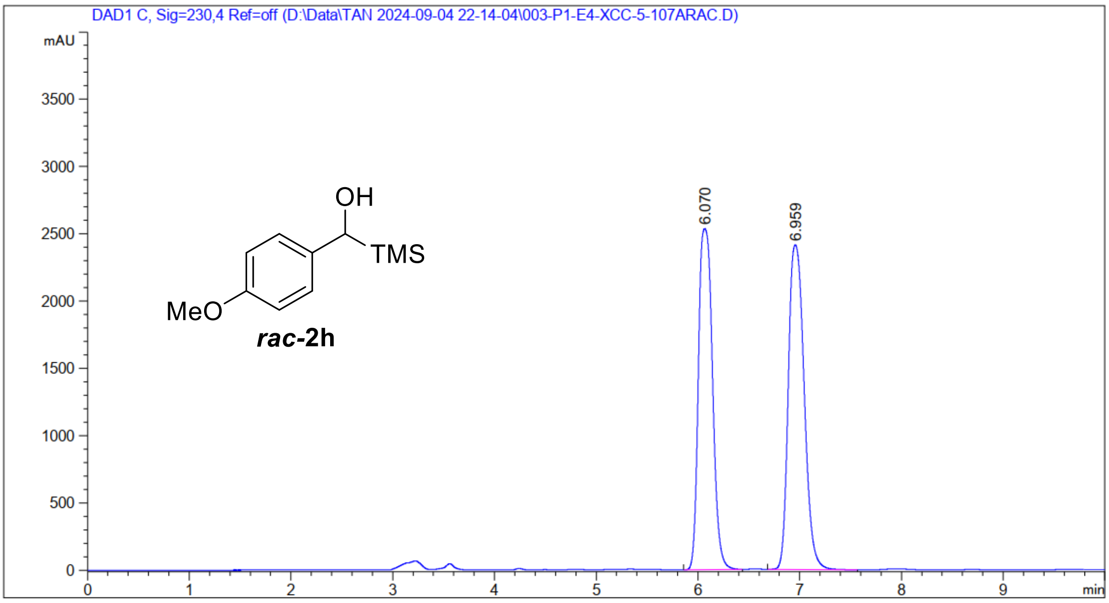

Peak #	RetTime [min]	Type	Width [min]	Area [mAU*s]	Height [mAU]	Area %
1	6.070	BB	0.1152	2.42925e4	2536.16602	48.2766
2	6.959	BB	0.1483	2.60269e4	2413.53735	51.7234

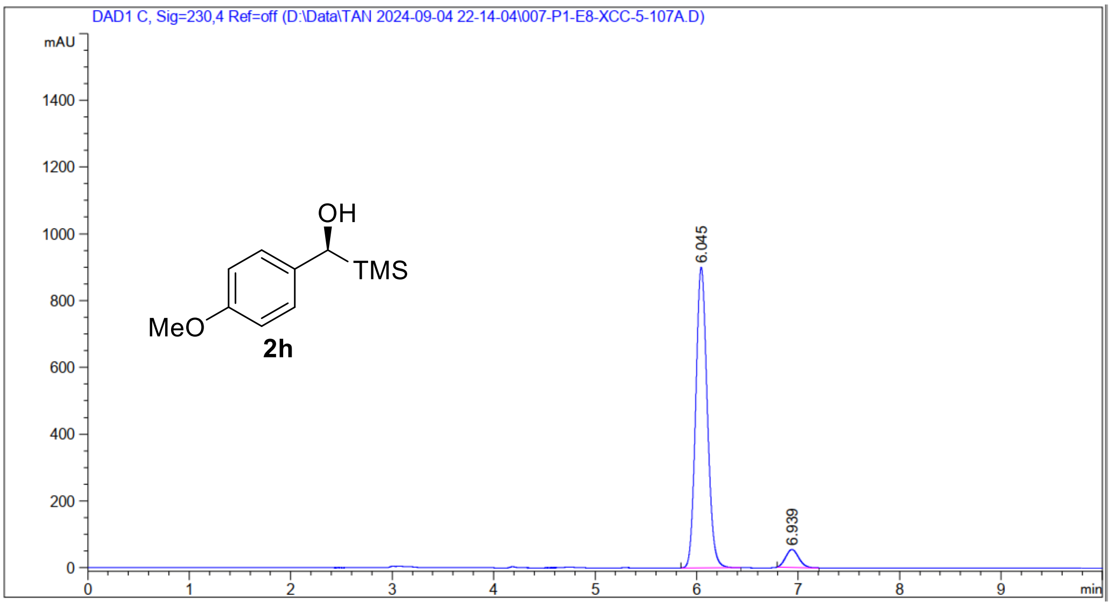

Peak #	RetTime [min]	Type	Width [min]	Area [mAU*s]	Height [mAU]	Area %
1	6.045	BB	0.1196	7104.01807	899.89355	93.7601
2	6.939	BB	0.1052	472.78326	53.05763	6.2399

(*S*)-(3-Methoxyphenyl)(trimethylsilyl)methanol (2i)

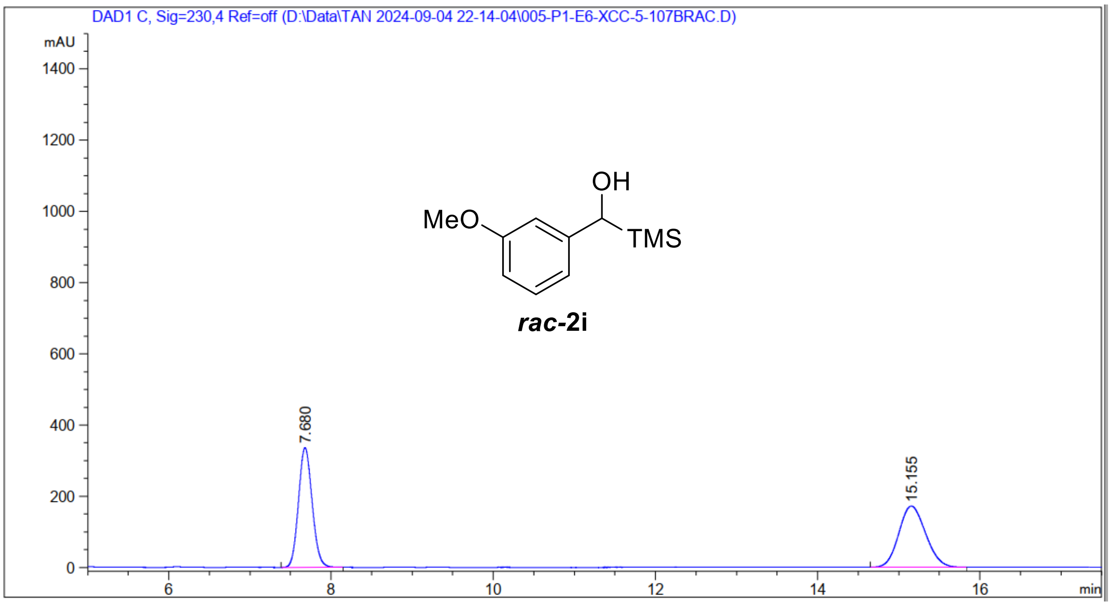

Peak #	RetTime [min]	Type	Width [min]	Area [mAU*s]	Height [mAU]	Area %
1	7.680	BB	0.1803	3935.83472	336.21399	49.9855
2	15.155	BB	0.2688	3938.12183	171.73387	50.0145

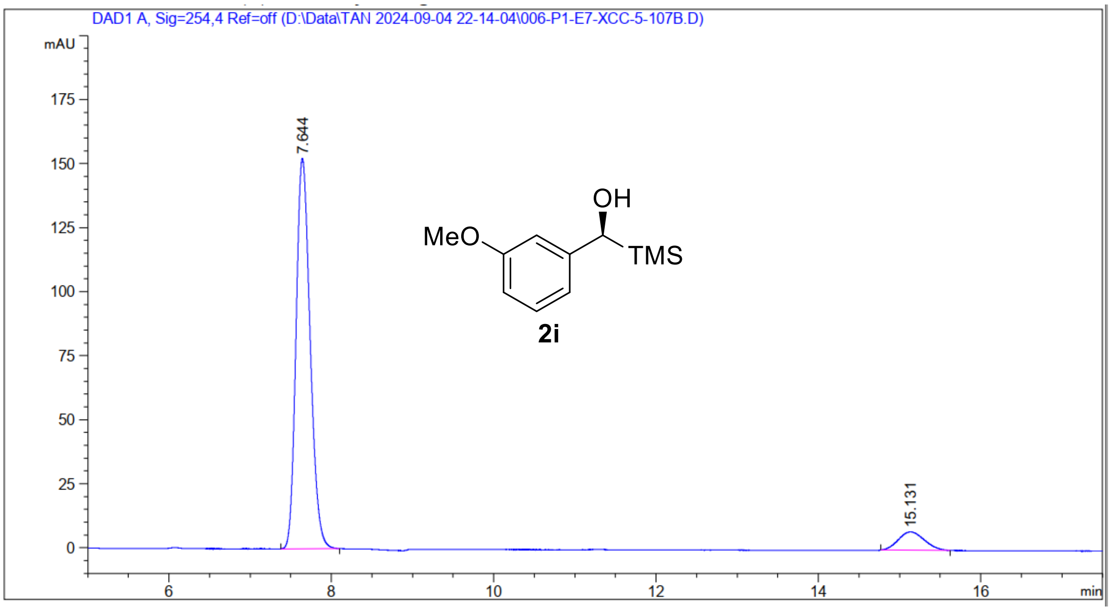

Peak #	RetTime [min]	Type	Width [min]	Area [mAU*s]	Height [mAU]	Area %
1	7.644	BB	0.1813	1833.07947	152.42348	92.0402
2	15.131	BB	0.2600	158.52855	7.13305	7.9598

(*S*)-[1,1'-Biphenyl]-4-yl(trimethylsilyl)methanol (2j)

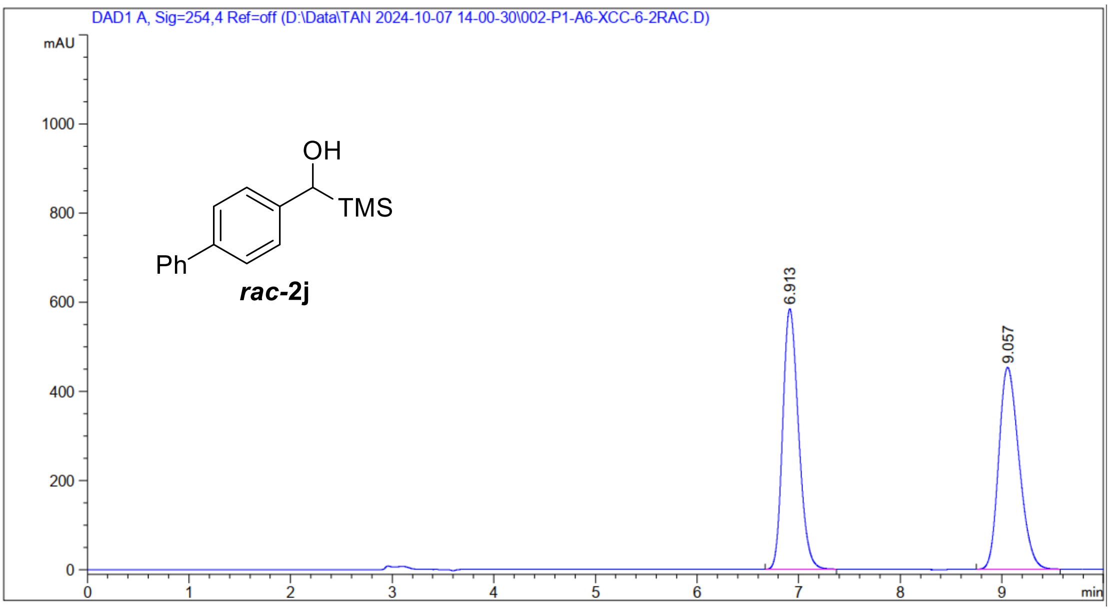

Peak #	RetTime [min]	Type	Width [min]	Area [mAU*s]	Height [mAU]	Area %
1	6.913	BB	0.1627	6300.06396	583.93652	50.0112
2	9.057	BB	0.1960	6297.24463	452.81622	49.9888

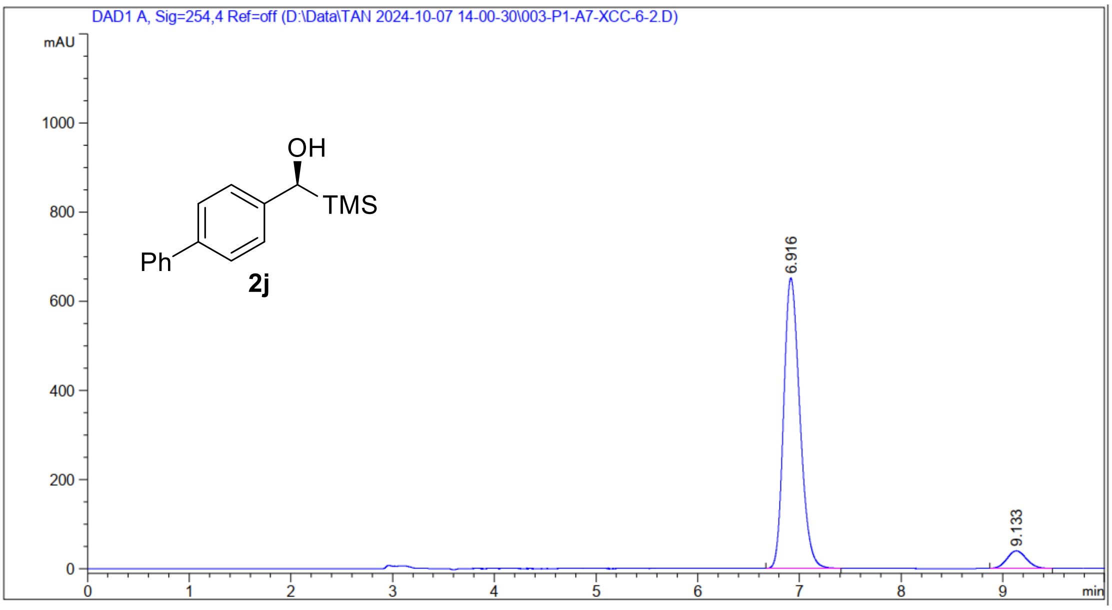

Peak #	RetTime [min]	Type	Width [min]	Area [mAU*s]	Height [mAU]	Area %
1	6.916	BB	0.1632	7164.98730	651.34113	93.2691
2	9.133	BB	0.1545	517.06934	39.21046	6.7309

(*S*)-4-(Hydroxy(trimethylsilyl)methyl)phenol (2k)

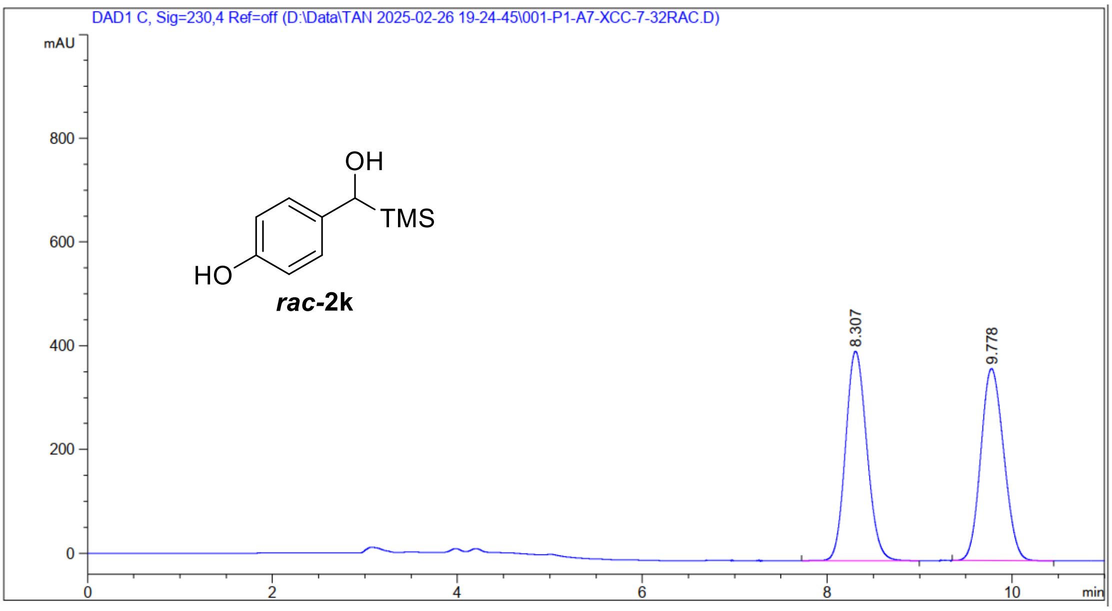

Peak #	RetTime [min]	Type	Width [min]	Area [mAU*s]	Height [mAU]	Area %
1	8.307	BB	0.2464	6452.06348	403.83145	50.1874
2	9.778	BB	0.2647	6403.86719	370.34589	49.8126

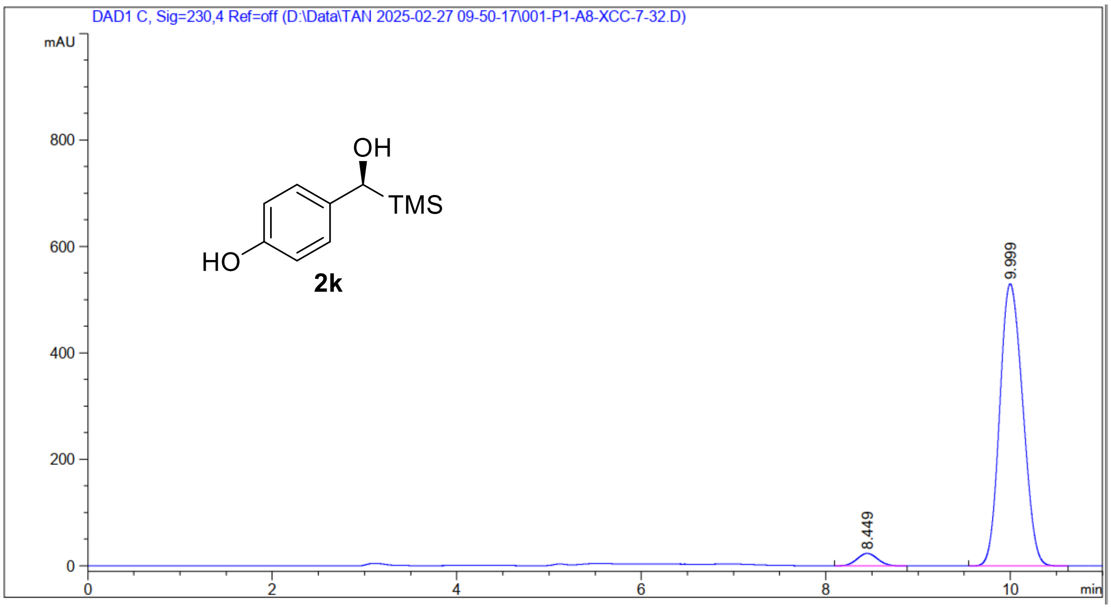

Peak #	RetTime [min]	Type	Width [min]	Area [mAU*s]	Height [mAU]	Area %
1	8.449	BB	0.2001	364.48062	23.41961	3.8176
2	9.999	BB	0.2681	9182.97754	530.04736	96.1824

(*S*)-Naphthalen-2-yl(trimethylsilyl)methanol (2l)

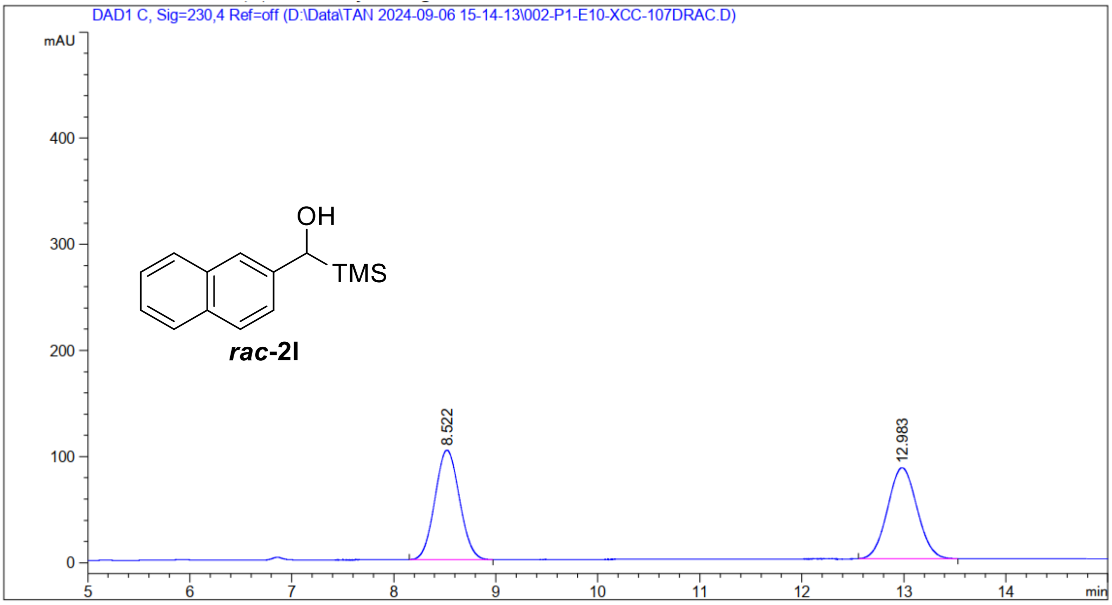

Peak #	RetTime [min]	Type	Width [min]	Area [mAU*s]	Height [mAU]	Area %
1	8.522	BB	0.1977	1719.17395	103.12933	50.0136
2	12.983	BB	0.2363	1718.24219	85.74165	49.9864

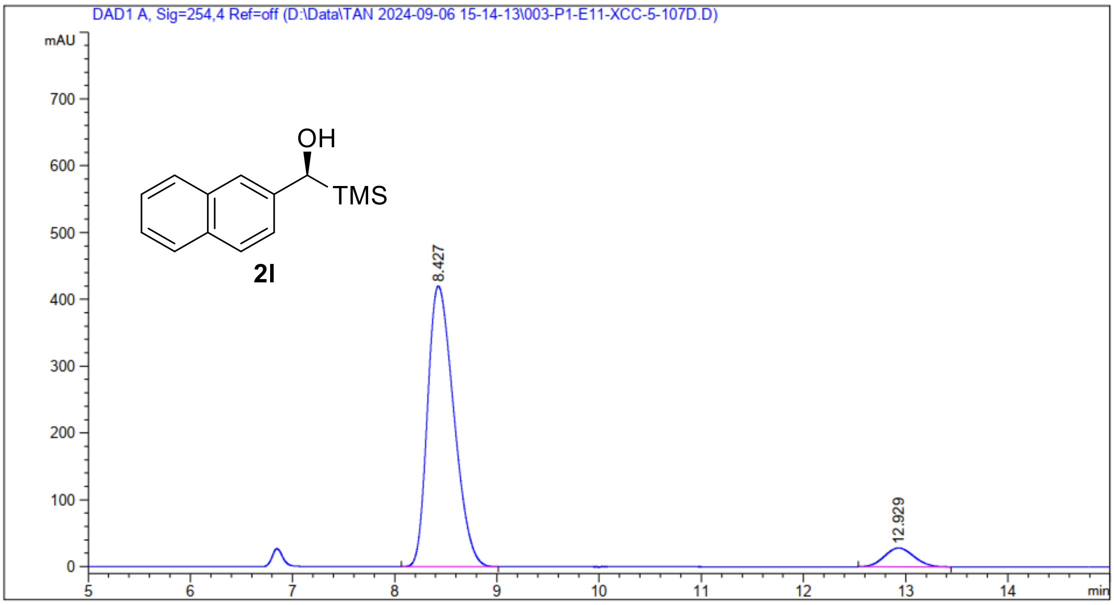

Peak #	RetTime [min]	Type	Width [min]	Area [mAU*s]	Height [mAU]	Area %
1	8.427	BB	0.2405	7463.18896	420.13156	92.9749
2	12.929	BB	0.2349	563.90778	28.12954	7.0251

(*S*)-(3,4-Dimethylphenyl)(trimethylsilyl)methanol (2m)

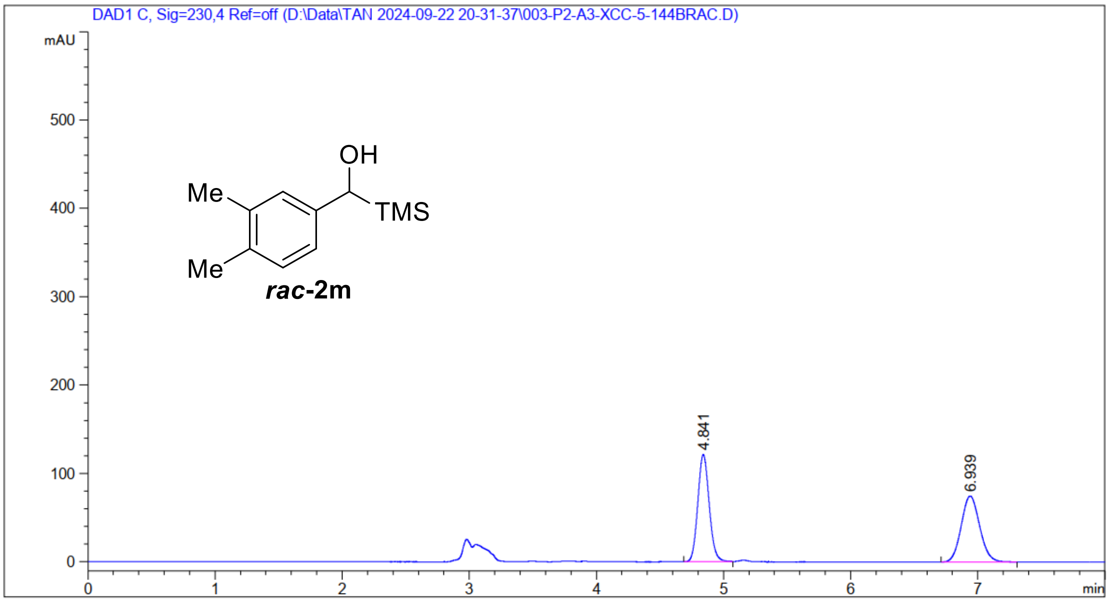

Peak #	RetTime [min]	Type	Width [min]	Area [mAU*s]	Height [mAU]	Area %
1	4.841	BB	0.0954	753.11749	121.28550	50.0111
2	6.939	BB	0.1416	752.78247	74.68680	49.9889

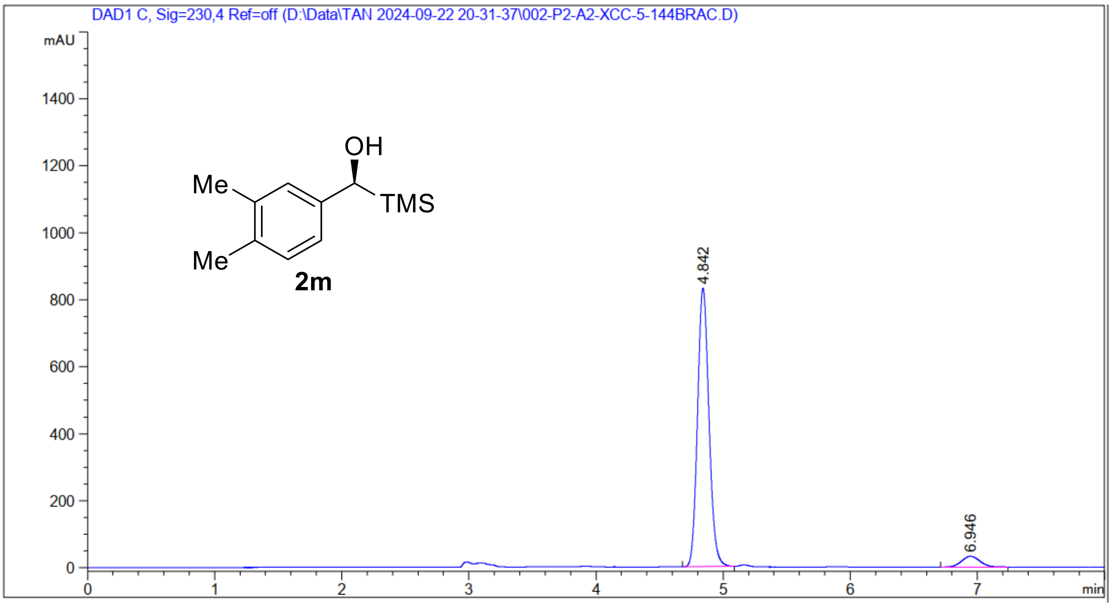

Peak #	RetTime [min]	Type	Width [min]	Area [mAU*s]	Height [mAU]	Area %
1	4.842	BB	0.0972	5196.46631	831.46716	94.2106
2	6.946	BB	0.1178	319.33279	32.17984	5.7894

(*S*)-(3,5-Dimethylphenyl)(trimethylsilyl)methanol (2n)

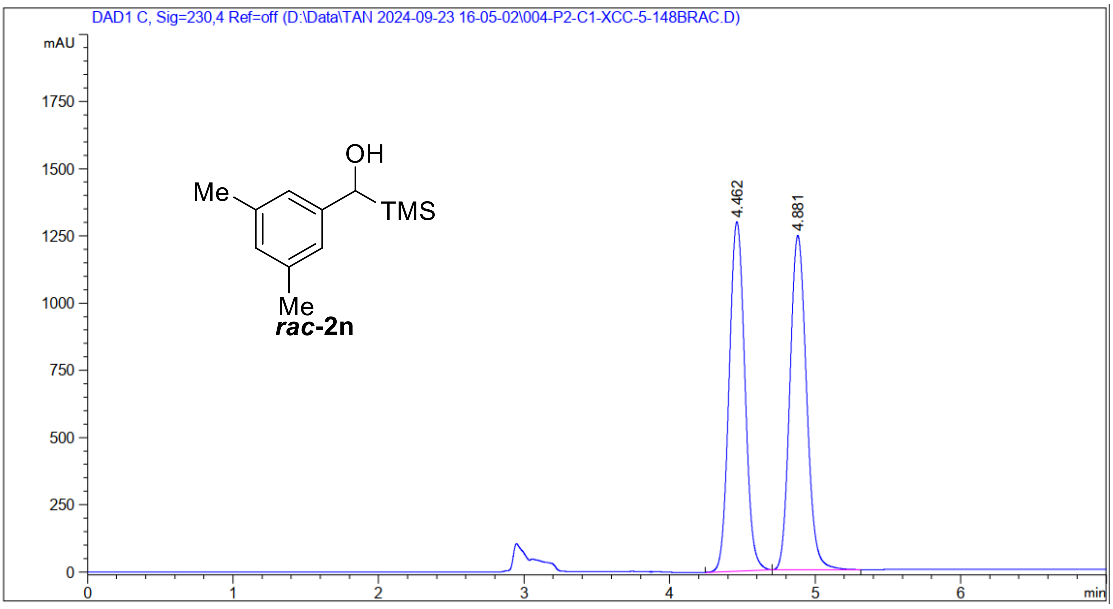

Peak #	RetTime [min]	Type	Width [min]	Area [mAU*s]	Height [mAU]	Area %
1	4.462	BB	0.1180	9740.73047	1300.08984	49.7254
2	4.881	BB	0.1228	9848.30273	1243.63184	50.2746

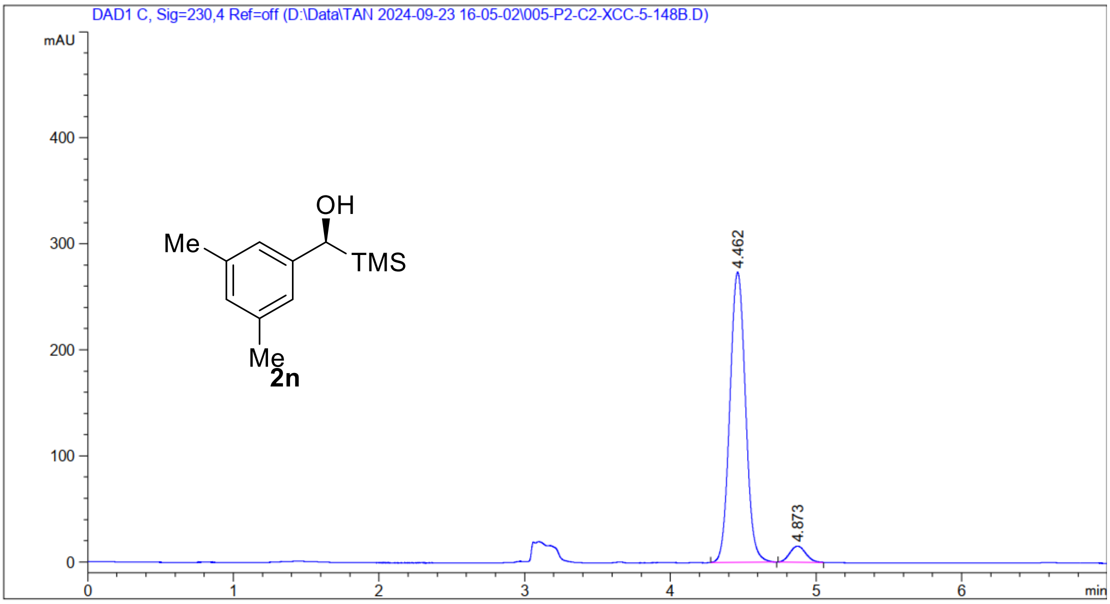

Peak #	RetTime [min]	Type	Width [min]	Area [mAU*s]	Height [mAU]	Area %
1	4.462	BB	0.1147	2039.08850	273.70169	94.7144
2	4.873	BB	0.0887	113.79297	15.10122	5.2856

(*S*)-(3,4-Dimethoxyphenyl)(trimethylsilyl)methanol (2o)

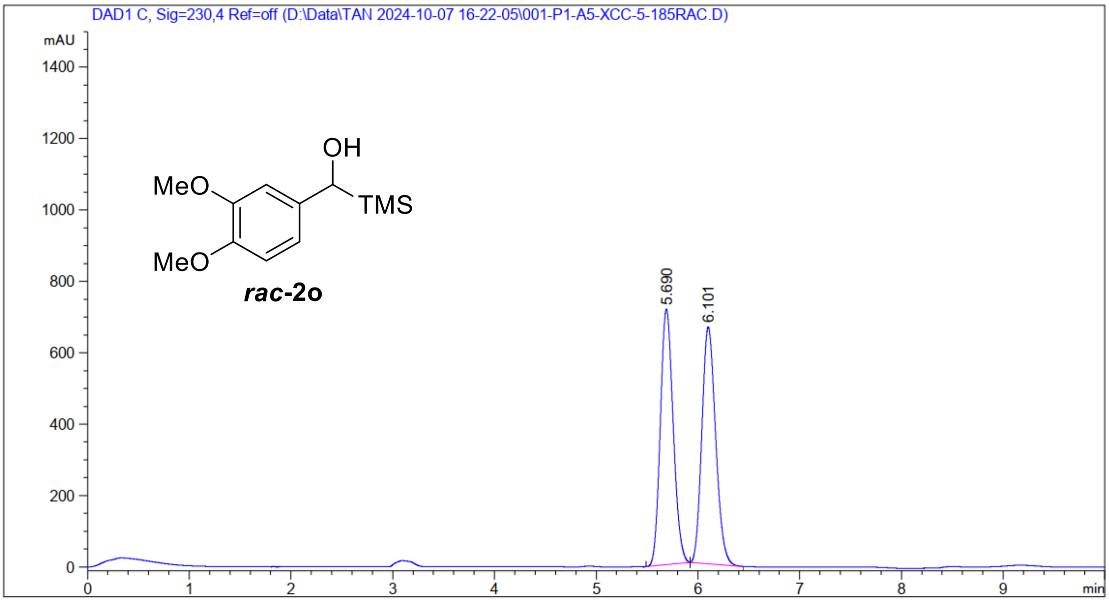

Peak #	RetTime [min]	Type	Width [min]	Area [mAU*s]	Height [mAU]	Area %
1	5.690	BB	0.1352	6300.49463	716.71497	50.2499
2	6.101	BB	0.1448	6237.82568	663.68109	49.7501

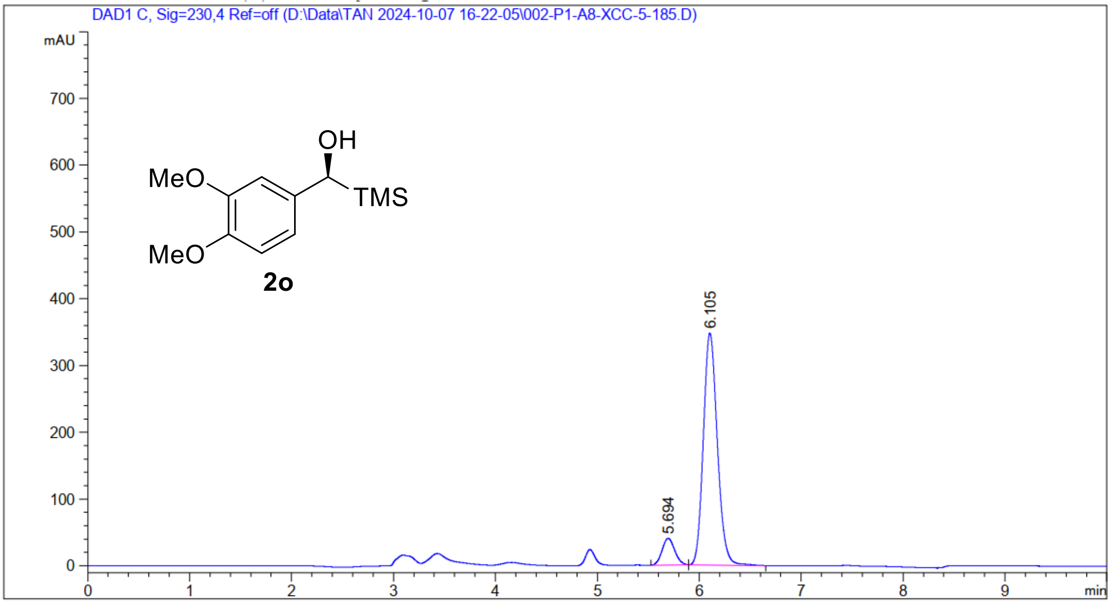

Peak #	RetTime [min]	Type	Width [min]	Area [mAU*s]	Height [mAU]	Area %
1	5.694	BB	0.1052	341.89828	39.85588	9.3729
2	6.105	BB	0.1446	3305.82104	347.52756	90.6271

(*S*)-(3,5-Dimethoxyphenyl)(trimethylsilyl)methanol (2p)

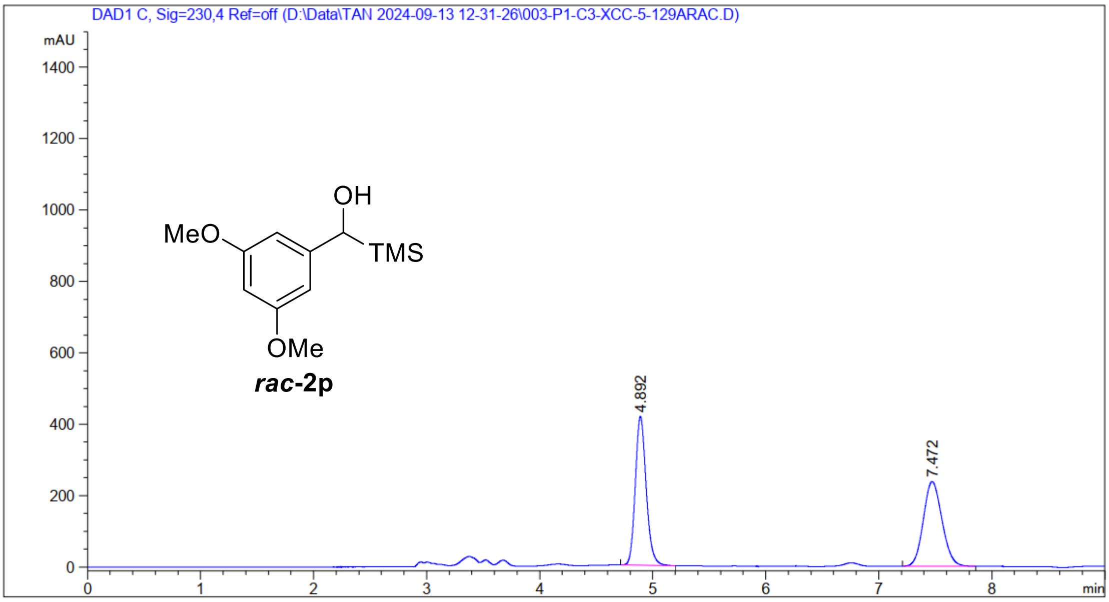

Peak #	RetTime [min]	Type	Width [min]	Area [mAU*s]	Height [mAU]	Area %
1	4.892	BB	0.1019	2798.35034	417.07315	50.7853
2	7.472	BB	0.1591	2711.81152	236.99205	49.2147

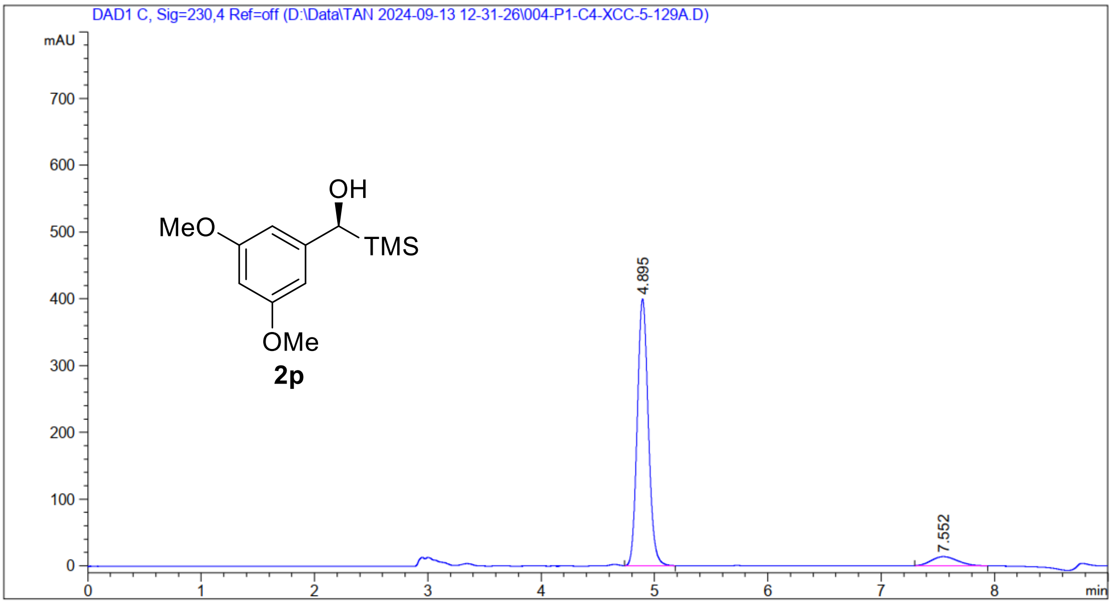

Peak #	RetTime [min]	Type	Width [min]	Area [mAU*s]	Height [mAU]	Area %
1	4.895	BB	0.1042	2702.28003	399.46021	92.5577
2	7.552	BB	0.1872	217.28110	13.55641	7.4423

(*S*)-(2,3-Dihydrobenzofuran-5-yl)(trimethylsilyl)methanol (2q)

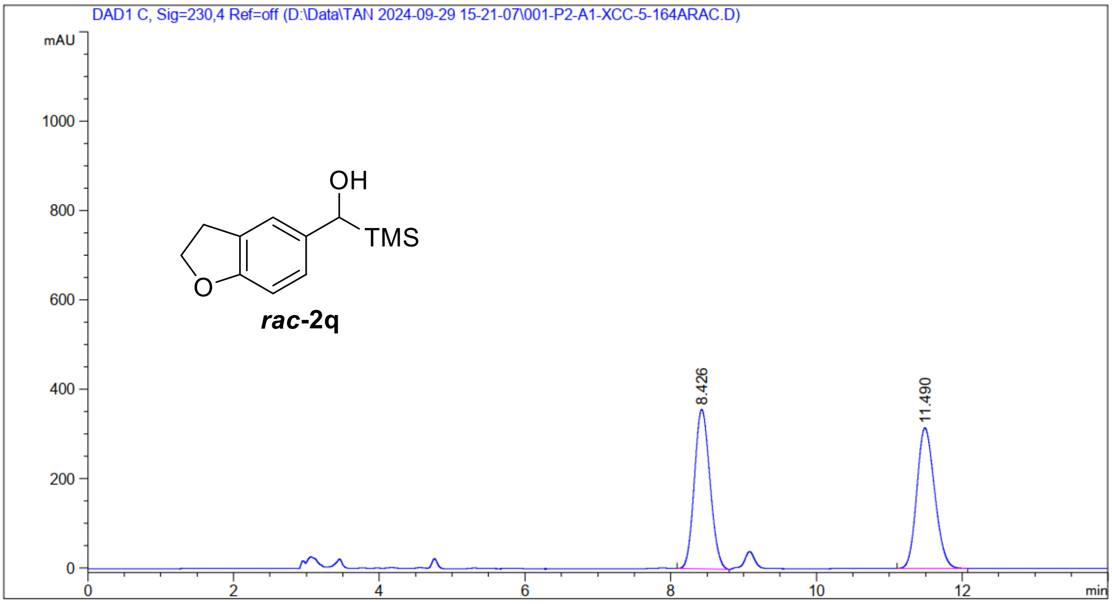

Peak #	RetTime [min]	Type	Width [min]	Area [mAU*s]	Height [mAU]	Area %
1	8.426	BB	0.2116	5328.07080	357.31839	49.9027
2	11.490	BB	0.2359	5348.84570	314.15219	50.0973

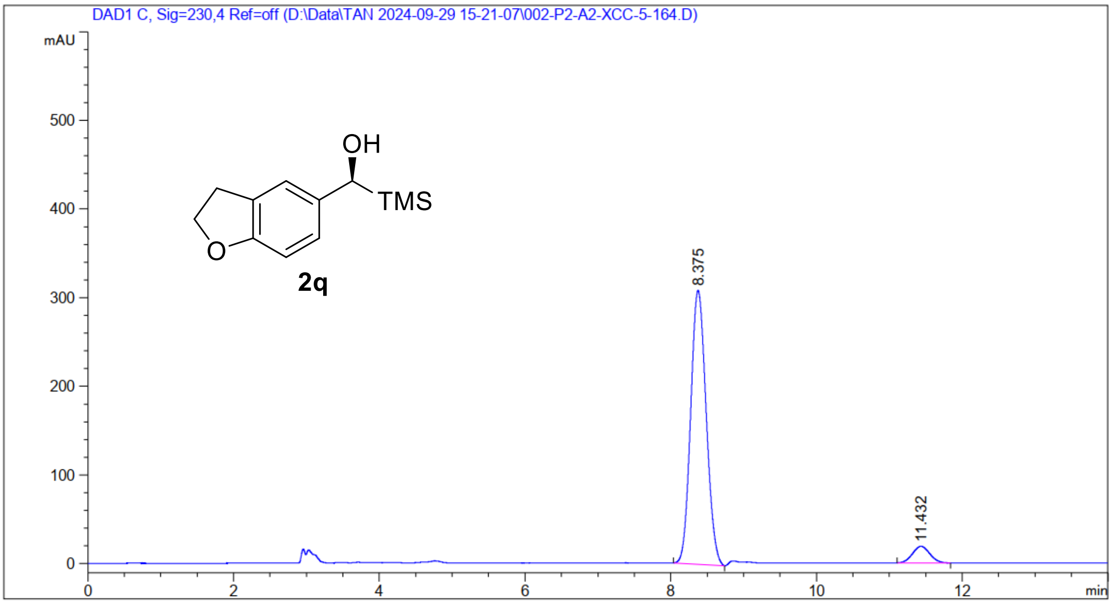

Peak #	RetTime [min]	Type	Width [min]	Area [mAU*s]	Height [mAU]	Area %
1	8.375	BB	0.2100	4558.03271	308.80978	93.6723
2	11.432	BB	0.1936	307.90393	18.69691	6.3277

(*S*)-(3,4,5-Trimethoxyphenyl)(trimethylsilyl)methanol (2r)

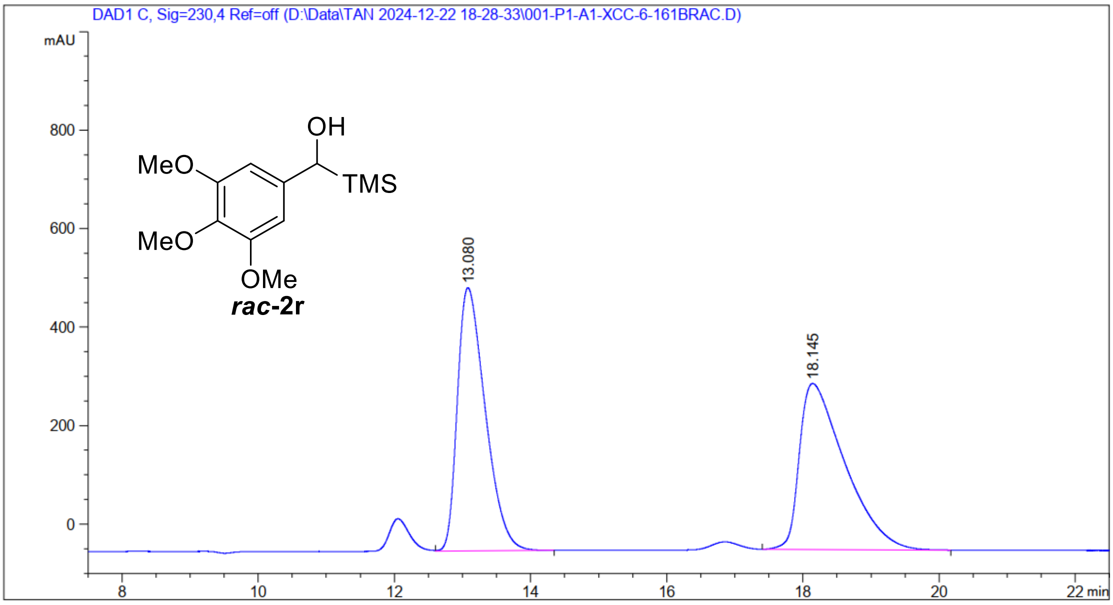

Peak #	RetTime [min]	Type	Width [min]	Area [mAU*s]	Height [mAU]	Area %
1	13.080	VB	0.4049	1.52084e4	534.51648	49.3049
2	18.145	BB	0.5734	1.56372e4	336.94989	50.6951

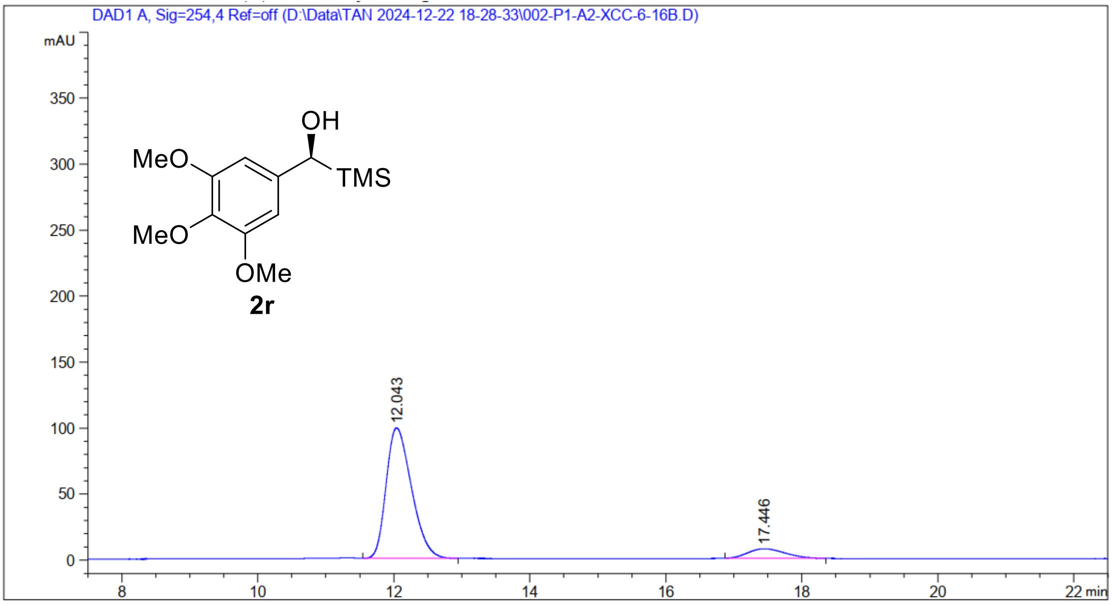

Peak #	RetTime [min]	Type	Width [min]	Area [mAU*s]	Height [mAU]	Area %
1	12.043	BB	0.3072	2557.57300	98.88496	90.2499
2	17.446	BB	0.4472	276.30725	7.24116	9.7501

(*S*)-(4-Fluorophenyl)(trimethylsilyl)methanol (2s)

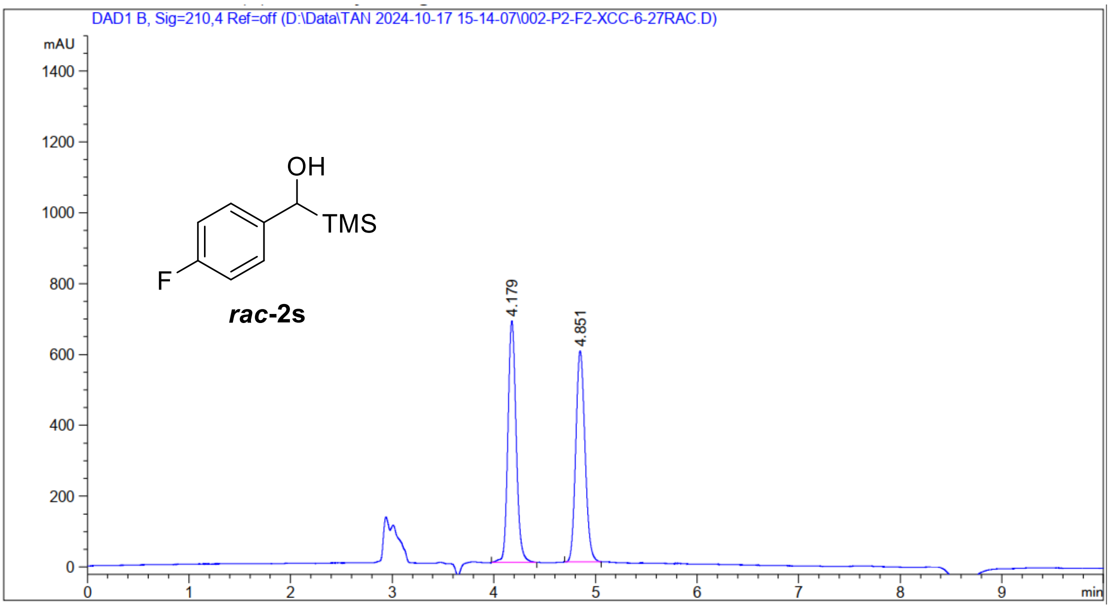

Peak #	RetTime [min]	Type	Width [min]	Area [mAU*s]	Height [mAU]	Area %
1	4.179	VV R	0.0668	3848.71704	681.51715	50.6229
2	4.851	VV R	0.0749	3754.00464	594.97705	49.3771

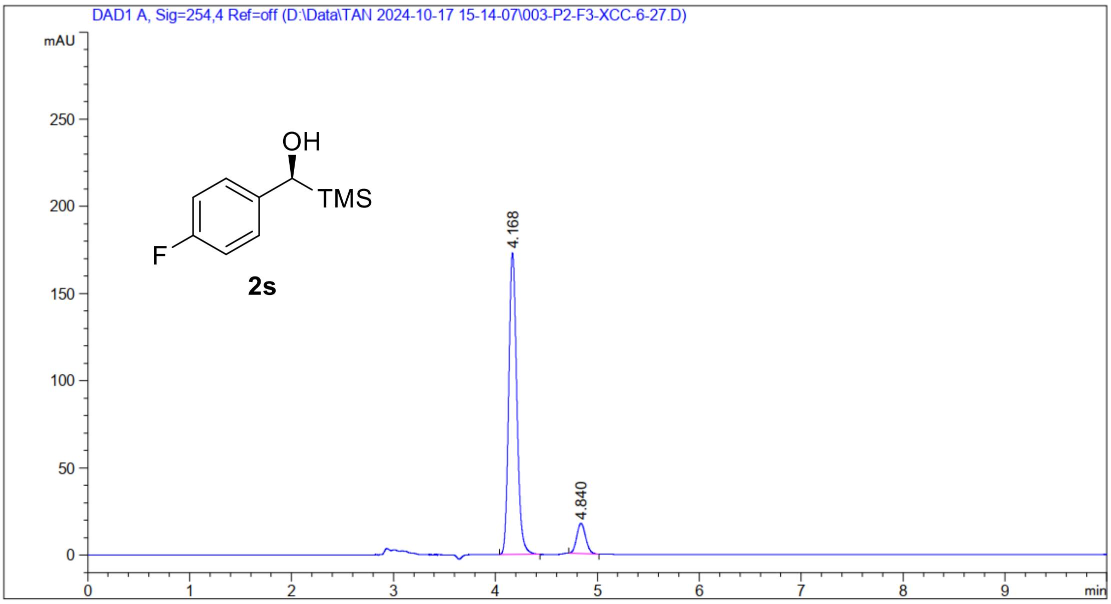

Peak #	RetTime [min]	Type	Width [min]	Area [mAU*s]	Height [mAU]	Area %
1	4.168	BB	0.0833	953.38055	173.05212	90.0204
2	4.840	BB	0.0720	105.69062	17.35915	9.9796

(*S*)-(3-Fluorophenyl)(trimethylsilyl)methanol (2t)

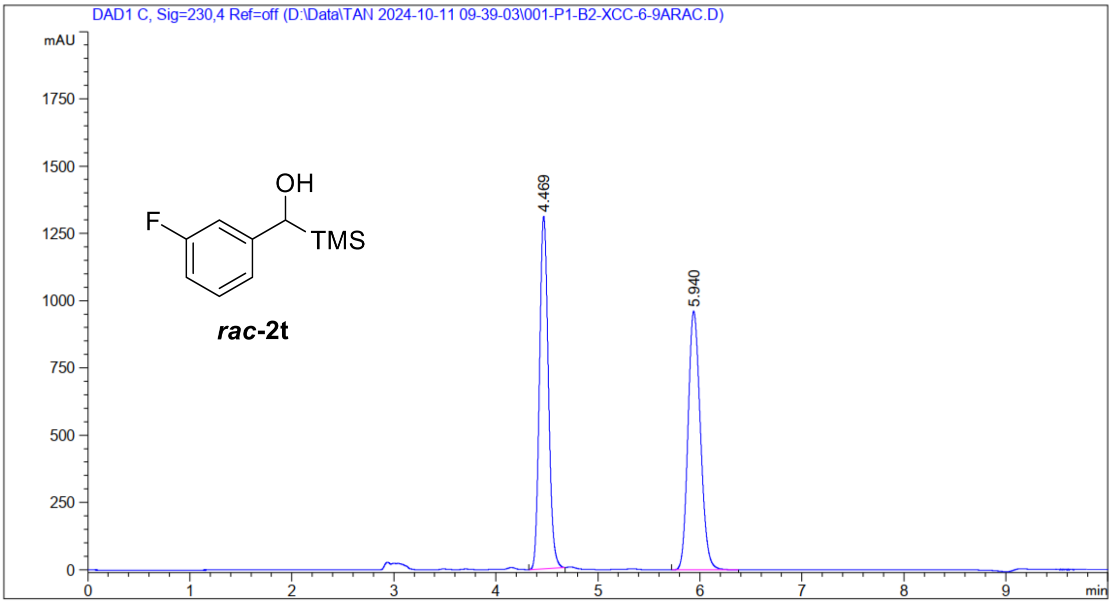

Peak #	RetTime [min]	Type	Width [min]	Area [mAU*s]	Height [mAU]	Area %
1	4.469	BB	0.0943	7909.77246	1310.51721	49.3626
2	5.940	BB	0.1298	8114.04150	961.45618	50.6374

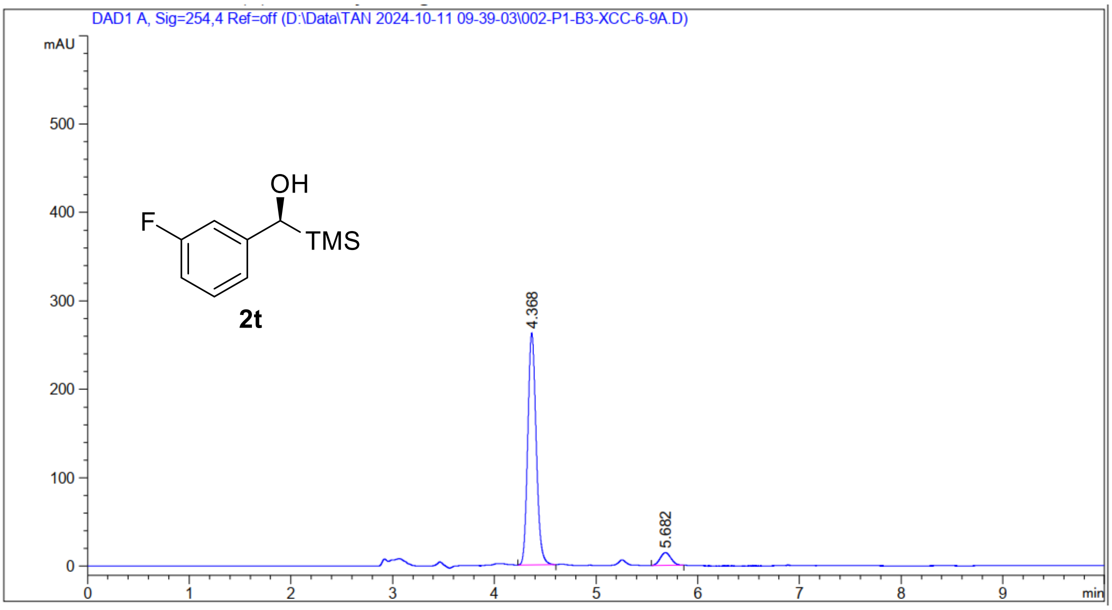

Peak #	RetTime [min]	Type	Width [min]	Area [mAU*s]	Height [mAU]	Area %
1	4.368	BB	0.0877	1505.26575	262.19183	93.3791
2	5.682	BB	0.0875	106.72832	14.43992	6.6209

(*S*)-(2-Fluorophenyl)(trimethylsilyl)methanol (2u)

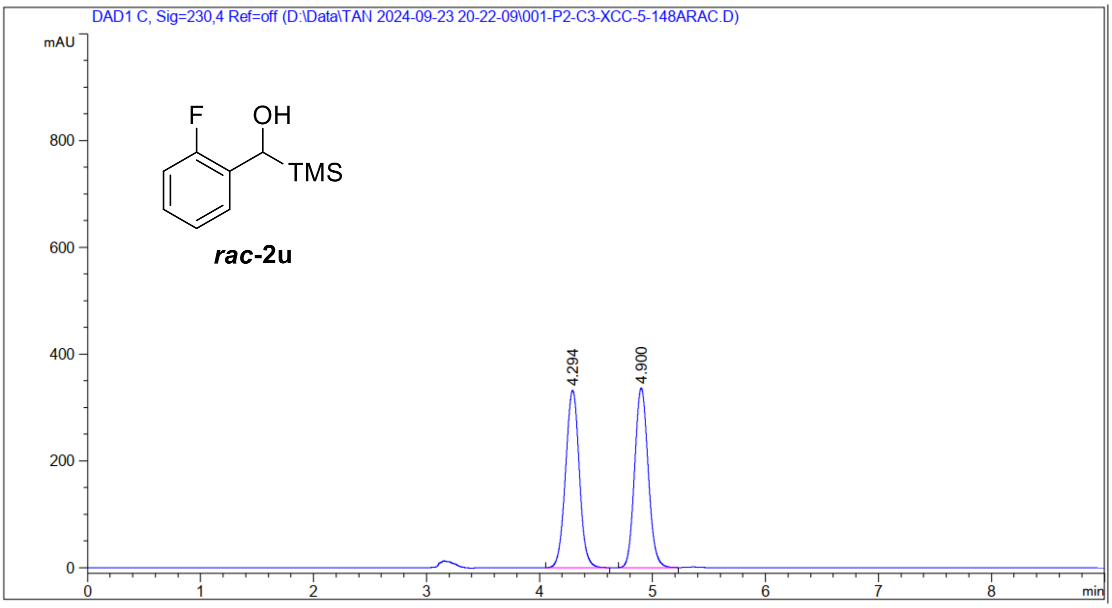

Peak #	RetTime [min]	Type	Width [min]	Area [mAU*s]	Height [mAU]	Area %
1	4.294	BB	0.1315	2819.55981	332.17838	50.1153
2	4.900	BB	0.1294	2806.58691	336.20316	49.8847

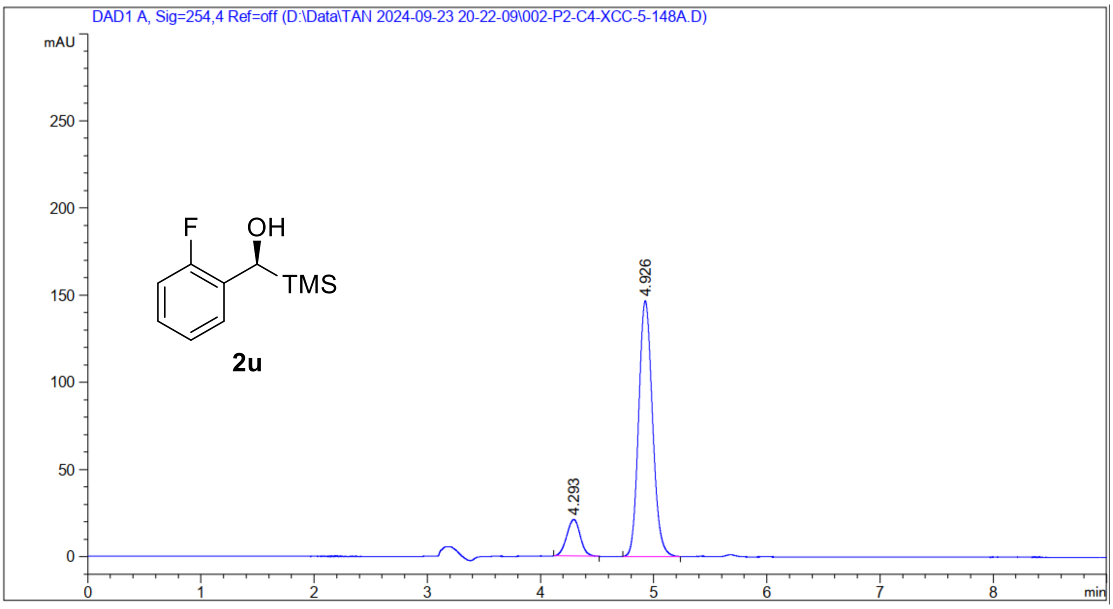

Peak #	RetTime [min]	Type	Width [min]	Area [mAU*s]	Height [mAU]	Area %
1	4.293	BB	0.0982	175.28568	20.99181	12.3499
2	4.926	BB	0.1272	1244.03784	146.62498	87.6501

(*S*)-(4-Chlorophenyl)(trimethylsilyl)methanol (2v)

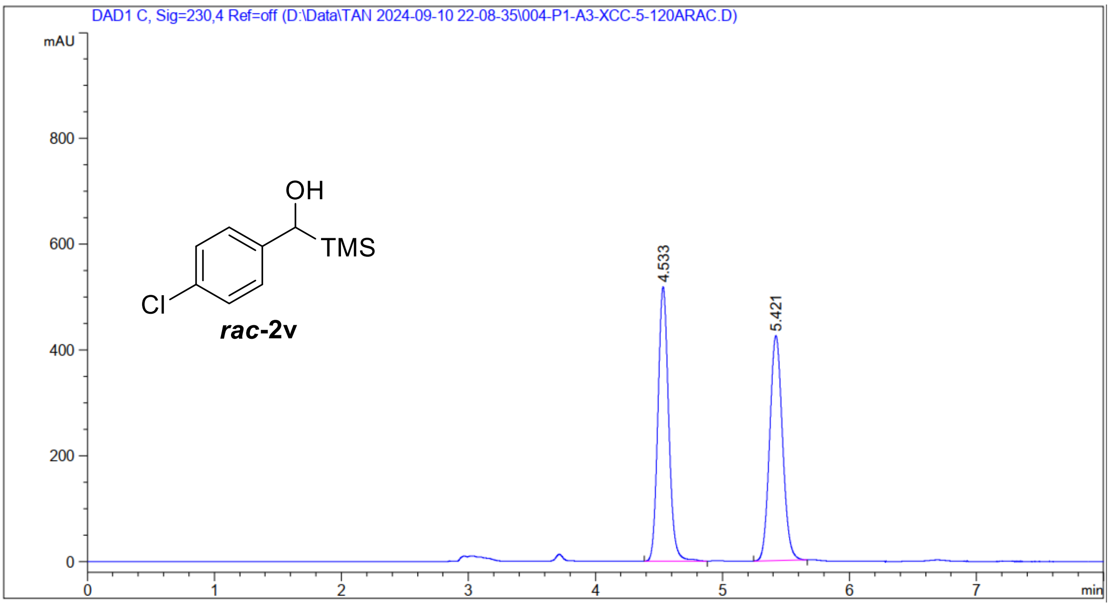

Peak #	RetTime [min]	Type	Width [min]	Area [mAU*s]	Height [mAU]	Area %
1	4.533	BB	0.0901	3038.57935	518.56390	50.4330
2	5.421	BB	0.1076	2986.39868	425.77988	49.5670

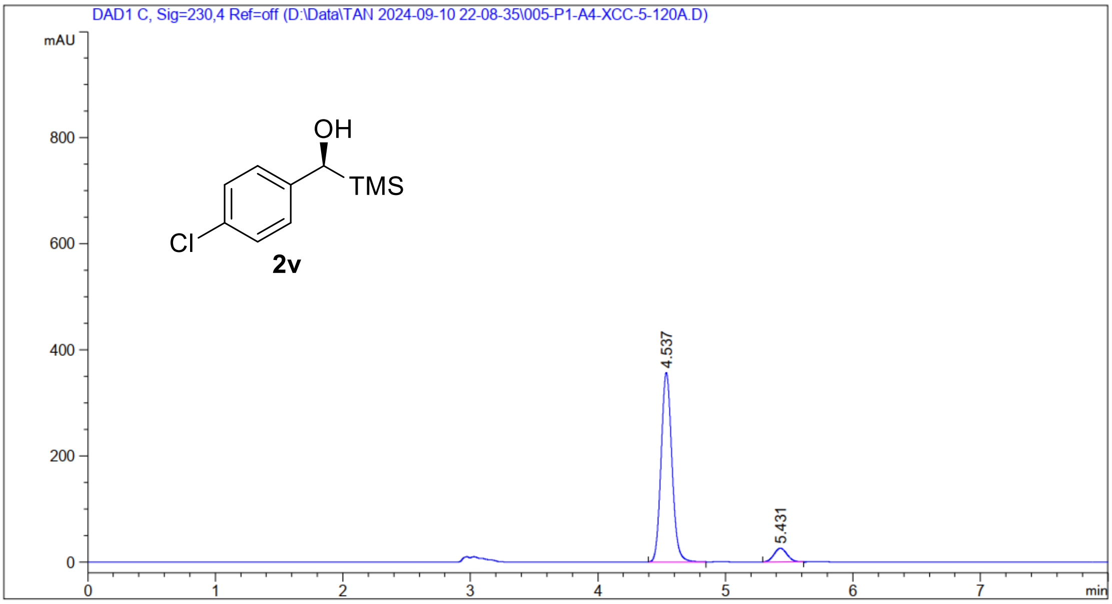

Peak #	RetTime [min]	Type	Width [min]	Area [mAU*s]	Height [mAU]	Area %
1	4.537	BB	0.0903	2109.61987	357.32703	92.0246
2	5.431	BB	0.0844	182.83295	25.85925	7.9754

(*S*)-(4-Bromophenyl)(trimethylsilyl)methanol (2w)

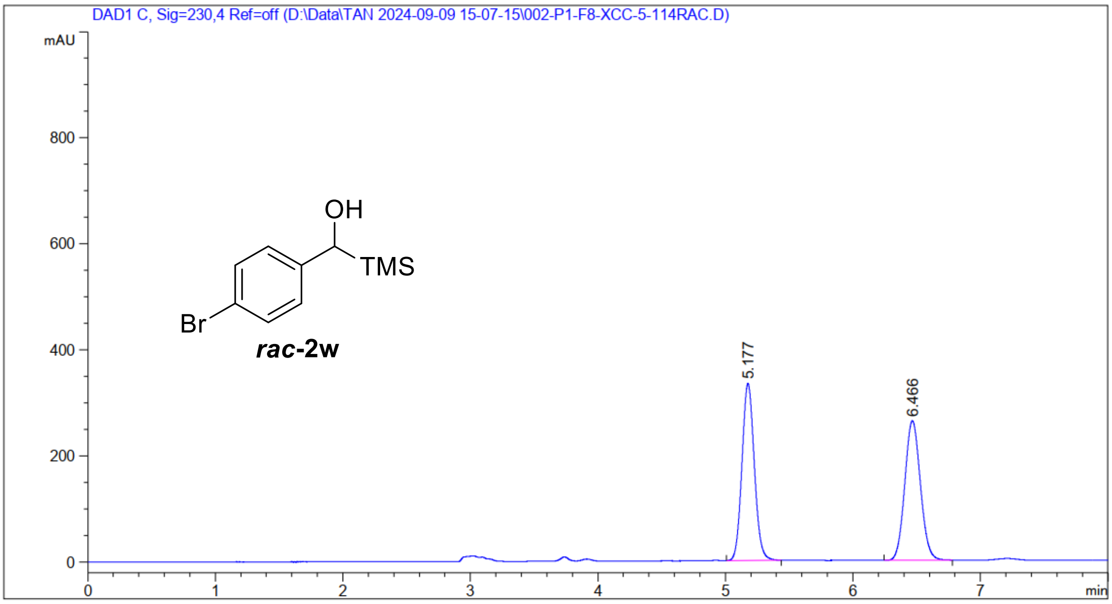

Peak #	RetTime [min]	Type	Width [min]	Area [mAU*s]	Height [mAU]	Area %
1	5.177	BB	0.1052	2250.65283	333.73514	50.0815
2	6.466	BB	0.1277	2243.32886	263.29794	49.9185

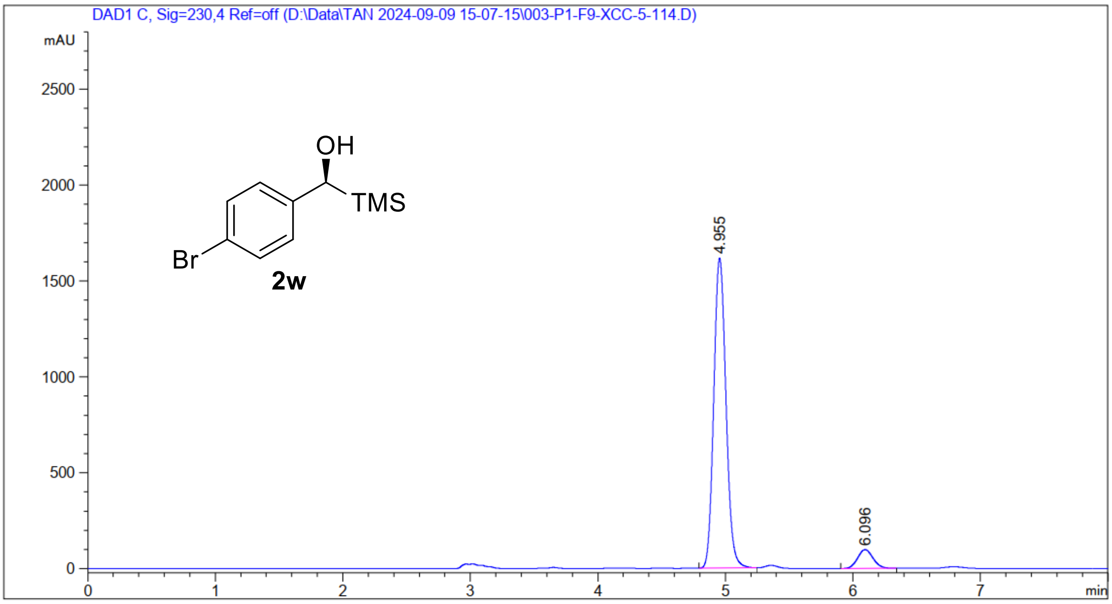

Peak #	RetTime [min]	Type	Width [min]	Area [mAU*s]	Height [mAU]	Area %
1	4.955	BB	0.1024	1.07257e4	1615.77515	92.8731
2	6.096	BB	0.1182	823.06653	98.06172	7.1269

(*S*)-(3-Chlorophenyl)(trimethylsilyl)methanol (2x)

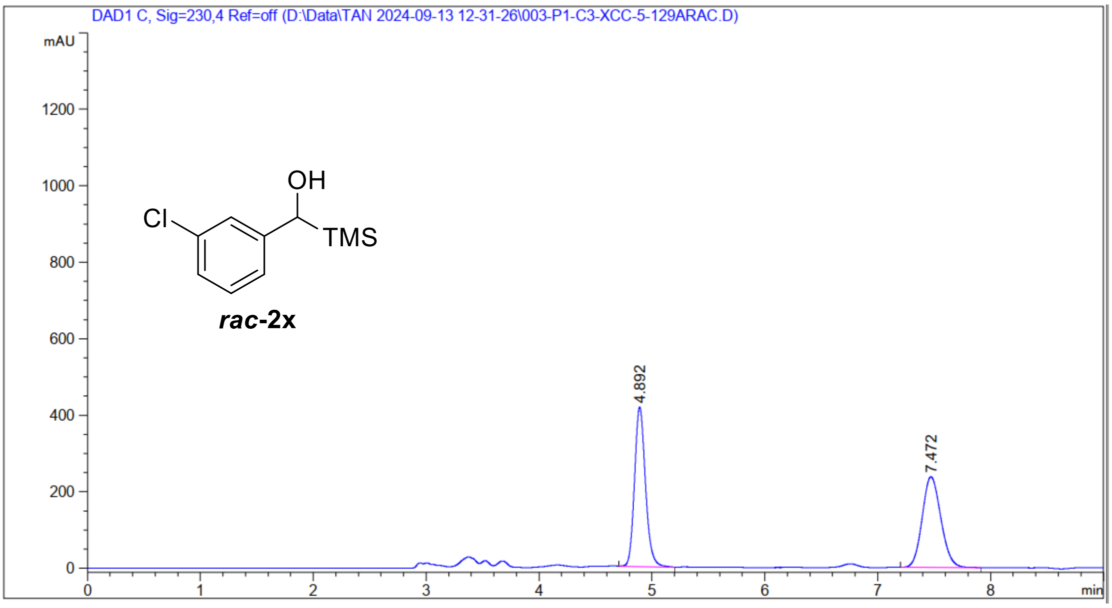

Peak #	RetTime [min]	Type	Width [min]	Area [mAU*s]	Height [mAU]	Area %
1	4.892	BB	0.1037	2799.17603	417.11002	50.7461
2	7.472	BB	0.1712	2716.86670	237.10440	49.2539

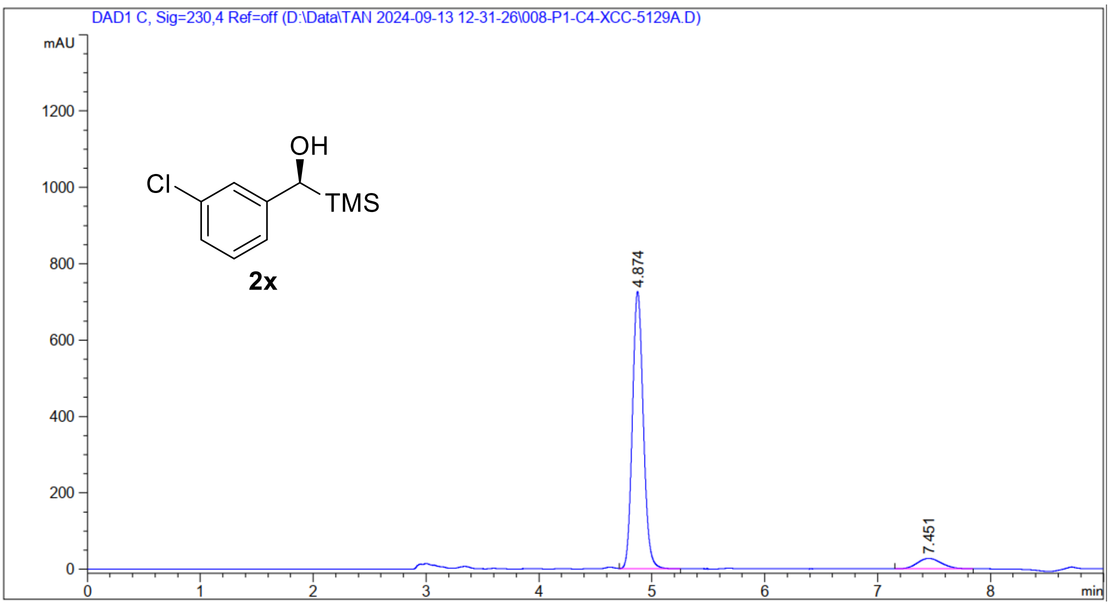

Peak #	RetTime [min]	Type	Width [min]	Area [mAU*s]	Height [mAU]	Area %
1	4.874	BB	0.1035	4850.83252	725.80487	92.2416
2	7.451	BB	0.1742	407.99878	27.44214	7.7584

(*S*)-(3-Bromophenyl)(trimethylsilyl)methanol (2y)

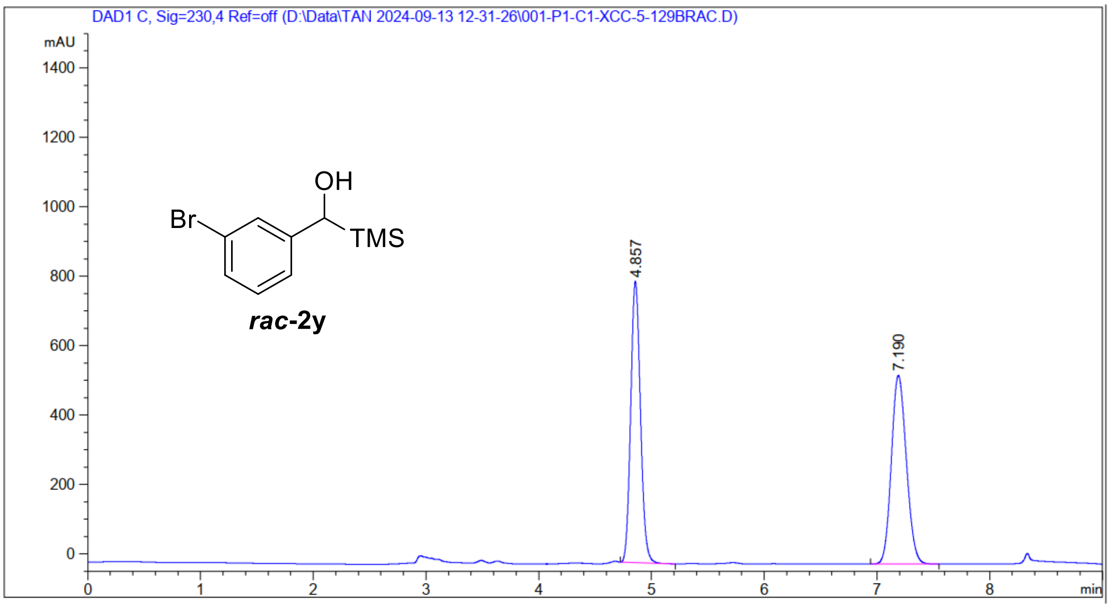

Peak #	RetTime [min]	Type	Width [min]	Area [mAU*s]	Height [mAU]	Area %
1	4.857	BB	0.0935	4865.32080	811.04492	49.2385
2	7.190	BB	0.1424	5015.81152	543.58459	50.7615

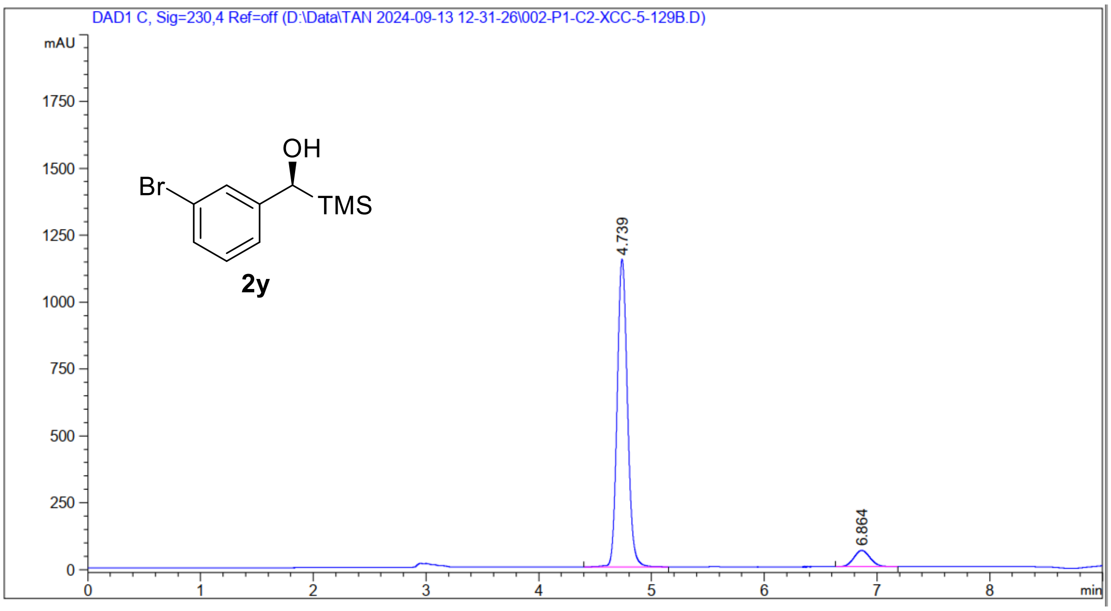

Peak #	RetTime [min]	Type	Width [min]	Area [mAU*s]	Height [mAU]	Area %
1	4.739	BB	0.0987	7286.50195	1150.18323	92.2896
2	6.864	BB	0.1400	608.75189	60.82007	7.7104

(*S*)-Thiophen-2-yl(trimethylsilyl)methanol (2z)

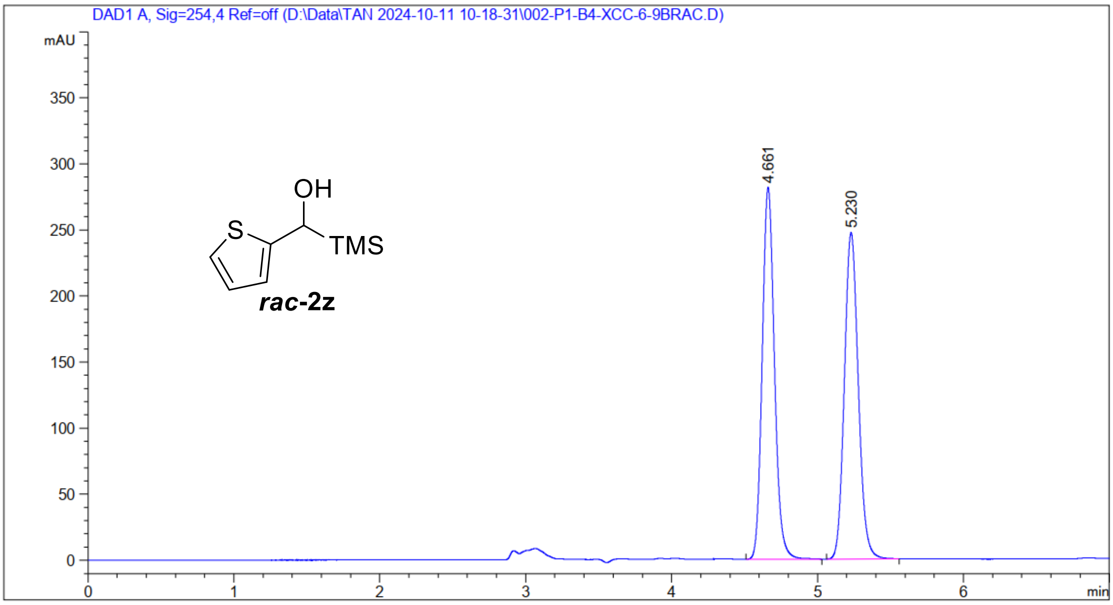

Peak #	RetTime [min]	Type	Width [min]	Area [mAU*s]	Height [mAU]	Area %
1	4.661	BB	0.0896	1637.06860	281.36581	50.0378
2	5.230	BB	0.1020	1634.59790	246.99731	49.9622

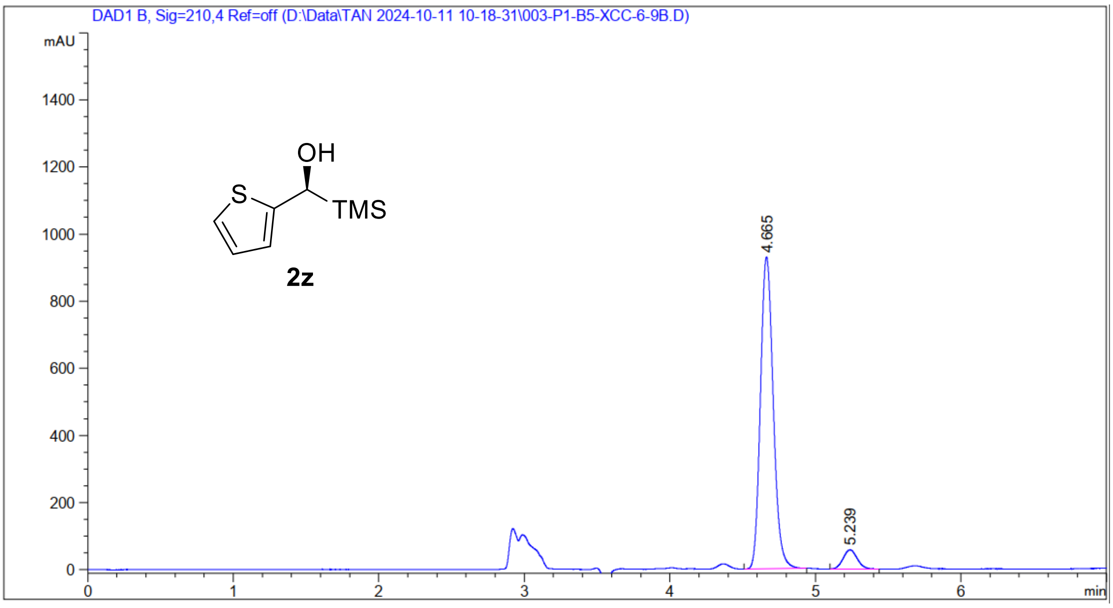

Peak #	RetTime [min]	Type	Width [min]	Area [mAU*s]	Height [mAU]	Area %
1	4.665	BB	0.0731	5680.97070	928.51733	93.6085
2	5.239	BB	0.0797	387.89407	57.46755	6.3915

(*S*)-Furan-2-yl(trimethylsilyl)methanol (2aa)

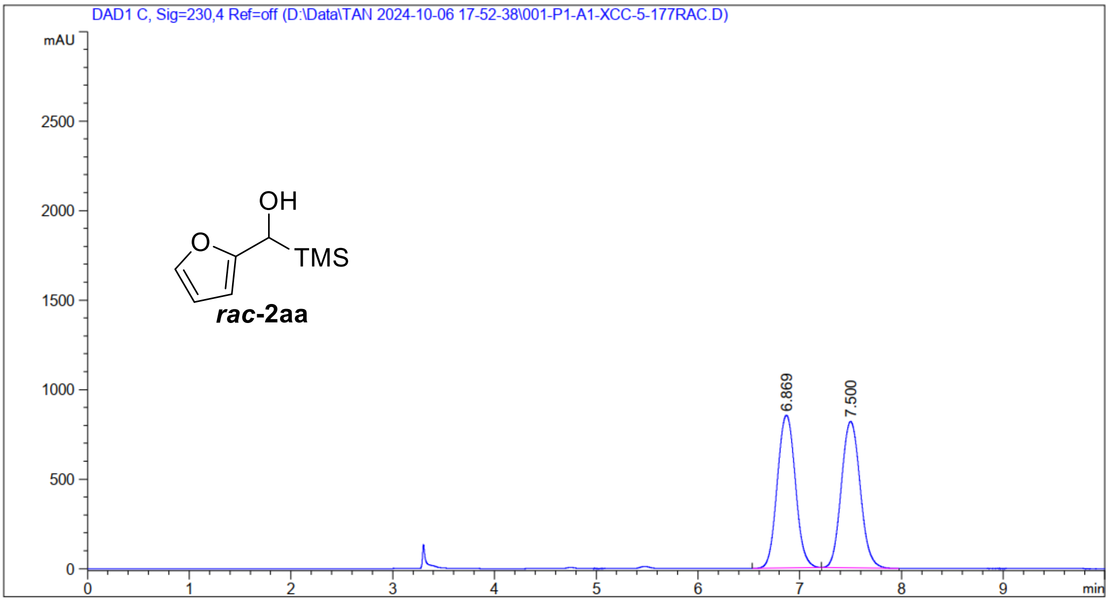

Peak #	RetTime [min]	Type	Width [min]	Area [mAU*s]	Height [mAU]	Area %
1	6.869	BB	0.1917	1.05653e4	853.44714	49.9959
2	7.500	BB	0.1965	1.05670e4	817.53333	50.0041

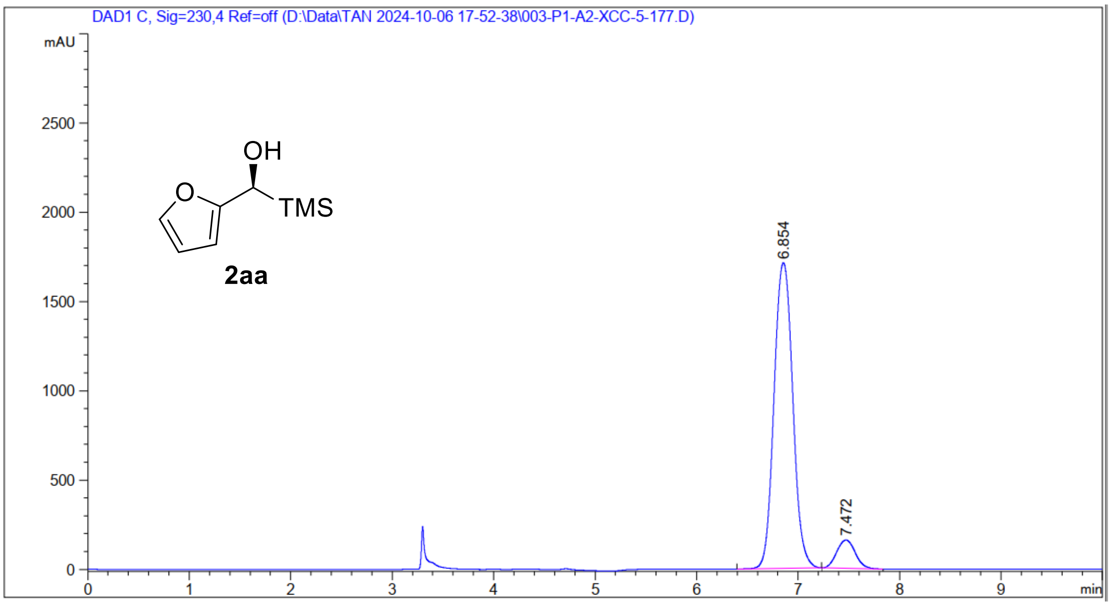

Peak #	RetTime [min]	Type	Width [min]	Area [mAU*s]	Height [mAU]	Area %
1	6.854	BB	0.1560	2.19312e4	1711.37878	91.6206
2	7.472	BB	0.1508	2005.77075	158.17307	8.3794

(*S*)-4-(Hydroxy(trimethylsilyl)methyl)phenyl 4-(*N*,*N*-dipropylsulfamoyl) benzoate (2ab)

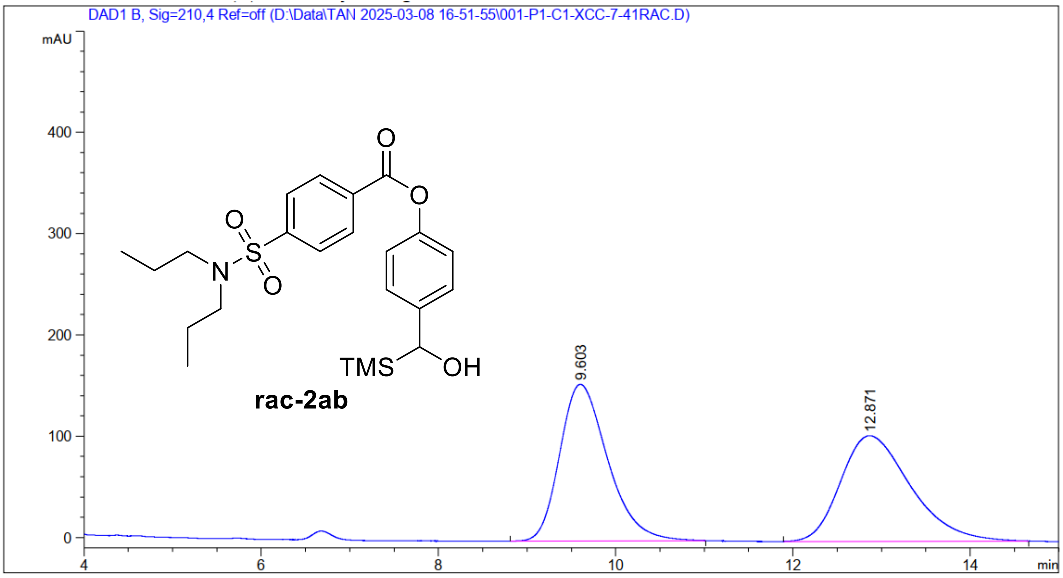

Peak #	RetTime [min]	Type	Width [min]	Area [mAU*s]	Height [mAU]	Area %
1	9.603	BB	0.4416	5806.49658	154.63449	50.3897
2	12.871	BB	0.6412	5716.68945	104.26207	49.6103

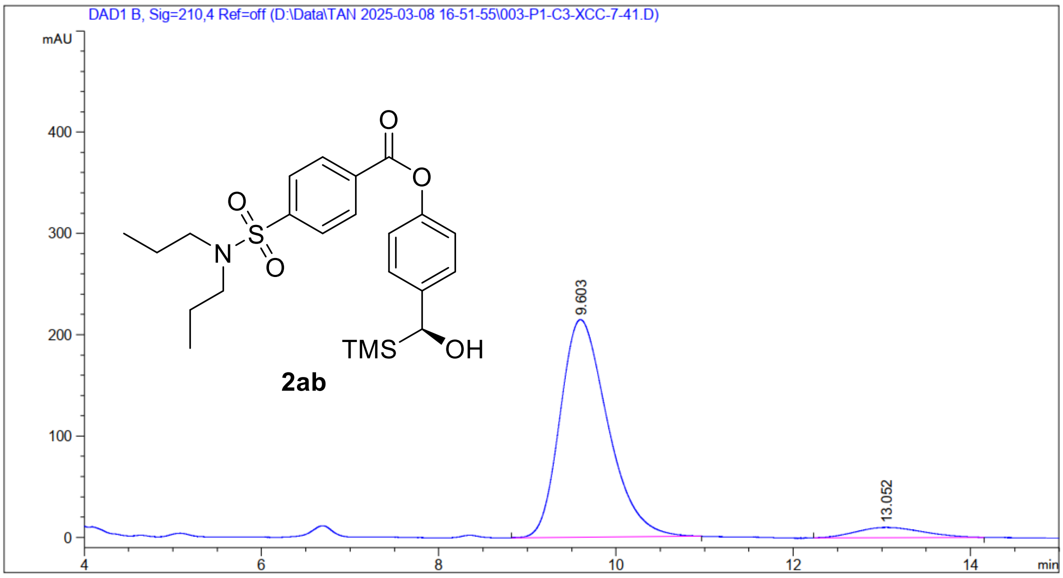

Peak #	RetTime [min]	Type	Width [min]	Area [mAU*s]	Height [mAU]	Area %
1	9.603	BB	0.4404	8018.19141	214.83624	93.7112
2	13.052	BB	0.6161	538.08618	10.21388	6.2888

(*S*)-4-(Hydroxy(trimethylsilyl)methyl)phenyl 2-(1-(4-chlorobenzoyl)-5-methoxy-2-methyl-1*H*-indol-3-yl)acetate (2ac)

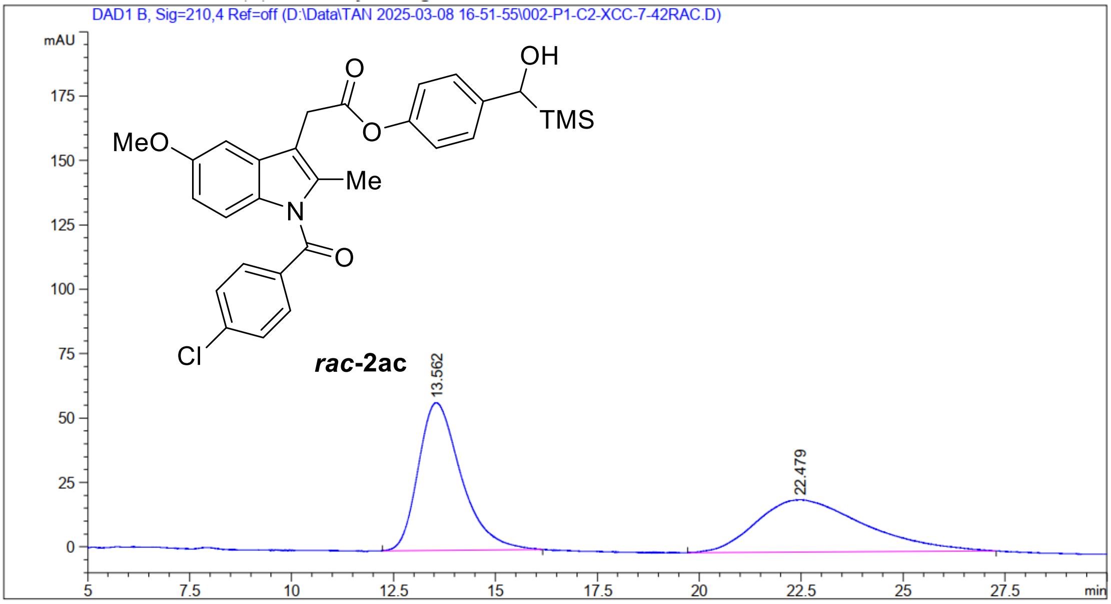

Peak #	RetTime [min]	Type	Width [min]	Area [mAU*s]	Height [mAU]	Area %
1	13.562	BB	0.8597	4180.80811	57.27035	52.7993
2	22.479	BB	2.1481	3737.49609	20.32753	47.2007

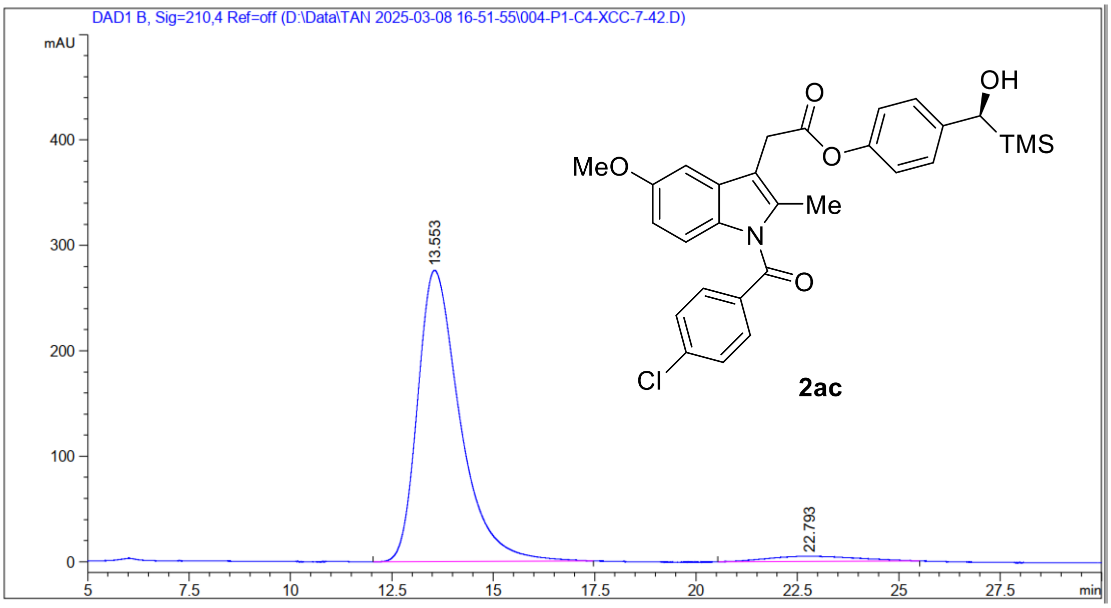

Peak #	RetTime [min]	Type	Width [min]	Area [mAU*s]	Height [mAU]	Area %
1	13.553	BB	0.8630	2.00614e4	276.31781	96.2699
2	22.793	BB	1.8505	777.30157	4.93802	3.7301

(*S*)-4-(Hydroxy(trimethylsilyl)methyl)phenyl 3-(4,5-diphenyloxazol-2-yl)propanoate (2ad)

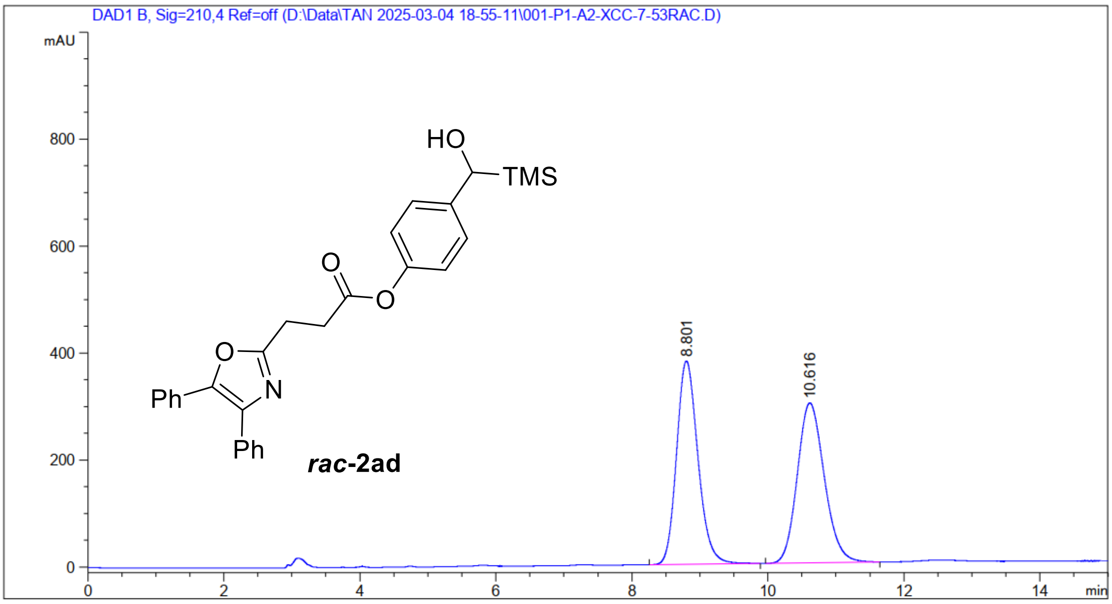

Peak #	RetTime [min]	Type	Width [min]	Area [mAU*s]	Height [mAU]	Area %
1	8.801	BB	0.3079	8289.20020	380.06766	50.0888
2	10.616	BB	0.3393	8259.82227	298.95059	49.9112

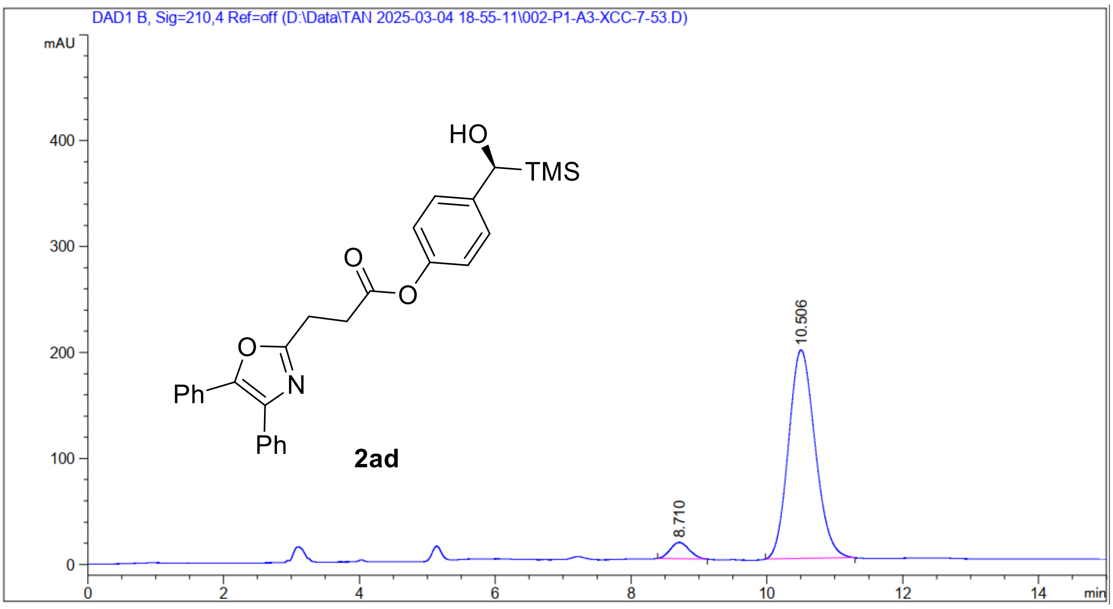

Peak #	RetTime [min]	Type	Width [min]	Area [mAU*s]	Height [mAU]	Area %
1	8.710	BB	0.2264	298.37320	15.41937	5.2894
2	10.506	BB	0.3177	5342.54639	196.83578	94.7106

(*S*)-4-(Hydroxy(trimethylsilyl)methyl)phenyl 5-(2,5-dimethylphenoxy)-2,2-dimethylpentanoate (2ae)

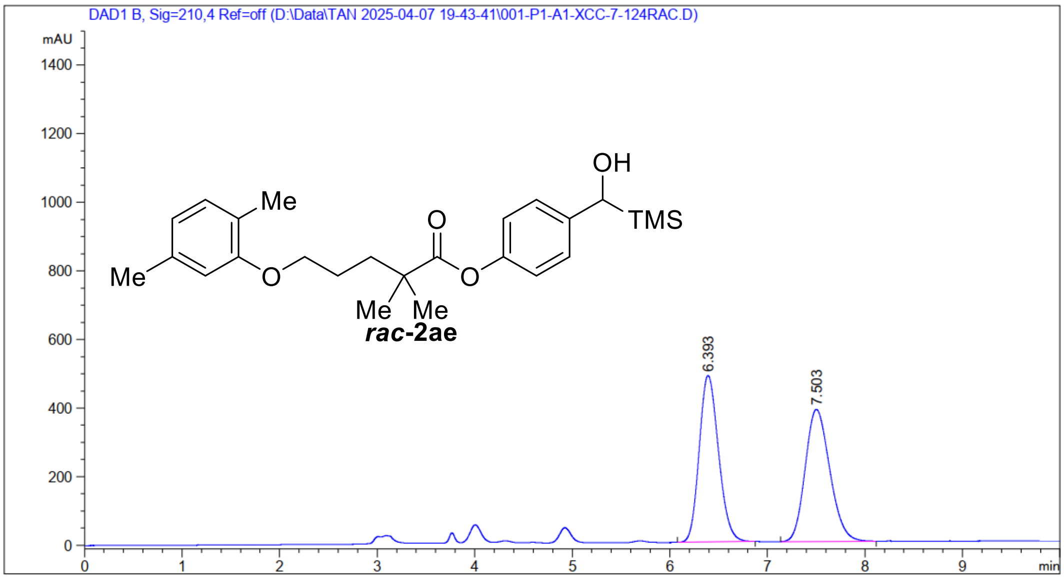

Peak #	RetTime [min]	Type	Width [min]	Area [mAU*s]	Height [mAU]	Area %
1	6.393	BB	0.1662	6687.80078	485.86334	49.7817
2	7.503	BB	0.2050	6746.46338	384.80957	50.2183

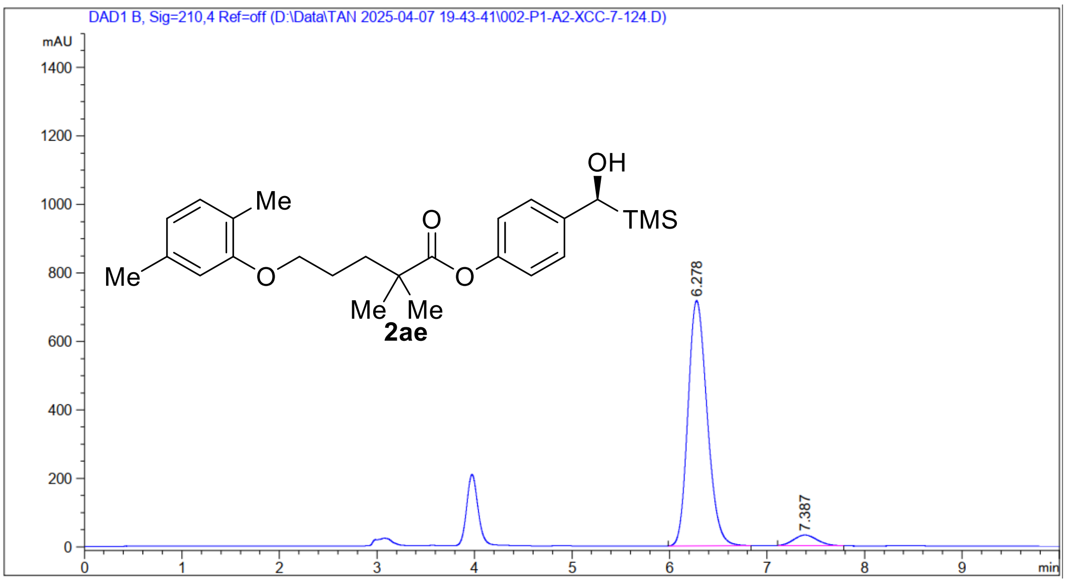

Peak #	RetTime [min]	Type	Width [min]	Area [mAU*s]	Height [mAU]	Area %
1	6.278	BB	0.1718	9770.98730	716.22662	95.0063
2	7.387	BB	0.1956	513.57562	30.73561	4.9937

(*S*)-3-Phenyl-1-(triethylsilyl)propan-1-ol (4a)

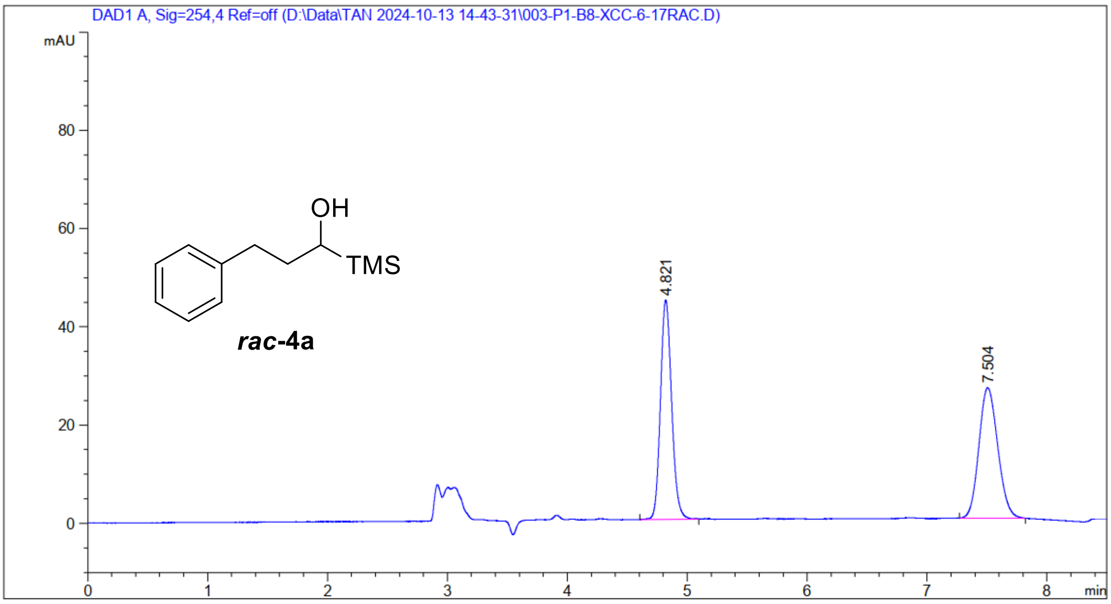

Peak #	RetTime [min]	Type	Width [min]	Area [mAU*s]	Height [mAU]	Area %
1	4.821	BV R	0.0900	289.08710	44.68517	49.9324
2	7.504	BB	0.1296	289.87024	26.54908	50.0676

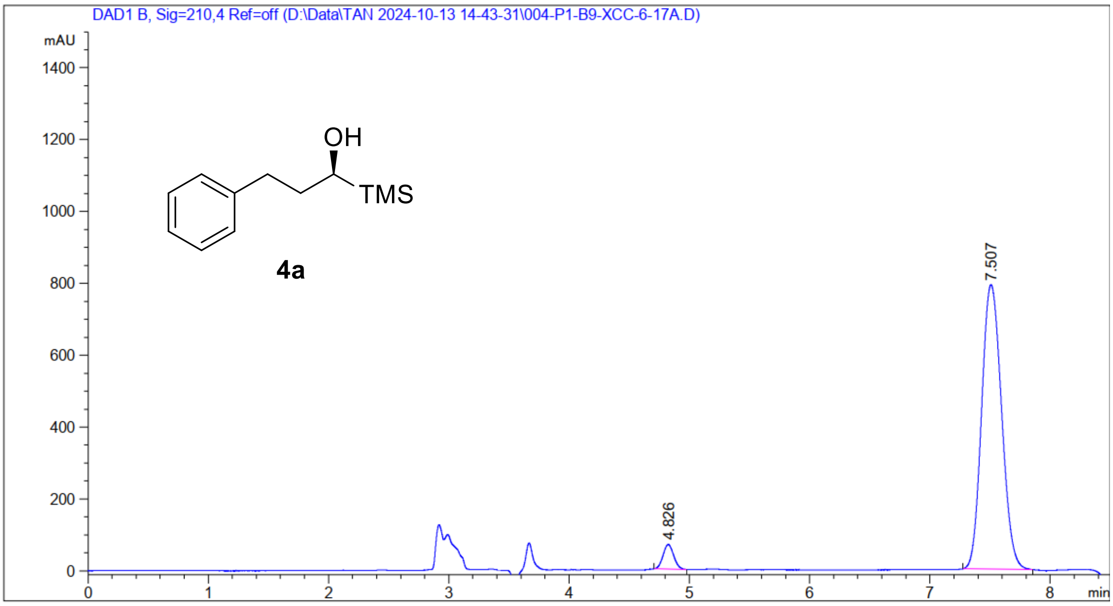

Peak #	RetTime [min]	Type	Width [min]	Area [mAU*s]	Height [mAU]	Area %
1	4.826	BB	0.0727	422.12897	68.66978	4.4364
2	7.507	BB	0.1348	9092.90820	791.35217	95.5636

(*S*)-3-Phenyl-1-(triethylsilyl)propan-1-ol (4b)

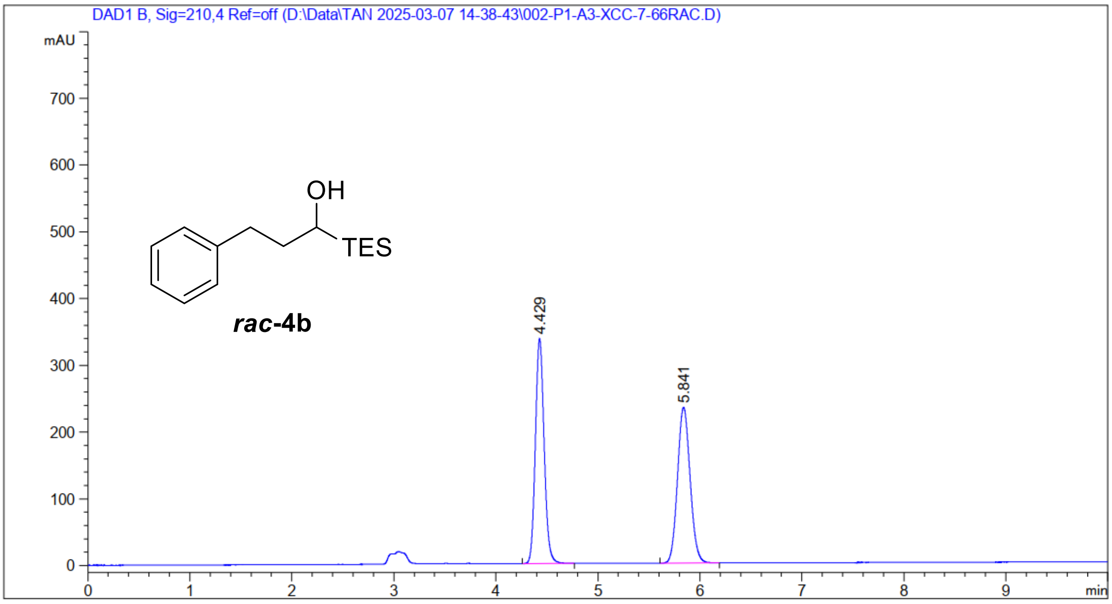

Peak #	RetTime [min]	Type	Width [min]	Area [mAU*s]	Height [mAU]	Area %
1	4.429	BB	0.0919	2005.39783	336.92072	49.9825
2	5.841	BB	0.1327	2006.79980	233.57422	50.0175

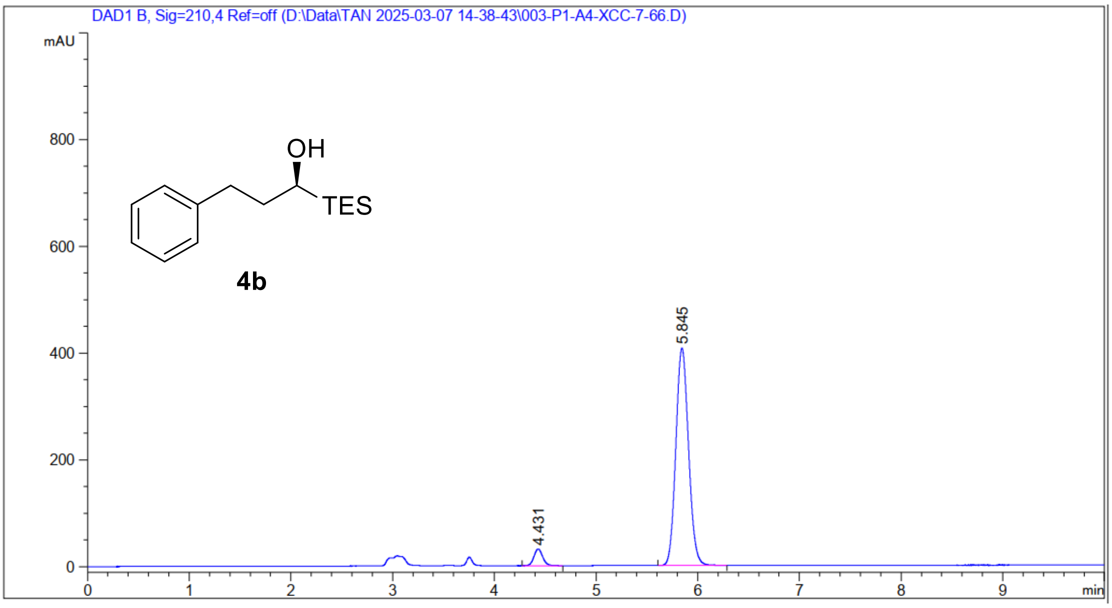

Peak #	RetTime [min]	Type	Width [min]	Area [mAU*s]	Height [mAU]	Area %
1	4.431	BB	0.0898	187.42412	31.31925	5.0502
2	5.845	BB	0.1345	3523.80713	407.22504	94.9498

(*S*)-1-(*tert*-Butyldimethylsilyl)-3-phenylpropan-1-ol (4c)

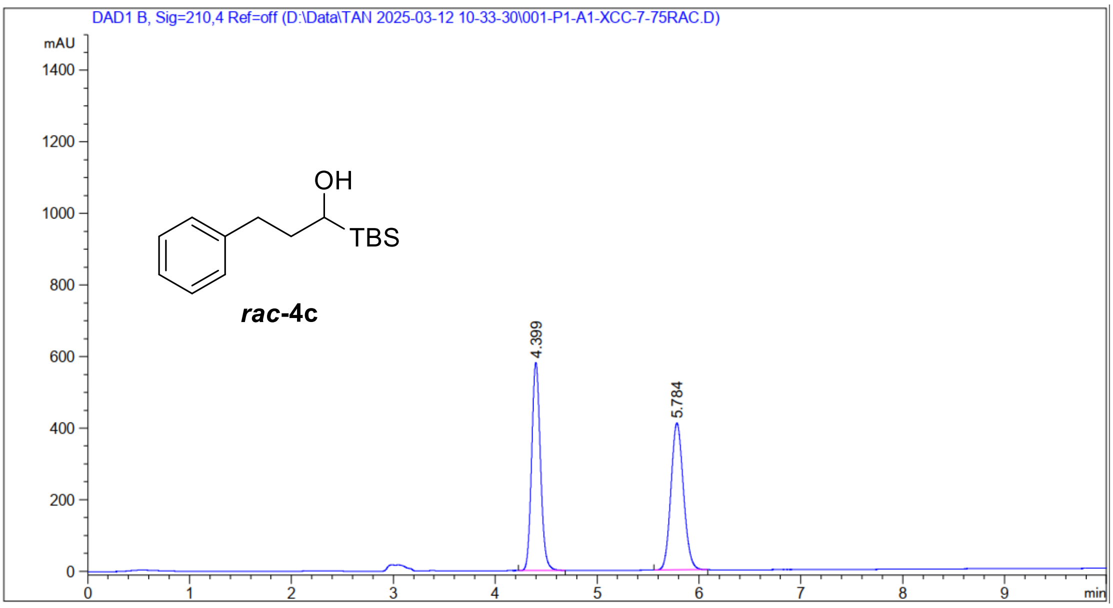

Peak #	RetTime [min]	Type	Width [min]	Area [mAU*s]	Height [mAU]	Area %
1	4.399	BV R	0.0922	3488.71680	581.63928	49.7714
2	5.784	VV R	0.1263	3520.76001	410.95364	50.2286

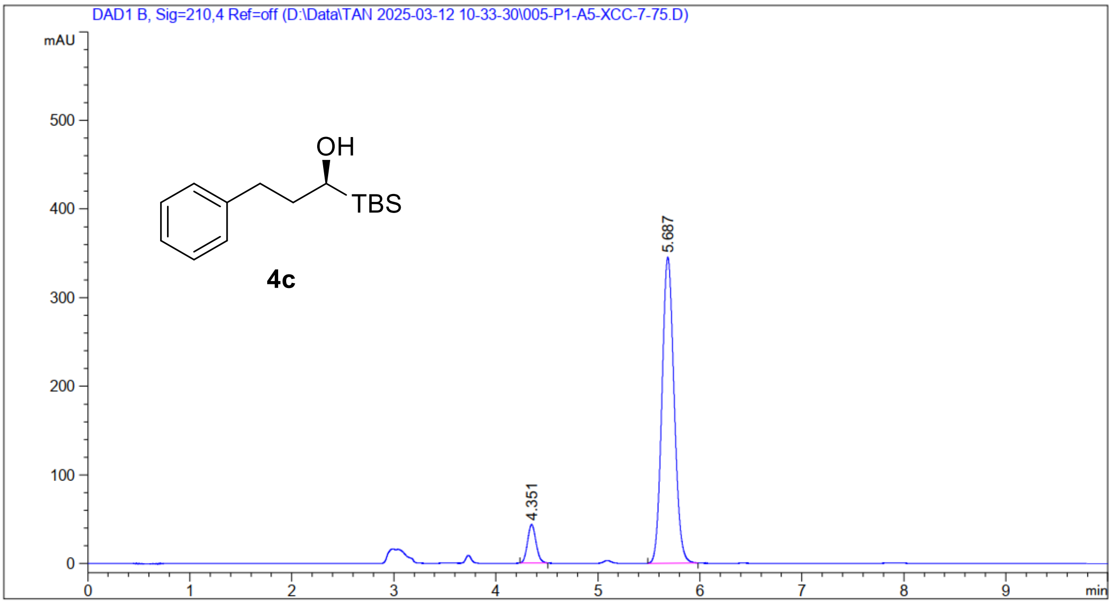

Peak #	RetTime [min]	Type	Width [min]	Area [mAU*s]	Height [mAU]	Area %
1	4.351	BB	0.0712	249.80141	43.46273	8.0402
2	5.687	BB	0.1170	2857.11328	345.09247	91.9598

(*S*)-3-(*p*-Tolyl)-1-(trimethylsilyl)propan-1-ol (4d)

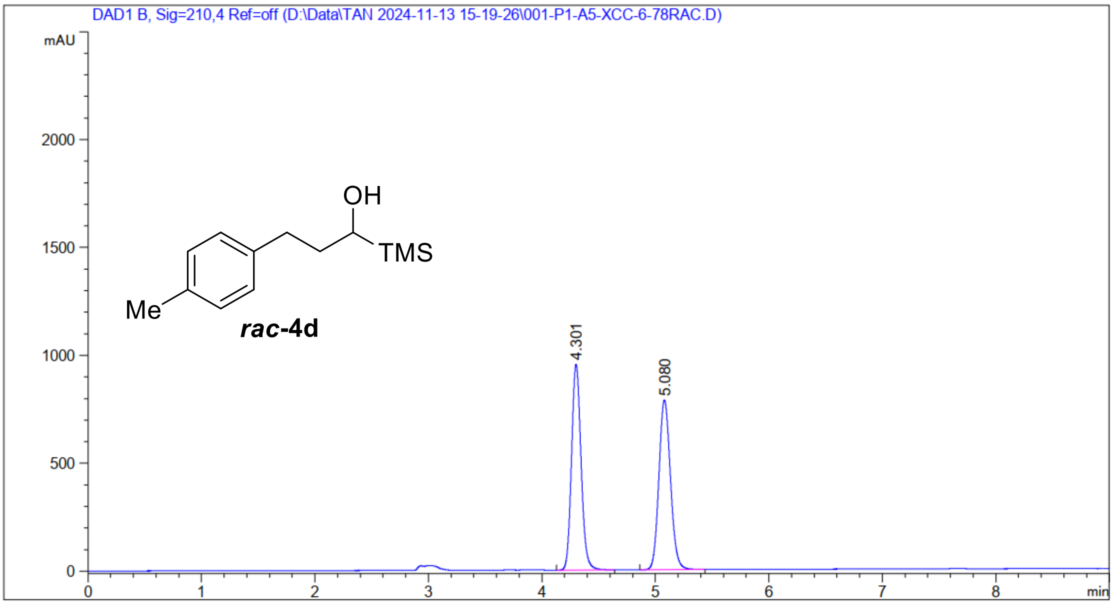

Peak #	RetTime [min]	Type	Width [min]	Area [mAU*s]	Height [mAU]	Area %
1	4.301	BB	0.0903	5566.83789	953.73187	50.0106
2	5.080	BB	0.1101	5564.48535	785.82770	49.9894

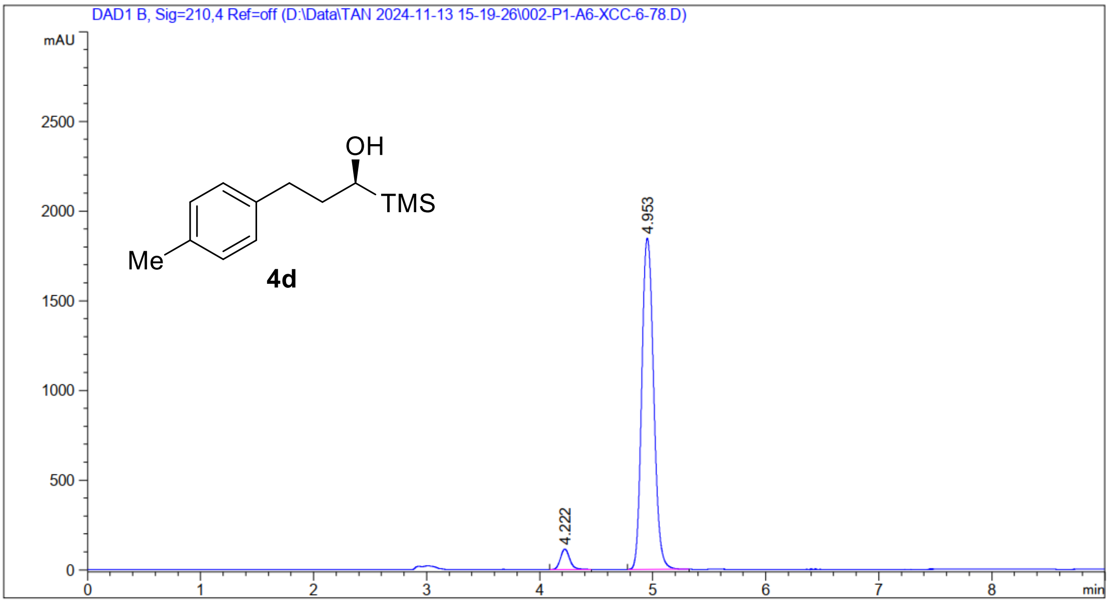

Peak #	RetTime [min]	Type	Width [min]	Area [mAU*s]	Height [mAU]	Area %
1	4.222	BB	0.0848	653.60559	114.30977	4.6153
2	4.953	BB	0.0880	1.35080e4	1845.63977	95.3847

(*S*)-4-(3-Hydroxy-3-(trimethylsilyl)propyl)phenol (4e)

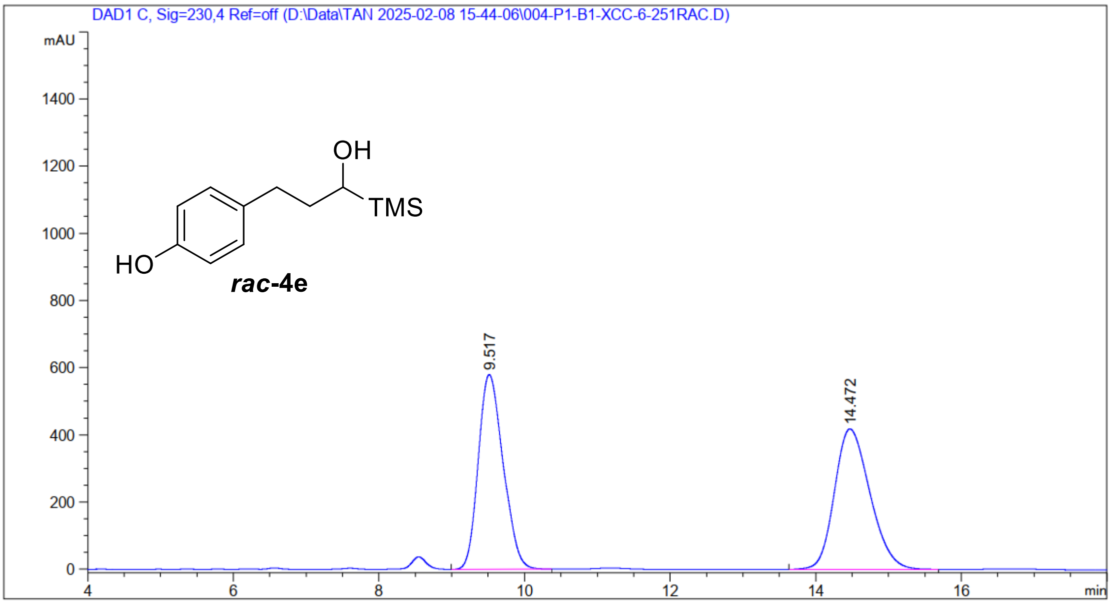

Peak #	RetTime [min]	Type	Width [min]	Area [mAU*s]	Height [mAU]	Area %
1	9.517	BB	0.2934	1.37621e4	580.00909	49.2220
2	14.472	BB	0.3973	1.41971e4	418.37814	50.7780

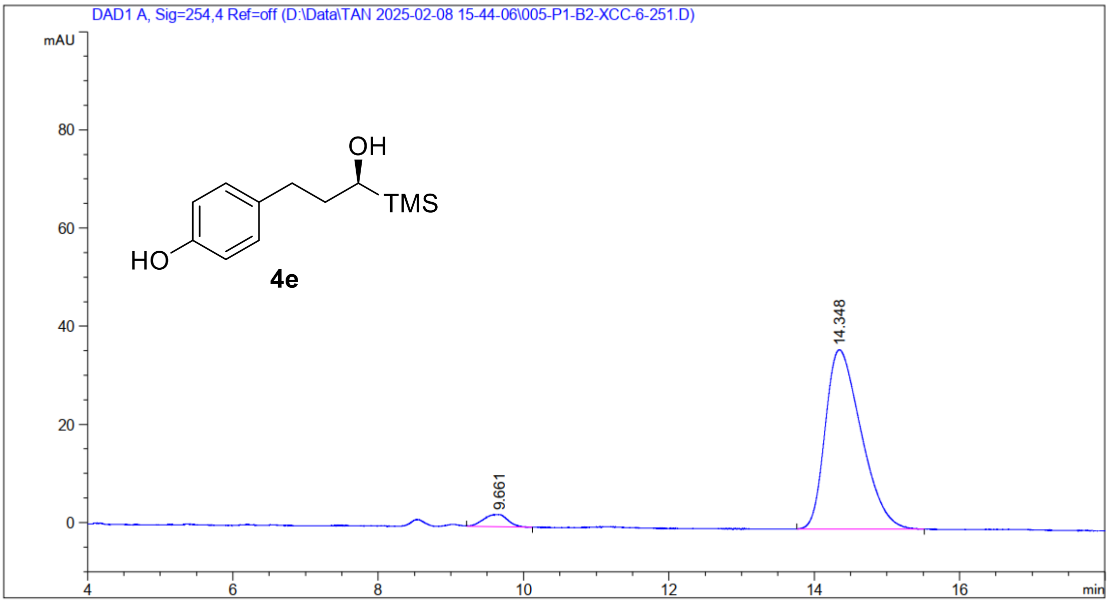

Peak #	RetTime [min]	Type	Width [min]	Area [mAU*s]	Height [mAU]	Area %
1	9.661	BB	0.2696	57.35513	2.51000	4.3783
2	14.348	BB	0.4109	1252.61853	36.54478	95.6217

(*S*)-3-(4-(Methoxymethoxy)phenyl)-1-(trimethylsilyl)propan-1-ol (4f)

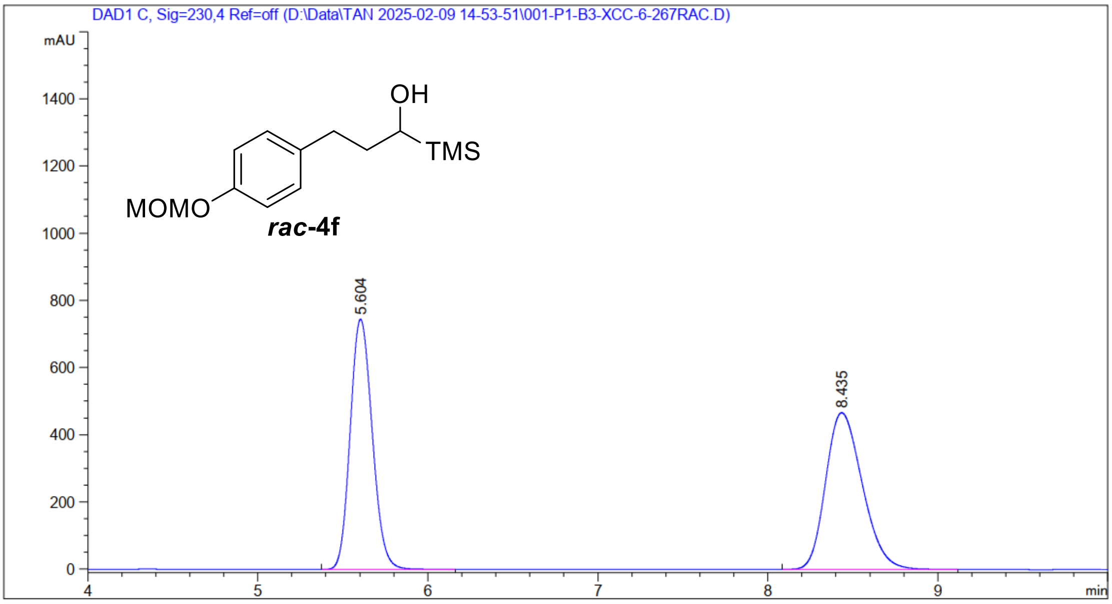

Peak #	RetTime [min]	Type	Width [min]	Area [mAU*s]	Height [mAU]	Area %
1	5.604	BB	0.1411	6714.74414	745.10626	48.9862
2	8.435	BB	0.2264	6992.68213	466.55502	51.0138

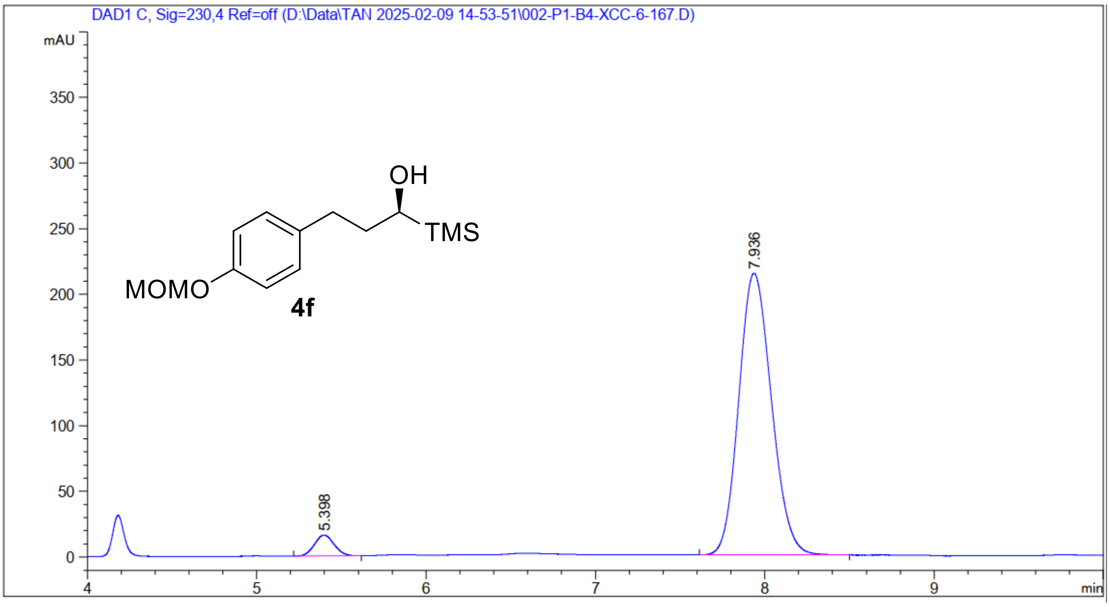

Peak #	RetTime [min]	Type	Width [min]	Area [mAU*s]	Height [mAU]	Area %
1	5.398	BB	0.1177	128.60040	15.92336	4.2866
2	7.936	BB	0.2057	2871.43140	214.43277	95.7134

(*S*)-3-(4-Fluorophenyl)-1-(trimethylsilyl)propan-1-ol (4g)

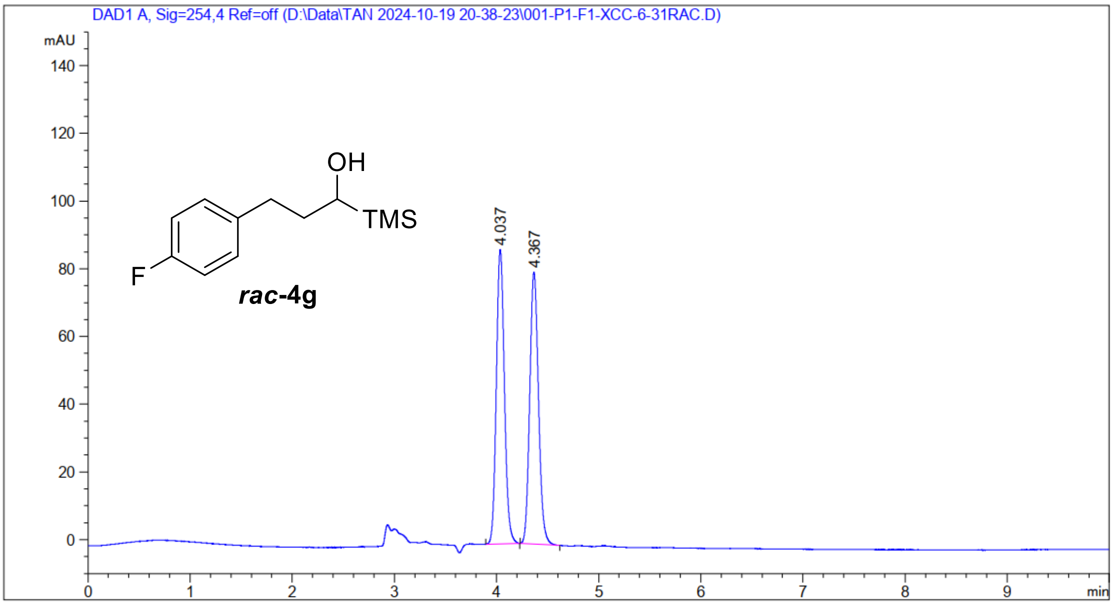

Peak #	RetTime [min]	Type	Width [min]	Area [mAU*s]	Height [mAU]	Area %
1	4.037	BB	0.0836	476.74393	86.97322	49.9508
2	4.367	BB	0.0904	477.68341	80.32137	50.0492

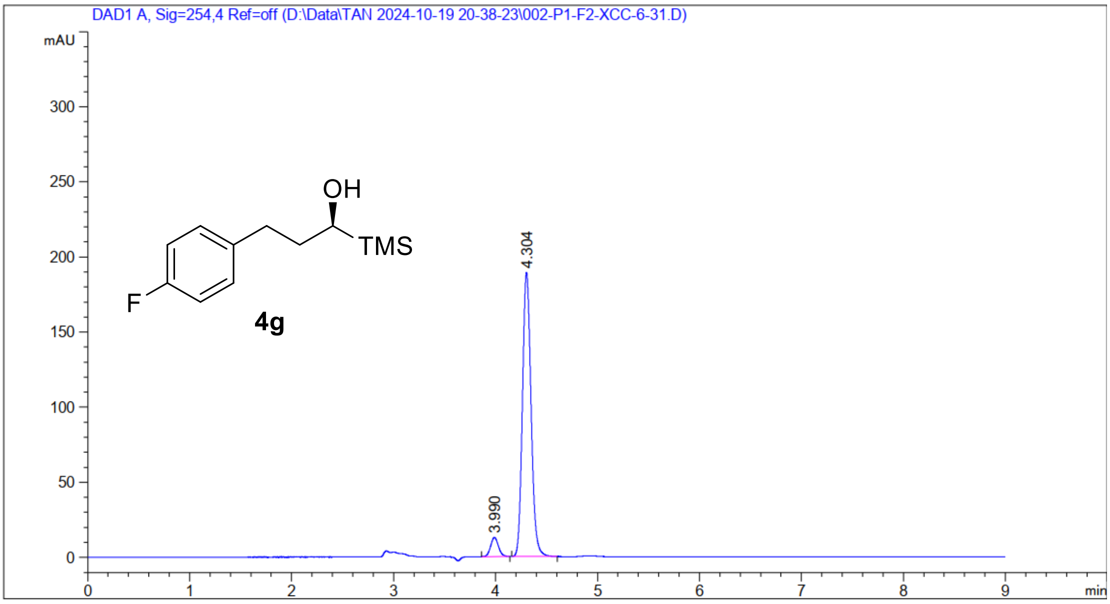

Peak #	RetTime [min]	Type	Width [min]	Area [mAU*s]	Height [mAU]	Area %
1	3.990	BB	0.0678	68.07247	12.74747	5.7874
2	4.304	BB	0.0903	1108.15259	189.22800	94.2126

(*S*)-3-(2,3-Dihydrobenzofuran-5-yl)-1-(trimethylsilyl)propan-1-ol (4h)

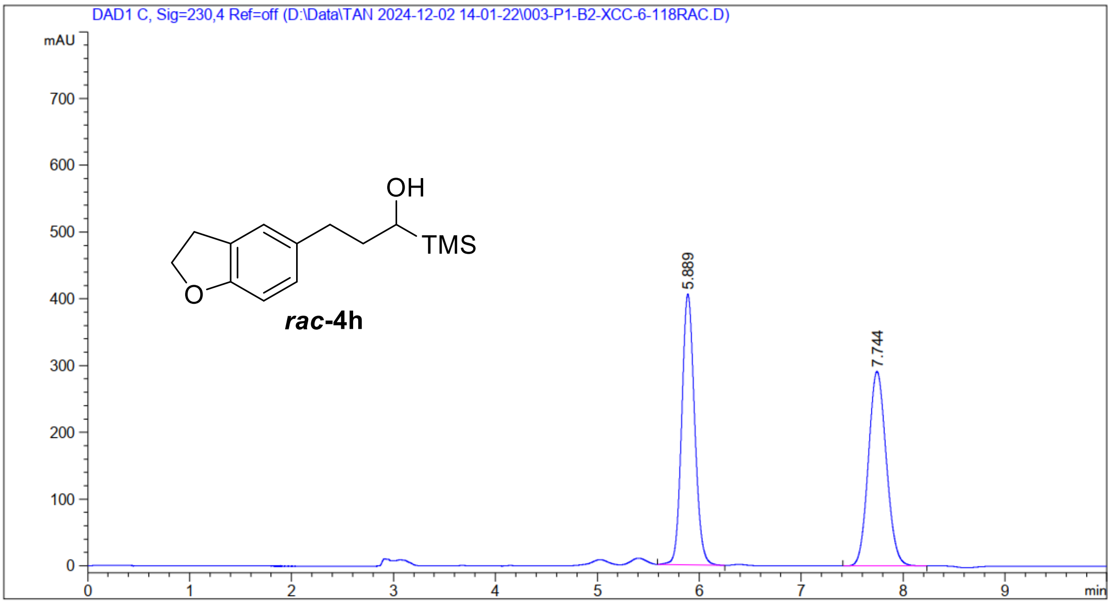

Peak #	RetTime [min]	Type	Width [min]	Area [mAU*s]	Height [mAU]	Area %
1	5.889	BB	0.1354	3559.11914	405.56720	50.0074
2	7.744	BB	0.1890	3558.07251	291.34955	49.9926

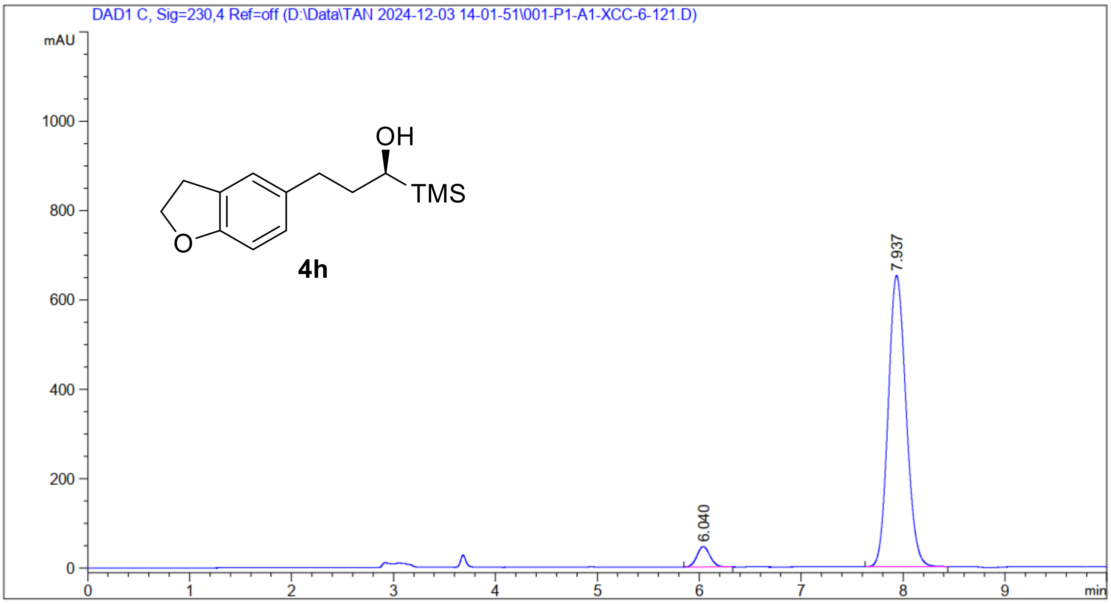

Peak #	RetTime [min]	Type	Width [min]	Area [mAU*s]	Height [mAU]	Area %
1	6.040	BB	0.1237	405.59677	45.85690	4.8746
2	7.937	BB	0.1852	7915.06885	651.72052	95.1254

(*S*)-3-(1,3-Dioxolan-2-yl)-1-(trimethylsilyl)propan-1-ol (4i)

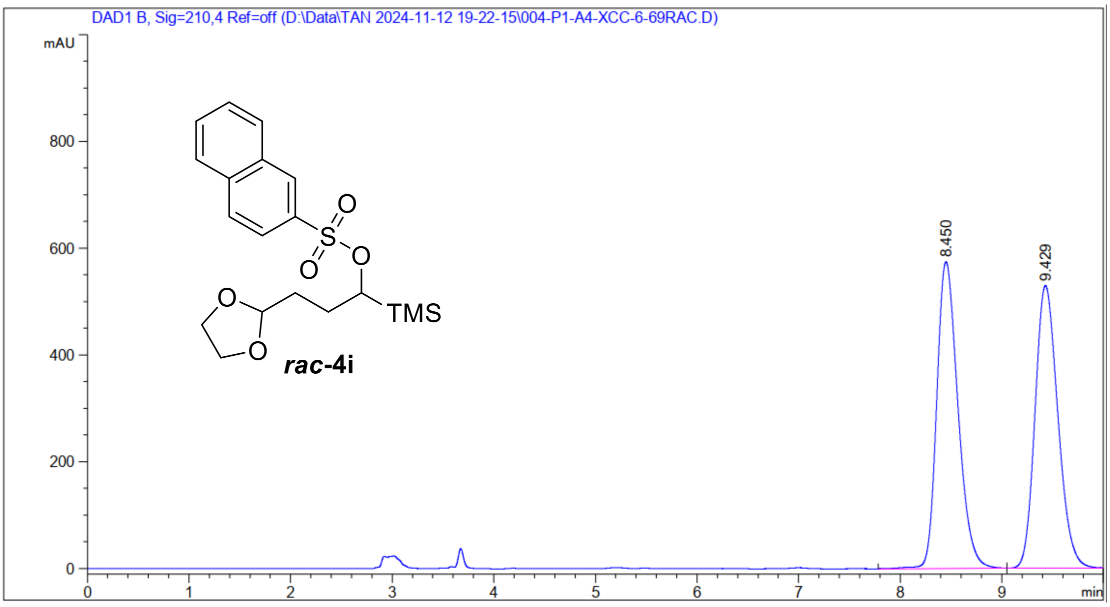

Peak #	RetTime [min]	Type	Width [min]	Area [mAU*s]	Height [mAU]	Area %
1	8.450	BB	0.2208	8311.30371	574.42340	50.1786
2	9.429	BBA	0.2345	8252.15527	528.91815	49.8214

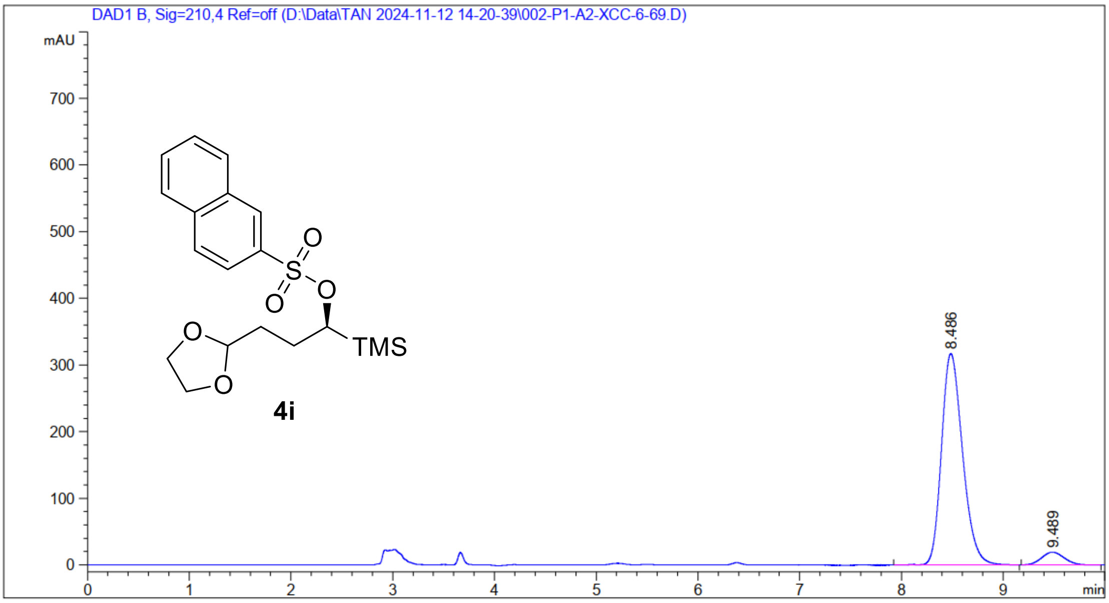

Peak #	RetTime [min]	Type	Width [min]	Area [mAU*s]	Height [mAU]	Area %
1	8.486	BB	0.2190	4566.26953	317.10995	93.9042
2	9.489	BV R	0.1848	296.42188	19.18578	6.0958

(*S*)-4-Phenyl-1-(trimethylsilyl)butan-1-ol (4j)

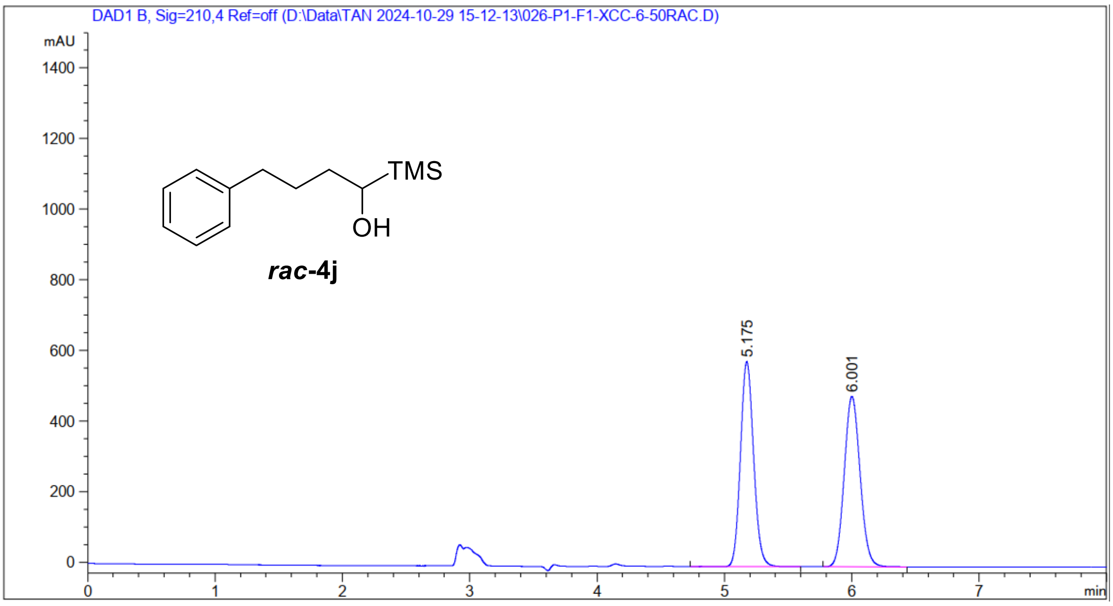

Peak #	RetTime [min]	Type	Width [min]	Area [mAU*s]	Height [mAU]	Area %
1	5.175	VB R	0.1114	4176.06250	580.48578	49.9699
2	6.001	BB	0.1320	4181.09717	482.83255	50.0301

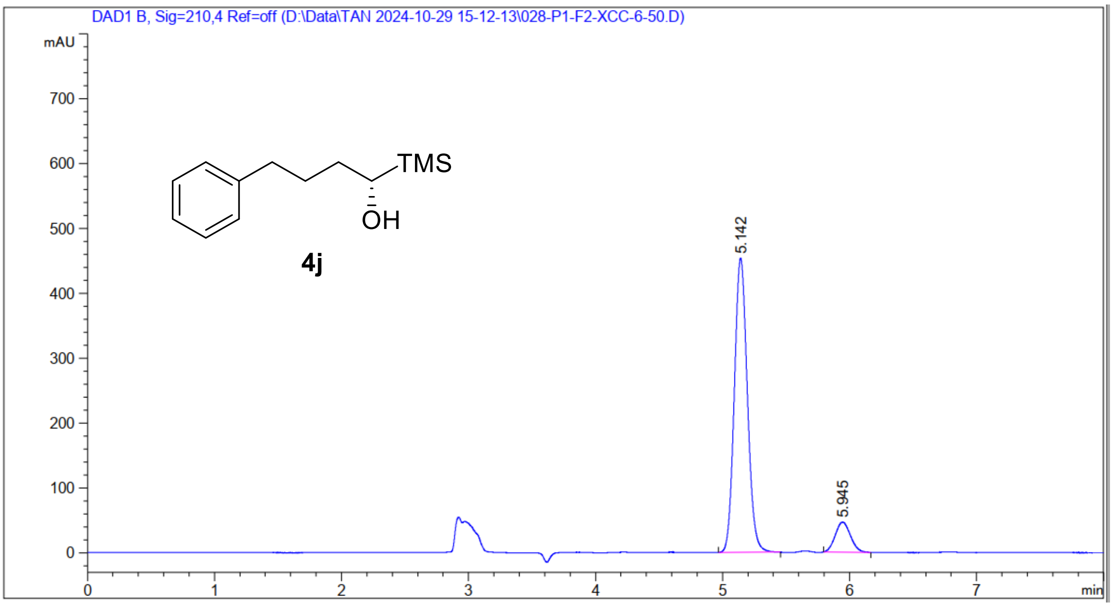

Peak #	RetTime [min]	Type	Width [min]	Area [mAU*s]	Height [mAU]	Area %
1	5.142	BB	0.1041	3231.14722	453.60529	89.3645
2	5.945	BB	0.0976	384.54843	46.33111	10.6355

(*S*)-1-(Trimethylsilyl)heptan-1-ol (4k)

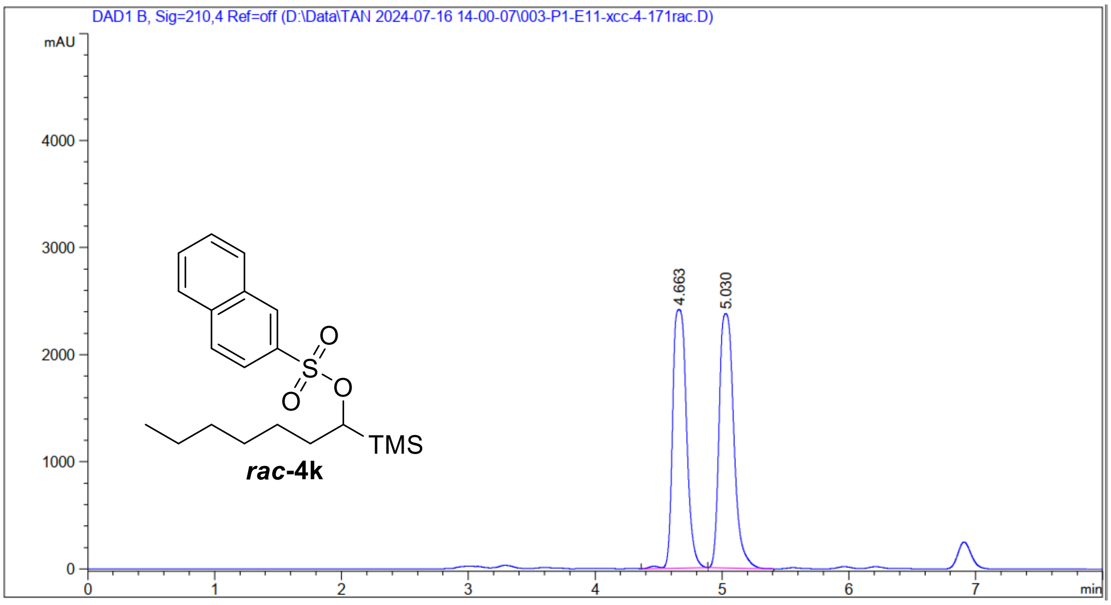

Peak #	RetTime [min]	Type	Width [min]	Area [mAU*s]	Height [mAU]	Area %
1	4.663	VB R	0.0890	1.82394e4	2417.43140	49.0114
2	5.030	BB	0.1121	1.89752e4	2376.69971	50.9886

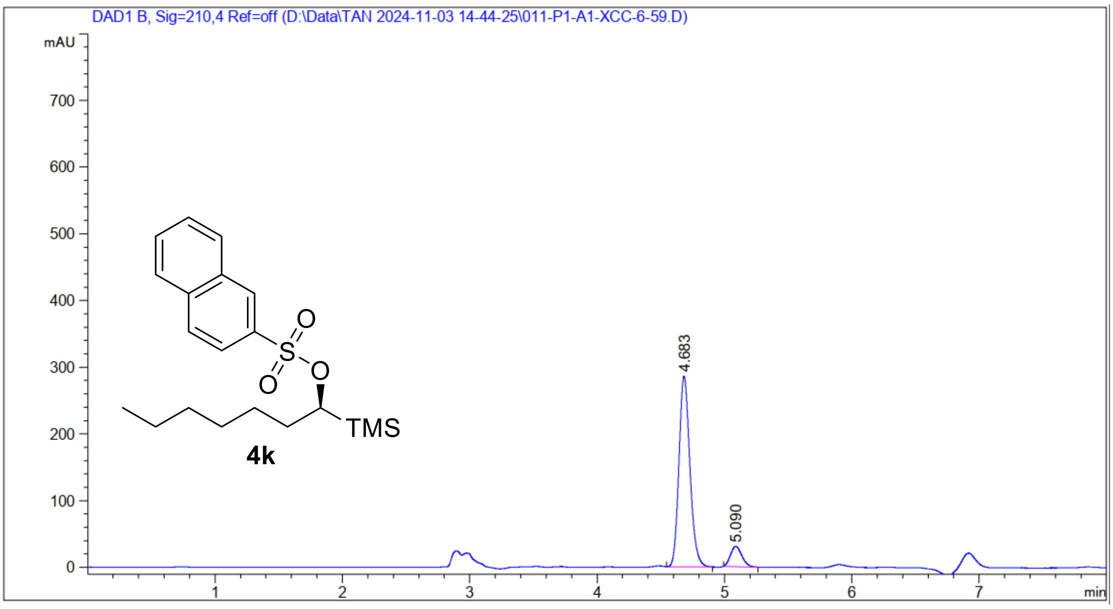

Peak #	RetTime [min]	Type	Width [min]	Area [mAU*s]	Height [mAU]	Area %
1	4.683	BB	0.0884	1706.37891	285.68268	90.1419
2	5.090	BB	0.0731	186.61363	30.28716	9.8581

(*S*)-6-Fluoro-1-(trimethylsilyl)hexan-1-ol (4l)

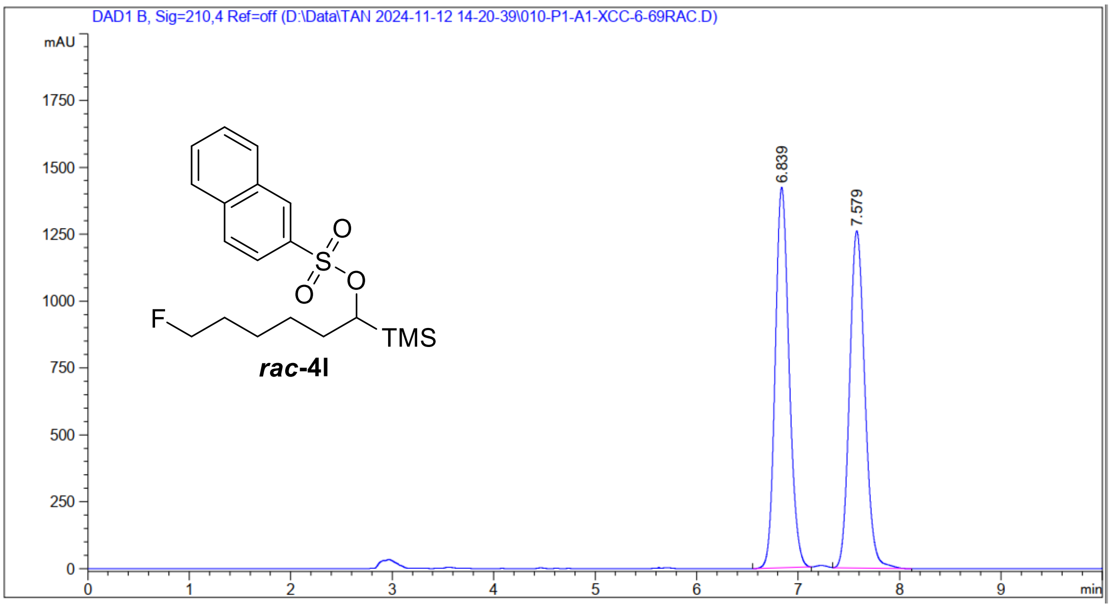

Peak #	RetTime [min]	Type	Width [min]	Area [mAU*s]	Height [mAU]	Area %
1	6.839	BB	0.1457	1.34936e4	1422.66858	50.5507
2	7.579	BB	0.1632	1.31996e4	1260.48608	49.4493

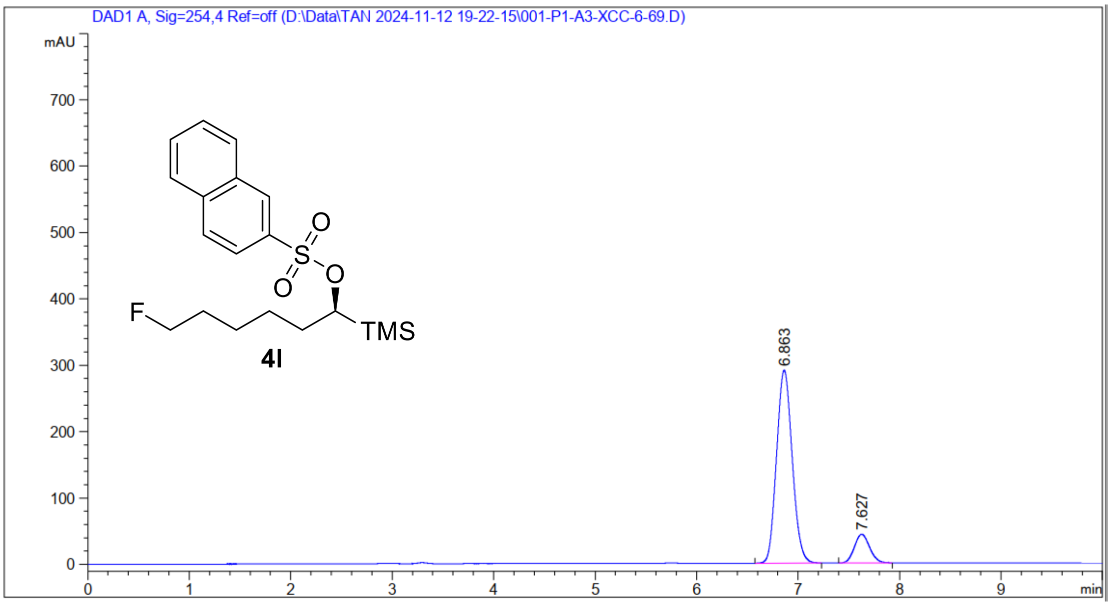

Peak #	RetTime [min]	Type	Width [min]	Area [mAU*s]	Height [mAU]	Area %
1	6.863	BB	0.1624	3112.29663	290.67694	87.3575
2	7.627	BB	0.1281	450.41406	43.20665	12.6425

(*S*,*E*)-3-Phenyl-1-(trimethylsilyl)prop-2-en-1-ol (4m)

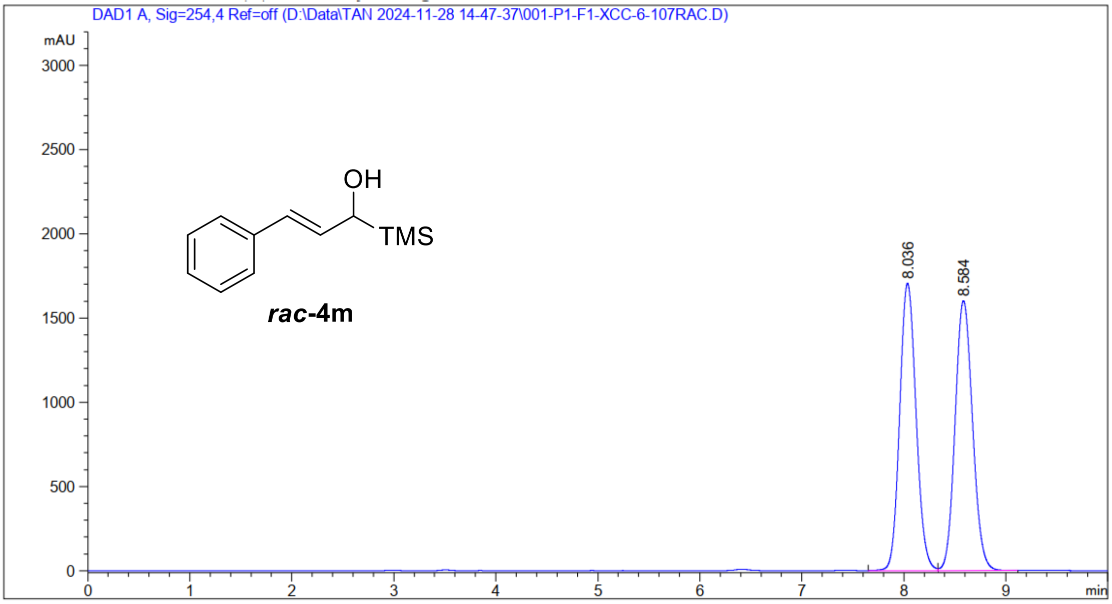

Peak #	RetTime [min]	Type	Width [min]	Area [mAU*s]	Height [mAU]	Area %
1	8.036	BV	0.1741	1.90192e4	1705.01892	49.9260
2	8.584	VB	0.1860	1.90756e4	1601.52979	50.0740

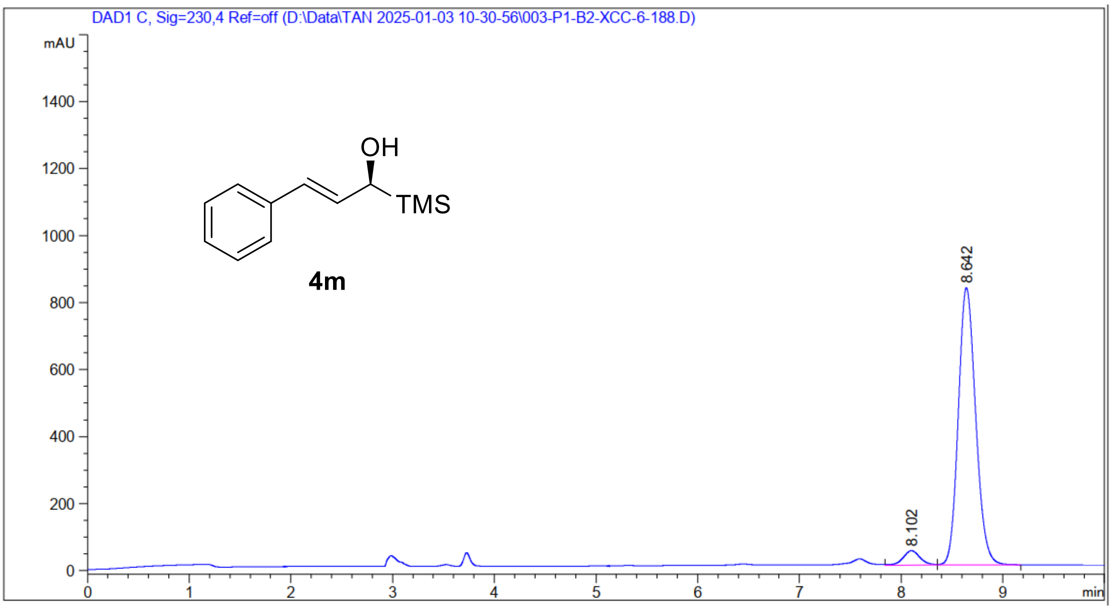

Peak #	RetTime [min]	Type	Width [min]	Area [mAU*s]	Height [mAU]	Area %
1	8.102	VV	0.1784	506.28687	43.29777	4.8950
2	8.642	VB	0.1846	9836.68945	827.84106	95.1050

(*S*,*E*)-2-Methyl-3-phenyl-1-(trimethylsilyl)prop-2-en-1-ol (4n)

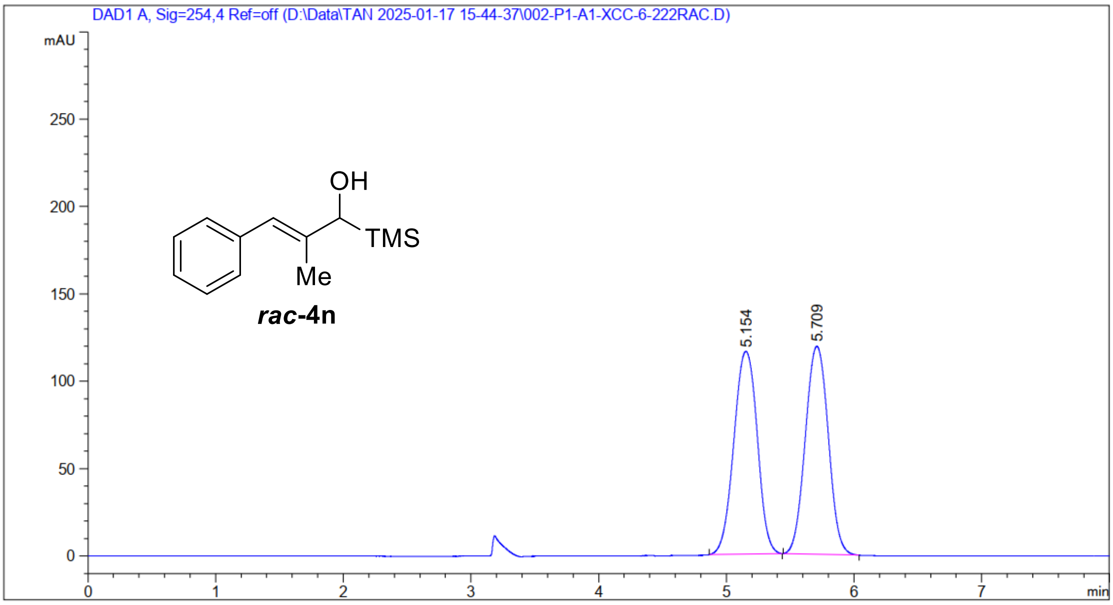

Peak #	RetTime [min]	Type	Width [min]	Area [mAU*s]	Height [mAU]	Area %
1	5.154	BB	0.1498	1476.16504	116.02759	49.8421
2	5.709	BB	0.1491	1485.51855	118.99217	50.1579

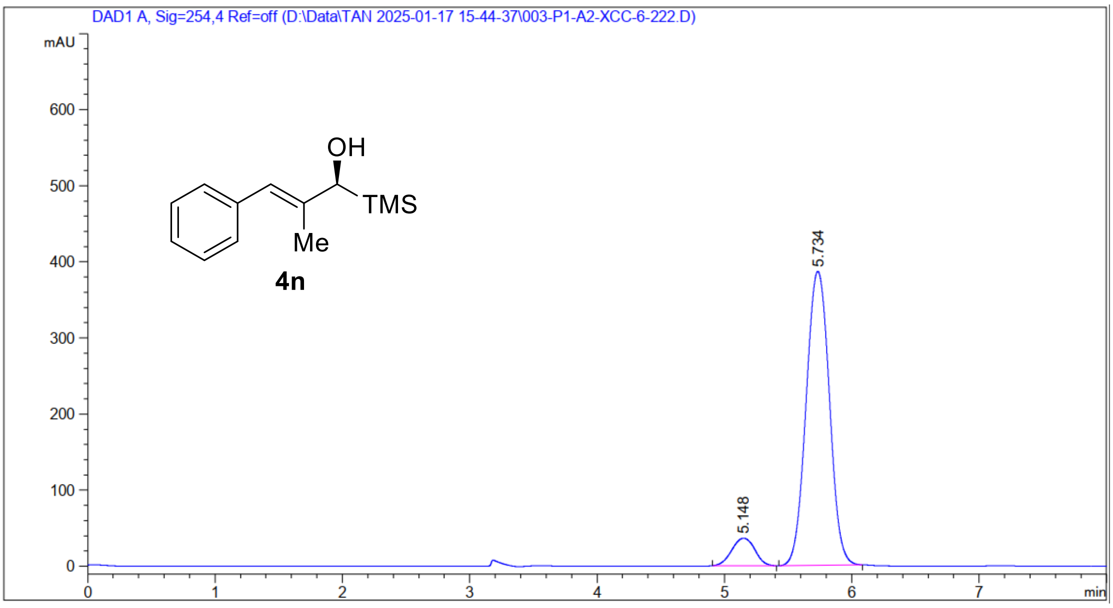

Peak #	RetTime [min]	Type	Width [min]	Area [mAU*s]	Height [mAU]	Area %
1	5.148	BB	0.1470	452.77850	36.05513	8.4729
2	5.734	BB	0.1634	4891.06592	386.25552	91.5271

(*S*)-2-(Phenyl(trimethylsilyl)methyl)isoindoline-1,3-dione (5a)

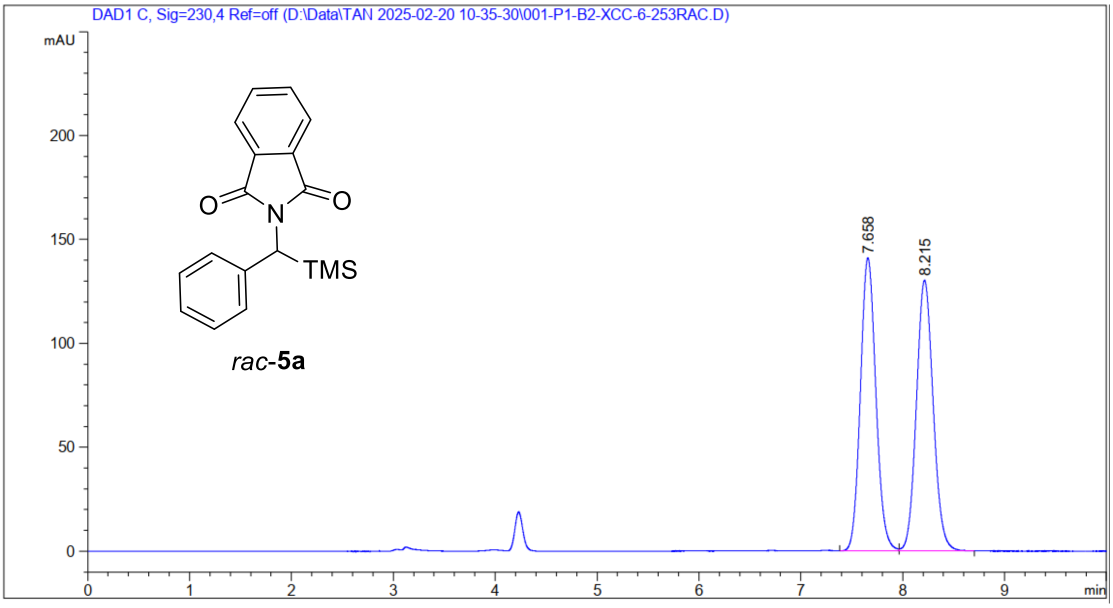

Peak #	RetTime [min]	Type	Width [min]	Area [mAU*s]	Height [mAU]	Area %
1	7.658	BV	0.1627	1489.66797	141.05608	49.9827
2	8.215	VB	0.1756	1490.69934	130.15657	50.0173

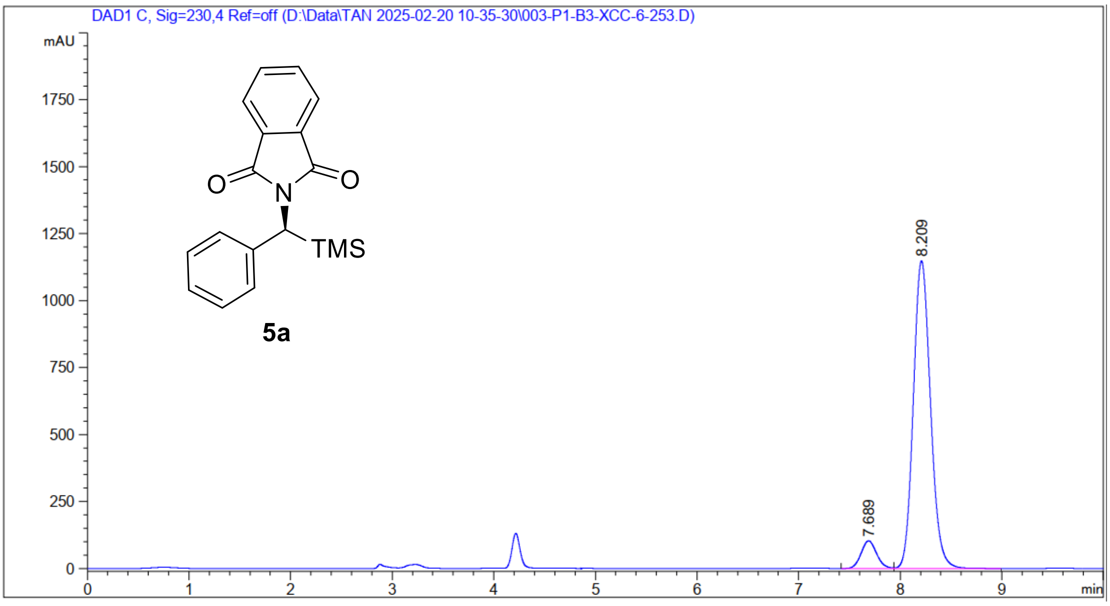

Peak #	RetTime [min]	Type	Width [min]	Area [mAU*s]	Height [mAU]	Area %
1	7.689	BV	0.1625	1089.18823	102.92950	7.4933
2	8.209	VB	0.1821	1.34462e4	1148.14001	92.5067

Asymmetric transfer hydrogenation

(*R*)-Phenyl(trimethylsilyl)methanol (2a)

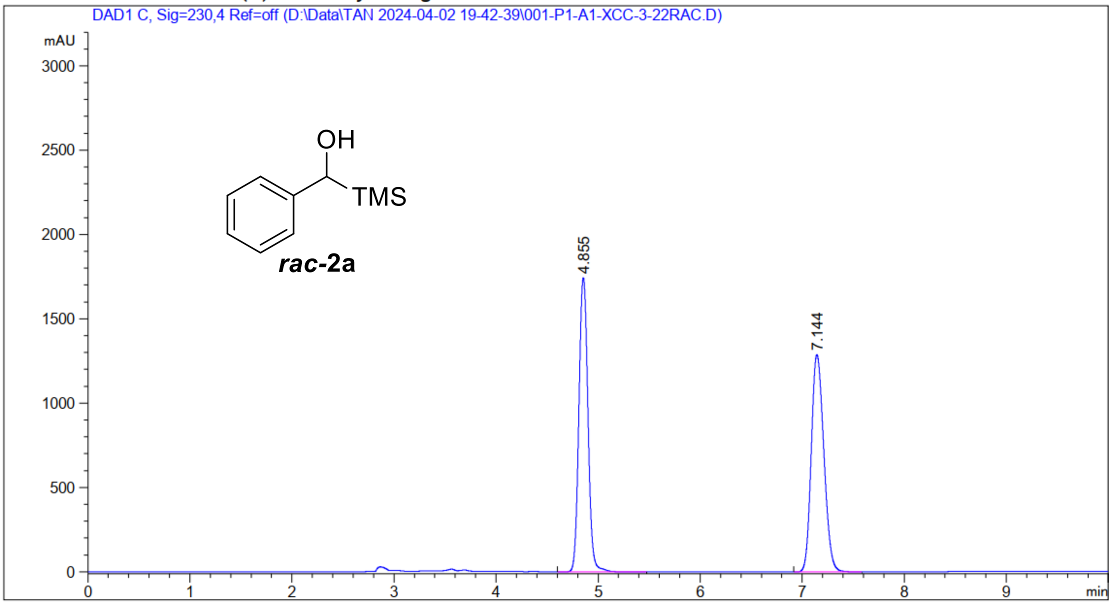

Peak #	RetTime [min]	Type	Width [min]	Area [mAU*s]	Height [mAU]	Area %
1	4.855	BB	0.0967	1.07248e4	1744.13904	49.3407
2	7.144	BB	0.1341	1.10114e4	1289.18213	50.6593

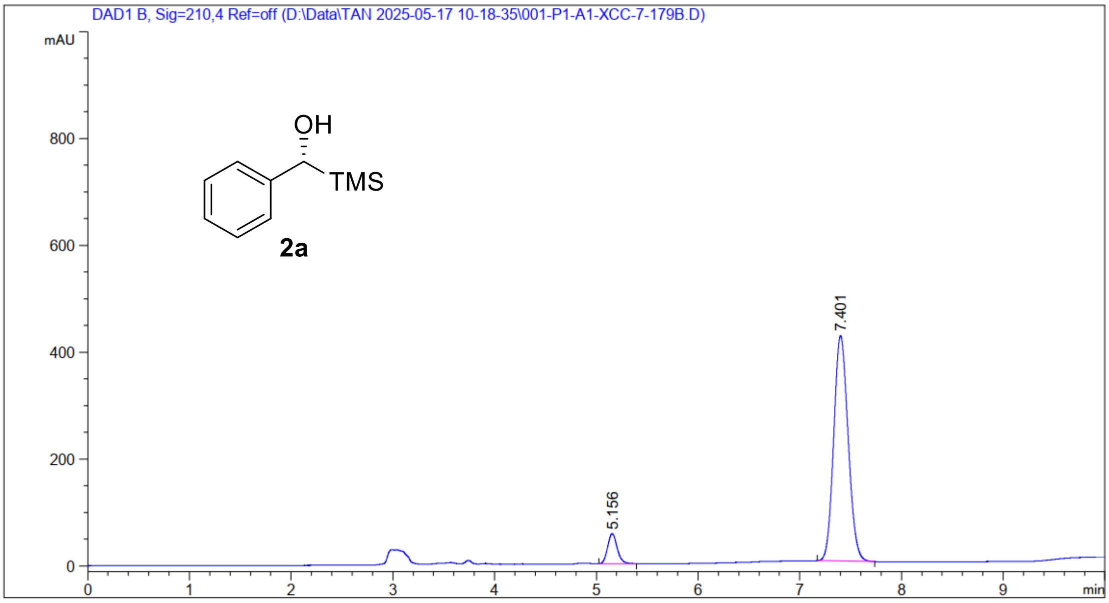

Peak #	RetTime [min]	Type	Width [min]	Area [mAU*s]	Height [mAU]	Area %
1	5.156	BV R	0.0810	368.79822	56.20835	8.1600
2	7.401	BB	0.1271	4150.77051	421.58954	91.8400

(*R*)-*p*-Tolyl(trimethylsilyl)methanol (2d)

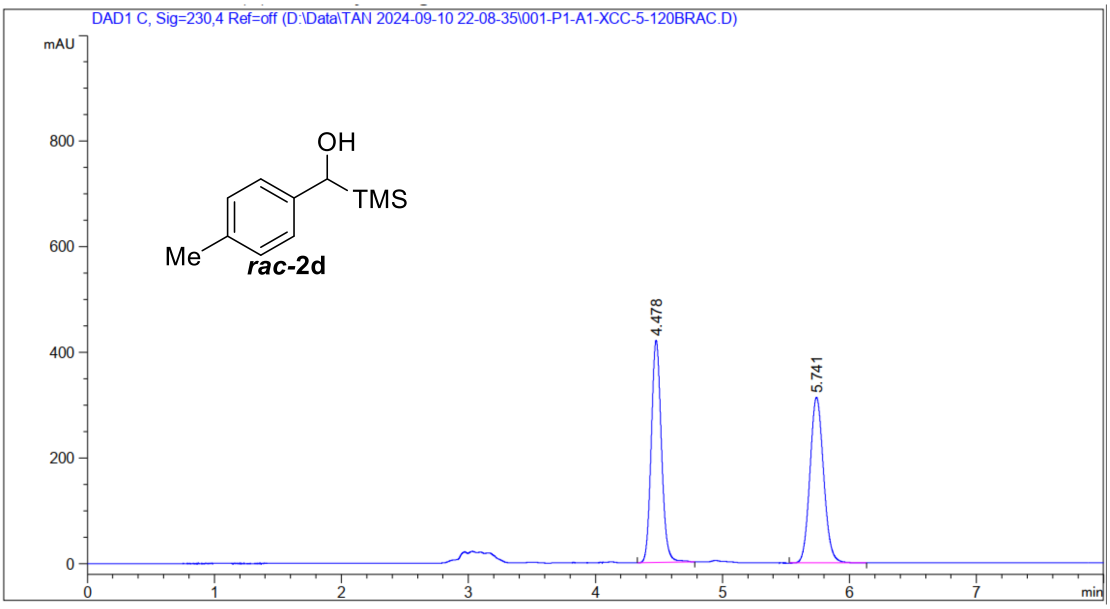

Peak #	RetTime [min]	Type	Width [min]	Area [mAU*s]	Height [mAU]	Area %
1	4.478	BB	0.0865	2344.88110	420.91937	49.9523
2	5.741	BB	0.1169	2349.35522	313.66473	50.0477

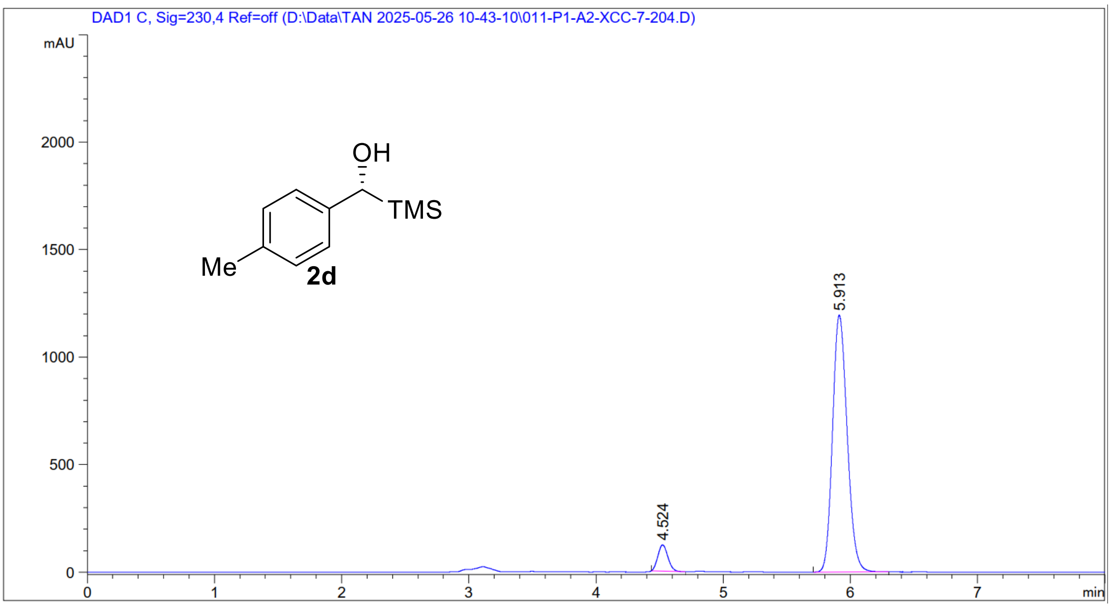

Peak #	RetTime [min]	Type	Width [min]	Area [mAU*s]	Height [mAU]	Area %
1	4.524	BB	0.0776	662.16321	121.44176	6.4843
2	5.913	BB	0.1220	9549.70508	1194.77234	93.5157

(*R*)-*m*-Tolyl(trimethylsilyl)methanol (2f)

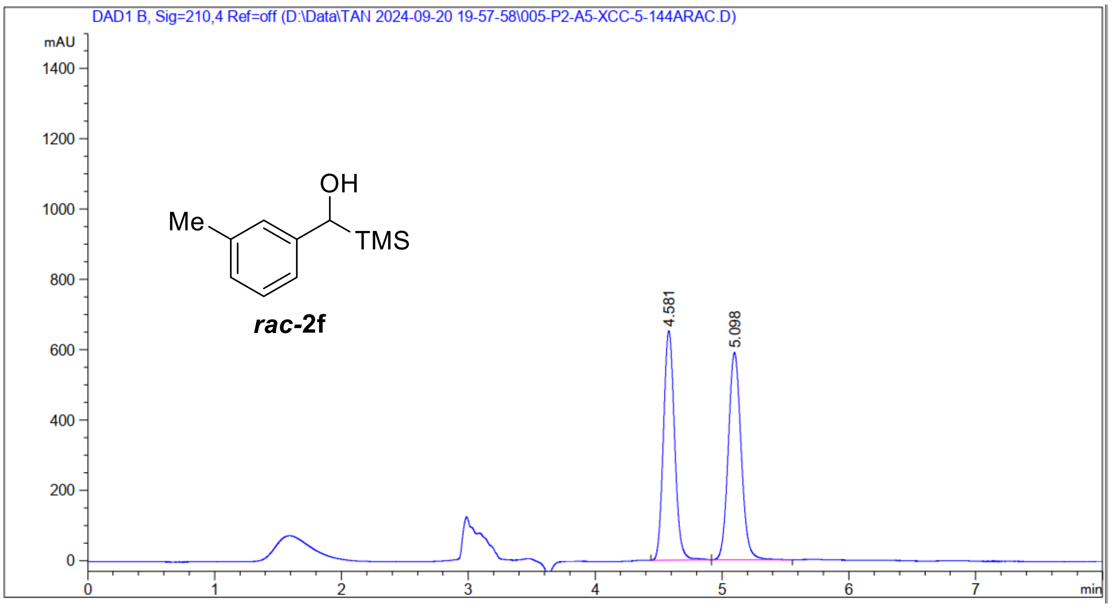

Peak #	RetTime [min]	Type	Width [min]	Area [mAU*s]	Height [mAU]	Area %
1	4.581	BB	0.0923	3987.31519	652.72144	48.9608
2	5.098	BV R	0.1088	4156.58203	589.30377	51.0392

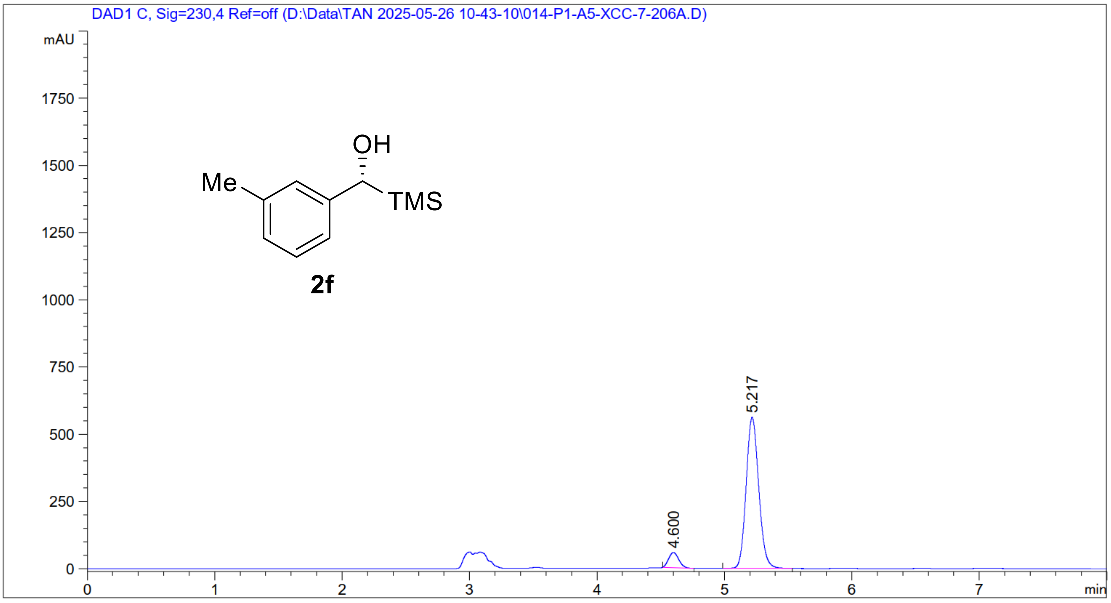

Peak #	RetTime [min]	Type	Width [min]	Area [mAU*s]	Height [mAU]	Area %
1	4.600	BB	0.0767	310.54611	56.62999	7.4383
2	5.217	BB	0.1055	3864.41943	562.06458	92.5617

(*R*)-(4-Methoxyphenyl)(trimethylsilyl)methanol (2h)

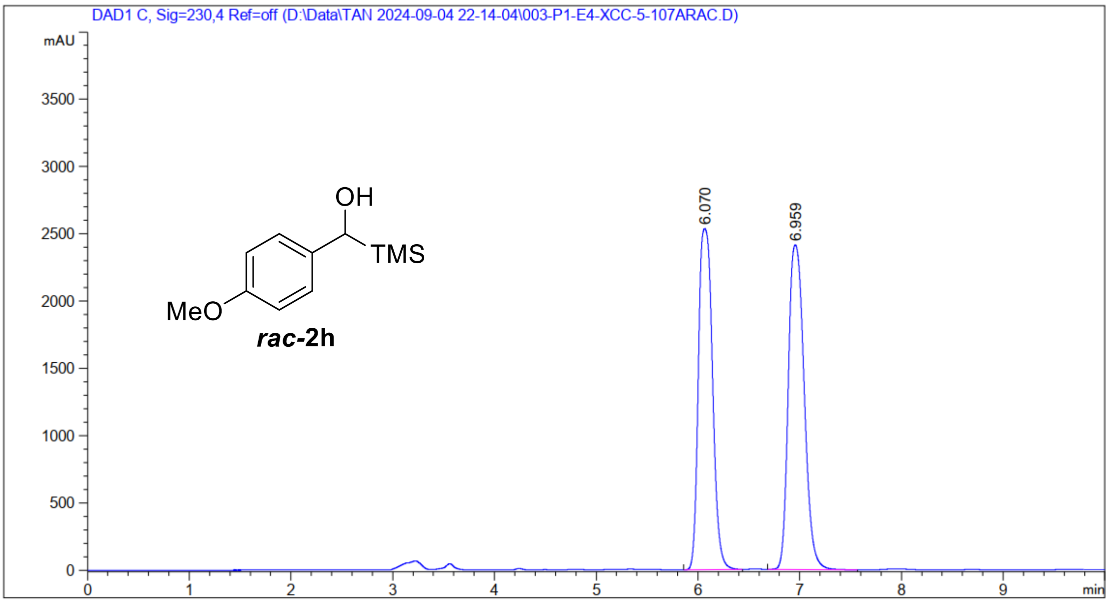

Peak #	RetTime [min]	Type	Width [min]	Area [mAU*s]	Height [mAU]	Area %
1	6.070	BB	0.1152	2.42925e4	2536.16602	48.2766
2	6.959	BB	0.1483	2.60269e4	2413.53735	51.7234

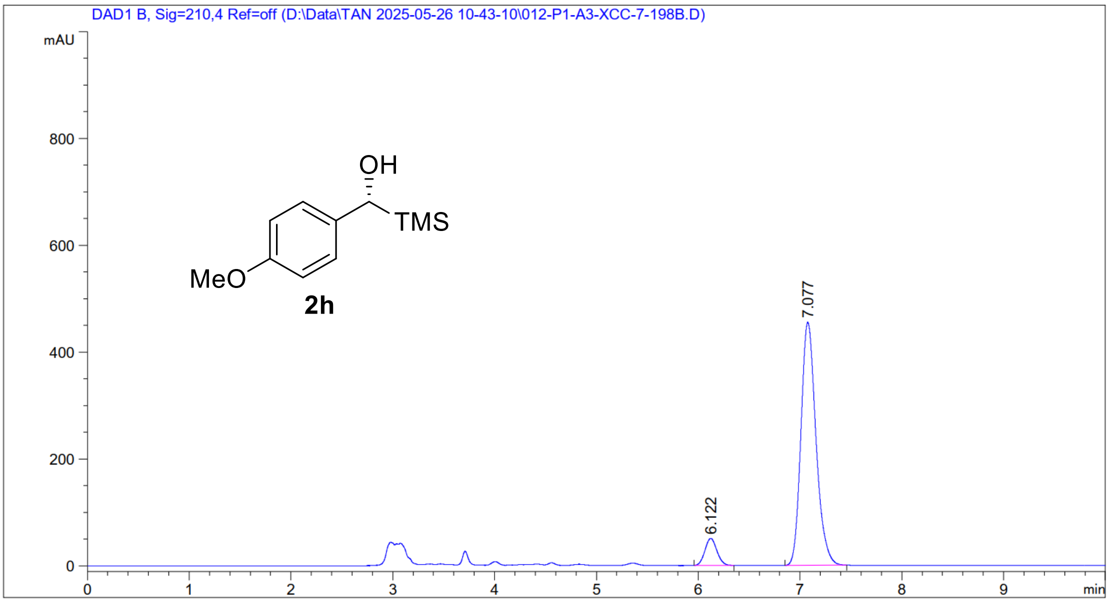

Peak #	RetTime [min]	Type	Width [min]	Area [mAU*s]	Height [mAU]	Area %
1	6.122	BB	0.0961	413.39233	50.65737	8.1396
2	7.077	BB	0.1270	4665.37549	455.00049	91.8604

(*R*)-(3-Methoxyphenyl)(trimethylsilyl)methanol (2i)

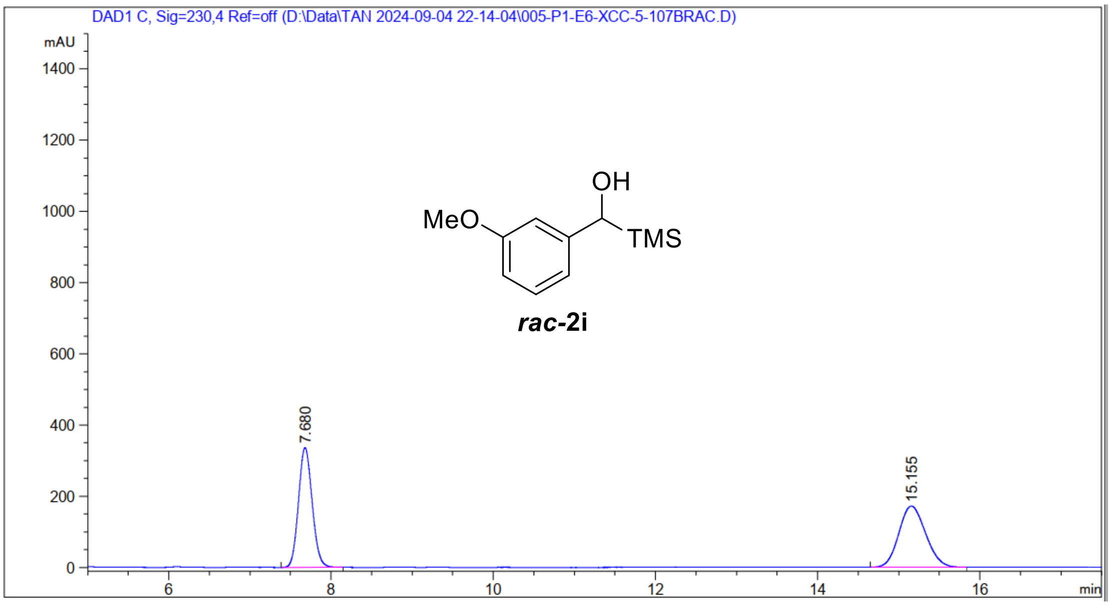

Peak #	RetTime [min]	Type	Width [min]	Area [mAU*s]	Height [mAU]	Area %
1	7.680	BB	0.1803	3935.83472	336.21399	49.9855
2	15.155	BB	0.2688	3938.12183	171.73387	50.0145

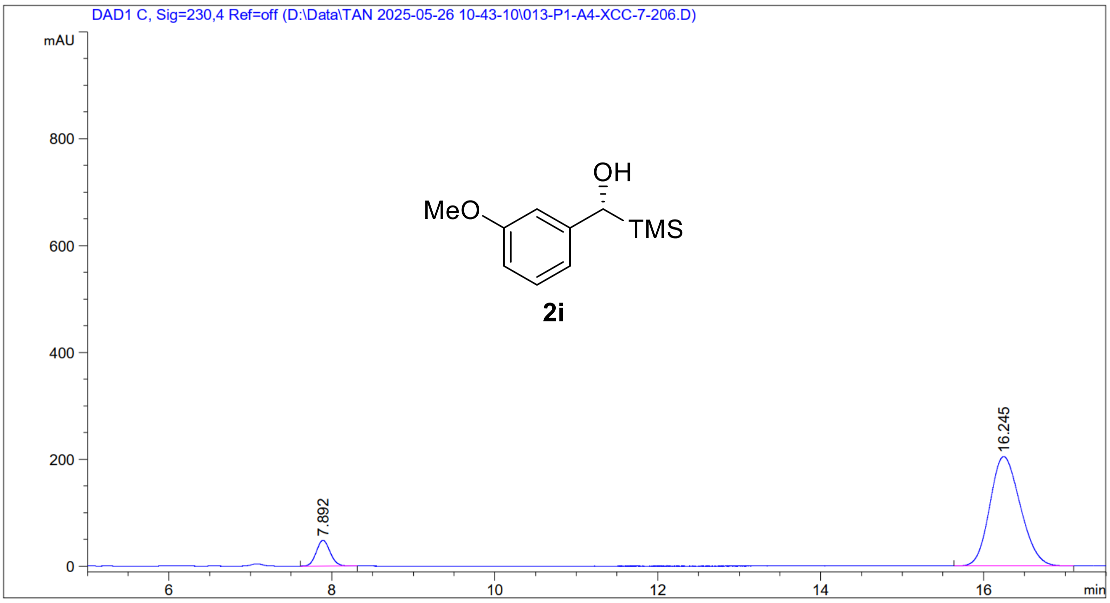

Peak #	RetTime [min]	Type	Width [min]	Area [mAU*s]	Height [mAU]	Area %
1	7.892	BB	0.1758	572.59442	48.28869	9.7137
2	16.245	BB	0.3372	5322.10938	204.79288	90.2863

(*R*)-(3,4-Dimethylphenyl)(trimethylsilyl)methanol (2m)

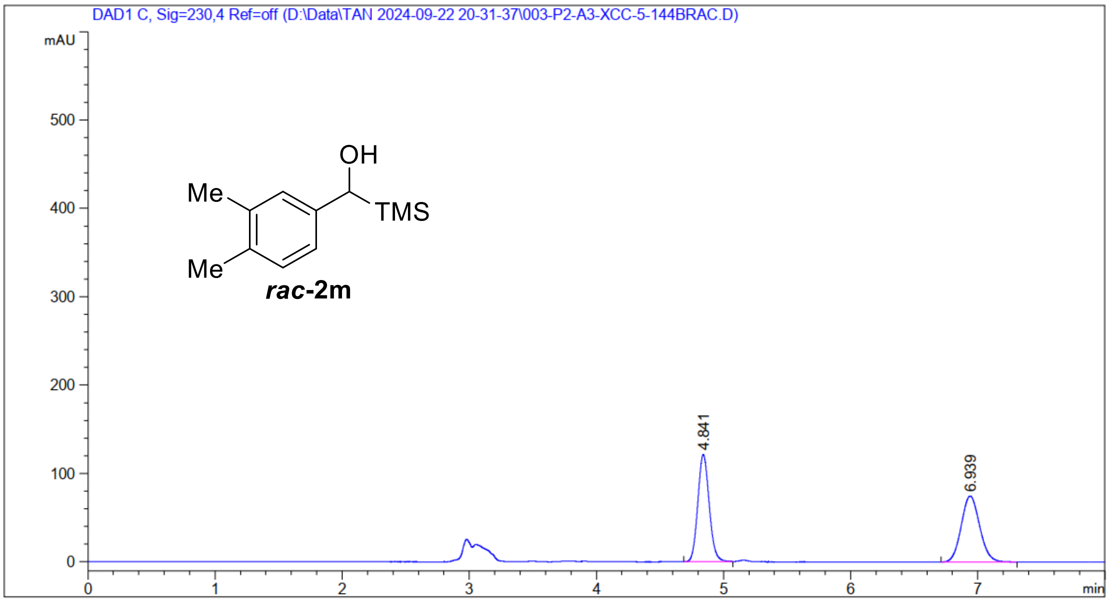

Peak #	RetTime [min]	Type	Width [min]	Area [mAU*s]	Height [mAU]	Area %
1	4.841	BB	0.0954	753.11749	121.28550	50.0111
2	6.939	BB	0.1416	752.78247	74.68680	49.9889

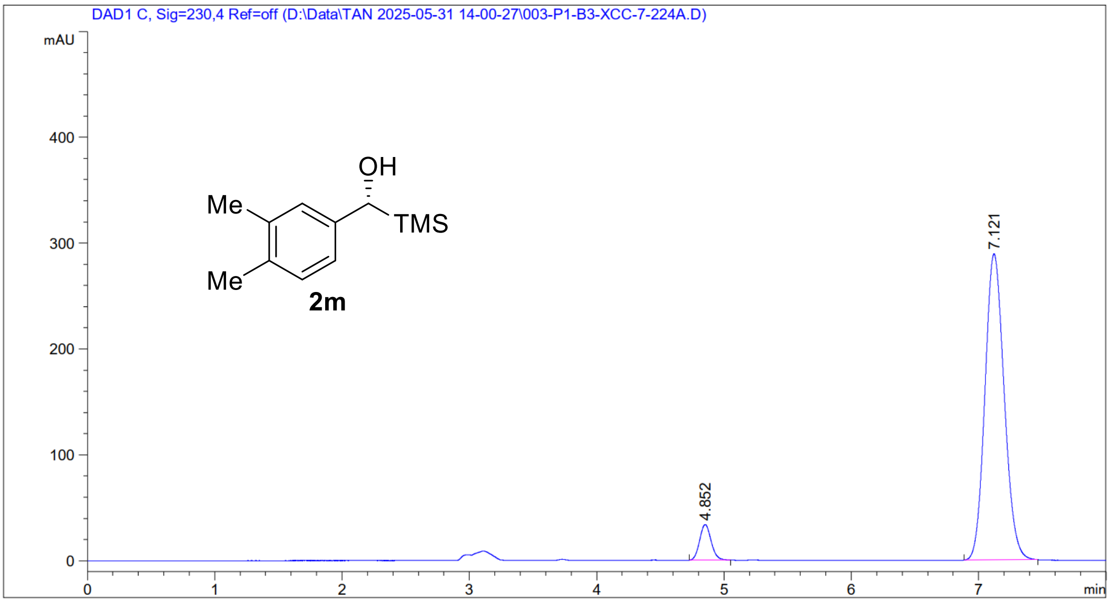

Peak #	RetTime [min]	Type	Width [min]	Area [mAU*s]	Height [mAU]	Area %
1	4.852	BB	0.0745	209.99826	33.56850	6.4737
2	7.121	BB	0.1436	3033.87622	289.08490	93.5263

(*R*)-(3,5-Dimethylphenyl)(trimethylsilyl)methanol (2n)

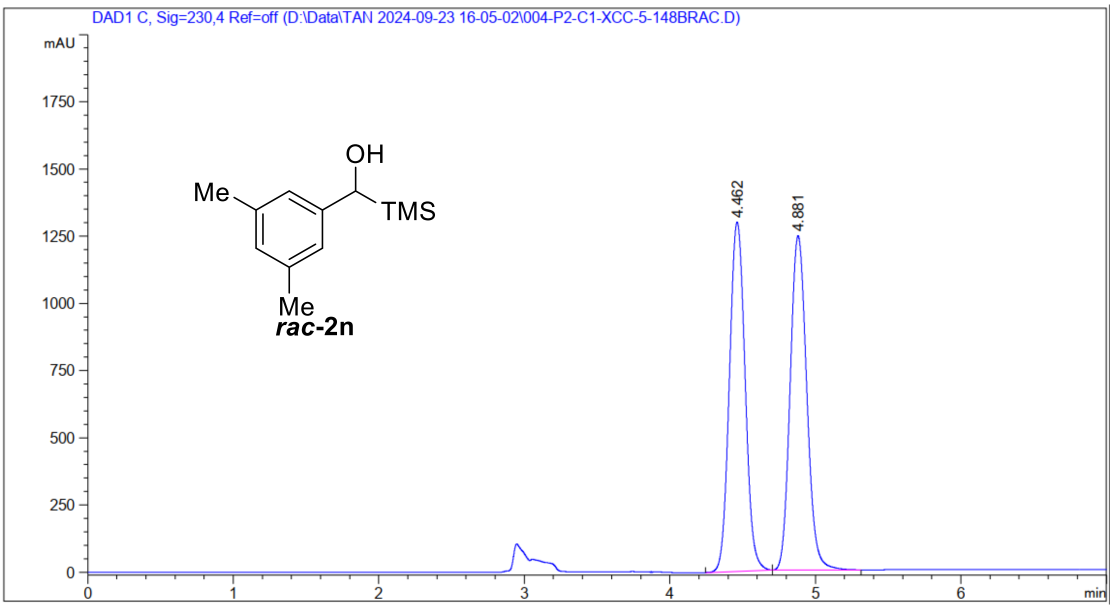

Peak #	RetTime [min]	Type	Width [min]	Area [mAU*s]	Height [mAU]	Area %
1	4.462	BB	0.1180	9740.73047	1300.08984	49.7254
2	4.881	BB	0.1228	9848.30273	1243.63184	50.2746

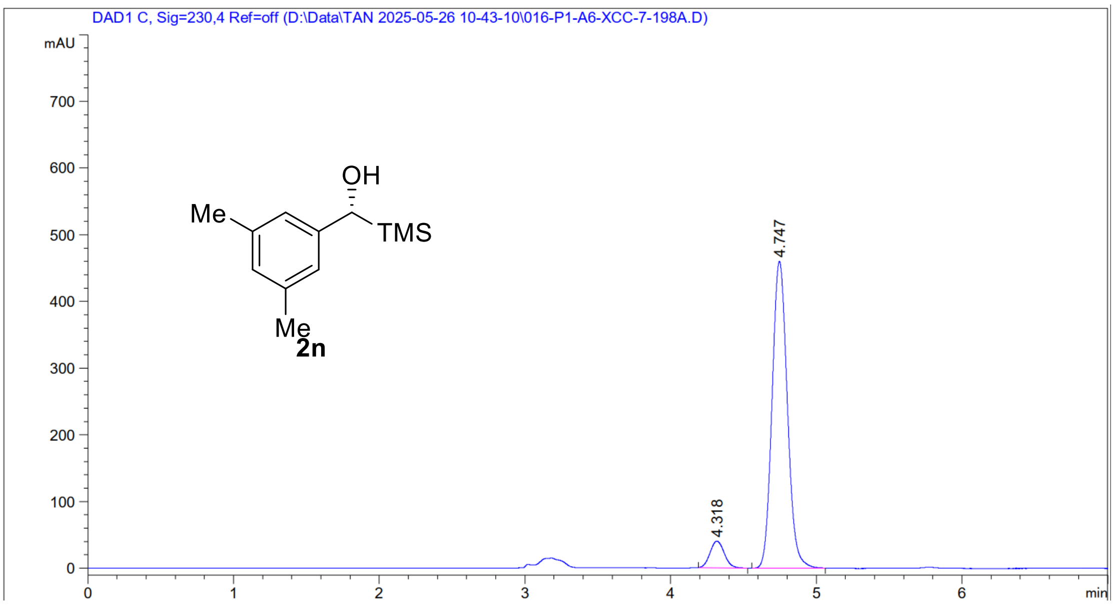

Peak #	RetTime [min]	Type	Width [min]	Area [mAU*s]	Height [mAU]	Area %
1	4.318	BB	0.0801	269.43109	39.91011	7.4594
2	4.747	BB	0.1122	3342.53223	460.21054	92.5406

(*R*)-(2,3-Dihydrobenzofuran-5-yl)(trimethylsilyl)methanol (2q)

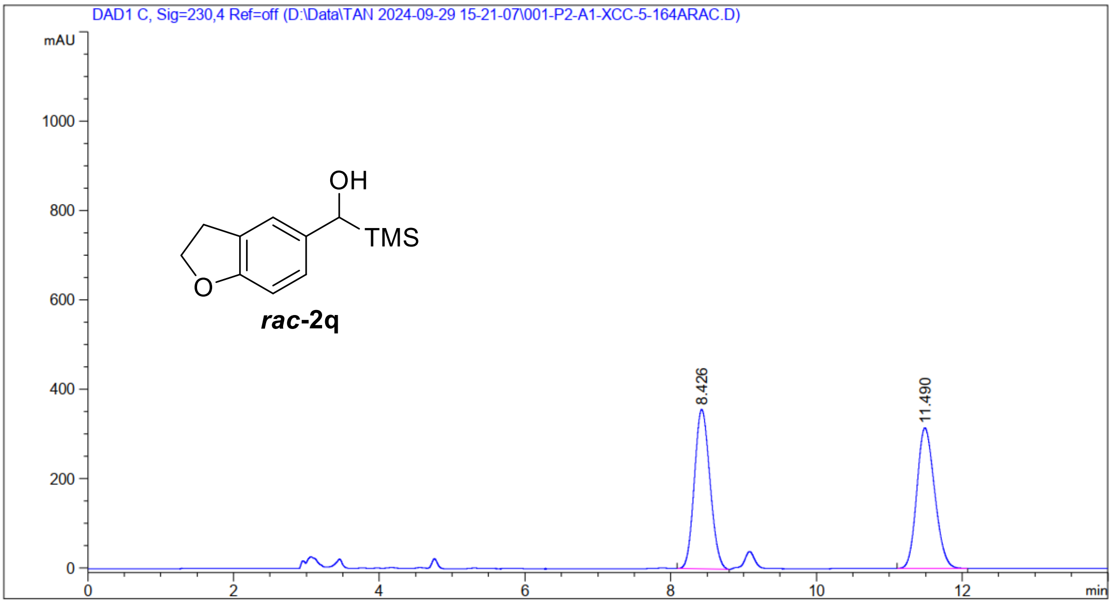

Peak #	RetTime [min]	Type	Width [min]	Area [mAU*s]	Height [mAU]	Area %
1	8.426	BB	0.2116	5328.07080	357.31839	49.9027
2	11.490	BB	0.2359	5348.84570	314.15219	50.0973

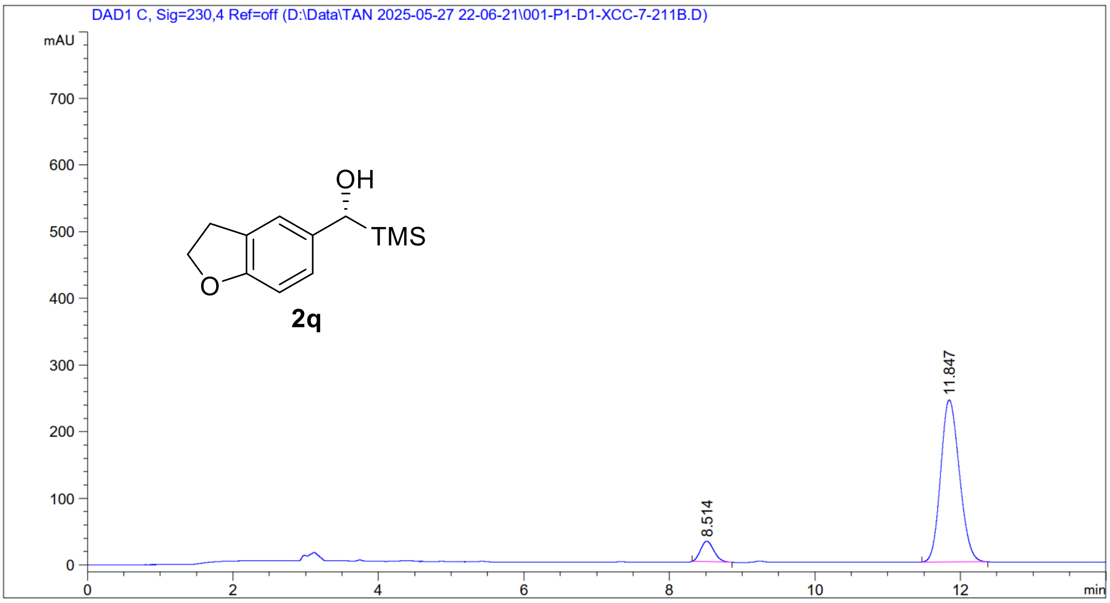

Peak #	RetTime [min]	Type	Width [min]	Area [mAU*s]	Height [mAU]	Area %
1	8.514	BB	0.1497	392.77789	30.74581	8.2931
2	11.847	BB	0.2105	4343.40332	243.21109	91.7069

(*R*)-(3-Fluorophenyl)(trimethylsilyl)methanol (2t)

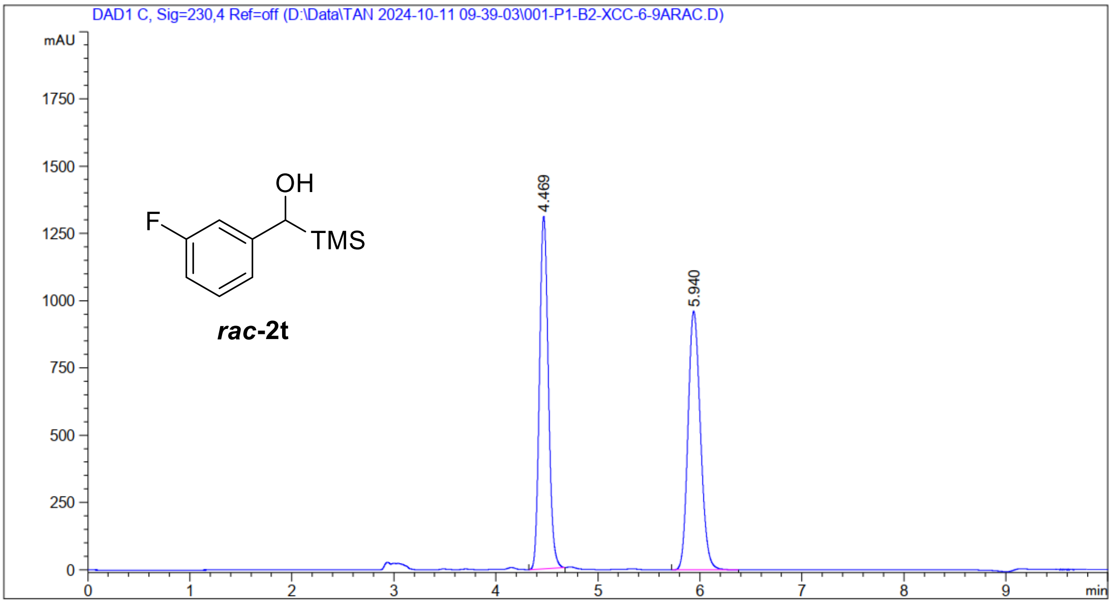

Peak #	RetTime [min]	Type	Width [min]	Area [mAU*s]	Height [mAU]	Area %
1	4.469	BB	0.0943	7909.77246	1310.51721	49.3626
2	5.940	BB	0.1298	8114.04150	961.45618	50.6374

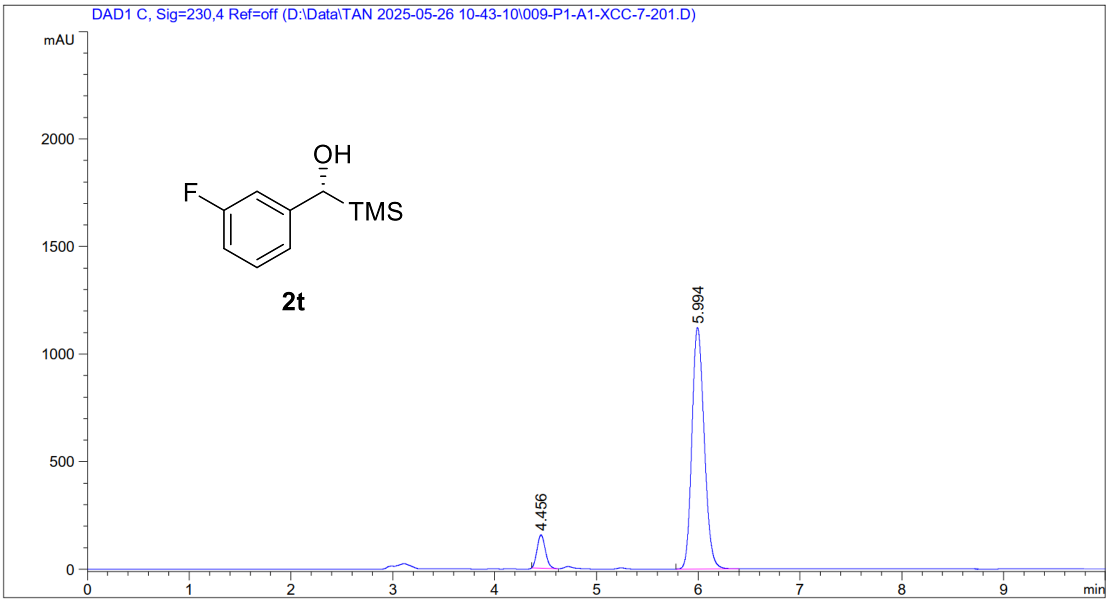

Peak #	RetTime [min]	Type	Width [min]	Area [mAU*s]	Height [mAU]	Area %
1	4.456	BB	0.0807	862.67358	153.76422	8.3877
2	5.994	BB	0.1265	9422.27930	1122.94714	91.6123

(*R*)-(4-Chlorophenyl)(trimethylsilyl)methanol (2v)

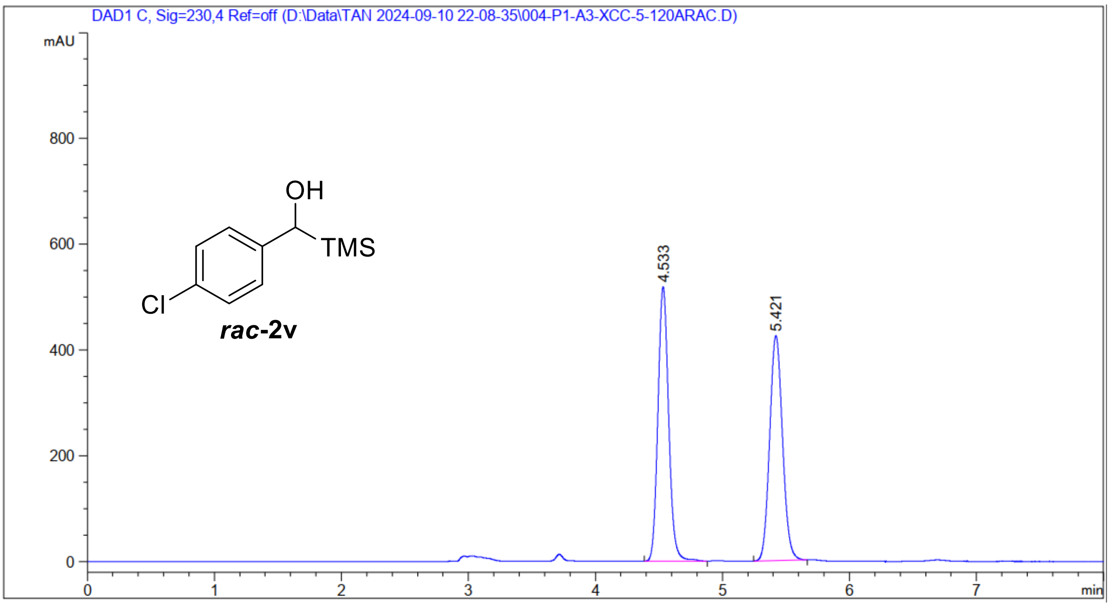

Peak #	RetTime [min]	Type	Width [min]	Area [mAU*s]	Height [mAU]	Area %
1	4.533	BB	0.0901	3038.57935	518.56390	50.4330
2	5.421	BB	0.1076	2986.39868	425.77988	49.5670

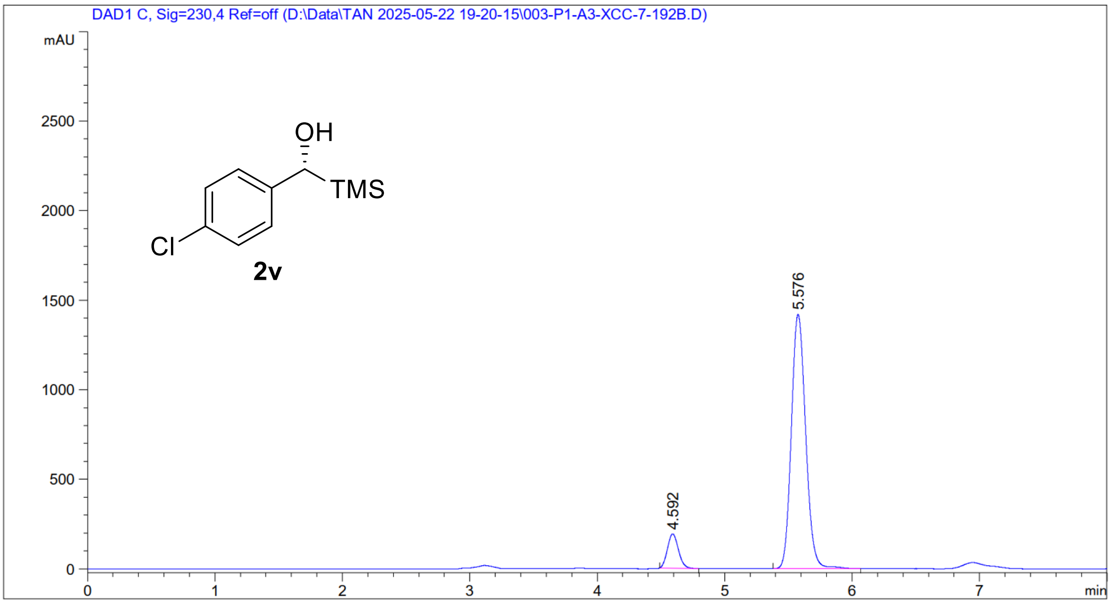

Peak #	RetTime [min]	Type	Width [min]	Area [mAU*s]	Height [mAU]	Area %
1	4.592	BB	0.0867	1140.41626	191.18417	9.3320
2	5.576	BB	0.1183	1.10801e4	1419.71338	90.6680

(*R*)-(4-Bromophenyl)(trimethylsilyl)methanol (2w)

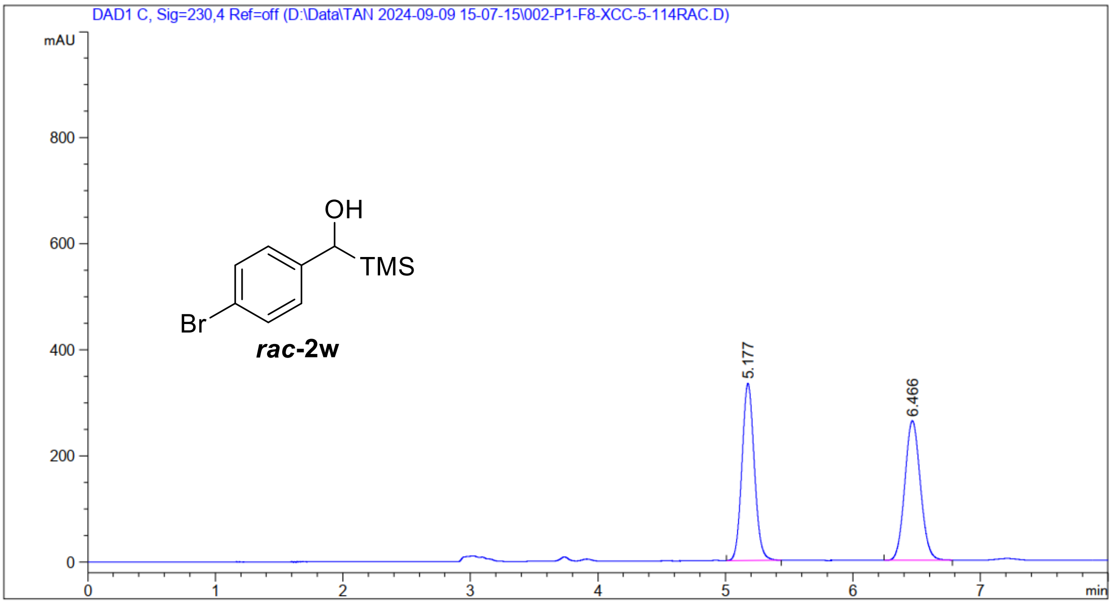

Peak #	RetTime [min]	Type	Width [min]	Area [mAU*s]	Height [mAU]	Area %
1	5.177	BB	0.1052	2250.65283	333.73514	50.0815
2	6.466	BB	0.1277	2243.32886	263.29794	49.9185

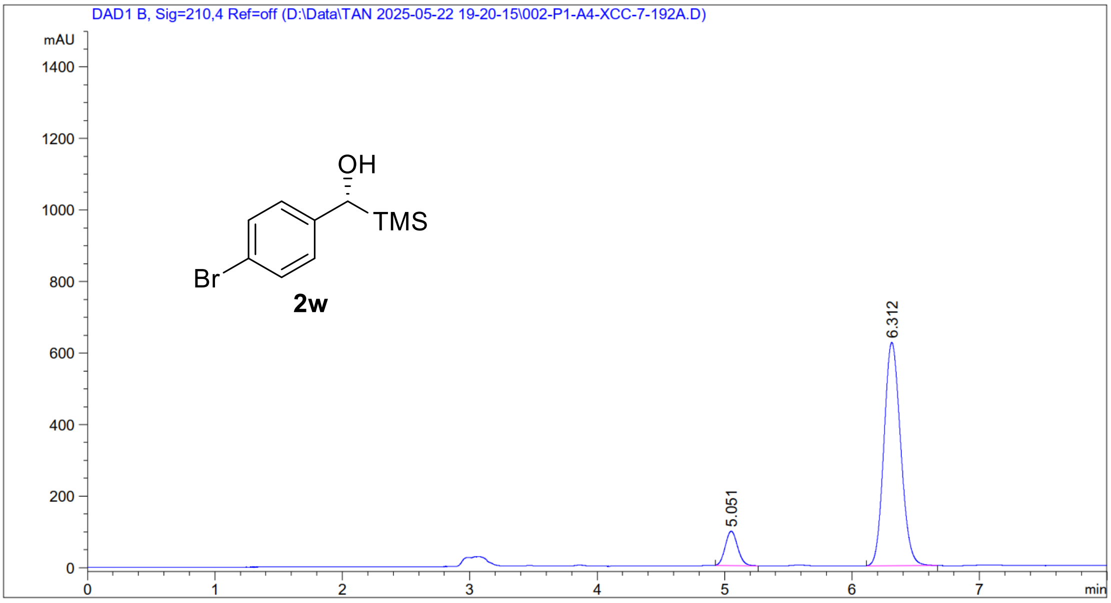

Peak #	RetTime [min]	Type	Width [min]	Area [mAU*s]	Height [mAU]	Area %
1	5.051	BB	0.0871	645.39911	96.00502	10.1987
2	6.312	BB	0.1163	5682.82129	624.58990	89.8013

(*R*)-(3-Chlorophenyl)(trimethylsilyl)methanol (2x)

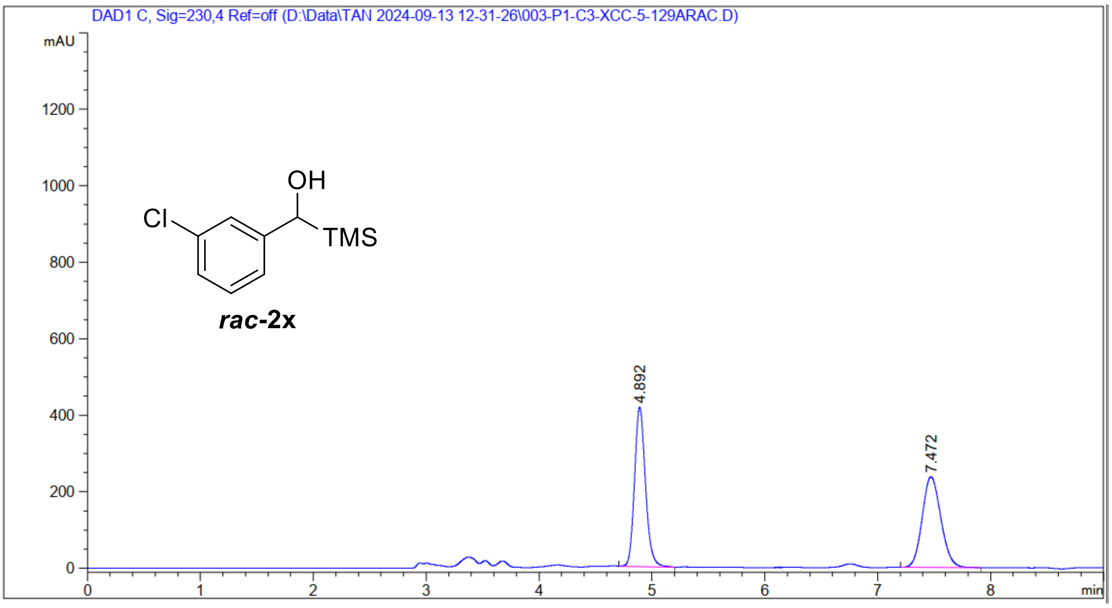

Peak #	RetTime [min]	Type	Width [min]	Area [mAU*s]	Height [mAU]	Area %
1	4.892	BB	0.1037	2799.17603	417.11002	50.7461
2	7.472	BB	0.1712	2716.86670	237.10440	49.2539

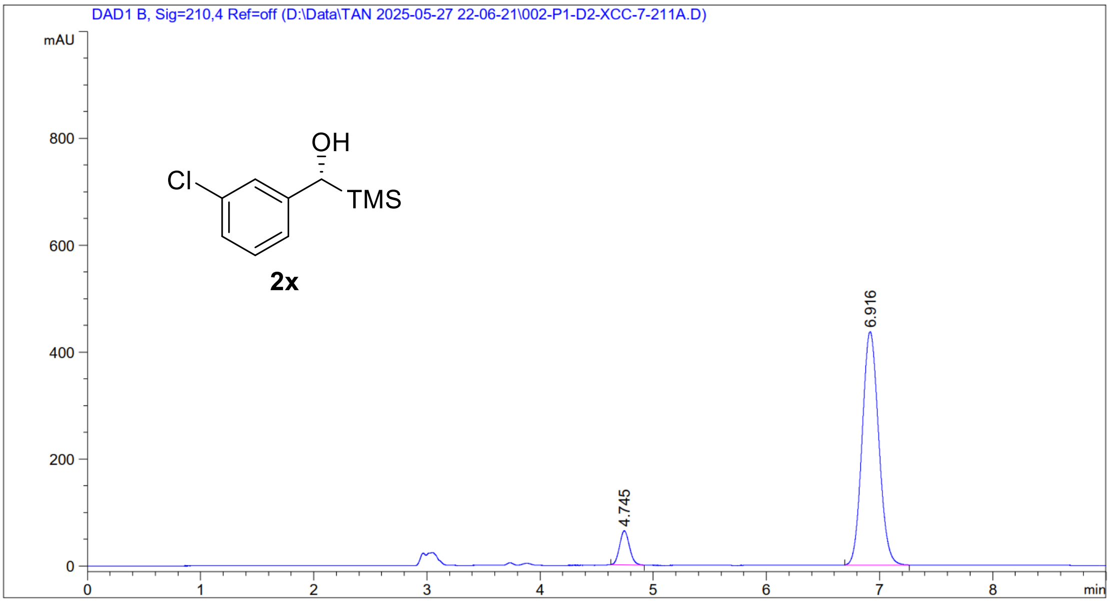

Peak #	RetTime [min]	Type	Width [min]	Area [mAU*s]	Height [mAU]	Area %
1	4.745	BB	0.0794	384.65152	63.47871	7.9966
2	6.916	BB	0.1205	4425.55176	436.45630	92.0034

(*S*)-3-Phenyl-1-(triethylsilyl)propan-1-ol (4a)

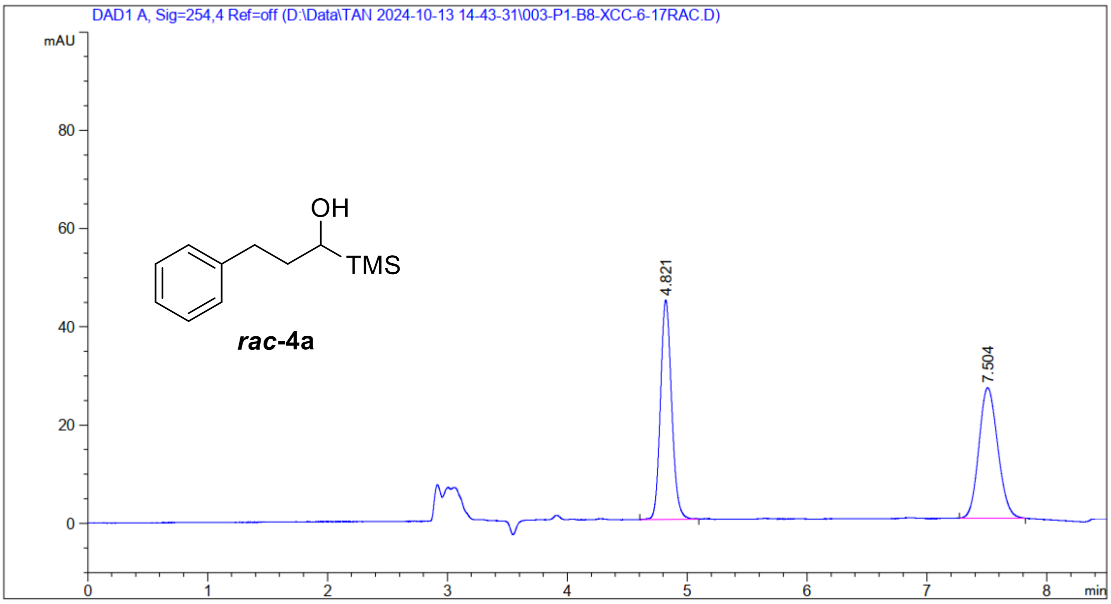

Peak #	RetTime [min]	Type	Width [min]	Area [mAU*s]	Height [mAU]	Area %
1	4.821	BV R	0.0900	289.08710	44.68517	49.9324
2	7.504	BB	0.1296	289.87024	26.54908	50.0676

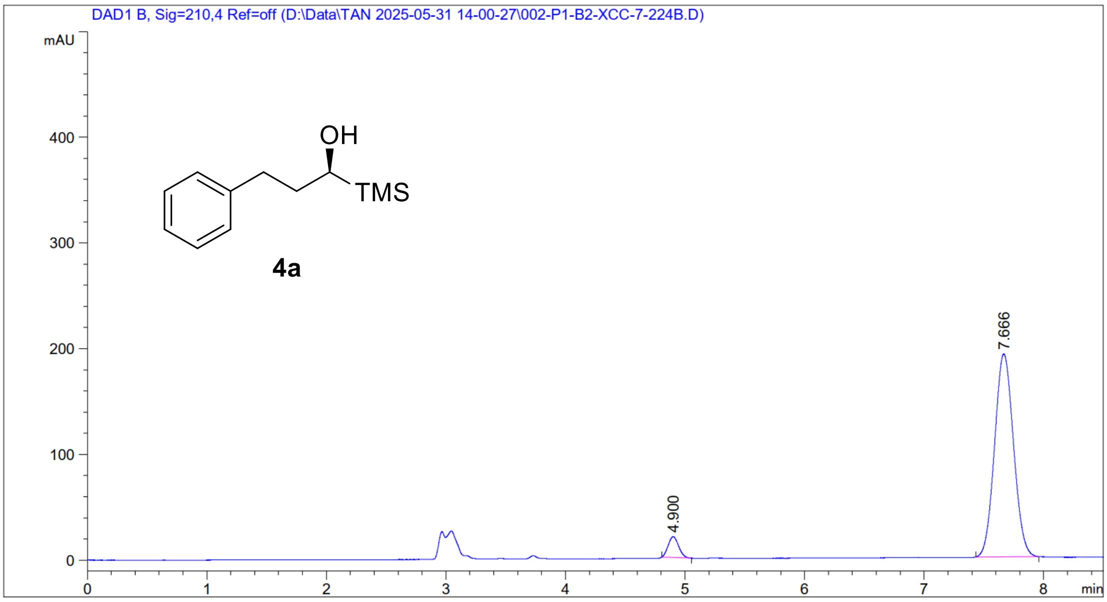

Peak #	RetTime [min]	Type	Width [min]	Area [mAU*s]	Height [mAU]	Area %
1	4.900	BB	0.0720	119.60882	19.71880	5.3616
2	7.666	BB	0.1300	2111.23730	192.03357	94.6384

(*S*)-3-(1,3-Dioxolan-2-yl)-1-(trimethylsilyl)propan-1-ol (4i)

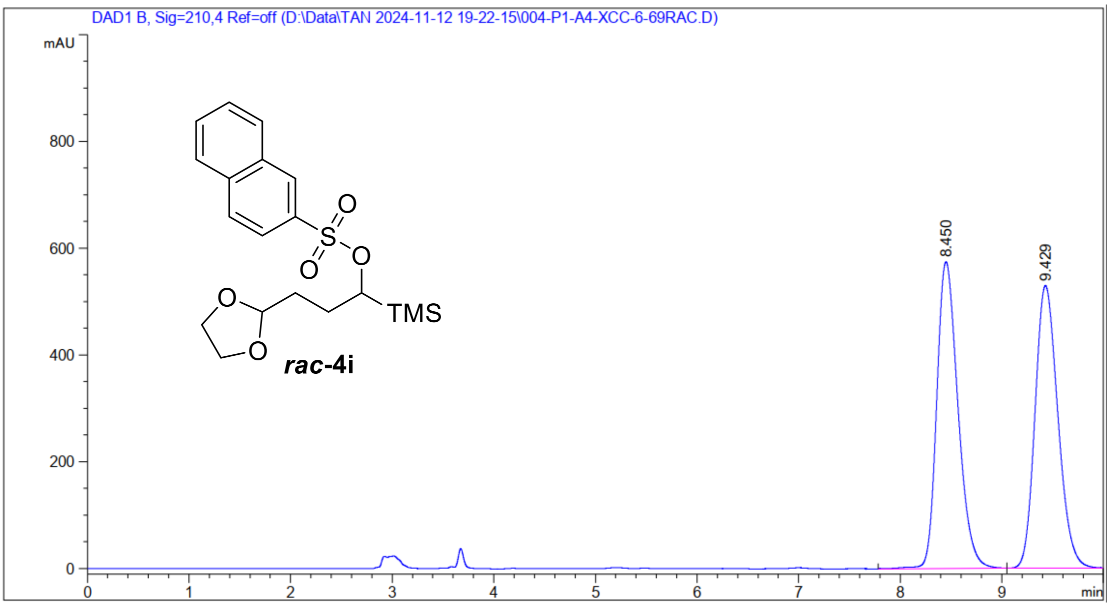

Peak #	RetTime [min]	Type	Width [min]	Area [mAU*s]	Height [mAU]	Area %
1	8.450	BB	0.2208	8311.30371	574.42340	50.1786
2	9.429	BBA	0.2345	8252.15527	528.91815	49.8214

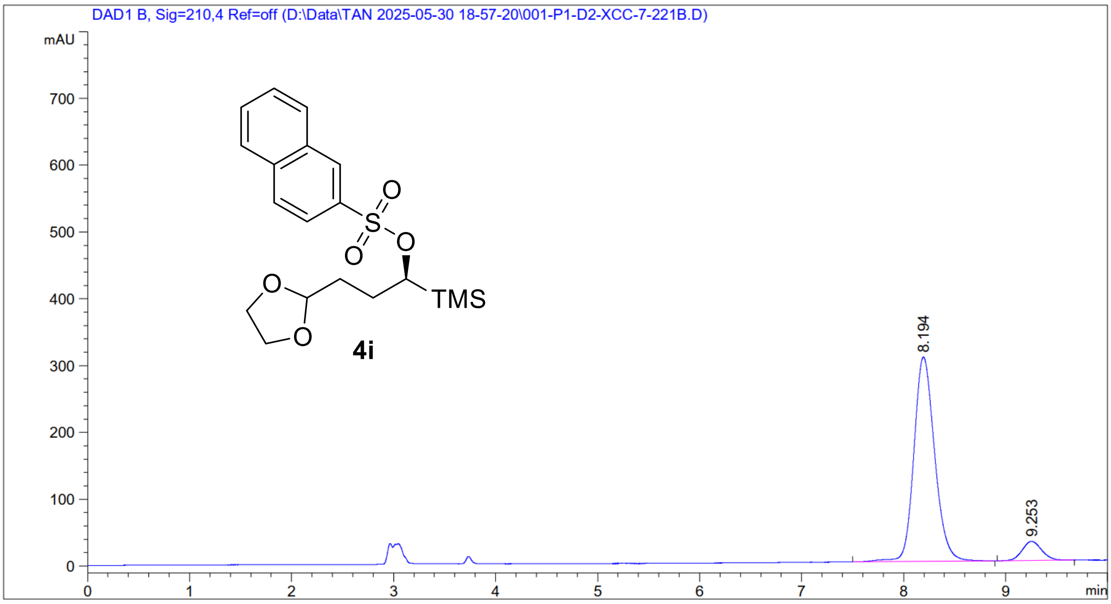

Peak #	RetTime [min]	Type	Width [min]	Area [mAU*s]	Height [mAU]	Area %
1	8.194	BV R	0.2097	4332.25000	305.97113	91.8101
2	9.253	BB	0.1680	386.45898	28.71489	8.1899

(*S*,*E*)-3-Phenyl-1-(trimethylsilyl)prop-2-en-1-ol (4m)

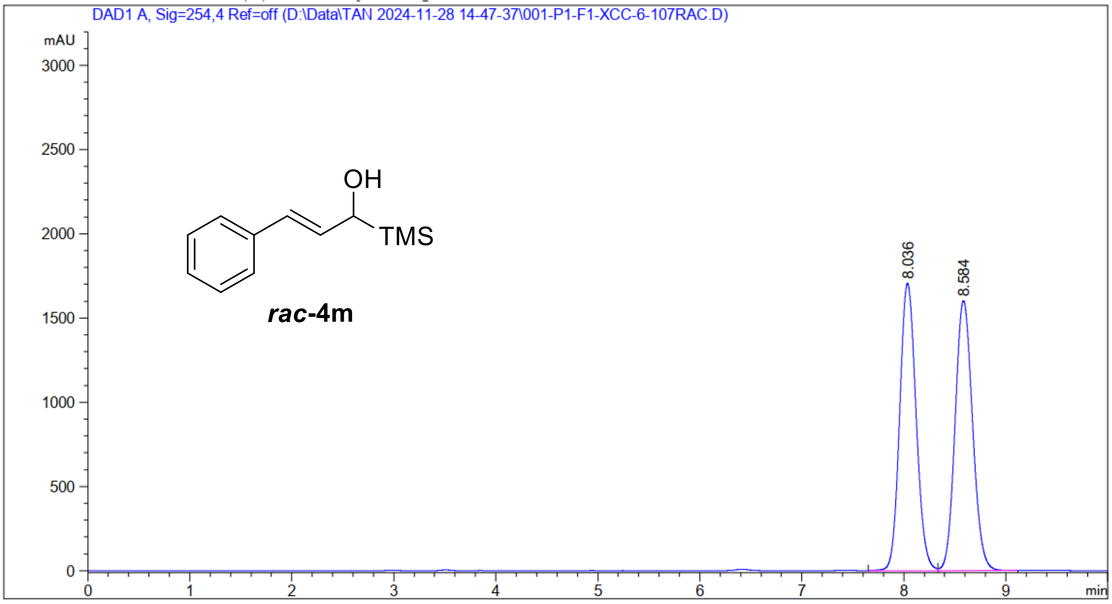

Peak #	RetTime [min]	Type	Width [min]	Area [mAU*s]	Height [mAU]	Area %
1	8.036	BV	0.1741	1.90192e4	1705.01892	49.9260
2	8.584	VB	0.1860	1.90756e4	1601.52979	50.0740

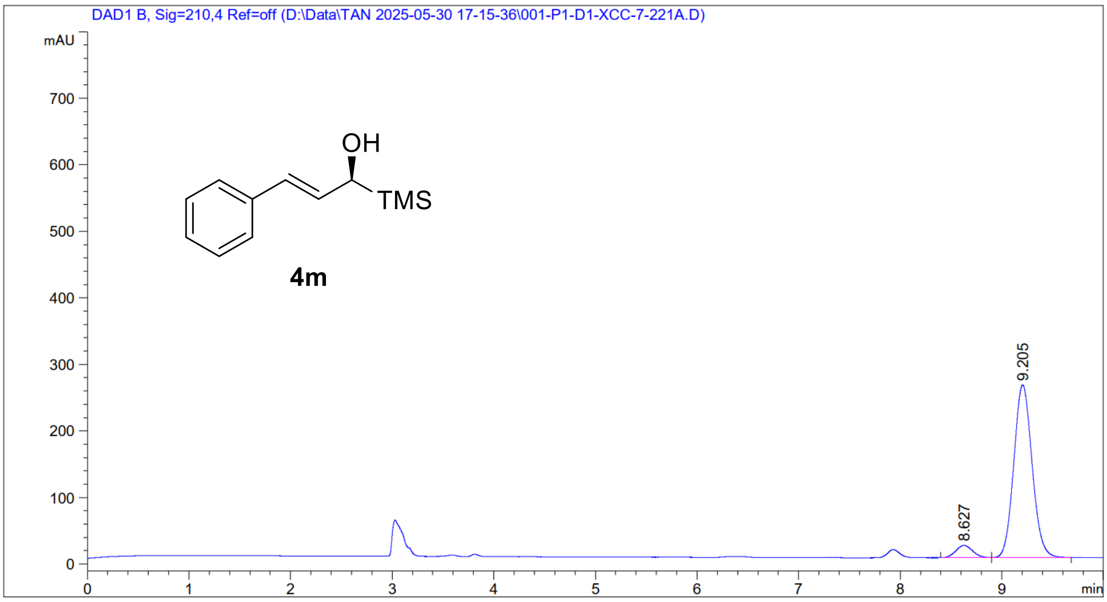

Peak #	RetTime [min]	Type	Width [min]	Area [mAU*s]	Height [mAU]	Area %
1	8.627	BV	0.1786	213.29449	18.48726	6.1570
2	9.205	VB	0.1931	3250.94019	259.64172	93.8430

XIV. References

1 Vaggu, R., Thadem, N., Rajesh, M., Grée, R. & Das, S. Acylsilane Directed Rh-Catalyzed Arene C–H Alkylation with Maleimides and Visible-Light-Induced Siloxycarbene-Amide Cyclization: [3 + 2] Carbo-Annulation in Ru Catalysis. *Org. Lett.* **25**, 2594-2599 (2023).

2 Fan, Z., Yi, Y., Chen, S. & Xi, C. Visible-Light-Induced Catalyst-Free Carboxylation of Acylsilanes with Carbon Dioxide. *Org. Lett.* **23**, 2303-2307 (2021).

3 Reimler, J. & Studer, A. Visible-Light Mediated Tryptophan Modification in Oligopeptides Employing Acylsilanes. *Chem. Eur. J.* **27**, 15392-15395 (2021).

4 Yang, X. *et al.* Photoinduced regioselective trifluoroalkylation of ketene dithioacetals with CF_3SO_2Na. *Org. Chem. Front.* **11**, 5061-5066 (2024).

5 Patel, S. S., Gupta, S. & Tripathi, C. B. Organocatalyzed Hydroacylation of Enones by Photosensitization of Acyl Silanes. *Chem. Eur. J.* **19**, e202400240 (2024).

6 Dharavath, P., Vaggu, R., Manda, R., Grée, R. & Das, S. Visible-Light-Induced Insertion of Siloxycarbene into Amide N–H Bonds: Synthesis of Carbinolamides from Acylsilanes and Amides. *J. Org. Chem.* **90**, 1727-1732 (2025).

7 Xie, Z.-Y., Li, Q.-Q., Liu, Y., Cai, B.-G. & Xuan, J. Photoinduced Asymmetric Formal Siloxycarbene Insertion into sp3 C–H Bonds Enabled by Chiral Phosphoric Acid. *Org. Lett.* **26**, 5827-5832 (2024).

8 Saleem, M., Ratwan, A., Yamini, P. & Yadagiri, D. Visible-Light-Induced Siloxycarbene Addition to N═N of Azodicarboxylates: Synthesis of Acyl Hydrazides from Acylsilanes. *Org. Lett.* **26**, 2039-2044 (2024).

9 Li, Y. *et al.* Light-Dependent Amide or Thioamide Formation of Acylsilanes with Amines using Elemental Sulfur. *Chem. Eur. J.* **31**, e202404555 (2025).

10 Masuda, R., Anami, Y. & Kusama, H. Umpolung Synthesis of Selenoesters and Telluroesters via the Photoinduced Coupling of Acylsilanes with Electrophilic Chalcogen Reagents. *Org. Lett.* **26**, 8011-8016 (2024).

11 Saleem, M., Abhishek, P. & Yadagiri, D. Light-Induced Reactivity of Nucleophilic Siloxycarbene with Heterocumulenes: Synthesis of α-Ketoamides, Hydantoins, Oxoacetamidines, and Amides. *Org. Lett.* **26**, 10291-10298 (2024).

12 Priebbenow, D. L., Pilkington, R. L., Hearn, K. N. & Polyzos, A. Fluorinated Ketones as Trapping Reagents for Visible-Light-Induced Singlet Nucleophilic Carbenes. *Org. Lett.* **23**, 2783-2789 (2021).

13 Lee, S. & Shin, I. Reversed-Polarity Synthesis of N-Sulfonyl Ketimines with Imidoylsilanes and Diaryliodonium Salts via Palladium-Catalyzed Reactions. *J. Org. Chem.* **87**, 6552-6561 (2022).

14 Yamamoto, K., Hayashi, A., Suzuki, S. & Tsuji, J. Preparation of substituted benzoyltrimethylsilanes and -germanes by the reaction of benzoyl chlorides with hexamethyldisilane or -digermane in the presence of palladium complexes as catalysts. *Organometallics* **6**, 974-979 (1987).

15 Nagy, A., Collard, L., Indukuri, K., Leyssens, T. & Riant, O. Enantio-, Regio-

and Chemoselective Copper-Catalyzed 1,2-Hydroborylation of Acylsilanes. *Chem. Eur. J.* **25**, 8705-8708 (2019).

16 Chandrasekaran, R., Pulikkottil, F. T., Elama, K. S. & Rasappan, R. Direct synthesis and applications of solid silylzinc reagents. *Chem. Sci.* **12**, 15719-15726 (2021).

17 Capaldo, L., Riccardi, R., Ravelli, D. & Fagnoni, M. Acyl Radicals from Acylsilanes: Photoredox-Catalyzed Synthesis of Unsymmetrical Ketones. *ACS Catal.* **8**, 304-309 (2018).

18 Miller, J. A. & Zweifel, G. [(Trimethylsilyl)acetyl]trimethylsilane, a versatile synthon for stereoselective syntheses of functionalized trisubstituted olefins. *J. Am. Chem. Soc.* **103**, 6217-6219 (1981).

19 Asokan, K., Zahir Hussain, A. & Ilangovan, A. Efficient amidation of weak amines: synthesis, chiral separation by SFC, and antimicrobial activity of N-(9,10-dioxo-9,10-dihydroanthracen-1-yl) carboxamide. *Org. Biomol. Chem.* **22**, 309-319 (2024).

20 Ying, W. & Herndon, J. W. Total Synthesis of (+)-Antofine and (–)-Cryptopleurine. *E. J. Org. Chem.* **2013**, 3112-3122 (2013).

21 Woodring, J. L. *et al.* Series of Alkynyl-Substituted Thienopyrimidines as Inhibitors of Protozoan Parasite Proliferation. *ACS Med. Chem. Lett.* **9**, 996-1001 (2018).

22 Han, F. *et al.* Electroreductive Cross-Coupling between Aromatic Aldehydes and Chlorosilanes Enabling the Synthesis of α-Silyl Alcohols. *Org.*

Lett. **26**, 7037-7042 (2024).

23 Mitsui, A., Nagao, K. & Ohmiya, H. Catalytic Reductive Cross-Coupling between Aromatic Aldehydes and Arylnitriles. *Chem. Eur. J.* **27**, 7094-7098 (2021).

24 Hwu, J. R., Tsay, S.-C., Wang, N. & Hakimelahi, G. H. Interconversions among .alpha.-(Trimethylsilyl)alkoxides, .alpha.-Trimethylsiloxy Carbanions, and Carbonyl Compounds Accompanied by the Trimethylsilyl Anion. *Organometallics* **13**, 2461-2466 (1994).

25 Huckins, J. R. & Rychnovsky, S. D. Synthesis of Optically Pure Arylsilylcarbinols and Their Use as Chiral Auxiliaries in Oxacarbenium Ion Reactions. *J. Org. Chem.* **68** (2003).

26 Blanc, R., Commeiras, L. & Parrain, J.-L. N-Heterocyclic Carbene-Mediated Organocatalytic Transfer of Tin onto Aldehydes: New Access to *α*-Silyloxyalkylstannanes and γ-Silyloxyallylstannanes. *Adv. Synth. Catal.* **352**, 661-666 (2010).

27 Schwarzwalder, G. M., Matier, C. D. & Fu, G. C. Enantioconvergent Cross-Couplings of Alkyl Electrophiles: The Catalytic Asymmetric Synthesis of Organosilanes. *Angew. Chem., Int. Ed.* **58**, 3571-3574 (2019).

28 Bhat, N. G. & Villanueva, M. A. Facile hydroboration of (Z)-1-trimethylsilyl-1-alkenes with dichloroborane–dioxane complex: An easy access to gem-dimetalloalkanes containing boron and silicon. *J. Org. Chem.* **691**, 1298-1300 (2006).

29 Yi, H., Mao, W. & Oestreich, M. Enantioselective Construction of α-Chiral Silanes by Nickel-Catalyzed C(sp3)−C(sp3) Cross-Coupling. *Angew. Chem., Int. Ed.* **58**, 3575-3578 (2019).

30 Salvado, O. & Fernández, E. A modular olefination reaction between aldehydes and diborylsilylmethide lithium salts. *Chem. Commun.* **57**, 6300-6303 (2021).

31 Cossrow, J. & Rychnovsky, S. D. Optically Pure α-(Trimethylsilyl)benzyl Alcohol: A Practical Chiral Auxiliary for Oxocarbenium Ion Reactions. *Org. Lett.* **4**, 147-150 (2002).

32 Frisch, M. J.; Trucks, G. W.; Schlegel, H. B.; Scuseria, G. E.; Robb, M. A.; Cheeseman, J. R.; Scalmani, G.; Barone, V.; Petersson, G. A.; Nakatsuji, H.; Li, X.; Caricato, M.; Marenich, A. V.; Bloino, J.; Janesko, B. G.; Gomperts, R.; Mennucci, B.; Hratchian, H. P.; Ortiz, J. V.; Izmaylov, A. F.; Sonnenberg, J. L.; Williams; Ding, F.; Lipparini, F.; Egidi, F.; Goings, J.; Peng, B.; Petrone, A.; Henderson, T.; Ranasinghe, D.; Zakrzewski, V. G.; Gao, J.; Rega, N.; Zheng, G.; Liang, W.; Hada, M.; Ehara, M.; Toyota, K.; Fukuda, R.; Hasegawa, J.; Ishida, M.; Nakajima, T.; Honda, Y.; Kitao, O.; Nakai, H.; Vreven, T.; Throssell, K.; Montgomery Jr., J. A.; Peralta, J. E.; Ogliaro, F.; Bearpark, M. J.; Heyd, J. J.; Brothers, E. N.; Kudin, K. N.; Staroverov, V. N.; Keith, T. A.; Kobayashi, R.; Normand, J.; Raghavachari, K.; Rendell, A. P.; Burant, J. C.; Iyengar, S. S.; Tomasi, J.; Cossi, M.; Millam, J. M.; Klene, M.;

Adamo, C.; Cammi, R.; Ochterski, J. W.; Martin, R. L.; Morokuma, K.; Farkas, O.; Foresman, J. B.; Fox, D. J. Gaussian 16 Rev. C.01 (Wallingford, CT, 2016).

33 Chai, J.-D. & Head-Gordon, M. Long-range corrected hybrid density functionals with damped atom–atom dispersion corrections. *Phys. Chem. Chem. Phys.* **10** (2008).

34 Marenich, A. V., Cramer, C. J. & Truhlar, D. G. Universal Solvation Model Based on Solute Electron Density and on a Continuum Model of the Solvent Defined by the Bulk Dielectric Constant and Atomic Surface Tensions. *J. Phys. Chem. B.* **113**, 6378-6396 (2009).

35 Lu, T. & Chen, F. Multiwfn: A multifunctional wavefunction analyzer. *J. Comput. Chem.* **33**, 580-592 (2012).
