# Supplementary material for: Rationally designed Fe-cyclopentadienone with unique orientations for efficient asymmetric hydrogenation of acylsilanes
Source: Nat Commun. 2025 Dec 26;17:1167. doi: 10.1038/s41467-025-67933-9 (PMC12858946; doi:10.1038/s41467-025-67933-9)
Supplement: Supplementary file 2 — Description of Additional Supplementary Files [file 41467_2025_67933_MOESM2_ESM.pdf]

Description of Additional Supplementary Files

File Name: **Supplementary Data 1**.

Description: Cartesian coordinates of the optimized geometries.
